# Genome organization of epidemic *Acinetobacter baumannii *strains

**DOI:** 10.1186/1471-2180-11-224

**Published:** 2011-10-10

**Authors:** Pier Paolo Di Nocera, Francesco Rocco, Maria Giannouli, Maria Triassi, Raffaele Zarrilli

**Affiliations:** 1 Dipartimento di Biologia e Patologia Cellulare e Molecolare, Università Federico II, Via S. Pansini 5, 80131 Napoli, Italy; 2 Dipartimento di Scienze Mediche Preventive, Sezione di Igiene, Università Federico II, Via S. Pansini 5, 80131 Napoli, Italy

## Abstract

**Background:**

*Acinetobacter baumannii *is an opportunistic pathogen responsible for hospital-acquired infections. *A. baumannii *epidemics described world-wide were caused by few genotypic clusters of strains. The occurrence of epidemics caused by multi-drug resistant strains assigned to novel genotypes have been reported over the last few years.

**Results:**

In the present study, we compared whole genome sequences of three *A. baumannii *strains assigned to genotypes ST2, ST25 and ST78, representative of the most frequent genotypes responsible for epidemics in several Mediterranean hospitals, and four complete genome sequences of *A. baumannii *strains assigned to genotypes ST1, ST2 and ST77. Comparative genome analysis showed extensive synteny and identified 3068 coding regions which are conserved, at the same chromosomal position, in all *A. baumannii *genomes. Genome alignments also identified 63 DNA regions, ranging in size from 4 o 126 kb, all defined as genomic islands, which were present in some genomes, but were either missing or replaced by non-homologous DNA sequences in others. Some islands are involved in resistance to drugs and metals, others carry genes encoding surface proteins or enzymes involved in specific metabolic pathways, and others correspond to prophage-like elements. Accessory DNA regions encode 12 to 19% of the potential gene products of the analyzed strains. The analysis of a collection of epidemic *A. baumannii *strains showed that some islands were restricted to specific genotypes.

**Conclusion:**

The definition of the genome components of *A. baumannii *provides a scaffold to rapidly evaluate the genomic organization of novel clinical *A. baumannii *isolates. Changes in island profiling will be useful in genomic epidemiology of *A. baumannii *population.

## Background

The genus *Acinetobacter *comprises 26 species with valid names and nine genomic species with provisional designations that were defined by DNA-DNA hybridization. *Acinetobacter baumannii*, *A. pittii *and *A. nosocomialis *are the three species more frequently associated with human diseases [1-3]. *A. baumannii *is the species that is more frequently isolated in hospitalized patients, especially in intensive-care-unit (ICU) wards. The capability to survive in dry conditions and resistance to disinfectants and antimicrobial agents contribute to the selection of *A. baumannii *in the hospital setting [1,2].

Epidemics caused by multidrug-resistant (MDR) strains of *A. baumannii *were reported in several hospitals worldwide and shown to be caused by *A. baumannii *strains resistant to all classes of antimicrobials including carbapenems, exhibiting variable resistance to rifampicin and tigecycline, but still susceptible to colistin [2,4]. Outbreaks were caused by clusters of highly similar *A. baumannii *strains that were assigned by several genotypic methods to three main international clonal lineages initially named European clones I, II and III [1,2,4-6], and now are referred to as international clones I, II and III, respectively [7,8]. The predominance of international clone II lineage world-wide and the occurrence of hospital outbreaks caused by MDR strains belonging to novel genotypes not related to the three main clonal complexes have been reported during the last few years [4,8-10].

We have recently reported [11] the draft genome sequences of three *A. baumannii *strains, 3990, 4190 and 3909, respectively assigned to ST (sequence types) 2, 25 and 78, which are representative of the most frequent genotypes responsible for epidemics occurred in Mediterranean hospitals [9]. Here we compare the genomes of the 3990, 4190 and 3909 strains and the genomes of four wholly sequenced MDR *A. baumannii *strains, two assigned to ST1, one each to ST2 and ST77. Data helped to define core and auxiliary genome components of the *A. baumannii *genomes.

## Results

### Features of the genome of ST2 3990, ST25 4190 and ST78 3909 strains

The draft genome sequences of the ST2 3990, ST25 4190 and ST78 3909 strains, isolated during cross-transmission episodes occurred at the Monaldi Hospital, Naples, Italy between 2006 and 2009, comprised 4,015,011 bases, 4,032,291 bases and 3,954,832 bases, and generated 3,806, 3,910 and 3,721 protein coding sequences by automated annotation against *A. baumannii *AB0057 genome, respectively [11]. The ST25 4190 strain contained two plasmids, p1-ABST25 and p2-ABST25, that both carry one copy of the carbapenem-hydrolyzing oxacillinase (CHDL) *bla*_OXA-72 _a gene which encodes a protein identical but for a single amino-acid substitution to the product of the *bla*_OXA-24 _gene. The ST2 3990 strain contained also two plasmids, p1-ABST2 carrying a complete *tra *locus, and p2-ABST2 carrying one copy of the CHDL *bla*_OXA-58 _gene. p1-ABST2 and p2-ABST2 were homologous to plasmids pACICU2 and pACICU1 identified in the ST2 ACICU strain [12], respectively. While p1-ABST2 and pACICU2 are almost identical, p2-ABST2 shares only two third of the coding sequences with pACICU1. The plasmid p1-ABST78 identified in the ST78 3909 strain shares approximately 80% of the coding sequences, including the *bla*_OXA-58 _gene, with plasmid pACICU1 (Additional files 1 and 2). The different plasmids were classified using the PCR-typing procedure recently described [13]. A conserved scaffold that includes four/five direct perfect repeats that can be defined as "iterons", and the gene encoding the replicase repAci1 belonging to the Rep-3 superfamily and assigned to the GR2 homology group, was found in plasmids pACICU1, p2ABST2, p2ABST25 and p1ABST78. The repAciX replicase (Rep-3 superfamily, GR10 homology group) is encoded by plasmids pACICU1 and p2ABST2, the Aci6 replicase (GR6 homology group) by pACICU2 and p1ABST2 plasmids. A protein identical to the replicase encoded by plasmid pMMA2 carrying the *bla*_OXA-24 _gene [14], is encoded by p1ABST25. While sharing common sequences, all plasmids exhibited a mosaic genetic structure that might have been generated by multiple recombination events. The hypothetical gene products encoded by the plasmids found in the *A. baumannii *strains 3990, 3909 and 4190 are listed in Additional file 2.

### The *A. baumannii *chromosome

Making use of the Mauve software [15], the proteins putatively encoded by the draft genomes of the *A. baumannii *strains 3990, 3909 and 4190 [11] were compared to the ORFs encoded by the wholly sequenced genomes of the *A. baumannii *AB0057 and AYE strains assigned to ST1, ACICU strain assigned to ST2, ATCC17978 strain assigned to ST77 [12,16-18].

*A. baumannii *genomes exhibit extensive synteny. Sequence comparisons revealed that 3068 coding regions are conserved, at the same chromosomal position, in the compared *A. baumannii *genomes. A file including all conserved gene products is available upon request. Genes encoding proteins shown or hypothesized to be important for pathogenicity are conserved in the analyzed strains at the same relative chromosomal position (Table 1). The set includes OmpA, the outer membrane protein which has role in biofilm formation [19] and induces, when secreted, death of epithelial and dendritic cells [20], the DD-endopeptidase, which contributes to the resistance of *A. baumannii *to bactericidal activity presumably by remodelling the cell surface [21], phospholipase D, an enzyme crucial for proliferation in human serum [22], proteins involved in the formation of capsule [23], type I pili [24], and iron metabolism [25]. According to the published annotation, OmpA, DD-endopeptidase, phospholipase D, and many other deduced gene products are smaller in ATCC 17978 as compared to their orthologs. Size differences do not denote allelic variation, but are determined by the criteria adopted to select the initiating methionine in ATCC17978 ORFs.

**Table 1:** Gene products involved in pathogenicity in *A.baumannii *genomes

Gene products					Strains		
	
	AB0057	AYE	3990	ACICU	4190	ATCC17978	3909
**capsule formation**							
tyrosine kinase Ptk	91	3818	936	71	3295	49	2600
Tyrosine phosphatase Ptp	92	3817	935	72	3296	50	2601

**type I pili formation**							
CsuE	2565	1324	787	2414	3382	2213	744
CsuD	2566	1323	786	2415	3383	2214	745
CsuC	2567	1322	785	2416	3384	2215	746
CsuB	2568	1321	784	2417	3385	2216	747
CsuA	2569	1320	783	2418	3386	2217	748
CsuA/B	2570	1319	782	2420	3387	2218	3415

**iron metabolism**							
nonribosomal peptide synthetase BasD	2811	1095	2421	2579	tblastn	2383	1389
nonribosomal peptide synthetase BasC	2812	1094	2420	2580	3813	2384	tblastn
ferric acinetobactin receptor	2813	1093	2419	2581	3814	2385	3376
ferric acinetobactin transport system periplasmic binding protein	2814	1092	2418	2582	3815	2386	3375
ferric acinetobactin transport system ATP-binding protein	2815	1091	2417	2583	3816	2387	3374
ferric acinetobactin transport system permease	2816	1090	2416	2584	3817	2388	3373
ferric acinetobactin transport system permease	2817	1089	2415	2585	3818	2389	3372

**hemin utilization**							
biopolymer transport protein ExbD/TolR	1827	2051	351	1629	227	1063	1994
biopolymer transport protein ExbD/TolR	1828	2050	352	1630	228	1064	1993
biopolymer transport protein	1829	2049	353	1631	229	1065	1992
TonB family protein	1830	2047	354	1632	230, 231	3708*	1991
TonB-dependent receptor	1831	2046	355	1633	232	1606, 1607	1990, 1989
heme-binding protein A	1832	2045	358	1634	234	1608	1987
heme-binding protein A	1833	2044	359	1635	235	1609	1986
Zn-dependent oligopeptidase	1834	2043	360	1636	236	1610	1985
ABC-type dipeptide/oligopeptide/nickel transport system permease component	1835	2042	361	1637	237, 238	1611	1984
ABC-type dipeptide/oligopeptide/nickel transport system permease component	1836	2041	362	1638	239	1612	1983
glutathione import ATP-binding protein GsiA	1837	2040	363	1639	3719	1613	1982

* The asterisk indicates one of the 436 proteins putatively encoded by ATCC17978 not included in the GenBank:NC_009085 file. tblastn refer to unannotated 4190 and 3909 proteins identified by tblastn searches.

Multidrug resistance is a key feature of *A. baumannii *and several genes have a role in establishing a MDR phenotype. Genes encoding efflux pumps and resistance proteins shown or hypothesized [26] to be involved in the process are conserved in all strains. In contrast, genes encoding drug-inactivating and drug-resistant enzymes reside in accessory DNA regions which are present only in some strains (Table 2). Among these, are worth of mention the extended spectrum beta-lactamase *VEB-1 *gene, found in the AYE genome, the *bla*_OXA-20 _class D beta-lactamase gene, found in the ACICU and 3990 genomes, both assigned to ST2 genotype, the CHDL genes *bla*_OXA-23_, found in the AB0057 genome, *bla*_OXA-58_, found in the plasmids of 3990, ACICU and 3909 strains, and *bla*_OXA-72 _found in the plasmids of 4190 strain, respectively. Promoter sequences within flanking insertion sequences likely influence the expression of many of these resistance genes. Interestingly, the majority of the genomes harbour mutations in *gyrA *and/or *par*C genes.

**Table 2:** Antimicrobial resistance gene products encoded by *A.baumannii *genomes

Gene Products				Strains			
	
	AB0057	AYE	3990	ACICU	4190	ATCC17978	3909
Class C β-lactamase	9, 2796	1110	2437	2564	2076	2367	1404

Class A β-lactamase	283 (TEM-1)	-	-	-	-	-	-
	-	3623 (VEB-1)	-	-	-	-	-

Class D β-lactamase	1757 (oxa-69)	2122 (oxa-69)	2827 (oxa-66)	1560 (oxa-66)	63 (oxa-64)	1517 (oxa-95)	1089 (oxa-90)
	-	-	3514 (oxa-20)	0226 (oxa-20)	-	-	-
	0551 (oxa-23)*	-	-	-	-	-	-
	-	-	p2ABST2 (oxa-58)*	pACICU1 (oxa-58)* (2X)	p1ABST25 (oxa-72)*	-	p1ABST78 (oxa-58)*
	-	-	-	-	p2ABST25 (oxa-72)*	-	-

AAC (3)-I aminoglycoside acetyltransferase	291	3573	-	-	-	-	-
AAC (6')-I aminoglycoside acetyltransferase	-	3630	3516	223	-	-	-

APH (3')-I aminoglycoside phosphotransferase	288	3578	-	-	-	-	-
	-	-	3897	1948	560	-	-

ANT (3'')-I aminoglycoside adenylyltransferase	293	3570,3618	-	-	3268	-	-
	171	3739	1641	156	2954	131	2919

Chloramphenicol acetyl transferase	280	3587	-	-	-	-	-
	3104	798	3709	2932	1731	2691	1443

DNA topoisomerase II	3037 [R^1^]	0867 [R^1^]	0747 [R^1^]	2869 [R^1^]	2907 [R^1^]	2626 [S]	0539 [R^1^]
DNA topoisomerase IV	0232 [R^2^]	3679 [R^2^]	1415 [S]	0214 [S]	2382 [R^2^]	-	3413 [R^2^]
RNA polymerase β Subunit	0369 [S]	3489 [S]	2179 [R^3^]	0303 [S]	3155 [S]	0287 [S]	0411 [S]

Dihydropteroate synthase	265, 294	3568,3616,3612	3142	228	-	675	-
	3095	807	3700	2923	2684	2680	1433
Dihydrofolate reductase type 1	-	3644	-	-	-	-	-
Dihydrofolate reductase type 3	540	3315	3351	467	3501	457	403

R, resistant; S, susceptible; R^1 ^81 Ser →Leu; R^2 ^84 Ser → Leu; R ^3 ^535 His → Leu; * carbapenem-hydrolysing class D beta-lactamase; ^+ ^ORFs identified by tBLASTn.

**Figure 1:**
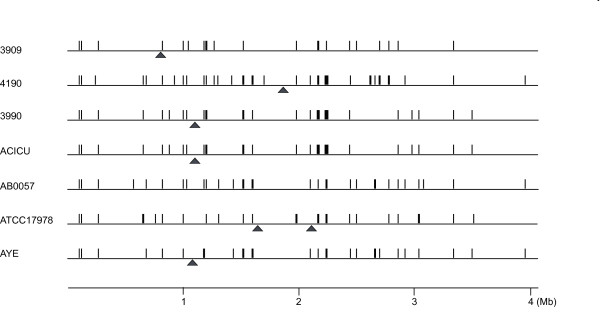
**Comparison of *A. baumannii *genomes**. The seven *A. baumannii *genomes analyzed have been aligned. Accessory regions are denoted by vertical bars. Strain-specific deletions are marked by triangles.

**Figure 2:**
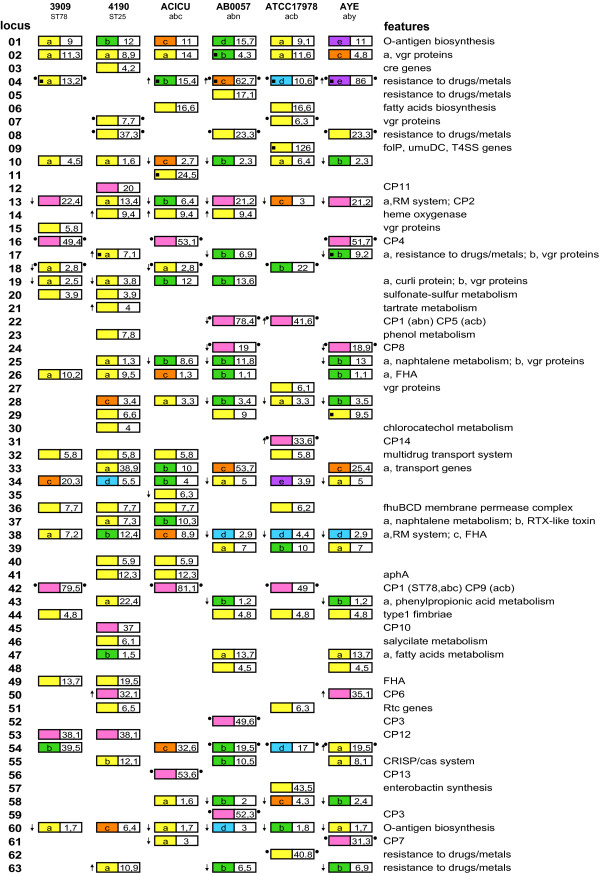
**Variable regions in *A. baumannii *genomes**. A chart of the genomic islands (GEIs) depicted as bars in Figure 1 is displayed. Each line corresponds to a chromosomal locus. Different GEIs inserted at the same locus in different strains are marked by different colours and lower case letters. Sizes of GEIs are given in kb. Black boxes within GEIs denote mobile sequences, down and up arrows to the left indicate that the GEI G+C content is lower than 36% or higher than 42%, respectively. Dots flanking GEIs denote TSDs. The strain names and relative acronyms used throughout the text are given at the top. Acronyms below complete genomes are those used at Kyoto Encyclopaedia of Genes and Genomes (KEGG).

Shared synteny lets to represent the *A. baumannii *chromosomes as ˜4 Mb long DNA segments homologous to each other throughout their lengths (Figure 1). DNA tracts, ranging in size from 4 to 126 kb, are present in one or more strains, but missing or replaced by alternative DNA segments in others (see vertical bars in Figure 1). Some of these regions correspond to DNA sequences earlier suspected to be mobile because found in *A. baumannii *but not in *A. baylyi *DNA or vice versa [17,27]. Specific 15-36 kb regions are missing in all strains but AB0057 (see triangles in Figure 1), and may therefore plausibly correspond to strain-specific deletions. Many of the accessory genomic DNA segments exhibit characteristic features of genomic islands, such as the presence of insertion sequences at one end, a GC content different from the bulk chromosome, insertion within tRNA or non-coding RNA genes, target site duplications (TSDs) at the ends formed upon genome integration [28,29]. For sake of simplicity, all the accessory DNA regions have been called GEnomic Islands (GEIs). GEIs found at the 63 variable loci identified in the *A. baumannii *genomes, and some of their properties, are diagrammatically reported in Figure 2. TSDs flanking GEIs are reported in Additional file 3, and GEI gene products are listed in Additional file 4. In text and figures individual GEIs are referred by the locus number and the strain acronym used in Figure 2. Core and accessory chromosomal DNAs are fully conserved in ACICU and 3990 strains. Because of this, only the ACICU GEIs are shown in Figure 2. In draft genomes some GEIs reside in different contigs. The colinearity of the contigs and the GEI DNA content of the corresponding chromosomal regions were assessed by sequencing PCR products bridging contigs ends.

A close look at *A. baumannii *chromosomes further identified about one hundred DNA regions encoding 1-2 ORFs smaller than 4 kb conserved in one or more strains, but missing, or replaced by non homologous DNA of comparable length, in others. The potential gene products encoded by these smaller accessory regions, that we called *mhrs *(for micro-heterogeneity regions), are reported in Additional file 5.

### Categories of genomic islands

Some islands are strain-specific; others are completely or partially conserved in more than one strain. Non homologous islands are inserted at the same locus in different strains, and some loci are extremely heterogeneous, featuring up to 4-5 alternative islands. Some islands are composite, and changes in their organization among strains are correlated to changes in the number and association of specific DNA segment. Thus, for example, G54_ST78 _can be viewed as made by ABC segments. Segments AB are missing in G54_acb_, segments AC in both G54_abn _and G54_aby_, and segment C is replaced by a shorter DNA segment in G54_acb _(see Additional file 4 for a direct G54 islands comparison). On the basis of the putative gene products, GEIs can be broadly sorted into a few categories. Properties and overall organization of relevant GEIs are below discussed.

### Resistance islands

**Figure 3:**
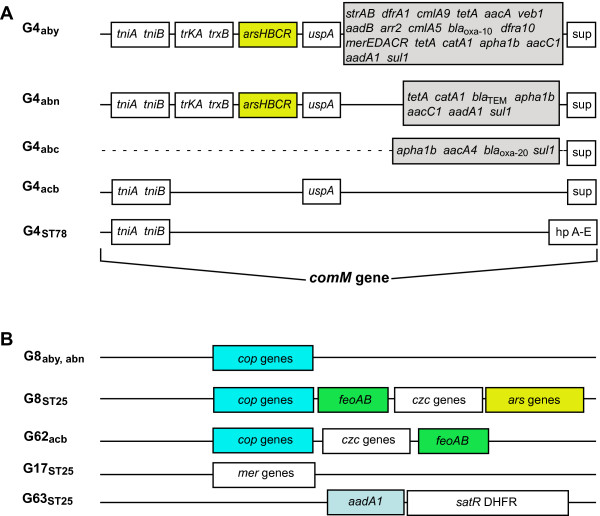
**Resistance gene islands**. **A) **Diagrammatic representation of G4 islands. The structure of the resistance islands and gene symbols are as in reference 30. Grey boxes represent MARR. Deleted DNA in G4_abc _is marked by a dotted line. **B) **Resistance genes in other GEIs.

Many of the accessory drug resistance determinants of Table 2 found in AB0057 and AYE are encoded by genes located within G4_aby_, G4_abn _and G5_abn_, which correspond to the resistance regions previously described as AbaR1, AbaR3, and AbaR4 [16,30], respectively. G4_aby _and G4_abn _are both inserted in the *comM *gene, and result from the association of the 16 kb Tn6019 transposon with multiple antibiotic resistance regions (MARR), which are delimited by Tn6018 elements [30]. Tn6019 features genes involved in transposition (*tniA, tniB*), an arsenate resistance operon, a universal stress protein gene (*uspA*), and a sulphate permease gene (*sup*). MARR are inserted within *uspA *and vary in length and composition [30]. The G4_abc _island of the ACICU genome corresponds to the AbaR2 region [30], which carries few resistance genes and lacks Tn6019 sequences (Figure 3A). G4_ST78 _is similarly inserted in the *comM *gene, and features genes homologous to *tniA *and *tniB *(38-40% identity of the gene products), but lacks resistance genes and encodes a set of hypothetical proteins (Figure 3A). G4 is missing in strain 4190. However, resistance genes are scattered in different GEIs of this strain (Figure 3B). The *aad*A1 (streptomycin 3''-adenylyltransferase) gene, flanked by *satR *(streptothricin acetyltransferase) and *dhfr *(dihydrofolate reductase) genes are found in G63_ST25. _Genes involved in resistance to mercury (*merRCAD *cluster) are located in G17_ST25_, and a 4.5 kb DNA segment containing *feo*AB (ferrous iron transport operon), *czc *(tricomponent proton/cation antiporter efflux system) and *ars *(arsenite transporters) genes are found in G8_ST25_, next to the *cus *(copper resistance) genes conserved in all G8 (Figure 3B). The G62_acb _region also contains *cus*, *feo *and *czc *genes involved in heavy metal resistance. These genes differ in sequence and overall arrangement from G8_ST25 _homologs. This supports the notion that the set of accessory genes had been independently acquired by the strains 4190 and ATCC17978.

Additional resistance genes found in GEIs include an aminoglycoside phosphotransferase gene (G41_ST25_, G41_abc_), a dihydropteroate synthase gene (G9_acb_), and an ABC-type multidrug transport system, conserved in all the G32 islands.

### GEIs encoding surface components and transport systems

GEI-1 and GEI-60 host genes involved in cell envelope. Heterogeneity among *A. baumannii *strains at the level of O-antigen biosynthetic genes was already noticed (16), and is correlated to the presence of alternative glycosylases. The G44 island, present in all strains but ACICU, 3990 and 4190, is a four gene operon involved in the assembly of fimbriae (type I pili) by the chaperone/usher pathway [31]. G44_aby _corresponds to the surface adhesion protein region annotated as Cus1R in the AYE genome [18]. G19_ST25 _and G19_ST78 _are related islands which both carry an operon encoding three hypothetical lipoproteins. Of these, one exhibits homology to CsgG, the key factor in the secretion of curli, the proteinaceous component having a role in host cell adhesion and biofilm formation in many *Enterobacteriaceae *[32]. Purified CsgG forms ring-shaped complexes analogous to those formed by outer membrane channel-forming proteins [32]. The CsgG-like protein, in association with the two co-expressed lipoproteins, may influence the permeability of the outer membrane of *A. baumannii*.

Filamentous haemagglutinin (FHA) is a major virulence factor in *Bordetella pertussis *[33]. *fhaB *and *fhaC *genes, respectively encoding the haemagglutinin and the transporter protein, have been identified in many pathogens [34]. *fhaBC *gene clusters are found at the same loci in strains 4190 and 3909 (islands G26_ST25_, G26_ST78_, G49_ST25 _and G49_ST78_), and strains ACICU and 3990(islands G38_abc _and G38_ST2_). The transporter proteins are highly conserved in the four clusters, whereas FHAs vary in length (1834 to 4812 amino acids), mostly because of changes in the number and organization of body sequence repeats [33]. A 3216 amino acids long calcium binding hemolysin protein, unrelated to FHAs, is encoded by G18_acb_.

Cyclopropane fatty acids (CFA) are phospholipids found in the bacterial membranes in the late exponential and early stationary phases of cell growth [35], which derive from the corresponding unsaturated fatty acid (UFA) phospholipids. The synthesis of CFA is catalyzed by the enzyme CFA synthase, the substitution of a saturated by an unsaturated fatty acid by the enzyme delta-9 acyl-lipid desaturase. CFA synthase and delta-9 acyl-lipid desaturase are both encoded by G47_abn _and G47_aby_.

G33_ST25 _is a large island which encodes four different transport and translocation systems: i) Tat (twin-arginine translocation) proteins, involved in the translocation of folded proteins to the cell envelope or the extracellular space ii) a TonB/ExbBD complex iii) a Opp (oligopeptide transport proteins) complex iv) a sulfur utilization system, made by a FMNH2-dependent sulfonatase and three ABC-type transporters, which resemble the products of the *E. coli ssu *gene cluster [36]. Two unlinked copies of the sulfonatase gene are also present. Genes involved in the capture and intracellular transport of iron are found in different islands. G57_abc _carries a gene cluster involved in the synthesis of the high-affinity siderophore enterobactin. Heme oxygenase is an alternative to siderophores to capture iron from the environment [37]. G14, an island which is conserved in 4190, ACICU and AB0057, carries an operon encoding a heme oxygenase, an outer membrane and a TonB family protein. The presence of a flanking *fecIR *gene cluster suggests that heme internalization may be regulated by the Fec transduction system [38]. The *fhu*BCD genes, which catalyze the internalization of iron III hydroxamate compounds, are located on G36, an island conserve in all strains but AB0057 and AYE.

### Metabolic islands

Many GEIs carry genes encoding proteins involved in specific metabolic pathways. G23_ST25 _carries a *mph *(multi component phenol hydroxylase) gene complex, involved in the conversion of phenol to cathecol, flanked by a sigma54-dependent activator gene. It has been shown that the expression of *mph *gene complex described in *Acinetobacter sp*. PHAE-2 is dependent on the alternative sigma factor RpoN [39]. G37_ST25 _carries *nag *genes, involved in the metabolism of naphthalene. In *Ralstonia *[40], *nag *genes are arranged in two separate clusters, involved in the conversion of naphthalene to gentisate (*nagAGHBFCQED *genes), and gentisate to pyruvate and fumarate (*na*g*IKL *genes), respectively. In G37_ST25 _*na*g*IKL *genes and *nagGH*, encoding the salicylate 5-hydroxylase, are linked, and flanked by benzoate transport genes.

G43_ST25 _carries genes involved in the catabolism of 3HPP (3-hydroxyphenylpropionic acid) and PP (phenylpropionic acid). In *E. coli*, the dioxygenase complex (*hcaEFCD *genes), and the dihydrodiol dehydrogenase (*hcaB *gene) oxidize PP (phenylpropionic acid) and CI (cinnamic acid) to DHPP (2,3-dihydroxyphenylpropionate) and DHCI (2,3-dihydroxycinnamic acid), respectively. These substrates are subsequently converted to citric acid cycle intermediates by the *mhp *genes products [41]. The *hca *and *mhp *genes, separated in *E. coli*, are linked and interspersed with additional genes (see Additional file 4) in G43_ST25_. G21_ST25 _potentially encodes 4 proteins (tartrate dehydratase subunits alpha and beta, a MFS transporter and a transcriptional regulator) possibly involved in the metabolism of tartrate. Proteins exhibiting homology to the dienelactone hydrolase, an enzyme which plays a crucial role in the degradation of chloro-aromatic compounds, are encoded by the islands G30_ST25_, G34_abn _and G34_aby_. G46_ST25 _is made by an operon including the salicylate 1-monooxygenase (*salA*), a benzoate transporter (*benK*) and the *sa*lA regulator (*salR*) genes. A salicylate 1-monooxygenase is also encoded by G25_ST25_. The genes *fabA*, *fabB*, *fa*b*G*, *fabF*, *acpP*, *pslB*, *acsA*, involved in the biosynthesis of fatty acids [35] are conserved in all *A. baumannii *strains, at separate loci. Orthologues of all these genes are clustered in G6_abc _and G6_acb_.

### Phage islands

Many variable genomic regions are relatively large (19 to 82 kb) DNA blocks which potentially encode typical phage products. These regions have all been classified as cryptic prophages (CP; see Figure 2). Three to six CPs were identified in each strain. Six of the different 14 CPs identified are present in two or more strains, the remaining 8 are strain-specific. CPs characteristically carries at one end an integrase gene, and many are sharply defined by flanking TSDs induced upon insertion. CPs are poorly related to each other, and even CPs of the same type differ in size and coding ability. Ten of 14 CPs were assigned to four groups on the basis of sequence homologies (Additional file 6). CPs found at the same locus encode identical or highly homologous (> 80% identity) integrases. CP1 encode different integrases, which are homologous to CP5- or CP9-encoded enzymes. This explains why CP1 and CP5 in AB0057 and ATCC17978 (G22_abn _and G22_acb_, respectively), and CP1 in 3909 and ACICU (G42_ST78 _and G42_abc_), and CP9 in ATCC 17978 (G42_acb_), are inserted at the same locus. CP3 are integrated at different sites of the AB0057 genome (G52_abn _and G59_abn_), but the target in both is an *arg-tRNA *gene.

Remnants of prophage sequences are found in G33_abn _and G33_aby_. These islands share the G33_abc _backbone, but contain also large DNA segments, reiterated in a head-to-tail configuration, in which genes encoding phage and hypothetical proteins are variously interleaved. G33_abn _and G33_aby _hypothetical gene products exhibit poor homology to all CPs gene products, and therefore were not included among CPs.

Phages may acquire ORFs named morons [42] by lateral gene transfer. The PapS reductase (3'-phosphoadenosine 5'-phosphosulfate sulfotransferase) encoded by CP13 (G56_abc_), the toxin-antitoxin (TA) system encoded by CP1 (G42_abc _and G42_ST78_), the proofreading 3'-5' exonuclease epsilon subunit of the DNA polymerase III in the above mentioned CPs, the *umuDC *gene products, which are the components of the error-prone DNA polymerase V, again in CP1 (G22_abn _and G42_ST78_) and CP5 (G22_abc_) can all be considered morons. Not surprisingly, these enzymes are frequently associated with mobile genome elements [43]. Unlinked *umuD *and *umuC *genes are conserved in all *A. baumannii *strains, and an *umuDC *cluster resides on the 64 Kb pACICU2 plasmid.

G9_acb _also contains an *umuDC *cluster. This 126 kb region, found only in the ATCC 17978 strain, is a composite genomic island, carrying at one end a dihydropteroate synthase gene, at the other a DNA mismatch repair enzyme. G9_acb _carries a complete set of type IV secretion system (T4SS) genes, arranged in the same order in which T4SS homologs are found on the 153 Kb plasmid of *Yersinia pseudotuberculosis *IP31758 strain [44]. Because *umuDC *genes are carried by this plasmid, one may hypothesize that raises G9_acb _had been imported from *Yersinia*. In addition, a G9_acb _gene cluster, including an integrase, a DNA helicase and a TrbL/VirB6 conjugal transfer protein is highly homologous to a gene cluster from *Enterobacter cloacae*.

### Additional islands

G3_ST25 _carries a *cre *genes cluster. In *E. coli *the *cre *locus includes a response regulator (*creB*) a sensor kinase (*creC*) and an inner membrane protein (*creD*). The corresponding two-component regulatory system CreB-CreC controls the expression of a variety of genes, among which the *creD *regulator. Overexpression of CreBC causes modification of the envelope, inducing the colicin E2 tolerance phenotype [45].

G51_ST25 _and G51_acb _carry the *rtcA *and *rnt*Z genes, encoding the RNA 3'-terminal phosphate cyclase and the RNAseZ, respectively. The cyclase catalyzes the ATP-dependent conversion of the 3'-phosphate to the 2', 3'-cyclic phosphodiester at the end of various RNA substrates [46]; RNAseZ is responsible for the maturation of the 3'-end of a large family of transfer RNAs [47]. In *E. coli *the 3'-terminal phosphate cyclase *rtcA *gene forms an operon with the upstream *rtcB *gene. Expression of *rtcAB *is regulated by *rtcR*, a gene positioned upstream of *rtcAB*, but transcribed in the opposite direction, encoding a sigma54-dependent regulator [46]. *rtcBA *and *rtcR *genes are conserved in both G51_ST25 _and G51_acb _islands, separated by *rntZ*. Interestingly, only *rntZ *is present at the corresponding chromosomal position in strains lacking G51.

In type I restriction systems the three subunits S, M and R, which may variably associate to form a modification methylase or a restriction endonuclease, are encoded by *hsd *(host specificity of DNA) genes. Alternative *hsd *genes reside in G13_ST25 _and G13_ST78_. The former are clustered in one operon, whereas *hsdSM *and *hsdR *genes in G13_ST78 _are at distance, as frequently found in other species.

Homologs of a cytosine DNA methyltransferase and a restriction endonuclease, which may constitute a type II restriction modification system, are encoded by genes residing in G38_ST78_.

The G55 islands found in strains 4190, AB0057 and AYE are closely related, and all include a CRISPR (Clustered Regularly Interspaced Short Palindromic Repeats) block, flanked by a *cas *(CRISPR-associated) gene cluster. CRISPRs are repeated DNA sequence blocks found in the genomes of approximately 40% of bacteria, often next to a cluster of *cas *genes. The CRISPR/Cas system provides a form of acquired immunity against exogenous DNA, foreign DNA sequences being first integrated at the CRISPR locus and eventually degraded by Cas proteins [48]. Horizontal transfer of CRISPRs and associated genes among prokaryotes is documented [49].

Gram-negative bacteria contain a variety of genes encoding proteins enriched in dipeptide motifs (valine-glycine repeats) hence called Vgr. Islands encoding Vgr-like proteins are found inserted at eight genome variable loci (loci 2, 7, 15, 17, 19, 25, 27 of Figure 2). Vgr proteins are associated with ligand-binding proteins at the bacterial surface [50], and are involved in biofilm formation and swarming and swimming motility in *Burholderia *[51]. Intriguingly, Vgr proteins, along with Hcp (hemolysin co-regulated) proteins, are components of the type VI (T6SS) secretion apparatus, a transport system extensively conserved among Gram-negative bacteria [52]. Secreted Vgr proteins assemble a cell-puncturing device analogous to phage tail spikes to deliver effector proteins, and are also able to covalently cross-link host cell actin contributing to T6SS pathogenicity [53]. A T6SS gene cluster is conserved in all the analyzed *A. baumannii *strains.

### *A. baumannii *GEIs in other species of the *Acinetobacter *genus

**Figure 4:**
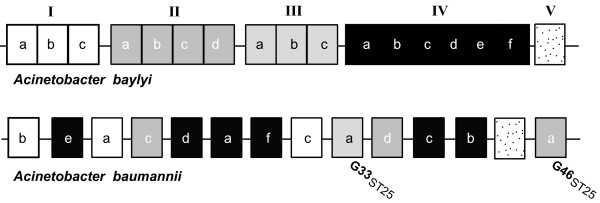
**Scrambling of *A. baylyi *DNA islands in *A. baumannii***. Genes clustered in *A. baylyi *in the so-called archipelago islands [27] are conserved in the *A. baumannii *chromosomes, but are unlinked. The relatedness of two *A. baylyi *islands to *A. baumannii *4190 strain GEIs is shown.

*Acinetobacter baylyi *is a non-pathogenic nutritionally versatile soil bacterium. The chromosome of the *A. baylyi *strain ADP1 carries metabolic genes involved in the utilization of a large variety of compounds. Most of these genes are clustered in five major catabolic islands, grouped in the so called archipelago of catabolic diversity [27]. The organization of the *A. baylyi *and *A. baumannii *chromosomes is different, and most catabolic islands of *A. baylyi *are conserved in all *A. baumannii *strains, although ungrouped, at separate loci (Figure 4). Interestingly, some archipelago genes were found in G33_ST25 _and G46_ST25_, two accessory DNA regions specific of the *A. baumannii *strain 4190. Prompted by this finding, we checked whether twenty GEIs, including G33_ST25 _and G46_ST25_, were present in *A. baylyi *(GenBank: NC_005966), in the complete genome of the diesel-degrading *Acinetobacter *sp. strain DR1 (GenBank: NC_014259) [54] and in the nine draft genomes of the *Acinetobacter *genus deposited at Genbank. GEIs encoding filamentous haemagglutin and vgr-proteins, as those corresponding to cryptic prophages were not searched because of their heterogeneity. The results of the survey are summarized in Table 3. Seven islands (GEIs 14, 20, 21, 23, 29, 44, 51) are conserved in one or more genomes, flanked at one or both sides by the same genes found in *A. baumannii*, but their dimensions vary, as consequence of gain/loss of DNA segments. As expected for mobile DNA, some islands were missing, and only flanking genes could be identified (genomic empty sites). Segments of G13_ST25 _and G43_ST25 _are spread among non-*baumannii Acinetobacter *genomes, thus suggesting that both GEIs might result from multiple recombination events. Recombination likely contributed to the formation of the large DR1 island encompassing genes found in G37_ST25 _and G37_abc_, two non-homologous GEIs encoding enzymes involved in naphthalene degradation and a RTX-type toxin. Curiously, the two *A. baumannii *islands are separated in the DR1 island by 10 kb DNA homologous to *fha*BC genes found in G38_abc_.

**Table 3:** Distribution of genomic regions in non-*baumannii Acinetobacter *species

*A.baumannii *GEIs	ORF contained	*A.baylyi *ADP1	A.ca*lcoaceticus *RUH2202	*A. haemolyticus *ATCC19194	*A. johnsonii *SH046	*A. junii *SH205	*A. lwoffii *SH145	*A. radioresistens *SK82	*Acinetobacter sp*. ATCC27244	*Acinetobacter sp*. DR1	*A. nosocomialis *RUH2624	*A. pittii *SH024
G13 (ST25)	**[A to L]**	-	C	HL	HL	AB	HL	-	EFG...HL	C	C	CDE HL
G14	**[A to H]**	**[A to H]**	**[A to H]**	-	-	-	-	-	**[A to H]**	**[A to H]**	[ ]	**[A to H]**
G18 (ST78)	**[AB]**	-	-	-	-	-	-	-	-	**[AB**	-	**[AB**
G20 (ST78)	**[A to C]**	[ ]	-	BC	-	-	-	-	[ ]	**[A to C]**	[ ]	**[A to C]**
G21 (ST25)	**[A to E]**	-	[ ]	-	-	-	-	-	-	**[A to E]**	[ ]	**[A to E]**
G23 (ST25)	**[A to H]**	-	[ ]	-	-	-	-	A to G	-	**[A to H]**	**[A to H]**	**[A to H]**
G29 (ST25)	**[A to D]**	-	-	-	-	-	-	-	-	**[A to D]**	**[A to D]**	[ ]
G33 (ST25)	**[A to AF]**	**[A to AF]^#^**	-	-	-	-	-	-	-	**[A to AF]^#^**	-	-
G35 (abc)	**[A to N]**	-	-	-	-	-	-	-	-	[novel GEI]	-	C, N
G36	**[A to I]**	-	**[A to I]^#^**	-	-	-	-	-	-	**[A to I]^#^**	[A to F	[C to F
G37 (ST25)	**[A to H]**	-	-	B to E	-	-	-	-	-	**[A to H]^#^**	[ ]	[ ]
G37 (abc)	**[A to G]**	-	-	-	-	-	-	-	-	**[A to G]^#^**	[ ]	**[A to G]^#^**
G43 (ST25)	**[A to V]**	-	-	-	-	-	FG...H to V	FG...H to V	-	-	A to C	-
G44	**[A to D]**	-	[A to D]	-	-	-	-	-	-	[A to D	A to D	**[A to D]**
G46 (ST25)	**[A to E]**	CDE	CDE	-	-	-	-	-	-	CDE	[ ]	[ ]
G47 (abn, aby)	**[A to R]**	BL	-	L	-	BL	-	-	-	**[B to R]**	**[B to R]**	**[B to R]**
G51 (abc)	**[A to G]**	-	**[A to G]**	**[A to G]**	-	**[A to G]**	-	-	B to L	**[A to G]**	C	**[A to G]**
G57 (acb)	**[A to H]**	M to AG	-	-	-	[ ]	-	-	-	[ ]	[ ]	-

ORFs in each island are referrred to by capital letters. Brackets denote ORFs flanking genomic islands. Conserved genomic regions are highlighted in bold. Dots between letters denote that corresponding ORFs are not contiguous. #Genomic regions larger than those identified in *A. baumannii*.

**Figure 5:**
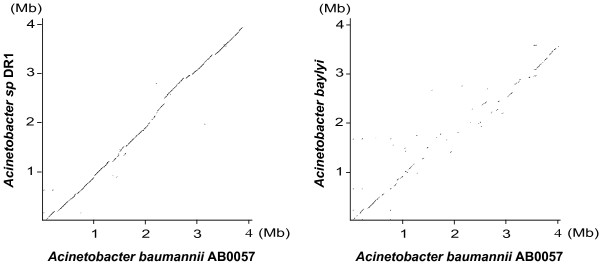
**Dot plot comparisons of *Acinetobacter *genomes**. The degree of relatedness of the *A. baylyi *and *Acinetobacter sp. DR1 *chromosomes to the *A. baumannii *AB0057 chromosome is illustrated by dot plot comparisons.

A high number of GEIs is conserved in the genome of the *Acinetobacter *sp. strain DR1. Interestingly, dot plot analyses showed that gene order is more similar between *A. baumannii *AB0057 strain and *Acinetobacter *sp. strain DR1 than between the same *A. baumannii *strain and *A. baylyi *(Figure 5). According to *rpoB *sequence analysis, DR-1 strain belongs to the *A. calcoaceticus*-*A. baumannii *complex, and is closely related (99.7% identity) to gen. sp. "Between 1 and 3" [3].

### Genomic regions in *A. baumannii *strains of different genotypes

The distribution of 18 genomic islands in the *A. baumannii *population was monitored by PCR analyses. Coding DNA regions of 600-1500 bp, representative of each GEI, were amplified from the DNA of 23 *A. baumannii *strains associated with 21 epidemics that occurred in 14 hospitals of the Mediterranean area from 1999 to 2009, including the sequenced 3909 and 4190 strains used as control. Nearly all the strains were representative of cross-transmission episodes, and were isolated with identical PFGE types from more than two patients of the same or different institutions [9]. Strains belong to eight different STs, and 10/23 strains are ST2. PCR data are summarized in Table 4. Taking into account that negative data may denote partial island deletion or polymorphism in sequences targeted by the primers, the conservation of islands seems to vary significantly among the analyzed strains. G43 and G51 had been found in most strains but not in the two strains assigned to ST78 and some strains assigned to ST2. In contrast, G18 is missing in all except one ST25 strain (4190), and G57 is found only in 3 strains of different STs. G47 seems to be a marker of ST1 strains, being found in all 5 strains assigned to ST1, as well in two strains assigned to ST20, which is a single locus variant of ST1. Similarly, G6 and G11 seem to be markers of ST2 strains, being found in all 10 ST2 strains. Interestingly, the three islands are also present in the single ST3 strain analyzed. G37 is also found in all ST2 strains and also in strains assigned to ST3, ST15 and ST84. G32 is found in all but not ST1 and ST20 strains. All the eleven islands found in the genome of the 4190 strain are conserved in the other two ST25 strains analyzed, with the exception of G8 and G63, both missing in the 3890 strain. Of the eleven islands, two (G23 and G46) are found only in the 3 ST25 strains, six (G3, G8, G63, G43, G21, G51) are also present in strains assigned to other STs. No correlation was found between the pattern of island distribution and PFGE profile among strains.

**Table 4:** Distribution of genomic regions in *A.baumannii *strains of different genotypes

Strain	ST type	PFGE type	G47	G37	G11	G6	G57	G18	G51	G32	G20	G43	G3	G21	G33	G23	G46	G63	G8
																			
AB0057	1	nd	1	0	0	0	0	0	0	0	0	0	0	0	0	0	0	0	0
AYE	1	nd	1	0	0	0	0	0	0	0	0	0	0	0	0	0	0	0	0
700	1	A	1	0	0	0	0	0	1	0	0	1	1	1	0	0	0	1	0
3891	1	B	1	0	0	0	0	0	1	0	0	1	1	1	0	0	0	1	1
3887	1	C	1	0	0	0	0	0	1	0	0	1	1	1	1	0	1	1	0
2979	20	D	1	0	0	0	0	0	1	0	0	1	1	0	0	0	0	1	0
3130	20	E	1	0	0	0	0	0	1	0	0	0	0	0	0	0	0	0	0
ACICU	2	nd	0	1	1	1	0	0	0	1	0	0	0	0	0	0	0	0	0
2105	2	F	0	1	1	1	0	0	1	1	0	1	0	0	0	0	0	1	1
2638	2	F	0	1	1	1	0	0	1	1	0	1	0	0	0	0	0	1	1
3892	2	F	0	1	1	1	0	0	0	1	0	0	0	0	0	0	0	0	0
3990	2	F	0	1	1	1	0	0	0	1	0	0	0	0	0	0	0	0	0
2735	2	F1	0	1	1	1	0	0	0	1	0	0	0	0	0	0	0	0	0
3858	2	F2	0	1	1	1	0	0	0	1	0	0	0	0	0	0	0	0	0
3889	2	G	0	1	1	1	0	0	0	1	0	0	0	0	1	0	0	0	0
4026	2	H	0	1	1	1	1	0	0	1	0	1	0	1	0	0	0	0	1
4030	2	I	0	1	1	1	0	0	0	1	1	0	0	0	0	0	0	0	0
4009	2	J	0	1	1	1	0	0	0	1	0	0	0	0	0	0	0	0	0
4025	3	K	1	1	1	1	0	0	1	1	0	1	0	0	0	0	0	1	1
3890	25	L	0	0	0	0	0	0	1	1	1	1	1	1	1	1	1	0	0
3865	25	M	0	0	0	0	1	0	1	1	1	1	1	1	1	1	1	1	1
4190	25	N	0	0	0	0	0	1	1	1	1	1	1	1	1	1	1	1	1
ATCC17978	77	nd	0	0	0	1	1	1	1	1	0	0	0	0	0	0	0	0	0
3909	78	O	0	0	0	0	0	0	0	1	1	0	0	0	0	0	0	0	0
3911	78	O1	0	0	0	0	0	0	0	1	1	0	0	0	0	0	0	0	0
3868	15	P	0	1	0	0	1	0	1	1	1	1	1	1	0	0	0	0	1
3871	84	P1	0	1	0	0	0	0	1	1	0	1	1	1	0	0	0	0	1

Positive or negative PCR amplification are indicated by 1 or 0, respectively; nd, not done.

## Discussion

Data reported are in line with the results of previous analyses [16], indicating that the genomes of *A. baumannii *strains isolated from geographically different regions are closely related and share the same overall organization. Shared synteny made possible to align the seven *A. baumannii *genomes throughout, and obtain a robust chromosomal scaffold by which easily distinguish core and accessory genome components in each strain.

The ST2 strains ACICU and 3990 exhibit 99.9% sequence identity, and share the same core and variable genome components. Mapped differences are restricted to size changes of ˜40 intergenic regions, which vary in the two strains because they contain a different number of short sequence repeats. A major difference can be ascribed to a > 36 kb CP3-like element, found in the 3990 strain only, the chromosomal location of which has not yet been determined. Two CP3-like prophages specific of strains 3909 and 4190 have not yet been mapped as well. The ACICU and 3990 strains are however phenotypically distinguishable, since the his-leu replacement at residue 535 of the *beta *subunit of the RNA polymerase made the 3990 strain not susceptible to rifampicin (MIC > 500 mg/L). Sequence comparisons revealed that 3068 coding regions are conserved, at the same chromosomal position, in all *A. baumannii *genomes. Accessory coding regions, including both GEI- and mhr-encoded ORFs, varies from 433 (3909 strain) to 707 (AB0057 strain). In estimating the number of conserved coding regions, it was taken into account that many correspond to a single ORF in one genome, but to two or even three adjacent ORFs in others, and vice versa. Likely most "double ORFs" are artifactual, since mutations are known to be introduced by PCR amplification of DNA samples prior to sequencing. Accessory DNA regions correspond to 12% of the 3909 genome, 19% of the AB0057 genome, and to 14-16% of all other genomes analysed. Although closure of draft genomes and addition of whole genome sequences of other strains may lead to the definition of a few additional GEIs, data clearly indicate that *A. baumannii *strains exhibit less variation than *E. coli *strains, which may share only 60-70% of their coding capacity [55].

Many *A. baumannii *GEIs have a role in drug resistance, biosynthesis of surface components, iron metabolism, and this may confer advantage in the course of an infection, since successful pathogens encode multiple adhesins, are equipped to sequester iron from the environment and can escape therapy. Less clear is the advantage conferred to *A. baumannii *by other islands. The functional role of the RNA 3'-terminal phosphate cyclase, an enzyme conserved among Bacteria, Archaea and Eucarya, encoded by G51_ST25 _and G51_acb_, is debated. The same holds for vgr-like proteins, encoded by several GEIs, though it is worth noting that six of the ten genomic islands identified in the pathogenic *P. aeruginosa *PA01 strain [56] encode vgr-like proteins. Some GEIs carry genes involved in lipid metabolism. G47_abn _and G47_aby _carry genes controlling the formation of CFA and UFA phospholipids. Cyclopropanation plays a role in the pathogenesis of *Mycobacterium tuberculosis*, a specific CFA synthase being required to modify the alpha mycolates on the cell envelope, and pathogenic *E. coli *strains have higher CFA contents and are more resistant to acid shock than non-pathogenic strains [57]. G6_abc _and G6_acb _carry homologues of genes involved in fatty acid metabolism (Fab genes) conserved at multiple loci in all *A. baumannii *strains. Additional Fab genes may confer metabolic advantage, and is worth noting that Fab and other GEI-6 genes reside in OI-47, a genomic island conserved in all O157:H7 *E. coli *strains [58]. Finally, Many GEIs, most of which unique to the 4190 strain, carry genes and/or operons controlling specific metabolic pathways, such as naphthalene and phenyl-propionic acid degradation.

Several GEIs correspond to cryptic prophages. Of these, a few may have conserved the ability to replicate as phages upon appropriate stimuli, and CP3, CP9 and CP14 encode lysozyme. However, none exhibited homology to bacteriophages so far identified in *A. baumannii *[59,60]. Few CPs are decorated by morons, accessory genes unnecessary for the virus, which may be helpful for the host bacteria when the prophage is integrated in its genome. Advantage conferred by morons is debated. PapS reductase functions in the assimilatory sulphate reduction pathway, and could serve as a fitness factor under conditions of iron limitation [61], *umuDC *gene could convey a mutator phenotype on the host [62]. As previously noted [16], the high variability exhibited by prophage sequences suggests recent insertion/and or rapid loss, and a large pool of phage genomes.

Genotypic characterization of *A. baumannii *isolates during outbreaks occurred in different geographical locations showed the prevalence of clusters of highly similar strains [4,10]. Data presented suggest that strains assigned to distinct genotypes according to MLST analysis may harbour specific GEIs. However, variability exists in the distribution of other genomic regions between *A. baumannii *strains assigned to the same genotypes, thus suggesting that horizontal gene transfer and recombination may occur between strains of different genotypes.

The identification of sequences homologous to several GEIs suggests that the genomes of non-*baumannii Acinetobacter *spp. may function as reservoirs of accessory *A. baumannii *DNA. Bacteria of the genus *Acinetobacter*, including *A. baumannii *isolates, are naturally competent [63] and have likely exchanged DNA in evolution. A few GEIs are perfectly conserved in different *Acinetobacter *species, but many vary in size and content, and have been plausibly remodelled both by recombination and insertional events. Comparative analyses also demonstrated a marked difference in the genome organization of the non-*baumannii Acinetobacte*r sp. *baylyi *and DR1 relatively to *A. baumannii*.

Differences among *A. baumannii *genomes are also correlated to large strain-specific deletions, which are interestingly associated to selective loss of function. The 3909 strain lacks *mucK *and *tcu *genes which enable the growth on cis, cis-muconate and tricarballylate as sole carbon sources [64,65]. The 4190 strain lacks *tau *genes, needed to utilize taurine as a sulphur source in sulphate starvation conditions [36], the AYE and ACICU strains lack genes enabling growth on d-glucarate as sole carbon source [66], the ATCC17978 lacks genes involved in the metabolism of anthranilate, molybdate transport, biosynthesis of the pyrroloquinoline quinone cofactor, chaperone-usher pathway, growth on dicarboxylic acids as the only carbon source [67]. All these large deleted regions can alternatively be viewed as GEIs conserved in the population but missing in one or a few isolates. Sequencing of additional *A. baumannii *isolates will set the issue.

## Conclusions

The definition of the genome components of *A. baumannii *provides a scaffold to rapidly evaluate the genomic organization of novel clinical *A. baumannii *isolates. Distinguishing conserved from accessory components in *A. baumannii *chromosomes is a functional framework useful for further investigations on the biology and the genetic organization of this species. Changes in island profiling will be useful in genomic epidemiology of *A. baumannii *population. Data provided in this work will facilitate comparisons of *A. baumannii *isolates, and help to define the features of *A. baumannii *as species as to pin down its pathogenic traits.

## Methods

### *A. baumannii *strains

Comparative genome analysis were performed on whole genome sequences of *A. baumannii *strains AB0057 [GenBank:NC_011586] [16] , ACICU [GenBank:NC_010611] [12], ATCC17978 [GenBank:NC_009085] [17] and AYE [GenBank:NC_010410] [18] and draft genome sequences of *A. baumannii *strains ST2 3990 [GenBank:AEOY00000000], ST25 4190 [GenBank:AEPA00000000] and ST78 3909 [GenBank:AEOZ00000000] strains [11]. The GenBank:CP000521 file, which contains 436 hypothetical proteins putatively encoded by ATCC17978 early annotated as AS1, but not included in the GenBank:NC_009085 file, was also used for comparisons. The genome sequences of non-*baumannii Acinetobacter *species *A. baylyi *ADP1 [GenBank:NC_011586], *Acinetobacter sp*. DR1 [GenBank:NC_014259], *A. calcoaceticus *RUH2202 [GenBank:ACPK00000000], *A. haemolyticus *ATCC19194 [GenBank:ADMT00000000], *A. johnsonii *SH046 [GenBank:ACPL00000000], *A. junii *SH205 [GenBank: ACPM00000000], *A. lwoffii *SH145 [GenBank:ACPN00000000], *A. radioresistens *SK82 [GenBank:ACVR00000000], *Acinetobacter sp*. ATCC27244 [GenBank:ABYN00000000], *A. nosocomialis *RUH2624 [GenBank:ACQF00000000] and *A. pittii *SH024 [GenBank:ADCH00000000] were also used for comparison. The *A. baumannii *strains used in PCR analyses of GEIs have been previously described [10].

### Genome analyses

Gene products putatively encoded by the ST25 4190, ST78 3909 and ST2 3990 strains were identified using xBASE2, comparing the draft genome sequences to the genome of the *A. baumannii *strain AB0057 used as reference template [11]. The corresponding amino acid sequences are listed in Additional file 7. Predicted ORFs were subsequently compared to the gene products of the wholly sequenced *A. baumannii *AB0057, ACICU, ATCC and ABAYE strains using MAUVE [15]. Homologies under looked by MAUVE were detected by BLAST and tBLASTn analyses. Gene products encoded by aligned coding regions exhibited at least 50% identity. *rpoB *gene sequence analysis for genomic species identification was performed as previously described [3].

### PCR analyses

The conservation of specific GEIs in a set of *A. baumannii *strains was assessed by PCR amplification. PCR reactions were carried out by incubating 20 ng of genomic DNA with 160 ng of each primer in the presence of dXTPs (200 nanomoles), 1.5 mM magnesium chloride and the Taq DNA polymerase Recombinant (Invitrogen). The sequences of the oligomers used as primers, the experimental conditions, the length of the amplimers, the coding regions amplified are all listed in Additional file 8. PCR products were electrophoresed on 1.5-2% agarose gels in 0.5×TBE buffer (45 mM Tris pH 8, 45 mM Borate, 0.5 mM EDTA) at 120 V (constant voltage). The 100 bp ladder (Promega) was used as molecular weight marker.

The co-linearity of contigs and the DNA content of the corresponding chromosomal regions were assessed by sequencing PCR products bridging contig ends.

## Competing interests

The authors declare that they have no competing interests.

## Authors' contributions

Conceived and designed the experiments: PPDN, FR, MG, MT, and RZ. Performed the experiments and analyzed the data: FR, PPDN, and MG. Wrote the paper: PPDN and RZ. All authors read and approved the final manuscript.

## Supplementary Material

Additional file 1 **Structures of plasmids identified in ST2 3990, ST25 4190 and ST78 3909 strains**. the figure shows the circular maps of plasmids p1ABST2, p2ABST2, p1ABST25, p2ABST25 and p1ABST78 with relevant features. ORFs and direction of the transcription are represented by arrow-shaped boxes. Plasmid sizes and names of various features are reported. Click here for file

Additional file 2 **Coding capacity of plasmids carried by strains 3909 3990 and 4190**. the table lists ORFs of plasmids p1ABST2, p2ABST2, p1ABST25, p2ABST25 and p1ABST78. Position, number of amino acids and putative function are reported for each ORF. Click here for file

Additional file 3 **Target site duplications**. sequences duplicated at the ends of GEIs upon genome integration are listed in the table. Base changes in left and right TSDs are marked according to IUB codes. Residues missing in one TSD are in parenthesis. Known target genes are indicated. Click here for file

Additional file 4 **GEIs organization and ORFs content**. the 63 sheets of the EXCEL file correspond to the 63 genomic loci carrying GEIs shown in Figure 2. The ORF number, the amino acid length and the hypothesized function are given in each sheet. For draft genomes, the corresponding contigs are indicated. Identical or closely related ORFs present in different GEIs are positioned in the same row and labelled by the same colour to facilitate view. ORFs denoted as tb were identified by tBLASTn analyses. Grey and orange bars denote closely located ORFs putatively co-expressed. Homologous coding regions are boxed when a single ORF in one strain corresponds to two or more contiguous ORFs in others. Click here for file

Additional file 5 **Micro-heterogeneity regions**. coding regions present/absent in the compared *A. baumannnii *genomes, denoted in the text as *mhrs *(micro-heterogeneity regions), and their hypothetical function, are listed in the table. Alternative regions present at the same locus are marked by different colour characters. *mhrs *containing two or more ORFs are boxed. Click here for file

Additional file 6 **Cryptic prophages**. structures of cryptic prophages identified in *A. baumannii *genomes. Prophage types are boxed to highlight their relatedness as resulting from MAUVE alignment. Different CP1 and CP2 are shown to illustrate the degree of genetic variation of *A. baumannii *prophage families. Click here for file

Additional file 7 **Gene products putatively encoded by strains 4190, 3909 and 3990**. ORFs of strains 4190, 3909 and 3990 and the corresponding contig number are shown. Click here for file

Additional file 8 **Genomic regions, amplified genes, primers, amplicon sizes and cycling conditions used in PCR surveys**. (none, title sufficiently describes data). Click here for file
