# Supplementary figures and images for: Genome organization of epidemic Acinetobacter baumannii strains
Source: BMC Microbiol. 2011 Oct 10;11:224. doi: 10.1186/1471-2180-11-224 (PMC3224125; doi:10.1186/1471-2180-11-224)

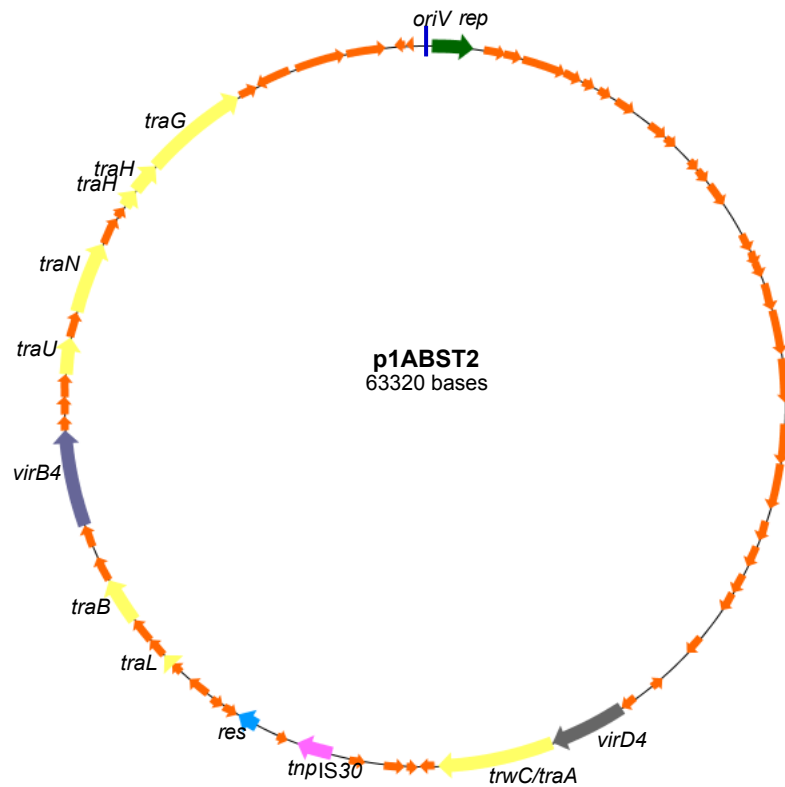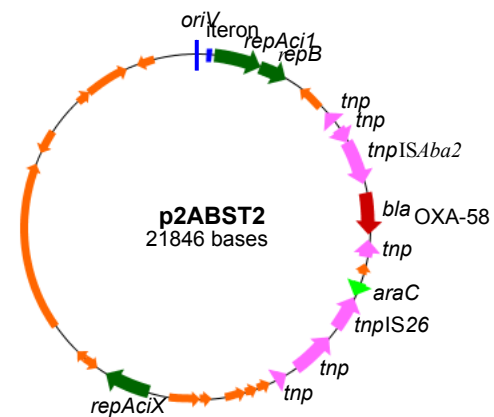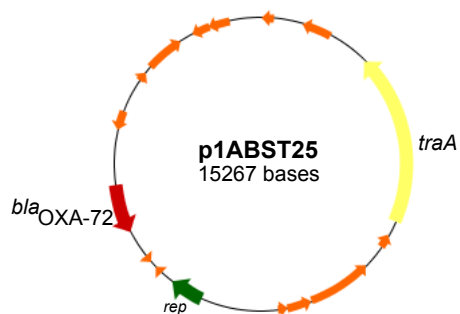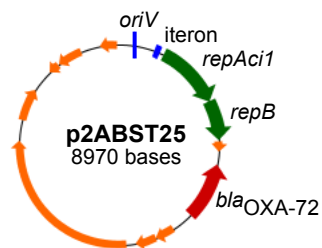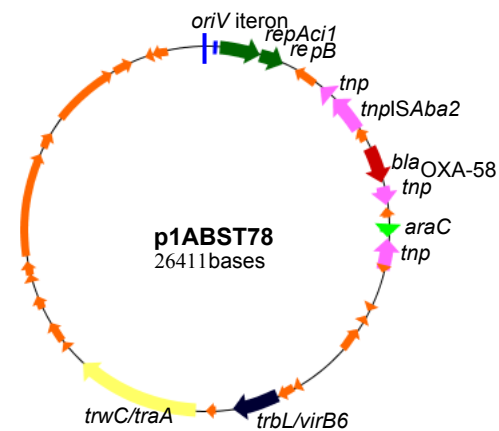

Supplement: Additional file 1 — Structures of plasmids identified in ST2 3990, ST25 4190 and ST78 3909 strains. the figure shows the circular maps of plasmids p1ABST2, p2ABST2, p1ABST25, p2ABST25 and p1ABST78 with relevant features. ORFs and direction of the transcription are represented by arrow-shaped boxes. Plasmid sizes and names of various features are reported. [file 1471-2180-11-224-S1.PDF]

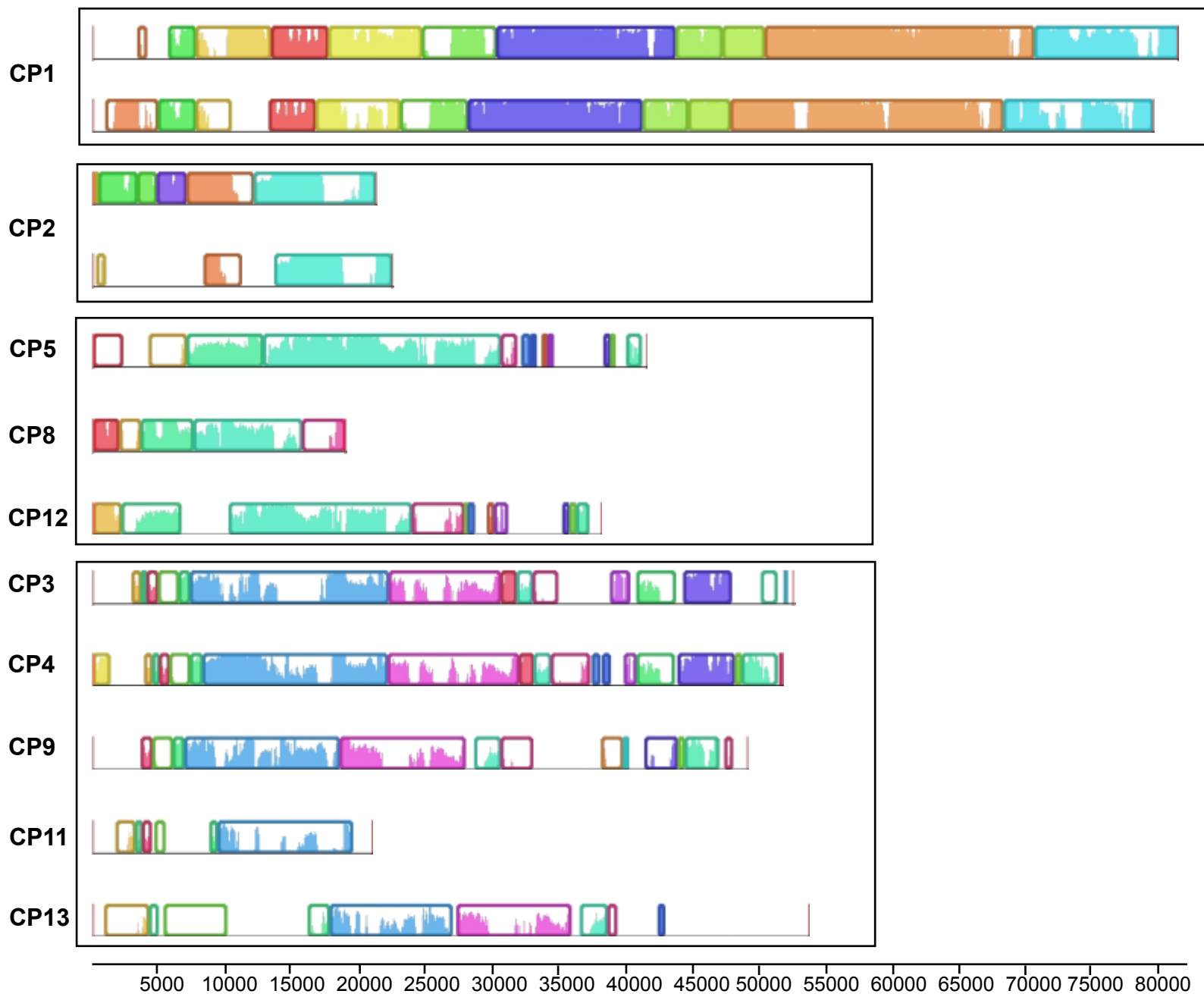

Supplement: Additional file 6 — Cryptic prophages. structures of cryptic prophages identified in A. baumannii genomes. Prophage types are boxed to highlight their relatedness as resulting from MAUVE alignment. Different CP1 and CP2 are shown to illustrate the degree of genetic variation of A. baumannii prophage families. [file 1471-2180-11-224-S6.PDF]
