# Supplementary material for: Genome organization of epidemic Acinetobacter baumannii strains
Source: BMC Microbiol. 2011 Oct 10;11:224. doi: 10.1186/1471-2180-11-224 (PMC3224125; doi:10.1186/1471-2180-11-224)
Supplement: Additional file 7 — Gene products putatively encoded by strains 4190, 3909 and 3990. ORFs of strains 4190, 3909 and 3990 and the corresponding contig number are shown. [file 1471-2180-11-224-S7.DOC]

**ST25 4190 ORFs**

ST25 4190 cg1_0002

MRGCSKNHGQPPIIEGIFWQPDNDTTPPKGNWHYLGINTFVPQWSVVESKSWWKNSNLPQWEKAVDLQKIKQQPWAKNLILGLAGEYNEHEARANVVALGEKSAQIIQEQNDASLKGYYFPVEADPTWLRVSTLGHVLEKLPSPIWVSVYSGESEPENYDLWVKSWLPQQAGVFFQDGVGVGVRTPEQARRILDQLEQTLGKDKTVIVLEAFRTKKNGQFRAAYPWEIISQIKAYEGKKIYIFDGPHYMGRWSVYIVGLWYRLVYGSTPATINEPKNSK

ST25 4190 cg1_0003

MKKIFISIVFGTRPELIKLAPVILLAKQDARFQVEVIFTGQHDELVRDAIDFFGVEIDHRLKIMNAGQSLNQLLIHGLTQLENIYTDGQKRDAIVIQGDTTTVLAAGLVAFSMKIPVAHVEAGLRSYDLDHPFPEEGNRQLVSRITKWHFAPTEQSKRNLLNEQIPPSLITVTGNTVVDAVYLGRKLIAEKTGLKNQLEPYGIELKQNDKVVLITAHRRENFGEGIQNICNAVEYLAKHHPDLHFIWPVHLNPAVHNVVHDKFKNHAQIHLVKPLDYPSLLAIIDRSTFILTDSGGLQEESPSFNKPVLILRDTTERPEVVEVGAGVLVGTNQQKIIEEAEKLLTDSQHYQKMAHVENPFGDGRAAQRILDEIARTYN

ST25 4190 cg1_0004

MGAAISTLGMTGISSVYAQEQVDPAYKPGNPIYDKWDRFYKIEQSQPQEAEKILVELSKLTPTDIKVWKSLTYLQIRLEKREEALQSLRQARNLAPQDDTLKLQEAYLLNQQKKDREALVLFKELSSSSDPEIAAKATQAVKNLSGGEVKPYFKDIYFAPSYESRYDDVIFPLKMRYGKNIDNGRAQVYAFLNLNRDTQSQGGVRPEIIDENAATLGLGANYQPWTSIPVRAYVEVGGSYDLIDRNRKRFRESVVGGVTGYQEWYSQSNCDHSLCLDNYFTDLYGNVATYSREDYNVIGDLRLRTGLNLYKGESGSVQAYVKLHGLADSEDEYYNNLFEYGPGISWQPFNYQPIKLRVERLYGNYFKDVPVNTKDHYNNTRVELVFYKDF

ST25 4190 cg1_0005

MAFPHHTEHHAIQRSGWLRASVLGANDGIISVTSLIMGMAASGASSHTLFITCVAGLISGATSMAAGEYISVKSQEDIEKSDLAIEAKELNKYPQKELDELTQIYISRGLSKELAKEVAIQLTTHDALGAHARDEIGIHENTAANPIQAALSSAASFSFGAFFPMLAILFSPEHLIMPSVLITGIAALAILGALSSYFAGTSKIKGSLRITLWGILAMAFSSWIGSLFNVTPL

ST25 4190 cg1_0006

MSLLQQLAIKHPIFLAPMAGVSTPELAAEVSNQGGLGSLGLGANTPQSAREQILKTQALTENPFQVNFFCHRSTELNVEKAKQWLEYLRPHFEKFGAQPPQELHCIYSSFLDNDDFLNIVLETKPKAVSFHFGIPHPHQIKALKEAGILTMVSATNLIEAQAIEAAGIDIIIAQGIEAGGHRGIFNQTFDGAIKTSDLVQLIVQHCTLPVVAAGGIMTGLQAKHMLGLGAAAVQLGTAFVQCQTSNASAEYRKALFSKPVTQISASLSGRPARGILNHWHTKIDSPTRPVQPEYPYTYDLAKQLNALASKHHDYGFGAFWAGSNVAQIRELEAPDLVNQLVVEMLDNE

ST25 4190 cg1_0007

MYIGPYQLSNNLIVAPMAGVTDRPFRTLCKYFGAGHAVSEMMTADKTLRMTKKSLYRANFDGELAPISAQIAGSDPEQLAEAARYQVANGAQIVDINMGCPAKKVCNKLAGSALLQDEDLVARILDAVVAAVDVPVTLKTRLGFLNGQENILRVAKRAEEAGIAALALHGRTREDMYLNTARYELIKHVKELIHIPVIANGDIDSPEKAKYVLDYTGADAIMIGRAAQGRPWIFREIAHYLKTGEHLAAPNIEEVKEVLLGHLSELYQFYGEYSGCRIARKHIAWYTKGLRSSNEFRQNMYKVETTAEQALVVESYFNQLLAEGNLMSDVQVEQVNLLETH

ST25 4190 cg1_0008

MVAVCMSLAACGDKAWWSNNDEPELESQQIKRLIPSRVHDRESWAKDIDDIMKDLDIPKTKQNVCSIVAVVDQESNFVANPQVPGLGQKAVEEVSTRLNEKFEDKLGKTIGGTIAGYFEEVLRTQPSPDNNYMSQMRKVKTEKDLDLLYREIFDFMAKHYHVSALTGAAKLVGQDIGEKMNPITTLGSMQVHINYAKANKRSSMNTAALRDDLYTEYGGLYYGIHRLMVYPADYDKAIYRFADYNSGMYSSRNAAFQKMLKELTDKDISLDGDLLLYTKDGDPRATQSESEKELITVFASNNVLVTPRQIRDDLKLEKEKKFESTQTYIALTKLYKSKTGKEPLYAIMPQVVISGPKLSRDYNTNWYATRVNGRYETCMQRAKRIRL

ST25 4190 cg1_0009

MYATSETNKTIKNLALFDFDGTLCSKDSFTGFIFYALSKRHIVKQGLKILPWIQAYYLNFYPAHAMRAKLFRSMFRDTPAIELQRLGEEYAQELVSALSPEIFAQLQQHQLLGDQVVLVSASIDIYLAPLCKLLGIELICTETQVKNGMMTGYYSTPDCSSEQKKLRISEQYPLKHYQRIYAYGNSSEDLDMLSLATHPFMVGEDRILPSLTPQKKLA

ST25 4190 cg1_0010

MIEVVDGQRKLSECKRIVVKIGSSLLTANGQGLDLDAISHWAKQIADLHNAGHEIILVSSGAVAEGMVRMKLASRPTDLPSLQACAAIGQMGLIHTWSSVLENHSIRTAQVLLTHDDLADRRRYLNSCDALQNLIDWRVIPVINENDTVSTDEIRFGDNDTLAAMVAGQVHADLLIILTDQQGMFDSDPRHNPDAKLLSTVRAMDDVLFEMAGGGGVLGRGGMVTKVRAARLAAKSGCPTLIASGESDNVLSRVMAGEMLGTLFTTDKDRMTAHQQWLAAHLQTAGRLVIDDGAVEAIKLKHRSLLPVGVKTVEGHFDRGDVVECVDKQGKRVAVGRVNFSSRSAEIIKGLSSDKVYQVLGEARSLEMIHRDHMAIY

ST25 4190 cg1_0011

MRFVDEAVITVEAGDGGNGVASFRREKFVPFGGPDGGDGGRGGSIYIQADDDTSTLVDYRYTRKFRAERGKNGAGANCTGRGGEDVVLKVPVGTTIVDTDSGDIIGDLVEDGQRVMVASGGEGGLGNTHFKSSTNRAPRKCTTGTKGEFREIRLELKVLADVGLLGMPNAGKSTFIRAVSAAKPKVADYPFTTMVPNLGVVDADRHRSFVMADIPGLIEGAAEGAGLGIRFLKHLARTRILLHIIDVQPIDGSDPAHNAKAIMNELAKFSPTLAKLPIVLVLNKLDQIAEESREEWCQHILDELQWTGPVFKTSGLLEEGTKEVVYYLMDQIEQQREREVEDPEYAAEVRAFREQLEAETREQTIAAKEAYRAMRKAQRLESMMDDDDDFDDDEDDGDVESIYVRD

ST25 4190 cg1_0012

MILTAILFWFYLNIQASVVVSAHESDIRLPESLETKIHVGNNLQVQSIGKLDTSIDIDRQISVPLKGRYLADLEFEVETPITVSVDYATTLKVDQVMPLETTTDLIYKNKLLPRFPLKLNIPIKLDIPFQLKRSYTIPVKIAFNGPVYFEFDESINLPIKHQFNPSLNVNDAMEMGNISSFNAIMYNSVQHTKANLDMKMDLPLRNVHP

ST25 4190 cg1_0013

VFKHFNIYIPLKNQAVAIDLQEPLQVQVKVKDALNVDVKGRVNANIPINEKLNIPLTQTLTPRVYFDNMVPISTVIPVKETLKVDQSLPIDTKVQVRVLGKDITLPLKGNIPLQLDVPIDIQVPFDQKVHLKFDAPVKTVLKENLHIPLNAQLKTNIPIQGSLNVPVKTALNASVDVKNTLPVKIEHGELKIPLSSMSLNRVKSGEDNSSPVQNQTK

ST25 4190 cg1_0014

VSKDTIIALHAEHQGRWKNREEIAERMIALIGQLYREKNIVVSVYGRSLINRSVIQILKTHRRTRVVDVELSVVNTFPILEALAKVENIGSAEVDIGKLAVEYKEKGGDVDAFVAQAVESIKGSATSEQPKDVVLYGFGRIGRILARLIISQSGLGRGLSLKAIVVRKSSDGDLAKRASLLRRDSIHGTFAGTISVDEENEAIIANGNFIKVIYASSPSEVDYTQYGIENALLIDNTGKWRDAEGLSQHLKCPGVARVVLTAPSKGEMKNVVFGVNNSDILDEDKIISAASCTTNAITPILKVLDDKYKVLNGHVETVHSFTNDQNLIDNYHKADRRGRAATLNMVITETGAAKAVAKALPALKGKLTGNSVRVPTPNVSLAILNLTLDKEVDREEVNEYIRQISINTNLQGQIGYTNSTEVVSSDFIGSRTAGVYDAQATITSGNRLTAYVWYDNEVGYSCQVLRIAEQMCGVSYKKIPAETNA

ST25 4190 cg1_0015

MLKGVILSVMASLTFGVLYFYTQLLGQLDSEQTFGWRIIATLPFLTLFMWWSGDLSHIKNIYQRILAKPSLLLLLITTSVLTSVQLWLFLWGPMHGRGLQVSLGYFLLPLVLVLAGSVLYGEKISKFQWVAIVLAIIGVGHEIFRLGSIAWETALVAIGYSAYFLLRKKIKTDNLGGFWWDLLIIMPVAIYLTHTGLLPYSKFLDQPTLGLVIAGLGVLSAIGLGCYILASRYLPLVVFGLLGYLEPVLLALASLVLGESIGKEEWFTYLPIWCAVFVLVLEGAFHLYQQQQKAKNLQLNIEKYQKRLKK

ST25 4190 cg2_0017

MKKLILAAACAAASGTIFANSSDTTEQRINLLETELQKLKAELAAQKQTQNNLQVKQVKIEENIEKTKATVTEKPVAPSWVTWTDNVKVYGIARIDAAVDFKSSPDSGGRTTSSLYRTPFESEKRSNHARSDAMINASRLGVYFNSPNKAVTGNIEADFFDSSNMGTGDGKFRIRHAFFTYKDWTFGQTWSLMSNMETRTEAVDYTQFVGTSYTRTPQIRYDWKIDANNDLKVALEYTGSRVSAFPTLTGRYTFKQGPLTALAQGFINEKSTDVATATVKKVSWGAGGGLKYQLTPQQSVQANYQYIVGDQKFMPYTTQSGLANSTNLNAAGDFSLSEDKTDLVMNKLNSINIGYSYKFNEQWRANLSASMFDYDDDTAYAKLNPDANKRLTDYAANVFYSPIEQMDIGMEYHQGKREVFDGRTADVSRVNFVSMYKF

ST25 4190 cg2_0018

MSVQKRSREDIAKLIANDIPEGSYVNLGIGLPTNVAKFLPKDKEIFLHSENGVLAFGPPPQPGEEDQDLVNAGKELVTLLDGGCFMHHGDSFDIMRGGHLDICVIGAFQVAVNGDLANWHTGKADDVPAVGGAMDLAVGAKTIYVYMEHVTKKGEPKIVKELSYPITGEQCVNRIYTDLCIIELKDQKAYVKEMVDGLTFDELQAVTDCELIDARVA

ST25 4190 cg2_0019

MINKITSDIEPILKAIPDGATIMTGGFGVTGQPAELIEALIDLGKKELTIVNNNAASGDRGLTNLIIAGCVKKMICSFPKSAGSTVFQDLYRAGKIELELVPQGNLACRIQAAGAGLGAIFTPTGYGTKVAEGKETRTINGKNYVLEYPLEADFALIYADKADRWGNLTYRKAARNFGPIMAKAAKTTIVQVNDTVELGTLDPECIITPGIFVQHVVKVGDI

ST25 4190 cg2_0020

MDMTTKTVTENMSSTEAIQTPKKIWITAFIFAFFTLLCDGADLGFLALSLTSLKAEFHLTGVQAGTLGSLTLFGSAVGGLIGGWACDRFGRVRIIVFFIAFSSILTCALGFTHSYMQFAVLRTIGAMGLGALYIACNILMSEMVPTKHRSTVLATLMTGYTLGSLLATLLAGHIIPEHGWRFLYWIAITPVVLSVLMHFCVPEPESWKKARQLKALEVAQSNQPKKRQNPYLEILRDKKHGTMFILWIISTGALQFGYYGVSNWLPAYLESDLGIKFKEMAMYMVGTFLIMMFAKVIAGIVADKLGRRAVFAFGTIGTALFIPVIVYLNTPTNILWMMLFFGFLYGIPYAINATYMTESFPTSIRGSAVGGAYNIGKVLSIFSPLTIGYLSQNGSIGLGLLVMAAAYFICGVIPLLFIKDRLFNPQKAE

ST25 4190 cg2_0021

MIRDEGMLEQLLSTIRDFVKNELIPRENEVVEKDKIPDDIVQQMRELGLFGLTIPEEYGGLGITMEEEVRVAFELGQTSPAFRSLIGTNNGIGSSAILIDGTEEQKQKYLPRYASGEIIGSFCLTEPESGSDAASLKTSAVKDGDFYVLNGTKRFITNAPHAATFTVMARTNPEIKGSGGISAFLVEANTPGITLGKIDQKMGQKGSHTCDVIFENCRVPASALIGGVEGVGFKTAMKVLDKGRLHIGAYSVGVAERMLNDALHYAIERKQFGQPIANFQLIQAMLADSKAEIYAAKCMVLDAARRRDNGENISTEASCAKMFATEMCGRVADRCVQIHGGAGYISEYAIERFYRDVRLFRLYEGTTQVQQLIIAKNMIREATA

ST25 4190 cg2_0022

VSTGNNFVEVDFSIQKIAVVKINRPEAKNALNGEVRKQLAQVFTELSFNDEINAVVLTGGEEVFAAGADLKEMATATSTEMLLRHTERYWNAIAQCPKPMIAAVNGYALGGGCELAMHADIIIAGKGATFGQPEIKVGLMPGAGGTQRLFRAVGKFHAMRMIMTGAMVKAEEAYLIGLVSQVTEDDQTIPTAIQMAQSLAKMPPIALQQIKEVALMSEDVPLNAGLTLERKAFQLLFSTEDKNEGVQAFIEKRKPSYQGK

ST25 4190 cg2_0023

MKMVRKVLISAGGSGIGRCIAEVFLNNQDEVFVCDINAKSLEQFQQDYPKLHIYTCDLADHEQIKLMFNEAVQKLGGIDILVNNTGISGPTVAADELSFDDWNTVINLNLNSTFLITQLAIPYLKQAQAGVIINMSSIAGRLGYPYRLAYSTSKWGLIGFTKTLSMELGADNIRVNAILPGAVDGDRVQRVLQARADVAHTSLEEVTQNALKNQSLKYFVNPKHIADLCLFLASDSGRSISGQILPIDGDKQCLS

ST25 4190 cg2_0024

MIDMTYDIKSMAVIGVGVMGSGIAQIAAQSGHTTYLYDAKAGAAEQAKEKLAATFQKLVDKNKITSEQAAAANNHLIVAHQIEDLKNCDLIVEAIVERLDIKQSLMQQLEDIVSDNTILASNTSSLSITAIAANCKKPERVVGYHFFNPVPLMKVVEVIRGLKTDPLIIDALNDLSRAFGHRPVVAKDTPGFIINHAGRAYGTEALKILNENVCDISEIDRILRDGVGFRMGPFELLDLTGLDVSHPVMESIYHQYYEEARYKPNPLTKQMLDAKQLGRKVNQGFYNYETGSKTGEQPAKFVERLAQYPKVWIAADFLDDKKQLEDYLTQHNIALDINPEPQTDSLCLVACYGEDTTQAATRLGINPEQAVAIDMLYGIAKHRTLMPSLITKPEYRQAAHSIFNLDGNMVSMINESIGFVAQRVLAMVINLGCDIAQQNIATVDDINAAVRLGLGYPFGPIEWGDQVGSEKILLILNRITALTHDPRYRPSPWLQRRVALHLPLTFTHES

ST25 4190 cg2_0025

MLNAYIYDGLRSPFGRHAGELASIRPDDLAATVIQKLLEKTGVPGADIEDVILGDTNQAGEDSRNVARNALLLAGLPVTVPGQTVNRLCASGLGAVIDSARAITCGEGELYIAGGVESMSRAPFVMGKAESAYSRDAKIYDTTIGSRFPNKKIIAQYGGHSMPETGDNVAADFGISREQADLFAAQSQAKYQKAKEEGFFADEITPIEVFQSKKLPPKLVSEDEHPRPSSTVEALTKLKPLFEGGVVTAGNASGINDGAAALLIGSEAAGQKYGLKPMAKILSAAAAGIEPRIMGAGPIEAIKKAVARAGLTLDDMDIIEINEAFASQVLSCLKGLNVDFNDPRVNPNGGAIAVGHPLGASGARLALTVARELIRRKKKYAVVSLCIGVGQGLAMVIENVS

ST25 4190 cg2_0026

MSQNTETEKPINFDEIRLDPISHIHRENNPQFIASLARGLELLRCFSANNQVLGNQELAKMTGLPKPTIARITNTLVSLGYLKQLPNSTKYTLDIGVLSLGYAALSNISIRNIAHQYMEEMSQYVQAPVAMATRDRLNMVYIDVVQIETALNMRRPIGSTLPLHSSSMGRACLAATPERERNFLLDALKQKNPENWPAIKRSLERAFRDYQDYGYCLSLGEWHKEVNSVAVPLVSSKHGLYVFNCGAPSFHLNPEKLEGEIGPRLIHMVHNIQDALNETH

ST25 4190 cg2_0027

MTIGMMKNALYTPTIILMAIAAGICTGGNYFSQPLIHSISVSLNLTETTTAWVPTLAQITYACGLLFLMPLGDIIEKRKLLFIFMLLASSGLIISGFSHNIYLLLLGTIITGLFSSAAQLLLPLAASLVPIQQSGRVVGFLLSGLMMGVLLARSLSGLMSTLFAWNIIYLVSGFLLLVIAFILHRNIGLFAPTKSENYAKTIRSLPQIFIQNRRLRTRTYIGGFTFACVSLTFTTMSLLLAPAPYFFSDFTIGLFGFVGVLGTFVANFSGKFIDQGYIHKISIYCGIGLILSWVLFLLLPYHFAFYIIALLILYASLSAVHVTNQSIVFKLNQELRSRFNAIYMTGYFAGGALGTTAGSYAWKHFGWTGVCILGLIFAVLCLYYCVSDAKNQHN

ST25 4190 cg2_0028

MKTKITELFEIEHPIIQGGMHYVGFAELAAAVSNAGGLGIITGLTQRTPENLAKEIARCREMTDKPFGVNLTFLPTVNTPDYPGFVEAIIKGGVKIVETAGRNPEQVMPYLKTAGIKVIHKCTSVRHSLKAQQIGCDAVSVDGFECGGHPGEDDVPNMILLPLAADALEIPFVASGGMADGRSLAAALAMGADGINMGTRFIATQEAPVHANVKQAILNATERDTRLIMRALRNTERVMNNAAVEKIVAKEKALGDNIKFEDIIEEVAGVYPKVMIDGDVNAGAWSCGLVASLIHDIPTCDELVSRIVKEAEDIIRGRLLGVL

ST25 4190 cg2_0029

MMQQNNKTQSELKTGTIHKYFDRNILTLTMSNATKKNAINYEMYAALSQALDQAATNQDIHVVVLTGEDSAFTSGNDVNAFEKRDTTTSEPPASIVFLKSLATFPKPIIAKVNGVAIGIGSTMLLHCDLVYASQNSIFQFPFVNLGLVPEAGSSYILPRLLGFARASEIILLGEKFSAEQAKNYGLVNEIFEATELDQVVEDVAIKLAHKPSQALQLSKKLLRDMPIDDLLNRIDHESTIFSRCLQGCEFKEALSAFKEKRKPNFHNQTA

ST25 4190 cg2_0030

MNDAVDAEQTEKLLAPLRHELLHPIEDMQDENDRQFITALARGLELLRCFTPKHQHLGNQELAQMTGLPKPTITRLTHTLSRLGYIKQVPNSSKFQLSVGVLAFGYSMLSNVSIRSIAHPYMKNLADYAGAAVAMATRDRLNMIYLDVVQGKGNMTMRRQVGTYLPIHLSSMGRACLAAMPEDEREFLLNAIRNKHKEDWIKINRDLDKAFKDYQDFGYCFSIGEWHKDVNSVAVPLIHEQHGLLVFNCGGPSFIMNREKLEEDIAPRLLHMVNNIRTEIS

ST25 4190 cg2_0031

MGALNGIRVLDLSRVLAGPWCGQILADLGAEVIKIERPKVGDDTRSWGPPWMKDDSGQDTQEAAYYQSTNRNKLSVAIDIASSEGQELIKALIQDTDVVIENYKAGSLKKYGLDYESLSAINPKLVYCSITGFGQTGPRAEEPGYDFIIQGMGGLMSVTGERDDLPGGGPQKVGVAFSDLATGLYSTIAIQAALLNRHVTGLGQYIDMALLDVQIATMANQGMNYLSSGNIPKRYGNAHANIVPYQVFKASDRDFIIACGNDTQFIQLCRSIGLPDLPNDSRFARNADRIKHRDEIIGILQTHFLTKTADEWVDAIYAAKVPVGVINNLEQAFQEPQVIAREMLVEMNHPQREKLKVIGSPIKLSRTPVEYKTAPPLLGEHTQAVLGRVVSSEKLAELKEKGVIG

ST25 4190 cg2_0032

MIRDQETLNQLVDMIRQFVEGVLIPHENEVAETDEIPQEIVEQMKALGLFGLTIPEEYEGLGLTMEEEVYVAFELGRTSPAFRSLIGTNNGIGSSGLIIDGTEAQKSFFLPRLARGEVISSFCLTEPDAGSDAASLKTSASKRW

ST25 4190 cg2_0033

VQVKDGDFYILNGTKRFITNAPHAGVFTVMARTNFDIKGAGGISAFIVDSQTPGISLGKRDKKMGQKGAHTCDVIFENCRIPASALIGGVEGVGFKTAMKVLDKGRLHIAALSVGAATRMLDDSLNYAIERKQFGQPIAEFQLIQAMLADSKAEIYAAKCMVLDAARLRDAGQNVSTEASCAKMFATEMCGRVADRCVQIHGGAGYISEYAIERFYRDVRLFRLYEGTTQIQQMIIARNMIRAAS

ST25 4190 cg2_0034

MATNYKEGLSRIQPHTWKIAFIFAFLALLVDGADLMLLSYSLNSIKADFGLNSVQAGMLGSFTLAGMAVGGIFGGWACDKFGRVRIVVISILTFSLLTCGLGFTQSFLQFGILRFFASLGLGSLYIACNTLMAEYVPTRYRTTVLGTLQAGWTVGYIVATLLAGWIIPDHGWRMLFYVAIIPVVIAVLMHILVPEPEAWQKSRLQQPVVAQNAQKTSAFKLIFQDKRNRNMFILWALTAGFLQFGYYGVNNWMPSYLESELGMKFKEMTAYMVGTYTAMILGKVLAGMMADKLGRRFTYAFGAIGTAIFLPLIVFYNSPSNILYLLVTFGFLYGIPYGVNATYMTESFATAIRGTAIGGAYNVGRLGAALAPATIGFLASGGSIGLGFVVMGAAYLICGVIPALFIKEKIYDPQKS

ST25 4190 cg2_0035

MTTNNTQIQAVVFDWAGTTVDFGSRAPILAFMALFKDNKVEITVEEARAPVGLEKRDHIAAVLKMPRVAAAWQEVYGQAATEADIDRLYKDFIPYQLKSIPTCSALIPGALETFENIKKRGAKIGSNTGYASMMIGRLVKDAADQGYRPDCIVTASDVKFGRPYPEMLWKNLIQLDVSDIKTVVKVDDTVVGIDEGLNAGCWTVGLAISGNEVGLSYEEWSALSADEQIVLKDKAYQKMNASGAHYVIDSVADLPNVLDDIERRLGQGERP

ST25 4190 cg2_0036

MYIEVNAKEMSNIMNMQPQLDMDLENKYLLLTPGPLSTSATVRRAMQKDWCTWDQEYNSLVQDIRSRLVNLATSQPEKYTAVLMQGSGSFAVESVLGSAIPRDGKILIINNGAYGARMVQMARCLNIEVVELNYLETQTPDLQEIERVLEDKTISHVACVHCETTTGILNPIQDIGRLVKAAGKIWIVDAMSSFGGVPMDMAELEIDFLISSANKCIQGVPGFGFILAKRDCLEACKGLARSVSLDLYDQWNTMEQHNGKWRFTSPTHVVRAFYQALLELETEGGVAARYQRYLQNQQTLSKGMQQLGFELVLGEECPQSPIITTFLYPTAETFSFQSFYETLKKSGYVIYPGKVTDLNCFRIGNIGEVYIQDIEGLLAAIASYCENHLNQAVTA

ST25 4190 cg2_0037

MNNAQFDLIVVGAGILGLSAAIQAQEQGLKVCIFEKNAKPVGATRRNFGMVGTSTLTHPEQQWRKYALETRSFYQRIQTETDISFAQRQGVYLANTALEWQVLNEFAERANNYQIPVHLLKHDELVTQFSYLNPAQQFQGGLVFEEDYSVEPHVVGQRLLAYAQSQGVEIYTNACVVQTQYQQESCQVRLASGEAYRANKVLICHGEVIDVLYPDLLQSLNLKRCGLQMALTQPFHQNLNASLYSGLSISRYPAFEICPSHAELVKASQQGFIKEFGIHILIKQNEFGELIVGDSHEYYSINEAPQFEQREEINEFIQTYCHEKLGLNLPPIQKRWNGYYLTHEHELACVTEAEKNIFLVSAIAGKGMTTGAGFMKEILEQNIY

ST25 4190 cg2_0038

MLNEAASAVLKTQPRRKTEYSLRSNVALAAVAIALTLAIIAPLMMLFETAFFDENQNFVGLENFYNYFDSPALLSSVFNSVWVACAATIITVFLASIYAFALTNVNIKGKGFFKLVAFLPILAPSLLPSLALVYLFGKQGVFKPLLGDIQIYGPIGILISYCFWLFPAILMLMMVSFRSVDQRLIEASLSLGKNIWKTHYHVTLPAIRYGLISASLVAFIYVLTDFGIPKVIGGSFNMMALDVYKQIIGQQNMSMGAVISILLLLPAVFVFIFDRIQSKRHARFQAFQTKPYVSASNKKLEVVLSIFCGLVSGSILLIIFTAVLASFIQSWPYDLSLTLAHYSFEYVDGGGWAAYFNSVRMALFSTVFGTALIFMVALLTERFKAHPLIKNYVQALVLLPLAVPGLVLGIAYILFFNQQSNPLNVLYGTMTILVISTIVHYYTVPHLTLTNAIKQIPLQLDQAAQTLGTSKWKTFWKVYLPMCFPALCDVSVYIFVNAMTTVSAAIFLYSPDTSLAAVAVLNMDDAGDTVAAVAMSILILTTSCVVKLIHWLFTRKIMARSQQWRESAH

ST25 4190 cg2_0039

LQSSLLGQNQKGELAGHLLSTLTTYKYPEHGPSVLEVMDIQKKFQHFQALAHINLDLKAGEFVSFLGPSGCGKTTLLRIIAGLEKPDYGKVIKKGADITLLSAEKRHCGIVFQNYALFPNLNVEENIAFGLDKKRWDKSQRSQRIQQLLELIELPDIAKKYPNQLSGGQQQRVALARAIAPKPDILLLDEPLSALDAQVRLNLRQKIRSIQTQLNLPTIMVTHDQEEALSISDRVVVMNHGVIEQIDTPHNIYYKPQTQFVAKFIGTMNFLKATCVVPNQLEVLGFIPLNLEQQKLKAGQNYSIGFRPEAVELVDDFGSDKESLYLPIKVLSTEFLGAKRRLFCAIHIDGIEQAKHLLQIEIENTKFKSLQELMFIKVPNQLIHVFDEQGYALC

ST25 4190 cg2_0040

MKQYVQQTIKLGTASVLSVVIAIQMGCSSATSKQSEEITVYTAVEADQLKQYQEELHKAYPELKVKWVRDSTGVITAKLLAEQKNPQADVVFGVALTSLLVMEKKDMLEPFKPEGIENLKPEFVSQKPVPTWTGMDAWESAICVNKVELEKRKLPIPKAWKDLTNPIYKNLIVMPNPASSGTGYLDVTAWIQIWGEKQAWDYMQALDKNISQYPHSGSKPCKMAAQGEIPIGISFGYPAFKLKAEGAPLEVVYPTEGLGWEMEASAIVKGTKKLSSAQKFINWSVSKAANEAYAHNFSMVAYKGIENTTMDFPKNLPQMLVKNDFYWAAEQRERILAEWSKRFEK

ST25 4190 cg2_0041

LSVEHTVSSANHRMQLFDDRISTALYDSLINLV

ST25 4190 cg2_0042

MEQDQARIPKYIPIRDAIAHDIESGVLEAHAKLPSERVLSEQFQTTRVAAREALLALETDGLIYRLDRRGWYVRSPRIIYHPQSTKNFNQFVIEQGYEPSTEVISSELTQATSWDAKHLKVEKGHPIYSVWRRRCINGRPVLVEHLRVNAELFPEFLTHDLKQSITLLMANQYNCHITRADINLYPTALSEQQAKALHVNVGALGLYICRSNRNEEGVITDVDQEYWLHDVLDLHFEARA

ST25 4190 cg2_0043

MDILKTQVAIIGSGPAGLLLGQLLYKAGIDHIIVEQRSAEYVASRIRAGILEQVSVDLLKQAGVDQNLKEKGLPHSGIEILTNGELHRVDLAALTGGKQVTVYGQTEVTKDLMTAREAAQLTSYYEAQNVQVKDFYTAPKVEFEYQGKAFQIQCDFIAGCDGYHGVCRASVPEDKIKTFEKVYPFGWLGVLADVPPVADELIYVQSERGFALCSMRSETRSRYYLQVPLTDHVEDWSDEKFWDELKNRLDPESREKLVTGPSIEKSIAPLRSFVTEPMRFGKLFLAGDAAHIVPPTGAKGLNLAASDIAYLSSALIEYYAEGSEQGINEYSEKCLQRVWKAERFSWWMTHLLHRFETESEFDHKIKQAELSYVLGSIAGKTTLAENYVGLPYEIKQIDSFKHAS

ST25 4190 cg2_0044

MPSLDAFLEHPNSHEKIRQDDYIAGLAKGLALLEAFGTDRQRLNVTQVAERTGISRTAARRYLKTLKYLGYLETDEHYFWLTHRVLRFSSSYLSSAHLPKVAQSFLNLLCAQTSLTFSIVVLDDNEVVPIARSYLPQQDNLRVSPYGMHLGNRLPAHATSTGKVLLAALPEEAQHTWVKNYGLKRLTPFTIVDESKFFEVLKGISLSDYCLSKEEHELGVIAIAVPVFNAQGQAVAALNCMSQTNRVQEDYLVQQILPLLRNTANELRNVI

ST25 4190 cg2_0045

MVMAKILVLKSSIMGEGSQTNRLIDVMLEHRKDQGLQDDITIRNLAEMNLPVLDLEIFQALRGAENINQDIQQIVALSDELIAELKNTDLLVIGAPMYNLNVPTQLKNWFDLVARARQTFRYTETYPQGLVEGVKAVVVSSRGGIHVGQETEAVTPYLKAVLGLMGIHDVDFIYAEGLDMQAYRSNALDLASQQVKEFAI

ST25 4190 cg2_0046

MQHELSAISIFVTVVEAGSFVKAAEQLHLTRSAISKNIARLEEQLGVVLFKRTTRSLSMTDEGALFYEHSRRALSEIQNAAALLDQRKINATGRLRISVPVLFGQLFAAPLMVKFAHQHADLQLEISFNDRNVDLIEEGFDLCIRIGELPDTTQLVAKPIGEHRMLLCASPDYLNEAGPLENIEDLDLHATIADPHFGQMQKWRLQKENAEPLFIKPKAKLLLNDLQAIKNAALAGAGIAWLPDWLIQNELQDGKLVQVLDTMSSIAFPIHLVWPTLPFMPLKTRLAIDYLAQHLPSKLNKQA

ST25 4190 cg2_0047

MSNEKFEKGLAIRKQVLGEEYVNNSINNADEFNLPLQELVTEYCWGAVWGREELSKPERSLINLAMISALNRPHELKLHVKGALRNGVPKEKIREVLLQVAIYCGVPAAVDSFRIAKEAIKEFESEQ

ST25 4190 cg2_0048

MIMQLNELTLFRQQAFVAGKWCDADHQQTSEILNPATLEIIGTVPNMGKAEAERAIEAAKEAWPLWKNKTAKDRSIILKKWFDLIISNADELAFILTSEQGKPLAEAKGEILYAASFIEWFAEEAKRVYGDIIPSPYPDARIVVNKQPIGVVAAITPWNFPAAMITRKVAPALAAGCPCIVKPAPETPFTALALVDLAVQAGVPAEIFSVITGDAAHIGDAIFESDVVRKFTFTGSTPVGKMLLERSAKTLKKVSLELGGNAPFIVFDDADLDAAIEGALIAKFRNAGQTCVCVNRFLVQAGIYEKFIAALSQKIQNFNIGNGLEAGHDIGPLINANAVKKVEAHIQDALDKNGRLVIGGKRHTAGELFFEPTLIADVTADMDVATQETFGPLAAVFKFETEQQAVEMANATEFGLAAYCYTKDLGRAWRMSEQLEYGMVGINKGLISNEVAPFGGIKQSGLGREGSKYGIEDYLEIKYTLFGGLNI

ST25 4190 cg2_0049

MKKSYLQAALSTALMAVIYPSYAGISFGNTDDEYGKLTVSGYVRANYQDKHYGESASDQKIRFDAAQLKLDYERGPLFGHAEYRCYQYDHLCDFSTLIDGYVGYKLNKTDQVVAGVQPIPFGPGRFWGNSLYGSMSTTAGLEDVHNLGLNYHFELPTATKFDVGYFAVDGGNYTGSTQDSGRYTANYVSSDDPNKTDLQEKNMWVGRVTQDINLGINGLTTQLGGSYWTSDIENKAASSDGRRHAWALFGKANYGNFNVTLTGGKNDVTNQDQLNPNQSLMGSYDTEYYVANKGTFYTADVGYSFKNVKQIGNITPYFMYSHYDKDQDSMKDSIRNIIGVSIDHKQLSLVAEYIMSKNDPFIGGTQDSLAKGDDNQWNKLLNLTLFYYF

ST25 4190 cg2_0050

MSQNENSNNAFAIESHSIDYVAPSERNGKVRDLFTLWFCTNIAPLAVITGAMSILTFNLNIVSALLAICCGHFFGAAILALTSAQGPQVGIPQMIQSRAQFGRYGALLVVLFTTLIYLGFFISNIILSGKTLHTVVPAIPVPTATVIGAIAATTIGVIGYHFIHKFNKIGTWFMGGSLLIGLMIMVPHINADVLAKGSFNMKGWFAMFGLCAVWQISFSPYTSDYSRYLPKSIGIYKPFIFTYLGASLGTIFAMAFGTIAVSIGSTADAMEAVKSGTGLFGYVLMILFLCNIIGHNAMNLYGAVLSCITSIQTFAGQWMPSRNIRVVLSIIVLVLATLTALWASSNFISFFLNVIFALLFILVPWVSINLLDFYVVNKKSYDIQSIFARDGGIYGKFNAKALTAYFIGIAVQIPFLKNAFFTGALADVIPDVDISWVIGLVVSLVVYYVFNLGKIKCSAPALNAKLPQ

ST25 4190 cg2_0051

MTADFQLYINGQFESGAATFESINPATGEVWAHMPEARTEEVNRAVQAASQALKAPEWAGLTASQRGKLLYKLADLIEKAAPQLAQIETSDTGKIIRETSSQIAYVAEYYRYYAGIADKLEGSFLPIDKADMQAWIVREPVGVVAAIVPWNSQLFLSAVKVGPALAAGCTVVLKASEDGPGPLLAFAKLVHEAGFPAGVVNVITGFGPECGAVLSSHPDVAHVAFTGGPETARHIVRNSAENLAKVSLELGGKSPFIVFADADIQSAVNAQVAAIFAATGQSCVAGSRLLVEESIKDEFVQRLVERVQTIKIGLPHEMATEFGPLCTLRQRQKIEQVVASSIQQGAKLLTGGKSLERAGYYYPPTILDCTDAPQADCITTELFGPVLSVDSFKDEAEAVQKANSTPYGLAAGVFTTNLSRAHRMTKVIGSGIVWLNTYRAVSPLAPFGGHGLSGHGREGGANAVLDYTTTKTVWLRTSDEPIDDPFVMR

ST25 4190 cg2_0052

MRFSLFVHMERVSDQQTQKQLYDEMIELCQIADRGGMHAVWTGEHHAMNFTIAPNPFLNLVDLANKTKNVRLGTGTVVAPFWHPIKLAGEAAMTDIITNGRLDIGIARGAYSFEYERMVPGMDAWSAGQRLREMIPAIKNLWKGDYEHNGEFWQFPKTTSAPQPLQQPHPPIWVAARDPNSHEFAVQNGCNVQVTPLHLGDEEVEKLMGHFNAACEKFSDVPRPEIMLLRHTYVADSEEDAQLAADEINTFYNYFGAWFKNEREINQGLIAPLSPEEIAAHPFYTPEAMRKNNVIGQAQEVIDRLKAYEAMGYNEYSFWIDTGMSFERKKASLERMINEVMPAFA

ST25 4190 cg2_0053

MRYRKENHLKTYGVYYGNQTTGTVRQIVKEEV

ST25 4190 cg2_0054

MQNKSNDTAPRHFNLTARILHWLMAAAILAMLFIGVGMTTSMTWRPWLIDIHRPLGIAILLLVIIRLINRLCYPIPPLPPTVPRWQAFLAHASHWLLYILMFSLPLLGWATLSAGNWPVTLFPGWDLPPIAPTNSTLYAWFRTLHGILAWLLFAVVIGHLSAALLHAWIYRDGVFSSMALGPSSKKEEAD

ST25 4190 cg2_0055

VGIAVIAGALVLAFAWTAGLFGHRTTSKTFLGDTLKNFDPGYRRAHGKGICFDGTYHSNGAAAALSKATVFTKSDIPAIGRFSIGSGNPHAADNSTATVSMALLLSPADHSQWRMKLNNFPYFPTRNAEGFLAQQKAFKPVPSTGKPDPALVEAFLKEYPEARKSIEQKAKMPLTGSFSGAEFYVVNAFILRAANGQEQPVRWSMRPHSKFISLTKEQRDQADHDFLFKDIKKRLAEGPLYWDLVLQLAEPGDPVNDPSQPWPKGRKEVIAGTLEVKNVTDQVNGACRDINFDPTLVPPGIELSDDPVLAARAAIYSQSYNARLREIGFGKATDAVGK

ST25 4190 cg3_0056

MHLKTLSFGLAVALASTVTLAAPVDYKIDPTHTATVFSWNHFGFSTPSANFSDIQGVIKVDNAKPANSSVNVTIPLSSVNTNVPALDKEFQEEAWFNAAKYPNITFKSTKVETKDKKHFKITGDLTVKGITKPVVLDAVLNKQGEHPMAKVPAIGFNATTSFNRSDFGLGNYVPNVGDKITVNITTEATAASAAKK

ST25 4190 cg3_0057

MVNTAQAQSTSTPYRVTLTDPSGHQWFADEPTDKGGQDTAPNPVQLLLSALGACTTITLEMYANHKGIKIEHVQVDLALNPNGDPEAGQNNIERKITLKGEFTEDQHKRLLKVAENCPIHKLLTSNITIQTELNI

ST25 4190 cg3_0058

MTTPLNERRIANAIRVLAMDAVQQANSGHPGAPMGMADIADVVWREFLNHNPNNPQWANRDRFVLSNGHGSMLQYALLHLTGYDLSIEDLKQFRQLHSKTPGHPEYGYAPGIETTTGPLGQGIANAVGFALAEKTLAAQFNKDDLKVVDHFTYCFLGDGCLMEGISHEVCSLAGTLQLGKLIAYYDDNGISIDGEVEGWFSDDTEERFKSYGWQVLRVDGHDADAIRQATVEAKAETQKPTIIICKTIIGLGSPNKQGKEDCHGAPLGKDEITLTREALGWTEEAFVIPADVYAAWDAKAKGNEAEAAWNEVFAQYQAKYPTEAAELLRRISGDLPAEFSAQADAFIRETNAKAETVATRKASQNTLQAFGPLLPELLGGSADLAGSNLTLWKGCQGVQENPAGNYVYYGVREFGMTAIANGVALHGGFIPYVATFLMFMEYARNAVRMSALMKQRVIHVYTHDSIGLGEDGPTHQPIEQIASLRGTPNLNTWRPADTVETAIAWKSALERKDGPTALIFSRQNLPFQTRTEEQIQNAAKGGYVLAQEKGELKAIIIATGSEVSLAMEAYAQLEGVRVVSMPCAEEFMKQDAAYREAVLPAHIRARVAVEAAHVDYWWKFVGLDGKVIGMTTYGESAPAKDLFQFFGITTEAVVAAVKELTA

ST25 4190 cg3_0059

MADQISDAILDAILKEDPYARVACETLVKTGAVVLAGEITTTANIDVEAVVRQTVNGIGYHHSDLGFDGSTCAVINMIGKQSPEIAQGVDRQKPEDQGAGDQGLMFGYASRETDVLMPAPISYAHRLMERQAELRRSGALPWLRPDAKSQVTFAYENGKPVRLDAVVLSTQHDPEITQTQLKEAVIEEIIKPIIPAEMFHAATKFHINPTGMFVIGGPVGDCGLTGRKIIVDTYGGMARHGGGAFSGKDPSKVDRSAAYAGRYVAKNIVAAGLADKCEIQVSYAIGVAEPTSISINTFGTAKVSDELIIQLVREHFDLRPFGITRMLNLIQPMYKQTAAYGHFGREGSDTAFTWEKTDKVEALKDAAGL

ST25 4190 cg3_0060

MDYEYGVNRNMDMIYQLMPQLCRNKHNDIDIA

ST25 4190 cg3_0061

MTQLINKGGFRERANRSRKYQQSENKQVALPSNQYQPQTKLQDNQNEMIQAKASTAETSDD

ST25 4190 cg3_0062

MNLLLIVVLGAIVEIAVWIGVAQFISGWYVFFWFIIAFFIGLTLIRNSTAGIMPQLQQMQMTGQLGNDPTVTKKLSLMLLRGFLLMLPGLVSDVLALLILIPGVQTAFRNIAMRTLAKRQQAMMNKMMGGMGGAGAGQNPFADLMRQMQDMQNQQGGGSQYHDSSVIDGEAREVTPDHKKIEHKNKD

ST25 4190 cg3_0063

MNIKALLLITSAIFISACSPYIVTANPNHSASKSDEKGEKIKNLFNEAHTTGVLVIQQGQTQQSYGNDLARASTEYVPASTFKMLNALIGLEHHKATTTEVFKWDGQKRLFPEWEKDMTLGDAMKASAIPVYQDLARRIGLELMSKEVKRVGYGNADIGTQVDNFWLVGPLKITPQQEAQFAYKLANKTLPFSPKVQDEVQSMLFIEEKNGNKIYAKSGWGWDVDPQVGWLTGWVVQPQGNIVAFSLNLEMKKGIPSSVRKEITYKSLEQLGIL

ST25 4190 cg3_0064

MNYSLDSNFRLIDCNEAEHAVAILEILNDAIVNSTALYDYVPRSLDSMKTWFSVKRENGFPVIGVVDELGKLLGFASWGTFRAFPAYKYTVEHSIYIHHEHRGCGLSKVLMQALIQRAQQAQLHVLIGCIDATNQASIGLHEKMGFTHAGTFKQVGFKFGQWLDAAFYQLILNTPFEPVDG

ST25 4190 cg3_0065

MDDINIRIAHRIRELRLARGYTLDVLAARCQVSRSAISLIERGETSPTAVVLEKLANGLEVPLTQIFTNEAGNSSSQPLIRRNEQAEWRDPETGYIRRTVSPPNLKLPFQIVEIEFPPHSRITYETNESSRVVQQQLWVVEGKIEIQLGETSYALGQGDCLAMQLDQPVIYSNHSAQVARYILVVSNQLVPALKESL

ST25 4190 cg3_0066

MSNLKNAAMNGYFSVFNPKDLYCPPNFPYIHNEEFEII

ST25 4190 cg3_0067

MPLIIGIDPGSRLTGYGIIEKDGSKLRFVDAGTIRTETQEMPERLKRIFAGVERIVKFHGPTEAAVEQVFMAQNPDSALKLGQARGAAIAALVNLDLQVAEYTARQIKQSVVGYGAADKEQVQMMVMRLLNLTIKPQADAADALAAAICHAHASGSMSKLTVLNALGGMARGRSRSSSRRR

ST25 4190 cg3_0068

MRDVLEDVINRKMQEDKNMQAVVYHSQDIQAWEQRWFAAQNSSLGLMQQAAWSITQQLIVLFQKQNFKNIAVWCGQGNNAGDGYFIASYLKKAGFQVEIFAADLGESTDLHHAVDFAKKNDVQVHQGFEVSKTFDCHIDALFGIGLNRELNGYWQHAIQQFNGQAGLKISIDIPSGLHANTGSTLSCAIRADHTFTVLGLKAGLFTGQGKEYAGQIHLINLIPIDQELKPLAYLTPTHIKLPKRLAFGHKGSYGHVLVIGGHEQMGGAVIMAAEAAFHAGAGKVTVVCHSNHHQAILSRAPNIMLRDINDFDENGIKEILSQVDAVCFGMGLGRDEWAHQIYQQWFNYLNQTSHLEVVLDADALWFLAKQPEKLSLHIYATPHPGEAATLLGCSTWQIENDRIAAIYALQQKYAGQWVLKGAGSLILEDNLYICTQGNAGMGTGGMGDVLAGMIASLKAQFHKAVTLHEIVTLHAQAGDQLAKQGMRGLQAYEMNQAITQVVNQ

ST25 4190 cg3_0069

VQQIDPHELKAWIKAQALDLGFADCVIAKPDAQEQMPCFLEYLERGYHADMTYLEENLEKRADPTLLVPGTKSIICVRMNYLVESPKPRYVPFEPNSAIIARYARGRDYHKVMRGRLKTLATRIREKVGDFESRPFADSAPIFEKSLAESAGMGWTGKHTLLIHKKSGSFFVLGELFTSLDLPFDEPATSHCGSCSACIDICPTQAIVEPYMLDARRCIAYLTIEYKGIIAEELRAGIGNRIFGCDDCQLICPWNSFAKTASIPDFNPRHGLDNISLLDIWQWDEATFLANTEGSPIRRTGYQSFKRNIAIGLGNAPYSKEIVDQLNKGKSLHDEIVNVHIDWAIEQQLNQL

ST25 4190 cg3_0070

MDMTMTLRNDWTREEIQALYEQPFLDLVFKAQQVHREHFTANTIQVSTLLSIKTGKCPEDCKYCSQSAHYDSKLEAEKRIAVEKVISEAKAAKDSGSSRFCMGAAWRNPHERDMPYVLEMVREVKALGMETCMTLGMLNQSQAERLKDAGLDYYNHNLDTSREYYSHIISTRTFDDRLNTLDYVRQAGMKVCSGGIVGLGESREDRIGLLHELATLPIHPESVPINMLVPIEGTPLADVEKLDVIEWIRTIAVARIIMPHSYIRLSAGRESLSDSDQALAFMAGANSLFSGDKLLTTPNAGEGKDQALFNKLGLTAEKPKPTVSDLSVDAMSA

ST25 4190 cg3_0071

MSKDFKALTYIADDNIADTVIWLLHHQDVFESFHFDVLSQELSVTHAAGRDIIRVGTFLNATYGILVTSV

ST25 4190 cg5_0074

MNMMTTLNLRALVTNDNGEPKTTSYAVAEAFNKSHSHVMRDIKKIIKQCGEEFAKSNFGLTFENKKIGNTERKTPFFRISKDGFMLLVMGFTGEKAMKTKIEFINAFNWMANQLSQVFQSKWARYNSVSHEYQSKKDHISCSARDMRAWRDEKPVLEKELSQLEMELQPSLLQSMGSI

ST25 4190 cg5_0075

MLTLEEIEIKRQELERHLHDMMGAELQKWQRENKLCISDVNIRLASNHCLGGPKQNIVTGVSVDLDYKP

ST25 4190 cg5_0076

VQSLVVSSPVDTGAYRASHIVSIGSGDYGVREPSTNALQDAAIQAVKFKLGSLIYIQNNQPYAERLENGWSDQAPLGIYSTTFTYITQKYGG

ST25 4190 cg5_0077

MSASKSVGAKMVNPDYVPEWYISPFQHVQYTLARNQLHMDLLFEDMDKDDQFLDMGADAQVSTFSDGAYAIVQIGDTADKDRIQVYGLLLHEAVHVWQIVKKRMGERDPSVEFEAYSIQAIAQDLFEMFEASEVKKHGVEGSKAEQL

ST25 4190 cg5_0078

MGLHDEIQADITEAFNEDLADAVHSFTCDRVISTNWNPKTNTSENIIEHYEGRGVLFGSYSQYEIQTLGVLATDKKATVLQNEVSMTPKIDDEWLTALGSFRVIHIQQDPASTIWKCQLRKV

ST25 4190 cg5_0079

MSFITVDDANSILGSDFAPDSDKARLVQLANVWMKKRIGFVPDPIDPLLKDAACEIIKGILAKEIYNGKDQQLKRKKVKADSVESEKEYQDGSEAISSFEQIAIDFIDSLDLKDPNASFNGFGIPLYRA

ST25 4190 cg5_0080

MKLIYTRIAAAAALEVGTIANPDYYENPNRSAEEVIIYGDYPKIQNDYEALDIPVEVRKLEEPVKTTLATVSAVIGITPELQKVVDETKAECEKVVEENTQLKQKIAILEQAGGNQSELSSENSRLKDAAVLADKALKDAEAQVVGIKAEFEAFKNDIPAMQTRIAELEAGKAEENPATETAANDFENWSNDQLKEYLASKNIGYKPSATKAELLKLIPKE

ST25 4190 cg5_0081

MTTTVNSDMIIYNQLAQTAYLERLQDNLNVFNQASNGAIVYRNEIIEGDFNKEAFYKVGGSIKHRDVNSTAKVVPEKIGSGESVGVKVPYKYGPYASTEEAFKRRARTPEEFAMILGYDLADALVAGRLQYSLASLKAAISSNPDMVAQGSIAVDGRKALTRGMRKFGDKFGRISLWVMNSDTYFDIVDDAITKQIYGESEIVIYGGLPGTLGKPVLVTDAVGDDDAFGLQIGAVTVTESQVPGFRAYDINDEENLGIGMRAEGAFNLDILGYSWDTSKGENPDLTLLGSSANWKKHATSNKMTAGTLLDLSGTTTG

ST25 4190 cg5_0082

MKLKTVTIDGKVYAEVDGDKPIYIHDDGKEMPHDAPHSVATIARLNNEAKTHREAKEAAEKALKAFEGIEDPVAAKKALQTIQNLDDKKLVDAGEVEKVKAEAIKAVEEKYAPIVEQRDALEASLHKELIGGGFARSKYIQDNIAVPVDMVQATFGHHFKIEEGKVVAYDPNGEKIYSRVRPGELANVDEALESLVGGYQHKDLILKGGKGTGGGFQGGGKGGAPAGMKRSEMSVSQKADYIKEHGNDAFLKLPN

ST25 4190 cg5_0083

MKMTEEVTEQELAEKSVAPRVTKAQIDALMGRVTYTVEQRPGGTTSTFVHAFLDGKFFLATGFSACVNAENFDAEIGERMARGNAEKLAENKLWELEGYRLFAKNF

ST25 4190 cg5_0084

MNCDVFSGPATITATFDVGGPQGIRLVGDEPRQEVWRVKET

ST25 4190 cg5_0085

MAADIEKLFEALTQHQAYLYRASSKTVNELLALFNDDTSKMLSKLRDLLDELNESEKVALAGGKYTTSNLREIRDLISQWFASVNLTLPEAFAVSATALAVYEANYVAKLYGAKINKPDGEKLFLSAKKVPLAGGALVDDLLSRIAESARQKVEYAIRDGINSGKTNQEIVQRIRGTKRLNYEDGILNGTKTDIERTVRTVRSHVANQAYLNSFNQIGFEYVRFVSVLDGRTSKLCASLDGSVWEINDPAKRVPPLHPNCRSILVPVEKDGKLVGERPFVMDERRVKDIPKDERSQLIGQLDANTTFKEFFKKTDDFFQKEWLGPKRYKLYKEGKFDFDKFFDPEGRLYTLDQLRKLDEQTFKELGL

ST25 4190 cg5_0086

MSDVTFKHPEYVKNLPYWQKLDDVCEGEDAVKAKGEKYLPKPNAHDQSPANKSAYLAYLIRAVFYEVTGTTSNSLVGAAFATDPSFKFPPELAHLERNANGAGLSAYQLAQNGIRHLLKHYRCALYVDYPAVTPARNLAEFKQQKAYPMIHLLNAIDVINWDSMMIDNQKKLCLVVIREFTSERGGDGFSKTEVEQYRVLRLEPNSEGVYAYSVQVYTKGDKGTWVGGEKKFPTDYNGDTWSYIPFTFVGAIDNSEEIKKPPLLPLANLNLAHYRDSADFQESVFYMGQPQFYAKGVNWAWYDEAKKRGIYIGAKVLLPLPENGDLGIVQAEPNTLAREAAKDKWEQMKEMGARLIEKGSAAKKTATESNSDDAVQHSVLSLCVVNMNEALSMALRWAAKFVVPNVDVLTKDELMFEISQEFNKQGYLAELARQLFEAALQGRSSFKSWWEYNQTGMFPKQKYEEELQNVEAEQDGTLNQKVE

ST25 4190 cg5_0087

MFKERRGYKWMQNWHHLEICEALMKVYRGEIKRLIINVPPRYSKTEIAVINFMAWCFGKKPDCEFIHISYSAMLAANNAFQTRNMVQEKAYKKVFPDLKLREDSKAKDFWRTDAGGVCYATGTGGTITGFGAGKMREGFGGCIIIDDPHKADEAKSKTIREGVIDWFQNTLESRTNSPETPIIVIMQRLHEDDLAGWLLGDRKDGVPVAGGNGEVWEHLCLSAIQEDGSALWPAKHNIQKLKQMEQAAPYVFAGQYRQMPSPPAGGFFKPDNIEIVDALPADVVKQVRAWDFGATENEGDFTAGVREALGADGFTYIVDVTKGQLGPDNVNKRLKQVTELDGMGVTVRIPQDPGQAGKSQASAFVKLLAGYDVKAKPVSGDKLTRAQPFAAQVNVGNVRMLRGDWNKDFIEELRNFPNGTHDDQVDAGSDAFNELNGGFEAFFADMGFAR

ST25 4190 cg5_0088

MATLKEPVKIFIVQSLACRDTPQEVAELVKQEFGVDIDRVQVATYDPTKVAGKNLSKKYVELFEKTRDEFDKGLIDIPIANKYYRLKQYQRQLEKTRNVKTALKILEQAAKDIGGQFTNRQEITGKDGGPVQTVNSEIPVPMEDYLKARREVLDEY

ST25 4190 cg5_0089

MDQIRPFPPTDFIDQAEEEEAIRLIPAPDLKKWVVANFLTLGGPLHNPDHDHIAELLHDNEEFLAFAWASSAYTRAKRMVLGQCEKVMFQQGGWKKARQEQQMRDWFGFVPTYLITIDATFCDKANDNEFCALLEHELYHIGVERDEDGEIIYSDHTGLPKHYLAGHDVEEFIGVVKRYGPSKNVKRLIEVAKNPPFVSNLDISKCCGNCVIN

ST25 4190 cg6_0091

MFEMDKCREEFEKTRTFKSIYSTLMYFEESLDLYTTKEKWREEDAWRLTYAWWAYKELQTRIDSKREATKGGFSQWFKTTNYFGAYFTDLDLTLNHRDQFENDIINSAWLAHQSRQTEVDELQKRVDQQGLIIAKAMSIASDLQKSWSMFEIGKKLEQAIKGEG

ST25 4190 cg6_0092

MKDFAIAIIYGAALFVSIKYAWRWYNGELSTPAIMEWFGRGFFFAWGVIAATLTMVLVIRLITEYVK

ST25 4190 cg6_0093

MEIFFLIIGTLAAFSGGKDHPSGHNIALAIMTIGCYACAVAMIKI

ST25 4190 cg6_0094

MNAAVVTEKLSNLEWVGQQMRAKTASYETSTASTGEKAPTWEERCGAISSIEDEATKAYCEMLVWGDSRDTTQAFKTLVEHIGEILHEAASKERQRHHFDLKLFCMKVARMQVFFKMRPVIKEDRTLQGQLKFCGIDEIKADTYSKNYAYLGAMVDIILKDMEDEIDFYVGQYRKKLNN

ST25 4190 cg6_0096

MTPKEKQDIYEWNTAHNELHEEFMEGFEEPQFIKGFKVGIWLAFCAAIIWIFWHFLG

ST25 4190 cg7_0097

MDNFEEAWKANRHVWESTKNLAKAMYLAGQQSQQAKVEELQNLYTQQGINMLKMQRRVDAALEQVQGNLKYVEQDRRENFEFLQMAMIRAFKELEQALKGENNEF

ST25 4190 cg7_0098

MNFDNEMIKGISQSDFEKAFAKQMMNDRVSDQMQKDMEALQKLNSGNYVIVPKEPTQRMLNAGHVAMNPVKGSDVHSGTNQKRRECYKAMLRAYQEYGDQ

ST25 4190 cg8_0099

VFFAVITLFPEMFEAITAYGISGRAAKRDIVQVTCINPRDFAEGNYRRVDERPFGGGPGMVMMAEPLAKAINHAKQLASQAGCVHVPVVYMSPQGKTLNEQAVQQFVDYDGLIVLCGRYEGVDERLIQHYVDQEWSIGDYVLSGGELPAMVLLDSIIRRLPNVMSDEQSAIQDSFVDGLLDCPQYTKPDQFEGLEVPEILKSGHHANIEKWRFLQRYQRTLERRPELIEQVTLTKQQKKWLSDEQG

ST25 4190 cg8_0101

MTPTQNVPEDRIQIGQLRSAYGLNGWLWVYSNTEPMSNMFDYLPWYIETKAGWQIVDVKRWKPHGKGLVVALKGVSDRTGAESLVGANIWIAKSQLPKADVDEYYWSDLKGLTVLGLDDDEQEVNLGQIHELFETGANDVMVVRATPDSIDSEERMIPWHKDVVQRVDLEAGRIYVNWGVDY

ST25 4190 cg8_0102

VLPMVGAGIIRKNYKAFIMVVIRLARGGAKKRPFYQIVVTDSRNARDGRFIERIGFFNPTAQGQAEKLRLDADRFAHWVSQGAQPSERVASLAAQAKKATA

ST25 4190 cg8_0103

VKNGFSLIEIMVVVAIVAILAAIATPSYLQYLRKGHRTAVQSEMMNIAQTLESQKIVNNRYPSDATIQSIYGSNVSPIQGQALYKLAFATLNDSTWVLTATPISTSSQAGDGIICLNDQGQKFWAKGATVCALSASSSWTE

ST25 4190 cg8_0104

MVVIVIVAIFASIAIPSYQSYSRRATASAVKSEILKLAEQLEQHKSRNFTYRGFTTTSITLPRGGYTIQITDDTTTGNLLTNSAANGQTWVIRATTTDSRNFNFIAKNSGIRCQSLTVTAVDNDCGGTTTCNTCEANSEVWQ

ST25 4190 cg8_0105

MKLKLKNFKPNNLWYAVYSSSMIFTWLMTSSVVQASDLQIYASPTAGKKTIVMMLDTSGSMTNNSYGENRLAMLKNGMNAFLASNNPVLNDTRVGLGNFSANGDSRSGQILVAAAPLGDASTLNTVGSQRYKLKQAVANLTAGGSTPSAHAYAEAAAYLMGTTTYSETNYAIRKDSYIKRVRRSDNRTEYSYCTNYRDSQIDTANLWQPCRSNSYWSSWSTNNPGVDNATAYDTSSDWTYYYTTFNYAVANADSGIPKSKSNDTASNPNIVVDRNATNSNAVYQSPLPAVANRQSCDGQGIYFLSDGEPNNTTNTRSASVMSTALGSTFGADFNCSGGLSNTTADSGWACMGEFAKRLFDKTKNPAGVSIQTAFVGFGSDFSSLSSSDVKNACRLSSRTQSDRKGDDACSPNQSTNAVAAPGYGNGGFFPTQSSQGVTDSVIAFINNLDKVPLEPLTTGAISVPYDALNPKNLQEYGYLRAFEPNPANTYLTWRGNLKKYHVVLSGANAGAFEANSGGLVYNASGAFRTGTKDYWNSSTYTDGGKVFLGGSYANVPLPIAGQPETRDAEGNITKYYYAVQSKIRNLFTDVSAVAADGSLTKISTSGTNLLKIPAAPPEGTNPFDTVANTASYVLGKFDPSTGQNILKAFPISLKLKILNYLGYSTDINATTLPSSLVTSNEPYLSMGGSIHSLPVQLTYNGTLDDNGNLTSAREQSILYGTMEGGLHIVDASSGIEQMVFVPADILNDSVASKALVVGQSDASAPAHGMDGAWVSDPAYNITTVGSGSSAVSKVTAKQMNIYGGMRMGGSSYYGLDVLSPTSPKLLFRIGADQTDYSRMGQSWSKPVLANIRYNGSIRRVLIVGGGYDQCYEKPNITLTDACFTNGKAKGNAVYIIDAKTGQRLWWTSDTGSNTDNANMKHSIVSRISTLDRDADGLVDHLYFGDLGGQIFRVDLNNNQTKTNSTYSSFGVRVVRLANLATNDSTYDGTNDYTGGNAPRFYEPPTVTIHDYGIHTFITVGIASGDRSTPLDVYPLTGREGMTPASALSGRPVNNVYGIIDRDFVKKNLMSLTDNQLETKDITRTGLRKNPQILRTGETRVAQIFFPTTGVGKGGWYRSLSSTSDGTEKANNSFRIKGGLKAFEEPMAITGNLIIPVYDPQGTGIVAADPCLPRVVGETDRQTYCLPFGACLNPDGTINSNKENPSGFQTKTGSNCPAGVSECNTNVIGAGIRNITFVPKRDEPPVTNSCGKLQLSGNENGTGEWQCTSHLLPVRWYERYR

ST25 4190 cg8_0106

MTHLNYKNIKGRSKETGATLIVVLIILLIVISVGVLAIRVAIVSLKVATNSQVGQLNFQSSDTPLELIVQMNPTTLTNITNVIGAALKAHESNPGAEYNFCYKPTSTSVSFAQTRGASLLRAGSANNAVVEDGGVAGFCDLTSDYGSNRQAVVTQVAVSIPTDAMPDIPGSNLPRDINLSEGTQLPKSMLSTQRIRVITTAFLPAYASTSLETLQSDCLSTSSAKISDNFDSALTDKQTLADCLANHNVPFSTQVQEFNYTNKLTEITAPGS

ST25 4190 cg8_0107

MAQSFISNKEQGFTLIELIVALALGLILVAAATQLFIGGLLSSRLQKANAEIQDSGIFGLEYMARDIRLLNYGNVVNPQLTDTTPRGGIVLTGSTATNANNINFIPKVDTNTYIPEALLSRGAGDTVSTANNHWKGLSNIQNSSNAEVQSDQLTIQFIAPTNMTNCEGVNVLAGDLIVQRYFLRVDNNGSSQQDYALACDANTPAVSATAQPDIVNGLGDAGQIILPRIDHFHVLLGTKNAAGNFAYYTIPQYRVAAQAARDASPAVAAPRILSIQISVLARSTNNAQNKAIDPNQSFLMLDQNVHAADNRNRFLRRVYSVTIALRNAMGETI

ST25 4190 cg8_0108

MHHYKQKAQAGVGLLEVLVALILLAIGVLGYVALQLRAMDASSEALSKSQAILVMRGLAENIRTNSTQASQYPTFVRSYSNYTSDTPAPTSCFNSLCTASQLAQFDAYQAARNANQLGMRITMSNCPGVTNTMVQQRQCLFVFWGKTAPVITTNGTNTSVDVSSCMSNNGVYVNNSTCLMMEAY

ST25 4190 cg8_0109

MRGIIPQEGFTLVELMVTIAVMAIIALMAAPSMSNLLESKRLDGNQRDLINTLSEAKSQAILGRQNVSVNLNSTASNTPTSLNWQTASNNTLELKNIAADGTQSSLTTTTLAFNANGVVANITQDTLLSICNSRINKKKVIILTKLGTLVFKAEETC

ST25 4190 cg8_0110

MEIVLANPRGFCAGVDRAIAIVNRALECFNPPIYVRHEVVHNKFVVDDLRQRGAVFVDELDQVPDDSIVIFSAHGVSKAVQQEAERRGLKVFDATCPLVTKVHIEVTKYAREGTEAILIGHEGHPEVEGTMGQYDKLKGGDIYLVEDEADVAALEVRHPEKLAFVTQTTLSIDDTAKVIDALRAKFPNIQGPRKDDICYATQNRQDAVRDLAEKCDVVLVVGSPNSSNSNRLRELAERMGKAAYLVDNADQLEQSWFNDTCKIGVTAGASAPEILIKQVIQRLQDWGAQAPKELEGREENITFSLPKELRIHVTQA

ST25 4190 cg8_0111

MSGLLFVVSAASGTGKTSLVKALLDRVSNLHVSVSHTTRGQRPGELDGVHYHFTTKDEFLDQVNQGGFIEYAEVFGNYYGTSQATVKQQLAQGHDVLLEIDWQGAEQVRKLFPESKQIFILPPTQFDLRQRLSNRGTDSVEVIEHRLSCAVEDMQHYVNFDYIIINDDFNKALHELEAVITANRLVLSQQAKRHQNLIQDLITPQPKQE

ST25 4190 cg8_0112

MEFWKFIQEFLQTFGTAITSFFMGFIMAYFRTKKKLGKADWAESIMCGLFSVGVWSLLEWLNVPQIVSVGIASGIGYMGTHFVSNLIEKRVNRNE

ST25 4190 cg9_0113

LKNDLTKFEVAINKLVKVPLTQNQFDALASFTYNLGETNLANSTLLKKLNKSDYQGAADQFLVWNKAGGKVMKGLVRRREAERALFLKK

ST25 4190 cg10_0114

MKLFYIIVILTSGILLSACEKRQFEASSEDQVENTLDATVSRKVLSTDNLADIKHDFIYLDSIINAPELVNLKEDMGRRINEGSSLSSALAYGKTTTELYYNKLNEFEYKSSEISAIKDKLQEGLQLSIEAYDISSKVKFNDRGYPEDLEKWDELDELQQRMNQSADELNEDFKAVRLKLEKAAETAEEKVES

ST25 4190 cg10_0115

MQQLMIMVTEVGKLEHTCNLLAEVNKGGKVIKVFDYNGNQLPINIDGTVTFNRRRWELPIKVDLK

ST25 4190 cg11_0117

MIEFVDYTSMMKLRRAYNLGTRNEETRAAANLYEKLRKLKMLDQLKQEAMTKRYKEAV

ST25 4190 cg11_0118

MKDLRDYVLDAPNNTIQCYVQELIKSGYLEKDSYATYKATQFAKDLLNVKGELKA

ST25 4190 cg11_0119

MKKVISFSGGRTSAYLVYLFKDNPDAHFVFMDTGAEHPATYQFIRDIVKHWAIDLVCLRTVVDPEMNKGVSYKIVAIDDLKQDLEPWKEMLKKYGSPYYDMPFCTARMKTEPFEKYCNDVFGKNNFERWIGIRFDESKRLPIEVLEKLSLPIHKKASHQKSGFRYLAEICELDKDQILDWWEEQPFDLAITEHLGNCVFCIKKHLPKVALAAKDEPEQAVKWIEVTQGAEVRSEGRKYNHHRMYRERLHMSDVIEAFKDHDRDELYKALRSSKRYESGSCSESCEAIV

ST25 4190 cg11_0120

MAKEFDCPVIALAQLNRGAEGHKPVASDLKDSGQIEQDADQIIMVHPILEKEANAPTGVTELIIAKNRHGKRGSVNVQDRLDICRFVGMSFPVEERGAA

ST25 4190 cg12_0122

MNAPANGTLITTQIANVAETLGLVNVNPQELKETLIQTAFRTETPATDAQMASLLIVAGQYKLNPWTKEIYAFPDKNKGIIPVVGVDGWSRIINGNSNFNGMEFKFSENMVQMEGAKVAAPEWVECIIYRKDRDHPTVVREYLAECYRAPFKAKSGYTVEGPWQSHPSRFLRHKATIQCARLAFGFVGIHDQDEAERIAESGQPIKDVTSEVPEGYQAFEDEHLATLKSEAQYGTERLQAAYVAIPKGNLKKHLWEVHSISLKEIAQFADKALQRQGETYEHSPA

ST25 4190 cg12_0124

VQHNNEYKVAKYGQWDGYPSGQGAGILEFLKGEFNKALFIQKLDNIFEPTDEQVKAWYRDAGNTRDDGYVDYDVSKRFAANHPSLSRGAGSDILGIIQNSESPIPMRKYLEFAAESLFCEWAYVIDLDKNTFEVFQGFNKAPLDSSERFASVTSPDSDEGYYQVKFLESFDLDNLPSEEDFIAQLEREED

ST25 4190 cg12_0125

MNAHPEIIEVSRLQALIKDSVNALLPLSSEKDTVITDGGNWIHLRYVGRGTEQIQLELGDQFSIKTKIAYLSEALKRLAEIRNELRGG

ST25 4190 cg12_0126

MGFVGVVSALTAAYAFTPANKEPVTVAAPFKVESIDPENEQAVLQTANEKFTLEVDFDAQYSIDGNGYQAWREVEINEIKDIRVYDEDGEVLAYVDRLDVVEIKDLIESGIRERI

ST25 4190 cg12_0127

MSFGIIQEVIHNNNSIQGFLVQLANSEGTTLVAGINDKYQNSLGKNDLVYWGFVSPSKEHFKFEASGYILALLEPEYDVDKKEWLIAKDLTK

ST25 4190 cg12_0128

MSSLQERMHQAKKHYESTRNKKLKNTEMAEFCKVSKASVGQWFNGPTKELDGSNLTLAAEFLGVNHKWLAGERAPMLLENKSNANVIFNNDEISKIPVLDYVQAGLFHSVGYDGVNPVGETYTTYKSKKEKSVFSLTVQGDSMLPDFKPGDLLTIDTALMPKPGSFVVAQNGDYEATFKKYRVIGYDDFGREIFELVPLNPDYPTLSSVNNNISIIGVMVLHMRKYK

ST25 4190 cg12_0129

MKKSDAIQAFKTKVGVAKAIGISKQAVSLWGDMVPEGSASKLLLVNPNIPHTIKAA

ST25 4190 cg12_0130

MARARNIKPSFFTNDDLGEINPLARLLFIGMWTIADYKGCFEYKPKRLKVQILPYDNCDIEQLVNDLEKSGFISIYSVRGRKYIKAINFAKHQNPHKNEREGGSEIPDIDEADVEEEEKSLKNNEWANIENNREQDGTDRADSLNLIPDSLNLIPSTPEPKIGKTVDEMFTEFWEIYPNKKSGPKAAKEKFKKINFKKHSFELIMTSLEKHIQSLDWIKEGGKFIPHATTWINQERWNADIGSTQQTSGFNSNYGYQSSQQQTISEQAKWDKFLNQNQIWDVTPKKPLLIEGVGHA

ST25 4190 cg12_0131

MREFTFEDALRLITKMRGFYGKKFTDQWAGVDPKDIAESMVECFQGLTAEDFKRGVTKMMKSTFCPSIPEFRSWCEPKASDWLDAHEAWAIAKNSIEYGTGREMTVVWTEQAAKAFEKCADLVATGDKFQLAEAKKIFVSIYERLVTEAKDQGLKPVYNVSLGVDPDQRITAIKQAEVAGFLSTQETQLQLEHKQTKEEQQADAERYKTIAQKAIAELREKLKIQAPVNKMAEEIKEVQPWELKPDTEYWPDPFDQKEEFKQMLEADGLKLPMALRGAA

ST25 4190 cg12_0132

MLGDCLERMKEIETGTVDMILCDLPYGTTCCSWDAVIPFEPLWEQYERVIKENGAIVLFAAQPFTAVLATSNLKLFRYEWIWEKPAATGFFNAQFQPLRAHENILVFYKAKPTFNPMKTFGHERKTAKRKDIGSEHYGKQVNIKSYDSTERYPRSVQLFSSDKQKANFHPTQKPVALCEYLIRTYTNEGETVLDNTMGSGTTGVACVNTGRSFIGIEQEQKYFEIAQERIAQAGTEKDMQPDLFGEAV

ST25 4190 cg12_0133

MIVVKEVHEAFKNNTYHELKNYVLAEMEVSVLNMVELGR

ST25 4190 cg12_0134

MNYKEMMALRCAYNHGLKTTETRAAACLYVKLRRAGLLEHLKAQQETPAPTARKKISERANPNDVNQLVNWMTSKYGRQAALARQLGVSACVVEKVKNTGTCTQETLSRLKTAQQNIIKLEKKNENKRKRA

ST25 4190 cg12_0135

MRVDSTAFTDNPRARARFLETKKKAKEFLRQRRGYKRPDFNRMILDLRNLGWSHEKIAYVLDVSGGSTVSSWSTGSIPEYIHGEQFIMLWQEQTGIDRVPREGEWQTYKYDIGQLDLLETLDVFAAQLDEELQQ

ST25 4190 cg13_0136

MSLDATLWAWKAPVNSATQRLVLVSLADRAGEDFTCYPSAERLHRDTLLNRKTILKVISDLIELGLVLDTGKKIGNGVRVLKLVGVDCRENQPTSTEIGTGTKIGTGTEIGIPTHPKNGTSTHPNFGIQNLPMNLPKNLPREKTEKSKAKNEYSENFEKFWSAYPTCKRKSDKSGTYKTFTKHEGSFAIETLLSILEKQKSDVSWTKQDGEFIPSPSVWLNQKQWENEYWFQVNNPVVAPDFSNAQLQYGDW

ST25 4190 cg13_0138

LQALASSNQSQVAEQLGIDPSTLSRMKNDRKSNGLTELENCLVLLDILGFKTVLKKYRMISEEKLNALFVMSKAWMESKQTIDDLFQDDIEDFGMCFELGYKEKA

ST25 4190 cg13_0139

MTKSEALALLNCTVTQLAAKLGISHNAVSQWDETKIPLAREYQIRDLANGKQPIKRTNATA

ST25 4190 cg13_0140

METLGIRLKNLRKQKKLTQQALADLVGVSKTSVIYWEKDENVPKHESLMALAKVLGSSTEYLLKGKEPKNLTNFNIQDFIIKHGLTTKEEASFDADSIIEPDVVEFDEVNGYIWIDVVEANFSCGTGESIEFHFDVINEKYPFPPSFFQRKMVDPKCLRLIKAKGDSMADYIHDQDLVGIDLSQTEIVDGGIYAVYFAGEGMIKQIFKEADGSLVLHSFNEKYRDRIITEQNGLNFKVMGRQVWRAG

ST25 4190 cg13_0141

MELDNAKLPINQIIARINDAAKNGEALVLTAEEVRILSKDIGDKVFIPVLTNEQVVQLVKEGKLGQRIDNTKK

ST25 4190 cg13_0142

MTNKLFLFSCEHEDISIVKMEQVASHLVGLLRHSNMTRSEIALQLGWKKSRVTKVLSGEENLTIKTISKISRLLGYDFDVIFHNKNYERPKQPWQIDRERKKAHLVEKSHRNKTTFLFDLQTGQQVVEDVLSGNEKEFYISVNSSRYNNGKTINIVAQEVNTLPNTTSSYFNVNQEIKVGYEQR

ST25 4190 cg13_0143

MLGHKVNQNTAVHSATVALPMSGLLSLIDTLNNIFDNPDFRNNYIQDVEKLTQDLKQRFNSSSE

ST25 4190 cg13_0144

MTQKFEIKNRWTKEVLFTCDIPEGMESGMIARHAVEAAIAADANLRGADLYDANLRGANLRGADLRGADLRGANLRGADLRGANLRGADLYDAKLRGADLRGADLRGAKAAPLFVYGLCWDVIISGLGKMRIGCQEHSVADWKSFDDARITRMDSEALEFWNQHKLMLLNMCDSYVHPVEETSNDSE

ST25 4190 cg13_0145

MIQNKYSIKQAFIDGTSGFLLFWIVFFLFVGLLRSCADEQHVNELKAKENLYVRVQVEGVE

ST25 4190 cg13_0146

MNSKALCLQANPNLYCKSIGGGMFQFYQIFEGQYFFANSAKTEKAAWDLALKKLEQDKEPLMDNFQEIINKSVEESRVQIAHTAGHLAVAHQAFANDYLLNVANQTLFMLGTTLTAEEFETEIEGLKKHLIESLRGNNS

ST25 4190 cg13_0147

VLSVDTRTHAVVTDKDGIESPIGLRFKLTEAQVNSLNEQLKYYAEELADEELRGG

ST25 4190 cg13_0148

MEISNTVIISNSDNHPSIGFGHIQDDGVWVVYEGEYDFMNPSKVTHWMPLPEPPKN

ST25 4190 cg13_0149

MNAPVNGTLITTQIANVAETLGLVNVNPQELKETLIQTAFRTETPATDAQMASLLIVAGQYKLNPWTKEIYAFPDKNKGIIPVVGVDGWSRIINGNSNFNGMEFKFSENMVQMEGAKVAAPEWVECIIYRKDRDHPTVVREYLAECYRAPIKSKNGYLIDGPWQSHPSRFLRHKATIQCARLAFGFVGIHDQDEAERIAESGQPIKDVTSEVPEGYQAFEDQHLATLKSEAQYGTERLQAAYVAIPKGNLKKHLWEVHSISLKEIAQFADQALQRQGETYEHSPA

ST25 4190 cg14_0151

MATFQKRNGRVTATVRIKPHPAKSKTFDTLRDAKKWAQETEVRLKNEKLEIFDHIIFKDALIEYRDTVSINKRGYEKERRKINFLLKAMYVDQPLIQVNKDFLTEWREQRLLNVKGATIRREFILLSAFFTWCIEVKRWLSVNPLREIKFPSESPHRERVISDEEIEILLPFLSTEMRYIFLIALQTGMRLSEICNLKWEKIRLSKSYLILDLTKNGRAREVPLSSQAVEIFKSIGPKKQGYVFSITSDDATDEFRDAKLEAGLEGFTFHDSRHTAATKIALKIPLLDLCKMFGWSNPRRAMIYYNPTSSEIAARLSQP

ST25 4190 cg14_0152

MTEVKFVSMPASELAQVIEKACENAVTKVLAAQGDELLNITQLCERIPGLSYHSFKKLAKEHRFKDIKGRYSLTAVKAALQSH

ST25 4190 cg14_0153

MSELYSSQAVIDVLNERERQIQIKGWTSEHDDKYEQNELVRAGAGYANHVVERGWVYSDYGAEAYQDEEVPDFWPWGDCYWKPKSPRQDLVRAAALLIAEIERLDREVKAESMEDL

ST25 4190 cg14_0154

LGDDMTDLNKEREAFEKLSEIAEILNEEKSHFNGDFYDLPFNSCAESFINGAWYAWQEKAKAQAVLDTQQKLTDTYYLEGSDYVVDCPFEYDIEIDKGEVLELQKWQRTESTKVYFANIYKDEDNFEILQFASKAEAENAVAENLKFLEASESGAEE

ST25 4190 cg14_0155

MNDLEINGYKIFTNPDEAVYAAKSKEDVYNYFVENYGSTEECQDESKEQFINNLNEVELDSDCAQRNREWINEDTGMISTSSYYQEYKHVASKDEGTEVIAFLVW

ST25 4190 cg14_0156

MTTLYDIGYDLAEQVERIQDLLAEGASSDSEEVQLLLEGMVAKEGEWKEKSKRVAKFVHQMMLEEKLIATEAQRLSDKAKRIKSTYGYLHDLLLDQMLEFGVSEIEDPVLSIKVKENPWSVVVKNEEEIPAQFKREKTTVEVDKRALLNARESITDIKGIEFIRTKKLAFK

ST25 4190 cg14_0157

MGNYQAFNLNTESAKQADAGGRIETTGKYVGVIKSMEFVTSKQGTQGFEINFESDSKEFTNFTIWTVKADGTALSGVHKINAIMACASVKSLTPTDQKLEKYDFDLKQKVQQTCVVAPEMTNKRIGVLLQRENYLNGSGQQRHQMNFFASFNADSELMAKEILERKTSPELLPKALDRLIAMGDAQRAQQNAPQQSGGYGQYSQTQGNQSSDLDDDLPF

ST25 4190 cg14_0158

MSIATLILGQSGTGKSTSLRNLNPNDVLLIQVVKKPLPFRSAEWKYLSKDGGSIYVTDSPEVIIKRMQQTSKPIIIIDDYQYVMANEYMRRSTETGFNKFTEIGRKTWDVFTEASNLADNKRVYILSHTEEAESGKTKIKTIGKMLDEKITLEGMVTICLQTGVINEQYIFHTKNSGLNTVKSPIGLFESDHIENDLEAVDTAICDYYGIAKTETQTTTETA

ST25 4190 cg14_0159

MEWISVEERLPAFQEETSILCLLKDQQKGFWYPRPYALLIEVGWWIPQKEIFVCDGVEDAKHIISHWMPLPEPPKN

ST25 4190 cg14_0160

LDLSTDIQVEGETVYFDLTYGCNVLNCQIKAETTYDTREVTDQSSGCARDQEYEVLVVDTKTHAVVTDKDGIESPIGLLFQLTSTQVADLNEQLKYYAEELADEEAGVV

ST25 4190 cg14_0161

MEHIDPCFKKAFEEMELLKKDIILPKGMWLLLHGCPVQLKEQTLVRADPTDILFALDMVEKRNSISLVKIDITGLLDNLKELNPEIDF

ST25 4190 cg14_0162

MYVKFVDQGVLAIQQIPTDDFNNFLASQDKDAFLKEQLMINADYRKRIQTRSAIAKSLFQG

ST25 4190 cg14_0163

MNNSKLPINQIIARINDAAKHGEALVLTAEEVKILSKDIGDKVFIPVLTNEQVVQLVKEGKLGQKINNTKD

ST25 4190 cg14_0164

MSLHSRIRQKLEEKKLRAADLARATKKSPVAVKKWLDGTSVPTAENLKVIAKFLGVSDDWLLYGGPVEQESNNLPQLNVLDIEAFKQKYNIPDSEEAVKFVQTPTKPFPIQKRYVPVKAYSKMGMDGYFTDMGYDGNAGDGYVPTHTAGPRAYGIKGTGDSMFPAIRNGWYVVCDPDAEPVPTEFVQVCLKDGRCTIKEFVGINGGVLSLLAVNGGERLSFDMDEVESITAITDIVPPSQHRQEHPYSH

ST25 4190 cg14_0165

LSEKTGYSTVTLWKWENNGIPARTQAVLQVKTKGKLKADLQALTA

ST25 4190 cg14_0166

LQALASSNQSQVAEQLGIDPSTLSRMKNDRKSNGLTELESCLVLLDILGFKTVLKKYRMISEEKLNALFVMSKAWMESKQTIDDLFQDDIEDFGMCFELGYKEKA

ST25 4190 cg14_0167

MSKTTFNFIQWYESKYPEFVNRYGALKRLYDSDLDSFFIEEIDELYKEFKQGGVV

ST25 4190 cg14_0168

MSLYTTGHPVVDKIASLNIEGNVIPANWFNTFKLENGKPDTNAVILLSEIVYWHRPTIVRDEDSGHIVSVKKKFKADLLQRSYQSLADQFGFSRKQVKEALDRLEKFGVIKRHFRSVDVNGQKLSNVLFIELVTHVLFEVTTLLTSKVGPSSLESHDLPPYREDPPHLEGDTYTENTTEITTDSKLSTAELEKILKGKKPCEALVAIGLDLEVAKRFNEYRKTLKKPLTLDAVIKHYHESCNAGISTNDAARIVLSESWIGFASRYNWKPAFETLNGSAQQQTPADMKNADLNYGDW

ST25 4190 cg15_0169

MYEEQQKNLENLKKNAAIFDENRKLLEERDLLERDFTYNYEIGNQKFEKTSNYEYFDCLEDAEQFPEYFEYYFFYNKYIEQITKDEGIALTALLPVRQIYSNINGLKIQGVLPFPSLDWRKTPMGSIHYIDEIRDHPPYNWDGRKVCEDPIIKEMSKVRHTDKDVWLITQDAEDFNYSLRQLIDKLYFVKRPPQNPQACGIYVFDKYISRPRDAADSLRDPKKYVDYFLLVYKKKYQRMYVSASSHTSMKFRIPPKVFFYSLLFIAIFTIAIVGFMKIPIFQSFGSAIKQMTGQEKDAFSQLKAGPQATPNPSETIADKLKREAECGTLTPEQCADLKHPEVRNKQLQQVNDVRMETIAVKYNPNRPYEMDTSKIEYQVTSKPVFAGCMKKNGRYVAYTEQGTILHDVSQSDCRKLIEDNDRPYNYFKQQPQGFGGQPQQVQQQVMPTPVQHYDAEFVAKYQAAKAQGLI

ST25 4190 cg15_0170

MYVGEKFLSTAFKKLLLGAGLGLVSFGLSQSVFSILLNYVNNQFQQLSGLFYLIDLAGVDVAISYILSAISIRITMDAGKLSIRKLQ

ST25 4190 cg15_0171

MRNLKYLIFIIISLFYVPSYAAESSVSYYLANYTPNFRSSSINTTCQSLASDIQSKLGSSSTIYFLDSASITDYSGYNFVCVIGRMHYDERTIHGYYYGYKEIQQTKCPQSGTPIPVYFEPNSPIPLRTCKQNSDGTYCITEYTGDKNKPLVISGNKYQNITHASVSEIPSPSCTPEFSKSSCDPKDPYGGCYQPPDDGCNRMSDGSIYCPPDVPPPPIQSGCSNNATYCDMPPTGCGSDYVPGTFNGQQICVRKSNPPPIDPIDQPPSASEPPPIDPNDPPPTSAPPPIPNESNTILRSILDAINAVNNKLTWLKDEIVNSVNNVSRTLGITNQKLDAVNSSVKETTAAVKQTTTVVNNVKAAVDANATTVKTAVEANTAATNGVKSAVEANTTSTANKLNEVVNAINNKPVGGGGGTAVNLESTNNLLKGIQDWLAVSDDTNPEDGEIKVVKNEIDTNFDGNLVNATGTCPQPMQISFSIVQTYTIQFSYETFCLGASLARPWIIFVGMLTAFFIVTGHYRGGSND

ST25 4190 cg15_0172

MALQNVEVIQQEAHKKTWFQRFRKSAKYSLVVPTMLAVNAYAADAPAGPETASIITYIGLVVIAVGAVGAAWIMVPLAAKGIKALVRAF

ST25 4190 cg16_0174

MTKRTINIYGARQNNLKNISLKIPKHKIVVFTGVSGSGKSSLVFETIGAEAQRQINETQDSFVRNRLEHFGVTDVDKIENLNVPVIINQKPLTGNIRSTVATITDIYANLRLLFSRMGEPFVGYSNVFSFNHPNGMCPQCEGIGIEQTIDIYKILDLDKSLNEEGAIDFPTYQPTAWRWTRYADSGYFDLDKKLKDYSEKEWDLFLYAPQH

ST25 4190 cg16_0175

MVKHLGNSLVDLLYIFDEPSIGLHPKDLDNIIKIIQKIRDKGNSVLLVEHDPDLIRTADHVIDMGPLSGINGGEIIYQGTFEELKNSSGLTGAFFRRPNTYKKEPRMGNEWISIKNAHLFNLKNIDVDIPKNCLTVITGVAGSGKSTLISKVLPQQYPETKVVDQSAITASIRSNLLTYLDLLDPIRQLFAKTNKVSAKLFSFNSEGACPQCKGSGIERVELAFLDDIEMTCDVCHGSGYAPEVLKYLYKDKNIAEILKMTVAEASNFFEDRILQQQFNSLMSLGLDYIAIGQRLNTFSGGERQRLKLTKQINETDNIFVLDEPSTGLHPSDTEKLISLLNNLVEKGNTIIVIEHNLDIISQADWIVDIGPLAGDKGGELVFSGTVSGLLSAEKSLTGYYLKKYLEL

ST25 4190 cg16_0176

MSQFFIRRPVFAWVIAIFIIIFGLLSIPKLPIARFPSVAPPQVNISATYPGATAKTINDSVVTLIERELSGVKNLLYYSATTDTSGTAEITATFKPGTDVEMAQVDVQNKIKAVEARLPQVVRQQGLQVEASSSGFLMLVGINSPNNQYSEVDLSDYLVRNVVEELKRVEGVGKVQSFGAEKAMRIWVDPNKLVSYGLSISDVNNAIRENNVEIAPGRLGDLPAEKGQLITIPLSAQGQLSSLEQFKNISLKSKTNGSVIKLSDVANVEIGSQAYNFAILENGKPATAAAIQLSPGANAVKTAEGVRAKIEELKLNLPEGMEFSIPYDTAPFVKISIEKVIHTLLEAMVLVFIVMYLFLHNVRYTLIPAIVAPIALLGTFTVMLLAGFSINVLTMFGMVLAIGIIVDDAIVVVENVERIMATEGLSPKDATSKAMKEITSPIIGITLVLAAVFLPMAFASGSVGVIYKQFTLTMSVSILFSALLALILTPALCATILKPIDGHHQKKGFFAWFDRSFDKVTKKYELMLLKIIKHTVPMMVIFLVITGITFAGMKYWPTAFMPEEDQGWFMTSFQLPSDATAERTRNVVNQFENNLKDNPDVKSNTTILGWGFSGAGQNVAVAFTTLKDFKERTSSASKMTSDVNSSMANSTEGETMAVLPPAIDELGTFSGFSLRLQDRANLGMPALLAAQDELMAMAAKNKKFYMVWNEGLPQGDNISLKIDREKLSALGVKFSDVSDIISTSMGSMYINDFPNQGRMQQVIVQVEAKSRMQLKDILNLKVMGSSGQLVSLSEVVTPQWNKAPQQYNRYNGRPSLSIAGIPNFDTSSGEAMREMEQLIAKLPKGIGYEWTGISLQEKQSESQMAFLLGLSMLVVFLVLAALYESWAIPLSVMLVVPLGIFGAIIAIMSRGLMNDVFFKIGLITIIGLSAKNAILIVEFAKMLKEEGMSLIEATVAAAKLRLRPILMTSLAFTCGVIPLVIASGASSETQHALGTGVFGGMISATILAIFFVPVFFIFILGAVEKLFSSKKKISS

ST25 4190 cg16_0177

MQKHLLLPLFLSIGLILQGCDSKEVAQAEPPPAKVSVLSIQPQSVNFSENLPARVHAFRTAEIRPQVGGIIEKVLFKQGSEVRAGQALYKINSETFEADVNSNRASLNKAEAEVARLKVQLERYEQLLPSNAVSKQEVSNAQAQYRQALADVAQMKALLARQNLNLQYATVRAPISGRIGQSFVTEGALVGQGDTNTMATIQQIDKVYVDVKQSVSEYERLQAALQSGELSANSDKTVRITNSHGQPYNVTAKMLFEDINVDPETGDVTFRIEVNNTERKLLPGMYVRVNIDRASIPQALLVPAQAIQRNISGEPQVYVINAQGTAEIRPIEIGHQYEQFYIANKGLKVGDKVVVEGIERIKPNQKLALAVWKAPAVANHASSVETKTSIAEGAQP

ST25 4190 cg16_0178

MFDHSFSFDCQDKVILVVEDDYDIGDIIENYLKREGMSVIRAMNGKQAIELHASQPIDLILLDIKLPELNGWEVLNKIRQKAQTPVIMLTALDQDIDKVMALRIGADDFVVKPFNPNEVVARVQAVLRRTQFANKATNKNKLYKNIEIDTDTHSVYIHSENKKILLNLTLTEYKIISFMIDQPHKVFTRGELMNHCMNDSDALERTVDSHVSKLRKKLEEQGIFQMLINVRGVGYRLDNPLAVKDDA

ST25 4190 cg17_0179

LGLIGGQQSGSLDPHLSASGSGITRGFAIYNKLWEWDENMLPRLALAEFAEPNHNASEWTIRLRKGLEFHHGKTITADDVIFSVKRLTDPKLASPFRNLVQWIDRDRIQKLDEYTVRIPFISTIPGFVALPETWVNFGGIVPTDFDPIHNPVGAGPYKVKEFIPGQRSVFIRFNNYFKADKPYADSFEVIDFKDQVSRLNALLAGQIDVANAISPEYIKILEQAKHIQTIRSATNTHNSFDFNTQQAPFNDPRVRQAFRLIANREELVQRGLNGQGRIANDLYSPQDPAFLNLPQRQQNIAEAKNLLTQAGFKDGLSVELVTPVSSAQPALIFAEQAKQANVNIRVKQVDFATFNGPDRSKWQLSSNATNVGTPYLSTAVVNDAPISTTNRVNFKDPEYSELFFKALAEPDLAKRKIHLAQAQKIQHERGGMLIWGFSHTIDAAAKNIGGLAPEHTIFPTWRFEKLWKA

ST25 4190 cg17_0180

MRQPFQLKPLVIALSVLGATLPLYVFAEETPQQAGDSESDVITLSDVVVTGAAKKTTVVPKRKVTSIYGTDSSVLDTPRVVSQVSEQQFREDVIRSADDLVKYAPSITRGGGQNANFAPQIRGQNSEVFQDSQRIYSTRHPTNLNAYEEADIVAGPTGVIFAPTSGSGGYINYLTKKPNFNKAETNISGTVGSIYSGKGAKPNFSVSIDHTAPISKELAFRVSATAQKTDDYYDNVKNNFNAFYGALAWRPNDRLRVDWNISYDDYYDFNVTHGWNRASQQSVDNGLYYKGRATPIIQNGSTFWSPVFESGAANSKVIGWQTRQKNDKNQYVAVGGVQTTPLPNSTADQAGTIKGWVYDPSIPGNELVKLDDNVSGRSEDKNSAKRFSTQLKVIKDLNDHWSIANSTLYQNNKDLGDSVGSFFTDLDHELIDNRLEFLGDYDFEIAGVKINNKSSTGGTYRHEKFTSLAANNSFNINPYDLTNDPSQKNPGDLLGLINNSGSTGGWIGQAGVPQYSQYFGYLNLPRMYPIGKGLYAEKGGFPPNGGGAVYTGSGFWDTLSVFTQQNFTFNDVFGINLGINHSSVRAKLENPLVLTPTDVRSSSDKYSLLSYQLSTFIKPTAKSTLYFTYDKSTALNTGVFGPFLIWGAGNQLNPLAFDSQSELKEVGFKYEPITDQLFLTLSGFEQKRDLSPDTNGNMARFEIKGIESSLRWQLQKNIAIGGNFTYLDAEYSSIIPAGFSPFGFHADNATVWGDNNALNQRKAGRYDAAGIPKYSASAYVDYQHESGFGVNLSGWWTSYWYTNLSQTVKVPNNYNLDLGLYYRQPQWTVGLNILNLTNERNFVNGLAGSNSEFLQPMRPLTVQGQFSYKF

ST25 4190 cg17_0181

MWGVTASVSTLALANDDADAISAEQQAQVVNAKDVKKLGDVIVTAQYRAQNIQKVPTAITAVSGKDLAAKGSTFIGDVLTYTPNAAAENPDGDSRPRWYIRGLGTGDVAASTVFPVGIYADGVYLNAPVAGAGDLFDLERIEVLRGPQGTLYGKNTTAGAVNYISRKPVFTDKPTGYGTIGIGDHNLRTFEGAVNGAISDNVAVRGAFYSEDRDGYAKNLANGENYGDVDKKSFRFQILGKINDDWNALVNLHSQTYNGLGNNGSLSIGKYWGVYERPQGRDTNLDLPESNKIQHDGASLTLTGDLGGGHTFTSITAFDKTTQKSISDGDYTPYDVARSYSDNEWRQYSQEFRVSSDAEKRLSWIAGFHFFNEDLDSTGVSARVNKTLPNGAPAQTAGTPAFRDITYNQTTQSFALFGNSTYKFTDKFKVTGGLRWTSEEKDIDLDLVQITSGDYTKGSWWQKDGYSNAVYNPAPNANGSTSRKKRWNELTYDITPEYEITPDINTYFRFARGFRSGGFNTGLSSSLTQLADVNPEYLNSYELGLKSSLLQGNLTANANIFYYDYKDIQTNLLVATEGQGGGVTSVLANGPKAEVKGAELELDYLATDNLRLRFAGAYLDSEYTDFVDKNPVTNVVNADNTGNSLVRSPKYTIGLGGKYTFNLDSGARVIVGTDAKYRDREFFLVNRQDYSVDPILSQKGYTLWNANVGYISANNKYQVNAYVKNLLDEEYQVHGRPNGPAGQYVLTYGNPRQVGVSLTAKF

ST25 4190 cg17_0182

MGIEFFTRLPLHGETEFLPGDPRNRGDWANIENVENTGAVSNYEVGDDFTYIDYLSQVARAAEINGFAGALMVNAPTGEEPWTVCSLLARETKKLNFVTAFQAYHFSPYNAVQTAATYQRATGNRLVWNIINGGSEVIQRQVGDDLPHDERYARATEFLDVVKGYWNNPSFHYKGKYYSAEGGGLKYPLNKASLPIICTAGSSEAAREFGAKHADYYLMRAEKPEEIAALIADVRTRAKKYGRENIKFGLSIDTIARRTEQEALAEAQRFLDEAAEKQRLHAAAAHAGLRSARVLSFEKEYAEKDGSKNVSDFFIHPNVWSGFGYIGVPPGVALVGSYQQIIERIEEYNSIGIELFFLAGYPHLEESYRLGEHVLPHFKKQRARLQPTVASPEFDIAL

ST25 4190 cg18_0183

MEIDRRVRAKEFMMLMSIGRTKFYRMIKNGEIPQPIKVSEKEVFWHESSVKKVVEKHKDNSDMIAC

ST25 4190 cg18_0184

MNKIVVKKHNGGTIAQNKRARHDYFIEEKFEAGMSLLGWEVKSLRAGRMSLTESYVIFKNGEAFLFGAQIQPLLSASTHIVPEATRTRKLLLSRRELEKLMGAVNQKGYSCVPLACYWKGHLVKLEIALVKGKQLHDKRATEKERDWQRDKARIFHK

ST25 4190 cg18_0185

MSLPEKHLSIVYHSPYGHTAKVASAIASGAEVMGVKVHVMNIEHIDWDVLDASQGIIFGSPTYMGSVTADFKKFMDSTSKRWKNRLWQGKLAAGFANSGGLSGDKLAVLQQLNIFAMQHGMLWSGLPLMTTGHTETDLNRLSSCLGLMTQSDNAPVEITPPQGDLDTAKWFGEYIAGLLYRLN

ST25 4190 cg18_0186

MEFVQGLPQGVFVGQTRVHHPLALPAEQTELTGFNLALHVKDEAQRVQQHRIMLLDEFAQFGVKKMTWMTQTHSTICHTVNEQIPFTALIGDGLITQTKGHALMMMTADCLPVVLGNAEGTEIANLHAGWRGLAGGIIENTVAAMQSPPTWAWLGAAISQPCFEIGAEVKTAFCSKYPELETAFIDGAAPNKFHADLYAIARFILQSLGVEKVLGGDQCSYQQQDEYFSYRREAKTGRMATFVFM

ST25 4190 cg18_0187

MTSAQSSNTNFSETDFNLLEDSEDADNHTSDTTATRLSLQVQLDESYLGQRIDQVAAMVWSEFSREKLKQWMKEGHLLVNGNIVKPKYRCEGNETLTLNVELEAQTNSQPENIPLDIVYEDEDILVINKPVGMVVHPGAGNTSGTLVNALLYHSPKSAELSRAGLVHRIDKDTSGLLVVAKNLEAQFSLSKQLGDKSVYRIYDLVTYGNIIAGGTIDEPIKRHPVDRVKMAILPGGRDAVTHYNVKERFRDFTRVQAQLETGRTHQIRVHFSYIGHGLVGDQVYMNRVRIPAGASELLIETLRGFKRQALHAAKLGLKHPRTGEEMLFEAPWPEDFTQLVNVLRTENAAY

ST25 4190 cg18_0188

MLALSLGVASAFVGCSSNPSKKEVVDTGPQSSEQAYFDKAQKALDRGQYLDATKSLEAIDTYYPTGQYAQQAQLELLYSKFKQKDYEGAIALAERFIRLNPQHPNVDYAYYVRGVSNMEMNYDSLLRYTSLQQSHRDVSYLKVAYQNFVDLIRRFPSSQYSVDAAQRMKFIGQELAESEMNAARFNVKRKAWIAAAERSQWVIEHYPQTPQVPEALATLAYSYDQLGDKATSQQYIEVLKLNYPSLVNKNGTVNMRAARKEGNWINRATLGILGRESKSVTPDTTTSSEAEPKRSLLNRVSFGLIGNSGKEEAEETTNTPVEAPKSERSWTNRLSFGLLDKPEPKAAEGTTIAPATSSNEAPSASPADNEADDAAQ

ST25 4190 cg18_0189

MAIPQHTIDQILDRTDIVDLIGQRVKLKKTGRTYSGCCPFHQEKTPSFHVYRDKQYYHCFGCQANGNAIRFLMDIDNRNFIDVMKELSSNTGVELPKDNTDNKKLSYTRQVTKQSTPPKTVAEPTTQAPIEDQYNTFEPVYFDDPFAQFEQPFSFDEPVQEGNLYDLLENVAQFYEHQLPTSQKAKNYFKQRGLSDQTIQFWRLGYAPEDWQHLEKAFPYDIEGLKQLGLIRSSDSGRDFDLLRDRVIFPIRDPKGRVVGFGGRALNDEIKPKYINSPDSEVFHKNQLLYGLYEGRKLKANDWLMVEGYMDVIALQQYGITGAVATLGTASNTEHLNILFKQNNRITIAFDGDAAGQKAARRTLEIALPLLNDGRELKFFVLPNDHDPDSLIRREGVENFQKLLQQAPLLSDFVFAHLTGQHDISTPEGKSLVMGELRELTELLPKQGSFRYLLTQSFREKLGLGKRFTPQISHDASLSFNIQTKDEDFAIAILMHHPFLYIHFEGLRAYIPQDELLAKILAILNRIFDELPDDQELATYYVLGACSTYCHEIADIMQRTNIQALTQAPEVADKLAKEYALGLQERYLRQKLKSKISLVESRNLRQQLNELTKQISLRLLS

ST25 4190 cg18_0190

MSFFALGVNHQTASVELREQIAFNAERLSNLLAEQRHHESLKDLVVVSTCNRTEVYAMAEDAESLLKWLADANNIDVKQLIHHVYRYENAQAITHLMRVASGLDSLMLGEPQILGQVKSALALSKEAQTVSPELNSVFEYAFYAAKRVRSETAVGSHAVSMGYAVAQLALQVFSKPEKLTVMVVAAGEMNSLVAKHLAEMGVAKMIICNRSRERADQLAQEIAHQVEVEIIDFSDLAENLYRADVVSSCTGSLHQVIAYADVKTALKKRRYQQMLMVDLAVPRDIDPKVESLDGVYLYGVDDLQSVIDENLAQRRQAAVEAEVMVNQLATQLITHQKVKEAGSTIHAYRQHSEEISQRELTHALEALHHGGNPEQVLQQFAHRLTQKLIHPTSMLLREAAKAESPDYFEWLQQHLQDVFDHERKPKR

ST25 4190 cg18_0191

MSSYVYARPVQETYAVHSFDQALQQSMVAEFALAYDDIPTALHNYTVLAIKSNSTTIKQRALDVALEYNDLQAALNIATHWVVQEPKDVPALFYLSHIALKTHEYQLAAETLDKILKIDPTADLEQILASIAPENAQDREVLLTALRSSAEKDNPSILALIANLEAQKWSVRTSLKYH

ST25 4190 cg18_0192

MKANLLIALSDQEGALKWYAKSSRKYKKNLDIRLAEVRYLIQINQPQLALEKLEKIIESNPHAEEALFIAGLTSIDLKQYDKAEQYLVDLRNSAKYQNEAYYYLAINAERKQHYETAKAYYRLVDGSLYIVSRRNLIAIYDKQENLHDALRFLTQERVNYPQHASFLYQAQAEILKKMGNKKAALNLLDEAIKNLPDDPELIYAEVLLLDPYTDRDKLDKTLKQLLQLEPNSPTYLNAYAYTLALQNRRLKEARQYAEQALEYAPEQASILDTLGYIAFLQNDYEAAAEALGKAYELSHNINIGIRYAKALYMQGSLTQFSAVLQQLKQKHANDPQLQQLDALILPTSAKKS

ST25 4190 cg18_0193

MSKLAQLCTAICGSSVLFLTGCQHFTQPKAAVTHQVQDEKHFNLQGKIGVRTPQQTGSAFFTWIQQQDNFDIELSGILGVGKTQIQGKPGEVTLNSAKTGLITAASPEELLERATGWQAPITHLTSWILAKPATLSAQISKDNANRVSQLIEDGWTVNFSYDGEQTLPNKLVLKQALAEDKENRITMVIQNR

ST25 4190 cg18_0194

MIRVPSPAKLNLFLHITGRRENGYHELQTIFQLIDLYDWMTFTPLSEDEIQIEGLGEVQLEQNLIYRAAQILRPHAQNPCGLHIKIEKNIPMGAGLGGGSSNAATTLIVLNQLWQCGLTEEQLAQFGVKLGADVPIFIYGLNAWAEGIGEHLSFIDLDQKQFIVLKPDCFISTQLLFSQKTLTRDSKPTTFCAYQLEPSNFGNNFEPLARELYPEVEEAMQYLDQFGHAKLTGTGACVFAEVTDEMNVDDILKHAPCKAYLVHSLKESPLRHFKVAS

ST25 4190 cg19_0196

MGVMQFSITLEGDTPPQILLGQNLGGAIVTKLEQVKQELVSAAELAKVYNLSVTTIREKLVSINQGTGGKHMYDPERARQILTKKDANKRGRKRAN

ST25 4190 cg19_0197

MKLPKPIKRGKTYRITVTYENKRYSCTRDTEKECEQWAAMKLLELKSGKVQEEKGIKTPYPFKMLCEKYYAEKGVKLRSKHVIRNKLDNLERIVGELASKSIYDFKPSDIARWRNKRVLEVKNGTVLYEFSIFSSIFTYAQKELFLIESNVWQNVIKPEKGKSRSQRITFEDQEKILQQAKWDKNNPPRFVKHYVCWAMLFALETAMRQGEILGMRREDIKDGFVHLPMTKNGEARNVPLSKEAKRLLSILPSNTDILLPVKAETFKRTWIKIRDAADLKHINFHDTRHEAITRMVRERKLPVEVLAKITGHKTIGILINTYYNPNAQDLVEMFNSSES

ST25 4190 cg19_0198

MQLTLVRHGEAAPPVNGNDIKRPLTARGHAQAEQTATFLKDIVKPDIFVVSPLLRAQETLAHIQTYFKDVPVLLCDKIKPDDDAKEAIEWLSQIPYESIVVVCHMNVVGHIAELLTHENFNPFALAEARIYDQAVIANGLSTQKNSFIPTI

ST25 4190 cg19_0199

MAEFKFTDLVEPVAVDKKTALRITVLGGGSFGTAMANLAARNGCDTMIWIRDAETAEEINKTHINKRYLPDFTLESSLRAVSDLEQAVCDRDIILVAIPSHSFRDVLKQIAPYITAQAVVSLTKGVEAKTFSFMSDIIREELPEVPYGVLSGPNLAKEIMAGMPSGTVIASDSELVRYAVQHALHSALFRVFGSDDVHGVELGGALKNIYAVAMGIGAAYKIGENTKSMILTRALAEMSRFAVKQGANPLTFLGLSGVGDLFATCNSPLSRNYQIGYALGSGKTLEQASKELGQTAEGINTIVQVRGKAQELDVYMPITNALYEVIFEGAPPLNIALSLMKNGHRSDVEFVLPHHEV

ST25 4190 cg19_0200

MTDSAIQIIHQNIHQRQSIGQLVEPAPNTDQLELAFQAALTAPDHHRLKPTRFVIVSGDQRAAFGEVLAKALVDLGESDPAQLERVKQHPFRAPLLILALTQLQDHPKVPHFEQILSTGAAVQNLLLSLQAQGFSTMWRSGAVVESNWLKQHLGLQPHDLISGIIYVGTAAKAIAPRADIDSKEFVKVWQA

ST25 4190 cg19_0201

MKKIILASAMSGVVLLLGACATTPKNTLAIQKENNQFEVTGIGKTNLIAKNNAVTAAQKTCSKSTTPVIVDEKTSYHGVLKDVVSEKTGQMVEAAAGVIGTLTGKNASLAKDDDYQTTLTFYCKIN

ST25 4190 cg20_0202

MQALLFGYATTYDVNHVDYAILDQSNGQISHELISKLDGSGIFKRVATLEYTEQIKQVIDNRQALLIITIPNDFESKLNNNQSAPIQVIVDGRNSSTAMVAGSYLNKIIGQFNQQKFNSALPISLETRTWYNPNQESRWSLMPALIAALSMMQTLLLSALSVAREREQGTFDQLLVTPYTPLKIMIGKALPPIFVGLMQSTIILLIILFWFKIPMNGSIGLLYFGLFSFNVAVVGVGLSISALSLNMQQAMLFTFLLIMPLMLLSGLLTPVENMPKALQVVTYANPLRFGINLVQRVYLEGASFAQVKLNFLPMIVLGIVTLPLAAWLFRNRLS

ST25 4190 cg20_0204

MSFAFWSRLKALVRKETKQLLRDKSSLGIGLVLPVILILLFGYGLSFDLNQARVGVVVDQSSPQVNQVLAGLNGSRYLTSLEFHNLPEAEQAIRNGKIDAILHLPSDFANQAQQGNAKVQLLLNGGSTTIATVLEGYVAGALATAPSIQIDRSPILASASAVKIEQRIWFNESGNSTWFLVPGLMVLILTLIGAFLTGLLIARERERGTLEALFVTLVRPFEIVLAKLIPYVVVGMIDIVICIVAAHFIFEVPMRGSLLTILSASFLYLIVSLLLGLTISGFAQSQFQASQIALLASFMPALMLSGFVFDTRNLPLVVQIISQLLPATHFMVLIKTLFMGGDDWKLWFKECGILLGYIVVLICAVNFSLKKRLR

ST25 4190 cg20_0205

MIDSKAVVQAHNLFMTFKAENKNSADISAIVDLNMQIKKGELTALVGPDGAGKTTLLRLIAGLYKASSGSLHVLGFDIEKYPQVVQDRISYMPQRFGLYEDLSVQENLNLYADLHGVPKNVRNQRFKRLLEITDLTQFTQRLAGQLSGGMKQKLGLACTLVRSPELLLLDEPSVGVDPLSRRDLWIIIEQLVQEENLSVIISTAYMDEAEKCAYVYIMHEGKILRQGSPEQLKALVHEQTWQIKPSEQIKTRIVQAQLLGNSGEIIDAVPRGEQVNFISRQKELSTNILPLGLVANRRPPELEDAFMMLLQQNQKQQISISQQTFRSEQNNNSQSDQAVIVVKDLVRTFGDFTAVANTSFTVQRGEIFGLLGPNGAGKTTTFRMLCGLLPASSGYLEVAGKNLRTARAEARAKVGYVSQKFALYSNLTVLENLKFFGGAYGLSGKKLDQQIDKALQQYDLKPQIKSGDLPGGYKQRLSMAAALLHEPEILFLDEPTSGIDPLARRSFWYSIGKLANQGITIIITTHFMEEAEYCDRIAIQDAGKLLALGSPQQVRELASKDKHIVDMNEAFIAIVEQARALKHAG

ST25 4190 cg20_0206

MNKKVIAGILVVIAVVAIGFWVWGYNHKKQLDNDVLTLYGNVDIRQVSLAFEQSGRIQKLLVQEGDKVQAGQVLATLNTNALQIQAKQAQAQLKAQQEAIIKQEVGARPEEITQAKAQLASAQAELDKTNKNLQRLQILVSSTDGRAISQQELDYAKSNQHSAEAAVRERQANLELLIKGARKEDREATRAQYEVTKANLDLIKYNLTQAELRSPVNAVVRARLQEVGDMTTAQKAVYTLALTNPKWIRVYVNEQDLSSIKMGGTAQVIRDAYPNQPINGKIGYISSVAEFTPKTVQTEEIRTTLVYEVRVYVNDPNDQLKMGQPVTVKVPLAFSEKAHD

ST25 4190 cg20_0207

LTKTKIIEAAGPLIAQYGFAKTANKTIAKVANVDLAAINYHFDGRDGLYQAVLMEAHAHYLDEQYLLELVESTYSPEEKLSLLLETLLHKLTEKDVWHGKVFIRELFSPSEHLLSFIELTGMRKFFLIRKLISQVANLDENDPAVLPCILSVMTPCMMLIIAGPNAQAPEPLKNIAQMPLHDLVEHFKKFSLAGLKAISQSNLKN

ST25 4190 cg20_0208

LANRKLKIGIVVGEVSGDTLGVKLMRSFREQGIDAEFEGIGGPQMIAEGFNSYYPMEILSVMGIVEVLKDLKKLFAVRDGLINQWTQHPVDIFIGIDAPDFNLRLSKSIKEKNLPIKTVQYVSPSVWAWRQGRVHGIKQSIDLVLCLFPFEKVFYEQYEVPAAFVGHPLAKQLPLENPIQIAKQELGVDENQKHIALLPGSRKGEVERLLPMLLGAANILHTKYPDIQFLIPAINDARKQQIEQGVEQLAPQLKAKIHILENTDSESKIGRMVMNASDIIALASGTATLEAMLMHRPMVTFYKLHWLTYLIAKFLVKIPYYSLPNIIAGKKVIEELIQADATPENLAAEIEKLMNVETAQIQVMQHLTMHKQLISGNTEDPVQAILQCLNS

ST25 4190 cg21_0209

LRHASHTPNHEIKHYADGHFEIYVGEAFERVVRDQIDFLKRIVPVK

ST25 4190 cg21_0210

MKYDLNKKVVLITGAAGGIGAATAREFYALGANLVLTDMQQEAVDKLASEFEASRVLPLALDVTDAVATKDVVQKTIKHFGHLDIAFANAGISWRDGASTIASCDEAEFDKIVEVDLLGVWRTVRAALPEVTRNKGQILITSSVYCFVNGMANAPYAASKAAVEMLGRCLRTEIAYTGATASVVYPGWTATPIAKVAFGGNATVTKMIEAAFPAWLRKPISPEYMAQAIVKGVQRRQPRIFAPVRWVPFSILRGMFNAASDAMVIRHKKLQGLLQQLESESKPAQK

ST25 4190 cg21_0211

MSKYKLKDKVVVITGSTGGLGLAIAQALQAKGAKLALLDLDLNKVESQAKQLGGQSIAAGWVADVRSLESLETAMAKAAKHFGKIDVVIANAGIATTEALEHMAPETFERTIDINLTGVFRTFRAAIPYVKQTQGYLLAVSSMAAFVHSPLNTHYTSSKAGVWALCDSLRLELKYLNISVGSIHPTFFKTPLMDNIQNDPAGKAVWKGNSGIWKYISIEEVVSGVVESIERRKDMTVVPKINTPIAKAPALFRNVIEFLGFNRKQLKQTMLLAEKNQNSF

ST25 4190 cg21_0212

MKDKNFESRRLSKFPHYFGIAEIGDVISINNDKELLAEIINFRPFIQAVDFQINNEYLEKIPKNKEKNYWRDGVRKIDDDTYNAIINLANLGPISKTKTLLSNEDSQSFESGVEGKPSLQITTKYERDQKLRKQAILIHGYTCVACGFNFEEFYGDYAKGFIHIHHIEPLFSVGEKVVNPETDLVPLCANCHAVIHRKRDKTLTVDDLQKMIKRNVV

ST25 4190 cg22_0213

MNKPTPKTYRTTNWSSYNRALINRGNISIWFDPNTQWYAQPQNKQGRNQTYSDTAIQCCLMIKSLFRLSLRMVTGFVQSLIKLCGLNWTAPDYSTLCRRQKHIDIAISYQKSSDGLHLLVDSTGLKFLGEGEWKRKKHGPEYRRQWRKLHIGIDAETLQIRAVQLTTNNVSDSQVLGDLLAQIPLDERIDSVYTDGAYDTKHCRQVILDRDAHALIPPRKNAKPWKDQKLRSLERNELLKTVKRLGRTLWKKWSGYHRRSLVETKMHCIKLLGDKLTARSFSSQVNEIHARIAVLNKFTELGRPHTQVVT

ST25 4190 cg23_0215

MLTKTVKSFGLAMGLLACSLPLYAKNNVVVVATGGTIAGAGASSANSATYTAAKVPVDALINAVPQIQDLANVSGIQALQVASESITDKELLQIARQVNELVKKPTVNGVVITHGTDTLEETAFFLNLVVHTDKPIVLVGSMRPSTALSADGPLNLYSAVALAASDDAKNKGVMVLMNDSIFAARDVTKGINIHTNAFVSQWGALGTLVEGKPYWFRQSVKRHTNASEFNIENIKGDALPTVQIVYGSDSMLPDAYEAYAKAGAKAIIHAGTGNGSVAKYIVPTLQNLHDKNGIQIIRSSRVPQGFVLRDAEQPDSKYGWVAAHDLNPQKARLLAALALTKTNDAKEIQRMFWQY

ST25 4190 cg24_0216

MINQVDFFKCLSDQTRLNILKLVLNKQNICVCELTEQLELSQPKISRHLALLRTHGVLLDERKGQWVYYSLNPDLPVWALDILKVIENDESGTKVKQQDQSFITTTCCD

ST25 4190 cg24_0217

LALIRHAGIEPIVIEYLQTPPSKDELIQLIKDSNLSVREAIRKNVEPYKDLELEQDHWTDEQLIDFMVQYPILINRPFVVTPKGTRLCRPSEMVLDILDSQNLGYFAKEDGEVIIDEQGRRLK

ST25 4190 cg24_0218

MYLLSLAVGLLVGVLYYVLNVKSPAPPLVALVGLLGMLIGEQLLPFIKSYF

ST25 4190 cg24_0219

MTEINLILKNGKITTLDPQNPEVQAIAIADGKVVRTGTTDEVMKLATPTSKVVDLNGRRVIPGLNDSHLHIIRGGLNYNMELRWEGVPSVADALRLLKEQADNTPAPQWVRVVGGWTEFQFAEKRLPTLEEINKAAPDTPVFVLHLYASALLNRAALDVLGFNKDTPDPPGGKIVRDEKGEPTGLLLATPSAMILYSTLGKAPKLPVEDQVNSTRHFMRELNRLGITSAIDAGGGGQNYPEDYDVIKQLHDQNQMTVRIAYNLFAQKAGQELDDYRRWTEMTFPGDGDELFRMNGAGENLTWSAGDFEDFYEPRPDLPEKMEGELEAIVEHLAEKKWPFRIHATYDESINRLLNVFERVNSKQPFATRFIIDHAETVSERNIERIGALGGGIAIQHRMAYQGEIFVKRYGAEAAQATPPVKKMLELGVPVGAGTDATRVASYNPWVCLYWLTTGKTVGGLPLYDEKNLLDRQTALKLWTKGSAWFSGEKDIKGSLTAGELADLVVLSDDYFKVEAEDIQWIESVLTVLGGKVVYAGAEFKQDDPPLPPASPTWSPVKRFGGQWRLSENRNAPSNQSLQSQSALCACASSCGMHGHSHAWMLDVPVNDKDKKSFWGALGCSCFAF

ST25 4190 cg25_0221

MNTKYRKPLAGTQLEYYDVRQAVEDIQPGAYEKLPYTSKVLAEQLVRRADAENLTAYLTQLIERRQDLDFPWYPARVVCHDILGQTALVDLAGLRDAIADKGGDPSKVNPVVPTQLIVDHSLAVEYGGADPDAFAKNRAVEDRRNEDRFHFIEWTKTAFKNVDVIPAGNGIMHQINLEKMSPVIQARDGVAFPDTCVGTDSHTPHTDALGVISVGVGGLEAENVMLGRASWMRLPDIIGVEFVGQRQAGITATDIVLALTEFLRKERVVGAYLEFFGEGADSMSVGDRATISNMTPEYGATAAMFYIDQNTIDYLRLTGREDTQVALVEQYAKEIGLWASDMTKAEYPRVLRFDLSAVTRNIAGPSNPHARVSTSDLKEKGIAGVVENRSDGLMPDGAIIIAAITSCTNTSNPRNTVAAGLLARKANELGLVRKPWVKSSFAPGSKAAALYLEEAGVLKDLEQLGFGIVAYACTTCNGMSGALDPVIQQEIIDRDLYATAVLSGNRNFDGRIHPYAKQAFLASPPLVVAYAIAGTIRFDIEKDALGYDKEGNPIYLKDIWPSDAEIDALVKEAVKPEQFRKVYIPMFDLGEVEQAKSPLYDWRPQSTYIRRPPYWEGALAAPRTLANMRPLAILGDNITTDHLSPSNAILMDSAAGEYLHKMGVPEEDFNSYATHRGDHLTAQRATFANPKLYNEMVRRSDGTIKQGSKARVEPEGEVMRMWEAIETYMNRKQPLIIIAGADYGQGSSRDWAAKGVRLAGVEAIVAEGFERIHRTNLVGMGVLPLEFKPGVNRKTLKLDGTELYSVIGNIAPRSTLTLVIERATADGKEEIVEVPVTCRLDTEEEVSVYEAGGVLQRFAQDFLEGQVA

ST25 4190 cg25_0222

MSSNETTTGFKPKKSVALSGQVAGNTALCTVGRSGNDLHYRGYDILDLAAGSEFEEVAHLLVHGKLPNKAELKAYKAKLKALRGLPAALKTALEQLPPSAHPMDVMRTGVSVLGCLTPEHEDHNEAGAKDIADKLMASLGSMLLYWYHFSNNGRRIEVETDDDSIAAHFLHLLHGEKPSEEWIQAMHTSLILYAEHEFNASTFTSRVVAGTGSDMYSAITGGIGALRGPKHGGANEVAFVIQQRYDNPDEAEADIRKRVENKEVVIGFGHPVYTVSDPRNEVIKKVAHDLAQAQENTKMYLIAERLEAVMKEVKNMFPNLDWFSAVSYHLMGVPTAMFTPLFVIARTAGWSAHVIEQRQDGKIIRPSANYTGPENLEFKPLAERG

ST25 4190 cg25_0223

MAKQSAGQLFRDAVAQEKPLQVVGTINANHALLAKRAGYKAIYLSGGGVAAGSLGLPDLGISNLDDVLTDVRRITDVCDLPLLVDADTGFGASAFNIARTTKSLIKFGAAAMHIEDQVGAKRCGHRPNKAIVTQEEMVDRIKAAVDARGDDSFVIMARTDALAVEGLQAAIDRAGAYIEAGADMLFPEAITELDMYKQFAQKTGVPILANITEFGSTPLFTTEELASADVSLALYPLSAFRAMNKAAETVYETLRKEGTQKNVVDIMQTRKELYERINYYAFEDYLDSAFAKKK

ST25 4190 cg25_0224

MQDLTFAPEPLQSQGRTLTENVFKQIQTAIVLGQIPAGSKISEPELARTYGISRGPLREAIHRLEGQRLVERTAHVGARVVSLSLQQFKELYQIRASLEGLACKLAAQHIDKKQILALRDVLRMHAEDENFKAGKGYYLQEGQDDFHYCIIKSSGNKTLEKMLCDELYHLIRMYRIQFSNTPDRPSKAWDEHIRILDAIAEGDGELAELLMHRHINASYKIIEQTLLQAQGEHNNG

ST25 4190 cg25_0225

MEQFNADTRSEKVNLSIGLYYNEDSIVPQLETIIEAQKRIEPKNGKTKLYLPMEGFKPYREAIQALLFGANSPAVKAGRAVTIQTLGGSGALKVGADFLKTYFPNSDVWVSQPTWDNHVAIFNGAGIKTHFYPYFDAETRGVDFDGMLSTLKTLPEQSIVLLHPCCHNPTGADLNPAQWDQVIAVLKDRNLIPFLDIAYQGFGDGMEEDAYAIRALDQAGLNFIVSNSFSKIFSLYGERVGGLTFVCDDAEAAQCTFGQLKATVRRIYSSPPTTGAWLVDEVLNDAELNQQWQGEVKEMRERIIKMRSILKDELTKALPDRDFSYLVNQKGMFSYTGLTAEQVDILREEYAIYLVRSGRICVAGLNMNNVYTVAIAMAEVLAKSVEAA

ST25 4190 cg26_0226

MTARIQKGKLAIAKELYDFIENEALPGSGLDSETYWKNFEQVVVDLSPKNKALLAKRDELQAKIDEWHRNNKFELGAYKAFLTEIGYLLPEVEDFQITTENVDEEIALLAGPQLVVPVRNARYCLNAANARWGSLYDALYGFDVISEEGGAEKGKGYNPVRGAKVIEFAKNFLNEIFPLAQGSHADATKYAIEQNKLVVNLKDGTKTGLAHEAQFVGFNGEEANPSEVVLLSNGLHVIIEIDANSPIGQTDLAGVKDLTLEAAVTTIQDLEDSVAAVDAEEKVEGYRNWLGLMKGTLQESIEKNGKTIVRALNKDREIKNLIGGTTKLHGRSLMLLRNVGHLMTNPAILVDGEEIFEGIMDALVTPLLSIADIRSENENKNSRKGSMYIVKPKMHGPEEVAFAVELFERAEQALGLPAKSLKIGIMDEERRTSVNLKNCIAAAKDRTIFINTGFMDRTGDEIHTSMEAAPVVRKEAVKTQKWIAAYENRNVAIGLKCGLQGKAQIGKGMWPKPDSMKDMLATKAAHPNAGASCAWVPSPTGAVLHAMHYHQVNVKARQDQLKAEEMLSLDDLLTPPFATDTNWSAEEINNELENNCQGILGYVVRWVDLGVGCSKVPDINNVGLMEDRATLRISSQHVANWLRHGIVTREQVEEVLKRMAKIVDEQNANDPLYKPMAANFETSIAFQAASDLIFKGCEQPSGYTEPLLHAARLKLKGYTGD

ST25 4190 cg26_0227

MGMNVGSNNDDDVMLEVNMTPLIDVMLVLIIMFIITIPAPNNAININLPNGTPPPTNEKPLEIIDVRIDAAGKVFWNNQQVSDRKALEDFISRCCRQKRSRPNQT

ST25 4190 cg26_0228

MGMSVGSEDDDEMIGTINTTPLVDVMLVLLIIFLITIPVVTHTVPVKLPEEKNTPYATTPENIQLSVNKKGDIFWNESYVPNKEILLAKLQAVAQKRPQPEVHIRGDQLTHFEAIDQVISTTKQAGIGKIAFVTTPPASP

ST25 4190 cg26_0229

MMKKSTQPLVHFASASLIAACSLLPLSSVFAEETSNAPVATATTEATSNTTTPTPPPKPATSETVKNPYGLEALWREGDLVAKSTLFILVLMSIGTWYIIISKLLQQGKVKRQGKEAEKHFWEATSLDNAAEGLEQSSAYRFIAEKGINSTKSHGGSLLERIDFNTWVSISIQRAIEKVQNHLGGGLAFLATVGSTAPFVGLFGTVWGIYHALTAIGISGQASIDKVAGPVGEALIMTAIGLAVAVPAVLGYNWLTRRNKAVMENVRSFGSDLHAVLLSGEINTNNSINRSIK

ST25 4190 cg26_0230

MVEKVAKVPEPPKEVEKVATPVQKTTPVAQPTKVATPAPAAPSTPSPSPVAAPAPVAAAAPAPKPAGVTRGVSEGSAGCEKPEYPREALMNEEQGTVRIRVLVDTSGKVIDAKVKKSSGSKILDKAATKAYSLCTFKPAMKDGVPQQDWYEIEYPFVIE

ST25 4190 cg26_0231

MGMTFTDIENKSAKRLIGIAAVIFLHLLVAYILMSGLANNIQKPAEKPVELQIIQDIKPPPPPKPEEPKPKENHLSHQKW

ST25 4190 cg26_0232

MAQAAETEQSSTDEKPTKVVKVAVTGSSIKGVAAQSASPITIIKGEDLANAGVTTVEEALTRVSSNQAGFSTDQNVGASNTDGSTANLRGLGSDKTLVLLNGRRLAANPFGTSTVNLNIIPLAMIDRIEVLRDGASAVYGADAVAGVINFITKKTYQGVGISAGLLQPEHKGGDKQDISIFGGYGDLDEDGFNIFGVVDYRRTNGIMAKDRKISERGGVLPELGLDGRSANAFTSNFYDPISGVSGNPYAQNGQCLGPAESAEEGFCYANTQALIGIKPDVENVSVLGRGTFKVNDNLNAIGEYIYSRSEVITSVAPDPFSRSNLSTRVTLPSDSPYYPGNGIVPSVKGLSGAPLELYLRSHAGNRIAKSINESHRLFAGLEGDVWGWDLNTGVTYAKSEASDNFVTGQLNKTKLQDALNNGTLNPFGESSDPNIWNNLSVKGKYNEATLESTTADFSISRPIFTLPAGEVGFALGGSFTNQDWKQHINADLVRQAPSSGTDPSKPDNSGDRDITSAFAELQIPITKTIEAQLAARYDDYSDFGDTFNPKFAIRWEPLKQLMFRTSYSTGFRAPSLYEINAAQSKTYTGAKYNDPFYCPGGEVVEGKNKNDFCNTQFMKLQGGSKDLQPEESKSFTAGFVFEPIKNLVLTVDYFNIKIDDLIAQVGEATIFGEPEKYSALFVRNADGTIDYIKTNLFNSGGIKTEGFDISLNYLTPMTSTGRFGFGIDGTYVSKLDYRDSKGEPWTGQVGLYEDPAVVRWKHTANINWAYENWKLVFEQQFVRGYDDQNQIGEEQYDHHKVSDYTLYNISGTYKGFKNLELTAGIKNIFDEDPSASNVLDNFQYGYDPRYGDPTGRTYYLRGTYKF

ST25 4190 cg26_0233

MYIFYTNNFETFRQQQKFFQTENLFLYQKS

ST25 4190 cg26_0234

VGMNLKLKFFKLAILSLAITSVPCAYAITPAQPNKILHVAYEAPDDGFDMVKTTNFYSANIAEAIFEPLLKYDYLARPLQLVPNTVESLPKVEQDGKVYIFKIKPGIYFTNDPAFKGKRRELVAEDYVYTIKRILDPKNRAPSVSFIDGKLQGADAVVAQAKKTGKFDYATPIAGVKALDRYTLQFTLTRQDYNFPYILAYIAFGGVAKEVVDYYGDRIGMHPVGTGPYMLSKYVPRSKVELVANPDYRGFVWNFKSTGTPWDNQLVKEMFGKKMPQVGKVVVSIIEEEQSRWLAFQSGQLDFDKLTANAVPQALDGNQLKASFKKRGIKHYPNKDPEITYTMMNMRDPVIGGFSPEKIALRRAITLSYNQKESIQQAYKSQAVRSQMFIPEGVNGYNPKYKSSVGYNPLLANKLLDHYGYKKGADGYRTLPNGKPLILKINTENSSASVIHSELWKKNLDAIGIRADFKVSNFADNLKAATQCKYMIWSGAWIADYPEGDNFAQLLYGPNAGQGNHACYQSKTYDALYTQAIHLPPQQRLPYYEKLNRQIEADNPWIIHVTRIRNWLIQPQVQGFKAHPMMNTNWQYLDITPIKK

ST25 4190 cg26_0235

MTENLKQKLKHCSVGLFFAGMALTGSISIFAKSPADPNKVLRYVFPTAETGFDPAYVHDLYSAHVLTSIFETLYTYDYLARPAKLIPHVATAMPEVSADGLTYTIHIKKGIYFTADPAFKGKPRELTAYDYAYSFKRLLDPNLRSPNSWLLEDKIEGMNALVKAANKSGKFNYDQNVSGLQTPDKYTLVIRLVKPDYNFPLLLAHDPTGAVAREVIEKYKDKAGFVMGHPVGTGPYMLSKWIPASRIVLKANPEYRGFTWNFNASSPGDEAIVKRLKGKQMPQIGTIDIQVMEENQSRWLAFQRGEVDIIQLEGQLVSKAIKDGKLRPELAKEGVQLSRIVDPEISYIYWNLKDPVVGGMSKEKIALRRAIAMSRSIDQEIKLVRNSDAERLHFPVPPGVVGYDPQYRSSTPYSVKAANLLLDRYHYKKDASGWRTQPNGKPLVVEYKARNDSIGQQSAELWKKNFDSLHIRMVYKPMLFSDLLRSQKQCEGMFGSSAWIADYPDGDNFMQNFYGPNTHMTNWSCGSIPEFDELYRQSQQVKPGPERDVLYRKMTRLLEVYMPVQMSYARYRNMLAQPRIIGYKKHPILHAEWMYFDIDTNTKSNH

ST25 4190 cg26_0236

MKKIGDTMKNNTLKMTTLCMLTIGISQFASAEATRATLPLLKAEQLPAWCDSNLKKIQQEISSFEKTPVKNDAAAAPILAKWDKIFAHLEDFSGPIGLYSNVDPDAKLRKAAEDCEIKINQFHTDIFQNSKLYNVIKNTKATDPIDKKYRQDILDQFEDTGVQLEPAKRARMKVILDELTKLEQEYNRNIRDNPEKLEFTPEEMKGLPESYISGLKKNAKGNYLLGFEYPEYRPFMELADNDDARKRYQIAFTRRGTEQNLKLLKQAIDLRYELAQLFGKASYADWALKDRMAKTPEAVNKFLAEVQKTVAPLERKEVEELRAFKAQTLKTPLDKTEITRWSEAYWSEKLRKSKYQIDQEKLRDYFPTLAAQKWLFAISSDLYGIDFKPVKVKAWQDEVEYYDVVDKKTGKLLGGLYMDKFPREGKYGHAAVWGVYGGSTLTNRFPISVLVTNFNRKGLNSDELETFVHEFGHALHGILSNTRYTSQSGTSVERDFVEAPSQMYEEWARRKETLSKVADYCDPACPRVDDELIARLKAVHNYGRGLRYARQTLYAQYDMALHTADALKVKPLETWQKMEAATALGYVPTTEFPGQFGHLMGGYQAGYYGYMWSEVLALDMLSAYGDNLNNPQVGQRYRQTILSQGSQKPAAELVKDFLGREPDNKAFFNEITGQRVK

ST25 4190 cg26_0237

MLAYVIRRLWQMIPTMLGVVLLIFILFNWVGGDPAYILAGKMSNPEQIENIRKQLGVDQPYYVQLWIFIKQILTFDYGASWSTGESVSQIILTRLGPSLTLLIPLTILQTVISIILALAVAAVRGSLTDRMVMMLCTIGMSISILVYIMFFNMFWLIN

ST25 4190 cg26_0238

MLVVSIAPTLRLYRSFVLDEVNQDYVRTARAKGVGESRILSVHVLRNASIPIITDVMSNLPALLIGAFLIERFFGIPGIGREVIIAVERSDFPVIKAITVYIAAATMIFNLIADLVYKVVDPRVQLK

ST25 4190 cg26_0239

MLSVLSKRKLETQTAPASAGLWRLAMRRLRADKIAMASLIVVLFYLIVLILSLTGVIASDWNKEVAVSYAPPTFIGADKTTEALKSTAAVDEELPENPVDPLKDVIHQLKAEIKQEKSSGSAIDYYGVVDPLADDMKAIDQQLGGHLLDQQSELKSTLIFGADKWGQDVLKKTIKGAETSIIVGLVSALLAVTLGTLLGAISGYFGGWVDDILNWFYNIFTSIPYLLLVLAIAAVLQQKGILSIVLILGLTGWTGVYRLIRAEYMKHTAREYVLAAKAIGVGHFRRMFIHIFPNVSHIALVQMSILVVSFIKSEVILSFLGFGVPVGVVSWGSMLNEAQSELLLGKWWQLVAASVAMAVLVTAFSMFTDALRDALDPKLK

ST25 4190 cg27_0241

MGYQKIVVPADGDKITVKADLSLNVPNHPIIPFIEGDGIGVDITPAMKKVVDAAILKAYGGKRSIEWMEVYCGEKANKIYGTYMPEETFEALREFVVSIKGPLTTPVGGGIRSLNVALRQELDLYVCVRPVRWFQGVPSPVQHPELTDMVIFRENSEDIYAGIEWKADSEEAKKVIKFLQEEMGVTKIRFPEGCGIGIKPVSKEGTQRLVRKAIQFAIDNDKPSVTLVHKGNIMKYTEGAFKEWGYELALDRFGGELIDGGPWVKIKNPKNGKDIIIKDVIADAFLQQILMRPADYSVIATLNLNGDYISDALAAEVGGIGIAPGANIGGAIAVYEATHGTAPKYAGQDKVNPGSIILSAEMMLRDMGWTEAADLIIKGISGAIAAKTVTYDFERLMPGATLLRCSEFGDAITQHMED

ST25 4190 cg27_0242

MKIVILNKPYDVLSQFRKDEAHMTVSDFVDDPTLRIAGRLDMDSEGLVFLTDHGGLNQFITNPANKKYKTYLVQVDGDVTEEALEQLRKGVELNDGMTLPAKAIKVSEPEWLWDRDPPVRYRASIPTSWIEISICEGRNRQVRRMTSAVGFPTLRLIRTKIGTIDLVQLGLQPGETKEIEPLLYPDFKDVPAEQPYRSRSYVKKPGGTGGKPMVRKNKDGSAKKSGTKRIWQMDESEKPRRKTNGTTRPNTKAPRGRSRSNSR

ST25 4190 cg27_0243

MAGEKSTIIYTLTDEAPLLATYSLLPIIETFTKPAGIEIVKSDISVAARVLAEFADYLSEEQKVSDNLAELGRLTQDPDTNIIKLPNISASVAQLTACIKELQSKGYAIPDYPENPATEEEKTIKARYGKCLGSAVNPVLREGNSDRRAPAAVKNYAKKHPHSMSEWKQWSQTHVSHMEEGDFYHGEKSMTLDRPRNVKMELITNSGKSIVLKPKVALQEGEIIDSMFMSKKALCDFYEKQLDDCREAGILFSLHVKATMMKVSHPIVFGHCVKIYYKEAFEKHGKLFDELGINVNNGMAGLYEKIETLPTSLREEIIEDLHACQEHRPALAMVDSAKGITNFHSPNDIIVDASMPAMIRAGGKMWGADGKQYDAKAVMPESTFARIYQEMINFCKWHGNFDPKTMGTVPNVGLMAQKAEEYGSHDKTFEIPEAGIANITDLDTGEVLLTQNVEEGDIWRMCQVKDAPIRDWVKLAVTRARNSGMPAIFWLDPYRPHENELIKKVQKYLKDHDTTGLDIQIMSQVRAMRYTLERVVRGLDTISVTGNILRDYLTDLFPIMELGTSAKMLSIVPLMAGGGMYETGAGGSAPKHVQQLVEENHLRWDSLGEFLALAVSLEEMGIKENNARAKLLAKTLDEATGKLLDNDKSPSRRTGELDNRGSHFYLSLYWAEALAAQNEDAELKAKFAPLAKALAENEQKIVAELAQVQGKPADIGGYYAVDPAKVSAVMRPSATFNAALSTVQA

ST25 4190 cg27_0244

MLHYQIEFDDYKQHLVHVTIRFLANPNQELWLPTWIPGSYLIREFSKHIEAVKAYDEAGRMLNIKKTEKNRWRLFNTDHELITVEYDVYAYDLSVRGAYVDQTRLYINPACVCLGLEGQEQSACELEIFLPDELKHFQLATGLASKSLVKGRFTLKADHYDQLIDSPFELADQTRFSFETHGIEHEFVISGTHATNVDRLKTDIEKICAAEIDLFGSAPFKNYTFMTMATGNSYGGLEHCNSTSLITPRDDLPKSNEPTEPSKDYQRFLGLCSHEYFHSWLVKFIRPENFANYNLHQEGYTSLLWIFEGFTSYYDDLILLRSGVISQKSYLDLLKSQIDRYLQNPGRFVQTVAESSFDAWIKFYRQDENSNNAGTSYYNKGALVALCLDLGLRLRGSSLDALMRRLYENTQNGVQVNERTIFDLCEELTGDSWVEQINHLINTTEELPLDQLFPEFGLSYTLKNDKTLAYGLKLADKPEGVLVQNAHREGAGAKAGISANDVIIAIDGLKATTKLIEKYAKQKGNYSVLAFRRDELLSFEVTSDSINLTEVELIIEDQSKADKWLKA

ST25 4190 cg27_0245

VKFFLSLFTLFSIFCTTLTNAALLNIAPESVEAAAWTIVDTQSGQIIAEHNSHVQRAPASLTKMMVAYIALKEIKAGKLKLNEVITATPVVSVVQWDESQMYLKAGEQISVDQLLAGLIVMSANDAAVTLAEKISGDVPHFVQRMNQEAQALGMKDTHFSNPAGITMPDHYTTAHDLSLLSQAVIHQTPEYLHYSKMPSFSYNQRFHHATNLALKYDPSVDGLKTGYTKAAGYNLALTASRPSFSPNLPQRRLLVIVLGTPSAVKRAEIADKLMNLAYAYTRDEVVIPEQKLIAELPVIKSTLKMFKVETKQPTIVTTSLYAEPTPIDLNTFDYATQRIQVLDSNQQPKVIAPLETTQTRVNIQLNEQKLTAPLMKAMNLATVSIYQNNQLIRSLQIENDVHIEEANIFQRIMMWFSSLFSIFSSSEHSAAKLYPIDSH

ST25 4190 cg27_0246

LVDQNLTHIWKPLLHEIAAGSLNPHEEQTNYFAHAKKHHYEFVLGTLTAVNKDKKIIDLIPPQVSKEQNTHIQQLSYDTLVLALGSVSNDFNTPGVRENCHFLDSRKQADIFQQDLLHLYIEAQNQPDQRTLNIGIIGAGATGVELAAELIETTKNFYRYGLKKIHPNQVKITLIEASERILPALSEKTAEHSAKQLQKMGVEILTKHRVEKIDEHCIYFSNGNQLNSDVTVWAAGVKAPKLLESLSDFERDNINRLMVYATLQTYSDPNVFAFGDCAHCQLDARHPPLGPRAQVASQQASFLVDAMAARLNGRSQPMFTFNDKGSLVSLSRNKAVGELLGDVSVQGYIAKTMYVSLYRLHQATIHGYTQAGLLTMKDLLTRRVRPKIKLY

ST25 4190 cg27_0247

LIEWIKNMTAIANNHVVSFHYKLTNAEGETLDQSQGEPLAYLHGAGNIIPGLENALTGKTVGDKFTVNVPAAEGYGEYNPDLVQEVPAQMFQGVDNIQPGMQFQAQTDDGVQIVTVKAVEGDNVIVDANFPLAGQDLTFEVEIVEIREASQEELDHGHVHGAGGHHH

ST25 4190 cg27_0248

MSNAYQLIWFRQDLRIHDHAALWHASQQGPSLGLVILSPEQWKQHADAPIKISFYLRQLRTLQKELAAIHIPLVIQVIPYWKEIANFISDFSKKYNIENVYANIEIGVNELKRDKTVQDYLNRHGKELFLFHDRTIFPLRSIRNQSQQPYQVFSAFKKACYTKLDTSGLPQCYPVPNKQNEVPLDFLQGENIDLDEIEKLFCSSLSKEQQDLWPVGEQYALEQLDQFIEESVCHYKLERDFPHIQGTSKLSPYLNIGILSIRQCLQALFRNQHGNFHLVNEGQQTWLDELLWREFYQHILFDFPHVSKHIPFKKDSQKIKWNHNPEHLTAWQTGQTGIPIVDAGMRQLLQTGWMHNRVRMITAMFLCKNLLIDWRIGEQWFMQHLIDGDLAANNGGWQWCASTGTDAVPYFRIFNPIAQSKKFDPEGEYIRQWVKELAHLDNKTIHEPYSSKTDLGLNYPKPIVDLKETRLKAIETFKNI

ST25 4190 cg27_0249

MQQKHPGRTLILWIIAAHLFMGMVAYEWLPVSFRGSWIFILLLLISGAFLEVGLSLIVGLVAFIGVAVYFLLDLGAQTHIERQLLLLFIIPLIPLFLSAVRHNIEINVQKYRDIQSYDRNYRRDIFPLSALKYFQRSFFKLLDHHNVQDYEVFEIRILNRSLIKEMLGDDVWKTTQNEMIKILSQHEPNEIVFQFADDDLENIYSIMIRQQKTEEDPVFLKKLKEITTLRLEEEYKIVPLPPEEMS

ST25 4190 cg27_0250

MSNLLNICGIVIASSQYPDATLQQFYRQYYHCEIKAEQIKAEVQSPSDLSMFFPYQDTWWPVFTIDQISSESFQKFIHNGIRPGIILPDEVFSFPHYFLLKEAVSQGAIPIVLFKTEQPQYFAAKATFSTAIGLRPMAAFVSTGWDENLISQPAGSYIIQLNSANLPLPSREVRQGQHLFYSAKGFNGHVSGYEIIINPPADLPLSNIRYPQLGISWNFNNIDYESTPEHISTNLIGYIFIVLSIVVVPLDLILTTTYPDLLGTFGSYISWISLVVGAILLLLLISSIIRRVRKNGSN

ST25 4190 cg27_0251

MAPIDILFLFGFLGIWIPQAFWAWLSYQAWKYSKTAEKELQNLPIPERWPVLSVLIPAYNEGVVIEDTLHAIAQQDYPAESYEVLLINDGSKDNTLEIAENLAKIYPCIKIVNVPKGMGGKGKSRTLNNGLPHAKGELIVVYDADSTPEPDCVRLLAQTLLADKKLVAVNGKVRTRNWQDSILTRFIAIEFIFFQWIFQGGRWQRFELSTLMGTNYVIWRDALETLGGFDEKSLVDDTEMSFRIFIGQKRIKWVPYAIGWQQDPPSLSVFVKQRSRWTQGNFYVTRKYLPVALRTPFPIGIEILNNIMCYILFVPALFWSHITLTLGLLDIAGISVPGPFTLLWGLSFCLYVAQMWFTLSLEKVKPELYFYSVLSYVSYSQIFLFIVFKAAFDMLKNKIQGNSLQWYKTERSKEKK

ST25 4190 cg28_0253

MNKHLLAKITLLGAAQLFTFHTAFADIPLTPAQFAKAKTENFDKKVILSNLNKPHALLWGPDNQIWLTERATGKILRVNPESGSEKTVFQVPEIVSDADGQNGLLGFAFHPDFKHNPYIYISGTFKNPKSTDKELPNQTIIRRYTYNKTTDTFEKPIDLIAGLPSSKDHQSGRLVIGPDQKIYYTIGDQGRNQLAYLFLPNQAQHTPTQQELNSKDYHTYMGKVLRLNLDGSIPKDNPSFNGVVSHIYTLGHRNPQGLAFAPNGKLLQSEQGPNSDDEINLVLKGGNYGWPNVAGYKDDSGYAYANYSAATNKSQIKDLAQNGIKVATGVPVTKESEWTGKNFVPPLKTLYTVQDTYNYNDPTCGEMAYICWPTVAPSSAYVYTGGKKAIPGWENTLLVPSLKRGVIFRIKLDPTYSTTLDDAIPMFKSNNRYRDVIASPEGNTLYVLTDTAGNVQKDDGSVTHTLENPGSLIKFTYNGK

ST25 4190 cg28_0254

MCANFKPLKREHSYQLDLLEPTFDYKSDVYPSDDCPIILSHQNEWEWRKVKFGMLPPYAKEVSFKYATYDARTETVDHKRSFKHAWSNDQFGLVPVEAIYEPKYINGKAHWYGIFRQDGKPFTLAAIYEETIIDEQKVRTMSLLTINADRHSFMKQFHKPDDEKRSIIVIPDHLRNDWLNCKHTDAPNFFLDMPLNEFTSLPKAEMNKSKKS

ST25 4190 cg29_0255

VFRKLHTFGVTGERFIALQTAGNLATTQAVIGHLQNALILQQEPNLYSINTMFEVADLVGKTLKSVLEDISSDTQEQMNYACSILVGGQIKGGDMQLYNVYPQGNFICATTDTPYFQIGESKYGKPILDRALYYAMPLDDALRCSLISFDSTLRSNVSVGLPLDALVYSKDSFVIPMGKRITEDDPYFSQISRQWSDTLRRGLQEMPKPTDDYWR

ST25 4190 cg29_0256

MNWAISVPGDKTLKRDIFNNIWITASQRYEYQHLTFMAQGIVELLNTEIGGVDVPIPSKIFLQSTAATQYDSEMLDFAQKIVTVKDRQHIALLSENILQKMPYQPDSTSVHTTATQAFHAAQGVCQDHAHVLIAMCRALQLPARYVSGYLFDQNYPHLASHAWAEVFLDNQWYCFDVSNQLFTPKHHIYLAVGRDYLDVAPIRGVREKGGTENMMSVVQVLAC

ST25 4190 cg30_0257

MKELSVYPVIYDQKVAWGDMDAFGHVNNVQYYRYIESSRILYMENVNILTPNIYTVVASSQCKYLKPVFYPDVLKVGVRVEELRKSAFRMAYILWSESQQQIVATGEAVMVCVNKMTGLKCEIPSEVKSKIIELEKSVGHDLIHLA

ST25 4190 cg30_0258

LALLSYGYGMGYVYIYWRQLQLQTNVWGLVFTFVVMSFIAQVIWLWIKRYSSREQRKRENIFQFKNLHPYEQLGIVWLLEAAEDQRVFIERVFTQSGLLKNIIDAKFLVLSGDYQKALEALDQSPPMAFELAELQRIEIFLAQNEAERALTHLEFLYQHQLSPWLEEIETAYQQRLTALWGQLALQQPWVYLRSMKYGLLDAEHRDLWLQQLLQQFDQASIDDLQALQQRYLDLESEIQTRPYSSKLLWLKLLARMPEMSIQHEVLTLHLLKEQFDPDVFYLWFQQQLLKQVPDYADVEEKINQFESQYMNLPVLTFAKWHVYMATGRQAEAEILLSLYPDNILMSYLRIKSTLKEDDELIKQLNLIFENDANFLKFKI

ST25 4190 cg30_0259

MRKLVSIIILLGIAWVAKLSYDMWQISRTVPELQQSLLQSEQQYANLNDQLVALQRQIQNQPSHNSKTTPLATTEVVQTGIAPTILIKQKLELIQFAIDQQQFIYAVDHLTQLQQTLPQYGIAPALQHSLNQALEQDKQAIQQFVLAQNQRHQLIDDLLQNIDKNIQQALKQPKFEMDQSEAVSWWKKWFRIEKVETPSINLMNRSVVLKEVQLRLLIAEQALNQGKMAEYQNELQTVMQKLNELPDATSQQLKNRIAKVAHLSVVPVPKLSTLGLIGS

ST25 4190 cg30_0260

MLFINTRPDSRAAELTHALRKKGFQVESLPLLELVAQPFSESLSQLYQQLTGAQAIVVVSPTAVDVGMQYLTQSGLKIDELKHIQWIAVGQATANRLHDYGIEAHVPEVETSEGMLNLPLLHRMQKSGSVAFWRGEGGRQFMMETLQQQGIQILNFVLYRRQCPQASYSTFKHLTQNSNYNLDKWLVLITSEASWKYWLELCLKNKAQPDCVYLVLGPRLFQLVKDYRDQQQAHFHIIQLENLSPSGMIHHIQAL

ST25 4190 cg30_0261

MLEFMKTLKIATRQSPLALWQAEHIRARLQELHPDLTVELVKFVTQGDKILDTPLAKIGGKGLFVKELEAALLDGRADLAVHSMKDVPMALPEGLTLAVICEREDPLDAFVSNQFEKFADLPQGAKVGTSSLRRKSQILKQRPDLQIIDLRGNVGTRLAKLDDGQYDAIILASAGLKRLGLAERIRHCIEPSVSLPAVGQGALGLECRADDQEVLALIQPLLHPETDVCVRAERAFNAYLEGGCQVPIAGYATLQDGKIHIEGRVGSVDGQTLLRAELTDEADNAQQLGENLARNLLDQGAGDLLKALY

ST25 4190 cg30_0262

MGHEVVATAHHGQDALEQARLHHPDVILLDIQMPGMNGLVCAEQLSQLNPRPAIVFCTAYDQHALEAFKSQAQAYLLKPIDPQELEQTFKQLTQLTQAQLSALPKPELLQDLKTQRHQIAAKTYRGVELIPVENIYYFLADQKYVTVRHKNGSVLIDETLKELETEFADQFIRIHRNALVSIAYLDGLELVSSGQYQVRLRGLDERLSVSRRHLSTLRERIHQL

ST25 4190 cg30_0263

MQARIHPHFLFNSLNNVVSLITIDPDKAESMLISLSRLFRASFQELKLVSLHEEIELSKQYLMIEQIRLGERLKVEWKVEIEPLQLKQVTIPLLTLQPLLENSIFHGVEPMMGKATIGVLVEILQNQVSIVITNPYTHDTINSRKGHGIALENVKQRLKAYYGNSVRFQVYKGEALYTTIMSYQYQSK

ST25 4190 cg30_0264

LAEAQSWHALNGSRLLQYIVFINWVILSFFALVEHFQDFFSQLQQKYALVIGFVMLQAIVVVTTIISNFFQFGVLKKNICYSAGLEHLFYQCPFIFKLWNIIRGLLSALFICSRTMVTPTICRIKCPYSGNAGSHSSSFFI

ST25 4190 cg30_0265

MTTSSNPPNSAAPNQTSGMWGGRFSEATDAFVAEFTASVQFDQRFYKQDIAGSIAHATMLAKVGVLTEAERDDIIEGLSTIRAEIEAGTFEWRIDLEDVHMNIESRLTQRIGITGKKLHTGRSRNDQVATDIRLYLRDEIDDILGLLERLQKGLLGLAAKNVNTIMPGFTHLQTAQPVTFGHHLLAWFEMLVRDTERLQDCRKRVNRMPLGSAALAGTTYPIDRAYTAELLGFEAVSENSLDAVSDRDFAIEFNAAASLIMMHLSRMSEELILWTSAQFKFVNIPDRFCTGSSIMPQKKNPDVPELIRGKSGRVFGDLISLLTLMKGQPLAYNKDNQEDKEPLFDAIDTVRGSLMAFADMIPALVPNVEIMREAALRGFSTATDLADYLVKKGVAFRDAHEIVGKAVALGVAEEKDLSELTLEQLQQFSDLITADVFDKALTLEASVNARDHIGGTSPKQVEAAIARAHKRLEQLYA

ST25 4190 cg30_0266

MSRQVFCRKYQKEMEGLDFAPFPGAKGQEFFENVSKQAWQEWLQHQTTLINEKRLNVFEPEAKKFLEEQREKFFNNDESVEKAEGWKPE

ST25 4190 cg30_0267

VLSHHISSQFNEDLQDVNTKFMTMGGLVEQQVANAIHSLLDTDANLAIDVQFKDNAVNQYERDIDEGLTLILARRHPAAIDLRMVIAMSKANTDLERIGDEAAKIARIAQNLCEEGGSPRGYMETRHIGNQVRVMIHDALDAFARLDADQALRVLLADADIDREYQSATRTLMTYMIEDPRHIARVINVMWVLRSLERIGDHARNIAEQVIYMAKGFDARHTKIEEIEAKVHEK

ST25 4190 cg30_0268

MLVAGLWSFTSSSFALDLVETYERAKLNDPTWQANQQQFEADQLNLGLATGALLPTVTLSGNITRNRQTVKRSNFPGVDQEGLSDALVSNTSTTKQATLSARQPLFRMDAWEGYKQVKTSVALSEVTLRLQKQDHVLNVAEAYFNVLRQQALTAAYLQEEKALLEQLNMMNAKLKEGLVARSDVSEANAQYQNARANRIATNVQLLLAQEQLSEYIGPYQDRLAVLRSDFIFQKPYPAQLDEWLGLAQQQNLKIQQARLQKRYAEDQRRVEKAALYPQIDAVASYGYTKQTPETLISTDGKFDQVGVEMNWNLFNGGRTRTSIKKASVELNKAQAQLDAAIRRANVDVKSAFMQVDTDRAKLEARKAAMDSSALVSQASKASYNEGLKSMVDVLLAQRNAFSAKQDYLNAQYDYLLNVLRLKAAVGQLGEKDLVELNSWLTYQ

ST25 4190 cg31_0269

MTEQNQTVSPEHEYLGAGKSLAYGLQHVLTMYGGIIAPPLIIGTAAGLEPAQIGLLIAAALFVGGLATILQTVGVKYIGAKLPLVQGVSFAGVATMIAIVTAGGGLSAVYGAVIVASLIGFFLAPYFSKIIRFFPPIVTGCVITVIGLSLLPVAVRWMMGGNSKAPDWGSAENISLAFLTLAIVIVLNITRNATIRRLSILLAIVLGTVLAYAMGFGDFSKVANGNWIQFPSFFAFGMPTFELSAIIAMLIVTLVIMTETTADIIAVGEIVGTKVDAERITNGLRADMLSSAAAPIFGSFMQSAFAQNVGLVAITGVKSRFVVAAGGLILVVLGILPIMGRLIAAIPVPVLGGAGLVLFGTVAASGIRTLAKIDYSEQKNLIIVATAIAAGMIPIIDHSFYANFPKWVQTLFHSGISSTCLMAIVLNIVFNHLNFFKSKEVNKTSNIVIDSHH

ST25 4190 cg31_0270

MISSHILDTNLGKPAANVLIKLFNADGMLLGEGQTNADGRVTDFGLSEFSTGSYSLEFATADYFESLNTETFFPKAVIHFLVKDASQHFHIPLLISPFAYSTYRGS

ST25 4190 cg31_0271

VQLVEFNQASEADVKTFLKHCVQIESWANELLSQRPYASLDSILEYAAQAARTWSWEEIKSALDNHPRIGEKKAQAELSELEKNFSNREQSAISADEETQMALLKGNIAYEKKYGYIFLIKASGLSSENVLQALQYRLLNNPETEKRIVHHQLAEIALLRLKQELSA

ST25 4190 cg32_0272

DFIIQQYAKPTTLNQTKETFCTEDIDHLTY

ST25 4190 cg33_0273

MKNLFIMCLAVGIFSGCSTGTLITKEGMTDNPKWHKTNDVIFNKELGTFPNLQSLSQVRSGMTKDQLYELIGRPQYDDGWRPREWNYLFHFNTPGKGENGIITCQYKVLFDQNMIASSFYWNPIDPENAACPPSFNPPKKEEPKQQVSIKRYTLGADALFAFDKSDLNHINLKGREDLDRLVAELHKFKEIRTIRILGYTDYLGREVYNLTLSQKRAETIKQYFLNNQINAQVLIAQGLGEQDPVKHCADGLSKNLLVKCLEPNRRVVIDVDGIIE

ST25 4190 cg33_0274

VGGIFLSALISTGVFAAGSADIGTPIVTAAIGPSNNCVSQAATNLTSVVPWTCLGQTATGGFTLINGVQANAQNVGQANLDARASGQEAIAIGFIDTTASGTNSVAIGETANALGKNSVAIGKNIIADNSNINEDLSNNVAIGSNSIASTKGSQYTVGSGGAVAIGYGEHANGVGAVAIGSSNTAEGDGAVAVGRINNAIGDGTIALGDSSIANGDRAVAVGSVAHAIGTAAVAVGDSSTADGWSAISIGRVANADADFSIAMGDFAQALSYSALALGRNSYIDANSNESIAQGYGSSVTTARNAVAIGSDATVSNRNSGIAIGDNAQVIVGSHINDVNGLPGSIAIGLNARSVGGSEVIIGSYAAQNAQRYDDGAKNTYGSDSVMLGNQAGRNSQYLYATFIGDQAGQNSIGQHNSYVGQNAARYRTGSYNTALGSNALTGISNTTNSTGNSNTAIGTASGVSLEGDGNTLTGVYTGQRIIGNNNSAFGNNAGANIKGNENIAIGYLSGVSSQGNNNVLLGSNSNIGVNTNNVVSIGTNTRATKENSIALGANANTVTDATLESEANLNGLTYGNFAGQVTNTGMQLSVGSAGAERQIKNVGSGSISETSTDAINGSQLYATNNILGNVANSTIDILGGDASLNSNGTLSMSNIGGTGQNTIDSAIAASRTKVAAGTNVADVVKTTGSNGQDIYTVNAKGSTASAGSSAVTVTAGTADANNVTDYAVDLSQSTKDSLVKADTALQSVVTQIDGVDVKTVNKDDNKVNFVTGDNVELTANADGSITVGTAADVTFNTVNTTNLTATGETKLGDSFTVNNGGSYYTGPITEGNHITNKTYVDQATAASRTEVEQGKNITVTSSTGVDGQNIYTVATADEVDFNKVTVGDTTITTDGIVIANGPSITKDGISAGDKKVTDVADGLISADSKDAINGSQLFGLGNNLTQLFGGNALYTNNQITWSNIGGTGQNTIDDAIKHVNDQAANANQGWNVSTDSGSNATSTVKPGQTVNINGDSDNGVLVTNSGNDIKVGLADQIKIGAGDNAVSIDGNSGTIQAGDVLIDGSKGNISAGKVTVNGEAGTVNGLTNTTWNPNNIFSGQAATEDQLQQVAQNATAAATAAKTTVSAGENITVSSSKNADGSTNYQVATSKDVKFDTVTSGSITTDKVSVGNITIDQTGINAGASKVTNVADGTINSTSKDAINGSQLHASNTNIYNYLGGGANYETNTGPTYNVGGGSYNNVGDALNSLDQQVTNVSNQLEQAFYTTNKRIDDLEDHANGGIAQAMATAGLPQAYIPGKSMMAISGGTYRGESGYAIGMSSISDNGKWVFKMSGSGNSRGDFGGTVGAGIQW

ST25 4190 cg34_0275

MRKYDNATTVENEGEILAGFQYRSLLSYQAYHHIKDIKYGSKNRSTLDFFPLEHVNKTVIFIHGGYWQWCDKSDFVFIAPYILAKGAQCVLLEYDLAPQSHISQIVAQTQQALDFIAQQNWKTDEVVLVGHSAGAHLGALCLNHPLLSKATLLSGIYDLLPIQESHLNHALNLSQEDILKYSPIHQQEQINIPCTILCGGLELSELKWQSQNYFEYRLSQGDDMISFEIIPEINHYSILEHYFKFIFK

ST25 4190 cg34_0276

MSSFDEIQNREAGLHKKLSAKQMGMIAIGGAIGTGLFMGSKFAISFAGPAVIVSYAIGGLIAFALMACLAEMTVQHPTSGSFGAYAEHYISPLAGFLVRYCYWACIVLAVGTEITAVADYMKLWFPNVGSWVWIGFFSLTLLVVNAYSVKAFGLVEYWFSTIKVFAIIVFILLSIGILTQSNQGMTQVVTHLSGHGGFFPNGFSGVWIGVIISIFSYLSIEMIAVAAGEAKDPEKAVKKAFKSTALRLILFYLLSLFLIVTLVPWTVLIGADATSPFVMVMKNVGIPYADSILNFIVIVAALSAMNSMLYISTRMLFSLSRAGDAPKVFGRISSNGVPVNALLLSAVGIGIASIVYTINPASAFPIMIALSMFGALFTWGSIFVTHMFFRRHMVQQNIQLKFKIPASRFISLFGFIAILSITVTTWFTSEFKSTLQFGVPLVLVLIFFYYLKRSTVKLNLNSSEETLK

ST25 4190 cg34_0277

VQYQMITREQCLYWDQEDELKKFKDEFALPEGVIYLDGNSLGARPKKSLAVAQHIISQEWGEDLINSWNKADWWGLPTRLGDKVAKLIGAEKGEVVISDSTTLNLFKVLSAAVKIQADKFPEHKIIVAEKDAFPTDIYIIEGFIDLIQKGYQVELIDGVEDLSRALEKDVAVVVLSHVNYRTGYFYDMASINEQIHSKGALVIWDLCHSVGAVPMHLNQTDSDFAIGCTYKYLNGGPGSPALLWVNEKHRDQFWQPLSGWWSHKKPFDMAQHYEPANSIRRYLCGTQPVISMSLIECGVDIFLHADMKKIREKSLKLTDLFIQLVHQECSEFGFELITPLDHKHRGSHVSYRHEFGYEIIQALIARGVIGDYREPAVLRFGITPLYLGFEDIWNAVQHLKETMLNSEWKNKSI

ST25 4190 cg34_0278

MNVDAIDLKILKYLQDNARLSNQELADLVNLSASACHRRVKILETNGIIEKYQAKVNYEKLGIKIEAIVEIKLAQLTENDHNFFLSQIKNFDEVINAYIITGESNYVLHVATKDLNSFSHFVINTLNKIKGVVSINSKIILQKIIQKPL

ST25 4190 cg34_0279

MIETERLILRQWKESDSEPFIKMGLDEDVMRFFPKLLAATESISLIQRISALIDENGWGFWAVELKETQEFIGFIGLHNQPEQFDFSPCIEIGWRIATEHWKKGYATEGAKAALDYAFNILNKDKVVSFTATVNKPSQAVMERLGMRKVKYFNHPKLPDEHALQKHVLYEIYHPKLEL

ST25 4190 cg34_0280

MKIGFIGGANDGIVINAANGIDTYKLEWHYKAEKHCEEQPSLGLNGPEYDYYNLLILRKNKEAKLFYVLTTMDKEDIANKINEYWEYSTSVGYDFDE

ST25 4190 cg35_0281

LIREPVTLAFATASSESAYPKVMDALNKFGVPKKVTSFVLPLGYSFNLDGSMMYTTFAVLFIAQAYNIDLSFTQQILILLTLMVTSKGIAGVSRASIVVISATLTMFHLPEAGILLLLGIDQFLDMGRTATNVVGNSIATAVVAKLEGDKVADTEELPEPVLIKSTQEQTIA

ST25 4190 cg35_0282

MKQKKLLKYIVVAILLGVLTGWICHHFFNEGEQLKQIASYFNIVTDIFLRLIKMIIAPLVFATIVCGIVSMGKSTSIGSITLKSMTWFITASFVSLAIGMGLANFFQPGAALDLALPTAQQLSSSSLPTTTGFTLQSFLSHVFPRSIAEAMANNDILQVLVFSIFFGSALAFVNQGKEKDSVIIRLTDELSKIMFRITDYVMMFAPFAVFAAIASAITVQGLGLIVDYGILIAEFYFGLLLLWVILFSVGAIVLKKDIFRLG

ST25 4190 cg35_0283

MSQKKSVLFQQLLPVIKQYQQSGFTHEKIVELLKDQHDLNLVTVKTFKSYLYRYAKVNPAMSKNTATLQSMPTSREIKKSSKLEHVCYDIRGPVLRAANEMEEQGHKIIKLNIGNPAPFGFEAPQEIINDVALNLPNAIGYVDSKGIFPARKAICQYYQQKGILNMHVNDVYIGNGVSELIVMAMQGLLDDGDEMLIPMPDYPLWTAAVNLSGGTAIHYKCDEENSWYPDIADIESKITSNTRGIVIINPNNPTGSVYPRHVLEQIVALAKKHDLILFADEIYDKIVYDGIEHVAVASLAGDQLCISFNGLSKAYRIAGFRSGWMAITGDKSRAADYIEGLDMLASMRLCANVQAQYAIQTALGGYQSINDLIRPAGRLYEQRNIAWEMLNEIPGVSCVKPEGAMYCFPRLDPNIYPIEDDEKLMLDLLRAEKVLLVQGTGFNWPTPDHFRVVFLPAENELREAIGRLGRFLANRR

ST25 4190 cg35_0284

MGKVNKTDREWQRELSPEEYRITRQKGTEPAFTGQYWNTKQHGTYVCRCCGAELFSSDAKYDSGCGWPSFFRPLNGSVIDEHEDLTHGMVRTEIVCHDCEAHLGHVFEDGPQPTGLRYCVNSASLQLKTQEKNDEETYP

ST25 4190 cg35_0285

MSNIYQFEAELLEGDIKQFADYKGKVLLIVNTASKCGFTPQFAGLEKLYEKYKDQGLEVLGFPCNQFGGQDPGSNKEIGTFCQRNYGVKFPMFAKVDVKGPEAHVIFRYLTREAKGILGSSTIKWNFTKFLVGKDGSVLNRYAPTTKPEALEADIEKALAS

ST25 4190 cg35_0286

MGSMNAIPQTFEFWQDRRMPYVETRRSCFGRTCYKSHSHPTFSIGAIDEGNSVFQSSFGTAQKISAGTLVIVPAHVEHSCNPMPNQAWSYQMLHLDLAWLNQLYSEFQEQGLDLHIPQHKPLIIKDESLYEAFTEMNETLFDAQKLIFEKEQSLLHCLIHLLLPHFILEEIQKPQYLYKDFLNLIDVISSSEGFISLEELAQRVGLSRYAIIRLFKANVGLTPHAFQINLKINQAREQLKQGVPLAELAVNLGFSDQSHFHKAFKAHTGVTPRQFQLAAAQ

ST25 4190 cg35_0287

MEVFLVIAFTHFLALLSPGPDFFLILTSLLQKGRRYTYGIVLGITLGNALILAGCLFSFMLLGNLSSILLLIFKWLGALYLAYLSFLCFNAARSTVLNFSTDVNNLSDQAKDKRNKIKSLVLGLQSSLLNPKNIMFYSSLMLLIQHKFSLFQKLLMSVWMVGVVLIWNILLVKLLAYERILVKIKRSATGLYYCSAVAFILFAVLLITYK

ST25 4190 cg35_0288

LIPLSIGEPKHPAPEFVKQAIIDNFNHLSTYPNSKGLPELRQSIADWLTKRFKLNSISAENHILPVSGTREGIFSFVQALINREDAPYVVMPNPFYQIYEGAALLAGAKPYFINCKEENGYLGDFDAVPAEVWEKTALLFVCTPGNPTGTVLSKEQFKKLIALSDQYNFVIASDECYSELWFDQAPTGLLEVCAELGRDDYKNCIVFHSLSKRSNLPGLRSGFVAGDADLLKPYLQYRTYHGAAMPVQHQLASIAAWNDENHVEENRKQYRAKFDLFQSELGHLLPLQKPDAGFYYWLKVDNDETFAKMLMEKAHIKVLPGRYLSRDTEQGNPGANHVRMALVADLAQCEEVVKRLKAIL

ST25 4190 cg35_0289

LLNYVSSHHDIKAINQWRTDVEKQLQDSYENGQSIREIIKARSDLVDEALIFLWKHAELDQTELGLFAVGGYGRREMLPYSDVDIMILSEHEINEENEKRISTFISSLWDVGNFKPGISVRTIQSCVEQAATDLTVATTLIEARLITGNSQLAKWPRRIVSQTWTDKTFYDAKMAEQAKRYHQHNNTESNLEPDIKNAPGGIRDINQIGWIAKRHFRVNRIYDLVHLGFISEFELAVLEEAESFLWEIRHHLHRLAKRDENRLLFDHQREIAAKFGYVRQEGQPVNYGVEQFMKRYYRTAQQVSTLNEMLLAYFSESVITPRLPNYERKIEVVNDHFKIVDNKLAVQHHKIFAEHPSAILELFYILANRPDIEGIRARTLRLLILAAKRINQSYRDNPEHQALFMSIIRSPYRLYDTLVAMKRYGVLGNYIPAFGQIMGLMQYDLFHIYTVDAHTLLLLRNLNRFREPEFAKEFPVVSSVFQRLARQDIVFIAALFHDIAKGRGGDHSELGAEDAIEFGRAHGFTERECKLIAWLIQNHLLMSLTAQKKDISDPDVVKDFAEKLGDMEHLDYLYTLTVADINATNPKLWNTWRASLMRQLYTHARDVIRTGLGRPVDYQMLIEDTKFAASELLVNNFALADVEKVWQELGDEYFIKESADEIAWHTQAILKHGDNPEPLVLLRAHRKAAQDAVQIFIYTRDQPNLFATTVAVLDRMNLDVQDAKIITASTAFSLDTYVVLDRFGTLLTDPEREETVKNALVKALSQPDQYPGLMQRRIPRQLRHFDIENTVDVTLNEALQQNMVEISTLDHPGLLARVGGLFMMQGLDIHSARIATLGERAEDIFFVTKKDGKPLNNEEVKLFSEKLKAALDEASNQICQH

ST25 4190 cg35_0290

MIRMSVTLGTPLQSSAFKVLLLGSGELGKEVVISLQRLGVEVHAADRYDHAPAMQVAHFSYVLNMADPAQLKQFIEKIKPNLIVPEIEAIATEVLLEIEASKTATVIPSAKAVNLTMNREGIRRLAAEELGLPTSAYRFADTLESFRAACDDIGYPNFVKPVMSSSGKGQSRVKSFDEVDAAWEYAMQGGRVNQGTVIIESQIDFDFEITLLTVRAKNPETGEIETHYCDPIGHRQDAGDYVESWQPQPMTAAALEEAKRIANKVTTALGGCGIFGVELFIKGDKVWFSEVSPRPHDTGLVTLASQFQSEFELHARAILGLPVNTARHSVAASAVIYAGVDASNLSYSNLNVALTHPDTDLRLFGKPEGFKRRRMGVATARAENTDLARSLAKETADQVSVQTNS

ST25 4190 cg35_0291

MSQVKIYANEQTIMQYRELLSHAIHQALIEELKYPVEKKFQRFISLKPGNFIYPSDRSQHYIIIELSMFAGRSPATKKQLIQTLFRNIQEQCRISPQDIEITIFETPKENWGIRGKNADELLLNYQVNI

ST25 4190 cg35_0292

MLIGFLVLAGLYFKFKKIVPATLTAAIVYGLIYTYLPL

ST25 4190 cg35_0293

MPAQTATFWQGAKDSQAIVLTYLPVSFAFGVSASQFGFTPWEAFFLSCSMYAGASQFLVVALLASGSSIWLTALTVIALDIRHVLYGPALYNLIPNKLNLKKTAVWAWGLTDEVFASGMIQLSQRKQQWSESWMLGLSLFSWMAWASGSLLGGLFADQVAHLPKFLQAALDFLLPALFLSFLLAAFERKHSLVVAVSLVVSALACYWINLSAAIFIGIFSGILAGLFKHYVLKQHDEVEA

ST25 4190 cg35_0294

MFNRQFDMFKAKIRLVYIAMMSAGIFTLQGCDNGNQGAEKKVEIKPAPKLTNDATVYAQKAWTLINTVEHLVYEKQLSQIDDKVRKPLRQLSTDWRINVKMTDSVTEGKYALCRKALTSLDIWARETLENNSSVAQKQADYERDKAQCKDAIDTPNLGNTSPK

ST25 4190 cg35_0295

MKKLSTILTAGVLAMLSVSAFACPKGTQLQGGTGPNHKGGKCVAVHGKATAQKAKKEATKTKQEVKKDLTMQKHDAMTSMTHAQHESHQMTHQMKQDAVKTANTAKAATKP

ST25 4190 cg35_0296

MLISALLSIIAQLECKVKNSALKHLFCGNRV

ST25 4190 cg35_0297

LSLLEKQNIIKIAFRKIEILDIEKLKVIAQVHS

ST25 4190 cg35_0298

MDDRILDLIYINRLKENTWFSVLPEAFQKFILEHGKQITFEKNSYVFHAQDEFDGIYTVLEGSISLGYVDVNGNEALSAIAEPIMWFGEISLIDHEPRSHDAIALKKV

ST25 4190 cg35_0299

MSNLEQKLSQYAAYHLNHQNILTHFIGIPLIVFSILCLTARAGIDIGNFKVTLAMVLIAFSTIYYLFLDKVFGLIMLMILVAVYPLASQIAELSLGQWLAASIGFFVVGWAFQFVGHYFEKKKPAFVDDVIGLAIGPLFVLAEFIFMLGFRKPLHERILHEARSKREMMDLSH

ST25 4190 cg35_0300

MGFNCGIVGLPNVGKSTLFNALTKAAIAAENFPFCTIEPNTGIVPVPDPRLDKLAAIVNPQRVLPTTMEFVDIAGLVAGASKGEGLGNQFLANIRETDAIAHVVRCFEDENVIHVNGKIDPLDDIATINTELALADLETVAKAILRLTKVAKGGDKEAVATKAVLEKIQPLLDEGKPARAADLSDDERKLIRGFGLMTLKPTMYIANVAEDGFENNPHLEAVKKLAAEENAIVVPLCNQIEAEISLLEDEDRAEFLEAMGMEEPGLNVVIRAGYKLLGLQTYFTAGVQEVRAWTVKVGATAPQAAGVIHTDFEKGFIRAEVVAYDDFVQYNGENGAKEAGKWRLEGKTYVVQDGDVMHFRFNV

ST25 4190 cg35_0301

MQDQLIDLLITGTVDTLLMVGASAFIAFLIGLPIAVILVSTSEHGIHPSQKINQALGWVINITRSVPFLILMVALIPLTRWIVGTSYGVWAAVVPLTIAAIPFFARIAEVSLREVDQGLIEAAQAMGCNRKQIIWHVLLPEALPGIVAGFTVTIVTMINSSAIAGAIGAGGLGDIAYRYGYQRFDMQIMLAVILVLIVLVMLVQATGDALAQQLDKRKV

ST25 4190 cg35_0302

MTMVSFGSQVDFSLPHIKIRGLNKFYQSQGQKLHALKEINLDIPQGKILGIIGKSGAGKSSLLRTLNGLEQVNTGSIHIHQQNIAELSHSELIQTRQRIGMIFQHFNLMSAKTVWENVALPLKVSNYNKADIDQRVNEVLALVGLADKSNYYPSQLSGGQKQRVGIARALVHHPEILLCDEATSALDPESTATILALLKKINQELGLTIVLITHEMQVIREICDQVVVIDQGEIVEAGQVWSVFSRPEQQITQELLNLEQITLPFKISRLPDENSTHIIVKLKYEAEAHQVPDIQELLARFKAPVNLYQSQVDIIQGHIIGSLLVGIPNVEIDLSSIQQDALTAIAQFEVLGYARPAH

ST25 4190 cg35_0303

MAIQFKQHKPWLLGITILIVVLGLVGYRYIKNKQADDVLTIGISPPYAELLQSVANEVEKQGVHVKLVEFSDWRAPNVAVQNGDIDANFFQQSVFLRHAVKETGYDLHAFGVGSGSHVGLYSKKYKSLEELPSNARVAIPNDPVNLARALILLHRAGLIQLKDINNELSTTQDIIANPKQLSFVEVEGPQTAHAYNDVDLIFGFPHYLKMAKVTDPHSALFLDPIDKKYAILFVTRRDYQDKNQKLATFVKAFQNSKQAQDILDKDFGKGMWFQGWK

ST25 4190 cg35_0304

MAQTASKKWLGIGLVVVVLFVALFLWNKHRQSGSNELVIGISPPFAKPLQAAADEAKKQGLNVKLVEFSDWNTPNITLNHGDIDANFFQHQPFLDNAIKETGFKLKAFAVGAASHVGLYSKKYKSLDELPQNARVVIPNDPVNQGRALLLLQQAKLITLKDSNNHLSALKDVVSNPKNLQFIEVEGPQTARAIDDVDLAFGYPHYLRLAKTADPNSALLLDDNTNKRYAILFVVRDDYQDKGEKLKKFVEIYQNSPKVKAVLDAEIGPKLWFPGWK

ST25 4190 cg35_0305

MADFKAKEILLNAFDMNCVGHINHGLWTHPRDESHRFNELSYWTEQAKTLENGLFDGLFIADITGVYDVYQNGIDLTLKESIQLPSHDPSTLISAMAAVTQNLSFGVTVNLSYEHPYQFARRFASLDHLTQGRIGWNIVTGYLDSAERLIGQKGLKDHDARYEQAEEFLELCYKYWEGSWENDAVKKDKARRVFTDPSKVHTIHHHGKYYQSEGVFQVSPSVQRTPTLFQAGASPKGMQFATRHAECVFIGGDKPEKIREQVKKIRTLAEQQGRSANDIKVFVGITVVVAETHDLAVQKLNEYRQYASPEAGLAHYASSTGIDLSKFADDEAIPYQKSNSIVSITEKFKEQQITKNDLKAQHVLGGRYPLIVGSGEEVAEYLIHLLDETDIDGFNLTRTVAPESHHDFIRLVIPELQQRGRYKTAYKTGSLRNKIFNRGDHLPEQHPVQAFRCSPYNNSKNLTKIEEITA

ST25 4190 cg35_0306

MTSYQSVQTKQTSASGQAHIIQNDAEALEIAKQFAEQFKKTAVERDAKRILPYTEIDALSQSGLWAITVPKQYGGAEVSSHTVAKVIALLSGADGSIGQIPQNHFYALEVLRNTGTETQKQRLYGEVLNGTRFGNALAEFKTKTSTHKQTNIRPHENGYLIQGEKFYCTGSLFAHRIPTLVLDDAGREYLAFVKSGSQGLKLVDDWSGFGQKTTGSGTVKFDQVFVDADDVIPFDTAFLQPTLVGPFAQIMHASIEVGIARAAFEESLQRVHQARPWIDSNVETANQDPLTIYELGRIAVDVRASEVLLKQAAQSIDAAKIETTPESIAKASIDVAKVRAHSTDIALKASSKLIELAGSRGSQSQDGLDRFWRNARVHTLHDAARWKYYFIGNYVLNGVLPPRRGTL

ST25 4190 cg35_0307

MTAIQGVIMSNSLSKTTAYVQVIQNDQQAINAAYQVADFALEGRNTRDQQRLLPHEQIESFSQKGLGGIRIAKKYGGAFVSNKTLAQVFRILSKGDANVGQIPQNQISLLNLIEIMGTEQQKQFIFSEILAGKRLANGGPERNTHDSKTLKTTLTIENGKYFLNGEKFYSTGTSFAHWLAIKAVHPEGHVVLVIVNRDAQGIEVINDWNGFGQRTTASGTVKLNQVEINPELIFDERLLTQVPTYRGAYSQLLQVAIDVGIAEAAFEDTLSTIHKARPIIDANVEKASFEHYTLQEVGKLNILLDAAILLLDEAAEYLDELDQLQTVTDEQVAKASILVAEAKVYANDAALQISEKLLELGGSRSSLSQHNLDQHWRNARVHTLHDPVRWKLHAIGNYYLNGHFPARHAWI

ST25 4190 cg36_0309

MSEYEEKKLFIEKIAQACDLLYDLSMEGSRITGRAKNNDDLIKVTNLLRDAEKIEEELSFSSNGLIITFYKVLNIFKNFDKLLNDSNYRRDVPAQFFYLIESKKAFYFDRVDSYFENYLNIIKIYKFFEEKSDHKSSEHGNQNLIFLGNKKLVVTDEYHSQDLIEIPDAIKFMSKFLTEDLSNGITNKEKDQILKKTLVNFFKDCETIKFSQIINDFKKLNHCVDSELDIYMSKFSYEDIKKEVEKEKVDFIVRLXKVFSDIQTQLIGVPVSVILAADKLKLGKLALEDPNKFYGISLTNLLVILAIGFYAIILAMLIRNQSNTLAALKDEIDHHESLFKNKHKGIAQKFINSFVQVKNRYEHQKKMLLWVDVLVCIAFGIIFLMAYVSMFSHLEILLPFMGGLLLIGIFIFYRSYE

ST25 4190 cg36_0310

MTIEILNAVIHGVKKQTNSSIAEPKYKPGCFNTSDPLLVKFTNDLLKAYSEEANTWADIASADINIFHQNLIKCFKEDEKTIDFYNFSKKVVDQIVGEIKRKYKATGGYVLLINYKYNITNYLLVVMLKLETRFGIDEKDLSFFETESFTMKNFHEAVRMNLDTWLARSVGEGIDEDGLPERCFAFVKKRGVDEDITQYFRLALGCENFAESTANTSALIRALDAYVESKSFLNEDSKMEFREQKRDILHNYLSTKIKEKQPVSLRDITALISPTDEKIKENEFLEFIKSNDKFKIDETFNPHKGKVKDLERVSGKIKGGSVSFPITEIGESIFYDQDIRELKIQKVPDELHQRILNAKGS

ST25 4190 cg36_0311

LFSSLLAVGLLQGCGDDSSSDQPKDDSNTTPPNLIVDNDPTSCSKLAQDGSSVVVGSNQNGDPAAPEGASGYRLGNTVKYADKYMVVANTPLAVKAGCDVLKAGGSAVDAAVAVQAVLGLVEPQSSTIAGSGFMMYYDAKTKQVTAYDGRETAPAAANEYYLIRQNIYDPNSPAPVPSARRSGRSIGVPGVMRLLEQAQKEHGKLKWNQLFGEAIGLADNGFRIPGRLADAIASNASNLALDANAMATYFYPDGSPRKVGETMTNKAYARTLEALASQGANALHTGPIAQAIVAKAGQTVGDDQARTPITPSLMTLQDLSNYQVKKRDPICTTYRDRYYVCTMPPPSSGGIAVAQTLGILENFDMSLYPPKNPENEGGVPDVMGVHLVSEAERLAYADRDKYVADTDFVRLPAQGIPSFIDKNYLKQRAALINPNQSMGVAPAGNFNPASGVDTTVEHGTTQFTIVDAYGNVVSMTSTVESSMGSFHMVDGFLLSNQLTDFSANPYDSTGALVANRVEGGKRPRSTMAPTLVFKGTTPDEFYMATGSPGGGTIIQYVVKTLVGALDWNLNAQQATSLVNFGATNSKNTNVDSSNVQLSLVDLIEGLKAKGHGISNTAQTSGISTIMKVNINNQSKYAGGVDPRREGIVLGNGAL

ST25 4190 cg36_0312

MKTQTPFAELSGGRLLLAAFVIALSNFMVVLDTTIANVSVPHITGNLAVSSTQGTWVVTSYAVAEAICVPLTGWLAGRFGTVRVFIFGLIGFTVFSFLCGLATSLEMLVFFRIGQGLCGGPLMPLSQTLLMRIFPQEKHAQAMGLWAMTTVVGPILGPILGGLISDNLSWHWIFFINLPVGIVCVLAAMRLLRVAETETISLRIDTVGLGLLILWIGALQLMLDLGHERDWFNSTSIVVLALTAAIGFVVFLIWELTDKHPVVDVKVFRHRGFAISVLALSLGFGAFFGSIVLIPQWLQMNLSYTATWAGYLTATMGFGSLTMSPIVAKLSTKHDPRALASFGLILLGIVTLMRAFWTTDADFMALAWPQILQGFAVPFFFIPLSNIALGSVLQQEIASAAGLMNFLRTMAGAIGASIAVTVWDDHAKVARSEMVSNLHTTEVQNTLLQNGFTADSTLGIISNLVDKEAITMSANHVFLLFAIVFVFAGLVIWLCPKPKQVSGMPSH

ST25 4190 cg36_0313

MTDAQSNVQETVPTTSASDDNMQNKRKKFLGFFALILLIAAILYAIWALFLNHSVSTDNAYVGAETAQITSMVSGQVAQVLVKDTQTVHRGDVLVRIDDRDAKIALAQAEAELAKAKRQYKQTAANSSSLNSQVVVRADEINSAKAQVAQAQADYDKAALELNRRAQLAASGAVSKEELTKAQSAVETAKAGLELAKAGLAQATSSRKAAESTLAANEALIQGVSETSTPDVQVAQAHVEQAQLDLERTVIRAPVDGVITRRNIQVGQRVAPGTSMMMIVPLNDLYVDANFKESQLKKVRPGQPVTLTSDLYGDDVEYHGKVVGFSGGTGSAFALIPAQNATGNWIKVVQRLPVRIALDPKELAEHPLRVGLSMEAKVDLSAK

ST25 4190 cg36_0314

MLDLYKIMGVYHAFKFRQRMSDEKKNWTGNIQLGAKTDLNVVLNQLCKAFNSCPFFKHSGMIMRVVDGHIEGYVEMQPDLIGNLAFQVLHGGVAATLLDSIGGIVAMEHLYRRSTPETLPETIKQVTRLATVDMRVDYLAPGRGKYFIARAEVLRLGRKGCTMRMTMVNDEDKQIAAGIASYAY

ST25 4190 cg36_0315

MTYTFNRPAFPATRMRRIRKNDQLRAMVSETQLTTNHLIYPVFVLPGQNQTQDIPSMPNIQRLSADLLLKKAERLLELGVSKLALFPVTPQEDKSLTAEAAWREDGLVQTTCRLLKKELPEMVLITDGALDPYTTHGQDGIIDETGYVLNDETVECLIKQALSHAEAGAEVIAPSDMMDGRIGAIRQALEANGHIYTNIMAYSAKYASSFYGPFRDAVGSASNLKGGNKYNYQMDFANRAEALHEIALDIQEGADMVIVKPGMPYLDVVREVKDTFGIPTFIYQVSGEYAMLAGAIQNGWLSDSVILESLMCCRRAGADGIWTYFAETAAEKLKEMN

ST25 4190 cg36_0316

MHIAIIGAGISGLMSALELVEQGCTISIFDQQQAGQAASWAGGGILSPMYPWRYVHAVNQLAQFGKASYQAWNQKLYPVTGIDFEIHDTGMLIFDEEDFDIGLSYAEQHQEPMQRCEYLQRDALEQVNPHISDQFQEAIYFPELSNIRNPRVLQSLIRYLKQHPNVEFFEHTPVKKLIQQGDVIQALQTEDGRKHTADHFVITSGAWSHYWNSQLQLEIPVEPVQGQMLLFKTPAHWLPTMCMNRVMYLIPRMDGHIVCGSSMAHRGFDTSTDETTQHNILEACLEMVPELADFPIVHRWAGLRPSSPNGVPYIGKMPEMGNLWANFGHFRNGLCMGSGSAQLLRQLMLGQPTLVDAKAYSPERLQNKILA

ST25 4190 cg36_0317

MNKNVLITGASGFIGTHLIRCLLQKNYNVVAVTRQAGKKSDHPALQWVQKFEDISTRQIDYEVNLAGANIGEKRWTESRKKQLIESRVNTTQKLYAWLKQSQIFPEVIVSGSAIGYYGIDAQEKWTEVCTEQSSPQPIFMSQLCQEWERAALADAQQNTKIIRLGVVFGQGGGILPKMLLPIRLNLIGQIGHGRQPVVWVHIEDVLNAIEFIFKHPQSAQIHNVVAPENVTQKVFVEQAAKVLNKKPMLSAPSTVFRCLLGEQSQLILNGQYVKPAALQAEGFEFAYPQLKMALENILASG

ST25 4190 cg36_0318

VLTLIVGVIAALLPASVGGILTAVPYLGAMIFVLFRFLKKERRAPTVPEKKKFTLGFTLIFWGYNLCGVLFGLFLFSRKDPEILQNFMLYLKQPQFLSIMVIMLLMLAIPLYLITYWFYGKQAQRMANKMFNAS

ST25 4190 cg36_0319

MNNIAYFEIQSSNPARDAQFYQAVFGWQFKLDESLPIEYYRIETPSIMGGLLKRPAQTPPMEYGTNAFTCSVQVENFDEVAAKILAQGGIVAMDKFAIPGRAWHGYFVDLDHNVFGIFQVDENAA

ST25 4190 cg36_0320

LLLSGQTMNFENKMAIHKYEVKIQWEGNTGTGTSSYRSYKRDFSIQHPQKTTIQGSSDPAYLGDVTRWNPEDLLVASASACHKLWYLHLCAVNHIHVVSYVDHALGFMEDTDPVKRGHFTQIILRPEVVLEKGADQELAAKLHEEAHHECMIANSVNFPITCEASFSFAE

ST25 4190 cg36_0321

MSVSMSVHFLHTSDWHLGQFFYNHSRHYEHQQFLSWLLTQIQEKQPHALLIAGDIFDVINPGSQAQKQLYQFLADAHRIAPHMQTLMIAGNHDSGYRIEQVEPLFRKIQCKNCGCGSLE

ST25 4190 cg36_0322

VGVVRWNEDKTLDLDRLLLPIYNQNQDIVAWCLALPFLRSAEITGFNEHTTNSKNAIAYLHQQLIAEAKRRKTPDQALILMSHAHMQGGETSDSERPIIIGNEEALSTTLFEDAVDYVALGHLHKPQKVGQPHIRYSGSPIPLSFSEINYKHQVVEVKIDPSQDTDSRLQFEAVEIPRCIQLHRIRGELNEVLQQLKALPHGVIENIDHREYVDIEYYSLTPPQPNLRQQFEAALPPDRYRLVRISRQYVNKDATNSNTTQHIALEPPTPEKLFQNIWEKQGYNADDAVLKDFLSLVQEAQKHLENDASH

ST25 4190 cg36_0323

MKILSIRIKNLASLADEHFIDFESAPLAHAGLIAIVGKTGAGKSTILDAMCLALFNRVPRLKDSDGKLKDVDGSELLTNSPLTVLRRGTGHGFAELCFIAQDQKRYLARWEIKRARENPNGKLQSVQRHLKCLTDGVVLADKAKAVDEKVKQITQLSFEQFTRAVLLAQSEVTAFLKARDSERGELLEYLTNSSIFAKIGELAFRKTADIAKQRKQLEEFLGHIEILSDEEIAAFTEQYQQAEQNYQQLEQQKHVLDKQQQWFERKAKLEQEVQAKQQQFQTQQNHHQQLASEREQLKRLEIFSEIRPQVFQQAQNLQTLQQVEPQIQQAQTKFNELVQIFETGQKQYQLAEQQLKQTLDFEQQHQHALNQVRQSIQERAFIADEYKKCKEKRQVLEQNLSPLQQQRNAVQQHIAQLEQNKIHLQQQLIQTQQYAELDKGLSAHLHQLGQFIQNYQTIEEQLGNPTLARQKLSEAKSELEQLTASLGTVEQIELKLEQQRKDKDQKLAQVTQLDLIQQKIKFIMSYMLSFSNSPKNILKPPRKKSNLKPFAS

ST25 4190 cg36_0324

LQQQQEQQAIALEQTKFNAWQTQQHALTQCRAELDQVQKYLAQLQVKQTHLQQELEQAFNLNQLHIELNQAPEQILQTLNELRQATQTAISLFDSENLRLTQAIKQHNQLVQTIQRNESLLNTAQQWQQQVQHIVECLSEAEQHAWQQASSQTAKQTWAILDARAKQLEQQEQLSQRFEQQQQELKMLSANLEQMTKQIDEIDQNLQEITLKGQQNNEKAVSLIQQMTGRSDIKPHEWLIEHDAKRQHQQTAYHEAKQRFEQTRQHFEQQKQALDQLKHQYQHTEQHQQQIDGQIQNWLKAHTDFQASDLTALIQINSAQEQDIRNRLNHAERLLSEASSALKTMQEQLSEHLQTQPDIEYEKLVTLIQDNMAKLKAQLEVRDGLKLKLELHQQNLAKQQKYAEQIQNIQQEEHRWSKISGLIGDAKGKEFRDYAQQYHLDILVEHANQQLAMLSQRYTLKRLDQSLSLAIIDHDMDGETRSVASLSGGESFLTALALSLAIANMASGSMKIESLFIDEGFGTLDASSLHMVMNALDQLQNQGRQVILISHIQEMHERIPVQIQVKPLGAGASTIEIVG

ST25 4190 cg36_0325

MNYLQDARQHVLQQIRTACELAQRAPETVQLLAVSKTHPSERLREMYAAGQRAFGENYLQEALDKIDALQDLDIEWHFIGHVQRNKTKHLAEQFDWVHGVDRLIIAERLSNQRGDDQAALNICLQVNIDGQDSKDGCAPEDVAELVAQMSQLPKIKLRGLMVIPAPDNTAAFADAKKLFDAVKVQHAHPEDWDTLSMGMSSDLEAAIAAGSTMVRVGTALFGARDYSQKG

ST25 4190 cg36_0326

MTYFDLLGEIMDITELLAFSVKNGASDLHLSAGMPPMIRVDGEVRRINLPALEHKDVHRLVYDIMNDKQRRDYEEKLETDFSFEVPNVARFRVNAFNQNRGAGAVFRTIPSKVLTMEDLGLGQIFKDICDYPRGIVLVTGPTGSGKSTTLAAMLDYINENRYDHILTVEDPIEFVHQSKKCLINQREVHRDTHGFNEALRSALREDPDIILVGEMRDLETIRLALTAAETGHLVFGTLHTTSAAKTIDRVIDVFPAEEKDMVRAMLSESLQAVISQTLLKKNGGGRVAAHEIMIGIPAIRNLIRENKVAQMYSAIQTGANHGMTTLDQSLKGLVARGVISPQTARTAAKQPESFL

ST25 4190 cg36_0327

MDFNDLLNLMVEKKSSDLFITDGVAPSMKINGQIVPISKNSLSGEVIGQLLHSIMSEKQRREFAETRECNFAIMNREKTARFRVSAFQQRDMPGMVLRRIETKIPSIDDLQLPPVLKDLSMTKRGIIIFVGATGTGKSTSLASMISHRNHNSKGHIITIEDPIEFIHEHAGCIITQREVGIDTDSFEIALKNTLRQAPDVILIGEIRSREVMDYAIGFAETGHLVLATMHANNANQALDRIIHFFESDRHSQLYMDLSLNLKAMIAQQLIPTPDGNSRRAAIEILINSPLISDYIRKGEIHEIKDLMKRSRELGMQTFDQALFDLYKAGQITYKDALKHADSPNDLRLTIKLADEGPDQLSDGKQHLTFDRQ

ST25 4190 cg36_0328

MPISNQDLRKAGLKVTLPRIKILELLENSKQHHLSAEDIYKTLLEQGEDVGLATVYRVLTQFEAAGIIQRHHFENNHSVFEIMQEDHHDHLVCQNCNKVIEFTNDVIEKEQHSVAEQHGFTLTGHSLNLYGYCNEPECQEALRKK

ST25 4190 cg36_0329

MQKLVLTLFVTSLLAGCSIFGVYKVDIPQGTPLTKAQASQVQVGMNFQQVRFLLGSPTVTDPLNPQRWDYIYNYIPGTYAKKAKIPAAHGQHLKIYFDETGTVTKIEGLETIPESQPGLPASKEAILTAPPL

ST25 4190 cg36_0330

MERTQQVWVAFAVPEQQFLISVPFEEGMTAAQAVQASGLSNQVDLPEPLQLGIFGIKVETDAVLHAGDRVEVYRPLTINPKDIRRKRAAKNPVGRYIKGNRFKQSAQ

ST25 4190 cg36_0331

MATLAPTMGIQIDRQIDFWLLWFGTMLLLALPVCYLEIALAKRSKTTALNALSSLTREADSSPKWRVVGWLAVVFIPFLAGNVLSTASNILVAQFAPSISGQIIFAGLAVVALVLSFIPRQILILLMTLGVIASIVLANMMGSTLQPWHWTSVEFKEWGNATVLALVASGLGLGLYWQNSVGAVQAQEGATKTVLPIWLAQLIAVVAFGFFSLQAQLPVLTWIFTGVMTSALFVQLAREQFAQRQLMPVLQWVIIVVAIAVWAVPEVHNLFTLILMLWGLLICLIYAVFAGWIMKISHLRKSMNFSNELFYNLWRIAVRIVLPLSIIVAMIAVIGQSI

ST25 4190 cg36_0332

MKMKWASEYNTGIDVIDEQHKRILDYINEIDDVKDDEDRRRIKDVLDNIIDYTQSHFTFEESLQEEADYKYRVPHKRVHDLFIKKIELYRERFEMGHTIEAELQEILAKWLINHIQHDDADYVGAVKENMMGIIREKEKKKGKNWSVPFFLITLN

ST25 4190 cg36_0333

MNCATPTDITFSFRGFSQRAGITSDHSDGFGIAFFEDKACRLFVDNQSAVESPIADLIRNYPIKSRNVIAHIRKATQGKITLENSHPFIRELWGRHWIFAHNGDLHDFNPPLSGRFTPVGNTDSERAFCYLLDQLVEVFGYEEPTLEQIFEVLEKISPQIAEYGTFNFCLSNGQALFSYAITKLHWLVREYPFNNAHLIDLDVEVDFSQVTTPEDRVAVITTEPLTHNETWIAYQPGEMILFQHGKPIKKAITQVERLIREEKNPELKRITRADQY

ST25 4190 cg37_0334

MKPDISELSVEELKRLQEEAEALIASKKDQAIEDAYNQIIEIAENVGFSVEQLLEFGAQKRKKTTRKSVEPRYRNKNNAEETWTGRGKQPRWLVAEIEKGAKLEDFLI

ST25 4190 cg37_0335

MKKKSKQWKWWFFALIAFLIFIILQIPATWLISKFSKNNQTVHNVSGNIWQGQADWHRGALRGTIHWKTRPLDLLLLRFAADVDIHSGNTQLTGIMAYGFGKKVIVRDMNGQIAPETLKQIVNWQWPVNSIQLKDIQFNYKKEQGFAAVDGQLHWGGGALIYNIGDRQDRMNMPSLSGQLTDQNGQLQVDIRDQRNQKMANLLLDANMMLDVQLTQRLLLNVPSYDGKAGLDTFVISSRQPLLQGGN

ST25 4190 cg37_0336

MKVWIDKLQQLQWQKLDRLSVVVLAILILWLCWKLASFFWLVIAPPQLMQFDRVELGSQQPQIPNISTFSLFNEPSANAAQESVNLELQGVMVGYPNRFSSAVIKIDNTAERYRVGETIGSTSYQLAEVYWDHVVLSQGNGSTRELQFKGLPNGLYQPMTPDASQQSATPSQPTEPMNTAQQALGQAIQQMQGNREQYLRDMGVSGNSGEGYEVTERTPTALRNKLGLRPGDRIVSLNGQTVGQGQTDVQLLEQARRAGQVKIEIKRGDQVMTIQQNF

ST25 4190 cg37_0337

MALLNHQRPLWALLAAAPLIATVSSSAYAQTWKINLRDADLTAFINEVADITGKNFAVDPRVRGNVTVISNKPLNKDEVYDLFLGVLNVNGVVAIPSGNTIKLVPDSNVKNSGIPYDSRNRVRGDQIVTRVIWLENTNPNDLIPALRPLMPQFAHMAAIAGTNALIVSDRAANIYQLENIIRNLDGTGQNDIEAITLQSSQAEEIITQLEAMSATGASKDFSGARIRIIADNRTNRILIKGDPQTRKRIRHMIEMLDVPSADRLGGLKVFRLKYASAKNLSEILQGLVTGQAVSSSNNSNNSSNSSNPINSLIGNNQNSGSNTSGSNGASISTPAINLNGNSNSSNQNNITSFNQNGVSIIADNAQNSLVVKADPQLMREIESAIQQLDVRRQQVLIEAAIIEVSGDDADQLGIQWALGDLSSGIGLLSFSNVGASLSSIAAGYLSGGSAGAASAIANGANKGNGATLGLGNFDNSRKAYGALIQALKTNTKSNLLSTPSIVTMDNEEAYIVVGQNVPFVTGSVTTNSTGINPYTTVERKDVGVTLKVVPHIGEGGTVRLEVEQEVSAVQDSRGQAADLVTSKRAIKTAVLAEHGQTVVLGGLVSDDTSLSRQGIPGLSSIPYVGRLFRSDNRSNVKRNLLVFIHPTIVGDANDVRRLSQQRYNQLYSLQLAMDKNGNFAKLPEQVDDIYNQKMTPPSIASKPKNYQQVPSGGKSSTITTPVAVEPTVQKQTLQLPDPEINRTKNTVTTTTLRPSTAP

ST25 4190 cg37_0338

MTWKLQAITGEFTGQEINVDRDMLVGRHQDADLLLQAAEISRRHAALLLKDQALWVQDLNSSNGTFVNDIRIEQEKQLHDGDIVQFASLKFSVFAPAQENTDLAEIEVEPVQTAPTQDLSDQGMPSIAERAAETEVSRDGMPQRVSVPKPAPIPEGVDIHAQPEQTPVAIEEPVSRVTEEKEQQKNASIGLVTIIILVILAVLAWLFFK

ST25 4190 cg37_0339

MSVAQLSKRDLILFDLDGTLVDSAADLYRSMNLSLQSLSWPSVTEAQIREWVGKGASKLCESVLLHIFGKLDVEQHKVLLQKFVEVYGAELCVNTQIYPGVPEFLKHCQTLNIKMACVTNKPVKLAQGLLDALELSSYFQVVLGGDSLPERKPHPLPLLHCMESLKISASQSLMIGDSSNDIEAARRAGIDCIVVSYGYNHGENIYDCQPQQVVDSLAELIV

ST25 4190 cg37_0340

MTTLAQFEQLKAAGYNTIPVYRQRLADTETPLSVFARFKDQTQAYLFESVEGGENWARYSMIGLGESTVFSCNAGVLSIQHADGSVTQQNCLDPFQYIREFQKQFKVPAAKLLPDLPSFTGGLVGYLGYDAVRYIEPRLKNVPTADPIALPDLWLMLSKTVIVFDNLKDTLFLIVHADTEQSNAYEDAQQKLDQLEQLLATPVSLQARPHTPPHFESITGKAKFLETVEKVKEYIRAGDVMQVVPGQRMVSDFDGEALQVYRALRHLNPSPYLFLVQGQTITDKKPFHIVGSSPEILSRLENGIATVRPLAGTRPRGKTKEEDIALEKDLLSDEKEIAEHLMLIDLGRNDVGRVSKIGKVQVTDQMVIERYSHVMHIVSNVQGEVRDDIDALDVFKATFPAGTLSGAPKIRAMEIIDEVEPVKRGVFGGAVGYLGWHGEMDMSIAIRTCVIRDKKVYVQAGAGLVADSNPESEWNETQIKARAVIKAVELSSNGLIL

ST25 4190 cg38_0345

MARQTPITRYRNIGISAHIDAGKTTTTERILFYTGVSHKIGEVHDGAATMDWMEQEQERGITITSAATTCFWSGMGNQFPQHRINVIDTPGHVDFTIEVERSMRVLDGACMVYCAVGGVQPQSETVWRQANKYKVPRLAFVNKMDRTGANFFRVVEQMKTRLGANPVPIVVPIGAEDTFTGVVDLIEMKAIIWDEASQGMKFEYGEIPADLVDTAQEWRTNMVEAAAEASEELMDKYLEEGDLSKEDIIAGLRARTLASEIQVMLCGSAFKNKGVQRMLDAVIEFLPSPTEVKAIEGILDDKDETKASREASDEAPFSALAFKIMNDKFVGNLTFVRVYSGVLKQGDAVYNPVKSKRERIGRIVQMHANERQDIDEIRAGDIAACVGLKDVTTGDTLCDEKNIITLERMEFPDPVIQLAVEPKTKADQEKMSIALGRLAKEDPSFRVHTDEESGQTIIAGMGELHLDIIVDRMKREFGVEANIGKPMVAYRETIKKTVEQEGKFVRQTGGKGKFGHVYVRLEPLDVEAAGKEYEFAEEVVGGVVPKEFFGAVDKGIQERMKNGVLAGYPVVGVKAVLFDGSYHDVDSDELSFKMAGSYAFRDGFMKADPVLLEPIMKVEVETPEDYMGDIMGDLNRRRGMVQGMDDLPGGTKAIKAEVPLAEMFGYATQMRSMSQGRATYSMEFAKYAETPRNVAEGIIAKFQAGGKKGDDE

ST25 4190 cg38_0346

MLSLKQGAPIKLMRTNTQAGQLKYPQSPHFGREQC

ST25 4190 cg38_0347

MPRRRVVAAREILPDPKFSSQTIAKFMNHVMQDGKKSIAESIVYGALERVQEKNKVDPVEFFETTLEKVRPMVEVKARRVGGATYQVPMEVRPSRRTALAMRWLVDAAAKRSEKTMALRLAGELLDAAEGKGAAIKKREDVHRMAEANKAFSHYRF

ST25 4190 cg38_0348

MIQQFVQVSCTRRWPYYTSLELQIYEEFKQAKR

ST25 4190 cg38_0349

MATTNQLIRKGRTTLVEKSKVPALKACPQRRGVCTRVYTTTPKKPNSAMRKVCRVRLTSGFEVSSYIGGEGHNLQEHSVVLIRGGRVKDLPGVRYHTVRGSLDCAGVKDRNQSRSKYGAKRPKK

ST25 4190 cg38_0350

VAKKLSSTGLNILQQSQIGEVSKLVEPLSNGKTVEGSARNKSPFEVEQYESPQQMLREHLPKVTRQVFGRHFRKVNGIATFISPDWNEKISSYLFDWLNDFSSKSTLTEKILEEAGAKDLFELTKDTSRSQRLSQALIEQNKLIASIQGAITGVSGMVGAAVDIPVSLVLVLRTIYQTGRSHGFDLTEATDQDVVEFIFKEVDISLIAEKQTVLLALKALRNMLETQDIQQFQQVLGSSNDIETLKSWLVDENGEFKWSWLNKVPQLAAVGKFTPVAGAVLSAVYSWKLQEDVGHKAQAIFGAARHYLNEHPSEHLSPLQAYYAAVTLIQKASPRLLNVGENGSVHAAQHHKIENHDVISKVSVVVKSNTSEKSEEKVQENVHQGIEHLAEKHVVEHEHSEQKPALEPESEENDDVIEGQKYS

ST25 4190 cg38_0351

MKRVVITGMGINSCIGNSLEEVTHSLKNGISGTRFNPTYAELNFKSHVSAAAEQDFDNIDRKLKRFMGVCAMYAYNSAVAAVEHAGLKAEDLADNPRYGIAGGSGGGSTASVVEMTELLETKGARKVGPFFVPRNMTNTITANVGVAFKLQGIAHSIASACATSADAIGYAYNLIALGKQDLMLAGGGEEDHWSQSLLFDAMGALCSKYNDTPETASRPYSKDRDGFVIAGGGGFVVLESLEHAQARGANILAEVVAYAANSDGADMVAPSGEGATRCILMALEEAKQHGVDKIDYVNTHGTSTPAGDVTELKAMERAFGEGKVPPLSSTKSMTGHSLGAAGVHEAIYSVLMLQNDFIAPNINVTELDEGTEGFDIVLEKRDTKLNTVMSNSFGFGGVNACLIFKKWDA

ST25 4190 cg38_0352

MDAPSDAFLWVKALHIIAVVCWFAALFYLPRLYVYHAMSDDATSHQRFEVMERKLYRGIMWPSMIATLITAHFLVDWGDATRHYHEATWFYLKVGLVALLVIYHLVCGYYRKKLIGNAHYKSHKFWRFFNEMPTLILFAVVILVVVKPQF

ST25 4190 cg38_0353

MKSLAFIHRNDTRFAVGDFYPVLSVFSYHELGNTLSPFLLLDHLGPGKIAPSMKRRGVNDHPHRGFETVTLVYAGELEHKDSSGGGGFISAGDVQWMTAASGVIHRELFSEEYSRSGGPFEMIQLWVNLPAANKMNSPRYQSLKKAEIPIVKLENEAGFVRIIAGQFKHVMGPALTHTPITVLDVELHAGQQAAFPAQSGETALVYLRSGRAQFQKDEDVLEEQGLAVMSNQGEHFSITALQSCKLLILTGKPITEPINGHGPFVMNTYDEILQAYDDIKNGRFAK

ST25 4190 cg38_0354

MNLFTSKTMFTLNAFKLVIYRQFIIFTLFIFRHLKSYPIRFLTPEEKALAQRVFGSLLDCERPKIIATRYLPWQAHGILMAPNGNIYVNLSDYSSNYALESKFIQGIFIHELAHVMQYQRGIHVLLKGALLQSAYYLSFKYYNPYKYTYHPQKAFSSYNIEQQGEIARDICSGKLPNIICLPRLDL

ST25 4190 cg38_0355

MKSFKVALAQFSPHIGNIDSNTQKMIEQANQAKKQDADLIIFPELSVIGYPAEDLLLRPNLNKRMQKAFAQLSEVKDIVMVFGFVNQTEDGQRYNSAAVMKDGQVLGVFNKHNLPNYGVFDEKRYFQKGHQHLVFEYLGHKFGVLICEDIWSINTVKQLSQLNVDTVLVLNSSPYEVGKPQHRKQTLSELAKQLHLNIVYVNQVGGQDDLIFDGTSFVSNQNGEIALQAPSFKEDLYIAEFDRDTKLYKVVESAPALETFAEIYQGLVMATRDYVERSGFPGVILGLSGGIDSALTLAIAVDAIGAERVQAVMMPYTYTSQISVEDAAEQARRMGVTFGIAEIHSIVNSFMQTLYPFFGNSPADATEENLQARARGTLLMGLSNKFGNLVLSTGNKSELSVGYCTLYGDMVGGFAVLKDVYKTIVFELAKYRNSLSETPVIPERVITRPPSAELRPDQKDQDSLPAYDVLDAILYAYIEEDLGQADIIAKGFDKEVVEKVIRLVDRNEYKRRQGAIGPRITSRAFSRERRYPIVNGWTAND

ST25 4190 cg38_0356

MTEQKNSSKTTALAQALLLEQVEFFKKQLSIENSPIYFRQFIQLFMQHADEIKLYEVVDLEQLQAVVKRYTFEMQLGAGLLEFIGEIAQRIYLSAMKSPVQLQDLVSDHQFEMWLFKFLEMEHIPHYLNQFLRTSPSVQQLCQYIATSTLEQKLPKFLTASRVDDYHFEWQHKLKKFSFLQQQRLEHKLETWIASFIHEQLTELSLLSAEDLESLIRHIWEDIRHKKIYEFMKQLTPLDVEEFFVLIYEYWKELRQSQFMQGLILYGVEVFYDFYKDQSLFEVLSAIGLSETDLQTEALRFYPKVMDAFNEHGILEPLLQALLAPFYQSSKTLDIIEKHLSE

ST25 4190 cg39_0358

MNAKVNITNRAGASFPVRRMNFDFNDVPEYWMNGSAGLTHFMTALSALFPAGEKFFIDSVRAVRYHPAIKDNEELQKEISAFIGQEAMHTQEHVNFNASAQKFGHDVETLEKFTDTAIQTARKTFAKLVKPFGMTQEMVDLTATTALEHFTATIASQLLVNTHIQELMTDKTMSTMWYWHAIEENEHKAVAFDVYEGVFGKGVKAYALRTSSLVFAMALIFAIQSSFVVRLLKQDHKLNLDELLVIYKYGYSPSKGIITGMAKEMLAYFKPGFHPNDLDTVSLLKTWKSKLGL

ST25 4190 cg39_0359

MNAKVNISNRAGASFPVRRMDFEFAQVPRYWANYDAGLTHFMTALSALFPEGEQFFVNSTRAVRNDPKLADPALQKEISAFIGQEAMHSKEHLAFNASAQAYGYDVRKIGKTDR

ST25 4190 cg39_0360

MKPFGYTKEMIDLTGTCALEHFTSTIAAELLQNPEIQAMFQDDTMYHLWMWHAVEENEHKAVAFDVYTNMYGQGPKAYFMRSTALIIAMALIFATQSYFTAKLLKTDDKLTWKDTKYMLKFMYGRKGFMTRQIPELLDFLRPKFHPNDSDTTALLATWREKLGL

ST25 4190 cg39_0361

MIRTMQASDIDSVAQIEKLVQTHPWSRQQFVESLNSYQCTVIELNNKVVGFCILQPVLDEANLLLMAIDPQMQGKGLGYQLLDASIERLENHPVQIFLEVRESNKAAIGLYEKAGFHQIDVRRNYYPTQEGGRENAIIMVKSCTDDFASLF

ST25 4190 cg39_0362

MVFLDPIHRIDVVTLSELSRLGFRRSGDFVYRPECHLCRQCLSCRVPVTDFQMNSMQKKAWKRNQDLTMTVLPTRQASQIHYDLYERYINERHADGDMFPPSLDQFEKFLVHSCTDSFFLELWKDNRLISVSTCDLMDDGLSAVYTFFDPDEHRRSLGVYSILNQIEYVKTLGLEYVYLGYWVPHSAKMNYKSQYTPLELLLDGQWRRLNRSLSPEEINQLGNSLMTTLPSEWNNLIIK

ST25 4190 cg39_0363

MKTLPLSQYIFPDPEESDPDGHGLICIGADLSPSTLYEAYTHGLFPWFNEDEPICWWSPEPRCIIYPQNYKPSKSLIRNMKKYDYTITVNRAFEQVIRSCSLPRSYANETWISEDIIEGYCGMFDAGYGYSVEVWQEEQLVGGLYGVTIGKGCFGESMFSTQTDVSKMAFYTLMLIGQENQLPWVDCQLVNSHLISLGACTLSRQEYLKSLQDVIIHPSINWKKYQERVFSSKTIALNAKLME

ST25 4190 cg39_0364

MSARHSRLIILGSGPAGYSAAVYAARANLKPTLIAGLQLGGQLTTTTEVDNWPGDPEGLTGPVLMDRMQAHAERFGTELVYDHINEVDLNVRPFVLKGDMDEYTCDALIIATGATAQYLGLESEQKFMGQGVSACATCDGFFYKNQNVMVVGGGNTAVEEALYLSNIAEHVTLVHRRDSLRSEKILQDHLFAKEKEGKISIVWNHEVEEVLGDNTGVTGVRLKSTKDDSKQEVQVQGLFIAIGHKPNTSMFEGQLNLRDGYIQVQSGTSGNATATSVAGVFAAGDVADSIYRQAITSAGSGCMAALDAEKYLDNL

ST25 4190 cg39_0365

VSSVYAQRLLMTLFLVSFGIYMFLATVTYTPFDPGWMHISSDTQQVSNASGIAGAWIADLLFGFLGWASLLIPIFLFVEAIQVWWPHSFLNRPFRYAAQFFLILVVSSLLYLHWNVPADTLDNAAGGIIGYELGQSLSQLLTIYGATLFLLVFGVVLFTLAFGVQWSKTWVTLKAMPGYLQDLFYKNVSPNESAYDLTTQPANKAATVKVAEMPKAEEVTDEVELNAQSEKAQSKASSTRHDEIAERLFADVLAKEQSQPETIEEKPTPQPENFERTLEQAQQLKKDSERLVATGEVWRALQRDDVSHKQEIDALLRAADDSSEQPSTHEQFQQTVYQAKQNQLSNTHQGLDWNDDEIFDELLAAVPNSKTATDVHTPFVQDHHVEPEVTPQAPQTLVESSVPISNLNQSPKNLSNEEVFDDFDDLLIDEDIPPATQPIKASSYAQSSAFVKAPIQTTIGAEKLSKEEFIEAWQETAGKPQDDLEDEFDFDAPLTDASGRPMSRAMQVAKKRLDLPTLPGFELLDKVDPNKKVNFTEEQLSRLSELLEIKLQEFNVKAQVVEAQPGPVVTRFELDLAPGVKASKVTNISRDLARSMSMASVRVVEVIPGKPYIGIEVPNSAREMVRLIELLETPAYRDPSALISMAMGKDISGNPVLTDLAKAPHMLVAGTTGSGKSVAVNSMILSMLLKYTPDQLRLILIDPKQLELANYNDIPHLLTPVVTDMKDAVSALNWCVNEMERRYKLMSFLKIRKLSDYNRKVEEALANGEDLIDPTWKPSDSATQERAPRLTPLPSIVIVADEFADMIMQVGKKAEEMITRLAQKSRAAGIHLLLATQRPSVDVITGLIKANIPTRVALRVNSKIDSRTILDAGGAEDLLGHGDMLFLGPGKIEPERVHGAFISDDEVNRICDAWRERGEPDYVDEILTPFDEEPASRGFEEGDGGSDRDALYDQCVSFVLETRKASTSSLQRKFSLGYNRAARIIDQMEENGIVSAMGANGKRDILV

ST25 4190 cg39_0366

MLVALITLAFIHFCALITPGPDFFLVSQTAVSRSRKEAMLVVAGITAGVMFWASLALMGLNIIFEKMAWLKQGLLIAGGLYLCWLGYQMLRSAFSKNQQEEVTTVELPKAPYLFFMKGLLTNLSNPKAVIYFGSVFSLFLANPQLDQVHWLLFIIVSVETILWFCFVTFIFSLPSFRAAYRNFSKWIDGISGGIFTVFGIFLIGNR

ST25 4190 cg39_0367

MALSQPATFNEEWSDERVFAYLNQLPPNGVNADFHVLYHAFKHMRPNDYERLLTKFVADGRDINATNPEGERIHDVIAKFPRQSAPFLEVLAKFA

ST25 4190 cg39_0368

MAGFWSKLFSSEEKPSSAQTAKDRLKVIVASEQGLGRRLSQDKIDQMKKEIMQVVSRYVSGVGEQHIQMQVRSEANIEMLEMNINLPEER

ST25 4190 cg39_0369

MLTIDDISKDILRVPTLGVIPECPSVLQASNEGKPVILYSETKAGQAYDDLVARFLGEDRPYRHITAQPKGWLARLFGA

ST25 4190 cg39_0370

VAKIVVVTSGKGGVGKTTTSASFATGLALRGHKTVVIDFDVGLRNLDLIMGCERRVVYDFVNVINNEARLQQALIRDKDIENLYILPASQTRDKDALSDEGVARVIDELSQEFDYIICDSPAGIERGAILAMYHADEAIIVTNPEISSVRDSDRIIGMLDSKTKKLNTTKDVYVNIYVLHVLTLNVLTVRKC

ST25 4190 cg39_0371

VIRQQLSNILNEGSYQGTVVIIDSTVEQELIALIQLLVSMGLQPMAVIDGILGDEARAIQFPVLPADQPLQRIKPTAEQVAIVEKPTSAQASVETKKPLNNNAVAHITSYHDEILRTGQSLVQDQGDIILKAGMNSGSEVIASGNIHIYGTVRGRVIAGAGGHAAARIFCQSLEAELVSIAGTYCVADDIPKHVVKKPVHIYLNEKQELEFEALEL

ST25 4190 cg39_0372

MVFIIIFNLFCLAANFFLMSSIGAWFFEHIHLPQVLSFYRTHLHPWVITSEAWFIGFLIIELLVRWGIAIVYKHHKRWFFFPFIHWYEILAIIPQLRFLRLFRAGIIAYRLHELGYPVIPESWRKTGLFYYRVVMEELSDRVVITVIDGIRYELETSSSHKQIIHDLVNHHREQFTVTLTALLQESLATALKEQKPVITRAVGKIVDQAIEDTPELTQLLRLIPLVGGRIEQQIQSIGQRLGENISAGLIEPLIEGSAAHPNSTYQLISQKISHINIDNHELEQLVESVVFETLEAVRKQVKVKQWQQTLAVYDRIKE

ST25 4190 cg39_0373

MSTAPNKQSALKKITRGLTVGTVITGSTFFHGPPVLALGLTKLFKKSSKIDETNIQITNSWLGVNNWLIDHVLPNLKWDISIDEGLDLNMQGRYLMTCNHQSWVDTTVNQYFGLTRMPLTRFFTKWELIFIPFVGQAFKILGFPMMKRHTKEQIAKNPELKDRDMMEARKACEQLLSQPFTLLNYLEGTRFTQEKHDQQKSPYKHLLKPKAGGLALALNILGDKIDALVDMTIVYPDGVPGYGDFWLGDVSKIAVNLRKIEIPDWVLGGNYEDDPVYRERFQQWVHELWTEKDQLIEKMKAQYVTTNL

ST25 4190 cg40_0374

METILGLCIGVGLSAACGFRVFVPLLVMSIATMMGWFEPSKGFEWLALPSVCLALGVATVCEVAAYYIPWVDNALDTVATPAAMIAGTLATMAVSSGEMSQFASWAAAIIVGGGTAGVVQMSTVAARGVSTATTGGLGNFIVATGEWIGAILLSVSAMLVPALVAIVVLIAVIWVVRWIRHKKQEQAHTPL

ST25 4190 cg41_0375

MTVMLVKPHLKFKESYNNYINELADEDRHPLTTEFDHTDFESFLNKLRDYEKGESLEEGHVPNITYWLVEDNEVIGASNLRLKLNEQIEYCGGHIGLGIRPSKRGQNFGSKLLELTIQEAWKLGLTELYIHCYKSNLASAKTIQANNGRLHSEITLDQVVQRYVVYKNPIKIFF

ST25 4190 cg42_0377

MNEKQTRCPKCLTVYKVSLTQLTVAQGMVCCPKCNLSFNALTNLVNVEQPETVNSSALAHGSMQTRYMTHSHVLDIFDQKVENSNIDLLTYLNNLNYFHNEPINALPNLNLAEQATSPEQDNGKKHSWLYYTLWSSANLILILVFTFQILWFNPKLMQQSPAISHAFNYVCQILHCNKSAEQYQLLYIEKVKLRKIGTEHTQLSGVLINQNNKSIELPNLKLSFGKETDQEEIIFTPQQYLVENLRGIQRIPTNTPFRFQVELNKSKKSLVNYRLEIVHP

ST25 4190 cg42_0378

VKWLQIHITVDQEQVEFTETLLMSLGAVSVTLDDAEDQALLEPLPGETPLWNKVIVTGIYQQDEQDPIDVDTLEAFLKAQLPDVPMRHEELEDQVWERAWMDYYEPIQIGEKFWIVPEWLEPPEADATNIKLDPGLAFGTGNHASTFLCLQWLGKTDVKDKIVIDYGCGSGILGVAALLLGAKKVYATDIDPQAVLATKQNAELNGVLDRLYVGLPEEFDQEFKPQQADVLVANILAGPLMALAPEFAKLLKSDGDFALAGVIEEQVADVSGVYSEFFDILDVEKREENWCRISGKRKTTN

ST25 4190 cg42_0379

MKTTMLKILTASLICFSGVTFTHAADTSKAETQKQIRVCAKKQQGEWVSYNHNGVTFNGTCQPNENGKLQFKAPAP

ST25 4190 cg42_0380

MHYPKVYDVIVIGGGHAGTEAALAAARMGRQTLLLTHNIETLGQMSCNPAIGGIGKSHLVREIDALGGAMALAADKGGIQFRILNSRKGAAVRATRAQADRVRYKAAIRETLENQANLDIFQQAADDLIVEGDTVKGVVTQMGIRFDAKTVVLTTGTFLGGVIHVGLEKSSGGRAGDPPSIALAQRLRELKLPVGRLKTGTPPRIDARSVDFSVMTPQPGDFPSPVMSFMGDVSMHPEQVNCYITHTNEKTHDIIRGGLDRSPMYTGVIEGVGPRYCPSIEDKIHRFSDKDSHQVFLEPEGLDTHELYPNGISTSLPFDVQFELVRSIRGMENAHILRPGYAIEYDYFNPQALKFTLETKAINGLYFAGQINGTTGYEEAGAQGLLAGLNAARRAWEQEEWTPKRDQAYMGVLVDDLITLGTKEPYRMFTSRAEYRLMLREDNADQRLTTIGRELGLVDDVRWAAYCEKMEAVERETSRLQHLWAAPNNPMGKKFVEMTGADLSKECSAIDLLKRPNINFGQIAELTGSDVSQQVGEQIEIAVKYEGYINRQHEDVAQLKRLEETKIPADFDYDVVSGLSREITQKLKTVRPETLAQASRIPGVTPAAVQLVMITIRKNNMTKKRLNYLSV

ST25 4190 cg42_0381

MSVTEKETSNSLYRQWQILSRLPTGKWIGTRELQEMLEREGIEISLRTIQRDLNQIAQRFPIESNKAVPQGWRWQSDAPIQSLPHMTSSQAVTFMMVEEHLKHLLPPSLLDEMGPWFDLAKRSLSTQNNVRQWINRVRIVPASQPLIPPVVDRAAQQAIYEGLLQDKQIECVYRARVNQGEDKTYILNPLALVQKGAVIYLICTRHDKTEVQTFALHRFKSAKVLESRALHPVNFDIDHYIDSGALGFRVDYNQPTESIQLTLTMTEQTAKTFYESQLSKDQTVTPIEENIVEVTATVPFTSQLVWWLRSFGKKLLHIEPVQVHNAVREIEPDSK

ST25 4190 cg43_0383

MPSFQSADAQINYQTFGEPSSPALVFSNSLGTNYGMWQKQFNELKDQFFVICYDTRGHGSSSTPNGPYTVEQLGEDVVRLLDHLNISKAAFCGISMGGLTGQWLAIYYPNRFSHVIVANTAAKIGQEQAWLDRAKLVREQGLQPIAATAASRWFTDPFIQSHPSIVNNLCNDLSAGSAIGYANCCEALAKADVREQLKDIKIPVLVVAGTQDPVTTVADSQFMQQRIPQSKLAEIDASHISNVEQPEVFNKILTDFLAD

ST25 4190 cg43_0384

MSEVKILQSVQQFMTRQHGHFIDGKLATAEHLDKVDIVNPSTEQVVAQISIGSQQDVESAVKSAEHAFQNAWAETTPYERGVKLNKLADLIEQYGEELAQLETLSTGKLINISRHLEVAQSVIFLRYFAGWATKINGQTMQPSIPSMQGEKYTAFTLRQPVGVVAGIVPWNFSLMIGVWKIGSALTTGCTIVLKPSEFASLSLLRLAELAIEAGIPAGVINVVTGKGDTGQYLIESPLVKKVSFTGSVPTGIAIGKLAMNSDLTRVSLELGGKNAIAVLADANIDEILPTLLQATFVHQGQVCASPERFFVHRTKYDELVEKLSKALSSFKIGSAMDERSMFGPLSNQPHFHKVKHYLDMAKANNQIIAGGEALDQTGYFVQPTLISFKNTDDPLFSEETFGPVVGVMSFDTDEELVQLMNQSRFGLTASIWTNDLSKALRLIPKIEAGTLWVNMHTFLDPSVPFGGVKASGIGREFSDAFIEDYTELKSVMIRY

ST25 4190 cg43_0385

VHAGELEIKPTFEATFGAFSSGKSYNGENLHDERINWQEGHLKYGAKGQYTFKNNQLYASLSGISSATFGDGDAGQNSNGKERKTDVNEWVIGFKDGTNKDELTTYDLSIGRQNIMIGDGFFIAGDALNLGKAPADGTLDRGGAYYLAARRSFDFTSLLQYKPQENTTLKLAYLKSDNKAQFSPELFVTDLMYQLKDKGVGFTYLNVLDLTDAEQVSGRQDLKNYALRLNYQPLPEIQVKTEFVVQDKKDGQENAGYLALNYNFLSSKYTPSLGYRFSHFSDQYDPMFYGNTVGFGTWFQGEVAGNYAGPFNKNADIHQLSYMMNLKENFMLGALAYKFKTVNKSLTNIDGYELDVFSVWSVNKKFNVIPLVGLYKPKHDIHSGGTQNYDDKTNVYAQLILQYIY

ST25 4190 cg43_0386

MKPMRKQRLLNDIQKILALNLSIAASFWGLNTQAFAHGGKADMVSLYQNMNEQGAKVVKDDFTNTYTITKGSIVVRAKPNSNQVLVNGKPLKLSVPLLIKDGKPVIGKDFFNEVFQSGLDQTFKIENTFHPLNSLNSDEIKKTFDVINRSKYAYKNMRFAELKLKEPEKAKVWDYFINHKDFKEDRIASFILLKGNQAIEGEVNLNTQQVTKWNVLKDTHGMVLLDNFEAVQRVIETSKEYQDALRKRGITDVKKVIATPLTVGYFGGKDGLDKQLNILKVVAYLDVGDGNYWAHPIENLVAVVDLDKEKIIKIEEGAIIPVPMANRPYMSNKPVASKLKPLNITEPEGKNFSITGQTIHWGNWCLHVALDSRVGLQLSTVTYKDKGVKRKVMYEGNLGGMVVPYGDPDIGWYFKSYLDSGEYGMGTLTSSILPGTDAPDNAVLLDAVIADYKGQPQVIPNAIAVFERYAGPEYKHHEIFGNQDASEARRELVVRWISTVGNYDYMFDWVFSQNGVIGINAGATGIEAVKGVKARTMHDSTAKEDTKYGTLIDHNIVGTTHQHIYNFRLDMDVDGENNSFMHMDPVVKANDKGGVRTSSMQIDSKVITNEQNAAEKFDPSTIRLLTNFNKENKLGNPVSYQLIPFAGGTHPVAKGANFSKDEWLFKRLNFMDKQIWVTQYNPDERYPEGKYSNRSTHDTGLGQFIGNNENIENKDLVVWMTTGTTHVARAEEWPIMPTEWVYTLIKPWNFFDNTPTLNLGEEEQSTQHDHH

ST25 4190 cg43_0387

MSGFLLKNILKNMSAENIKLWYATEDGNAFEDALIALIKPTINYKNINSSNNLMEKAERFIIENLAKPDLTPKLIAEHIGVSLRHLYRLFLQENLSINKYIQLKRLEKVKADLLDKKNKQNSIAQIALKWGFWDGAHFSKIFKKTYGISPKEFREGMSV

ST25 4190 cg43_0388

MYPQGLVSQISVHLSREKFLKKILVPNILEN

ST25 4190 cg43_0389

MNQYVSDALLTWTHHIKNVCGNFETDFDGTRNLFIGEVQCFLLGDTEIAFIKNNANKIVRKADEIDRVNNRFCFFDFAVFGKNAARV

ST25 4190 cg43_0390

MMNVLQQIDEFTVDPLQFRRALGNFATGVTIVTAQNAQGEKVGVTANSFNSVSLDPPLILWSIDKKSSSFSVFEEATHFAVNILSGTQIELSNKFSRRNIDKFADTNFQLGAGRAPILENCSAVFECERYQVIEGGDHWIIIGKVVRFHDQGRSPLVYHQGAYSCVMPHPSLQVKQTEQSGVDQTHYGHLYNNVCYLMSRAFKAYQTDYIPKQMASGFRTSESRLLLVLASGTASSKEDLPRDIAMPMQEVERSAEILKFEGLLVDHDNLYALTEKGKQTAQYLFDIADSHQNEVFKKYSEEQKNIFITMLRDFAGVA

ST25 4190 cg43_0391

MDWVINLATTHDFPEQQIHKEYFQVETDTSGDSFEVVAERSGKIIMVEAGETILQALAKEGIEIEMSCEQGVCGTCMCDVIEGEPDHRDVYFTDEEKASNEQILVCCSRSKTPRLVLDI

ST25 4190 cg43_0392

MTTLYDVVVKNRHVEGGNIAVMEFESATSAALPKVEPGAHIDVHLPNGMVRQYSLCQNPNDEGKFRLGILRDPESRGGSISAFDEIRDGMQIQVSEPKNLFPLLKAKHSVLIGGGIGITPLITMAYQLAHEGASFELHYCGASPEKCAFVDEIKNGVLAQYTTFHFKSEGASHRAIF

ST25 4190 cg43_0393

MKQVVLVTGAASGLGNVIAEYFASQGHQVILSASTLEKAENAKAQSQYAQNMFPLKLDISVEADFHAAVQWIEEKFSKLDVLINNATVTKATPVLEITAADFDWVTQVNQRGTFQACQIIGQYMAKKGYGRIINMASLAGQNGGTATGAHYAATKGAIVTLTKIFAKEFAAKGVTVNAIAPGPMESPIVHSVVPDEKMEQFIQNIPVKALGSMHFIAETCGLLASPNASFVTGTTWDINGGLFMR

ST25 4190 cg43_0394

MNMNLQLLNEVTAFIWAEADMLDHSEYREWLNLWSEKGIYIIPIDPNLTDYENNLNYAYDDNHMRKLRVERLENGEAISTAPKANTVRSISRVRVIQDQNDEIVLRCAQNLREFRKENLKHYTADITYHLTRDADTGFKINRKIINLVNSTDTLAGISYIL

ST25 4190 cg43_0395

VPSPESYGDCLDKSELPLVSLRVEEYNGMIFASFKEDIQPLEEFLGPAKKWIDLFMKQGAGYPIKVLGEHRFRFPGNWKIQLENTTDAYHFPLVHKSFLSSVDEKTEELFNFENQPGFVEDLGNGHSVMVMIPELVDLEEDLMERPIQERFEDLAQALRDEGHEELEVRRIVRAVGGSGFNLNLFPNIACSMAFFRVLQPISVAETEIHHSVITMDGGPQIANQYRLRLHEHFQGPFGFGTPDDSEAWERVQHGANAGNDLWIMLNRGLPGEVKTEDGLKSDVSAETGMRAAYQQWKKMMTA

ST25 4190 cg43_0396

MNSIIPTHNIGIDYDALVKSDRAHTSLYKDERIFDEEMEKIFYSTWVWVAHTSEIPEGGSYKTINIGKQPVVVVRDRKRKSMYF

ST25 4190 cg43_0397

MTDKVNIAEIIERLEQLEAHNAIRNCINRYMEICDELNANTDLYELMNLFDQDCIWEGIGEKYAKSFGRYDSWQSIYDMFKRYTQKESHFVMNAHFVNSEQIYVNQNDANASWLMLQTSTFQDGRSHLNAAKLKVKFQKQADGTWKIKHFQTQNIFSRPVSHWHSEAELPVPSQE

ST25 4190 cg43_0398

MWNGIHDLPGVHVFGNLDDDQSMVGASTGWPWTTYLLADVPDIETVHAACNLFRTTVVGEGPYKLWKYCKVEARVGRELIIQR

ST25 4190 cg44_0399

MIDKSKSSLSEVLSQIKDGATILIGGFGTAGQPAELIDGLIELGVKDLTIVSNNAGNGDYGLAKLLKAGSIKKVICSFPRQSDSYVFDELYRAGKVELELVPQGNLACRIQAAGMGLGAVFTPTGFGTLLAEGKETRHINGKDYVLEYPIKADFALIKAYKGDRWGNLVYRKSARNFGPIMAMAADVTIAQVSEVVELGGLDPEHIITPGIFVQHVVQVQPAQ

ST25 4190 cg44_0401

MSYQKLSRDQIAQRVAQDIPDGAYVNLGIGLPTKIASYLPADKDVFLHSENGLLAFGPPPAAGEEDPELINAGKEFVTMLEGGSFFHHGDSFAMMRGGHLDIAVLGAFQVAANGDLANWHTGAPDAIPAVGGAMDLAVGAKKVFITTDHVTKQGEPKIVAELTYPVTGKHCVDRIYTDLCVIDVTKDGLKVIEKVEGLSFDELQALTGATLIDATQG

ST25 4190 cg46_0402

VVKADGTVTAGNASGINDGAAALLIASDDAVQAYNLKPRAKIIASTAVGVEPRIMGFAPAPAIKKLLKQANLTLEQMDVIELNEAFAAQALAVTRDLGLPDNSDKVNPNGGAIALGHPLGASGARLVTTALNQLEQTGGRYALCSMCIGVGQGIALIIERV

ST25 4190 cg46_0403

MSQLYASLFYQKDVTEIFSDSSLITYMIQVEVALAQAQAQVGVIPQNAANTIAQVAEQALEKFDFSALAVATGLAGNIAIPFVKQLTAIVKDTDEDASRYMHWGATSQDILDTACILQCRDALKIVEGQLQQCYTTALQQAKQYRHQVMIGRTWLQQALPITLGHKLARWASAFKRDLDRIQAMKSRVLTAQLGGAVGSLASLQDQGSLVVSAFAKQLNLTVPTSTWHGERDRIVEIASVLGMIVGNTGKMARDWSLMMQTEIAELFEPTAKGRGGSSTMPHKRNPVAAASVLAAANRVPALMSSIYQSMVQEHERSLGAWHAEWLAVPEIFQLCAGALSRTGEVLQGFEVNAEHMQQNLECTNGLIMAEAVMMALAPKIGRLNAHHLVEAACKTAVAQNEHLFDVVSQLDEIKGQFSQEEIRNIFKPENYLGNIQQQIDAVLKEAQGESK

ST25 4190 cg46_0404

VKQTISNRQGKQLAVYTDGLKDAPVLMFSNSLGTDHGMWQPQLNELKSHFNVITYDTRGHGESDVISDTTLQNLAEDVVDILDALNIEKAHFCGISMGGITGLWLGIHHPNRFNSITVANSAAKIGQTEAWLSRAESVEKNGLAELVKTTHTRWFSEKFDYQHNVVAQTTIQSLANTPAQGYANACRALAYADLRDEIAQIQIPALLIAGTEDPVTTVADAEFMQKAINNSQLAKLEASHLSNIEQPQRFTQELTRFIQQI

ST25 4190 cg46_0405

MASQDYAAPSKSIDAQALINDAPLSKYQWMIAIICFLIIFTDGIDTAAMGFIAPALAQDWGVDRSQLGPVMSAALGGMIIGALVSGPTADRFGRKIVLAFSMLVFGGFTLASAYATNLDSLVVLRFLTGIGLGAAMPNATTLFSEYCPTRIRSLLVTCMFCGYNLGMATGGFISSWLIPTYGWHSLFLLGGWSPLILMILVILVLPESYRFLIVKGKNPEKVRKILNHIAPAQIQNATAFHVPEEKTEVVQKKNVFGIMFSKPYAKGTVLLWVTYFMGLVVVYLLTSWLPTLMRETGASMERAAFIGGLFQFGGVVSALFIGWAMDKFNPNRVIAIFYFAAGLFAIAVGQSLGNSTLLAVLVLCAGIAINGAQSSMPALSARFYPTQCRATGVSWMTGIGRFGAVFGAWIGAVLLGNDWSFTAILSLLLIPATAAAVAVFIKSIVAHTDAT

ST25 4190 cg46_0406

MNDEQRYKQGIEVRTEVLGEKHVGRSLQNLNDFNQDFQNFISRYAWGEVWSRPGLPRHTRSLVTIAILLALGREDELRMHLRACFNNGVTKEDLKELILHSSLYAGLPAANAAMHMAEEVFKELGIEVNPIAAKEQD

ST25 4190 cg46_0407

MSQHSWGAYAQRNTDDHPPAYTPGYKTSVLRSPKNALISINETLTEVTAPHFSSNLFGPKDNDLILNYAKDGLPIGERVIVHGYVRDQFGRPVKNALLEVWQANASGRYRHPNDKFIGAMDPNFGGCGRTLTDENGFYIFRTIKPGPYPWRNRINEWRPAHIHFSLIADGWAQRLISQFYFEGDTLIDTCPILKTIPSEDQRRALIALEDKNNFIEADSRCYRFDITLRGRRATYFENDLT

ST25 4190 cg46_0408

MNNWNFQELKETPSQTGGPYVHIGLLPQQAGIEVFENNFNNQLVKDQTKGERIRLEGQVFDGLGLPLRDVLIEIWQADANGIYPSQADTREQKADPAFQGWGRTGADFETGVWSFNTIKPGATAGRKGSTQAPHIALVIFARGINLGLHTRVYFEDEAEANANDPILNSIEWAPRRQTLIAKRFEENGEVVYRFDIRIQGDDETVFFDI

ST25 4190 cg46_0409

MNDMNMKTLLSLSLLALPFFTAQVNAEPVALAAQTQPAATAVKTIVPITAKTKEQALAQAQVIANTADADLAEFRIDLLSFASDTKQVIALGHELKKILGNKPMIATIRTKNEGGQLEISDADYGKTYQAYLKNPFMDWLDVEMFRDQKVVSEIVQKAHQKKVLVVMSNHDFQKTPSQDEIEKRLLKQDQMGADVLKIAVMPKSKQDVFTLMNATLKVSQQTTKPLLTMSMGQLGTISRVATANMGGSYSFGMIGQASAPGQIDVTKLKQILQTVQPTNP

ST25 4190 cg46_0410

MNTLRLITLALGLMVSGIAAAQTYVVDRYQDDSNKGSLRWAIEQANANPSEASDILIQAVGKAPYAIKLNSALPEIKAPVKIIGTQWDKTGEYIAIDGSNYIKGEGAKACPGANPEQYGTNVRTMTLPGLVLRDVNNVTLKGLDIHRFCIGVLINRSSNNLIQHNRISNNYGGAGVMLTGDDGKGNPTATTTNNNKVLDNIFQDNGDGLELTRGAAFNLIANNHFVSTKANPEPSQGIEILWGNDNAVVGNKFENYSDGLQINWGKRNYIAYNEMTNNSIGFNMTGDGNILDSNKVHGNRIGVAIRSEKDANARITLTKNLIWDNGKDIKRCEAGGSCVPDQRLGAIVFGVPALEHEGFVGSRGGGVVIEPSKLQKTCTQPNQQGCNAQPNQGIKAPKLTANKGSVTVEVNGLPNQRYQVEFFGNQNTVSKEAEQYLGTITVATDTEGKAKANWKPTVKVASITANVTDRFGATSELGFVQVK

ST25 4190 cg46_0411

MPNVMKLSMLTIAILGSQFALANEPWSQDRQWLLGDWNGKRQQLEQQGYKFTASIMSQAATNLEGGYNDSNTFENAAQLSLGANFDLEKIAGWKDTTASLVVTKRDGNALTLERIKDPRSSQLGNAQEIYGRGKIWRLSQAWVKKGFVDNTVQVKFGRMGMSEDFNSSQCEFQNLLLCGGQLGKSIGSIWYNSPVGVWAANVKYQFAPEWTLGVGVYEVNPDNIKTESNSDGFNLDMNNVKGATIPVELAWKPKLAAFNGLPGEYKVGALYSTAEANDIKTAGKVHDGKQSFWINAQQQLSHAGQDPKRGLYVSFNGVVNDKATTFVQSTQQLALWYKGPFDSRPNDSIGFGIANYVVNDRAKDKQIATNESRGYYSYDPIASDYIPIQDDELNVELNYTYQWSPAVMLRPNIQYIHQPAGVKEVDDAWVAGLSVKVNF

ST25 4190 cg46_0412

MSESDSKFHILFKIWCFILGLALLATGIFYIVGGGKLVSLGGSWYFLISGLFITISAISIFRKKALGVWIFAAVFVGTVIWSLIDAGWEFWPLFSRLMFPAGLFAALLFTLPSIRRYQFQTSLASSAYAVGGLVVVGMLIALYQMFQPHPTVASSGEKLPLVPVDPAKKQVNWENYGNDAGGSRFVALDQINRDNVHKLKEAWRFRTGDFTTGSGNGAEDQMTPLQVGNKVFLCTPHNNIFAIDADSGKQIWKAEVNSKADAWERCRGVAYFDSTKPLVQPTLAGATPVTTVANNTACPRRVYTNTPDGRLIAVNADNGQRCADFGVNGTVDLLEGLGGGTKAPRFEVTSAPTIAGTTIVVGSRIADNVAADMPGGVIRGYDVITGKLRWAFDPRNPDPNYVLKPGETYKRSSANSWAAMSYDPQMNTVFLPMGSSSVDIWGGNRNPLDHKYNTSVLALDATTGKEKWVYQTVHNDLWDFDLPMQPSLVDFPLKDGTTKPAVVIGTKSGQFFVLDRITGKPLTKVIEQPVKAANIPGEQYSLTQPRSVEMPQIGNQTLTESDMWGATPFDQLMCRINFKSMRYEGLFTAPGTDVSLSFPGSLGGMNWGSIAFDPTHRYMFVNDMRLGLWVQLIKQTPEDIKIQASGGEKVNTGMGAVPMKGTPYKVNKNRFMSALSIPCQKPPFGTMTAIDMKTRQIAWQVPLGTVEDTGPLGIKMGLKAPIGMPTIGGPMATQGGLVFFAATQDYYLRAFDSSTGKELWKSRMPVGSQGTPISYVSPKTGKQYVVVTAGGARQSPDHGDYVIAYSLEK

ST25 4190 cg46_0413

MRFLVLATLSGLALMGCASVQQAPNTQETIRYASSPCFGACPIYSVEVTPTGLIRFEGKQYTKAIGVKEIQGSVKDYQKLADELKTYRPQTGTQSKTAGCQQTATDMPSYYITWIQPNGTETKLSHYTGCISPANNRLNKVMEKLPEQLGIKDLI

ST25 4190 cg46_0414

MSSIVYAFVGGLLLGIATVGYLYINGRIAGISGLLAQFISPSRDIFKSSAFWFIAGLVITPFIYGYFYQPEIEIKANSFVLILAGLLVGFGTRLGSGCTSGHGICGMSRLSKRSIIATAVFMFAGMLTVYTIRHVLG

ST25 4190 cg46_0415

MKNIFAFIFGGLFSLGLMISGMSNPAKVLGFLDIFGQWDISLMFVMLGAIAVAFIPFQRAIKKPKTIFNEQIQLPTNTQIDQRLIVGAFIFGIGWGIAGICPAPALTLIGLGHFEALYFIVAMLVGMFIYRILNKGN

ST25 4190 cg46_0416

MQQPLVKDFFDENTNTFSYVVADLATRQCAIIDSVLDYDAASATTKTTNADLIVDYVLAQNFKVQWILETHVHADHMTAAQYLKSKLGGTIAISQKISVVQETFSAIYNFDFKKFNENQPFDYLFEDYEHFKIGEIDAYNIPTPGHTPACLSYVIGDAVFVGDTLFMPDYGSARCDFPKGSAAALYDSVQKLYTLPDDMRMFLCHDYKPEGRDEYICQTDIKTQKQSNIHLNRRVSKESFIKMRQERDATLAMPKLILPSIQINMNGGNFPEPEVNGIRYLKIPFNYF

ST25 4190 cg46_0417

MSKDERIIQNQDNKKTIRHEDYIAGIGKGMAILDSFSSNQHRLNISMAAEKTGLTRAAARRHLLTLEYLGYLESDGHYYYLTPKVLKFSGAYLGAAQLPKVSQPLLNLLTNQTSLIYSVMVLDGYEAITIARSAAHQQTDRVNPYGLHLGNRLPAHATSAGKILLAHLSQEEQLDWLQKYPLQRLTKYTHVENDKFLELLQEIREQDWCYSREEHELGVHALAVPIYGQDFKVVAALNIVSPTMKTTEEYLVQHILPLLQETSRDLRQVL

ST25 4190 cg46_0418

MNNIHNLHGLDLSFFHRIDINLYPLFIAIYEQKSISNAASSLNITQSAASHALQRLRQQLEDDVFIRVGNKMSATPFAEQIYPTVKQALLTIQNISQQKHVFDPTSLKSLKIAVHDEIEPIILPRLAQHFQNLNLDLQFVSMKLDRKHIMADLATQQLDFVIDLEQYLSPKITFDHLVTDEFVICSPLKEVDLSRYINGRHIGVSSRRTGALLEDIYLNQHQKISRNVFMRCQNYSTALQVLGAQADAILTMPRMILQHLSYSPKIRLHSLPFEMTNIKMGMFWHEDLRDNLRHQFLRHEIIKLFQ

ST25 4190 cg46_0419

MFELSKKAQDFTERTRKFILEEIEPVEAKFWEEVHELNPDGNWKKWQWPELLETLKSKAKQAGLWNMFLPDEKLGAGLTVQEYAHIAELTGRSLLAPTVFNCNAPDTGNMELLWRYGSEQQKQQWLEPLLDGKIRSVFCMTEPDVASSDATNMQATALIDGNEIVLNGKKWWSSGLGDPNAKVIIFMAHTPDETKDRHHQHSMVLVPIDTAGVKIQRMLPVFGDYDAPHGHGEVHFNNVRVPLENFIGGAGQGFEIAQGRLGPGRIHHCMRCIGAAEKALELMIDRGMSRTAFGKEILKLGGNLERVADARVAIDQARLLTLYAAYKMDTLGNMVALTEISAIKVVAPSVLEKVVDMAIQLHGGAGVSRDTPLTGFFAQARSLRLADGPDEVHKGMIAKLELAKRGYFGRHKKV

ST25 4190 cg46_0420

MSVIDVGGEVREGEELDIGAVENWLKNQGVELVGPAVVTQYTGGASNWTYRLKYENTDLILRRPPKGTKAKSAHDMAREYMVQKNLAPYYPVLPKMVALCQDESVIGCDFYVMERIEGIIPRAKLPPELGFTEENVHELCVNVIDKLIELHQVPYENTPLAELGKGAGYCRRQVEGWDKRYEKVRTINVPSFKYVRKWLNDNIPQDSTTCIIHNDWRFDNVILDPEHPTEVIGVLDWEMATLGDPLMDLGSALAYWVEPTDNMIFRSTRRQPTHLKGMFSRKEVVEYYLQKTGLEPETGLFMKSSVYSV

ST25 4190 cg46_0421

VIAQQIYYRYYHKQTRNPAFKDFWIVIHALHIRALKLIAQQKLQESEFAQQSLQKIQGILRR

ST25 4190 cg46_0422

MTTIYLVRHGQASFGKSNYDELSENGEAQATLLGRYFKQILKEQPYVVAGTMQRHEQTAKLALNECFPDAVIHHNNLWNEFNHQQVFARYEPRFEQPELLKADVAQEQNPRAYLAKIFEGAIGRWTDGDFHHEYDESWPHFKERVETALQQLCDELAKTKPRYAIVFTSGGVISVAIGKLLELSPNRTFALNWAIANTSLTTLRLVGNEAQVLSLNEHHFIKAERPDLLTWI

ST25 4190 cg46_0423

MAKTILITGASSGLGAGMAHEFAAKGYNLAICARRLDRLETLKTELENQYGIKVIAKSLDVTNYDQVFEVFRAFKQEFGYLDRIIVNAGVGNGRRIGKGNFEINRATAETNFISALAQCEAAVEIFREQNAGHLVVMSSMSAMRGLPKHLSTYAASKAAVAHLAEGIRAELLDTPIKVSTIFPGYIRTEMNEGAKHLPFEVDAKTGCKALVKAIEKQPVKAYVPQWPWLPMSIAMKVLPLRLVNKLG

ST25 4190 cg46_0424

MELFHRFKPHTLAFATLFIMCSSSWAANPNQQTEDEWKFTLKNAYINRDFDNDALKDTGSWSQAASLFYKSKMHDTPLVIADKPITIGADASVQYAVRLSSDKHVADTVLPFNKETQSQASDYLKYGATLKLGYDKTLLSVGELWLDLPVTAVDASRQLLTSYWGTNLKSQLSDQLYAEIGRVEKVSPRNEEDFKKFSFTANGMTKESDGLNYIDLRYQFTPSLKVEYYFGNLENLYNKHYVGLEHTWKQPTFALTSKFKYFNAKDDGNTFDIDAQNIGLLETVKVKNHTFGLGYQQIMGESAYPLPDGFLPETYFINWNATGFFKEDEKSYHVMYGYDFKDYIPGLNAMVKYVYGHDFKAANGEKNHETESNVILNYAFQQPFLKGVALQYIRIDYNVKHGNDFGEDRLFVNYTKKILNKNYPLNYLG

ST25 4190 cg46_0425

MAVGVGEAALSPAAYSMFSDMFSKDKLGRAVGIYSIGAFLGGGIAFLVGGYVINLLKGVTLIEVPLLGALKAWQIAFLVVGLPGIIIGLLFILTVKDPARKGQQLNQSGQVDQVKFTQCLQFIKKHAKTFACHYLGFTFYAMALYSLTSWTPAFYIRKFQLAPTETGYMLGTILLVANTLGVFCAGWLNDWFIKKGRQDAPMFTGVIGIVGLIIPIAFFTQTDQLWLSVTLLIPAMFFASFPLVISATALQMLAPNQFRARLSALFLLVSNLIGLGVGTTLVAIITDKVFGNPLMVGSSLSIVGGLSCVLALALLFKGCKSFSESMKLEKLH

ST25 4190 cg46_0426

MNQSNIAVERTQKKTNAYAWYVVILCMLAYIFSFIDRQILALMIEPIKADLQLSDTQFSLLHGLAFSLFYAVMGLPLAYIADRFSRPKLISIGIIVWSLATATCGLSKTLFNYS

ST25 4190 cg46_0427

MLDHLEQFLPNKEPSSIQNFPFFWISQVNGKYSQLIEKSIKKLGIDNTRRKIILSTNALGEASITDIANLSTLKLTTATKAVYRLVEDGIVEVYSSTADERISMVKLTAKGVELVEQINQISVVTLAGILNAFSEDELHNLNHQLKKLFDLMPSS

ST25 4190 cg46_0428

MNIISQSVQKGNGKLKVMVKDTIDIQGLKTIAGSRALLEVEPAHDDAEVVKNILKADCEIIGKTNLHELAFGITGINHAFGTPINPKYPELIPGGSSSGSAAAVAAKQADFTLGTDTGGSIRMPAACCGVYGLKPTFGRVSREGVYPPSSSLDCVGPFANSVEMIEKAMQIIDPTFKPAECTRTPKLALLNVKADEVVWNCIYQALQKANLETVLEKVEHFEAAYDAGMQIINYENWQAFGELTQTGLIGSDVNNRLLKAAQTTLEQVKQAEVVKAQLTEELDALLEKYDALVLPTLPQIPPKVSEAENTVAFLNLTGLVRPFNLSGHPAISVPLETSEGLPVGLQIVSKHQKDEQLCAIAKFCVDAMQ

ST25 4190 cg46_0429

MMNKLSKMEFASQDKAVDLDALCQEIRERACAGEFDNQAYVSQDIIEKLKKIGVYRALVPKRFGGEEWSPRQFCELIETLSKADGSVGWVASFGMSPAYLGSLPEETLKELYQNGPDVVFAGGIFPPQPAEITDEGVVVRGRWKFSSGCMGADIVGVGISPLKNNEMQGLPRMAVMPANKAKIEMTWDTVGLKGTGSHDLVVEDVLVEKKWTFVRGEPSKLSEPFFKYPSLSLATQVLTVVGIGVAAAALEEFEKLAPGKASITGGSEIANRPVTQYEFAQADAEFQAAKSWFYQTMDIVWNEIIAGREATAEQISDMRLACTHAARVCAKVTRKMQMLAGMTAIYTNNPFSRFVNDTNVVTQHAFMGDATLQNAGLVSFGLKPTPGYL

ST25 4190 cg48_0432

MSGQEAKACTDSPDTCVNQLKNLPQILDEQLLSAASEMYLAKAMALSDSSECKISRFTKHKPTEEQKVIQNKYDECLDQQLSLLDKSIRYSYAYLFSTKRQPTDRIFDNRQVQIRDFYNQAIAKMVSVYDLRYPQKNVVEPQIHIGKSVYSIDFEFHRQLSGQKLEKLISSYNLNFSGLRTINRRDGFGSEFVAVFPSSGKEDINEYILDPLNYSYKNGVNPNIHHARYLAATIVAEPKHAKTIQEIINDPEFVIRVYDPYRTDNINVAGKQYPLAANFSAPYGLWLAENNLGVAAYLSLIDRDQHLTMPHLYMLEPYNPNKKIIVLVHGLASSPEAWIALTNDVMGDTVLRDNYQVWQVFYSTNMPILESRFQIYALLKQAFGSLNPSDPAAHDAVLVGHSMGGIISRLLVSDADITKPALEMMTIRQQNRFKKHPMVTERLQMHSISNFDRAIFLASPHRGTDYADRWFTLAARKIIRLPGAFLSAVATSLTTENLDIKDFLNNIDNGLIQNGPSDLSHQSKFMELTENINPHQGLIFHSIMGNITKSDDPNVITDGIVPYKSAHLEGAKSEKVLPGGHSIQLTPQAVLELRRILREHLVEHGLYKP

ST25 4190 cg48_0433

VLQNKLNSLPNTTVIMNALSTEVLGDGSQVTGLKYKDRATDEEHVVELAGIFVQIGLLPNTDFLKDSEVELTNRGEIIVNDRNETNVKGVFAAGDCTTVPYKQIIIATGEGAKASLSAFDYIIRSEQ

ST25 4190 cg49_0435

MNKLLVALGLAATVALVGCNKDKAPETGATTGEHLENAAQQATADIKSAGDQAASDIATATDNASAKIDAAADHAADATAKAAAETEATARKATADTAQAVENAAADVKKDAQH

ST25 4190 cg49_0436

MKLYYSPGACSLAAHIILNEINVDFDLERVNLKTHKTEKGADYYEINPKGYVPALEINPGLILTENVAILPFLAQHDPKQDLIPPSGLGRAKVLEWLGYLNSELHDAYAVFFGAALTNDEKTKAYAEIDRLLKYIDNYLAESDYDYLVNDNFGPADAYLFVLTNWSNSIEHDLTPYKHIIALRNKVAERQSVQIAMRDEGLIS

ST25 4190 cg49_0437

MKTFQLKTLSVFLSALGAFSYSSVAQAQLMFSQYVDGSSNKKGLEIYNPDGTTVNLADYEIQQFNNGGTAKTATFRLQGTLASKQKFLVGRSELQTELGNKVNQVAALSFNGDDAVVLVYKGTPVDRFGRIGEQPEAGWGTAVSSLGNSFKRIETENPALSIDPTAAFDLDHSWTAWANRNDFTNLSGTTTQPPIETVSCSSSDTPIADLATSAQNQTYTVRGVITADYRYANGFSGFYVQTPDTKARANVSNAIFVYIPNSSTVKGGQIGDEVILRGRLTSYQNQLQIDQLQQDIQTCNSNMANQVQPISLELPFSSLTGGSTHSPQRYQGMLVKLPQTLTVSENYNYGRYGELSLSLGRLYIPTNLYPALSPEAKALAQKNLLSKIIFDDGYNNQNRTPWLPTNFSAANTLRSGYQLKNIEGVLEYRFNGWRVQPVLGRTQPEVVTQTNPRQSVITKNANHIRVASFNVLNYDNGATGFPTQRGANTQAEFDKQHRKIVSALKSIDADVYGLMEIANNGYGPNSAIAHLTSALGPDWKYVIPENLDRLGNDVIAVAIIYNSKRVKPLNKAVVLDLGDKNRTTLAQTFQAVRGNKTFTVIPNHLKSKGCSGVDANSSDADQNDGQGCWNPTRVKAVDQIVQWLAKNPTQVPKQNALLVGDMNSYAKEAPILAFEKANYKVLLNDAKVGQGAQAYSYVFGVASDANGNGGAGNLDHAIADADLYPKVVRTFAWHINADEPTVLDYNEEYKTDEQKALFYGEDAYRSSDHDPVIVDLDLNGKDSNQPNDNQKSPIFDFLSQLMEWISQLFKRS

ST25 4190 cg49_0438

LNFNIAKVMSIDQLETQIAELDNLPEHREIVTFMRRSAPLNTSQRTALEQHRDLILEYPVGDLRQHFEHPERPLTVEIGFGMGRSLVLMAKANPERNYVGIEVHVPGIAQCVYEAGMAELKNLRVLDADAIQVLREMPDNSINCVQLYFPDPWQKKRHFKRRFVIHERMQLVEQKLELGGTFHAATDWEPYAEWMLDVLDNRPNLENLAGKGNSYPRPEWRPVTKFERRGIESGHKINDFIFKKIK

ST25 4190 cg49_0439

MLKILGRDNSINVRKVLWLCDELNLTYEREDWGIGFRSAQSPEFLELNPNGQIPVILDGDLVLWQSNSIIRYLANAYDKNHILYPTQAKQRAIIDQWVDWQAIELNNSWTYAFMSLIRHSALHQDPNLLQQGIDQWNKQMQILDQQLAKTQAYVAGTEFTLADIPIGLSVQRWKATPFDHPALKHVDQYFERLNQRKGFLKWGNNGQP

ST25 4190 cg49_0440

MKISLLGSGRVAFHLAKALLAQGHHIAQVYARDFEKTKKFAEQIQAKACQSLQEFQPSDLIILAVSDSAITELATQVHEIFPETLMVHTSGSTDIQVISNIHEKAGVFYPLQTFSLERDVDWQATPLFVEATNKDDLVILSDLANSLSKRVYQYTSKQRLTLHLAAVFACNFSNYCFDMAKQVVDAEQVDFGLLYPLILETAKKATENDPKQMQTGPAMRGDQNILVMHQSLLAQANRDDLKQVYQLLSDGIVKRHHSS

ST25 4190 cg49_0441

MSINPIELLKEKVSSTILNNQDGYLGEKTNALSKFYPILLSLLAAKPDLIGQLKNSLAPSLSDLFSHNEQIKNTVLTHLSGTAPNNEIENTLNSALKPSLNAISDVAGNDQQSIVNYLRQYAETIRSYLPGWAVGLLAPLGIGAGLSSVTSSTAPPLAAATETTGKSRGFLPIIALIILGLLIAWLWRSCQHKEATPAPETKAASGVEAAAAPATLTLSTDDKGAVSQCQAGIGDQGFLATLQTQVKQVFSATKDCDVDTSQTYAAAFTDKDALAGVLGALKGIPNASLEWVGDKITLKAGDAAALEALTAKVKALVPHTEVVAAAPETAEQSVSNSLSASQTALTAIDPNNVDVNALVKALNLQIINFASGSSDIPADNKAILDQAATLLNKVSGVKLDVGGHTDSTGNAAANKALSQRRAQAVVDYLVSKGVDASKLVAKGHGSEQPVADNTTEEGRFKNRRIEFSVAQ

ST25 4190 cg49_0442

MDHMSIVKIENVRVLDPIQKTDSVQTVYLQDGKLIAPVDQVEQTIDGQGKWLMPTMVDLCARLREPGQQQHGTLKSEGKAARANGILHVITPPDSKPIVQDNGALIHGLIEKAWHDGGIHMHIIGAQTQGLNGKQPANMAGLKKGGCTAVSNANAAFENDDVVVRTLEYAAGLGLTVVFYAEEPQLAKDGCAHEGFIASRQGLPMIPAIAETVAIAKYLLMIEATGVKAHFGLLSCGASVELIRAAKAKGLPVTADVAMHQLHLTEQLIDGFNSLAHVRPPLRSEQDKELLRQGLKQGVIDAICTHHEPLSSSAKMAPFAETQPGITAFDTYVALGIQLINEGLFEPLEWVTKVTSAPAQVANMTARWQAEAGWVLVDPELSWTVSKDTILSQGKNTPLLGQKLTGKVLQTFAV

ST25 4190 cg49_0443

MHIAALHQPSQVQLNQDGNLKHFLTIEGLSKENLTKILDTAQSFLDDNNNLINRPLLEGRTVMNLFFENSTRTRTTFEAAAKRLSANVLNIDIARSSTSKGETLRDTLWNLEAMAADIFVVRHSSSGAAHFIAKDVCPKVAIINAGDGRHAHPTQAMLDMLTIRRETKKPFEDLSVAIIGDIKHSRVARSDVAALQTLGCKDIRVIAPNTLLPVGFSEYGDHVRLFNNMDEGITGCDVIIALRIQNERIDSPALSSQSEFYRMYGLNKERLSLAKPDCIVMHPGPMNRGVEIDSSIADGEQSVILKQVTNGIAVRMAVLALSMQGQLQEQGLIEAIAL

ST25 4190 cg49_0444

MKDMMGNGFIANAEAFTLATQIYVRMRRVTGRVIDAMYFVQNKDYAKYVIALALEAEDDELKRCVERLMVLTDSIPEQSQKEMTVSIQTSEESEITAEDIYRAQVPHHYIGALR

ST25 4190 cg49_0445

MFWSIEFMNTSLRLNHYWITCADGLETLLQEEIEQLGTKVTERKAGRLIIEGTLEHAYRICMWSRLASRVLLPIHTYELERTHDARDVAEELYEGAISFDWSLIFAPQSTFAIRLHAEREIKVNTQFATLRVKDGVVDSFMEAVGRRPSIDTKQPEITLYVLAGKTEHTYCLDLSGDSLHKRGYRRFMTDAPIKENLAAAILQKAKLQERNPEIVLDPMCGSGTFIIEALMILTDRAPGLVRRFGFNGWHGHDRELWLSLKAEAAERHEKALEQPLPKFYAYDADWEAVKATRENIIAAGFEKLLGDIQIEERTLADWPDFGAENKTAFIVTNPPYGERLGDKASNRSLYLGLSALLQKNFPNQYAAIIAAQIEQADVLAFEAPETLRLMNGKLPIYVRFGTVKPEKVTQPFLANWQAQPVEMEEAQDFANRLQKNMTALKKWATKENIYCLRLYDADLPDFNLAVDLYGDRLHVQEYAPPKKIDPEKAKKRFNLALAAIRAVTGLNRDAIFIKTRARQTGTNQYTKQSTANKRFIVQEGKAKILVNLTDYLDTGLFLDHRQMRLRIAKEARGKHFLNLYSYTSTASLHAALGGAASTTSVDLSNTYLSWSKENFVLNGLTVDHADEQHMFFASDCFEWLKEGHEQYDLIFIDPPTFSNSKKFHGTFDVQRDHVSLIKRAMNRLTSEGTLYFSNNYRGFEMDEEIEALYDVEEITSETIGPDFKRNQKIHRAWKIQHPGLS

ST25 4190 cg49_0446

VVLPTNLWAAQPMNESALATQTGAVTAALPVIIAKAQDDAKKPEIEQRQVRQTLGDNYLQQVRWNSLRTSVLSPYQEQKQNQVIDGKEKKKLEMVALPERADRFTRYDFESKNVHGEVIITVK

ST25 4190 cg49_0447

MLQQCCQLLEDRLLKVAFIESASSGYLTSQFSIHKNSGADILLGGLVSYDPSIKISVLKVDPKLIETYTAESPQVTEEMCRLGQTLFQQADIVVSCTGLLKPGGSETTEKPVGTFFICISYLGRIYHYHYFLSGHAEQKLKTLTQKVADSIIALISSNQHKS

ST25 4190 cg49_0448

MRFEKFTNRLQQALSDAQSLAMGKDHTAIAGIHILSTLLEEPSNISLLQQAGARLPELKQKLEQALKDAPTIANPTGDVNLNPEAVKALNLADRYAQKAGDEFLSTDWVLLGLAETGETKNILSAVGVTPDSLRKVIENIRGSDKVMSNNHEDQRDSLNKYTIDLTERALSGKLDPVIGRDDEIRRTIQVLSRRTKNNPVLIGEPGVGKTAIVEGLAQRIVNGEVPEGLKNKRVLSLDLGSLLAGAKYRGEFEERLKAVLKDLAKHEGEIILFIDELHTLVGAGKGDGAMDAGNMLKPALARGELRCVGATTLDEYRQYIEKDAALERRFQKVLVDEPSVEDTIAILRGLKEKYATHHGVQILDSAIIAAAKMSHRYITDRQLPDKAIDLIDEAASRIKMEIDSKPEALDKLDRRLIQLKMQLEAVKKDEDAGSKAEVTHLEKQIAEVEKEYNDLEEVWKAEKTLVEGTKQAQVELDKARIAFEKAQREGDLAEAARLQYGVIPELQKQLEQDEVAEENEEPKLIRTKVTENEIAEVVSAATGIPVAKMMQGEREKLLHMEEFLHDRVVGQDEAVVAVSNAVRRSRAGLSDPNRPSGSFLFLGPTGVGKTELTKALANFLFDSDDAMIRIDMSEFMEKHSVSRLVGAPPGYVGYEEGGVLTEAVRRKPYSVVLFDEVEKAHPDVFNILLQVLDDGRLTDSQGRVVDFKNTVIVMTSNLGSQDVRELGEGATDDEVRTIVMNAVSQHFRPEFINRIDELVIFHSLKKAQIRGIADIQLDRLRSRLVDRDMSLTVDDSAFDLLIDAGFDPVYGARPLKRAIQQQVENTLAQKILSGDFVAGDTILVKGENGHLVFDKLKLS

ST25 4190 cg49_0449

MAELPQDSRRPGGIAVIPLTSDITQVTFQHKPVLISQEGQQRYAVFGIPLSTPLGSVQLDTNKAPIQIEVKSYPYAEQRLKVTNQDYVNPNQSQLDRYAQEAKEQNDVYSSFTQSSWQALPKFIRPTSGKFTNSFGRKRFFNGEERAPHSGLDIPAPIGQKVVAPADGVVVQTGHYFFNGQTVLIDHGQGLISMFCHLSEIKVEKGQHIRQGETLGLVGKTGRVTGPHLHWGMSLNNARVDPQLFLTTTH

ST25 4190 cg49_0450

VNIRLAQWEDTETICYLLKQMGYSQPLKVVQEQFQILSKDPNAITLVAADDLKVYGVLSLYFIPQIALQGEFAKICYLCVDEKMRSKGVGHLLVQEAERLAKQRGCDRMELHSGIQRPLAHQFYLREGYVEAPKYFRKALN

ST25 4190 cg50_0451

VQYMSLQQAKYYLMQQRSLLDTALNTGLSGTGRLHDLFIQLEGMTPGEYKQQGDGVELNYSVETSPFGDLLVVSSDKGICSVRFVDHSENIQEIVKQYFPKAQLKNHSPIWHQQIAQWFRQDFSEHLQQKLPLNLAGTPFQLQVWEALLTIPEGQLRTYQDIAEQIGKPKAVRAVATAIGQNPIAYLIPCHRVIRATGMVGEYHWQKGRKLALLAWEMAKQQGEIA

ST25 4190 cg50_0452

MTLDLFSPEPCSNLLPYDGEVQDYGCILTAEEAEQYFHYLYHHLAWRHDEAKLYGKHFITPRKVAWYGDEHYRYKYSGVFRDSLPWDKALAQLKQQVEKSLSEKFNSCLANLYEDGTQGMAWHSDSDVSLARTTTIASLSFGATRKFSFRHIQTKEKVEMWLQPGQLIVMRGETQQYWQHRLNRSTKILQPRINLTFRQFKFS

ST25 4190 cg50_0453

MAISSFGKILTVLDLFSVSRPVINVDIICEELGLSKPTSYRYLKELVSADLLKRINGTSGDYTLGSKIAVLDYVSRTTDPLVQISTPFMRNIVERTELCCLLTYLNDDYCIDIHHEIFKDTELLSYGRGCPRPIYVGASPKTMVSHLSKQRMHDYYERFQHELSESGFAVDEPSFIQRMRKIKKQGFYFSQGEIDPNVSGLAVPVRFSSKEVPLALTLVASKNRFDFLNIEKLIEILQENAALIEQRFMELSEKGEI

ST25 4190 cg50_0454

MLLKQILAFRPSKLDLIFAIKTFIAGMLALFVSFELDLINPMWSIGTVLIIANPYSGMVSSKCVYRLLGTVGGAIIALTITPHLINTPWIFTVVLSLWVGFALYVSLLDRTPRSYAFMLAGYSTAMIVYNAITYIDQYNIFDIALARVLEISIGVISSAVVSATILPMHIGSAIKQRVTKTLKDTENLFANLLNADQQQNNTQLLAAITRDTTDIHALAVHLSYEKGELHGMTKPLQEMLHQMSMVVANLVALSERIKQLQELNFIESHSDKLKQLSGHVIHFLEQKDQMTDENILQLPDEFENDFLNLNDSASEHQQVLLAAMKMDVRHFISNVLAVKVLWQRIKQGNKEIPDNITPMTTTYPSLHRDHGVAVRGGISAVLITFIVTGVWILSGWKAGFMMAQIGAVTACILTALDNPVPVLRIFIWGSVASAVLVFIYAFGIFPHVTTFWELGLVLLPMFLFAVSMMANQALMPVGMVLGINTMMGLNLHNAYSMDAVSYLDSSFGMVMGVLVSLVVIDVVRAMSPDTSANRILALHYRAMRQAIYIPYGIEFKVHLRGMLDRVGVLNTKMVQSEEIKKSIQQALVESSAIIDLSRLQELANQVPISSELAHHIGRLQQDLDELFRAKENEKGESDVLVQHIHQTLFELKQLASNIEDMTLRQRLFISLNNIAYSMCHVSSNQMNADRTLTGATAHG

ST25 4190 cg50_0455

MNQLELKKVIRPVILVVALMIAVYTIVHLWNYYNAAPWTRDGRVRGDVIQVSSDVAGLVTEVLVQDNQTVKKGQVLFKIDVSRRALDVEQAKSDLAKAKAAYAQAQAGLAQAKANLIKSSTNIKLAEKNANRYSNLMDGAISKQEQDQVFATRDQSHAEHEQLQAAIQQAEATIKQQQALVEAATSNLHLAELNMHRAAVVAPADGTLSNFDLRPGNYVQVGQAVAALLDRKQLYVVGYFEETKLDRIHIGDEATVQLMGDRQKIKGHVQGIASGIEDRERSSSSKLLANVNPTFSWVRLAQRVPVKIVLDEAPKNELAFVSGRTATVHIIEK

ST25 4190 cg50_0456

MTATQSKSIMAFDFGTQKMGMAIGQSAIESANPLPLFVMKDGIPNWDQLLKIVKEWQPDLFIVGLPLNMDDSESELSTRARKFARRLRHQTNIETWMVDERLTTREAREELGFYQEQGRAKKLSADSFAAALLIQSWYRNPVGLTP

ST25 4190 cg50_0457

VTKQYMTHRCLIAPPEMADDFFANTVIYLARHDEEGAQGIIINRPAGIQIKELLNDLDIDADNVNPHEVLQGGPLRPEAGFVLHTGQPTWHSSIAVGENVCITTSKDILDAIAHNEGVGRYQIALGYASWGKNQLEDEIARGDWLICDADMDLIFNLPYDDRWDAAYKKIGVDRTWLASEIGHA

ST25 4190 cg50_0458

MLTHLTLINFALADHLAIDIEQGFNVLTGETGAGKSLLLDALSACLGERTDTNYVRYGSDKADVTAVFTYQDNSPEAKWLKEHELDDDSGEIHLRRVIFATGRSKAWVNGRPSSLSELKELGRLLVQLYSQHSQQQLLEPPYPKHWLDRYSNFYAEANDVREAYSTWQRNIRQHQAALDAQATRLQRIATLELQIEELEEVIQTDYKEIEQEFDRLSHHEHIMQDCSYSLNALDEAEQNITQEMSSIIRRLESHAGRSEQLSEIYNSLLNAQSEIDDATSNLRQFIDRQSFDPERMEELNSKLEVFHRLARKYRTQPETLKEEYETWQSELEQLHQLEDPETLAEQVEKSHQEFLEKAQHLDNIRREAAAPLAKQLTEQVKPLALPEAHFEFKFEPLEQPNAEGLSFIQLLFTANKGIPPQPLARVASGGELSRIALVMQVMNAEKTEAEVLVFDEIDVGISGGTAEVVGRLLADLAQHVQLLCITHQAQVAAQSDQHLLVKKQQTDPASSTIVQLDENQIISELARMSGGVEINETTLQHAKQLRQLKFQASST

ST25 4190 cg50_0459

MKKTLSLPKAPIGMINRHKKINPAEMNKILNQHFNAFKQAAAQGDYVKAYQHVKKAVSLVPGHPGALSDLAYTELRLRRYDDAYQHYMQAIKASGSNVNTNLYDGLTEVCHHLNKKEEKIKFGRLAISTKKELTKNEPTLNIPTHKPVPFSPNPQENIIAFSLFGANARYCETSILNTKLAQEIYPEWTCRFYVDESVPELVQQRLQANGAQVVHVSPTQKQLSGLFWRFLVMDDPVVKRFLVRDADSIVSYREKTAVDAWLKSDQWFHLMRDSYSHTELILAGMWGGCTGIFHNIEAHIRDYVATGRYPDNRVIDQHYLRYCIWPTLKQSVLVHDSQQFDENSADFPVYDLTSMQNDQDNFHVGMDNGSPVVTTAVNHPTAEKVYWILFDENHDEVCRYDAIVSKSRNIEVNLPHAFAKKIQLQQWKLQVYPYEN

ST25 4190 cg50_0460

MSSTQFDHVTVIKKSNVYFGGACISHTVQFEDGTKKTLGVILPTEQPLTFETHVPERMEIISGECRVKIADSNESELFRAGQSFYVPGNSVFKIETDEVLDYVCHLEG

ST25 4190 cg50_0461

MAKPEYYYGVHSVESLLELEPERVLTLFTLKGRDDQRLQKILQLAEPFGISVQKASRDSLEKLAGLPFHQGVVAAVRPHPTLNEKDLDQLLAETPDALLLALDQVTDPHNLGACIRTAAAMGVQAVIVPRDRSASLTPTARKVAAGGAEKVKFIQVTNLARTLAHLKETTHVRVIGTMLDENALPIQKCDFSGAVVIVMGAEDTGLRPITQAQCDQKAYIPMSGNLQSLNVSVATGMALYEACRQRSEV

ST25 4190 cg50_0462

MSPRTQGYFFVLVTMCIWGGFTLFARLNAQWHISAWDIAALRFAIAFLILMPILIYKKDLAFLWSKHAVILALIGGVIYCLTVYTAFLYAPAAHAAIFLNGCIPICTAIAAYILFRQPFDKHTWVSLAIMITALVLMSALMLQSSTSAFGIGDLLLFISAIWWGIFTVLLKQWKLSAWHSMASVAIWSAIIYLPIYLLFLPKHFMDVDPLHLAVQSIFHGVFVVIIATLTYVAAIQRLGAFKTGSIVTLAPFIAAILAVPLLGESLSPSIVVGLVGMGIGALQPWRWFRQDSLTQKIKQQNTQN

ST25 4190 cg50_0463

MAFILGITGGIGSGKSAATQWFESQGIQVVDADIVAREVVEKGQPALQKIQQTFGDWVLQPDGSLDRRALREYIFQNPQARHTLEQITHPAIRQSIIQQLQNPKSPYVILVSPLLFETNQHELVNHTLLIDASEQTQIQRASQRDGQNQEQIQKIIAAQMPRERKRELANDIVFNDGLLEHLYQQLEPLHQSYLKRAN

ST25 4190 cg50_0464

MQDIIAYFIQNLTALYIAVALVSLCIGSFLNVVIYRTPRMMEQDWQQECQMLLNPEQPIIDHEKLTLSKPASSCPACQQPIRWYQNIPVISWLVLRGKCGHCQHPISIRYPVIELLTMLCSLVVVMMFGPTIQMLFGLVLTWVLIALTFIDFDTQLLPDRFTLPLAALGLGINTFNIYTSPNSAIWGYLIGFLCLWIVYYLFKVITGKEGMGYGDFKLLAALGAWMGPLMLPLIVLLSSLLGAIIGIILLKLRNDNQPFAFGPYIAIAGWVAFLWGDQIMKIYLGG

ST25 4190 cg50_0465

MAVKKAQMMPTFAYEGVDRKGVKIKGELPAKNMALAKVTLRKQGVTVRNIREKRKNILEGLFKKKVTTLDITIFTRQLATMMKAGVPLVQGFEIVAEGLENPAMREVVLGIKGEVEGGSTFASALRKYPQHFDNLFCSLVESGEQSGALETMLDRVAIYKEKSELLKQKIKKAMKYPATVIVVAIVVTIILMVKVVPVFQDLFASFGADLPAFTQMVVNMSKWMQEYWFIMIIAIGAVIAAFLEAKKRSKKFRDGLDKLALKLPIFGDLVYKAIIARYSRTLATTFAAGVPLIDALESTAGATNNVIYEKAVMKIREDVATGQQLQFAMRVSNRFPSMAIQMVAIGEESGALDSMLDKVATYYENEVDNAVDGLTSMMEPLIMAILGVLVGGLVIAMYLPIFQMGSVV

ST25 4190 cg50_0466

MSALHTSPKFTGFFRRLVEEKHVSAATMQTALDAAKRAKQDTVAYLIEEVHLSPSLLAETISAEFAEPYFDLDVYDTSQIPKDLVDQKLILKHRILPLIQRGQILYVATSNPSNIEAIDAIRFNSKLLVEPVIVEYHKLEKVLGQHFAEESSFDFNDEEFDLDVNLDGSTAQEDEEEAPQGDEAPIVKYINKLLIDAIRMGASDLHFEPYEKSYRVRYRVDGVLRQIANPPLQLANRLASRLKVMSQMDISEKRVPQDGRIKLKLSKSKAIDFRVNSLPTLFGEKLVLRILDPSSAMLGIDALGYEEDQKALFMEALDKPQGMLLITGPTGSGKTVSLYTGLNILNTESSNISTAEDPVEINLEGINQVNVNPKVGLTFAAALKSFLRQDPDIIMVGEIRDLETAEIAIKAAQTGHMVMSTLHTNSAPETLTRLRNMGVPSFNIATSVNLVIAQRLARRLCSQCKIPADIPKQSLLEMGFTEQDLAHPDFRVFQPVGCPECREGYKGRVGIYEVMKVTPEISKIIMEDGNALEIAAASEKLGFNNLRRSGLKKVMQGVTSLQEVNRVTSE

ST25 4190 cg50_0467

MSGSTITPWVVGNWKMNPMRANANQLIEEFKQLLQQNQIADENCHVGVAPVSIALTTVQAQLQDAARTVHTVAQDVSRVAGTGAYTGEVSAELLKDSQINFVLVGHSERRDIFGDNVEILKAKLQNALNAGMTVIYCVGESLEQREQGQAEQVVLQQICDIAPVVTAEQWQNQVVIAYEPIWAIGTGKTASPQDAQAMHAKIREGLCQLTPAGSNIAILYGGSVKAENAVELAACPDINGALVGGASLNAASFYQIVQAFAQSK

ST25 4190 cg50_0468

MIALILVQHGKGADAGASFGGGGAATVFGASGSGNFLTRVTAIFDSFILCDQFDSGCFCQKAND

ST25 4190 cg50_0469

VTSLTLAVFAKKQTTEAYSLKTVQTTAPAQTTSPETSPNAPKTGQ

ST25 4190 cg50_0473

MSYNAWDLFDNFIYSRHSRAIQSVVLTDTDLL

ST25 4190 cg50_0474

MIAPAVEACGVDLWGIEFLPQGKRSLLRIYIDRPVDENAEPVINEDGEVEQGRGIGVEDCVRVTQQVGAMLDVHDPISGEYALEVSSPGWDRPFFQLEQLQGYIGQQVALRLIAAVENRRKFQVKLLAVDLENEEIQVEVEGKHVLDIDSNNIDKANLIYQD

ST25 4190 cg50_0475

MGREILTVVETVSNEKGVSREAIFEALEQALVAATKKKFYEGTNSEEAQLRVEIDRRTGDYRTFRQWTVVADEDHEMPACQDAISDVDPAKWSIGDVRELEVESIEFGRIAAQIAKQVIVQKIREAERALVADAYESKVGELIYGEVKKQTKDGFIIDLGDNAEAYLAREEMIPKEILRPKQRVNAILYSVNREGRGAQLLLSRAKPEMLIALMKKEIPEISEEIIEIKAAARQPGVRAKIAVKTNDHRIDPVGACIGMRGTRIQAVQQELNGERIDVVVWSDDPAQYIASALEPADVSGIVLDEDAKSADIIFATNDQLARAIGSQGQNVRLASDLTGYKLDMMLEEEYRARQQNEAQQYLDMFVSRLDIEEDLAMALVEMGFTSLEEIAYVPAETFDEIELDAELVELLQSRAKEAALTDALKQQENIQEPSADLLNMEGMTSEIAYSLAARGIITVDDLADQATDDISDIEGLGHEKAGQLIMKARESWFN

ST25 4190 cg50_0476

MTDKSIKELALSVGRPVEKLLEQAREAGLPQRTADDIITTEQQDTLVNYLKKVHGQESGNTGKIALKRKTTSTAKVASTSGKAKTINVEVRKKQVFAKPNPEQIAAEAKARAEAEAKARAEQQAREAAEQKARLQTEQKAKATLDAMRAAHQQDSAAQSAPKAAVVVKKRGGGTVKPAPKPAETLEQKKAREAQTAQLKATEEAARRKAAEEAQQRTLEQMRKMASKYSNDDATATIRVIDDSPLASGLVGQAYEDSFNQEDREIKRGGATTNPRAGKKGGRRGQEEQSFVNHNKRGLKSSQANKHGFEKPVKKQVYDVEIGSSIVVADLAQKMAIKVREVIKTLMKMGELVNQNQTIDQDTAALVVEEMGHNPVLVSDTQAEDNLLEAAEEARGEQTTRPPVVTIMGHVDHGKTSLLDRIRRSKVAAGEAGGITQHIGAYHVETDKGIITFLDTPGHAAFTSMRARGAKATDIVVLVVAADDGVMPQTAEAIDHARAAGTPIIVAINKMDKESADPDRVLNELTTKEIVPEEWGGDVPVAKVSAHTGQGIDELLDLILIQSELMELKASAEGAAQGVVIEARVDKGRGAVTSILVQNGTLNIGDLVLAGSSYGRVRAMSDENGKPIKSAGPSIPVEILGLPEAPMAGDEVLVVNDEKKAREVADARADREREKRIERQSAMRLENIMASMGKKDVPTVNVVLRTDVRGTLEALNAALHELSTDEVKVRVISSGVGAITESDVILAESSEAVLLGFNVRADTAARQKSDQDGIDIRYYSIIYELIDDVKDAMSGKLAPEHRETILGVAQVREVFRSSKFGAAAGCMVMEGVIHRNKPIRVLRDDVVIFQGELESLRRYKDVVDEVRAGMECGLAVKGYNDIKPLDKIEVYDVQMVKRSL

ST25 4190 cg50_0477

MAGGQRLKRMADSVQRELSELIRQELKDPRLGGLVTISGVKVSPDLGYADVYVTVMGRELSDDQNEVAHRETLDILNKASGFLRQELSRRIKTRITPRLRFHYDKTNAYGNYMFGLIEKAVQDLPKRESDDEE

ST25 4190 cg50_0478

MEIIVSIFLVFGAFFMLVGSIGMVRLPDLFMRLHAPTKSSTLGLGSFLIASMIFFAFQGRFGFAELLITLLAFITAPVSANLIAQAALHLRLRSLSGEVPEAIERPLPWDRYKIGQRFSQNKPPMEPPQA

ST25 4190 cg50_0479

MVMGPSIVDRLLALDTLFLNATCLIIVLGIYWTTTSLFEGALLVAMLGFVSTAALARYFTTGHVID

ST25 4190 cg50_0480

MQLSKLLDRWFPHPFVSFLIIIFWLMLAHSLDASDLLMAVILGLVIPRLVRPFITRTPHIHWKPAIKLCFVVLWDIIISNFRVAKLVLGSPKKLHPKWYRVPLETEHEQVNSLLAMIITTTPGTVSAGIDQERGDILVHSLSTENTESDVQDIKQRYEAPLMEIFDVKSKSEDNA

ST25 4190 cg50_0481

MFDFLSFWQANTPIFSILIPAFTGFILLLLGNPGAGALKEDWRQPWRRGISLISAIAGLITAVSYLLVANTGQITVYQLSEWSAPFGIVLVLDRLSAFMLVLTYALAVPVLWYASENWDTRGRYFHAIFHFLLMGLCGAFLTGDLFNLFVFFEILLMASYVLLLHGQGKPRFQLGVHYVIINLLASALFLIGLGMIYGSVGSLNMADVSRLLPTLDADQHKLAVAGALLLFVVFGIKAAMLPVGFWLPKTYAVASTPVAAIFTIMTKVGIYAILRVNGTVFNDVLSQEIFKSWLLPIGLVTSLYGVIGAIGADRLRRFVGFMVLSSIGTLLIAIAMSNTQAWSGALFYLVHSTLIGAAFYLFCGWMTSQRGDFKDHLKIAPRIKQEKAAMLTYFLIAMMLAGLPPFSGFFGKVFILQATVETAYQGWVIGIVLLVSLLSIIALTRVGFILFWRASPPEEDPINPAYIIYRALPEKAPPRNDTVIYILLAGLVAYVAFASPVLHYTTNTAQQIQDHALYQQSILKTDQNGEVISVQPYDSTYLPETKYGGEVEDHNAYLIPDIISKDTFNGEHISEYKHRQIQQQEKLQTPTIADESQLKPMEP

ST25 4190 cg50_0482

MISLEFLLASAIGLLVATGIYLILRARTFPVVLGLAVLGYAVNLFLFAMGRLQVSSPAILTETTNITDPLPQALVLTAIVIGFATTAFIVQLALRSRYESGSDHVDAKEDISPTYDPREDEP

ST25 4190 cg50_0483

MDTSVLPIIILLPLILGTTLVSLLQRFSRGVTALGAIGVSLTSFGLLLTQAKTVLGGASIQQSWDWLPQLGINLSFRLDALGLLFSLLITGIGTLIYIYAYYYLGPKNSLSKLYLLLMLFMAAMLGISLSNNLIILLVFWELTSISSFLLVGYWSHYEAAQRGSRMALTITGMGGLAMLGGFVLLGQITGTYEINDIVGMKDLIQSHYLFVPTLLLILLGAFTKSAQFPFHFWLPNAMAAPTPVSAYLHSATMVKAGIFLLARLAPIFIGAALYHNIVTFVGLFTLCMAACFAIFKEDLKGLLAYSTISHLGLIVCLFGIGSPLAVAAAIFHIINHATFKAALFMIAGIIDHETGTRDLRKLSGIWQLLPFTATLTMITAASMAGVPLTNGFISKEMFFTELLASLSGSTVIFASIIATLAGIFAVSYSIRLVHGVFFDGPVGKQVPNKNAHEPPIGMRVPAILLATLCILVGIFPALLVEPLVNSVTRASLMQPDFAGTHLAIWHGFNAPLIMSIIALVGGTLFYFALAKDGMIRKIDLDPRLGRLQGRILFDLFLKHLLLTSRKIKQKTENGSLQSYLFLIIAFSIVMVTLPLFNQGLTTGTRELTHAPWIAVVLWLTLFSGCWMMLWFHHERIKAVLISGAIGLVVTMVFVTMSAPDLALTQITVDVVTTVLLLMSLSLLPQLTPYESRRSRRWRDAALAIIGGLGIGWITWLILTRDHNSISWFFAQQSLPLGGGSNIVNVILVDFRGFDTFGEITVLGIAAIGALCLMDGMRVHGTTMTQGLTYRFNPSPLMFRITASWVLPLALVVSVYIFMRGHNYPGGGFIAGLITSMALIIQYIALGQDQAEQMLKAQSGRLYEIWIGSGLTIAGLTGIAAWFWARPFLTSAHVYVELPVLGKLHLASAASFDLGVYITVVGATMLLISVLGDSRHSSMSGPVPHGEKSS

ST25 4190 cg50_0484

MKLESIQFKHIALFHDLKIQFQYDKQPITLILGEQATGKSMLLKHIYHALTWFPARLKDARTPGIVMPDQDISHSRLQSKIEVSIQIPPEFGQLPETTSTQQTDTSLCSWKLFKTLNSSGVGISQVETQQLDQTITLYHQVTRRDPLQGLPLLAYYPSERFVNDINLLNKNLPGITQSISAYDISFIPFTTFARFFEWLREISDIENAQAAHVVQRIILKQSNSQDQEEILRQLQLELAGHPKQLPSPNLYALKSALKIIFPELEDFYLQYHPKLQLMAHYKGEVLAFQQLSNTTKTWIALVGDVVRRLCLLNPLSLDPCLEGEGVLLIDQIDSQLDAAHCSEILNRLHRAFPRLQIIVTGSREELLEHAAVYQCFKLEHGKISPLDLNTTQQQLEHLYTLLQRDEALTPHMDPLPLDTELATAQIDSLYQQIQNLNEQQKNELLRMIHAGDTSEETSSL

ST25 4190 cg50_0485

VSNIEKPKATYEQAIAIDNARLGQSFKVIAYAGTGKTTTLQMISDAMPERRGMYLAFNKAIAGEAQNKFHRNVDCRTFHSLAFRSVPRGVTDKLRLPRLSPSFIAKEYRLEPITLRRMMGGRYEKYVLMPSRLASLVANAVSYFCSTSSQYPAPRHIQAPNWLHPDDITALQTHLYPAVERRWLESIDPNHQAGIGHDIYLKLWALSEPNIPADYVLFDEAQDADPLMLGILLRQKSTQVIYVGDAHQQIYAWRGAVNAMQQLPLPESRLTTSFRFGEAIADVANALLGGLNETVPLLGNPNQKSSVVNKPHTKMRDAILCRTNARAMELLLAGLVHGDKVSLQADHQKLNRFVDAASLLKQGKRVTDVPELAWFNSWHDVHEYCETNEGSDIKPLVKLVDDHGTDPLKKALAKITPLEQADYVISTAHKAKGLEWNRVHIEDDYQFKINGLEHKITDEELRLLYVACTRAKVSLNIHHLYDLIQQLKLRAPLSLRQSVG

ST25 4190 cg50_0486

MSNWLDEVKFDSNGLIPAIAQHHQTGRILMVAWMNRESLALTAEKNQAVYFSRSRQKLWHKGEESGHFQTVYEIRLDCDGDVIVLQVEQHGGIACHTGRESCFYRKLTPQGWEIVDAQLKDPTAIYGDKAKAESHDHAHITEQVDVLAHLGQLMQERKQAEADTSYVASLYKKGINKILEKVGEEGVETIIAAKDYAAQNTESNLNDLIYETADLWFHSIVMLGYFDLNPQLIMDELGRRQGLSGLVEKANRNKV

ST25 4190 cg50_0487

LDKIENFACRVCGLIQDEEPWGESGEDPNFNICDCCGVEFGYEDYTKESVKAYRNKWLEVGANWWKLKEKPEDWDIKKQLQNIPVNFK

ST25 4190 cg50_0488

MKISHLDHLVLTVSNIESTCHFYQTVLGFEVITFKGDRKALKFGNQKINLHQQGNGFEPKALQPTPGSADLCFISDTPISEVVAHLNQLNIQIEEGPIERTGAMHPILSVYIRDPDQNLIEISNNIRL

ST25 4190 cg50_0489

MKTPHFAIIGAGTAGLATAILLAREGNHVTIFEQVDELSPVGAGLLLQPAGLAVFEHLGVLDKALTLGAKVTGLEGQLPDKRLLVNSHYREASTNLYGLGIHRATLCHVLTQKLSEYSSQITWCMNHSVESFVEHNDEVRIFGSHQNQKFDACFDGLLIANGARSQLRPKAWVKVDKAYPWGAAWSIVPECQVLDSEILHQFYDRSRIMMGILPTGAIPTEPQQRLSSVFWSLPTPQLQSFLQDEQAKQAWLKQVSERWPKVAEWLKEILYNSQNQPKWLSANYRDVVMTQFGQGRIGVIGDAAHAMSPQLGQGANMALLDAWAFSQSLQQAQKNQNIDWSLLWQHYHQLRGSSTQFYQFLSRLLTPLYQSDHWWAGGLRDLVFPWMYQIPYFRKEMAITISGLKTGPFKQLDYDQVAQLPKENSAFHLESQSLSDY

ST25 4190 cg51_0491

MKSSDEIETTENKVVKKVVVYAVMVALVFISAMMVVFQVFEYRHDYRELSSYMRERDDLNAEWGRLLIEQQTFGATAQIGTRAVTQLRMFSPPAAETVVISLPMTSEQNK

ST25 4190 cg51_0492

MVDKRTKQTRKKQQSISEKPSLAFDMWRFYLLWAVVLLCFVVLIARAFYVQVINKDFLQNKANANILRTERIEAMRGVISDRHGVPLAISSPIMKIVIDPRDYFETKHLYDQITAELKQDPNNRKLKRQLPDKNLNLDELADVVGVDRADLKKQMNARPRSRYLVLKKEVPPQQADLIMKGNFQGVYAEKTYKRYYPQPQPNAQIIGLTNSEGQGIEGLEMQLNKQLSGVDGEQKIIRDKRGNRLKVSEVIREGEPGENITLSIDSRLQYIMYRELTAAGVANNARSATAIAVDVKTGEILAMTSWPSYNPNDKNGLSNKDAMRNRGAIDMFEPGSTMKPFTISAALETGQYTPNTIVNTSPGSMRLGWHTIRDTHNYGALTVSGVIIKSSNVGSAKIALSLPKETLPSFFNRVGFGKRSAVRFPGESSGLVLPVNKLNSSQIGTMAYGYGLNATILQLAQGYAMLANHGVKMPLSLHKLDQPPKGEQVLNPKIADQVLLMLEQVTMPGGTAKQANIPGYRVGGKTGTAHKLRADGKGYSNNEYRALFAGVAPISDPRLAVIVVVENPQGRYYGGLVAAPVFARIMQESLRLMNVPLDKPLNTPENPIRR

ST25 4190 cg51_0493

MTVSFQEIHPIEIDAQWPQQPFHGFSLDSRKVETGQIFIALTSYSQPEKTRTFAEAALANGALAVISETELGVANEWVCPDVRQRMGEWQKRYLQQADVVKPLRIIAVTGTNGKTTISRLIAELISSQQQRCAVMGTTGNGILPHLTPSTHTTLDALQLQNALHDYAKQGATFASLEASSHGLEQGRLNGCDIEIAVYSNLSRDHLDYHGTLEAYAEAKARLFQFNSLKVAVINLDDAHADLMIKSAQNNPAQPKILTYSLTQDTADYYIADLSYSLAGATFNLVSQQGSFAVESPLLGHFNVENLIAALIAAEQAGFDLQALVNFVPKLIGAPGRMQVIRDDERLFVVDYAHTPDALIQVLKTLKRHVSNQLWAVFGCGGDRDRGKRPLMTQAALDGANPVILTSDNPRTEDPEQIFADMKQGIDFSGHRMHEIHDRREAIKFVAEQAQAGDIVVIAGKGHENYQEINGVRHWFDDVVEVRSAIDAQHHTVDAAYPAQ

ST25 4190 cg51_0494

MHTSTTSTVPLEPWTAQQLQQATQGYWHKDQIPQTEIKRILTDSRHAESGDAFLALKGERFDAHNFVAQVAANGCQVAIVERPIDAEIAQLVVADTRLALGQLGAYRREQNAQLKVIALTGSSGKTTTKEMLGSILSRLAPTLITRGNLNNDLGVPMMLLELRKEHQYAVMELGANHQGEIDYTSKIVQPHVAGILNIGTAHLGEFGGRDGICRAKSEIYRHILPQGVAIVPQQDDFTAEIREAAKSHQIMSFGAGGDVFATEIELLPQSANFQLHTPQGSSFVRLPFAGEHNVQNATAAVAFALALGVSLEDIVKGLEQAQGAKGRLNFIQKAPHLFIDDTYNANPTSMRAAAQVLLQQNGIKVMVMGDIGELGDSSWQEHHDLGRDLAELPLDHIVAVGQFASAALEGAGLHSTKLKAFQTQAEALPFLINLIQTHQPQSMSFLFKGSRFTHMETLMADLMEKL

ST25 4190 cg51_0495

MLLWLFEQLAGYHSSFQVVRYLTLRSLLSVLTSLTIGLVLGPIMIRKLQALKYGQAVSSFAPENHAKKMGTPTMGGILILLSIGISTLLWADLSNPYVWIVLGVMVVFGAVGWADDWIKIRYKDNAGLPARKKFFWTSVASLGAGIALYLIATQQSNAEYTANMLDLLIPFFKNLSIPLSIVPLGLAFIIFTYLVINGASNAVNLTDGLDGLAIMPVVMVATGLGVFAYLSGDIRFANYLHIPYVKYTSELVVICSAMIGAGLAFLWYNAHPAQVFMGDVGALALGAMLGTIAVMVRQEIVFAIMGGVFVMEAVSVFLQIGSLRMRNKRVFLMAPLHHHYEKQGWKETQVVIRFWIITIMLVVLGLMTLKLR

ST25 4190 cg51_0496

MTKVAGLDQLSVINQKVVGEGEVLPQVVLKDGSQVQTGTVATMLHNIELYNAGQRGQIEEELKIAIPTLIKVGLFDLFEVDEWIKGTNAGRTFVGMHAKAYLQQKEQENN

ST25 4190 cg51_0497

MCPVCRQRLELVSKTWRCEQGHSYDIAKQGYVNLHVVQHKHSKNPGDTPESVDARRAFLQGGYYQPLQQAVVHLLKDLKAKMVLDIGCGEGYYTSAMQQVVEQCIGVDIAKNAVQRAAKLNDKVTWVVGTGATLPVIDQSMDVCTSLFSPIPQTEILRVLKDDGYLIVVTPATNHLYAMREALFEQVNPHTPQKFVEQLQDLFELKEQQVIDAPLVLDQQALKNLIAMTPYAYKASPERRMQLEQKAHLQVTASFQIYLFQKRK

ST25 4190 cg51_0498

MYLYIAPTLPEMSSLKKAPLLKPLQVYTADNQLIAEYGGKLSIPVEYKQIPPNFIHAFLAAEDSSFFEHSGISFKGLGRALSESVTGSDVQTGGSTITMQVAKNYYLSPERTLKRKLTEIFLARKIEQNLSKEDILSLYVNKIFLGKNAYGIAAAAKIYYNKSINELSIAQMAMIAGLPKAPSKYNPVVNPERALERRNWILGRMLQLGYISQAEYQKAVAEPINLNMPNRDLNNIHPYAGEMVRSELVKHFGEQAIDSGYKVYTTINAKRQAIAEKAVQDGLEAYDRRHGWRGAEAHDKPLSEFRAYANTYPAQVTKVNSSSFEALMQDGSTVTVQWSGMSWARPYRNANSVGAAPSRASQIVKVKDIVRLRPNEAKTAWSLVQVPKVQGQLIAINPNDGSIEAIVGGYNFYQSKFNRALQGWRQPGSTIKPFLYALALERGMTPYSMVNDSPITIGKWTPKNSDGRYLGMIPLRRALYLSRNTVSVRLLQTVGIERTRQLFMDFGLQEDQIPRNYTIALGTPQVLPIQMATGYATFANGGYRVQPHFIQRIEDAYGKVIYEAKPEYACIPCINAPETTDDAQVTTPDDQVVEVTNKELEQKEKTTKQLNLKQTDKNNSQYRQAQRILKSSSAYDMANILRDVIEHGTGRAALKIGRSDLGGKTGTTNDAKDAWFAGFNGKLVTVTWVGFDQPTTLGRREYGGIAALPIWINFMGQALQGTPAAWVRLEKDAQAPISRDKQEVTTEVGDKKTYRAAPPLARPLYRPAPPQPKTTNNDFDDLPGEEIVIPSKTTPPAMKPSQGAAPKREKDELENLINQIE

ST25 4190 cg51_0499

MGVDISSTSVKLLELSVKNGKYWVESYALMPLPENSVVEKNILNPEAVAEALERAMNLANPQTTHAAIAVPTSTVIHKTIEMDADMSDDEREVQIRVDAEQYIPFPLDEVSLDFEVLPDRLANPNRVNVLLVATRTENVETRVEVLELADLNPKLADVESYAVERAFSVFADSLPMGANTIGILDIGHTMTTLSVMQNGKIIYTREQVFGGKQLTLEIQSRYGLSLEEASRAKKDRSLPDDYEIEVLDPFLDAVVQQAARSLQFFFSSSQFNEIDHILLAGGNANIPGLAKLLQQKLGYRVTIANPFLQMGFSPQVDVQKIENDASSLMVACGLALRSFD

ST25 4190 cg51_0500

MATINLLPWREELREQRKKQFITLCFGVVVLGITTVFGGWFYFDHKLDDQEQANQLIMSTNQNLDQQLKTLNGLQEQRDAIIERMKLIQGLQSQRPVVVRLVDELVRVTPPAMYLTKFSRTGDKFTIEGKAESPNTVAELLRNLEASPWYRNAFMNSFLANEEKKDKAASSLLPRVEDHYGSFVVTVDLGEMGVTTTDDSAKPSTGESVGAAK

ST25 4190 cg51_0501

MSQNELQELSLEQLSAKKKKFNLDKFLQQFNTLDMNNYGSWPISVKITCWIFIFFAVLALGYFIVIQPKLQAIDNAQAQESNLLNEFREKDSKLRNLQQYQVQLQEMQANFNQQLEQLPKETEIPSLVEDINLTGVNSGLKFKNIRLEDEVKQEIFIEQPIAIEATGDYHAFGAFVSSIAALPRIVTMHDFTVDVSPAKDNKSDIPVLNYSIKAKTYRYVGATDTSEQTGKAPAASASTNTTVQPK

ST25 4190 cg51_0502

MMKIKQITLYVLACAVLVGCDSRIDAVNQEMANIRNQPPLPIEPAPIFTPVPQFNYAAHQLKSPFMPSSLAAELKIMAGKRVYPNFSRAPQPLESYALEALNMKGSMRNNRGQILALIQTPDQQIERVQVGNYMGMNQGRITHISPTQIDLVEIVPDGREGYVERPRTLVLIGPAP

ST25 4190 cg51_0503

MNHVFRQFSMGAVAIAIMQAASAQVSMTNIVPMQIAGQGTEIRVMFNGLPPQPQAYQLENPSRLILDFDKAQQGLKQSKISVATNEASSVDVTSDDQRSRLTVNLKDAGAFTTRVEGNTFILKINSAQTSNKPLPVVSAQPQGVSNIGFQRGSQGEGLVVVDLLGSNTPVDVQQQGSKVVIRTIGTKIPTHLARRLNVNDFATPVSSIDAYNDKGVGVITIQSSGSYEYMAYQAENKLTISLKRPQDKNVTSLYKTPNYSGNKLSLDFQDIEVRRVLQLLADFTGINMVAADSVQGNITLRLKDVPWDQALDIILKTKNLDKRRNGNVIWIAPVAELIKAEEEEAKAVAQSVKLAPLQTEYIQLKYAKAGDIMGLITQGSNNSNGLQHTSGGGTSTSTNLNTGVDSLGNNVGSLLSPRGTITQDDRTNTLIINDTAQSIDQIRKMIDLLDVQVKQVMVEARIVRASTSFTKELGVKWGILSQGITNNNNLLVGGSETTLWNLREPKKDETTGGYKYTIERPDNLNVDLGVSNPAGSIAFGLISMSDFMLDLELSALQADGYGEVISTPKVMTADKQTAKVATGQQVPYQSTTNSAAGSTATTSFKDALLSLNVTPSITPDGKIQMKLDISKDSVAGAAPNGELILNKNNINTNVLVDNGETVILGGVFEQTTMNSQTKVPFFGDIPVVGRLFRKDVKSDDKQELLIFVTPRIVNDTLARNH

ST25 4190 cg51_0504

MKAIEIGGALPSKAFETLPNIYLVGPMGAGKTTVGRHLAELLGREFLDSDHEIERKTGATIPWIFEKEGEVGFRTRETVVLNELTSRKALVLATGGGAITQAPNREFLKQRGIVVYLYTPVELQLQRTYRDKNRPLLQVENPEQKLRDLLKIRDPLYREVAHYTIETNQGAARDLAQKILQLILSNKLK

ST25 4190 cg51_0505

MQTLHVELGERRYPIFIGSQLDPKQLLEPYIHGQQVMIVSNVTVAPLYLSHYQEALESLGKTVATCILPDGEKYKDIQHLNLIFDALLEAGFNRDCTVLALGGGVIGDMAGFASACFQRGVYFVQVPTTLLSQVDSSVGGKTGINHPLGKNMLGAFQQPQVVLADMAQLNTLPERELSAGLAEVIKYALLGDEDFLVWLEENMDGLVARDADLLAEAVYRSCAHKARIVANDEKEQGERALLNLGHTFGHAIESYLGYGTWLHGEAVATGMVMAADLSQRLGWISNEDVARTKKIIQRANLPISCPQIPLDDFLGYMAHDKKVLNGQLRLVLLKQLGQAVITKDFDVELMKQAILANQHG

ST25 4190 cg51_0506

MTVSRTLWQNVQQYIWLVGGIVCLFLAFIFWVITDNKKLVEVEKSADSDAPVQIQPEKVATTPNLGALADEVRPLDLTTRTVASGEHEPEFRGTKFINENKKQWTLELFRASDEDIIKNFLKSRTDRNKFIYFRLSGEQQAEQYVLAYGTFKRSEDAVQQLTQINLQLPDSVKPQPQQFSSYAPLVNDLGADEMKGGNNQLYEVRLRPAALPRIDESLLMSAPASGTLNTQPKPASTNSATKTTIVRRDAAGNVVDVQQSNSNIDQPNKPAQTRVNENQPKNATNEGTRGN

ST25 4190 cg51_0507

MHMPSPNTVAPAQGLYQPDEFKDNCGFGLIAHMKGESSHHLVETAIHSLSCMTHRGGIAADGKTGDGCGLLLAMPKQFFRDEAKKLSDITLSEIFAVGTVFLNIDPALAQHSKNILTKEIESEGLRVLAWRIVPTNNDALGEIALQSLPAFEQVIVNCPMGVTEVEFNRKLFLARRRAEQQLQNDPLFYVTTLCSTVISYKGLMMPAAIAEFYTDLADERLKSHIVVFHQRFSTNTLPRWPLAQPFRYLAHNGEINTITANRNWALARTPKFENPLLPGLTELNPIVNRTGSDSSSLDNMLEILVGGGMDLFRALRMLVPPAWQNVETLDADLRAFYEFNSKHMEAWDGPAGLVIQDGRHAICMLDRNGLRPARWVITKNDYITLASEIGVWGYEPEDVVSKGRVGPGQILVVDTLTGKVLDTKDVSNHLKNMRPYREWLRDHAIRLNANPELEEQLVDKGLTGDALKAAQKMFMVTFEERDQLLRPIAESGQEAVGSMGDDTPMAVLSRQVRHVTDYFRQQFAQVTNPPIDPLRESIVMSLETCLGREQNVFEQGPEHADRIIISSPVLSNSKMQQIRSIERAGYEAVDIDLNYAETEGLQAAITRICEESAQAVRDGKTLIVLTDKNIRQGYLPANAALATGAVHHHLIKTGLRTDANIMVETGFARDPHQFAVLLGFGATAIYPYLAYDVINDLIAKGELLGDPIHAQANFRKGIEKGLLKVLSKMGISTVASYRGGQLFEAVGLSSEVVDQCFLGVPSRIQGATFVDLENDQKKLAATAWSNRKPIDQGGLLKFVFGKEYHAFNPDVINSLHKAVRSGKYEDFKEYAELVNNRPIATIRDLFKLKTTNPIPVEQVESVEAILPRFDSAGMSLGALSPEAHEAIAIAMNTIGGRSNSGEGGEDPARYGTIRNSKIKQIASGRFGVTPAYLTSAEVLQIKVAQGAKPGEGGQLPGGKVNGLIARLRYSVPGVTLISPPPHHDIYSIEDLSQLIFDLKQVNPKAMVSVKLVSEPGVGTIAAGVAKAYADFITISGYDGGTAASPLSSIHHAGSPWELGLSEAHQALRVNDLRGKVRVQTDGGLKTGLDVIKAAILGAESFGFGSTPMIALGCKYLRICHLNNCATGVATQQGHLRQEHYIGEPQMLINFFHFIAEETREWLAALGVASLKDLIGRVDLLEVLPGETEKHAHLDLSALLTSHPAAEGKAQYCEVEGNAPFDKGVLAERMVEEMLPAIESSAGGQFNFNVVNCDRSIGARVSGEIARRYGNLGMEAHPVVMNLTGTAGQSLGVWNAGGLHIRLEGDANDYVGKGMAGGRISIFPPKGSPFQTQNTAIIGNTCLYGATGGKLFAAGTAGERFAVRNSGAFAVIEGAGDHCCEYMTGGVVTVLGKVGHNFGAGMTGGFAYVLDLDNDFVDYYNHELIDLTRISTESMEDHKEFLLRIIDEHIKETGSAWAYKIRNEFDFYSRKFWLVKPKAANLQSLLKTTQADPQ

ST25 4190 cg52_0508

MQQWSLTAVEAGLLASAALFGMMFGAMIFGTLSDKLGRKKTILICVTLFSGFTFIGAFAKGPTEFAILRFIAGLGIGGVMPNVVALMTEYAPKKIRSTLVAIMFSGYAIGGMTSALLGAWLVKDMGWQIMFLIAGIPLLLLPLIWKFLPESLAFLVKSNHSEQAKNIVSKIAPQTQVNANTQLVLNESTTTDAPVRALFQQGRTFSTFMFWIAFFMCLLMVYALGSWLPKLMLQAGYSLGASMLFLFALNIGGMVGAIGGGALADRFHLKPVITIMFIVGSAALILLGINSPQFILYSLIAIAGAATIGSQILLYTFVAQFYPTALRSTGMGWASGIGRIGAIIGPVLTGALLSFELPHQMNFLAIAIPGVIAALAIFMVNLKASVAAQTPSTFNPQNTLTQQ

ST25 4190 cg52_0509

MEFVHRFKPCNLAITTLFGLLTSSSLWAADTTQQSEDEWKFTLKNAYINRNFDNDALKDTGSWSQAASLFYKSKMHDTPLIIADKPITIGADASVQYAVRLSSDKHVADTVLPFNKETQSQASDFLKYGATLKLGYDKTLLSIGELWLDLPVTAVDASRQLLASYWGTNLKSQLSDQLYAEIGRVEKVSPRNEEDFKKFSFTANGMTKESDGLNYIDLRYQFTPTLKGEYYFGNLEDLYNKHYVGLEHNWKQPNFALTSKFKYFNAKDDGNTFDIDAQNIGILETFKVKNHTFGLGYQQIMGESAYPLPDGFLPETYFINWNATGFFKEDERSYHVMYGYDFKDYVPGLNAMVKYVYGHDFKAANGEKNHETESNVILNYTFQQPFLKGFALQYIRIDYNVKHGNDFGEDRLFVNYTKKF

ST25 4190 cg52_0510

MGIRITGTGLFHPTEIISNEELADSLNAYVEQYNQENAEKIAAGELEELRGSSAEFIEKASGIKRRYVIEKSGILDPTRLRPRLSERSNDELSIQAEWGVIAAKQAMENAGVTAEDIDVVILACSNMQRAYPAVAIEIQSALGIQGYAYDMNVACSAATFGLKQAADAIRSGARRVLLVNVEITSGHLDYRNRDCHFIFGDVATASIIEETTTKTGFEILDIHLFTQFSNNIRNNFGFLNRSEDAVVDDKLFRQDGRKVFKDVCPLVAKIINAQLEKMQLTANDIKRFWLHQANANMNELILKYVAGKDADLSRAPIILDEFANTSSAGVIIALHCTGHEVDDGEYGVISSFGAGYSVGSIVVQKHVA

ST25 4190 cg52_0511

LNFQENLSVLNGLNIDQWVMARDRYRLNRLRKDKKANTAEIEKLFEQSNAKVRQRFERLPKIKLNQDLPVTQYADRLIEAIQKHQVIIVAGETGSGKTTQLPQIAMLAGRGLTGMIGHTQPRRLAARSVSQRIAEEVGEKLGESIGFKIRFNEQGSQDSIVRLMTDGILLAELGNDRFLSKYDTIIIDEAHERSLNIDFIMGYLKQLLPKRPDLKVIVTSATLDVNRFSNYFNNAPIFEVEGRSYPVEVRYRPISEMNIGGSDDDEFDDFEENLPRAVVQAVEECFADAEEKGHPEHADILIFSSTEQEIRELQETLQKYGPRHTEILPLYARLAVSEQQKIFSPSGKGRRIIIATNVAETALTVPNIRYVIDSGFARISRYNYRSRVQRLPIEAISQAAANQRKGRCGRVAPGVCIRLYSEEDFLSRPEFTEPEIKRTNLASVILQMQSLGLGELENFDFIEPPDFRLVNDGRKLLIELGALNEKKNELTKVGQMMARMPIDPRLARMIVGGAHFGVLKEILVVVSALAVQDPRERPADKQMQADQKHALFKEADSDFLFYLKLWDTLNPKGEAGMSENKRRQFAKQHFLSWLRLREWKQTHQQLVELAEGLKLSFNEKPANYENLHRALLTGLLSFIANKTDERNTFMAVRQQKAKVFPASTLHKTNTAWVMAFEMVETSQVYLRTLAKIDPEWILLAARDLLKYHYFEPHWSKKAGIVNAYAQISLFGLIIEPKRLVNFEKVDQPAAHEIFLRDALTTGNLGTVPPFLKHNLLKLEEVERVEDKLRRRDLVVDEETIYQFYASKIPEEIASRRSFEDWRATVEAKNPRYLYVEDDALWLNDRPTTQQFPDHLHNGQLRLATTYRFDPSHDEDGATVQIPVQALPQVDENIWSWGIPGWRLDLIEALLKALPKDKRRNLVPIPDTARKLMQGIDAVHLRQHIFSYLAFALRGEQITEKDFSFERIDPYLVPFIKVIDEKGKLIAKGRDLAELKARCRVETHRPVKQQKGEFKTFPENFTFEASQKVTGVVVKQYQALVPTKNFAELEAKDESGVVIQTFNDQAEAIKQHREGIIRLVHMQLGDLVRQLKKQISKPLALAYSPLGDKAKLEQMLVYATLQMAITTLPINADEFNKLLDQVKKQFLSYGQQALEQLGDIYIQWQQIRRQLLVLDPDVFGRSIDDIEDQLDLMSLGNFVYCQPVDLWQEYPRYLKALLLRLDRLPNNLQRDHAAIDDVDPWMDKVFKFKNDPRIKELYLMLEELRISLFSQPMKTKMPISPTRLQKLWDRLGIS

ST25 4190 cg52_0512

METMMSLINTEVKPFQATAYHNGQFIEVNETNLKGKWSVVFFYPADFTFVCPTELEDLADNYAEFQKLGVEIYGVSTDTHFTHKAWHDTSDAIKKIQYPLIGDPTWTLSKNFDVLIESEGLADRGTFVIDPEGKIQIVEINAGGIGRDASELLRKVKAAQYVHSHPGEVCPAKWKEGEATLAPSIDLVGKI

ST25 4190 cg52_0513

MANTAVVTSWKYNATSRHLKIFYSDGSGDLYHPVPEFVYDNLLRSTDKAAFVHKYLEFNLHFTRLSLVIAS

ST25 4190 cg52_0514

MPNTIFLQRVADLYDTFKQHDSQQSDRLQRYRNIEVESAQLISQLIRMQQAKSILEIGTSTGYSTLWLAEAAQATGGQVITVEIDAKRSAEAKRHVAELELSEIVQFWVGDAADYLKAAQEKFDFILLDAERDAYENYWPDLKRLMKPKGGVLVVDNVISHAAEVNRFIALIKKDPDYMSSILPVGAGLCLVVTK

ST25 4190 cg52_0515

MSLQRGIYQHYKGNLYQVFSVAKHSETEEELVVYQCLYGDYSTWVRPLDMFTETVQMSDGSVVPRFKLIQST

ST25 4190 cg53_0516

MGAVLLTLAIIFTQLSLIAFGGGNTILPEMQRQVVDIHHWMTAQEFSALFAMAQAAPGPNMMIVPLVGWHVAGLSGLLVTSTAKFLPSSLITVFVMRGWSKFKDKKWRRVLQLALQPVTVGTVLASAWIISEAAAINTLLIIMVVIATLLSLIKKVHPLHVLIAGAVFGVVLL

ST25 4190 cg53_0517

MNHVEIAVQKDTVPTCTELFLGFLTLGLIGFGGVLPLARKVIVEQRHWLSPEKFTELLGLCQFLPGGNIINLSVAIGMEFRGVRGAVSSLIGLIFAPTVIVVLLHYVYEQFQDNLMVKHLFEGLGAAAAGLLVATGLKMLKPLLCNPLAICVVVAAIVSIAFLKIPLLLTMLILLAFYSAIIWRRV

ST25 4190 cg53_0518

MLDLYKLHAFVVVVEERNITHAANRLFIQQPPLTRLLKKLEQELGTQLLIRQPRGIEPTEAGLALFKEAQLLLEHARHIPKLVQDVSQGKTGQLNIGFTSSAGLHPLISLVLRSYREMYPAVQTKLEEAGSQKQLDWLISEKLDIAFLRAPISRDIGLKHLHILNEPMVVALPIGHPLTQKKKLV

ST25 4190 cg53_0519

VAAGIGISIVPASMHNFWDHEIVYREFDDSIKLNAPIYLITRENENSAKVSNFIELMQKLLPTMT

ST25 4190 cg53_0520

VSLNELSVDNVNIPIAIGLVLMMYPPLAKVDYAALPEVFKDKKTLTLSLIQNWIIAPVLMFILAIVFLHNYPEYMTGLILIGLARCIAMVLVWNGLACGDNQYVAALVAFNSIFQILFFSTYAWLFLTFLPPYFGVAGQVIDVNFWTITHAVLVYLGIPFLMGFLTRLILVKSKGLEWYQTKFLPKISPISLIALLFTIVAMFSLKGGDVVSLPLDVLRIAIPLTIYFVVMFFISFFMSKRMGNNYPRTTAISFTAAGNNFELALAVAIATFGLASPVAFTTVIGPLVEVPVLISLVTVSLWLKKKYYS

ST25 4190 cg54_0522

MQPIHTHSTLQYIHISVPEILLSNIQIKNSWQDYNQEWIYRLDPPHASHPFQRDLYIIKSKNIETEDIKKLLDNTVFKNSKNKDDLKNIVEAETVIKEILDLSNYIPIENWLNDTGNRSIVESMIDKNKVKLLDII

ST25 4190 cg54_0523

MENTTNQYPSEVPAQVQPHQPGDQEKMHPEPEIIKASHKGSEKLKGKVAVISGGDSGIGRSVAVLFAREGADIAVLYLEEDQDAEITKQLIEKEGQQCLLLKGDISDPDLAKQNIDKVLQHFGKINILVNNAGVQYQQKEIESISNEQLEKTFKTNIFAMFYLTKEAIPYMEEGDSIINTTSITSYQGHDELIDYASTKGAITSFTRSLSNNLMKQKKGIRVNGVAPGPIWTPLIPSSFDAETVEKFGKDTPMGRMGQPSEVAPAYLFLASDDASYITGQVIHVNGGQIVNG

ST25 4190 cg54_0524

VHARGVGAHGYFQAYEGNERLTKAGFLTDPTIQTPIFVRFSTVQGPRGSADTVRDIRGFAIKFYTQEGNFDLVGNNAPVFFVQDGIKFPDFVHAVKPEPDTEIPTGATAHDTFWDFVSLVPESAHAVIWAMSDRAIPRNLRSIQGFGVHTFRLINAEGKSHFVKFHWTPKQGLSALVWDEAQKLAGKDPDFHRRDLYEAIEQGFYPEWELGVQIVEEEDEMNFDFDLLDPTKIIPEELVPVTPIGRFVLNRNVDNFFAETEQVAFCPGHVVPGIDFTDDPLLQARLFSYTDTQLSRLGGPNFHQIPINKPVCPFHNNQRDGIHQHTIHKGQAAYQPNSIDGNWPVETPPAASNGGFESYPEKISGHKLRQRSETFSDYFSQPRLYYKSLAPHEQKHVVDAYTFELSKVQRKHIRERQVQQILANIDLDLARQVGANLGIEVPDLTLNYKKTAVEKSAKLSFLAFPPEDIQGRKVAVLIHNMVKSDALEAIKNWAIKEGVILHLLAPSLAPVKDHEDNVIVADGMQMSEPSIAYDAVIIPDGDNLNTVMQDGVARHYLLEAYKHLKPIVFLGNKSDLLEPLGLVADEGTLIEDEFQHVAEKFKNLIKAHRVWSREQIAAHVPA

ST25 4190 cg54_0525

MDMNDNTKSTNKTSEMAGADAANKANTTQKTEQLDTVRDDATNEALTTNQGVKIADNQNSLRAGIRGSTLLEDFILREKIHPL

ST25 4190 cg54_0526

MKMPVVVKKRLLDLLQDNQQNYYELFVFFLDPDVSSFEKEKKARDFLVKEIDKLEMKEIDFPSDINLIKAWCENDNKKNCQEFQAYLNRRQSGQDREYFKNVAQAFEFLIKVSPTKKVDGAWLYSSVHYWNDPIFHELIITYLEELGLGEPKANHVCIYDDLLRSLGLDSFDLLLEDEYYHQAVVQLALGYAPPEFIPEIVGFNLGYEQLPLHLLISNYELAELGIDSKYFNLHITIDNIDNGHAYKAIKVIEDIYNKYRDKKLFLTKLKRGFALNNHGVSSSNIIKNLNTEDFVHRIFKRKALVGQLIHNETRQFGCKTINQWLSNPDDIAGLITHLTEHKWIKFNTDPEQSVFWRMINEENGKMFGVFNPVERQIIHDWIAGSDHSSNFLAYSRELKNSQRIQDYLFSYISDGELDALQERVQQSNDLAIKICKLTPFLAPDSHHKSIGLWSTRKYVELLFPYLGTFKN

ST25 4190 cg55_0527

VNIQQKDDTKHGSFSLLDGETVAGEMTYTWAGESMLIIDATDVNENYRGQGVGRQLLDALVAFVREKDVKVIPLCPYAKSVFV

ST25 4190 cg55_0528

MSSFHQIEQSTYEHSTPAWGAVFVMSLCCAVLIASEFMPVSLLTPISLDLNISEGQVGQAIAISGIFAVITSLSISRVFKALDRRHIILLLTLLMIVSGIVITFAHSAALFMLGRAILGVVIGGFWAMSTSIVMRLVPPLSVPKALGLLNGGNALATTIAAPLGSFLGSIIGWRGAFFCIVPIAIVALIWQFKSMPSLPAILSAEKSKNPFALLKRPIVLYGMTGILLLFMGQFALFTYLRPFLETVTHVDATMLSILLLILGLAGLVGTFVISLILHQHVYRYLILIPLIMAVIAGAFVFGGEHLWFVAILMGFWGFIGTSAPVAWNTWLAQTLHQDAEIGGGLMVAIIQFAITLGATIGGLLYDLHGYSATFYMSATVLLFGAITAYITWRNATHVTHQ

ST25 4190 cg55_0529

MINKENYNDLYAFLMVAREGSFTKAAGKMGISQSALSHTIRGLEERLGMLLLTRTTRSVSPTAAGERLKQTISASFDQIDNELSLLTSFRDQPAGTVRINASSHAIREVLIPKLKKIAGVYPDVQVEVTAYSGMVDIVAERFDAGVRFGSRVADGMIAVRISPDVKMAVIATPAYFEQYGIPHTPKDLINHQCIGYRLTTQGSVYAWEFVKDGQETKVKIQGQWIFNESYDIVEAVRAGFGLAYIPEDLIREDLKEGKLIQVLEEYSIQFPGYHLYYPHRRQLSPALRLVIDVLRE

ST25 4190 cg55_0530

MKIRIDIDGTQQPLFATLAITPTVEDFIKQLPLTLTLKDYDATEKIADLPTKLTTQNAPDGYAGKAGDLTYYAPWGNLAFFYKDSAVGYANGLIFLGKLDAIPHAFKQKQPIKISISQIE

ST25 4190 cg55_0531

MTTSPLFKKLKTHTVVAICAATIGLFSSTSAMAADMSNGANNFYQSQQVTIQKVTFKNQYNMDIVGNLVIPKNLNKKTKAPAIIIGHPMGAVKEQSSMLYAQKLAEQGFVTLAIDQSFWGESAGQPRNVVAPDIYAEAFSAAVDYLGTQSFIDPNNIGVLGICGSGSFVISAAKIDPRMKAIATVSMYDMGAANRNGLRHSQTLEQRKQIVAEAAAQRYIEFKGGETKYTSGTTHELTADTHPIQREFYDFYRTPRGEYTPKGSSRELTTHPTLTSNVKFLNFYPFNDIETISPRPMLFITGDQAHSKEFSEEAYKLAGQPKELYYVKGAGHVDLYDRVDLIPFNKLTSFFKQNLK

ST25 4190 cg55_0532

MSTQHVSFQQASPNEQAVILIVDDVPENLGLLHESLDQAGYRVLVTTDGLSAIEIAHRCLPDMILLDGNMPHMDGFESCIQLKASPITQFIPVIFMTGLSETEHIVRGFQVGGVDYVTKPLNIEEVLARVKTHLAHAKLLQQQKQVIDATETAILALDVHGKIVWKTQKASDLLNQYLPELEGFEAGLKAWFSDLEHEKDSKKLTSCSYSTPSQQLQLLLLTPWENEPSVAKNYLIQIKTSTPALEMADILKYCPQLTQREAEVMHWLLLGKTNKDIAEILELSPRTVNKHLEHVFEKLCVENRTAAVAHINSLVQEFH

ST25 4190 cg55_0533

MPNMSVMPDSSRPQQAPQRVIKTRREYNIWVADESIEDYALRYAPTSVRKWSPWTVTNTAISTVSFLAMEAIGATMLWQYGFSNALWAAIVVCTIIFLTSWPISYYAAKYNVDVDLLTRGAGFGYIGSTITSLIYASFTFILLAFEAAIMAMALELALGIPQVIGYLISALVILPLVVKGIGFINKVQAVTQPIWLLLLLLPWFFVLWKQPQILSSSLHFVGAVSNSTDFNLYYFGAACTLIFSFVIQIGEQADYLRFLPQKEKNRTAWRFAVFLGGPSWIIFGFLKVLMGMLLMVLAFQLFTPVSELDNPTYLYWVAYQQFIPNPQLALILTLALVCLAQIKINMTNAYAGSLAWSNFFARLTHSHPGRIVWLLFNVFIAIVLMEMGISHAVERILGLYSNIALAWIGAVVADLIICKPLGLSPKGIEFRRAYLYDINPVGVGALLIASVLSMLSYLGFFGLMAKGLASFIALGSAVLCVPIIAYLTKGKYYIARQPEKIQATSVANCVVCERDYELADMAGCPAYNGTICSLCCSLEARCHDLCKPDARWSVQLKKAIWHYLPERWASRLNSRVSLYLLLTLGLSIVLAVSLSLVYIQEKTYLETINAAAVPQLFTLFVKIYTILFLLMSVAAWWLVLNDESRRNAEIETHQQTALLIDEIAAHEVTSKDLQDARIAAERANDAKSRYVIGISHELRTPLNSILGYSQLLQKQEQLSEQGKMALGVISRSGQHLTSLIDGLLDLARIETGKISLNVVDVHFPNFIEQIIQMFQPQFEQKNLQFVYEIGDNLPHYVRTDKKRLEQVLINLLGNALKFTTSGTITLKVEYRFQTAYFEIRDTGCGIAEADLERIFNPFERGSNVVQGGFTGTGLGLPIVKLLVDLLGGQLSVTSQLNQGSQFKIKFYLPSKEVVHPISNVYSNQITGYQGERKRILVVDNEAVDRGLVANFLKPLGFMIEEAESGIDCLRRVPIFQPNLILMDLNMPLMGGWETARLLRQNNITNVPILIISANAGEREVNPQDAVLSEDFMLKPIDLNLLLSKIGDKLGLVWIDSKSETLLENNEVQALDLNIYKQEQAAVHTPIQRPENNDLLDLPQSLQQLNDLIGQGYIRGIQQALMQCRQNFPEHNELWEQLEQAIQKFDLKHAQRLIQDKQ

ST25 4190 cg55_0534

LTENDWEIDVMSVSEHPNLDTVQGTDSQKAVNETLEDYTLRYAPHSFRRWSPKVVAITALGGIAYLADFSIGASIGMSYGTTNAVFSILFAAIIIFLTGIPLAYYAARYNIDLDLITRGAGFGYIGSVLTSIIFASFTFIFFALEGSIMAQGLLLGLGIPLWAGYLISTVMVIPLVIYGMKALSKLQVWTTPLWLVLMIGPVAYLIYQEPTLVSQFATFTGHEGFAPVDMAAIMLGAGICLSLIMQIGEQIDYLRFMPAKTKENSKAWWAAVISAGPGWVILGAIKQIIGAFLGFYLLTKIPGVNSTEPVQQFNAAFHDMLPGWAALTLAVILVVISQIKINVTNAYSGSLAWTSAYTRISKHYPGRIVFVIVNLAIALALMEGNMFAVLGKILGFYSNFAIAWVVVVATDISINKYVLKLSPKEPEYRRDMLYNVNPVGMVAFLVSAGLSIAAFFGLLGSFLAPYSPIIALVLAFVLTPIMGLLTKGKYYIKSHDDGVKEPRYDAEGTPVATVYHCRVCEQGYERPDIMFSHKHNGTICSLCKTLDA

ST25 4190 cg55_0535

MDRTDKKILAELQLNGRLSITELAEKVGLSISPCHRRVKALEESGAIKGYRAELDPNLVGFEFSAIVFITLKEGDKQAVEKFENAVIEIPQIIQAQRLFGEPDYLLHVVARDLPAFQRLYDEKLSAIPSVQRLISTIVMKDVVPERLFPIG

ST25 4190 cg55_0536

MLFGHFITILLVAAGVGLLVANNPTALMILTIAGSAYLLWMGINLLLTPPTPNESDSEKAQSWLSWATKGVYVSGLNPKVFLLFLALLPQFIDTTASWSVTTQILAFGVVHMISCAIIYLMVGYGSEAILKTRPQAAQLVGRFSGGLMVVIATCLLIGQI

ST25 4190 cg55_0537

MPKKKEFEHGGARENAGRKAQFNEPTKVIRVPESQVNFIKNWLLNNVKTDHQTDFTSKLKIQQVHPNNDKIYHIPLATERVAAGFPSPAQDDIEQALDLNEYLIRNENATFIVKANSLSMLDAGIDIDDPLIVDRSIPAKSGDIVIALIDNDFTVKRLMIDTQFQPPKVWLKAENPDYQNIYIEEGQELVIWGVVTYNLKRMR

ST25 4190 cg55_0538

MKNQRDAVLGYADEQHAYYIRLIENDAVLGESYGIFCENPESEAAESIMTTLFLKKSFWLNGSEFTDIVKGLHPLTHS

ST25 4190 cg56_0540

MDLGLIKVFVCIYESRHISKAADKLNLSQPSVTYNLNRLRRQLNDELFTRSRGGVEPTKVAHELYPIFHQAIFNIESAIAQAQEFHPQNSNKTFRIGLSDAGEICLLPSLMAFLQKTAPSIQIEIEEIQSSKVEQWLIDGFLDLAVFNSTQTIMPKIETRTLFEERYVCIARADHPRLQQQLTLEQYLQEDHVAIKSSTGHIQVEQYLKTMDLKRRIRLEVPHFSVLQGVLQSSDMLVTLPSRAARHYLAHGNVQMFELPFAMDSFKVSLNWFNRSDDIIARKWLIQSVGQIFEVL

ST25 4190 cg56_0541

MEKLNANQLIDDARLTSLHWRVILLSALIIIFDGYDLVIYGVALPKLMQHWQLDSMTAGLLGSVALFGMMFGAMIFGSLADKLESYGFSRKRLIMLCVLIFSGFTVLCGFANSPKEFAIYRFIAGLGLGGVMPNVIALMTEFAPKKLRSTLVSLMFSGYAIGGMSSALLGMWLVPKFGWQIMFILAGIPLISLPIIWKFLPESIDYLVRQKKWNEVRDLLQQLAPEQSITDHTSIVLHQENQQTAARPLVALFTENRALVTVFFWLSVFMALLMVYALGNWIPKLMVEAGYDLSTSLVFLLALNLGGMLGAIGGGYFADRFHLGKVICSLFFAGAVSLYLLSYALPMVILYFCVAIAGAASIGGQILLLAYMAQFYPSPIRSTGIGMALGVGRIGAILGPILCGWLLSLHLPIEYNFIALAIPCVLAALSVIAISICNRTKRVMLNSSISP

ST25 4190 cg56_0542

VVGGGIAGLALASNLSKHAHLDVQMFESAPQFSEIGAGISFGANAVKAIELLGLTNEYHAIADKVSAPFQEVWFQWRNGYTDEYLSASIAAGVGQSSVHRADFLDAIIPHMPTQNVHFSKRLEAIEEQDDQVILHFNDGTQHECDYLIGADGIRSVTRQYVLASHHLPPVHPRFSGTWAYRGIISHASFKEALQQINGDTDLADIPQMLLGKDKHILTFPIRKGEQINIVAFCSNREDTVLPADTPWTKPVDKAQMLSDFSDWSESCQTLLGLIEQPTIWALHEIEELSTYQSVSGRVILMGDAAHAMLPHQGAGAGQGLEDALILATLLSTKNLQADQLTDVSAIYENLRLKRACRVQQTSRESGEIYECYSAQYPTFAAIGEHLEHRFDWLWQHDLEQDVAEAQQQLHQLGMATI

ST25 4190 cg56_0543

MKLVKLSFAGLALFASVQTFAAEQTTATPTTQASPSKFVEVPVANTDKAASEVAASEAAPAK

ST25 4190 cg56_0544

MNTVKRNRSPSIEKTEATKNSILKAALKKFLEVGFANTKIRDIADLAQVGVGTVYCYFKTKEDLFEGVINIMLNEEFMVVKADTLKPDQSVYEFLYKHFEQIIESIYSSGRDGISKLVIKEGQNFPQLKEIYFNKMVTPYLREFEKLATIANERKEISLTCTPAEFALLIVAPIWMGILYNGILQESESISINHIFYSNLYALFHSKVS

ST25 4190 cg56_0545

MQILRLSDYPQYKEMAAQWFSEKWQIPVEAYLESIQISIDQRHAIPQWYIVLNENKHLIAGAGVIDNDFHERKDLTPNLCALFVEENYRNQNIAKQILDFVREDLSNQGIQTLYLITDHTEFYEKCGWRFLMLVKDEEGEMVRMYVADTF

ST25 4190 cg56_0546

MLTRFLLPILGTTVLVTGCASSSQYPINESYGPEPKLPEPKTSLFPTVNIAPAQGWPSGVMPTPAEGLKVKAFAKGLEHPRWLYVLPNGDVLVAETDAPPKPEDSKGIKGKIMTFVMRRAGSSHPSANRISLLRDTNGDGIADQKTVFLKNLNSPFGMALVGNHLYVANTDSLMRFPYQEGETHITSTGTKVLDLPGGPLNHHWTKNVIANREGTKLYITVGSNSNVAENGLDQEKGRAQIMEFDIASGQSRPFATGLRNPNGMAWQPQSGKLWTVVNERDEIGSDLVPDYMTSVQDGAFYGWPYSYYGQHVDVRVKPQNPDMVARAIKPDYALGNHTASLGLAFYTAELMPQFRGGAFIGQHGSWNRKPHSGYKVIFVPFRSGQPSGPPQDILTGFLSEKGKAYGRPVGVAIDFSGAVLVADDVGNTIWRVSPVAETTYESPTQQSIRSSVTQP

ST25 4190 cg57_0548

MIDKSKSSLSEVLSQIKDGATILIGGFGTAGQPAELIDGLIELGVKDLTIVSNNAGNGDYGLAKLLKAGSIKKVICSFPRQSDSYVFDELYRAGKVELELVPQGNLACRIQAAGMGLGAVFTPTGFGTLLAEGKETRHIDGKDYVLEYPIKADFALIKAYKGDRWGNLVYRKSARNFGPIMAMAADVTIAQVSEVVELGGLDPEHIITPGIFVQHVVQVAPAQ

ST25 4190 cg57_0549

MNRQQIDALVKQMNVDTAKGEVDARVQQIVVRLLGDLFQAIEDLDIQPSEVWKGLEYFTDAGQANELGLLAAGLGLEHYLDLRADEADAKAGITGGTPRTIEGPLYVAGAPESVGFARMDDGTETGKIDTLIIEGTVTDTDGNIIENAKVEVWHANSLGNYSFFDKSQSDFNLRRTIFTDADGKYVALTTMPVGYGCPPEGTTQALLNKLGRHGNRPSHVHYFVSAPGYRKLTTQFNIEGDEYLWDDFAFATRDGLVATAVDVTDPAEIQRRGLDHAFKHITFNIELVKEAAAAPSTEVERRRASA

ST25 4190 cg57_0550

MLFHVRMDVHIPLDMPADKANEIKAVEKAYSQDLQRQGKWRHIWRITGQYSNISIFDVESNEELHNILQGLPLYPYMDIEVMALNRHPSSVRDDDS

ST25 4190 cg57_0551

MRIQTLVLVKITTTDGIVGWGEATTIGGLNYGEESPESVKANIDTYFAPLLTSVKDLNVAQTLKLIRKNINGNRFAKCAIQTALLDIQAKRLGVPLSEVLGGRLRNSLPVLWTLASGDTEKDIAEARKMIELKRHNTFKLKIGARPLQQDVDHVIAIKKALGADVSVRVDVNRAWSELECIHGIQQLQDGGIDLIEQPCAIQNTEALARLTQRFDVAIMADEALTGPDSAYRIAKNHGADVFAVKIEQSGGLIEACEVGKIAGLAGIDLYGGTMLEGPVGSIASAHVFATFETLAFGTELFGPLLLTEEILKEPLRYENFELHLPTAPGLGIEVDEDKIEKLRR

ST25 4190 cg57_0552

MELRHLRYFVTVVEEQSLTKAAEKLFIAQPPLTRQIKKLEEELGIDLFEKGSRPLKVTEAGLFFYQHAVQILTHTAQAASMAKKMKLVENVVKVGYVSSLLYGRLPQVIYLFRQKNPDIHVELIECGTRDQVEALKLGKIDLGFGRLPISDPAIKRLLLRKEKLKLAIHKKHPLSDFQESGIYLSQIINETIFSYPTTPKPNFFDHYSSFIYQIRSCSGQAD

ST25 4190 cg57_0553

MRKILGIASLIMSTGVVHAEQLNEQEQISPYSANVTFASQYISRGFQQTWGKPALQIGFDYANPNGLFVGTWASNVSSNYLRDASVEWDFYAGYLKTIDKFSIGMSVFYLLLPRCKKYG

ST25 4190 cg57_0554

VPQISYGPLSLKYFITYTPDYAGYNSNTMGGPEGKRSRGTTYLDLNFTQPINESWTFGAHYGYERIKNFSEANFQDMKVELIKDLGDGWTTGLAYTKAWDKDGYYRNYSNGEPDAPISNPIDSTFTVSVKKVF

ST25 4190 cg57_0555

MSKADYKNPIPSKSQHALIVQQLGLKIVSGEISENAKLPSEVDLCEEYKVSRPVFREAIRVLNAKGLTYSRPKIGTVVRPKEEWHLLDPDVLFWLIQTTPEHEFFKTLSTVRRVLEPELAYIAASTANEEDIERIKQAYEGMEKATTVEEFIEPDIQFHLAIAKATHNDLLAYMSKMLVLPLQQSIQVTSLRPNLQGHSLPRHKAILTAIENKDPLSARHASLVQLDDTKMAYDLIKK

ST25 4190 cg57_0556

MNNKQVLRSAAWFGTTDKNGFMYRSWMKNQGIPDHEFQGKPIIGICNTWSELTPCNAHFRKIAEHVKKGILEAGGYPVEFPVFSNGESNLRPTAMFTRNLASMDVEEAIRGNPIDGVVLLTGCDKTTPALLMGAASCDIPAIVVTGGPMLNGKHKGKDIGAGTIVWQMHEELKAGKIDLNEFLSAESGMSRSAGTCNTMGTASTMACMAEALGTSLPHNAAIPAVDSRRYVLAHLSGMRIVDMVHQDLRLSKILTKEAFENAIKVNAAIGGSTNAVIHLKAIAGRIGVDLQLDDWNRVGRGMPTIVDLQPSGRFLMEEFYYSGGLPAVIRRMGEASLLPHPQALTVNGQTIWENCQQSPIYNDEVIRKIDNPIRQDGGMCILRGNLAPKGAVLKPSAATPELMKHRGRAVVFENFDDYKSRINDPDLDVDETCILVMKNAGPKGYPGMAEVGNMGLPPKILAKGITDMVRISDARMSGTAYGTVVLHVAPEAMAGGPLAVVQNGDFIELDAYAGKLHLEVSDEELKQRLENLAPPAPPSFIGGYRKLYVEHVLQADEGCDFDFLVGCRGSEVPRHSH

ST25 4190 cg57_0557

MGADVGIGDAAYGLGAGLFFIGYFLFEVPSNLLLDKFGARKWFTRILLTWGLITMAMALIQGPKSFYLLRFLLGVAEAGFFPGVLYLITQWYPVRHRGKIMGMFVLSQPIAMMIAGPLASLLLGLDGIANLHGWQWLFVAVGLPAVLLALPTFLWLPDNIDKVKWLSIEQKQWLKNELVKDEAEYDQTRHANPLHALKDKRVLLLALYYLPVTLSIYGLNLWLPTIIKQFGGGSDIQIGFLSSIPYIFGIIGLLIIPRSTDRLNDRYGHLSFLYALGACAMFLSGWLNSPVMQLAALAVVAFCLFSSTAVFWTLPGRFLTGASAAAGIALINSVGNLGGYVGPFGIGLLKEYTGNMAAGLYFLSIVMLFGLILTYIVYAKLERQKTQTVNIQKPL

ST25 4190 cg57_0558

MQIIQFENRANQRAVAKVEGNMAYPVKDIQSVRDLALLAIRNKVSLEQQVEALGFESETYDYSSLLADLKVLPPLDHPDSTHCLISGTGLTHLGSASARDKMHQQNLSDDNSVTDTMRIFQWGLQKGRPAEGQIGAQPEWFYKGDGSIVVRPGAELPLPPFAEDGGEEPEIAGLYVIGEDLKPYRIGFALGNEYSDHVMERRNYLYLAHSKLRFCSFGPALRTGELPKHLVGTSRLRRDGQIIWEKEFLSGEDNMCHSLANLEYHHFKYQQFLKAGDVHIHYFGTATLSFADGIQAQVNDEFEIEMKEFGHPLKNKLAHMQPELPIGSVITL

ST25 4190 cg57_0559

MVIGHNFIGGSRSAQSSTLIKSIHATTGEALPYEFHHATEQEVNQACEAASQAFKTYRHTTPEQRAVFLENIADELDALGTDFLEIVSQETALPLARLQGERARTSGQMRLFAKVLRRGDFLGARIDTALPERQPLPRPDLRQIKIGVGPVAVFGASNFPLAFSTAGGDTASALAAGCSVVVKAHSGHMATADFVAQAIERAVEKSNMPKGVFNMIYGNGVGEPLVKHPLIQAVGFTGSLRGGRALCDMAAARPQTIPVFAEMSSINPMLMLPEALKNRGEKIAQDLADSVVLGCGQFCTNPGLILGIKSAEFSQLISNLTEIMGAKPAQTMLNAGTLKSYTAGLEHLTQHQGIKHLAGQTQQGNQAQPQLFKADVELLLAGDQLLQEEIFGPTTVIIEVKDKAQLIQALQSMNGQLTATLIADEADLTEFADVVPVLEEKAGRLLINGYPTGVEVCDAMVHGGPYPATSDARGTSVGTLAIDRYLRPVCYQNYPQSLLPEALKDSNPLQILRLVNGEMTKEAI

ST25 4190 cg57_0560

VLRKNAESSVEASSDREQEFLLLDRLYHFGIKVPEPLYFKKSPNFLNSDFFIMKKIEGVTEGHKLVRIAEEEKRKKITQDIGKQLALIHAIQSDDVLEKLLPKPDKDQYLENKLTDFLVQLDHLQRHRPILEYAIQWMFNNLPKVDDLVLVHGDYRIGNIMINDDQISGILDWEFTQWGDRREDLGWFTSKCWRFGQDENIAGGIGSYKDFAKAYAEISDIYIPEFEMKFWHVLSHVRWAIIAMQQSDRNNEMDTPSLELALTEFLVPRLEKNILEILGEKE

ST25 4190 cg57_0561

MSEQKIIDLIKASQAVIKNELLPQSGSQKYNLLMLMRSLEILQAYILQKDTCTLHRSGILQDYFSFPIKDIDEATQLFISDIREGKQSDQTFETLKALNLEELKITEPKVANHG

ST25 4190 cg57_0562

MDKYRLRIYLVRHGHVSYFDAANNPINPKFAQLSEQGIEQIQQLAHQLRDVSFEKIYSSTMPRSIQTAEILKRYQNEHKDIQSFDDIREIRAGRLREISLDRAELEIKKAYQFHKNNLELFVQGESWTHFISRVLTWFEGMILTANKDQNILISSHDIVNRILINWVYGHDFKDVYSQEQDYGCLNILDLTIENQKVISKRIKLQNFTPYNLIKNELFNSAMDDVYETYIATQGFKLKELSS

ST25 4190 cg57_0563

MITLSNELLELQQKVREFIQQEVIPLESDPRQDSHGPSEALRQELVSRARSWGLLTPHASREMGGLGWSHLQKAVAFEEAGYSALGPIALNIHAPDEGNIHLLDVVANDAQKQKWLKKLVAGEIRSCFAMTEPAPGAGSDPSMLQTTAIADGDDYIINGRKWLITGADGASVAIIMAKMEDGSASMFLTDTNVEGFILEKNMNAMDSCFSGGHGILRFENLRIPKENVLGEIGKGFKYAQVRLAPARLTHCMRWLGQARRAHDIATQYARERQSFGKRLGDHQGVGFMLADNEMDILTTRLAVHYCAQVLDLGEKGNYESSLVKVISSEGIWRVVDRSVQILGGQAMTDESVVCKIFKDARGFRIYDGANEVHRMSIAKKLLGKQA

ST25 4190 cg57_0564

MNIFDVKDKYILITGASSGLGHHIAELFAKEGANIVICARRLERLKELESHIKNEFGVKVYTFALDVNDRSAVKDMLSSLEAEGVTVDVLINNAGVSDTKRFLDYNDEDWDKIVDTNLKAPWQCAQEVVQHMIKAERKGSIINITSILSQSTNLGVSPYCASKAGLRHLTEVMAVELARFGINVNAIAPGYMITEINEEYLTSEAGQQLLKKIPTRKFVEFDDLNGPLLLLASQAGQGITGIEIKVDGGHSAAPI

ST25 4190 cg57_0565

MLNRKPINILVTYIKKPNINGFRSLVEQAISEVESNEIDLVECHISDVQDVAQSLVENGCQILLCTGATASFLQKKLNIDIQVIRTGTFDVIQAISNLKQYKRIALVGNTSTVDLENYSDIFALDIQQFNYDSYLDAKKTIHLIKNQGFDAIIGSPVAVELALNENLVGHLAISVPSLKDLLQNALRTLEKIRREQYSTLRLTEIFNHLNEGICFITKQKKISLINNSMSSLLEKDSSDVLNKSALEIFEGLNFDIDQENSHQLVQFKNLKLAVYISKLKNDYIDGYILRVQELNALEQSNSQFRKVSSKNFNTRYTFDQILTQNPNFQQIIKLSKAYAKTDSTILITGESGTGKEVLAQSIHNHSSRYKAPFVAINCASFPESLLESELFGYEEGAFTGAKKNGRIGLIEAAHTGTLFLDEIGDMPLHLQTRFLRVLQERQINRLGSVVSHHLDIRVIAATHANLEELVRNGTFRADLYYRLNILRVYTPSLKERKEDILYLANSFLEKYRKSLDDLPNILQKAFLHYSWPGNIRELENIVERINVLLQLDHEILTPEFLKQQLPELFQSQNHYTAQPLLKEVKVNQELSLIEQTLAEVDGDLDLAAQKLGISRTTLWRRMKLINN

ST25 4190 cg57_0566

MQHALTVYADQEIVTLKADGITKHRYTYKDAFERVAQFANALDRLNISSDAKVGTMAWNSYQHFELHYAIPCTGRIYHTINPKLAPEQLIQIINSAEDEVLIIEPDCLALVDSIYDNIKPVIKHFIVLGDPNKNLKAQFDFAFYEELIALEQSYYDWPDIPEERASGLCYTSGTTGDPKGVLYSHRSTVLHALILAMPNAIGLTHDSCIMPLVPLYHISAWGMPFNAVLSGAKIVWPHSFAGQTEKIFNLIQSEHVDISMAVPTIWNSFKNYLEDHHISSVSLKRAISGGSAAPYSLIESLSHYGISVENAWGMTETSSMAVCNRVNQIKNNVETQSIKCGKPIFGVQMRLRDENHQLLPHDGMHEGILEVRGHTIAKQYINQTKAVEEDKWFDTGDIACIDEYGYMHITDRAKDMIKSGGEWVSSVEVENAAMGYEKVAEAAVIAANHPKWGERPLLILVPKSPQEKIEHSEIVVFLSSKLHKWAIPSATILVEEIPHTPTGKISKKILRERYHDYFTNSAFAECK

ST25 4190 cg57_0567

MNIPQEANIVLDAKFKKMVKQRNRFAVILSLIVLSIYFIFIGTATFHPELLAIPLEASKVTIGLPIAAIVIVLSWIITGFYIFITNQYFDKQKEKLRKEYRYE

ST25 4190 cg57_0568

MNKYFAFTILTLFSSGAWASQSEPRNWTAIIMFACVVGISLLITFKAAKKTVSKEDFYTAGNAISAKQNGLAIAGDYMSASTLLGISSMVFFKGYDGFIYVTGFFVCWPILTFLMAERLRNLGKYTFADIVSYRLDPQRTRILATCGSLIVVCCYLILQMVGAGQLIKLLFGLDYKIAVMLVGCLMLVYVIFGGMLATTWIQIVKAILLLFGGTVLVFLAFKAFGFSLNNMAEAAVNAHKLGQKVLEPGPMLSNPINTLSMSIGLIFGLAGLPHILMRFFTVPNAAEARRSVFYASGYIGYFFIVVCILGLASIAIVGTNPQYFVDGQLGGDLIGGSNMVAMHLAKALGGNLLLGFLSAVAFATILAVVAGLALAGASAISHDLFQKVIKKGKATEKQEMVVSRLSVVVLGVIAIACGILFEKMNIAFLMGLTFGIAASANFPVLILSMYWKDLTTRGAIFGGFVGIISAITFVVLSPTVWVGVLGHEKAIFPFDNPALFSMPLAFLVSIVVSLTDKSERAKLDKQGFELQVVRSELGAQQGSFEKMPLH

ST25 4190 cg57_0569

MKCITFNGAGGPEVITVSERPMPELTDDGILIEVVAAGINGPDVMQRKGLYAPPPGVTDIPGLEVSGIVKQVGKQVTKFKAGDEVCALVPGGGYAEYVVAHQDNVLFIPEGLTLIEGGILLETFMTVWSHLFQHAKFRPNSSILIHGGTSGIGTTATMLTRAFGATQIFTTVSSKEHQEASLKIGADVAINYNEDDFVEVVKKHTDGKGVDFVLDIIAGDYIQRNYEVAAMNGTILQIGVLKGLADKVNIFPMLAKRLTHTGATLRSQSAEAKAEVMAALQEKVWPLISKGQIKPLLYKTFDLEEARQAHEFLDQGQHVGKLALIVKK

ST25 4190 cg57_0570

MNTLNEVSSTLNASEILSRQRKAFLKAEPPTLQQRKASLAKLRAAVIEYRSELQVAASEDFGHRSYHETDVMELVGIVQSIDYLTRNLRRFMKPEKRHVSLFYRSASAHIEYQPKGVIGVMAPWNYPISLTLIPLATAIAAGNRAMLKPSELTPRISEVISRMLAANFSIEEVAVILGGPEVGAEFSALPFDHLLFTGSTPVGRKVMKAASDNLVPLTLELGGKSPAIVGRGHVNSRTMTSIAYGKLSNGGQTCVAPDYALVHEDDLEAFIEEYNATVSRFYPNGPDCDDYTSIVNERHYARLQGLLDSAKQNGARIIELGVKPESAKNRARTLVPTLVVGAKDDDAIMKEEIFGPILPVRTYRDIDEVINYINERDRPLALYYFGEKDADCKKLLRRTTSGNVGINNTLIHVAQDDLPFGGIGPSGMGAYHGIEGFRSMSHAKGIFDQGRWNFPSFLHAPFGKFMELALAITLGKKKARS

ST25 4190 cg57_0571

MNTNKAFTEVEFGQQKVKVPKGGYYDRFRMNPDLDEVAKDPAAGNIDFFRSIPKKLVESRVGPVWAPNFYYRSGNVQVLMLAPVKLLKKKLPSPLVPLEAFPGYGLVALTFFTYTVCDNDPYNEASVAIVVRKPKAHGPHALELINSIRKRHFYAHVLALPVDTEIARVRGVYGYQLPKWLAEIDVKIDKKKVEATLSTYNKLDISLTTATPSLKRVKSESRMGKATMLHLVDNEWHQTLVQTNVLSFGEKLFPRKVKVTRHGGHVSQLLDQLGASTILRLDVIEDAQVVLNLPTKL

ST25 4190 cg57_0572

MGYVTTKDGVDIFYKDWGPRDAPVIFFHHGWPLSSDDWDAQMLFFLKKVFV

ST25 4190 cg57_0573

MDHYADDVAAVVEHLGVQGAVHVGHSTGGGEVAHYVARYPNDPVAKAVLISAVPPLMVKTESNPDGLPKEVFDDLQNQLFKNRSQFYHDVPAGPFYGYNRPGAKVSEPVVLNWWRQGMMGGAKAHYDGIVAFSQTDFTEDLKKIEVPVLILHGEDDQVVPFEISGKKSAELVKNGTLISYPGFPHGMPTTEAETINKDLLAFIRS

ST25 4190 cg57_0574

MNDDTKKCPFSQLTTDFGAPVVDNQNSMTAGARGPLLAQDLWLNEKLANFVREVIPERRMHAKGSGAFGTFTVTHDITQYTRAKIFSEIGKKQK

ST25 4190 cg57_0575

MVGNNTPVFFLRDPRKFPDLNKAVKRDPKTNLRSATNNWDFWTLLPEALHQVTIVMSDRGIPASYRHMHGFSSHTYSFINSANERFWVKFHFRTQQGIKNLTDAEAGELVGHDRESHQRDLFDAIERKDFPKWTLYVQVMPEQDAEKVPYHPFDLTKVWPHGDYPLIEVGEFELNRNPENFFLDVEQSAFAPSNLVPGISASPDRMLQARLFNYADAQRYRLGVNYQQIPVNAARCPVHSNHRDGQGRIDANYGGLPHYEPNSFGQWQEQPQYKEPPLKINGDADFWNYREDDNDYFSQPRALFELMTPEQQQALFDNTARAMGDALDFIKYRHIRNCYACHPAYGEGVAKALGLTVENAQAARETDPARGLPSFI

ST25 4190 cg57_0576

LSFSAQAVETNSSSVQQPSNSSTSQETTVVEFGPYKVNVPKGGYYDRFRMNPNLDEVAKDPAAGNIDYFRTIPKKLVDTRVGKVWSPNFYYRTSNIQLLMLAPISKVKAKLPAPLEPLQPIPGYGLVSLTFFSYAVGDVDPYDEVSVAVVVRQPGAHHFNTTELLSSMRNHKYYGHVLALPVNTEIARVRGVYGYQLPKWLTPIDMKIGDKSVQAHIYNTDGKPDLSVAAPLPEIKTVKPQSRIETKTMYQLVDGKWHSTSVESNTLAFGQKLFPKNVQVVKSGGPLSKLLDELGANKVLRFDVVKDAQLALNMPVPYPSLDKSTAKKIILKSPFYLL

ST25 4190 cg57_0577

VSKVWSFQRWLIAFFMAFLLVSYANASDQVQNQETAKSKGKILVVMTNHSAYPTRSDKTGLWLTELTHFYDVAKVAGYDMDFVSPQGGVVPIDERSMKPIYVDKSARKLLADRQFMYRLNHTLAPSSIDPTHYKAIYYTGGHGTMWDFPDNKELKNISETIYRQGGIVSAVCHGVGGLLPLVGEDGKPLISGRTVTGFANKEETLSGIKSQVPFSLQNSLINHGAKYKQGFLPFTSYVVSDDRIITGQNPQSSKEIAEAVVKRLSTLK

ST25 4190 cg57_0578

VGVQAFLDVLNSGNGKPMELMTPTEARQVLIDAQKGAKLPPAQVSDKTIQINGQSIQLKIVKPENAKSVLPVFMFFHGGGWVLGDFATHERLIRDLVRESGAAAVYVNYTPSPEAHFPVAINQSYEATKWVAKHGKEIGVDGSRLALVGNSVGGNMVAAVALQIKQSGGPKVRYNVMLWPVTNAGFDNASYNQYKKGYFLTKNMMKWFWDNYTTNANDRNNILASPLRATSEQLKDFPPTLIQTAELDVLRDEGEAFGRNLDKAGVPVTVTRYNGLIHDYGLLNPLSNEPSVKTALSQAGSELQKHLK

ST25 4190 cg57_0579

LIASAETAVVKRADVNKAVAIFMVKTHFKLKI

ST25 4190 cg57_0580

MKENKILGDQLCFSLYSVANALTRQYRPLLKDFDLTYPQFIVLLALYEEDDISLKELSEKTLFDSGTLTPLVQKLEAKEFLKRVSIKEDERVKKVILTDKALEIKEKVIDLPNQLRCSMHMNDEELTMLRKLSLKLLEDL

ST25 4190 cg58_0581

MNKHVEAEWRFPAKTAWAIFAAMIFGNFMAILDIQIVASSLNEVQAGMSASRYEVTWVQTVYLIAEIIAIPMSSIVSRVLSTRVYYTMCAIGFTVSSLLCALSWNLESLLVFRGIQGFMGGGMIPTSMTALYLLFPEAKRSLPLVMFGMISTLGPAIGPTIGGWLTNNFSWHWMFLINIIPGIIIATVIYSGPNIDRANYSLIKSMDWFSLIGMAMFLGGLEYFLDEGARHDWLADTGVRIAFMVCVVGGMIFFSRSFTQPKPLLDLSVFKNKNFTLSAITTFVIGMALYGLGYMIPVFLGQVREMNSSQIGHVMMVTGIVMFCFAPFLAWLIPNFDTRKTVFVGMILAGFGVWLNSHLSIHSDYDFMFWPQIYRGIGLMICLIVVSHLAMSTLPLSKVADASGIYNLMRNIGGAVGLALINSSLDWLTAMHVTQINQSMTPQNWIFTERLDRLTAQYQEVGANAQQIALSVIYRDIHFQALTSSFNDLLRMLAIIMFVTAFLTIFMDRGKKMNM

ST25 4190 cg58_0582

MIMDNVAQLETDTNFQSRKKITWGVFSVLLLFLVAGILYYFFVYRFYQSTDNAYVQADVTWVMPKISGEVMELLINDNQVVKKGETLAVLDHRDYQARYDQARSVVSLKEAALGVQQQNEKSAKSSISEANSGVVAAQADLTRLRKEFERYQDLLKDGVITRQNFEGVQSQYLTAQAQLSKAQAAVNAAEAQLGSLQASRAQLLADIQSANANLNLYQVDLASSKVVSPVSGKIGSLAIQKGSRVSPQTRLMAIIPENSLYVQANFKETQIEKMHIGQKVKLKLDAYPSLNFTGKIESFSPASGATFSLMPPDNATGNFNKVVQRIPVRIAIDSSPHIDLIKPGMSVSATVDLRT

ST25 4190 cg58_0583

VEYLTSLHEIYPVRHEFAGYPAAMTLADRVHSDHDIAPLEASKSYPDSIEKVLHFSGKARDIQDFERFLEQAKSANIQNLLLLTGDKLKEHHNGRDGQPRSRYLESVNAVMAAKHHGGFRIGVAFNPFKYVEAERDAQYLKLHKKIKAGADFIITQLGYDIEALKKAKSFLIKNNYSQQILACVMPLTLGRANFMVKHKVAGIVITPHMLKVLAEEKQAGHTDRVYLRCALQILICKHLGFAGIHLSACHKPEEQMLLESYIEQYRHLELKALEELWNSLWQVTSGKEFSPEITHFSCQPTSKQLIKYRQLHVMHEAMFGSKIAKGVGRFIFKASFWENALIAKLLLKTEVLSKHSLVGCESCGQCRLGDTLYICPETCPKGLANGPCGGTTLDRCEFGDRECIHSVKARLAKAVKQTEILKDKLIPTVPLETRGTSSWKNWYLATEA

ST25 4190 cg58_0584

VTIQTEQEFLASYDRRDYDAPLLTVDMAIFSVFNGQLQVLLIKRPNFPAKDQWALPGGFADLKHDQDLMATAYRKLVEKTGISSPYLEQVASVGNAKRDPRGWAVTILYFALIDFNAYQHQALAEYSEWIPVAKAQDLALAFDHNELLSLALERLTSKTRYTALPASLMPELFTLTELQTIYEIILGQSLDKKAFRRRMIEAGAVEETNHSKIVGKRPAQLYRYALDSFDFIFPRSLELPRNKESEDKQNNELSD

ST25 4190 cg58_0585

MTIKLNPLNAIDFYKADHRRQYPAGTEYVYANFTPRSSRLAKMLPDFDDKVVLFGLQGFIKHFLIDTWNEGFFKQPKDKVVAAYKRRMDSSLGEGAVPVDHIEALHDLGYLPLRIKALPEGSRVNMRVPVLTVINTDPRFFWLTNYIETVLSAELWKSCTTATIAYEYKRLLTQYAVKTGAPLDFVPVQGHDFSSRGMSGIYDAAQSGVGHLTSFIGTDSVASIDYAEEYYNATGVIGVSVPATEHSVMCMGTEDSELETFKRLICELYPSGVVSIVSDTWDFWRVITEFTVALKPEILARQPNALGLAKLVFRPDSGDPVKIICGDPDAKVGSPAYKGAVECLWEVFGGTTTDQGYKVLNERVGLIYGDSITLDRAQRILEGLEAKGFASNNLVFGIGSFTYNYLTRDTFGFAVKATWGQVNGVGRELFKDPITDSGVKKSAKGLLRIEESENGFTLFDEQTAEQEQGGALKTVFENGKLQYECTLDQIRERLSIA

ST25 4190 cg58_0586

MAIQLLDAARNEIPVKFTQFSGGERHVQIDEATLGSLSGNVLVRAKMASSHDVMDYLLLENILLTQGLTVDLEIPYFPYARQDRICAVGQAFSLDVMTKLLNINADKKAGKQGKVTVWDCHSEVTTTLLAANTSFSEVVNISSVDIIAKSEALSTLLKDEKTVLVCPDKGAKARTQMVADAFNSKRKQPITIIQCDKKRDPVTGKILGTHVHATDLSGLTAVITDDICDGGATFIGIAKELRRLNCHKVVLYVTHGIFSKGIEVFDGLLDQLFTSDSFPQQPSDKISVIAFVAE

ST25 4190 cg58_0587

MLYELFLFGLLSGITTWLFGFGGGFVAVPLLYTVIIQKWSNESNIGIHAMQIAVATSAFVMLCSASFATFRHYRSGHIDWQKIRFLGGGIALGGIVGAVMASLFNGNWLRWIFMGYVFITILDCYYRPGFIVTSRQKQHYGQNSELIKGGIIGWVAALLGVGGSVMTVPLLRRRGSSMAEAAAIANILTLPLSFTATLTYCVLSLWQSTPNGFIGLIWFEAALFLVAGTWIGLYFSEKFISKLPDLWRAKLYPLLLIIVLLVIFFVN

ST25 4190 cg58_0588

LLDTHSHRRAQFLYAPEGLMKVETEDGQWLVLPYSGVWIPAGKPHRVWLSKVSTYSLYIEVSNAPRQANYCEVIQVSPLLHQLLIQANKLSIDYSRSGRDGALIDLLLYELEAAPALPLFIPLPRNTLLSKQCAEFMQHPDIHSSPKDWAHDHNKSERTFHRWFKSETGMSFQAWRNRVCIIYALNALKENISITEIAFSLGYEHSAAFTAMFSKIMGYSPSQFQKRFHTHDKAL

ST25 4190 cg58_0589

MTLYYQLVGKSDYTSAVEFALYTRELLFPEIYHGQVPNDLQNFEQHYVHDPLGCFITVKDQNRIIGTIAYRAYDHRFDLNLPSNTVEVVKLFVLPEYRRKGIATQLCDMLFSHAKNSEITTLYLHTHPFLPAAEEFWRLQGFEVIQREWIDTYDTIHMSKSL

ST25 4190 cg58_0590

MIQIDQLNYAYGHKQVLKNIHLEFPENQFSVILGRNGCGKSTLFKLMAGLEPVKDGLIRYSGKPLSDFKGKDRAALLGFLPQFHKTVFPFLVKDVVITGRAAFSRYRPSKSDWECVDQALLDLDIEHLRDRPYTELSGGERQLVMIARILVQAPKVILLDEPTNHLDVYYQSYLMKKLRQLSRQNFTVIAIMHDPNLAFLFADHLFFMRQHEVVKPESKTRITDPKFLKSVYDVEFHEAMIQDKTIVIPDHSWL

ST25 4190 cg58_0591

MEKLKYYWFYPLPFIMIFISLLIGPTQTLSAWDYLLWGVQAVFGTPYFDAEQFRLMQNILVNVRLPRILLTFMVGAALATAGNGLQALFRNPLVDSYVLGISSGAAFGAALALSLSWLSPNLSAFIFGVCAVGLTYLFAHQKHESQTSLVMVILSGMIVSGLFLAGLTVIQYLSDPFKLQAIVQWTMGNLHQASWQKVQYAVLPICIGLAGLFAMRWRLNLMALGAEEAQAVGVNPRWEQLLLIALVTVCTSTSVAAAGIISLYGLFMPHIVRMLVGPDHRYSIPANMVLGGSFLLLIDNFSRVLLTFEIPIGIFTMLLGAPFFLLLMKTQRTHWA

ST25 4190 cg58_0592

LFESGLDGLYMLGQGDKVIGIPAEVYIQPLLFNAYSKIDKRIANKQLAAPSQGANATNIESLVLLKPDLVIVGSGQTQTIELLRQFGIAVYVMESGTYKQVKEELSEIAILSGAQKRAQEILNFSDEIVAEVAAKTARQPNKQSIYYAWSGGRIFSTSGRESITNDFIELAGAYNIVQTAANQPNVNPETLIEWNPDNIVLWNTNPKLIYERKELQGLSAVQNRKVFNLSPAFIYNPHTIKIIITAIYLNHGIYPEQSDLPVSTLQQRILTKLYGEQLAKALVQ

ST25 4190 cg58_0593

MTTEVLGYAALSSETPLVPFKFERRTPREDDVVIQIEYCGVCHSDLHQAKNDWGFSAYPLVPGHEIVGRVTSIGPKVTKYKVGDLVGIGCMVDSCRTCSACHSGLEQYCEEGNIQTYGGVDRHDQRPTYGGYSQSITCSQDFVLKVPENLDAQAVPPLLCAGITTWSPLRHWNVGKGSKVAVVGLGGLGHMAIKLANALGAEVTLFTRSANKEQDAKQLGAHHVVLSTDENQMKSVLNQFDLIVDTVPYNHDLKPYIPTLALNGTIVLVGYLGEISANSVPMILGRKSIAGSVIGGIKETQELLDFCGEHNIVSEVEMINMQNINEAFERMLKSDVKYRFVIDMKTLSED

ST25 4190 cg58_0594

MQNSLSEQQQAIIPIAAYAANGDLAKLSQSLKDGLDLGLTIAEIKEVLVQTYAYAGFPRSLNALTTFMQVLEQRKESGIRDIEGKTNSTLPKDYQALLQGTQNQTQLVGQPVKGALFEFAPAIDEYLKAHLFGDIFSRDVLNWQDREIATLSMLSALEGVDSQLQSHLNIAKTQGVSSHQFENIQKILAEKVSLQVGQRFQNVLTSFHNQSL

ST25 4190 cg58_0595

MNVKAVTFVTSLFISMGVPAMDKKSVTAFQTVKKANQQQVITGLDNIFTGQVAIKPLTDVTDSINASSAYVSFNANARSFWHTHPKGQYLIVTEGEGLVQEWGKPAEKISVGDVIYCPPGVKHWHGASGNSAMTHLALTATDEHGKNVDWLEPVSDEQYKEAGH

ST25 4190 cg58_0596

LAQLVDQWTREKGTFETAITGLTLYRAETLTKPSSSMMDASLCMIAQGKKQVILSEETYTYDSNHFLFTAIDLPVIAQVLEASVEQPYLSIVLRLDPYLLAQIMLEAHIPFKDVNTEKKGMAVGVVNSELNDAFIRLIKLLDTPQDIPILSPLIIKEIFYRLLMSPQGDRLKRIVAAGTTGHRIVKAIEWLKTNFAKPFSIEELASTMGMSASSFHQHFRDITSMSPLQYQKRMRLTEARRLLMTEEYDISSTSMQVGYESLSQFSREYKRFFGVSPSVDIKNI

ST25 4190 cg58_0597

MARIGFVGTGIMGMPMAMNLLKAGHQVKVWNRTSSKAESLKEAGAHVCSELEQVGKDVEFLICMLSDGKTCDEILFQERGAISQLKPESTVIVMSSIPVEFAKTQSEKCKERGLRYLDAPVSGGEKGAQNASLAIMVGGDAQTFSHAEHVLSAMGRPILVGEAGCGMLAKLVNQMIVASTIATVSEGLLLASKAGADPIKLKQALTGGFADSPILQQHGERMLNRDFKPGGTARNQHKDIHTAVSYAKSLELNLPIAQKVSQLFENMLAAGDGELDHSGLIRELERMNHV

ST25 4190 cg58_0598

MEDIKNVTALEEQTIKRNFKYALSRFLLFYFIMAFLDRTNIGFAALHMNDAIGITQTIFGLGAGVFFLGYFIAEVPSNVLLHRFGARIWIARIMITWGIIAGLMGFIHSGTQFIILRFLLGIAEAGFFPGVIFYLTLWFPAKYRARVFATFYLGLPIAQIIGAPISVGLMQWGNTIGYEGWRLMYVLEGIPSIILGLVCLKYLTNNPKEAQWLTAEQRQWLMNTLEREEREKEQSADAALTKGELIKQVFKNPLVWIMAIVYFGITSGSNAMFFFLPSVLESFRNTFGMQISLIQNGLLTAIPYAFAAVGMVLWSRRSDRKQERYKHGACAALMAAFAIAIALIVNQPWAIIVGFILLAIGVFSAINIFWTIPAQTLTGVGAAAGIGLINSVGNLSGFTGPYLTGYLYTTTGTYTVAFLAIAGFVAMGGLGLLLLAKLKSDSLKVEQQRLKVRTATEMK

ST25 4190 cg58_0599

MNVLITGGTGFIGKQIAKEILKTGSLTLDGKQAKPIDKIILFDAFAGDDLPQDPKIEVVIGDITDKTTVANITEKIDVVWHLAAVVSSAAEADFDLGMDVNLYGLLNLLEELRKKQTTPRVIFASGCAVFGGQLPEVVTDDTVVTPKSSYGMQKAVGELLVSDYSRKGFIDGRVLRLPTIVVRPGKPNKAASTFFSSIIREPLKGETAVCPVPPDTPVFITSPRRCVESMIKAASISSDALQDNRIIPLPGLTVTVKQMLEALEKVAGKQATDLVQWQEDKTIQRIVQSWPVQVKAEYAESLGFQADENFESIIQAHIEDTQN

ST25 4190 cg58_0600

MADLTYSSLNNWLKFKHLVLLETLARTNNMHLAAEQMNLSQPAVSKMLKEIEGLLGFQVFERLPRNMPVTALGEHVIRYAQRVLNDAKHFVEDIEILRLGGHGFLKVGGIFAATAVVIPNSIIEIKKQWPLLSIDVVEQTSDHLMEMLSEHTLDLAIGRFTDVTQSQFFDFQPLGPEPFCIVVNNAHPCAQKKFCTLEELLKWPWVLYPKGTPIRERMEGAFARAKVKIPLNTVNTMSMQTFLQILKGAPMVGMLPEAMVIDQVKEGQLQILETDLILDAQDYGILTRKDEPVSDIAAAFINILLKNAKRK

ST25 4190 cg58_0601

MNFTLNSQNSLPDDATQGCLIGRAWIPSQISGPSPIILRGNQVFDISEKFHTISELLESADPLKALSEIEGRRVGSIDELFANTVAEPDTDKAYFLAPIDLQVIKAAGVTFAASMLERVIEEQAGGDAQKAQSIREVVQGVIGDNLKTIEPGSEKALQLKEYLIEQKMWSQYLEVGIGTDAEIFTKAPVLAAVGTGQNIGIHPKSEWNNPEPEVVLVANSQGKILGATLGNDVNLRDFEGRSALLLSKAKDNNASCAIGPFIRLFDHTFTLNDIRTCDVELQIQGTDNFVLNGVSSMSQISRDPEDLIQQTLNENHQYPDGFVLFLGTLFAPTQDREQAGAGFTHKVGDVVRIHSPKLGTLYNTVMTSDKATPWNFGINALMRNLKQRELL

ST25 4190 cg58_0602

MNDQNKRIFLRSQEWFDDPEHADMTALYVERYMNYGLTRAELQSGRPIIGIAQTGSDLTPCNRHHKELAERVKAGIRDAGGIPMEFPVHPIAEQTRRPTAALDRNLAYLGLVEILHGYPLDGVVLTTGCDKTTPACLMAAATTDIPAIVLSGGPMLDGHFKGELIGSGTVLWHARNLLATGEIDYEGFMEMTTSASPSVGHCNTMGTALSMNALAEALGMSLPTCASIPAPYRERGQMAYMTGKRICEMVLEDLRPSKIMTKQSFENAIVVASALGASSNCPPHLIAIARHMGIELS

ST25 4190 cg58_0603

VGENIPLIVNCMPAGKYLGEGFHRAGGVPAVLHELQKAGVLHEDCASVSGKTIGEIAKNAKTSNADVIFPYEQPLKHGAGFIVLSGNFFDSAIMKMSVVGEAFKKTYLSDPNNENSFEARAIVFEGPEDYHARINDPALNIDEHCILVIRGAGTVGYPGSAEVVNMAPPAELIKKGIDSLPCLGDGRQSGTSASPSILNMSPEAAVGGGIALLKTNDRLRIDLNKRSVNVLISDEELEQRRREWKPTVSPSQTPWQEMYRNMVGQLSTGGCLEPATLYMRVVNQDNLPRHSH

ST25 4190 cg58_0604

MTIIGHNFIGGSRSAQGTTLLKSIHATTGEALPYEFHHATEQEINQACEAASQAFKTYRHTSPEQRAIFLENIADELDALGIDFLEIVSQETALPLARLQGERARTSGQMRLFAKVLRRGDFLGARIDTALPERQPLPRPDLRQIKIGVGPVAVFGASNFPLAFSTAGGDTASALAAGCSVVVKAHSGHMATADFVAQAIERAVEKSNMPKGVFNMIYGNGVGEPLVKHPLIQAVGFTGSLRGGRALCDMAAARPQPIPVFAEMSSINPMLMLPEALKNRGEKIAQDLADSVVLGCGQFCTNPGLILGIKSAEFNQLISNLTEIMGAKPAQTMLNAGTLKSYTAGLEHLTQHQGIKHLAGNTQQGNQAQPQLFKADVELLLAGDQLLQEEIFGPTTVIIEVEDKAQLIQALQSMNGQLTATLIADEADLTEFADVVPVLEEKAGRLLINGYPTGVEVCDAMVHGGPYPATSDARGTSVGTLAIDRYLRPVCYQNYPPSLLPEALKDSNPLQILRLVNGEMTKEAI

ST25 4190 cg58_0605

MSDCTKTNEKDQKILDAATKFFLIHGFSGTTTDMIQKEAGVSKATMYGCFKNKEAMFAAVIERQCTNMQKQIMSVETKAKNLRSALTEIGKTYLCFILSHSGLAFFRVCIAEAVRFPELSEKFFEVGPQRLANIIAGYLEKSIKQGEIELTSSSEVAANIFLSLLRSDAHLKCLTHPDYLISADEISTWVEYAVDLFLKNINYKLD

ST25 4190 cg58_0606

MNIRPLIGLAGAIFAAMTVEFNNRISSITLVDIRGEMGISVDSGYWVSSIYASAMIIGMILSTSWAVIFSMRRVLLFAIGLCLFSSVLIPFSPNIEIFYLLRGLQGLANGLTIPLLMACALRFLGPEIRLWGLACYALTATFFPNLSAALAAFYLDVIGWKMIFFQTIPFCALSAALVYFGIPQDPLNYSRIKTYDWTGAILAIIGLATLSTMLLHGNHLDWFHSKLICVLALISAITLPLFLIHEWRYPTPLIKPQMLEIRNFGYAVIALFCFVVIGMSTSTLPLNYLSAVHGYKPTQTMWIGLQIAALQFIYIPIVIKVLNQAWVDSRYVHGFGLLLVMVGCLGASQLDTTWNQDQFYFWHAISCLGQTCVVLSLLMMGTNSVHPTMAIYASPMINTPRAISGVLGVCLLDWVNRVRGEYHSTRLIENTAQHVFQNIQGPVINPLTPPVLSADGTERVSGGLSALNSAIQAQQSVLVISDQYLILAGLTAILFIVMLILPVRTYPPRIALIKLMNSHNSGKSL

ST25 4190 cg58_0607

MVIACIIVLVSILWILKVIFLPSSIVKTDDARVDVEYSTIAPKVSGNIEEIYIKDHQTVKKGQLLARIDARDYQAALAEAESNYAKAQADLNEAMLAVERQPTVIRETEAQLRKVEAGIKLTKDNTARYEQLQALGAESRLITQQSKTTLTEQYADLDSSKEKVIDAQYQLNQYKIQVQAKQAALKQAQAALDKAKLNLSYTEIRAPIDGMIGQKSANVGNFVGAGNPLMVVVPLDQVYVEANFREIELKQIKIGQPVTVYVDAYNVELKGVVDSFSPSTGAFFSPISATNATGNFTKIVQRLPLRIKLLENQPDIKLLRPGLSVVVSVDTTK

ST25 4190 cg58_0608

MKYLKIEYLKFSTLLFICSGHSILWAADTTQQDEDEWKFTLKNAYINRDFDNDAIKDTGSWSQAASLFYKSKMHDTPLQIADKPITIGADASVQYAVRLSSDKHVADTVLPFNKETQSQASDFLKYGATLKLGYDKTLLSVGELWLDLPVTAVDASRQLLTSYWGTNLKSQLSDQLYAEIGRVEKVSPRNEEDFKKFSFTANGITKESDGLNYIDLRYQFTPSLKAEYYFGNLEDLYNKHYVGLEHNWKQSNFALTSKFKYFNAKDDGNTFDIDAQNIGLLETVKVKNHTFGLGYQQIIGESAYPLPDGFLPETYFINWNATGFFKEDEKSYHVMYGYDFKDYVSGLNAMVKYVYGHDFKAANGEKNHETESNVILNYTFQQPFLKGVALQYIRIDYNVKHGNDFGEDRFFVNYTKKF

ST25 4190 cg58_0609

LVPTVNEVEKSTLKDLFNKGFALGTFLIWFTFFMSLLIIYMISSWMPTLLTNEGFNLSNASWLTSIFQIGGTIGAIVLGLLMDKMDATKILSTAYVFGGFFLICLGFGIEQANAVLLMFAMFGVGAGISGSQVGANAFASSFYPTHCRATGVSWANAVGRSGSIVGSIMGGWLMSLNLSSFEILSILAIPAFCAAVSLLLIKRLKKNQSQVLVASN

ST25 4190 cg58_0610

MWLCFLIVAIDGFDTAAVGFIAPALKAEWGLQATDLAPLFGAGLFGLMAGALIFGPLSDKLGRKPILIGSVIMFGIASVFASFSPDLQTLIIWRFLTGLGLGGALPNAITLTSEYAPTSRRSNLVTMMFCGFTIGSALGGIFSAQLLPHIGWHGILLIGGVLPLATVPFLYFLLPESLRFLVLKKKSPEKIERLFTVSHRI

ST25 4190 cg58_0611

MSFIFEPMTTAGLFIHGSEHKFPVRRIYCVGRNYAAHAREMGFDPDREPPFFFCKPNDLESVVPVNTDAVDIPYPSETSNFHYEIELVVAIGKNAKNVSVEDAVNYIYGYAVGLDMTRRDLQMAMREKGRPWEIGKAFDFSAPVGPIHPITQTGEINNADIHLTVNGKTEQHSDVSHLIWSVAETIANLSTLFELKAGDLIFTGTPEGVGVVVKGDVMRADITGLTGITVKVK

ST25 4190 cg58_0612

MKLYSFFRSGTSHRTRIVLNLKGLAYEATYISLAKNEHHQAAFKALNPQGFVPVLETESGNLLQSPAIIEWLEEQYPEPALLPTDVLGREKVRAIAALVGCDVHPLNNKRVLEYLRQNLGLDDTQINAWCAKWIQDGFTALEHILEQDQNREKFCYGKQPTIADAYLVPQVVSAERFKVDLSVYPNINEIYQHCMTLEAFQKAAPEQQADAF

ST25 4190 cg58_0613

MSNTQQPILIAGGGIGGFAAALALAKQGFKVQVFEQAPEIGEIGAGIQLGPNAFHAFDALGIGEIARSKAVYTDYMVMHDAIDEYQVGKIPTDEKFRERFGNPYAVIHRADIHGSLVEGAKQYGNLEIITNCHIQKVDQDDAGVTITDQNGKQYHGQALIGADGVKSVVRDTYVGDPALVTGHVVYRAVVPESEFPEDLKWNAASIWVGPNCHLVHYPLRGGKEYNVVVTFHSREQEQWGVTDGSKEEVLSYFQEICQKARQLIELPKSWRRWATADREPIETWTFGRVTLLGDAAHPTTQYMAQGACMAMEDAVTLGEALRVTDHDILKAFDIYQKARVARTARIVLSSREMGKIYHAKGVQRLVRNDLWRGRPTERFYDAMEWLYGWNVNKCLDAVKNYQGNVA

ST25 4190 cg58_0614

LFSHIDLINTSDQPCYLFIADEAPLQRKLGLYSVRERQ

ST25 4190 cg59_0615

MIDLSLIWVGIIGLGVLIYVVMDGFDLGIGIMFPFIKNSQERDVMMNTVAPVWDGNETWMVLGGAGLYAAFPLVYSTVLSALYLPIIFMVIALIFRGVAFEFRFKAHRTKHLWDLAFIWGSVLTSFLQGIILGAYIQGIKTENGIFAGGPFDWLTAFSIFTGIGVVAMYATLGCGWLILKTEKGLQQRMYELMPKLIIALLIIFGAVSLYTPLTHPEIAERWFSLPNLFYFSPVPILVLLFVGLILSACKKQQDHKPFIYTLALVFLAFTGFVISLWPNIIPPSVTIWQAAAPHSSQMFALVGALILIPIIITYTIVSYWVFRDKVRVGDEGYH

ST25 4190 cg59_0617

MKKSSVKLSQTQWFILLWLGGFLALAVIAGLFKVILIYAAPYLK

ST25 4190 cg59_0618

MYKSEQLAHSIRQLIESGTLNAHEKLPSLRDQVQRSGFSLMTVMNAYQELESQGLIYSKEKSGYFVAEQIALKPLEQYSVVSLNSKIEINSLVFKYLKSIQHESVVPFGSAFPDSQLLAAPKLIQIMGQLARQRQSYDQTASLPPGNLALRKLIAQRYCMQGIQTDPDDIVITSGGLDALNLSLQAVAKPGDYILLQQTVFYGAWQAAERLGLKVITIPEHPQHGFDLEAFEQVIHTYPIKVCWLMLNSHNPIGFTVSDEIKYKIAKLLHEHQIYLIEDDVYEELYYGGQKPLSMKYFDQQNLVLHCSSFSKTLGAGFRVGWVYAGKFSDHIQHLQLMSTISVNALIQNALVEFLSHHHYEKHLRTLRLSLERYKKQFYHYLKQHLPAICEIYYYPSGYFLWVKLPHKLDSMQIYEELIQQDIGVAPSPLFSVLPAQQHYLRINCSFEWNEKIQTALDQVIKTIQQRVEASL

ST25 4190 cg59_0619

MRCGNLSKTAAEMQLSNVSVHKALHSLESALRCPLFKNEGRNLIPLKSAYVFEERAQKIVQDIFITVNKTREAAGFAAKVLHLGSLYSLTVNTIPNVISGLKLRRSELDIQLLLSSNQDLVKKLKATELDAIIVALNETTQDDDFEILPMFSDDIFLAVNKDSKYAEFKDIDLSLLKEETFLTLTKGFATHNDSDIIFKKAGFSPKVALQVNDIFTLISMVSSGVGFALLPGRISAVYESSVKLIPLKQQYHMQQEIGLVFLKSKERDPNLLALIAECRMFASNFKR

ST25 4190 cg59_0620

MVELLLKDLTHLGLITAFALIALIMWLSTKLSKYLTNGRVHASAIAIVIGLILAWFGGKMTGGEKGLTDLALFTGVGLMGGAMLRDFTIVATAFEVQATEAKKAGFIGAFSLFLGTILPFIVGCMFAWIFGYRDAVSITTIGAGAVTYIVGPVTGAAIGASSEVMALSIATGLVKAICVMVSTPLTAKFMGLDNPRSAMIFGGLAGTVSGVSAGLAATDRRLVPYGALTATFHTGLGCLMGPTILFFAVKALVG

ST25 4190 cg59_0621

MIIYGVGLLALCTLVGVIAGDLLGVLLGVKSNVGGVGIAMILLICIRLWMEKRGMMTVETEKGVSFWGTMYIPVVVAMAAQQNVVAALSSGHMALFAAVVSVAVCTLTIAGLSRFNKASPLPKEEDTALNRIGGKVHG

ST25 4190 cg60_0622

MNYSNLLLSSLLTVGLYTSAHAQENLTEESAASSEDVTSTQTQHSRPHLQIIKDLKGIKVKDLKINANAAQPDTVKDPLQSLNRPIYSFNDMLDRHVLRPVAVEYREKTPEDVRGSYRQFRKNLGEPWNAVNQLIQGRPGRAAKTLGRFTINTLTTLGLADPASRLGLPPEEESFGVTLGYYGVPSGPFLMLPFFGPSTLRDGVGLAVDAQARPQKYIMDDQDGLYWSTNLLQAVDTRAQYLDLDQTIQGDQYAMIRDLYLQRKAFQIAEKKGDSADVSFIDDDESEDVPEDNTDKTEK

ST25 4190 cg60_0623

MYFIKPTRSIPFVEQALEALPDLQVIHIDDLDLYDPTIIAIADVQDFLTYKWRLPTIVIAFEHEGSALAQAWEAGALAGWVWNQLPKDLNKALTRIDAQYKRNQDSRDLPSAAELQKRLLPNPIDLLNYEVETFFQPSAYLSGDWYDYWKLNDKEVLFYLADVSGHGVTSSLLTSWMAAFHGRSKTPRQLIKKLNGMLVQENIEKHITIVVGILNLETHSLRWSSAGHYPPPIIFEPNQPPKILTTSSFPLGLTDELEVEEHVCTLNRHARFIICSDGALEPFDGGLNDQFQQLVQHLQNQSFQAPDHVADDIAIFSLCRMN

ST25 4190 cg60_0624

MSTGHVEYASLNGTHIFKLIGEVRAHSCISLDKLLNRIEQQENVVGAIVDLTQTTFIDSTVLGILAKLGLKLKQTHHIQAVMLSTNPDITTLANSMGLGQVFVILNYCGDPNVCTLTLTDEHITHNAMLRTVLDAHKTLMKLNANNQNMFEPLVKQLQKEQDTLEQVSDKQNA

ST25 4190 cg60_0625

MTLVSVVQMNSQDDIESNFQVIESLIQQSKAQNASLIVFPENFVCFAAGKQRETAEQFESIQQRLEKLAHQYQIWIVAGTLPCPFRPDGSIIQDGRVRTVSLCISPERTEARYDKIHLFDVQVGDAVGGYQESRFFEPGTDVVVTSTPFGNIGLMVCYDLRFPELALTLRQQGAHLLTAPAAFTYTTGQMHWQLLLQARAMDSQCYVLGAAQQGWHGEKRQTWGHAGATDSRGQVLSMIGYEGNGLITVPFDLAAQELVRTSMPLMMHRKLIHY

ST25 4190 cg60_0626

MWKYLFSIFCLGANIHCYATDFGTTNNFVSPNLQLKQNVLPPTPKNIPLPAFGQRIIGWGTGAEGARQRLENIQPADVSMIKKQGTTLEMITAWQDFYEQEQQRNENNPTAKYRARLMKKIADLW

ST25 4190 cg60_0627

MDQDCQNLKLENQLCFLIYSTNLALNQLYRKLLTPLGITYPQYLVMLVLWEKDEITVSEIGSKLFLESSTLTPILKKLEALQLLNRTRSKEDERQVIITLSEKGKKLKEQAVNIPTHILEASSCDTATLLGLKDQLTQLRTNIAK

ST25 4190 cg60_0628

MSLEKVVYRAKAKATGGRDGRATSSDGVLDVQLGVPKEMGGAGGAVTNPEQLFAAGYSACFLGALKFVANRDKFNISKDAYVEGEVGIGPIPTGFSIEVTLNVYLIGMDREEAEKLVAAAHIVCPYSNATRNNIDVTFNIVTE

ST25 4190 cg61_0629

MCLLAESFTTKGQYQKLGSIIFQPVDESMNNEDNFVTAWKKLAIKTDKCVLNTQK

ST25 4190 cg61_0630

MTAALSAALGALVCWLATSYFAWQSFRTAGARASKQVLSNMYRGMLGKFAIMIVGFILILSNVKPLSPLALLFGFILVQAMSWVAPFWVSRLQKRV

ST25 4190 cg61_0631

MAAEEHALTSTEYIKHHLTNMTYGKMPDGTWKLAETAEEAHSMGFTAIHLDSMGWSIGLGVIFCLLFWIVARAANAGVPTKFQSAIEMIIEFVDSSVRDTFHGKSRLIAPLALTIFVWIFLMNLMDLIPVDWIPQVAAFVGANVFGMDPHHVYFKIVPSTDPNITLGMSLSVFVLILFYSIREKGVGGFVGELALNPFNPSNPVAKALLIPVNLILELVTFLARPISLALRLFGNMYAGELIFILIALLPFWIQWALSVPWAIFHILVITLQAFIFMMLTIVYLSMASEKH

ST25 4190 cg61_0632

MELTLGLVAIASAILIAFGALGTAIGFGLLGGRFLEAVARQPELAPQLQTRMFLIAGLLDAVPMIGVGIGLFFIFANPFVG

ST25 4190 cg61_0633

MNINLTLIGQAIAFAFFVAFCMKFVWPPLINAISERQRKIADGLNAAEKAKADLADAQAQVKQELDAAKAQAAQLIEQANRRAAQLIEEARTQAAAEGERIRQQAKEAVDQEINSAREELRQQVAALAVTGAEKILNQQVDAEAHNAMLSQLAAKL

ST25 4190 cg61_0634

MAELLTLARPYAKAAFAYASEQGATDNWSNALQVLSAAVQDEAFSAYLNRPELTPAEQVKLFAKVLGEDQSQAVSNFLTLLADNDRLVLLPEIAAEYEQLKSQNNNNVDVVIESAFPLTAEQEQLLKSALEKRFNSTVTVSVEVKPELIAGVVIRAGDQVIDDSALNKLEKMRTRLLA

ST25 4190 cg61_0635

MQQLNPSEISALIKQRIGDLDTSATAKNEGTIVMVSDGIVRIHGLADAMYGEMIEFDGGLFGMALNLEQDSVGAVVLGNYLSLQEGQKARCTGRVLEVPVGPELLGRVVDALGNPIDGKGPIDAKLTDAVEKVAPGVIWRQSVDQPVQTGYKSVDTMIPVGRGQRELIIGDRQTGKTAMAIDAIIAQKNSGIKCVYVAIGQKQSTIANVVRKLEETGAMAYTTVVAAAAADPAAMQYLAPYSGCTMGEYFRDRGEDALIIYDDLSKQAVAYRQISLLLRRPPGREAYPGDVFYLHSRLLERASRVSAEYVEKFTNGAVTGKTGSLTALPIIETQAGDVSAFVPTNVISITDGQIFLETSLFNAGIRPAVNAGISVSRVGGSAQTKIIKKLSGGIRTALAQYRELAAFAQFASDLDEATRKQLEHGQRVTELMKQKQYAPYSIADQAVSVYASNEGYMADVEVKKIVDFDAALIAYFRSEYAPLMKQIDETGDYNKDIEAAIKAGIESFKATQTY

ST25 4190 cg61_0636

MANLKEIRAKVASIKSTQKITRAMQMVAASKMRRAQERMAQGRPYADNMRRVIAHLVQANPEYKHRYMVDRPVKRVGYIIVSSDRGLAGGLNINLFKKVVQHVKAQQEQSIEVQFALIGQKAVSFFKNYGGKVLGATTQIGDAPSLEQLTGSVQVMLDAFDKGELDRIYLVSNGFVNAMTQKPKVEQLVPLAPAEEGDDLNRTYGWDYIYEPEAEELLNGLLVRYIESMVYQGVIENVACEQSARMVAMKAATDNAGQLIKDLQLIYNKLRQAAITQEISEIVGGAAAV

ST25 4190 cg61_0637

MSSGRIIQIIGAVIDVEFERTSVPKIYDALQVDGTETTLEVQQQLGDGVVRTIAMGSTEGLKRGLTVTSTNAPISVPVGTATLGRIMDVLGRPIDEAGPVATEERLPIHRQAPSYAEQAASTDLLETGIKVIDLLCPFAKGGKVGLFGGAGVGKTVNMMELINNIAKAHSGLSVFAGVGERTREGNDFYHEMKDSNVLDKVAMVYGQMNEPPGNRLRVALTGLTMAEYFRDEKDENGKGRDVLLFVDNIYRYTLAGTEVSALLGRMPSAVGYQPTLAEEMGVLQERITSTKSGSITSIQAVYVPADDLTDPSPATTFAHLDATVVLSRDIASSGIYPAIDPLDSTSRQLDPLVVGQEHYEIARAVQNVLQRYKELKDIIAILGMDELAEEDKLVVYRARKIQRFFSQPFHVAEVFTGAPGKLVPLKETIRGFKGLLAGEYDHIPEQAFYMVGGIDEVIAKAEKL

ST25 4190 cg61_0638

MATMQCDVVSVKESIYSGAVTMLIAKGAGGELGILPGHAPLVTLLQPGPIRVLLENGTEEIVYVSGGVLEVQPHVVTVLADTAIRADNLDEAAILEARKNAEQLLANQKSDLDSAAALAALAETAAQLETIRKIKNRAQ

ST25 4190 cg61_0639

MKKVLQGAFFPFVIFASGMLLLGCDQAQDHAKEQESVQTNDQISEQADDSQQEITATQNKTELKNGNVFYIVRDAANLQLKAGDYIEKLKDTQLDVEQAIQDKDQHELKTTVTTLKAQLEGLNQALLGLDIRSQEVENIRQSLLQANQQALSMPLLNGKLEQINFDQIEKQLNTIQMDMVKLAAMIMAGDEKSDSKTDS

ST25 4190 cg61_0640

MTQSVYHIPVKAISGETVDLDQYKGKVLLIVNTASKCGLTPQYEGLEKLYQAKKDQGLEILGFPANNFKEQEPGSDEEIQQFCSLNYDVHFPLFSKISVAGEDKHPLYQVLTTAQPERIGEGPFRERLEGLGIPTNPAPEVLWNFEKFLVNKNGEVVARFAPNLTADDEQIVKAVEAELAK

ST25 4190 cg61_0641

MKNEDQQFSLLESIEVHEFLYEKEDIVRPHASLWGDFNFSLNGILEIQVEEQIYLSPPSYGLWIPPQTVHQSTHIDHEIHYICIRLHPRLCSILGDICRCFSIQPFFRTLVLQILEQQKQTEKPEYLEHLLQVLFDQLQQAPAYSHYLPQTRHPVLLPILEKLSDPLLFNLSLQQLLQNFSVSDRHLLRLSQQELQLSLSEWRNRAKIVYAIHQIRQGTPIKRLAYDLGYQHSSSFIEFFKRYTGQTPVQIRNN

ST25 4190 cg61_0642

MDQSTTSKARPMTSTQGSWFAILAVAIAAFALVTSEFLPVGVLNSVAADLHISVGTAGLIITVPGIMAAIAAPLLPVSVKQLDRRYVLILLTAIMVIANTITAFAENFHVLLLSRLILGISIGGFWATAIALSGKLAPANLPIAKATAVVMAGVTFATVLGVPIGTWLSEFYGWRSAFGITAAIGLVVLVLQLIFLPKLLPESAIHIRDLPALLRTPKARSGMLIVLLIGLAHFCAYSYLAPFFKNVAGFNGTTISSLLLLYGIAGIFGNAFAGYSGNLNVRYTLAFVGTCFAIVFFGFPIFAIHEFGAIVLTALWGFAFGAFPTSANIWMFVHAPHAVEKGMPLFVGMFQVMIATGSLLGGYVVDHFNENTLIYGVLSFVALALISTFTLAKGLNNPKATCEN

ST25 4190 cg61_0643

MLLFWEHGYEATSISDLTHALEITAPSLYSAFGDKAGLFYKSIDYYLAHEACPIETIFLEAKTAKIAFELYLYDNVKRLVQPNKPAGCMLVVAAMNCSDATQEVQQNLLDKRIKTKEKLLKRLEQGVEQGDLPISAPLQEMTDFYATVIQGAYHSGQRWCKYRTIT

ST25 4190 cg61_0644

MSQVMKYHKWAFIFPILAVLIWSLNIVVTRYVSDYISPVSISFYRWLIAFIILTPFMFLSVWRQRRLVIPYLPKLAVLSAFGMVLYQGLAYTAAHYTSATNMGIVNAFIPIFTIFVSLAILKDVPNRFAVFGSILSFAGLLYVMSQGNINKLLSGGGHLGDALMIIAVFFYAFYGVFLKKWQLPLPIMTSLYVQIGFSVLFHLPLIFWFGLDALNAQNAPSAIYAGVFASLIAPLVWMLAVQQLGPNRTSIFMNLVPVFTAIIASIWLSEQWTIYHTFGGIMILVGIIMAQKKVNSKKSDLLPEQN

ST25 4190 cg61_0645

MITTSVTEAAECLQQGQVLAYPTEAVWGLGCDPFNEQAFQKILELKQRPIEKGVILLAGHISQVEHLLTSLPQTTQQEIIDCWTNHQPSERATTWLLPADQHIPSWIKGEHPLVAVRVTTHPLCVALCNAFHGFIVSTSANPSGQEPAHSLQDACQYFGSQLNYLNGDLGQSQQPSRIINALTGEVIRP

ST25 4190 cg61_0646

MLDVPFTMLTQLSSHHYHTLKVWYLVQHSLVSFKKIIDYFGNCEKATQPDCLAKWSSLGLHANHLKRVNEFQTPQGQAQFEQLVQQVRQHTDFILTPDDSGYPTQLLPYTDHPPIIFGKGQAQALLQPQIAIVGSRKPSPHGRQVAYDFAYYLSEKGFFISSGLAYGIDEAAHQGASTHQRTIAVTGTGLDTTYPAQNKKLAEHILAKNGAIITEFLPGTPPLQQHFPRRNRIVSGLSLGVLVVEATLKSGSLITANKAAEQGKTVFAIPGHIYSEFHQGCHQLIREGAILIDHPEQIIEDLALPTQWQSQQQNQTEEANTNTPEIPEHLIDLYQSLDWVGQNIDQLVVHHNIPVSELTSSLMELELLGLCMQQSGLYLRCRS

ST25 4190 cg61_0647

MKKVFNGMVNFHALGIKKHLLALALCTGVAIGTIAEVHATSPNHNPPSLKSNAPNVYVVKRGDTLWDISGHFLNKPWRWPEIWASNQHVKNPHWIYPGDRLLLCSLDGRPLVGKDEGDGCVGIIRRYTGQTTNLQPQVRVEALNNSVPVIPLEHIKQWLENSTILPADSITNTPYIVGTADQRVLAGKGQTIYARGQGLINGQRYAVYREGEPYYFTDNKGKKHSLGIELLQVASGVAVSSEKDITTLELTDSYNAEVRRGDRVMPEEQATLPTLFYPVDAKQVTDGGKIIRVMGSIGRAAKNSVVTLDRGTTQGIQVGQVFDITQQGESIRDPKTKEVIQLPGQQIGSLMVFRTFDQLSYAFVLESDLPIKVGSSIQPPQFND

ST25 4190 cg61_0648

MALLPILSFPDPRLRTIAKPVEEVTDEIRQLAADMFETMYAAPGIGLAASQVDRHIQLIVMDLSESKDEPMVFINPKVTPLTEETQPYEEGCLSVPQIYDKVDRPSRVKIEAINLEGQAFEIEADGLLAVCIQHEMDHLNGKLFVDYLSPLKRQRAREKVEKIVRQREREKVAVKR

ST25 4190 cg61_0649

LVFRSGLLLACAAVFLQIAVFLQPLLPKQYQIAPVCETITRALLKPKAQTEAMSHAMHHSHHQHHIEQAQVPDHHDHHDANHQCQYCTVYANLVLPPEFGVKEVLVRIQVRLVAYQQAFRHVYFALQRLFLLPQGRAPPLFA

ST25 4190 cg61_0650

MPHSKFLLQPLWVAMLAVSHSGLVFAESEKNDAETNTLHSLAPIVVTAQQGNDANGLIVHADPKQPIQPVPATDGADYLQSIMGFNSIQSGGTNGDVTFRGMFGSRIKILTDGTENLGACPNRMDAPTSYISPESYDRISVIKGPQTVQYANTGSAATVLFERQPEKLTSEKPYRGQASVLLGSYGRIDHNIEAAVGDEKKYIRLNANRSESNSYQDGDGNTVPSAWKKWNVDVALGFTPDENTWVEITGGKSDGESLYAGRSMDGSQFARESLGLRFEKKNITDVIKKIEGQVNYSYNDHVMDNFSLREFNPQDGMSMPMASNVARRTLNARLAMTNEWSQWSFISGVDTQNNKHSSRSSMRSNYLNQPRVTDMIFHSYGAFGELGYQWNDFNKLVTGVRLDRVTVEDERAKSKDFNTKLEKTLPSAFVRWENQHPEHELKSYIGLGYVERMPDYWELFSPIHGNAGSTNTFNGVNPEKTLQLDMGFQQQHGALSTWASAYAGLVDDYILMSYHHHPSMGMDGHGMSHGITAGAKNVDATIAGAEAGIGYQFTDHIQADLSAMYAWGKNTTDDKPLPQISPLEGRLNIRYVADKYNFGLLWRAVAEQNRVSLHQGNIVGYDLKPSKGFSTLSLNGSYNLRKDIDVSVGIDNVLDKTYTEHLNKAGSAGFGFASEEQFNNIGRNYWVRMSMKF

ST25 4190 cg61_0651

MTNKLSPEQSWWGWKLLIVASILSVLFMIIFYLAINNEPDYMPSQKKKQMMESHEQMNHAEMHSSSAQ

ST25 4190 cg61_0652

MQMISPTRLASFNDMQDSNFFTQFLNICCEKPVQPSYTEYVSLQHALYEGDIEMDKVIDWIMQNPKDHRIIFEKILFQGRDELSEPIPTELENFFNYIEQKPEWLDQRQIDEAVKFTHRLGINNGFILRDLSLMAGYLYPGFNQPLILTGALKKEAGTRLAETTKWWVDITEPQGLSRLSAGFTSTIYVRFIHALVRRQLKKSDRWDSEVWGIPLNQFDLAMTNLAFSSVVLLGIRALGIWPTKQEAKSFLHFWRYVGWLMGIDEKWLIQSEPEGWRLLYWMQFAHPRSDHSSIELGLSLSKEPFERKYLHLRSLQQKLAYRQHLELTQFFIGKKRMKLLGLPQQSASWFAYYLIVRNLLLYNGAKLSPKVEKFLSKSGRNIQKLGLTLYQNQGKAKTLASMHQ

ST25 4190 cg61_0653

MRKAPQQQRSKIIVDHILEATQQCIAQYGLLHTTTPKIAEKSGVSVGSIYQYFENKDQIIEELLRRKSELLGQQLKELVIQEGNIPLELLIPLAIELGFNALKADHGFFIEVLKHWHGYSHSQAAQILEKHFFEVGLYTFSRNPHQWDFEQVKHKSFVIINSTLFTMMRYVSQNNFLISEQQLKRELSSMILAYLSQK

ST25 4190 cg62_0654

MQDSIEQYMQKVGQQARDASRVLTSASTSLKNHALSAIYTALENNQAAILAANQIDMEKGRSNQLDSALLDRLELTPARFKGMLQGLKDVIALVDPIGEITDLAYRPTGIQIGKMRVPLGVVGMIYESRPNVTLEAASLAIKSGNAIILRGGSEALESNKAIAEAVKHGLKVAGLPEHSVQVIETSDRAAVGHLITMAEYVDVIVPRGGKSLIERVTNEARIPVIKHLDGNCHVFVEAQADLQKALPITLNAKTHRYGVCNAMETLLVDEKIAEVFLPHIAELYAEKQVELRGCPETRRILGTTVKPATEEDWYTEYLGPILAVKVVSGIDEAIDHINKYGSHHTDAIVTENYTLARQFLARVDSSSVVVNASTRFADGFEYGLGAEIGISTDKIHARGPVGLEGLTSQKWIVLGDGQIRQ

ST25 4190 cg62_0655

MDSEINQAYAGFGFFDIYHRDSFKQPARTTWIDGWKIEYMAIADPQTIHKTPIVIVGGAFQNFNSYKYCVEQLFESGPVILIDLPSMGANQQITNRDTGISAGTLELPDLSEMLGRWLDIVGIQKVSVMGMSLGSVVASCFAYHRPDLMDRMILMGVMQKTRKSWRMILEESLKLMQENRMEEFGQAVILYLVNHAKLDKTRMSPTAKKLFFRQMAEFTGTERERYEINCNRLLRLTDVPIPECKTLVAAGQYDSFTLPHENANFALQCPDMEFALIANADHVPQLQRRKETMSLFTSFLKGESIQNLDGIIPMTREQMQNMERRGEERITVLQPKTKLSHRECETEVPVTIVDVTFFGMYLKLDDVAQLEFVNEYPRDLALHLEDEEGAFSIECLIFEATEQGVRALFKHGSFELADRLSRFIARQKQAA

ST25 4190 cg62_0656

MTQHLSKHRHISFTAHYTGYIWYQMGISHEALATSKGKSLAYLVHPLESWAEKYVGGSMRTTLKQRHTMLDHDLEKLIQENPDLQVLEIACGLSPRGWWFRQHYPSISYRELDLPDMAQTKQNALQQIEKNAPEVLSVDLFTEAFAQAFEVFDSNRPLVVISEGLINYFDKDLLKQLIQSIAHYGASFKKLHYLTDLYPEPVKNKLASIIWNSSKLLKLMSRSSFSFHFKTPLEVKDFFKDAGFSQVNVEQPQIFFGQVSKDSDEEHLGDLVWTIHAQLK

ST25 4190 cg62_0657

MIGALMLDIAGTELTQEDIELLQAPQVGGMILFARNIESPQQVRALTDHMRQVRPDILIAVDQEGGRVQRLKSGFTLLPAMGRFGELYITQPQKALELAEQCGWLMATEVLAVGIDFSFAPVLDLNAISDVIGDRGFSKNIEDIAPLAGAFMRGMKKAGMANTGKHFPGHGSVKADSHVAAAIDSRSYDEIYNHDMQSFIKLMPELDALMPAHVIYDQVDPNPAGFSPFWIQEVLRNRLKFNGVLFSDDLSMQAACVAGGADARIQAALAAGCDMGLVCNDRSAACTALDGIANLELPNQERLERMRGRIPQIQVGETLSLGNDWQAVKTAIEEFKNSI

ST25 4190 cg62_0658

MKLQTIACAVAIATGGLFFSHTMNEARAATNTAAVSQSIQPTQEQALVARQLATLVDRQHYLNMRLDANTSNRILDMYLDSLDPDHSLFLDAEVQNYKKLYGSNFGASLKAGNLTGPFAIHQQYRERLKQFYEFMLAELKKPQNLKQPNTFIEVDREKAPYFKTSAEQQNHWRKMLVSQLINLTISREEEQAKQKALKENPSLADGQDLTGPEDLTPAQTLTKRYTRQLERISRVKSDDVLDKTLNAMLATYDPHSNYYPPIDAIELNRQTTLQLEGIGVSIRPERGNEDYTKIETIVEGGPASKSGQVKSGDRIVGVAQEGGKMIDVVGWSSSEIVGLIRGKRGTKVTLKLLGAGASMSQARNVTLVRDVIQEEDAGVRSRTVEVTRDGKKHLLGVIEIPSFYFDYRSRRAGQQYRSVSEDTANAFEALKAKKVEGIIIDLRNDPGGSLEEVARMLGQVIKSGPVVQIRDGNGNVSVFEDNDGGQQIYTGPLAVLVNLASASASEIYSAAIQDYERGIIIGSTTTGKGTAQVQLDTLAYGQATLTQRKFYRVTGGSTQNKGVVPDIKLVDIYNEEFGERKSKNALKWDTIPTAPFKREGSVQPYVAKLSQLSEQRVAVDPQFKYLNKRTAIAKVTSDQKQVVLDIDKRRAELLSLEKQTLDAENERRIATGQKPFPNWESYQASLDALAESRAKMKANQRPALPEEETFVNEAANVLMDYAKLQNR

ST25 4190 cg62_0659

MDGAMQGSYATALLKQQQLPESFQFYFKQKQHLSNTQELHQLHELVRPRLKIAIVTETWPPEINGVALSLLQLCQGLQKQGHKILLVRPEQKAKCHDFLPEQECLVMSQAIPKYPTLQFGWPQYLKVSKAFEKFVPDVVHIVTEGPLGLTAMQAAKAKDIPVSSGFHSPFQDFSRFFDLAFLVKPIQKYLCWFHNNTQVTCVPSRDTEEALRGFGITCPLVVVGRGVDTTRFSPKHRSENLRQQWGVDSDTRVMLYVGRLSPEKEVQLIVESYAAMQNIQQQKTKLVVVGDGPDLARLKALPEAKNIIFTGSLRGHDLAVAYASADVFVFASQVETFGNVVLEAMASGLPVIAYDYACAHQYLIHGVNGWLSPLGHKNHFIQQIYQLPSIQQLREMGIQACHKVQQSGWQLPVQQLEQAFYQVVKEPSADFT

ST25 4190 cg62_0660

MKFKNAKIKILDLDLRGCLYLNNFSHSQRVALFFKIISRAGDGPFWYLMLAIVWGMQGITYSLQIIYLLLGGSVGTAIYKFLKHKTTRPRPYQVHQVIVLGERPLDHFSFPSGHTLHAVMVTIVLGYIQPVLLAAMLPFMVLVALSRMVLGLHYPSDVIVGALIGAAVASLIIFVAPLLGIAL

ST25 4190 cg62_0661

MGLRWTDTIDIAIELSEAHPEVDPQWIRFTDLHAWVCALPDFSDDPNKSTEGLLEAIQMAWLDEVR

ST25 4190 cg62_0662

MAIERTLSIVKPDAVSKNHIGEIFARFEKAGLKIVATKMKHLSQADAEGFYAEHKERGFFGDLVAFMTSGPVVVSVLEGENAVLAHREILGATNPKEAAPGTIRADFAVSIDENAAHGSDSVASAEREIAYFFADNEICPRTR

ST25 4190 cg62_0663

MSSAVVVSSENLDGQQQSSSTPASPAAEKVNLLGMSRAELEKFFEDIGEKKFRAGQVMKWIHQYFVTDFAEMTNISGKLRAKLEQICEIKAPEVVHRHYSKDGTRKWVFRVGEGSGSLVETVLIPAEDKTGSRKTLCISSQVGCALDCSFCSTGKQGFQRDLTPDEIIGQLWMANYSYMEEVPVAERERSVTNVVMMGMGEPLLNYDAVLSSMHIMLDDFAYGMSKRRVTLSTSGVVPKIDQLAKDIDVALAISLHAPNDELRNELVPINKNIHWLS

ST25 4190 cg62_0664

LAQLIAACQRYIAKDGNESARKHVTIEYVMLEGVNDQPEHAQQLLKLLKNLPSKINLIPFNPFPHAPYGRSSRNRIISFQKTLSDAGFVCTIRQTRGDDIDAACGQLVGQVADRTRRAEQWQKKVAQRQEILRTQG

ST25 4190 cg62_0665

MLCMGVAVALLASGCQTSQTVKKDPEKAVKVRTQLAAEYIRSGDLDSAKRSLDQALSVDSRDATANMMMGILLQQEGSKSNLEKAEHYFKRAISSEPDNAQARNNYGTYLYQMERYNDAIEQFRIAGATLGYDQRYQALENLGRIYLKLGDIASAEKTFKQALLANRDSYISMLELAEIFYLQQQIPAATQMYEQYVRTVGQKNQGARALWIGLRVARANADKMGMQVLVNQLRALFPESPEYQRYLQLQYSTEAVWK

ST25 4190 cg62_0666

MEINPNSQQPTGSSLPTSALGNIQRPGEYLRQIRVSQKKELEQVSSDLNMPLKTLNALEQDDYKSLPEATFIKGYYRSYAKYLNTDATAIIQRFDEIYANDTGLLPNHALNNSPIKIMGKLPGSNSDRNKKWLKRALLAILIIAVVSLIVMGVQKWTSNKEDAELPKANQSNVEVLPMKGNASATVGDQLVLNFNRPTSVHIVDATGKVLATGRQASTLTLNGESPFQIRLDDATAVSLSLNQEQISLSPYTVNGKAEFRLSR

ST25 4190 cg62_0667

MIENPIKRRPTRKIRVGSVYVGGDAPISVQSMTNTETCDVDATVAQIERCVDAGADIMRVSVPSMEAAEAFGAIRKRVSVPLVADIHFDHRIALAVADYGADCLRINPGNIGSDQKVREVVAAARHHGISMRIGVNAGSLEKDLQKKYGEPTGQALLESALRHIDILDRLDFHEFKVSVKASNVFLTMDAYRLLSQQIDNPLHLGVTEAGIYRTGTVKSAIALGGLLMEGIGDTMRISLAAEPEDEIKIGFDILKSLGLRSNGINFIACPSCSRQEFNVIQVMQALEERLEDIRTPMDVSVIGCKVNGPGEAKEADIGVVGAAPRSLVYRNGEKSHLIDTNQLVDEIETMVRQRVQELEEAKSKEIIRSSS

ST25 4190 cg62_0668

MSSIVAIKGFNDVLPTQTAAWRRLEQHLASLMDAYGYQQIRLPIVEQTGLFKRAIGDATDIVEKEMYTFFDKGNPPESLTLRPEGTAGCVRALVEHNLLRGATPRVWYMGPMFRYEKPQKGRYRQFHQFGVETFGVATPDIDAELIMLTARLWKRMGVDHMVQLELNTLGETDERTEYRNALVAFLNEHKDALDEDSQRRLTTNPLRILDSKIESTQKILENAPKLHDFLKEDSLSHFQQLQDYLTAAGIKFVINQKLVRGLDYYNKTVFEWTTTALGSQGTVCAGGRYDGLVGQLKGKADQSVPAVGFAMGMERLLLLLEQVEQAEIVRDCEAFLVAEPAYQSKALVLAEQLRDQLEAANSNIRIKTGSQGSMKSQMKKADQAGAVYAIILGEREWEAQQLAVKELATAEQSQVALAELVPFLIEKFTK

ST25 4190 cg62_0669

MSLSDEEQFDSLKSFAKKYGSAMISGILIALIAFFGWEYWQKRNLATSQTETAKVQQLMDEANATADNPNALASITASADKIVKDDIDSVQAIQTQFVLAKLAYEKQDYAAAEKALKKVENSKVKDEGLIQVVKLRLADAQLAQNKYDEALKTLSGDVDPAFKATVEELRGDIFVAKKDIDSAKKAYQAAWDSLLERKQERQILQIKLESVGVLVEDPQIERPILETQVEES

ST25 4190 cg62_0670

MNQKFKLPLAIAIASAVLVGCSSNKVKEAKPNPLPKLTESNKSLVPVFSRSVSSTNKADPLRLQLDASEGVVFTLDPKGEVAAYRGKQRLWEKKVSKLGLSSGVEAAEGIVVVGNSKGQLFALDQATGEQKWTAQLSGALLSPSLVQSGRVITIANDGTVFAHDVSSGQQVWAYKLPNVQFSLRGQPSPVSLDPRTVLIASANAYVYAIDTISGIPRFQRRVAVSEGRSDIQRLIDIDGDPVVAGQLMVTTSFQGQVTVTDLASQRVVWSEDSSSTKRPEVYDNKVFVSSTDGKLTAYDLTTGEQLWQNDSLLNRHLSNPVVLGSDLIVGDLDGVLHLIDPTTGKLIGRSKTSGEVNTLRVIENQLYVSTRKGDLSIWQNR

ST25 4190 cg62_0671

MKPVIALIGRPNVGKSTLFNQITKSRDALVADFAGLTRDRKYGDATYQNKSFIVVDTGGIGESEGGIDNYMAEQSKTAINEADIIIFVVDARAGLLASDEQIARELRTLGKKIYLVANKVDGVHAEAALVEFYKLGMGEPLQVAASHGRGVQQMLEDVLQDIPEDENPEEHDKDTGLRLAIIGRPNVGKSTLVNRLLGEDRVVAFDQPGTTRDSIYIPFEREGRKYTLIDTAGVRRKGKVDEMIEKFSIVKTLQAMKDAHVVVVVVDAREGIVEQDLHLIGYALEAGRAMVIAINKWDNMSEYDRKQCKLDVERRFDFIPWARIHLISALHGTGVGELYPSIHRAYESANLKVSPAKLTQILNDATDQHQPPTVQGRRIKMRYAHMGGQNPPTIVIHGNKVDKTPADYRRYLENVFRKVYKLEGTPVKIEFKTSENPFEGRKSQVDERTAARRRRYIQKFKKAEKKFKR

ST25 4190 cg62_0672

MELEFAKHKHGKPYLLDHALHFNHSHSQQYYALALSERIKDIGIDVEELDRKVRLDSLAQHAFHPDEYATWQSLEQDREYWFKVWTTKEAVLKASGLGIRLDLNTLNTQAHPTNHGGLCSHELIGTFAYQNFVLGNMILTVAWRSEQSCRGFQFPSIQIHSLDR

ST25 4190 cg62_0673

MSEEQQVQPQLALERIYTKDISFEVPGAQVFTKQWQPELNINLSSAAEKIDPTHFEVSLKVVVQANNDNETAFIVDVTQSGIFLIDNIEEDRLPYILGAYCPNILFPFLREAVNDLVTKGSFPQLLLTPINFDAEFEANMQRAQAAAVEGQA

ST25 4190 cg62_0674

MAANVIVYSTSVCPYCVRAKQLLERKGVAYKEVNLSVEAPEVRAELMQRTNHRTVPQIFINDQFIGGFDQLYALEREGKLDELLA

ST25 4190 cg62_0675

VERWLEFMGNHPILFGTLGVLIVLFFIFEGQRNGRKISPQSLGILVKAKNALLIDLRDSKDFREGHISGSRNIPYSQIASHADELKASDRPLVFICNLGQVAGSALQKVAHHDSYRLDGGISNWKAQGLPLVKSKPKA

ST25 4190 cg62_0676

MALIRKRRLTEQQQRRIEKQHKTRQEEVDTSQDLDGLVVQHYGRQLEVQALSVPDHHPEKPQVAEGEPEPFWKPIELNSVWRCHTRTNLELLVTGDRVKWQADPNTGLGIITAIHPRTSLLTRPDRYHKVKPVAANISLIVIVFAPLPEPAPTLIDRYLVACADANIPALLVLNKSDLLTENDPILDMLKEYENLGYEVMICHSKGDISALSQRLDGETVAFVGQSGVGKSSLINVLIPDAEQKTNIISENSALGQHTTTSTRLINFGKNGALIDSPGIREFGLWHLDLDKIRMGFPEIEAHLGSCQFRNCTHTHEKNCGLKQAVEAGEILPRRLDSFLRLIDEIQEAQQKN

ST25 4190 cg62_0677

MSSTLNTRLIWIDLEMTGLDTDNDQIIEIATIITDDHLNVLAEGPVLAIHQPDRILNAMDEWNTRQHGQSGLIERVRRSKLTARDAELQTLEFLKKWVNPKVSPMCGNSICQDRRFLHRLMPELEQYFHYRNLDVSTVKELSKRWRPEIMSGLKKNASHLAMDDIRDSISELKYYREYFFIMNTDGKD

ST25 4190 cg62_0678

MQSNNPILTRVETVSDYSQPMTVQGAIQKSVMLTIIAAAVGVALFFYAAFTANVGIAYAASIVGAIGGLVLALITTFKPTTAPTLAIPYALFEGAFLGGISFTFQLKYPGVPLQALLATFVTTLVMFGLYKFQIIRATEKFKSVVISASLAIFIVFIVQMVMRLAFGSSIPYIFESNWLGIGFAAFVAVIASLNLILDFDLIETNAAYRAPKFMEWLCGIALLATLVWMYISFLRLLGLLSDD

ST25 4190 cg62_0679

MQFRKTLNIGIMLKIVLSEIGMTQGLLAGKRFLIAGVASKLSIAYGIAQALHREGAELAFTYPNEKLKKRVDEFAEQFGSKLVFPCDVAVDAEIDNAFAELAKHWDGVDGVVHSIGFAPAHTLDGDFTEVTDRDGFKIAHDISAYSFVAMARAAKPLLQARQGCLLTLTYQGSERVMPNYNVMGMAKASLEAGVRYLASSLGVDGIRVNAISAGPIRTLAASGIKSFRKMLDANEKVAPLKRNVTIEEVGNAALFLCSPWASGITGEILYVDAGFNTVGMSQSMMDDE

ST25 4190 cg62_0680

VGCAAVVKTPYQAPAVQVPGSFQYDKAKAKTASVEQYSDHWWTLFGDAQLNQLVTNVLERNSDLAVAGITLKQARLQADLTANKQGLRTSSSVSTGHSFDLNSGDDSAKGLSMSAGVSYELDLFGKLARQTEASKWEALATEQDLQATGQSLIATTAKLYWQLGYLNERYATAQQSLATSQKLYQLVQTQYKAGAVSGLDLTQAEQSVQSQKASLSQIEQQLVETRTAIAVLLHEPVQQLNIQEPQRLPRTALPAIGAGLPADILSRRPDLQAAELRLRKALATKDATKASYYPSISLTSSLGSSSTSLTELLRNPALTLGASLSLPFLQYNDMKKDIAISNLDYEKAIIQYRQTLYQAFADVENALSSRTELDKQVALQERNVELAEKTERLTEVRYRYGAVALKTLLDAQQTTRTARLSLVETKQSQYNAYVTLMQALGGSPVKELPQ

ST25 4190 cg62_0681

MALLSMNAHRMRTFLTMLGIIIGIASVVTVVALGNGSQQQILSNISSLGTNTITVFQGRGFGDNSKTANFKTLVPADADALMTQPYVSAVSPMVSTSKTMRYQQNEANATINGVSNDYFDVKGLVFKDGQTFDQRSVRDRSQDVVIDTNTQKQFFSDGTNPIGQVVLLGSVPARIIGIVEPQTSGMGSDDTLNVYMPYTTVMSRMLGQAHVRNIVVRINDKYSTSAAENAIVNLLTQRHGAQDIFTMNSDSIRQTIEKTTSTMTLLVSAIAVISLVVGGIGVMNIMLVSVTERTQEIGVRMAVGARQSDILQQFLIEAILVCLIGGVLGVLLSLGLGQLINKFAGGNFAVAYSTTSIVAAFVCSTLIGVVFGFLPAKNAAKLDPVAALSRE

ST25 4190 cg62_0682

MTKQALLEVSNLVREFPAGESTIQILKGIDLTIYEGELVAIVGQSGSGKSTLMNILGCLDRPTSGSYKVNGQETGKLEPDQLAQLRREYFGFIFQRYHLLGDLSAEGNVEVPAVYAGVTPADRKQRATALLTELGLGTKTQNRPSQLSGGQQQRVSIARALMNGGDVILADEPTGALDSHSGVEVMRILRELNAAGHTIILVTHDMQVAKNATRIIEISDGEIISDRPNVPDQSLEEVKSDPDAAPALQNKQKKAKVFQLGVQP

ST25 4190 cg62_0683

MPKIKPIKLVIIVVCIAIIAVLAWKFLKPKQQQPQYITAEVTRGDIENNVLATGTLDATKLISVGAQVSGQVKKMYVQLGDQVKQGQLIAQIDSTTQENSLKTSDANIKNLEAQRLQQIASLNEKQLEYRRQQQMYAQDATPRADLESAEAAYKTAQAQVKALDAQIESAKITRSTAQTNIGYTRIVAPTDGTVVAIVTEEGQTVNANQSAPTIVKIAKLQNMTIKAQVSEADIMKVEKGQQVYFTTLGDETKRYATLRQIEPAPDSISSESNSTTSSTTSSAVYYNALFDVPNTDGKLRIDMTAQVYIVLNSAKNALLVPSSALSSKQFSGQRKQQGQSADKASSTPSAERKHQGNGARLERLNLTPEQKQLIEQGKATLSVVRVLQADGTTKPTQILVGINNRVNAQVLAGLKQGDQVVIADSSENSAASANSGNNRRRGPMGM

ST25 4190 cg62_0684

VWKNIIYTLGQPNHLPALLKYHKNSRFILI

ST25 4190 cg62_0685

MKLDYLQALKRIPEARGAWILHGQEPLLEQNLLDTFRKSWQQQDIERQRYDISSVSDWKNVFNALNSLSLFSQQLAIEVHGNIKPDANGLKQLKSYIQHNETNLLLIVLPKQDSSSLKSAFFQVVEANGVVVALTANYPQDRQRILAAEAEKLEIQLDNDAWQWLMQHHEHNLLAAKNSLMRVRDTFPDQKLIQIEQLYACLQDQSRYTTYDLSDALLEGNLAQSIKIFQYLIGSGEPESLILWTLSKEMRLLMQLFEQPHNALQLGIWKTKVSLYQQALRRLNPQQFLGWSALLLQIDAAIKGMSNENAQHLMQQAIAELCGKTLFIH

ST25 4190 cg62_0686

MHLAQRLAAVVLTLGLSAGLVGCGFHLKGTNPTATPLVYKKLSLELPAKTDDLETQLKVYLTANGVQLSNDNDAYVLRVLEYTPRRQLLNGKLTEVLLRLTVTFQIEDRQGNKITEPRTLTAARSYQYDLATVNTENQQESYLQRIVIDDLAQQITRQISANRLPKAQP

ST25 4190 cg62_0687

MTISHIDPEYQANTIEPSVQQDWENRKVFKVADTVEGKHRYILSMFPYPSGKLHMGHVRNYTIGDVISRFYRLKGETVLQPMGWDAFGLPAENAAIAHKVAPAKWTFENIAYMRDQLKKLGLSVDWDREFATCTPEYYHWEQWLFVQLYKKGLIYRKLSTVNWDPVDQTVLANEQVENGRGWRSGALVEKRDIPMYYFRITDYAQELLDDLDTLQDGWPQQVLTMQRNWIGRSTGMEITFPSANTEIYADGLTVYTTRADTLMGVTYVAVAAEHPLALKAAENNPELAAFIEECRMGSVAEADLATAEKKGMATGLFVKHPVTGEELPVWIANYVLMSYGSGAVMAVPAHDERDFEFANKFNLPIKQVIDAKGADDADYSATEWQEWYGSKEGKLVNSGEFDGLEFQAAFDAFLAKLEPQGLANSKVQFRLRDWGVSRQRYWGCPIPMINCDTCGQVTVPEDQLPVVLPTDVVPDGSGNPLNKMPEFYETKCPCCGGDARRETDTLDTFVESSWYYARYASPDFTGGMVKPEAAKNWLPVNQYIGGVEHAILHLLYARFFHKLMRDEGVVQGNEPFTNLLTQGMVLADTFYREAENGKKTWFNPADIELERDEKGRIISAKYSGDGQEVIIGGQEKMSKSKNNGIDPQAIIDQYGADTARVFMMFAAPPDQSLEWSDAGVEGANRFLKRVWRLAASFLEKGNSATAIDKANLSKDAQDLRRKTHETIQKVSDDIERRHAFNTAIAALMELLNASNKFEAKDDNDVAVEREAITTLLTLLAPFAPHLSQTLLAQFGTDLTEATFPEVDASALTRNTQTIVVQVNGKLRGKLEVSVDISKDELLAQAKALPEVQQFLTGPTKKEIVVPNKLVNLVV

ST25 4190 cg62_0688

MNLSYLKTSLYIAATTAILLCSSQSQAQQYYKWMDQSGSTHYTTTPPPKGAKHLNKVSTYGSQPLLKNPTSNSEQPSQDKDKVVQEVTNVAVEKGAPAVPVPPAPSVSAPR

ST25 4190 cg63_0690

MNIKEYDYIIIGAGSAGNVLAARLTEDKDTTVLLLEAGGPDYRLDFRTQMPAALAYPLQGRRYNWAYLTDPEPHMNNRRMECGRGKGLGGSSLINGMCYIRGNAMDLEQWATHKGLENWTYADCLPYYKKAETRDIGGNDYHGDSGPVSVATPKNGNNVLFHAMVEAGVQAGYPRTDDLNGYQQEGFGPMDRTVTPKGRRSSTARGYLDMAKGRPNLTILTHATTNKILFNQKQAIGVEYIIGADQNNLQRALVKREVLLCAGAIASPQILQRSGVGQSTFLKSMDIDVVHDLPGVGENLQDHLEMYLQYKCKQPVSLYPALKWYNQPAIGAEWLFNGTGIGASNQFEAGGFIRSSDEFKWPNIQYHFLPVAINYNGSNAVKEHGFQAHVGSMRSPSRGRIKLKSKDPFAHPSILFNYMSTEQDWREFRDAIRITREIMHQPALDPYRGDEISPGKHLQTDAELDDFVRNHAETAYHPSCSCKMGEDEMAVVDGQGRVHGMNGLRVVDASIMPLIITGNLNATTIMIAEKIADQIRGREALPRSTAPFYVAS

ST25 4190 cg63_0691

MSDVQVHQLYIHGRYVEATSGKTFNSINPANGEIIATLQQASEQDIEAAVKSAQQGQKIWAAMTAMERSRILRRAVDILRERNDELARLETLDTGKAYSETSTVDIVTGADVLEYYAGLATAIQGEQVPLRESSFFYTRREPLGVVAGIGAWNYPIQIALWKSAPALAAGNAMIFKPSETTPLTALKLAEIYTEAGLPDGVFNVVQGAGREIGQWLTEHPVIEKISFTGGVETGKKVMASAAGSTLKEVTMELGGKSPLIICEDADLNRAADIAVMANFFSSGQVCTNGTRVFVPKSRLADFEKAVVERVKRIRIGDPMAEDTNFGPLTSFPHMEKVLSFIESGKQQGAKVLIGGGRATEGELAKGAYVLPTVFSDCTDQMAIVQEEIFGPVMSILSYETEEEVIQRANDTTFGLAAGVVTQDISRAHRIIHQIEAGICWINTWGESPAEMPVGGYKQSGVGRENGLTTLGHYTRIKSIQVELGDYQSIF

ST25 4190 cg63_0692

MTKRRVKPEHVRREEIMNAALDVIYEVGLSNTTIAQIAKKAELSTGIVSHYFGDKQGLINTCMQEMLNVLRRKTEQYRAEADSHPESQIKAIIDSNFDISQVNEKAMRVWLDFWSASMHVPDLSRLQKINDQRLYSNLKFYFLKLMDEQQASVAARGLAALIDGLWLRGSLSRHNEFDSELARSIAYDYVKTQLQFANKPLQERQNE

ST25 4190 cg63_0693

MVLKSDGYSSDHIRLNRFVFWSSAVSIGIFGLLFVLFPEKSQFWLTYVQEQVNHFFGWYYMLVIVLCLGFVAWLAFSKVGQIPLGKDHDKPEFGYLAWTSMLFSAGIGIALLYYGVAEPVDHFLRPPEGEAGTIQAARNAMTYSFLHWGIHGWVLYALLGVTLGYFAFRQDLPLALRSALYPIFGERVHGLVGDFVDGFGILATVISLVTNLGIGALVLVSGICYLIPEIPDNSATLITAVLIMMFVATVTTVVGIEKGLAWLSRINLRLLYALLLFVFLTGPTNHLLNGLVQNTGDYLNNFMGKSFDMYLYNQKASGWLGSWTVFYWAWWIAWAPFVGMFIARISKGRTIREVVLGVCLIPLGFTLAWISIFGNTAIHLILNQKQKVLGDMVLSDPALSLFKLLEYLPFNPYIAGIVVVICFVLFLTPVGSGTLMIANLSSKGGTSDSDSPIWLRIFWSVVITIVSIGLLLAGSFNSMQSAVVLCGLPFSVIILLYMFGLAKALKQDDFDPNVAKKQLAISKDISIPSNCDQNKVTEVL

ST25 4190 cg64_0695

MRFSMPKLISEKNQLQQLSLNSNGGVVYQPARGFFKVVYQGLILPNLPAPLRYFNYISLIGQPRIPLCYNANGIVTSAVDTATVLVSNSLHSVGHLKTYSIRRQCQLNPSRYQFDKTDLIEWQIPRVQLRRIDSEMSCDLIVQTPSDISNSSALQWGISDYWSILCHCEGEILYKGQKYEVNGLGRFKHARALHLPFLSLCFYTCQIINLNETTQVTLSQIRNQWNIILFSRLDIQELGKPPVTFTEDVNFHIHRVYPKVETPHGREMYLPREFSWQCKKNGKVIFELYGESRGDYKFGLAAGYIGSFRYQLSWNDQCLQGEGGYCEYIDCRPLRWQEKNQNEKMLDKLLLLQPCLYKK

ST25 4190 cg64_0696

MTNAPKPEQDLTHNEHSFDGITFEVVEEEDNPEGQKVKRRGIYLWPNLITTAALLSGFYSIIASMNGEFAQAIYAIFLAALFDGLDGRVARAIGAQSAFGEQYDSLSDLLAFGVAPAMLMYSWSLHDLGRIGLACCFVYTACAAFRLARFNVQIGVVDKRYFIGIASPLAAIIIISLVWVARDYPFIFDLRDIAIQTINAVIMVVVGLLMISNIKYYSFKQMDRKRVPFVVMLPVVLIFAAITYNIPMGILIVSIIYALSGFVTTLLAKKNNETIKT

ST25 4190 cg64_0697

MNYRHHFHAGNFADVMKHVLLLQLLNRLNAKDKPYRYIDTHGGAGKYDLSQAPAQKSGEFLTGIHRLVQLSDMEKRQAPEAIQQYLKLVEELRAQEGKGSYPGSPWFALQGMREIDKATIFEMQRDVFQQLRHNIHDKRAGLHERDAYEGLLAVIPPKEKRGLVMIDPPYELERKDFPQLVELLQSAYKKWPTGVFAVWYPIKDRAMIERFEKKMFKTGIRRQLICEICVWPDDTPVGLNGCGLLVINPPWQFSEQADQALQWLFPHLRMQETGGHAAVRWLVGE

ST25 4190 cg64_0698

MRSQPLPDCWNLDQILDDLNAHGFAIVNQAYSAEYHTQVAKECSHHFDEFREAGIQNGVVSTIRSDHILWINESLPVAEQHVETLSSFCQHLNQAFFLGIKEVEAHFACYNPGEFYALHRDNPQNKK

ST25 4190 cg64_0699

MSTVYYLHPEWQDDWGGQLRLQDKNNIWHIITPEPNRLVIFQSNLLHEVLVSKQQRLSITAWLRSGNSIWV

ST25 4190 cg64_0700

MSIETKAIVSRIGETDQLYLTENTPELALERAELRMQLVTLSRVRQEQIHFLQEAIVLLEQARMEYEEMPMSLYLNLSLHLAKAYMLYFELNKEKRFALIAQQILKPLAHHQHGDIYFFLAYASAAQQESALTRHWLTKYLSTAQCDLELLHEHPIFNPVRHEPWYKNLIKLRTH

ST25 4190 cg64_0701

MFEQQPTLQLLFEQLGLDSDDASIENFIRTHQLPAEQKLHEASFWSQGQSDFLKSHWEKDDEWIVVIDELNEQLHEDSVKKITNKKGLL

ST25 4190 cg64_0702

MKQPVRVAVTGAAGQIGYSLLFRIASGEMLGKDQPVILQLLEVPVEKAQQALKGVMMELDDCAFPLLAGMIGTDDPKVAFKDADYALLVGSRPRGPGMERADLLKVNGEIFIGQGQALNEVASRDVKVLVVGNPANTNAYIAMKSAPDLPAKNFTAMLRLDHNRALTQVAQKAGVAVADIEKLTVWGNHSPTMYADYRFATANGESLKDKINDPAWNKDVFLPTVGKRGAAIIEARGLSSAASAANAAIDHMRDWALGTNGKWVTMGVPSDGSYGIPEGVMFGFPVTTENGEYKIVQGLEIDEFSRERINFTLNELEEERAAIADMVK

ST25 4190 cg64_0703

VQGYVMHVQMTPAVCALDPSKQKQRKCLEGYSLTITGLIPETTRTDCSTQSSATLSPLQAKVVARVMPDNNARVQLWRSVGGCVPMNASQYFRTIINFAERLKIPANLTSSTNVEMQQSTLRQQFTKLNPSLPQNGIRFTCQLSRSDVVLTEVKVCYTVNGQYKQCSNHVVSNCPSEITIKGSY

ST25 4190 cg64_0704

MKNKYAHRSWLGAVCLLGCSLSYAAEEQFNDALNAANSGNTALLDQYQLAMQNDVLGYYPEYWKLNTNLGFQSPTSIVSFAQRYPQSAMAEKLAADYVEEKVKQADFASAQPILPYVSNPDQAENCALAQVRAKSGDALVFAEYKDVWLATESQPESCIGLGRMMLSSPLMSTQDKQQRLWVQLRAGLSGQALATAQTLGLNLSLAQLNQIQANPLNYLWSAPKTNDVDYAYLIFALGRLANNDLGNAFANVQRVAQGTPESVQKYLYRTVAYIGGTTVMKNNFNREVLQYFDASYGYPLSPEEAEIYARQAIRFSAWESLIRAIDSMSVSQKQEDRWQYWLARATEQRGDSNSKNTAHRIYKKLAESGDDYHNLLAKDRLGVRYNHQPYNDEPTASDLRRLDQNIHFNRAFTLRRINANPTYTNREWNWAVRQAYLQHDDGLLLAAAKRAHDMGWYDRAIYAADRTTNKHNDTYRYVTPHKTNVVSHSYNAGIDPAWAYGLMRQESRFVTSARSHVGAGGLMQIMPDTAKLIARQMGETYNPAALSEMNTNIRYGTFYLSMIQGQLSNNPVLATAGYNAGPNRARRWQPDYQPIEADQYTETIPLLETRDYVKHVMTNATHYGVILGQGAQSLIQRMKVIPTRSSP

ST25 4190 cg64_0705

MTVQTFIPNGAKAASENTVTQPTHTTDVSIKKLYIETQGCQMNEYDSHRMADLLGDSHGYVLTNNPNEADILLMNTCSIREKAQEKVFSELGRWRKLKEQNPDLVIGVGGCVASQEGDNIQKRAPYVDMIFGPQTLHRLPQMLDQHHAQVEKPKKEKIKLVDISFPDIEKFDFLPEPRVEGFKAFVSIMEGCSKYCSFCVVPYTRGEEVSRPLDDVLAEIAGLAEKGVREISLLGQNVNGYRGETFEGGICTFPELLRLVAEIPGIGRLRYTTSHPLEFSDELIQCYEDLPQMVSHLHLPVQSGSNDVLKAMKRNHTIDVYIDKIAKLRKIRPDMHLSSDFIIGFPGETDENFAETLQFIKDLDFDHSYSFVYSKRPGTPASDLPDTTPEHVKKERLAQVQQVIKQSSIEKTDAMLGKIERVLIEKVSDQDPNILVGTADNTRLVTFVGDASWIGRFAEIEITEIKTLNLVYGELLNLEPDVA

ST25 4190 cg64_0706

LTAAIRRTVTFPEISMERLKSILGAYNGHLKQIEQRLDVKITHRGDVFYIDGEIDAVGRAEALLQRLYEETEASQQISADLLHLLIQSSQTERNFELVGEEMDEHDAPMDVYFQTRKGRINPRGANQKRYVQRILQSDISFGVGPAGTGKTYLAVAAAVDMLERNEIQRILLVRPAVEAGEKLGFLPGDLTQKIDPYLRPLYDALYEMLGFEKVAKLIERQVIEVAPLAYMRGRTLNHSFVILDEAQNTTPEQMKMFLTRLGFGSRAVITGDITQVDLPRGQQSGLAHALRVLENIKEIHITRFHSRDVVRHQLVQKIVEAYEGWDGEQQRLNAEARAERKARQEALIAENDTAADLQHQDA

ST25 4190 cg64_0707

LKISLSLQQDFQSPELELKRAQLKKIIETTLRHVGYKEDCEIGIACVDLEESHQLNLQYREKDKPTNVLSFPSDIPEEVLPMLDALPLGDLVICIPVVLQEALEQKKTAQNHFAHLLVHGVLHLLGYDHETSDEDAEEMEGLEIEILAKLNIANPYQE

ST25 4190 cg64_0708

MLKKRSLSIEPFYYWWQDRVFIAAIILAILLHIFVLLIHFAMPAPSEQSTKEIAISIRPTEDVIKHADFLAQADQQGSGAFREAHRMSSNSPAPMPSDASTGEAQLETLEKVQQQRELKFEEKVLMTVLSWQKQAEESQRKKALEQLQSQFQAKAAMVASLEAQYLQRQQDFSRQQKIKTVDGIQAKKDASAAYLDKFREKVELYGNRYYPEEAKQQQLKGEVRLMVILNAQGGIRAIRLLESSGHPVLDEAAKASVRRGAPFGRFDANMKDISELRIIRTWRFDPAEAEFEVH

ST25 4190 cg64_0709

MQTLFSSLVKRLHKVYRKAEAFIEEERDFTLSQTFLNATLERYVTNNVEFLDDLHADLYHDWLRLYATMHVKGLETTLSVDLKLIQMEFNKEQQLIVFEQISNTQIVEAKYKNFWQKIAVKAAIFYYHKILKKDPLGMILERFDVAQEKDDLIFLDLNRWFAKKASIIETLGKVHINYARVREAELVVFGNVNLAALLFRDQDDDSEDELGDTEITPIQQKDV

ST25 4190 cg64_0710

MATKFLKSFSIATGISTTLLIAGFSSHALAMSPFQANYQFTYNNKGMGSATRALSQQGNNWTYQFTAKAGGIASASETSKFSFANGKIGSQSFSRTSKILIHNNTMSINFNPANKTISTKKDDKTRSFAWQAGVLDELNAELQIREDLKNGSLKSSYPLADAKEVENRRFVKQGNEKIKTSYGTFDTVKVVLQHKKPERSTIFWLAPKLDYLPVKVSHIDGKTSYGLLLTSYTGKTN

ST25 4190 cg64_0711

VHALEQKILTEGIVLSDQVLKVDAFLNHQIDPVLMQQIGKEFAARFKDAGITKIITIEASGIAPAIMAGLELGVPVIFARKYQSLTLKDDLYRAKVFSFTKQTESTIAISNKHINSSDKALVIDDFLANGQAALGLIDLIHQANAEVVGVGIVIEKSFQPGRDLLLEKGYRVESLARVQSLADGTVTFVKRIKWFK

ST25 4190 cg64_0712

MQPYILVLYYSKYGSTKEMAHLIANGVESAGVNVKIRTVPNIATVVTEAEPSIPEEGDIYCTLEELANCAGLALGSPTRFGNMASEMKYFWDQTTSLWLNGALHNKPACVFTSSGSMHGGQESTLLTMLPPLFHHGMMILGLPNSIPALSNTKTGGTPYGASHVSGPRHDQSLSQDEKILCEAQGKRLGEIVKKLHS

ST25 4190 cg64_0713

MLERFLKKLPFYNKTWFQFILFVIKRFEADRCREQAGSLTYTTLFAVVPMLTVFLVIISSIKALEPARQQLQHLIYSNFLPKSTIAFDKALNAFTEKSSNLTVIGVLFLFVTTVLMLTSIETVFNRIWRVKETRSGIVGFMRYWTIISLGPIILGSAFVISSTVASMNLLSNNFTGYQLDGAFLLWLISFVLTILGFFILYWTIPNRTVPLYAAAIAACLSAALFETLKNLFSFVMSNFTSYEIIYGAFAAVPIFLLWVFLSWNIVLLGVEVSFALTAFHSGKEQKRHPVLMLLDILELFYKKQKLGESVSDKEALEILGRGEVGRWPAYVLLLEEQNLVKRTDKDEYVLVRNLSQVDFWSFFTALPYPLPLRQDVSNVHDDDEWMEKIGPALVESNDYLAAKLSIPLSTIFEEK

ST25 4190 cg64_0714

MEERKNLDTRASALMVLLCMVLGLQQVVLKIAAPDISPIMQIALRSGLSAVLVLPLLWRDHSIHLFSYRQCKAGALVALFFSLEFYFVTQALKFTSTSHTVVLLYTAPIFVALGLHWKFPTERLNRAQWGGIVLAFMGIILTFYPTTTATNAPALAQVLLGDLYALMAGLAWALSTIIVRLSPLAQAPATQTLFYQLFGCFVLLLLIAFLTNQTTIHFTTLTILSLSFQTLIVSFASLLLWFWLLRNYLASRLGVFSFLTPLFGVLFSILLLEEKLEIKFIFGSILVLSGIVIMSLKQGKAKSS

ST25 4190 cg64_0715

MKKSAQRVQDFQEIPAHNAIPDLLWFRVRNAPAETVYPQHAHAWGEFIYAFSGVLEVNIDQINYLTPPPYGIWLPPYTKHSGINRNEVTHATLYVHESLCQKLPQKAGILLTSSLVPALLEHLRKNPQDETQVEYIRLLQVLLDQLIQAELVGSYLPTTDHPALAKILNDLHQCPADNSTLQELAERINMTERTLARYSQKELGMSLHEWRQRLKVMKAMTMLNQGKTIESIAFDLGYASASAFIYMFKRWMGFTPDQFRKLYQ

ST25 4190 cg64_0716

MKTRPSKIVCVGRSYADHVKELNNAMPGRPVLFIKPPSSLIGLDEGISWNPAWGSCHFECEFVLRIDQPLKGETDPQKVLQAIGAVTLGLDLTLRDLQNELKDKGQPWERAKAFDGSCVLADWVDVSEITNWEETRFNFHINDELRQDGNTALLMFNIAYLLVDINQVFSLEPGDVIMTGTPAGVGPLNAGDQLKMTLKGATQDFVWETFVKA

ST25 4190 cg64_0717

LTPVISIAIIIWGLYLGFSGVGRYYVALGVFFLVMQGLMRYWLLPMMFKRQFVRYQFGKSEQGIDLYQDYFELYAAGRKQAVQYSEVQTFAKGKLTYMLELKNKTVIIVPKRAFAQASDQTIFENTFKK

ST25 4190 cg64_0718

MTEQKSALSLRYYLNLEESQDGFGLVTFGKEAIHTVFDTCY

ST25 4190 cg64_0719

MSFFAKLFGFKQKTTAQEDVALKAAQELEPSNETSFIELIKNRRSIYAIGNNLSQSNDEIEKLIQEAIRHSPSAFNSQSSRAVILFGQSHHKFWNTVLEVLKTIIPAEAVSGTEQKIQSFAAGAGTVLFYEDQSVIKSLQEQFELYADNFPIWSEHSSAIAQFAVWNVLAEQNIGASLQHYNPIIDEKINTSFNIPAEWKLRAQLVFGSIEGAAGEKAFIDDESRFKTFG

ST25 4190 cg65_0721

TFETSRDTVNGALEVVSDLVGGDIQGATENATGILGTLIQNGSTATGI

ST25 4190 cg66_0722

VCDLVGGDIQGATESATGILDTLITNGTTATGLVTEIIGGVTGTIGGVTGGDSPLGLVTDLLGGLTGSVGGDSPLGVVTDLLGGLTGSVGGDSPLGVVTDLLGGLTGGVTGGTDNPIGIVTDIVGSLTGGVTGEGSLDVISNLLGGLTGGSLLGGVTSTVSSVTNTTHTIVPTSLLTDNFLENSFNTV

ST25 4190 cg66_0723

MNLALFDFDGTITHNDTFSLFLKFSLSKKTQILGGIRLAPYIAGYKLGWVTDKTIRTKLCQVGYIGYDAASLIYKGQEFAKKVIPSCIRENALERILWHKQQGDQVVVVSASLSVYLESWCKSLDLDVICNQLEIDNGILTGCFIDGDCGYLEKVNRIKNKYDLTQYSIIYAYGDTPNDYAMLELAHRKYYKWQEIN

ST25 4190 cg66_0726

MKPNDMTKNWVDPEAKAEAERYDNPIPSRTLILDTLEQLQTPQSHAELVDHFHIQDQKSIEALSHRLSAMVRDGQLMKDGFKFQLVADQPTYEATVYINSKGLGTAHIDGQDDLLLPERELRLVFNGDRVRVRQTSVDRKGKPWGFITEVIQHRVKQLIGKLSVHDGEYFIQPNNPNQHQPIPLEKELVEHAKANVGDMLRVAIDTYPTREEFATAHIIQSMADKADTEIIIPQTILEFGLPYEFPDEVIKEAESFKEPAEKDREGRVDLRDLALVTIDGEDARDFDDAVYAEKRPGGGYRVVVAIADVSHYVRLDSALNEEAEERGTSVYFPHFVLPMLPEALSNGLCSLNPHVDRLCMVCDLKLSRTGRVTGYEFYPAVMHSKARLTYTQVGQYFEGATDAIPKDRDIHKSLNTLFQLYQILKNLRVDRHAMEFETIETYMTFDELGGIKEILPRTRNDAHKLIEECMLLANVAAAEYALEHDIPMLYRVHEAPEFSRIQKVKDFVKLLGLPFPDQPTQADYQKVIEATKDRIDAPSIHAVLLRSMMQAYYGAKNAGHYGLAYEAYTHFTSPIRRYPDLLLHRAIKAHLKQKPYPLSGAALDDAGEHFSQTERRADEASRSVTTWLKCHYMQQHLGDEFIGIVSAVTEFGLFVTLKDLYVDGMIHISQLGDDFFIYDQASQNLIGQNRGQVFGLGDEVKIQVAGVNLEERKIDFQLIKQITHAGRVVRSRAPRTTKTSTQATEEVFGKPSVSTDGEKPVRKKKEKGKPSSYSKKSGKKTSTKAEAKPEKVKKKAKPKKKKYKRQV

ST25 4190 cg66_0727

MWLLAMTLQQATLPTPRFDQITLDNLKQDIQQAIQAGQEFLNTLTAAPSETDQQLAVLEQIDALENNLSESWGVLSHLNAVMNNAETREVYQSLLPALSEYYTQLGQHTALYQTYQLIHDSQSYSTLSPAQQSAIRLALRDFKLSGVALEGEEKKRYAEISARLSQLSSDFSNHVLDATQAYFKPLTEDQLKGLPESNVELLKQYGKQRELDQAVATLDFPAYFAIMTYADDRALREELYKAYVTRASDQSEQTEFDNSKIMEEILSLRQEMAKLLGFNNYAEYSLASKMAPDVKTVHDFLVDLAEHAHTPALQEVAELQAIAKQDGIDELKPWDTTYYSEKLKQQQFNLSQEALKPYFPAPKVIQGLFQIVQRLYGINIVEREAPVWHPDARYFELEDQGEVIGGFFFDLYARTGKRGGAWMSGFRSRMQTVHGLQKPICYMVCNFTPPVGNQPALLTHDEVITLFHEFGHGLHHMLTEVDNISVAGTHGVAWDAVELPSQFMEFWSWDKESLDVLSEHIESKQTLPQELLDALLNARFFQSGMQTLRQLEFALFDLTIHTKTPALNSEQIQQTLNDIRKQYAVVPTVDYNRFQHSFSHIFAGGYAAGYYSYKWAEVLASDAFDRFENEGIFNTQTGKEFRQAILAVGGKDTALEAFVNFRGREPKIDALLRHQGWTNDNKTA

ST25 4190 cg66_0728

MKKRFIAGAKCPKCEAIDRIVMLTTAEDEWIECIECGYSENRPTHIDEPETPAIPDEIGVIQFKPRRSD

ST25 4190 cg66_0729

MPLQRKQNSKLLWILIGGLSILVVLFFAAQWFFKKTETSAPMVDPQPASVPAPEKTTKPSESSEPVVQTQTANSQQLVNEDVLKQPIPAQESLAKEEVSKLNDIHHQLQDQEATLKAQHADADQLIKLKEEQVKLLEAQVKQQ

ST25 4190 cg66_0730

MQEYQEKRSGLGLLGYIWNEWISTFIILILLLMTLIIGTGEMIHGQLLRIGERLYGDPATGMQYSFLRAEPEKPTCDRHPNIDAQVKEQMKANASDDFASFFGAASESDVRASLLAAQQQCEEKYQAYDKTIKYIEANPGVRTYRAIETGFFGIFKFGTENRAILLVFMVLISAITASLKYHHIGLRNPATKIDYKVYSLFMLIGNALLSFSVISQYRSVINSGVTPTYETVTIYWIWMILFVVLTLISLYQLFVSPPPKREGGNIGLALLSVPLYAYMALITGIVFTFFMDYPMGQGIYLGLLVEFSGIFLNLALFIWAGMLLTQTRVMDLFLNVLRPWNLAPETLTWLILIAAAVPTAYTGASGIFVIAAGAIIYKEVWNAGARRQYALAVSAMSGSLGVVVRPCLLVVLIAMLDSRHVTSDELFGHGIYVFWLTAFIFLGVSLLLAEEKFRVNSPKVALPGMARAFVPVIPYILITVVVLAFYKYALDTGMNEFTAPVILPLVLIAMILYDKMVMPKEAPALNIHEAIVREHEKKSPFLQTHDPHSSQFRLGFGGALRFATSETVGHIGALIILMALSASVGGLIERSEVVELLPTHLGSIYISLACIALLLAIIGMCTDPFGAVILVAATIAPVAYENGIHPIHFWMIVLVAFEFGYVTPPVALNHLLTRLSVGDDEVNAADAEAKEKYTSFYYRYERWLLPIIVLFSSLVLVTYAPLILKLFGWYK

ST25 4190 cg66_0731

MKDHVLNKKVLLVAGCISVSAYLQVFPDYFIYWHESLLGEFWRLWTAHWVHVGWIHFLLNMMAFACLPFIFPHTKVWHILSLLFLLPPFISLVFYFYLPNIDAYAGLSGVLHGLYTAVALVYLQYRKERNFAFLVLGLIVAKLIWENTFGQTGTAQLIGSPVLTEAHLYGAIGGAIFGGCYWLIQRFK

ST25 4190 cg66_0732

MSKVQKICQGLAVSFLAASVLSGCSYVVKSGANVALGFTEKHVVPPILAMQDAEMVCNTGSALTPAIMSTKGMGADPTRVAVLMYAASGVCAENQALEAELRYLRASKAGQVTEAQDARIAQKRWAAIAAERQYAGYKLFADRWESKYKYHLGDSCPTMRSDIDQTVYLLGMISGLQAVTNDINSGGSVNVPKDIAAIVERGMVCLDNQKFWGAPNATRAVIWTLLPGANEGKPDPYETLKQSVQIGEQKGVRLSHALYAVAAQASGDDAKIRDALKTYAASQADDKPVNPNFKLIDSMAGLIVRGISDRYWTEHTGVRTGDDGMSHFWDEKNEGSAELDELFDGGAASEAPADTKTAPAQ

ST25 4190 cg66_0733

MEISMWMFLLIAVIMAVVVGRAGTLLKDYLEQQ

ST25 4190 cg67_0734

MQVYYFYRHQSDGYVFLSREKHTEHILEFFWIDSVRSDVVSQNEEWQQKIQQLADVSINEFHMRDIANIEHPCAFDTLEEYDLLIFRKLVTPDDEIKNGESHESVFGLATTPISFIFTPKVLISVREQGNKSIENYIQRLENILCKTLEEQNKTRKLPNSPVDLCLRLLNSMVDGYLDIRSPLTRRVEHWQQQLLQGNRRFKQWHQLFHENMAFQQVENLCEEQIETLQEFRDEIVENYHHVIGEKTHSSQDLLLVRLNDLMSHVERIQKHTLRLRSAIQSAIDLHFSAIANQTNENMRILAIITAVFAPLTLLTGIYGMNFEFIPGLKSPVGFWIMLGVMLLSTILLLYYFYRQHLVGRGEKSVIDLLAQQHRQDRMNLFWFLEYEPIKQTLKEVEKITRLK

ST25 4190 cg67_0735

MFNIVRKEFQFGQHQVVLETGRVARQANTVLITMGGVTVLVAVVAAPTAKAGQDFFPLTVNYQEKQYAAGRIPGGYGKREGRASEAETLISRLIDRPIRPLFPEGYYNEIQVTATVVSSDKTMEADIAAMLGTSAALAIAGTPFRGPIGAARVGLINGEYVLNPNFEQMAQSDLDLVVAGTESAVLMVESEAKELSEDQMLGAVLFGHDEMQIAIQAINEFAAAAGAKPSDWVAPAHNEELRAKLKEAFEAKISEAYTIAVKQDRYAALDALHAEAVAQFVPEEDVDGIADEVDYLFEDLKYRTVRDNILSGKPRIDGRDTKTVRALDVQVGVLERAHGSALFTRGETQALVTTTLGNTRDALMVDTLAGTKTDNFMLHYNFPAYSVGETGRESGPKRREIGHGRLARRGVQAVLPAADRFPYVIRIVSDITESNGSSSMASVCGASLSLMDAGVPLKAPVAGIAMGLVKEGERFAVLSDILGDEDHLGDMDFKVAGSANGITALQMDIKIEGITEEIMEVALNQAFAGRMHILNEMNKVISRARPEISMHAPTFEVITINPDKIRDVIGKGGATIRQITEETKAAIDIEDNGTVRVFGETKAAAKAAIAKIQAITAEVEPGKIYDGKVIRIVEFGAFVNIMPGTDGLLHISQISNERIANVTDVLKEGQEVKVQVQDVDNRGRIKLTMKDIEQA

ST25 4190 cg67_0736

MALTNADRAEIIAKFARAENDTGSPEVQVALLTAQINDLQGHFKAHKHDHHSRRGLIRMVNQRRKLLDYLNGKDHERYTALIGALGLRR

ST25 4190 cg67_0737

VKIFSTNTCPVPNDLESVIHHKDEVPAELGGMSEQQVQKIWKSIESLYKTGNYPLITFCLRRQGKILLNRSIGYAQGNSPAGLQENALVATPDTPVCLFSASKMITAMLIHLLDEKGEINLLDPVSYYIPEYGVNGKRRATIFHLLSHRGGIPYVDGDVTPELLFDKDEILRRLYAARPVSPAGNHLAYHAVTAGYILGEIIERVTGQDLRQFVHETIEKPMDMPYFNYGLKPEYRAEVALNCATGLHPRLGTDHYLNHVLGGGLQLAVDVTNDNRFMDTICPAGNIYTSAEQAGRFFEMLLSGGSYQGKQILSEKTIFRATLPTTGVNIDRTLLIPMRYALGPMLGSNPVGLFGPMTGQAFGHLGFSNILCWADPERDISVSLLTTGKSVVGTHLPALANLLYQISTQCPRIPRDQRRSLFGSDSHETDLV

ST25 4190 cg67_0738

VDNLNSPEQSDLSWLTTWSTFLNQAPFTAQTQAPEAAYFLQQLIEASLQGDSCIEISPEQIETLGQLVISAEQAKSQVAPCVHDGQGLALYRYWNLEQRLAEQIRRLKQQPIQPVSCEEHLDLLTDPHQRAALQMVTRQSLSIITGGPGTGKTYTLARIIAVLSQAIPHIRVAMAAPTGKAAQRMQEALQNSFNDPKLLESGLMSDELRNQSTQTIHRLLGMGHSQTPRFNQKQPLPYDVIVVDEASMLDLNLATLLFEAVPESCRIILLGDANQLASVDVGAVLADLQQIEALSENRVHLQTSRRFAEGALIGQVARFIQMQTGQIDHEQVLQQFEIDIVQVSELRSISLEADMPDVVQLEYLPEESPIALETYYQKLMVGFQGYVQSLKAYIQDDRSLEHIENIVKSFDDYRILTAVRHGPFGLQQLNQYAERWLQQQLGIVTLGGWYVGRPVMMTYNDYQLGISNGDIGLCFEHRTQPQQFEVYFPSLNKWVAANRLPKSIQSAFALTIHKSQGSEFTHTAVVLDQAAKNLLSQELIYTAITRAKKVVSLLVDPEALLQSFTVRTTRKSGLVEKVNRLVR

ST25 4190 cg67_0739

MNSRVQVSYQPIIDIEFSGLHLIEASAGTGKTYTLSSLMVRIFLEKYLPGQVIATTFTRAAAAELKSRIRARLVETHRYLDTKRSLTEKEILLQAEQESDLLLQHILKHFATRIAYACERLKLVIDQLDELFVGTLDSFSQKLLREFAFESGKIERAQITDDAKTYSRQLIHDVLREWIQSQPQTVIDALYLAGELKSVDSFVKLVEDSLNFSSAHFKLPEKPTIQFEQLAQLKQLAAEIDISLLEPYYSLDGEHYKHVSGTIFRNGAFNKLFSECLPQLLQVLKQSDSILIFDGSLAVHRELVFKFLGQLADQKVFKKCPAEISDGFYQHPCIQQIQQLFGVLKNYAEQFDQLHIYLKAYLCVEVKKRLPQVLQNKGETTFSQQIRTLSEALKGEQGQRFAVFVQARYPLILVDEFQDTNQDQDDMLASIWRHPERYQKGCMIMVGDRKQAIYGFRGGDMLTFLNAYKDIQAKHGREYKLIHNHRSVADLVEVVDALFQRQIDFGEQVQYDPIRAGTRPHPVLIDQNQPNPHPLRWLMLKDKETEAQQVAWKIRDLLNQSHAGQLYFQKDAQTQTLNEDDIAVLSRNHDGLDRVQFELERLGIRVNRPSKRSVFDCTIAQDVGALLTAILHPYDEAKVKRALISRLFAMDLKQLLQLEQTAEGLSQFMTGFDTIRELWSAQGFLVAWQQCLNQFGIWKNLVAVQSKDNERVVVNLRHLTEILSQHSEKYQGAQNLYHWYLKQLQSPLDREWELERRLSSEAGVQLMTIHQSKGLEFKIVFLLGADKPFRENNKTLNFSTQDITVPESAQTLTQRVVAIADKTYLNETELKQHEERALAEQNRLWYVALTRASHRVYALLQDTDGKSVSGLAFWKNRAEPFQHRCCTDEIILEQPPAARHLNQHINIIEIQAQHFPDQRFYSRGKTSFSYLAQHLRHKVGTDLLASQSHEAVLAEDELDQVISVEAPAAQPISWIKSNFPRGTLAGNFLHEIFEHIDFQCSDEWVSEIRRRFKNDYSSLWQDLLIKYQESFPEEQEAEYSLYHAVAEWLQEILSTPLYQGFRLNRLQPEHYLSECPFYLALSDRVLAMKRIQQLFAEYGMEMPELLEARSARYLNGSIDLVYFDGQRYHIADYKSNYLGENLADYSVESIAQSMSLASYWLQAGLYLVALHRYLKVKMQNYDIEQHLGGATYLYLRGMNGEAKQGYYYWQPSTEFVLRLDAILGYFAEDKIA

ST25 4190 cg67_0740

MGIHVIQSQRIDVLLQGVLASTSQPSTHPLQVLKAQHFIVPSPAIEQWLIQKLAEQQGMSANYQFHQRVRGFQWYAYQQVLTAHKEQVRKANIPRLIFKWRVHQALQEFIQPDVMSIDSSHPLHSIVQRIYDSADRLEQGIEKQLKKQKMLYWVAEQVADLFSNYMVYRGQCQRGCENMCTCPNNWLAAWGQGRALDIEKYIAHKDKEVSAFTLQQTQELERWQRWLWQQHFHDDFMQMQQIDELFWQELEHPERQKKALARLPQQIVIFTLLDLPPSQLQFLRRLGQYIDVLILHYNPSQEYWADSVDPLWKQRYDLGVKERFIAKNPQATDAEIADFFNKFTLNFNAEARESRHPLLTRLGKQARDHFSLLSNLSTGEEGKWVDAFVDDFPESLLGKVQSDILHLVEPQAKQYELAPNDDSIQIHVCHSTLRQLEVLKEQLIGWLAKPHEQPRRPNDILVLVPNLAEVEPLIRSIFPATATEQGVHLPVKIAGIASLDALNAWRAVIGRIHLMQGRFSFDEFADWLGLHATQQRYALEYAQVERILNLLADAGFKRGLDAEHLKRSLCDGDDDYRYSFKFALERLALGIAIPEHATFNQVLSYAKVQPGDFELIGTLIQIYQDLNERRDWLIMHEQKKSHTVEYWLQILSQDIIEFEQAGVAALKTAREIVKKQERMLTLASYYAETETGTLRKITLPLPYILDEIQRTLENQTAQAEPTGQITFAQIGQIRPLPYRLIVMLNLDAGQFPNRDTHVPFDLMDALRQQLGDRSRLEDDQGAFLDALLLAQENLWLFYNGFDVNDGEVRDPSSILQEFREHLALIVKPEPNAPDREVIEGIEIPTQLKQLYHLHYLQPFDPKGFMAENRYIRYQDHWFNVAMQIQQASGVLKPWANISYPLEIPDMLVLDSHQWIQDVTFPARLYLKTLGVENLGNVGVLDQNEPLLLDGLGRYTIRHFLQQNEQQAQPEVLLDQLPVGKVQYSAWQQGIFEQECLLERLHHYAPAVTQTTQRVWRIAKQLHMNITVPKSETQDWVSMEASSARAKRRAKVWLEYLLWLAYLNEGSAGTEKRRIVVFSDQTVICKGISSEQARQYLQPWFKIWRYAQQQPLVLPAALLLKPLEKAKAYQWETTNPTEKIKLDEKSYAELLKYWNETGTFTSIDMTQNEACKLHQDWRFILQEQDAQALLQHACDEFAYDLYHPVFQFQHSE

ST25 4190 cg67_0741

MKIDNIPDYIDPSLNLEQIRDECQELIQKRAYVSAGAAIVPIPFFDVVVDLGILSQLIPDINSRFGLAPEHVSVYDPKTKTIHWDELRKRGFQFSGFVVARTTVKKTFNGFFGKIVTKQVTKFVPLGGQLVAASLGYFMMKKIAQTHLNDSYNLAKRIQQKSRGTVVN

ST25 4190 cg67_0742

MSEQQPKFEIRDEEQLSVDLIEAQYALKESRGKPNAKSTLILMSGIELAGKGEAVKQLREWLDPRYLRVKADAPRVLTDSEAFWQSYSAFIPTEGQIVVMFGNWYSDLLVTATHVSEPLDDARFDAYVENMRAFEQDLKNNYVDVIKVWFDLSWKSLQKRLDKIDPSEQHWHKLHGLDWRNKKQYDTLQKLRRRFTDDWYIIDGEDEKQRDQFFAQYLLQHMRQLPEHETEVKGKWQQAKIPESLLKPAQEKMDKAEYKKELDKLSKKIADTMRFDKRNVVIAFEGMDAAGKGGAIKRIVKNLDPREYDIHCIGAPERFEARHPYLWRFWNRINEAEKITIFDRTWYGRVLVERVEGFASPIEWQRAYDEINRFEKDLFDSQTIVVKIWLAISKDEQEQRFKAREETPHKRFKITAEDWRNRDKWDDYLKAAADMFERTSTEYAPWYIVATDDKYTARLEVLRAILKQLRAD

ST25 4190 cg67_0743

MSKLAAFDELLQQYKTFNYHDNPVLAQRLQDVQTWLKERMKETHHDFFNLPEHKLMAQYFLNRLYGGPEFDALAAQIERLLKYAHKAEKVLPENAIKTGTKSVSLAVLATQLDEQVAMQLLEDYPADTVLTDEIMRLTLIKLDQAEARYQQLALLDDLGAALDKYMRSFMMFTAFKMCKGIAQKYHFELMYDFIQDGFSAMKPLKSAEAFIKTFTEKERQIVEKVHSGHPNPFRV

ST25 4190 cg67_0744

MSNENQKPTSPTEQAQSSDKVSPFLSSPLPQGTPQGQQQSLQQTLTDTPVSGSVPKYNLPRGAGNTGNVGETTHFGYSTVRTEDKAQKVAEVFHSVASKYDLMNDLMSFGIHRLWKRFAINMSGVRRGQHVLDIAGGTGDLAKVFSREVGPQGHVVLSDINESMLNVGRDRLIDAGCTNVDFVLANAETLEPFADNSFDLVTISFGLRNVTDKDAALASMFRVLKPGGRLLVLEFSKPVFEPFSKLYDLYSFTALPIMGKLVANDSESYKYLAESIRMHPDQRTLKGMMENAGFQNCDYHNLTGGIVAVHRGFKL

ST25 4190 cg67_0745

MWSILALGAVERIIHHLIDLDALTRIQLNQLQGKMLRVIIDSPQLSVDVFFDQEKVRLEPTVTGHSEKPSIFEQRPFDPQFKISEATATLHVKDVVELIKLLLSDPDQIGNIPLQGDYHLLQDIQKIMQQAEPDLAAHLSKWVGPQLAHEIGKIQLAPKHLKRSLQSHLFFAEDALKEDSGLFAPRWEMDDLNQATRKLNQDIDRLEAKLQQLNAQLQPTQD

ST25 4190 cg67_0746

MIPHVSRLLELWRIAAHYRLDTLFPADELPVKAKHALNIIKMHPAAWSSRERKNPLKLKEALEDMGPLAIKLGQLLSTRRDLIPPEILAQLVLLQDQVKPFDGEVAKQRIQDSLKADVNTLFARFDDQPLAAASIAQVHTAALHDGREVVVKVTRPDIRSQILQDFEILAWLGNTLESRLEAARALHLSEIIQDYRQIILNELDLSIEADNTRRMRHYFTGSTMMYVPEVYMDTKDVMVAERITGVPISDTATFDRLGMDRAQLAEKGLTIFFTQVFRDNFFHADMHPGNVFVETINPSNPRFIALDCAIMGELSKHDQMTVARMLLAVMNSNFMQLIQIVHQAGWIPPGTDQDALAREMRRTVGPMVSKPMDQLDFAGILIQVMDIARRFHLEIPPQLMLLLKTLVHVEGLGTDLYPQLDIWKLAKPILTEWVKSNMNPVKNVKELGQQLPDLLLGAQDFPSLIIDSLNGLKNQSAWQDRQLREIQQLRLQMEHQQRRSWMFGSTMLILLTIAIISPWFVSIILIVLSSLLALWRVLK

ST25 4190 cg68_0748

VFATSGPAYANSKHNFYVLTLSILSYSKWENVSTPTLCVIDNASITSTFQSYIQQLSYNYRVQTVNAKDFSKSHCQAVYFSTTPPQQQQNLIQNYPYRSLLSLSINNPECEVGSIFCLYNQNNYTTFKVNLDALSHSKVHIDPRVLLLAKNAE

ST25 4190 cg68_0749

METYAKQNLQLLSHTLLERIQPAVVFNDKITIEQILNEYTNDHSIRAIHIYDSEHHLIAQSFKLSSQTSVLEKWFDHWFLNEPVHLTIYHHQQNVGELTLFGSSEKILQFLKMIMVGLAIAMLFIVCALWWSVNLTYRQIMQAIYPLTNIAQIVSEQKAYNLRFPYNRIKEFQDLNTVFNELLEEIQIWDNQLQKENRKLSFQAHHDQLTLLPNRHYFYQILLDMFEDKDFDNKSALLFIDNNNFKSINDQFGHLAGDEVLKEMAKRLQTRLRHQDFIARLGGDEFAVILHSITHADHLISIAENLLESCKEPLHLNDQTIYFSFSVGIALSQFASSPEDFIMQADQAMYKAKTSEQHWFIYKPEN

ST25 4190 cg68_0750

LKLSFIALLCIALAGCLSFGPLKYRQVKMLKKEGFVLTNEGWTLGLPERLLFDFNDATLKQSHEAELTRLANQLNKYDLNKLKIVGHTDDVGNPEYNQKLSEERAQSVANLFLTHGFKKENIYVIGRGSTQPYVPNTTNENRAINRRVAIVIIP

ST25 4190 cg68_0751

MWNPNSIIFCFYLIFSILALWGIIEAWVHQSRTETIHPFKAFVHLLAFYLSYLLIPLWFFNLYAGWIGYYSIHETIFIFLLSGILIYARFIEPHMVRIDVHQYRLNPDRRFAKPVKVALIADLHIGLFSGHERQLKIIVKKLNEQQPDLVVVAGDWTYEPEDKLVQELSVLKDIKAPVYSVPGNHDEQYPGPPIQQLLKDALHFNDVMDIEGKIVEFDEFRLIGIGDLWAGKTDMRSMPDLPQDKPWLILSHNPDTVDMVPKLPNRPLMLSGHTHGGQVELPWVTNYIMKKVSILGHKRGFYSHEHADVFVTVGTGMVGIPLRFRVPPTIDIIELV

ST25 4190 cg68_0752

MAQSFHSKQLQTHQLAKGFLIKASIVVCSSFAVALTGCSTLPKHSPEPIQYARDIDTSQTSLSKIITPLREKNPNLTGYHLLNDPLEALAARLRLIDKAEKTLDLQYYIWDNDKVGALALHAIIRAADRGVKVRLLIDDNNAKKMEGVLLALSQHKNIEVKLFNPYRFRKYRAMDMILDLKRINRRMHNKSFIADNQVALIGGRNMTNQYYNVSDSYQFSDVDVMLVGAAVDDIVNSFDDYWNHEYAYSVQSIVNAQQHRLRYESLKQQLDAHYQQATVQNFLDLTSRSHAFDKWLDRNIKFDWVKAEVVKDSPDKIRSKAKKEEHLNFQLINHLEKPESNVDLISAYFIPEKQGAKILSTLAKEGVEVRVLTNSFKANDVAVVHAFYGKYRKELLKNGVQLYEFLPTPDKRDLNKNTDELATKAKVNMKGLSRSSLHTKLMALDEQVFIGSFNFDPRSAYLNTEIGVILDSPSLAKTIHHTMDENLNKYAYKLKLDPNNHIYWQQETPKGPVIYKKEPEMKWWQKAGMKLLSWLPLEGFM

ST25 4190 cg68_0753

VNSTLKKFSKIGLYGKKVTSATAAISEGFYLVYRHGLYKDPNNPVNTRYVQYFCRRLCQVFNLEVQVHGTIPREPALWVSNHISWLDIAVLGSGARVFFLAKAEIEKWPILGNLAKGGGTLFIKRGSGDSIKIREQITEFLKQNIPVLFFPEATTTDGHSVKRVHGRLLGAAIEAQRPVQVCLICYVNRHGELDTVAPFIGEMSFVEHIQRVLEMPKVTAHLLTLPPISVEGHDVKSLTQEVQEKMVEGLAQLHKKVLKPKS

ST25 4190 cg68_0754

VEYVDRHQGGERTILVSVSVQLLDDLDAEEFALLAQSAGADILEHIKVQRNKPDPKFFIGSGKVDEIAEQVQDLEASLVIFDHALTPAQERNLEKILKCRVIDRTGLILDIFAQRARTHEGKLQVELAQLKHLSTRLIRGWSADFEQQKGGIGLRGPGESQLETDRRLIRVRITQLKDKLEKVHQTRMQGRAARQKASIPTVSLVGYTNAGKSTLFNILAKSDVYAADQLFATLDPTLRRLEWDGIGTVVLADTVGFVRNLQHDLVESFKATLEETLEATLLLHVIDSSSHDMLDQIEAVEGVLKEIGADAPVLRVYNKIDLSGEEAKIIYSEPHVPDRVYVSAHSGLGLDLLRQAVQECLMGQLQHFSLVLKPAYGKLRTQLYALNVIQSEHYDDEGLLHIDIRIAPHKLEQLIRQAKLPIDEILGERASQFKRPLEEFEIKH

ST25 4190 cg69_0756

MGVGTTFIEHAPTASFPSNHMLIFSTIALSYLFAQRKIIGIILLFLSFVVAWSRIYLGVHFPLDMLGAFIVALLVNTAGYYFWNVYGTPLTAFFIHLYQIICKPLLDRGLIK

ST25 4190 cg69_0757

MRVRDVNIKVLTCWHFIRERYFMTTQEKQKKLSLRPLSPRDPEQPHRAATPLELLFDLIFVVAIATAGQQLHHAIIENHLWHALPSYLMVFFALWWAWMNFSWFASAYDNDDALYRCLTFVQIVGSLVMAAGIPDVFHSQDFDIIIVGYVIMRLALVTQWLRAAKHDPERRITAYRYAVGIVLVQIGWLVANFAHALSIPLFLLLVVVELFVPIYAEKYSPTPWHPHHIVERYALLTIIVLGESIVGSFNAIRDALAAQSINIPAVFLMIGGLMLMFAMWWAYFDRSEQHHHVKGVRPFVWGYGHYFVFVSIAAVGAALAAAVDVTTHHAHISDLYMGVIVAVSVVLYTSCIWILYEFQCLSGITKWFYPITALIILCIPFICNNVGYSVFAMGIVYTLRLFISNKFMENIEPKQV

ST25 4190 cg69_0758

MHLHILGICGTFMGSLALLARDLGHKVTGSDSNVYPPMSTQLENAGIELMQGYDRSHLQPHPDLVIVGNAMKRGIDAVEYMLNEGLPYISGPQFLADHVLQGKHVLGVAGTHGKTTTTTMLAWVLDQAGLNPGFLIGGVPLGFSESARLGGGKYFVVEADEYDSAFFDKRSKFVHYHPKTAILNNLEFDHADIFDDLAAIQKQFHHLVRTIPSEGRIIAPITETHIDEVLEMGCWTPVIRTSLEANEKAALSAELISIDGSHFKVLENGNVVGEVKWSMTGQHSVANALATIAAAQHVGVSLEKACEALSNFGGVKRRMELLGTINGIEVYDDFAHHPTAIDTTLDGARKRLGERRLWAIIEPRSNTMRMGSHKDGLAHSARLADEVIWYQPEGLDWDLQPVIEAATNHAQVSRSLDEIIERIVNEAGEGDAVVIMSNGGFGGLHQKLMSALKAKAA

ST25 4190 cg69_0759

MMKDVPLSPTVLAFVLLIISNCFMTLAWYGHLKFLHHAPFWQAILFGWAIALLEYSFMIPATRLLSDQGWTLGEMKITQEVVTLIVFVPFMVLLFKQPFKLDYVWAMLCLFGCVYFVFRSQ

ST25 4190 cg69_0760

LAWYKSSFYHFYALEMPMNAVATDTQPLVGIIMGSQSDWATLEHTANMLKQLGVPFEAEVVSAHRTPDRLFEYAETARDRGIQVIIAGAGGAAHLPGMCAAKTDLPVLGVPVKSSILNGVDSLLSIVQMPAGIAVGTLAIGPAGATNAAIMAAQILGLTRPEIAKNVADFRAAQTDKVASNNIPGQV

ST25 4190 cg69_0761

MDKTIGIFGGGQLGRMMAQAALPLNIQCTFFEANTDCPAGVLGQVFSSQDEQGLKQFIESADVFSLEFENTPVADVDVLTQTKTLHPPRIALATAQNRLSEKALFDELEIPVAPYRAVDSLESLKKAVAELGLPIVLKTATGGYDGKGQFVLRSEDQIDTAWAELGPAKSLVAESFVKFSREVSIIAVRGQNGEVKTWPLAENHHHNGILSHSIVPAPNSEALQPVAQDYITRLLNHLNYVGVLTLELFVTEQGLCANEMAPRVHNSGHWSIEGAVCSQFENHIRAVAGLPLGSTDVVRPTVMINIIGQHPKTEDVLALNGAHLHLYNKSERAGRKLGHITLMPVDSNELTNLCRQLAKILPEPLALTDDMII

ST25 4190 cg69_0762

MTQAQAELVINGSTISSNATVPVTNSDTYTPNNNFQSCLANLRSQAIAAGVSGSTYDRYTQNLTPDYSVIDKLNYQPEFSTPIWDYLSGLVDEERVALGKQKLAQHRDVLNRASQVYGVPAETIVAVWGVESNFGDISGKYPLLQALGTLSCEGRRQSYFRTEFFATMRILQRGDLTEDQLKGSWAGAFGHTQFMPSTYERLAVDFDGDGRRDLVSSTADALASTANFLNKAGWQTGMPWGFEVQIPAGMSIDGEGRRSKKPLSSWSARGVTRIDGSPLIQGPLSGSTPAGLMAPAGPSGPIFLVFKNFDAIYSYNAAESYGLAIAHLSDRLRGAGPFVSSWPTDDPGTSRAERREIQQFLINRGYDIGAVDGLIGDKTRVAIRQEQTRLGLNPTGRAGQQILRAFRQEQARKMMQ

ST25 4190 cg69_0763

MEIKMSYYHILIEVNDHISTIEQTRDIELFDIIELKPYLHSILLPYFNEQEIELEDENIEYKDILHLEVKQTLLPIEHLIEEEQKQLPSDTDVTITAYEIFNDRDLSQDVTSVIFDILEAVKLDQSIV

ST25 4190 cg69_0764

MRLTSLLLGMAFAFSNFQANANLATPLHTQVQQKNVQHLTYKIAEVDPRFGLSQEQLIQITQQAADIWKEGTGKNYFTYDPNAKLEIRLVYDDSQNRSAERQKIASQFKQDQQRVIDEQQQIKQLKQNLSQTQSDLENKKQILNEKLKNFDQQMMQFKEGKLAPEYTAKSLSKTQKDLQKQTVALKKDIAAYNQQAADLNKKVTHFNQINDEFNQSLNQFKQNAQADVFKKGIYNGKQIMIYEYSSIDDLRLTIAHELGHALGLKHSDQPGALMYSVRKR

ST25 4190 cg69_0765

MKKFLWWGISLYFSISLQLAAFAQSNLSNISFNQPLKYRIDYIDPRFELTKEQFIQIGKEAAEIWQKETGKTYFIYDSQAELTINLVLDNQQATKNERKNSINELLKQQEEWREKNKAILLFKQQIDQETSLLNKEKENLNYKFEQYQKDVSLFNQGNYGQFTQSSLNKRQAELSQLSLELQTKFSQHSNKIEMLNRQIKQINQQQNLLNQSIEQFNLSTTSGSKTFHKGLFSQNQIQIYGFTSFDDLRLTLAHELGHALGLKHTDDPKSLMYPLLREQDIHNFKLTNSDLDLLATLYGSNDENH

ST25 4190 cg69_0766

MAKIIGIDLGTTNSCVAVLEGDKVKVIENAEGARTTPSIIAYKDGEILVGQSAKRQAVTNPKNTLFAIKRLIGRRYEDQAVQKDIGLVPYKIIKADNGDAWVEVNDKKLAPQQISAEILKKMKKTAEDYLGETVTEAVITVPAYFNDAQRQATKDAGKIAGLDVKRIINEPTAAALAFGMDKKEGDRKVAVYDLGGGTFDVSIIEIADLDGDQQIEVLSTNGDTFLGGEDFDNALIEYLVEEFKKEQNVNLKNDPLALQRLKEAAEKAKIELSSSNATEINLPYITADATGPKHLVINVTRAKLEGLVADLVARTIEPCKIALKDAGLSTSDISDVILVGGQSRMPLVQQKVQEFFGREPRKDVNPDEAVAIGAAIQGAVLSGDKNDVLLLDVTPLTLGIETMGGVLTPIIEKNTTIPAKKSQVFSTAADNQPAVDISVYQGERKMAQQNKLLGNFQLGDIPPAPRGVPQIEVSFDINADGILKVSAKDKSTGKEQSIQIKANSGLSDAEIEAMIKDAEANAEEDRKFEELAKARNEADALISSSNKAVKDLGDKVTEDEKTAVNTAVSELEAATKENDVEAIKAKTEALQNILMPITQRAYEQAQQAGGAEGFDPNAFQGGDAGQQKADDGVVDAEFTEVKDDKK

ST25 4190 cg69_0767

MANEQNEQAQDIQNEQVEQSNEQTQAEGVEQANDVTVESLQAQITKLEENLKLEKARTANAVYEAQKSVERIQRESEKHKETVLEKFAKELLDSVDNLERAIQAAGDEETPVLEGVKLTLKSLLTTLEKFGVVEADTQNGFNADLHQAVGIDPNAKANEIGTVLQKGYTLNGRLLRPAMVMVGQ

ST25 4190 cg69_0768

MDTLKAIQVFVSIAQHGNLTKAAEHLNYSRAMVSRYLEHLEHTFSTRLFQRNTRKISLTPAGEKALLYCENILQQQQLLQELAAPEQHNGTIRFTCGLFLFELGVAECIRQFKRQHPHIQFDVYLTESTIDLIDAQVDLALRITQKVADGLIARPVCQIESVFCAHPDYLKQHSPLLHPRQLIQHECIAHHSQHQFWTLFDNEQQPQNYPLNVTFKSNDVNALYDMCLHAQGVAMLPTLLVERDLKEKRLKALFKNFTAPELSLSVVYASRQHLPRITQEFIAFMIENLDFYLKPQN

ST25 4190 cg69_0769

MKLFKLSASALATATVLTMAQSAFAQDLKIQSFLAKPEHFGVTSTLIEGDKEVLLVNAQFSKSEALRIAANILDSGKTLKTIFVSYGDPDYYFGLDVFKQYFPNVQIIATPETVKHIQDTQALKVKYWGPQMGANAPSQIIVPQAYTAKTLKLENESIEIKGKKELTYLWVPSAKAVVGGIPVSSGIHLWMADTPKTKDRMEVIQSLESIKALQPKIVVPAHMVEGAPQGLDAVNFSINYLNSYEKATKATKNATELSKLMQKQYPTLQSVDSLELGAKVVKGEMQWP

ST25 4190 cg69_0770

MKKLGLISVVLAGVAFTAVGCASEAGLNASGSAQTSASPSGVQAGVGVDAGVSGQAQPGQVDAGASGSVSGSATAQ

ST25 4190 cg69_0771

MSAKLVVTLLATSLLTVGCVAYTDDPYYRGGYGYHDHDDDRYDRNDGRRYSEWERKRWEERKRLYEQQRKDIREQQKDRREWEKRHREWEKKRLEDRDHDHRDYRHDD

ST25 4190 cg69_0772

MTAFNQKIQTLLDKGQGPAARALDRLPRLAQESLAKILGYSYQYPDLDSFTKCMMAVQIKQGRVGFIGSDPIESRRAFDAQMQAIRQKPTDIELVEDIRLPLQSGTIFARHYHPAPNKKLPLIVFYHGGGFVVGGLDTHDEVCRLLAKYAKVQVLSIDYPLAPEVSPQHLIQSCEDALAWVYQNRRQLKILKSRIAVAGDSAGGNISTVVAQKSVNKAYAPQAQLLIYPTVDFKSRHPSFYAYNEGLVLTNTDIDYVTQYYATHHNVELDDPIISPTYGNLKKNPPAFVITAGHDVLHDEAKIYSHKLRQNGVKMHYEEFPDQTHGFINLTPISRKAKRYTIEISKNFRKFWDKNS

ST25 4190 cg69_0773

MQTNLGNIEIELYDDKAPMSVNNFKGYIKSGFYKETIFHRVIPGFMAQGGGMTANMQEKTTRAPIKNEAGNGIANTRGTLAMARTSNPDSATSQFFINVADNNFLNRSPGNPGYAVFGKVVKGMDVVDRIVQAPTSNYGMHQNVPKQPIKIVDIKIKKSQK

ST25 4190 cg70_0775

MPLLWWLVVISLLFISFIFFLKRPQLAQIAYLDKKLWSLAYHSQNTISRVKILKVIDYQIFIVIYFEGHKTLTSIIWFDQMSLVEWKKLKTLEKLY

ST25 4190 cg70_0776

MSEEMTLEERKVIYRARRGLKEIDVYFDPYVKNYYLKADPAEKALFAELVEQEDPDLLDWFMEVSEPPRTELRDFIYKLKHYVHG

ST25 4190 cg70_0777

MQSGAIRPIPNMLPRHLFNEEHEAFRETVRKFYEKEVVPNIEKYEKQQHVNRDLWNKAGALGLLCTTMPEQYGGSGVDRLYSMILIEEQAYAMDSSTGFSLHSDIVANYINNFGNEEQKQKWLPKMATGETVTAIAMTEPGTGSDLQAVRTTAVLDGDEYVINGSKIFITNGYLCDMAIVVCKTGNSDKGSANLSLIIVEADRAGFTKGKPLNKIGMKGQDTCELFFDNVRVPKENLLGMEGMGFIMLMKELAWERMLVAIICQAGAEAAFAHTVQYTKDRKAFGKPVGAFQNTRFKLAELRTEIDFCRTYLDRCMELQLDEKLSVEAAAAAKYKISDMFSKVVDECLQLHGGYGYMMEYPIARAYIDHRANRIYAGTNEIMKELISRTL

ST25 4190 cg70_0778

MEKQVDVLIIGAGISGIGVAAHLSKNSPQRQFEILERRESFGGTWDLFRYPGIRSDSDMSTFGFNFKPWCKANVLADGASIKGYLGEVIDEFKLKEKIHFGHRVLSANYDSASKKWHVIVEDNKKKQQTWVANFVVGCTGYYNYDQGYAPTFPNQEAFKGQLIHPQHWPENLDYKGKKVVIIGSGATAITLVPAMSKGGAGHVTMLQRSPTYIASVPSIDFIYEKTRRFMSEEAAYKFTRARNIGMQRAIYALSQKHPKTVRRLLLKAIEMQLKGKVDMKHFTPSYNPWDQRLCVVPDGDLFKILREGKASVETDQIEKFTETGIQLKSGKHLDADIIVSATGLQVQIMGGVQATIDGEPVKSSEHMLYNGVMLSDVPNMAMIIGYVNASWTLKVDIAADYICRLLNYMDKNNYDEVIPSGDKSVMEEDTVMGSLSSGYISRAADVIPKQGKQAPWQVTNNYLEDRKSLKNAKFDDGVLRFHKHSEVTTRKPKLVS

ST25 4190 cg70_0779

MRHRNSGVKLGRTSSHRKAMFQNLANSLFEHELIKTTLPKAKELRRVAEPLITLAKNDTVANRRLAFARTRNAATVGKLFTVLGPRYKERNGGYLRVLKAGFRAGDAAPMAYVELVDREVNTSAE

ST25 4190 cg70_0780

MTRTANEFLTPQAIKVEAVSGTSAKVILEPLERGFGHTLGNALRRILLSSLPGAAVVEVEIEGVEHEYSTLEGLQQDIVELLLNLKGLSIKLFDQNEAYLTLEKQGPGDITAADLRLPHNVEVVNPEHLIGTLSATGSLKMRLKVSQGRGYETSDSRFPEGETRPVGRLQLDASYSPIKRVSYTVENARVEQRTDLDKLVIDLETNGTVDPEEAIRKAATILQQQIAIFVDLQKDQTPVAQEPREEVDPILLRPVDDLELTVRSANCLKAENIYYIGDLVQRTEVELLKTPNLGKKSLTEIKDVLASKGLQLGMRLENWPPASLRMDDRFAYRSR

ST25 4190 cg70_0781

MARYIGPKCKLSRREGTDLQLKSGVKPFDVKTKKANKAPGQHGQARGGKQSEYSLQLREKQKVRRIYGVLERQFSNYYKEAARVKGATGENLLKLLESRLDNVVYRMGFGSTRAEARQLVSHRSITLNGRRVNIASIQVKAGDVIAVHEGAKQQLRIKNAIELAAQRGIPAWIEVDHSKLEGTFKAAPDRSDLPAEINESLIVELYSK

ST25 4190 cg70_0782

MAKDTRTRKKVTRTVSEGVAHIHASFNNTIVTITDRQGNALAWATSGGQGFRGSRKSTPFAAQVAAEVAGKAALDYGLKNLDVLVKGPGPGRESAVRALGAVGYKINSITDVTPIPHNGCRPPKKRRV

ST25 4190 cg70_0783

MARIAGVNIPDNKHAVISLTYIFGIGRHTAKNILAAVGITETTKIRELDDAQLDAIRAEVAKVPTEGDLRREISMNIKRLMDLGCYRGLRHRRSLPVRGQRTKTNARTRKGPRKPIKK

ST25 4190 cg70_0784

MKVQASVKKICGSCKVIRRNGVIRVICSAEPRHKQRQG

ST25 4190 cg70_0785

MSPSSSGHVNMMKGQPFHVKYREIIRRMSFLIGALLVFRLGAHIPVPGINNAALENLFHANQGTILGLFNMFSGGALERMSILALGIMPYISASIIVQLMSTVIPSLEALKKEGEQGKRKINQYTRQGTLLLAVVQAVGMCAGLIGQGITLSSGLAFYIPAVTSLVAGTMFLMWLGEQITERGIGNGISMIIFAGIVAGLPKLIMQSVSSVDNGQTSLIGLVIFGLLSLGVLAAIVFIEKAQRRIPVNYAQKQQGRRIFTAQQTHLPLKINMAGVIPAIFASSLLLFPASLGQWVGSADPNAGLIKRSLQDLALVLSPGQPLYLVLFGALIIFFCYFYTALVFSPKEVSENLKRSGAYVPGIRPGEQTARYLDHILNRLTFIGAIYITVICLMPMILQSSFGIPFYLGGTSLLIVVVVVMDFMAQLQAHLTSHQYDNQTLMRKTTAHPKG

ST25 4190 cg70_0786

MTLRLNELAPAEGAKREHRRLGRGIGSGVGKTGGRGIKGQKSRKSGGVRPGFEGGQTAIYRRLPKFGFTSQIALKTAEVRLSELSKVEGDIVSLETLKAANVVRRDQIRARIVLSGEITRAFTVQGVALTKGAKAAIEAAGGKVEE

ST25 4190 cg70_0787

MKTIKVTQTKSSSHRLKNHKLCLQGLGLRRIGHTVEVQDTPSNRGMINKVYYMVSVEE

ST25 4190 cg70_0788

MAKVEQNEGLVEKLVAVDRVAKVVKGGRIFSFTALTVVGDGNGRVGFGRGKAREVPAAISKALEAARRNMITVDLAGTTLQHPVNARHGASRVYMQPASEGTGVIAGGAMRAVLEAAGVHNVLAKCYGSTNAANVVNATFKGLRDMTSPEKVAAKRGKSVEEIQG

ST25 4190 cg70_0789

MNEKKQSRLRRAKSTRLHIRALGATRLCVNRTPRHIYAQVISADGGKVLAQASTLDASLRSGTTGNIEAATKVGALIAERAKAAGVTKVAFDRSGFKYHGRIKALADAAREGGLEF

ST25 4190 cg70_0790

MSRVAKAPVTVPNGVTVTQNGRQVEVKGSKGTLSFNLHALVELKQEEGKLQLAPAKESKDAWMQAGTARAVLNNLVKGVSEGFERKLQLVGVGYKAAVKGTVVNLNLGYSHPIDYALPEGVTAETPTATEIILKSANKQLLGQVAAEIRAYRSPEPYKGKGVRYSDEVILRKEAKKK

ST25 4190 cg70_0791

MVKRVSRPGLRQYRGKDKLPSVKQGLGIAIVSTSKGIMTDRAARAAGIGGEVIAFVS

ST25 4190 cg70_0792

MSMQDTVADMLTRVRNAQMAKKQTVSMPSSKLKVAIANVLQQEGYISNVEVAQEETKSTLTITLKYFVRQASYRNG

ST25 4190 cg70_0793

MAKKGMINRELKREKTVAKYAAKRAELKATIANVNASDEERFEAMLKLQALPRNASPVRLRNRCGLTGRPHGYFRKFGLSRNKLRDTVMQGDVPGVVKASW

ST25 4190 cg70_0794

MARLKARYNDELKAKLQEELSIKNVMQIPRITKITLNMGVGAAATDKKLLDGAVADMQLIAGQKPVVTLARKSIAGFKIRDGWPIGCKVTLRGDQMYEFLDRLISIAIPRIRDFRGFSAKSFDGRGNYSMGLKEQIVFPEIDFDKIDRIRGMDITITTTARTDDEGRALMRAFGFPFK

ST25 4190 cg70_0795

MAKIKKGDQVIVIAGKEKGKQGTVLSVSEDRVKVEGLNLVKKHQKPNRVTGAEGGIVTQEASLHISNVAILNATTQKADRVGYQVIDGVKTRVYKSTGESVAVAK

ST25 4190 cg70_0796

MIQTETMLDVADNSGARRVQCIKVLGGSHRRYASVGDIIKVTVKEAIPRARVKKGDVMNAVVVRTKFGIRRPDGSVIRFDDNAAVILNNNKAPIATRIFGPVTRELRTEQFMKIISLAPEVL

ST25 4190 cg70_0797

LYGKSIRRSTKLHAHDENNVAKIGDVVTIKESRPISKTKAWTLVEVVEAAAE

ST25 4190 cg70_0798

MKTKDLREKSVEELKALLDEQQLNQFRLRMAKATGQLGKSHEVQVARKTIARIKTLLTEKQGNGQ

ST25 4190 cg70_0799

MLQPKRTKFRKVHKGRNTGLAHRGSTVSFGSIAIKATERGRMTARQIEAARRTISRRIKRGGKIFIRVFPDKPITEKPLEVRMGNGKGNVEYWVCEIKPGKILYEIEGVNEDLAREAFALAAAKLPFKTTIVTRTVM

ST25 4190 cg70_0800

MGQKVHPIGIRLGVVKRHNANWYANPKQYAEYLLKDLQVREFLTKKLKNAMVSNILIERPSGAAKVTISTARPGIVIGKKGEDIEKLQRELTNIMGVPAQVSINEIDRPDLDARLVAEAIASQLEKRVMFRRAMKRAVQNTMRAGAKGIKVEVSGRLGGAEIARTEWYREGRVPLHTLRADIDYATMRAETTYGTIGVKVWIFRGEILGGMKQVMNPAPAEERPAKRGRGRGEGQERRGRRGDRAADKGE

ST25 4190 cg70_0801

MEVTAKLRGAAISAQKARLVADLIRGKSVAHALNILNFSNKKAAVLVKKALESAIANAEHNNSLDVDDLKVSTIYVDEGMSLKRIMPRAKGRADRITKRTCHITVKVGV

ST25 4190 cg70_0802

MILPDFVGLTISVHNGRNHVPVIVTEHMVGHKLGEFAPTRTYRGHGVDKKSKR

ST25 4190 cg70_0803

MPIQKCKPTSPGRRFVEKVVHDHLHKGAPYAPLVEAKKRTGGRNNNGHITTRHVGGGHKQHYRIVDFKRNKDGVPAVVERIEYDPNRTAHIALLKYADGERRYIIAPKGLRAGDKVQSGNDAPIRPGNCLPLRNMPIGSTLHNVELKIGKGAQLARSAGASVQLLGRDGSYAIIRLRSGEMRKVHVECRAVIGEVSNQENNLRSLGKAGAARWRGVRPTVRGMAMNPIDHPHGGGEGRNKGIQPVSPWGQKAKGYKTRTNKRTTKMIIRDRRVK

ST25 4190 cg70_0804

MNNERIYQVLKGPVFSEKAQVLGDTAGVQVFKVDINATKLEIKKAVEKLFGVEVVKVNTTITKGKTKRFGRTLGRRSDVKKAYVTLKAGQDVEMADLGDTAESAAE

ST25 4190 cg70_0805

VNLKTVSGSAVELSEVAFGREFNEALVHQVVTAYLAGGRQGTRAHKSRADVSGGGKKPFRQKGTGRARAGSIRSPIWVGGGKTFAARPQDWSQKVNRKMYRGAMQCILAELVRQDRLVLVEEFAVAAPKTKELLAKLNDLNAARALIVTDAVDENLYLAARNLPHVDVVDATAIDPVSLIAFDKVVMSVAAAKKIEVELG

ST25 4190 cg70_0806

MAIGLVGRKCGMTRIFTDAGVSVPVTVIEVDPNRITQIKTLETDGYQAVQVTTGERRESRVTNAQKGHFAKAGVAAGRLVKEFRVTEAELEGREVGGTIGVDLFTVGQIVDVTGQSKGKGFQGGVKRWNFRTQDATHGNSVSHRVLGSTGQNQTPGRVFKGKKMAGHLGDERVTVQGLEIVSVDTERSVLVVKGAIPGATGGDVIVRPTIKA

ST25 4190 cg70_0807

VILYIFGLEFTGMSNQRIRIRLKSFDHRLIDQSAQEIVETAKRTGAQVCGPIPMPTRIERFNVLTSPHVNKDARDQYEIRTYKRLIDIVQPTDKTVDALMKLDLAAGVDVQIALG

ST25 4190 cg70_0808

MKSHLRVHYFQHIAGEGFGSCYSFLKAHHATITATEFFALPVDLPLEIEALPHIEDVDLLLIMGGTMSVNDEANYPWLKIEKRWIRRYLAAGKPAIGLCLGGQLIANALGAAVSRNRYQELGWSTVQRVPNLPKESFLLPEKINVMQWHSETFEIPKGAIHLAENSVCRNQMYQIGSNVLGFQFHPEMTPKVLNLLLENEQELSIFKGEYVQSLDELHHCDIQKFEQGNQLLNRAIEFVVNQ

ST25 4190 cg70_0809

MKKHNNFQTQLIHAPRKAPQFIETVQPPLFRASTIIFKSTSHLFDRHWTDPYDYSYGTHGTPTTFTLGDNIAQIEGGLYCLLAPSGLSAINLVNSAVLATGDEVWVPDNIYGPNLEHLNYLKDQYGINVQVYNPIDVSSFQPSDKTKLLWLEAAGSVTLEFPDLKALVKKAKAHGVLTALDNTWGAGLAFNAFDFSDEHLSVDLTIHALTKYPSGGGDILMGSVVTRDEKLHHRLFRMHAILGISVSGDDTAQIQRSLAHMSLRYEQQSNNTKTLLTWLKEQPQFAQVLHPSDKAAPGHQYWQEICSTGRSAGLVSVVFKSDYDISAVRRFCDALKLFKLGFSWGGPVSLVMLYDLKMMRKLENTHLQQGLLVRFCIGLEDPQDLIQDIENALKQM

ST25 4190 cg70_0810

MGTPIVIAKNINDTSKEIVLLSQFANRHGLIAGATGTGKTVTLKVLAESFSRIGVPVFLADAKGDVSSLAKAGEASDKFNERLKLLQIDSVPFAANPVVFWDLFGQQGHPIRTTISEIGPILLARILNLNDTQEGVLSAVFRIADDQGLLLVDFKDLKAMITFVSEHAAEFKAEYGNLSPASLGAIQRNLLALADQGGEQFFGEPALNILDFIQTDANGHGYINILAADKLMNTPKLYATFLLWMLSELFEQLPEIGDMDKPKLVFFFDEAHLLFDNASQALQEKIEQVVRLIRSKGVGIYFITQNPLDLPESVLGQLGNRVQHALRAFTPKDQKAVKTAADTFRANPNFKVDEAITELGVGEALISCLDEQGTPQIVERGWVMPPYSSFTPITPEERQTLISQSIIAGVYEQAVDRDSAYEMLQNKVAEREQQKQDAELAKQQAKEQETLAKQQAKEQERLAREQQKEEERAARERAKLTQDIVGTFAKSAARSLGGSTGQKISTGATRLIIWKIKFIFKSVY

ST25 4190 cg70_0811

MTPQQIELVKSTVPVLREHGVTLTTYFYKRMLNNNPELKNVFNLDDQTSLRQPRALAAAVLAYAENIENPTVLAKAVERITTKHVSLDIQPDQYAIVGDNLLHSISEVLNVPFESELIEAWKQAYLQLADILIGVEKQKYEQLESLKGGWAGWRSFEITQIDPLESGKRFTLKATDHEDVLTSPANAFISVKVQVPNQQLEQPKAFKFTEAQEDNTYHFDVQPEEDHTEFSVSNILLEHYRVGDQVQVSAPLTL

ST25 4190 cg70_0812

MQLNKFTDYALRILMYVARPSDAPYTIAEIAQDLYVSQNHLVKVVHFMGKQEWIITIRGKGGGIRLNPKALDLKLGTIVRILQGDHQIVECNTPPCVLRSHCGLKGILDQALESFYQSLDQYTLGEVLQQPKSASDHSPIAFLQL

ST25 4190 cg70_0813

MRRSEKSKDTKARLAPKQKISYEKKPDPAITAYAVQSLNWLRQAEFLMSAPKLALCVEDSGYEIAFAGRSNAGKSSAINALTNQKQLARASKKPGRTQMINFFSLGNPDQRLVDLPGYGYAAVPEDMKRVWQKELENYLIHRKSLQGLVLLMDIRHPLQHFDMMMLEWAYSRHLFVHILLTKADKLNRGPANKVLLEVKQQLKKMKLDFSIQLFSSLNKLGLEELASVMAGRLHFTLEQQPEFDVDAIPEASDEDAEE

ST25 4190 cg70_0814

MMKLLSFLKNKALGSSIDYKILPRKVKFDWKDTPVDWIPNQPFASYFINEINNILPAGEFWFCRLYNKVLPRITDEKLKQDVQAFIRQEAMHANAHTSANKEYLSARNIDIQRNLDIMNYLFTTALADKPFDKEVPQFLQEQWDLFRLGVIATVEHMTCVLGKYALYNKRWEELGADPEMVDLVKWHGSEEIEHRTVAFDLYRHLGGGYIPRYYLSLAVIVLVLGLWVDGAAHIMKQDPRFADAAKSRFFPAWVALEWYKISRKDNQVLPNPIWLIAQQIDYLMPWYDPVKEGSTEDAVSYLSQSPAAKRAELQAA

ST25 4190 cg70_0815

LPTEKNNVDMNLNTQILIAAILGLAFGFLLTMYPDTAFVKHSLYGLGILSSVFIGLLKMLLVPLIFSSIVVGVSNLQAGGQLGPVWKITFLCCITTTTLALILGISCAHLFEVGRGVDISIFQASMQSYQTPDSLNPATFFTNFIQNTLINPFKAFSDGNVLAVVVFALFVGVALVHGGERFKSIRVLSHQFFEMMMMLVGWVMKLAPLGIFALLAKLMATEDISVLSRLAEFAAVVTGTTIFHGAVVLPLLLWIFGCMSPWTFFKGTRTALITAFATSSSSATMPLSMKCAQENLGVRPQTAGFVIPLGTQLNMDGTALYEAAAALFVANLVGLDLNLTQQIVVCLTAMIASLGAPGIPSAGMVTMIMVLQSVGLPAEAIAILLPIDRLLDTVRTVVNVQGDMMISVVVDRYTKDKDISI

ST25 4190 cg70_0816

MNALTQELVELLTLEKLEENIYRGISRNLVGKRVFGGQVLGQALRAASYTTDRPAHSLHAYFLYGGDINAPIIYEVDRLRDGKSFVSRQVRAIQHGRVIFSAMVSFANPEEGLNYQHPEPDYPAPEALKSESELKEGILNFVPENVRASFMRERHVEIRPIDPVNPFQPQPEAPFNAHYIRTHDRIPKQLDDISLHQAIVAFYSDFTLMTTALRPHGLSYISPSLQCASIDHAIYFHRPLRADEWMLYDMEATVSAASRGLNFGRMWQNGQLVCSTVQEGLMRLREIETQ

ST25 4190 cg70_0817

VVTPHVLGEVPTEPVSTTETEAKTERQKVVCYVLQNHSRSNALVVDGETRRLNLKPALDPLVIGTHQEKASVLFLQHNDENNLLNPPPHAFPPRLIKLIEVLQANPELDIELVPVTVLWGRSPDKEDSWFKLLFSDTWATPSTVKQLVNIGLHGRQSYLEFHEPQSLRDLVEYAQKHYPNLSPATYIVSTLNDYLDRQREVVLGPDLSDRRNVMQSVLKSRDVQEAIRRESIRGKISMLEAERRAIGYVNEIVSDYSHSAVRFADLALTRLWTQLYDGVEVHNFSTVRELAKDYEIVYTPCHRSHIDYLLLSYVIYKRGLMVPYIAAGDNLNLPFVGQLLRGGGAFFIRRSFRGNGLYTSVFKEYLYSILSRNTPLEYFIEGGRSRTGRLLPPKTGMLAMTVHSHLRGRAKPIVFVPTYIGYERLMEGSTYVGEMQGKPKEAESIFGILKTLRKIERIFGKVHVNFGEPVFLDDLLKQHNAENVYIEKNDDPVPPAVSEAVNSSANAILENINRAVVINPVSLLSIILLATPKHTLDEEICIKQLEAYRNLASNFPYDQRTEVTPLSGKEIIAYGLKLKLIKRVQHALGDIIAIEDNQAVLLTYFRNNILHAFVLPSLIASLVEHNGKISRADLSNVIYTLYPFLKAELFLKWKSSELKEQIEQYADALVQSNLIQQDDEGNLISSAPNTEEHNQLVVLATPVKQSLERYYMTLALITQRGSGNISAKQVEELSHLLGQRLSVLYEFNSPEFFDKALFQSFIKVLTQQNYIRTNDQGQIEFDDNFRQMAAGAQLVLDEVTLQMLQHITTFTDEELKEALEAMASQQAKRRLKRKKA

ST25 4190 cg70_0818

VWCAYDPAGTQGDITRRLNDIRLYAQQYQVKFKVVSYQKEQQAIQAFDEGKCSGLAASNFNTYQYNHFMGSTSGIGLIPNNRTARNLLQLLNHPTIEKRLINKDYEAVGMIPVGTANMVMKTKKISKVAQLRGQRIGVLANNPPQQALVRSVGAQPVYVDLSNAIAQFQQNKIDIMPAPVYGLLPYNLQKDFGPDTQVINFPLAYFGVNIIIKPQAYPANFGRKIRAWFVQNSQLLTNRAIQWENHLPAYYWVDVSFYEKQSYDIMVAKIRNQYVRSGYYDAYFVELMKRLRCIDDPRYFECPMSR

ST25 4190 cg70_0819

MHILFIGYGKTSQRVAKQLFEKGNQITTISRSEKTDPYGTHLVQDIFTLDLSEIAPVDVVYILLSPDDSTVEGYQHTYVDSIEPIRQALKSHPVKRLIVVSSTRVYGENSGETIDDHSEIHPNDAQGHILHNMELLWQKYFPSQCVIVRPTGIYGASIDRLKRMAEHTQTYPNIHYSNRIHIDDLARFLAFLADFEKPHKSYLVANNAPVPLHEVLLWFQSQLDLPLLTLDSAHVSGKKIYAKHLFETGFQLEHPICFNDYLLCLNAHGTN

ST25 4190 cg70_0820

MTSVHQLQGVGSASAALLEKLNIFTTDDLLFHLPRDYEDRSTIIPMNQLVVGRSYLLEGEVKSVDFPPGKRKSMAALIQDEFGKVTLRFYHIYKNLTDKINLVTVYVFW

ST25 4190 cg70_0821

LKHHSDALPELLPKQYTNGYALKEALHYIHEPPVDANMIQLAQGSHPAQQRLIFEELVAHQISLLTRRAYIRQIASPAFPSSKVLAKKLLEALPFQMTNAQKRVSKEILSDLKQHQPMLRLVQGDVGAGKTLVAAVAACHALEADWQVALMAPTEILAEQHYLNFKRWFEPLGIKVAWLSGKQKGKARAHAEQQIKEGHAELIVGTHALFQDNVEFAKLGLVIIDEQHRFGVDQRLALRNKGAEQLTPHQLVMTATPIPRTLAMSAYGDLDTSIIDELPPGRTPIQTVTIPLDRREEVLHRIASNCREGKQAYWVCTLVEQSETLDAQAAEATYQEMKERFPDLNIGLVHGKMKADEKQAVMQAFKNNELQLLIATTVIEVGVDVPNASIMVIENAERLGLSQLHQLRGRVGRGAKASFCVLLYKPPLSQNGQERLSILRESNDGFVIAEKDLELRGPGELLGTKQTGDMGFRVARLERDDHLLSQAHYVAQQVLKDYPEQADALLKRWLPEAPRYAYV

ST25 4190 cg70_0822

MFKFLNPHYLFQLLSPCLLCEIGTREKYSLCKECWEQLPWLKQTIQRNDQSVLVGCHYAYPINRIIQQFKYEQKLHYQTLLAEVLQQLKFPKVQAIVPMPISKQRLTERGFNQSLLLANLLSKQLKIPVWQPVQRLNEHSQKGLSRLERFENIEQQFVALTQEKRRYRRVLIIDDVITTGSSIHALSQALKQLGCTSIHASCLAAASSTSY

ST25 4190 cg70_0823

VKTITVAAAVILNEQNELLLVRKRNTQAFMQVGGKLEPNEAPESAIQREILEEIGSPCVIEQFIGRFETAAANEPDHKLISHLYLVRLKQSPQIAAEIAEMKWVKFNDSETKLAPLTKEIVIPWCEQNLSITL

ST25 4190 cg70_0824

MNSICIFCGSSLGSNPIFQQIAQLTGEAIAKQGKTLVYGGGRSGLMGVVADSALQAGGQVIGVIPRALVDRELAHPGLTKLYVVENMHERKTKMADLSDGFIALPGGAGTLEEIFEQWTWAQLGIHQKPCAFLNVAGFYEDLLKMIQGTVDNGFSQARFVDKLIASDKIEDILQQFEQYQAPAPKWTNADVQP

ST25 4190 cg70_0825

MLEAFYATERGSLEDATINGGFDLHPELVWLDLIAPSQEEQQWVLDAYNQNLPTLKSLEDISSSARFYRDDDGILHISTYFLTKNKNYQVDGDAEDSSHMLAMVQTVAFILHKDRLFTLRGEKLVAFRAFRARARRNDYEMDYKDPTWILLGLLEAKLDELADILEDIHKDLEKYSTEVLNNHQREQILDLDDMITRLAQQEDMLGKAQLCLIDLRRVLTFLSRPRALGSHIYDADIRELSEDVRSLVEHDAFLFQKVRFLLDTTSGFINTEQNDTIRRFSILPSMLAPPMLIASIYGMNTDVLPFAHGTTSFIIVLLIIIGFFIGPIIYFRWKKMDLKRLLPQILYIKKHLNLGAFLSLLIKPNVGLLANGRLE

ST25 4190 cg70_0826

VVQYLNQELVVSGKIDFENAEQQYQAGLAIIKKQTSFPLIVDLKQLEHGNTLALAVLVQWLRQTPQKSGLHFKNVPEKMLKIIQACHLQEDLHLV

ST25 4190 cg70_0827

VNTLFKQTLTASILSTMIAGTAFAAPSEAPPDFIKRVADGLISRLKADHAKLQNNPALVKTIVRQNLDPYVDSQAFTRIVMGTYATNQYSTAAQRAQFETNFRNTLIENYGSAFAKYTNQTYTMRPYKATAGKNPVVTLDFNHNGEKIPVSFQLADKGSQWKIRNINVSGIDLGLQFRNQFAATVKRNGGDLNKAIATFQPDADAAVNQNKQK

ST25 4190 cg70_0828

MKSRTSELAVGIFVIIFGIALFFLAMKVSGLVGTNLSDGYTMKAQFDNVNGLKPRAKVTMSGVTIGRVDSITLDPVTRLATVTFDLDGKLTSFNAEQLKEVQKNALDELRYSSDYTQATPAQQKTMEQQLISNMNSITSIDEDAYIMVATNGLLGEKYLKIVPGGGLNYLKRGDTISNTQGTMDLEDLISKFITGGGAGKVAAGSSSAEEKAPASTDSSAQPSFVE

ST25 4190 cg70_0829

MNTIAWLGRLVIERIRGIGVAALMLLQIIFSLPSAGGFGRFVYQMHRVGVMSLLIITVSGLFIGLVLGLQGYSILVNVGSESMLGTMVSLTLLRELAPVVAALLFAGRAGSALTAEIGSMKQSEQLASMEMIGVDPLKQIVSPRLWAGIVSLPMLTVIFAAIGIVGGKLVGVDFLGVDEGSFWSGMQNNVQFGHDVVNGIIKSIVFALLCTWIAVFQGYACDPTPEGIATAMTRTVVYSSLCVLGFDFVLTAVMFGGI

ST25 4190 cg70_0830

MIAIMNNKTPLSTQSLIEVKNLSFNRGERVIYDNISLNIRRGQITAIMGPSGTGKTTLLRLIGGQLVPDQGEVLLDGKDIAQMSRQELFAARARMGMLFQSGALFTDMSVYENVAFPIRAHTKLSENLIAELVALKLESVGLRGTEQLMPTELSGGMNRRVALARAIALDPDLIMYDEPFAGQDPIVKGVLTRLIRSLREALDLTTIIVSHDVPETLSIADYIYVVAEGKIQGEGTPEELQAYASPFVKQFLTGSVEGPVEYQFSHQAYLDNEVRP

ST25 4190 cg71_0831

MLSLTPCAGRCSTVFGDQVCRGCRRFNHEVIQWNTYTAEQRLAVWKRLDDQLDQILVPMLPQANLQHVEGFILGKRVRLMEGASKGRKLYHALKICEKNKHLAHESGLGISEAQVKEVWTEFERRILALAKASYELAWLRADGISHHLLNMFEEE

ST25 4190 cg71_0832

MNATVEQLAPVEQQATAGWVVAALYQFKEVQDPADLQQRLLDLVKTINLCGTLIVAGEGINGTVAGDREAIDTIHQFLLNEGFNAMEYKESHSSDKPFRKMKIKLKKEIVTLGVEVKPRDLVGHYLDPKEWNELIARDDVILIDTRNDYEYKAGTFKGAIDPKTETFREFPKYVKKELEQHKDKKIAMFCTGGIRCEKSTSLLLQEGFKEVYHLKGGILKYLEETPPDESLWEGECFVFDGRTAVTHGVEEGANIKCHACGWPLTPEESALPSYEHGVSCLYCIDKTTEKQKAGFRMRQSQIAAAKRKRL

ST25 4190 cg71_0833

MSALDLDQLSEYLDGDHNEYGLDFAATHGFLCAIAVGPQFDRWLDELFEGNQKKVPAEIIQQIKVWLESIRQDLANENGIEFPFEVEEADVESSLGDWSVGFVDAMFLNEEAWFAPEYEEQLVDLTLPIMVFSGIDEEDPQMESFRRNGQLMDELAEEIPDNLNELYLMYHTPN

ST25 4190 cg71_0834

VSSTDFELDDNYGDDDDVGFDESSGKISAKESLEKRRLIDDLLAQRRLERELKDFDYDLDDDDLDDED

ST25 4190 cg71_0835

MSDMHSPTSQVAALISRGKEQGYLTYAEVNDHLPDSITESEQIEDIIQMLQDVGIPVHERAPESDDTMFGESADATDEVAEEEAAAVLASVENEPGRTTDPVRMYMREMGTVELLTREGEISIAKRIEEGIRDVLNSIAYWPNAVEVVLKEYNDFLTGERRLADILSGYLDPETDEDIPEVLEDVEELEEEDESSTKSTKEVKLDDDDEEEESEGDDDSEGDSGPDPEVAKVRFAELEAAWAQTKAVIEKHGRNSPEANEALESLATVFMMFKFTPRLFDIISEMIRGTHEQIRANEREIMRYAVRRGRMDRNQFRTSFPKQESNPAWLDEQIAKAPAEIKAHLEKVRPDVLAFQQKIADIEKELGLNVSEIKDISKRMAIGEAKARRAKKEMVEANLRLVISIAKKYTNRGLQFLDLIQEGNIGLMKAVDKFEYRRGYKFSTYATWWIRQAITRSIADQARTIRIPVHMIETINKINRVSRQLLQEMGREPTPEELGERLEMDEVKVRKVLKIAKEPISMETPIGDDEDSHLGDFIEDQNITSPIDAATSEGLKEATREVLENLTEREAKVLKMRFGIDMPTDHTLEEVGKQFDVTRERIRQIEAKALRKLRHPSRSEHLRSFLEND

ST25 4190 cg71_0836

MLDRTPSRELREDLWVFPMDYPIKLIGDAGEELRIAVVDILVKHFPDFDQTTLKVQESRTGKYHSLTAQLRFDELEQVHALYADLAACPQIRTAL

ST25 4190 cg71_0837

MSLDKPTLIIRQWNDLQDYQSKFESMKNLTNQRDENTADELWLLQHHEVLTQGQAGKPEHILIPSNIPVVQTDRGGQVTWHGPGQLVAYFMFDLNRLKWNVRTLVSFAEQFMIDVLKKYNIEAYAKPDAPGVYVDGRKIGSLGFKIRRGRSYHGLALNLDCALTGFQTINPCGYAGLEMVRIQDLVTPYPSFEQLCQDFIEYIKATGYFNDPEVKIE

ST25 4190 cg71_0838

VISTKAVKPTLQLAYVKLMMDVVGRGLVMASQVDSEIQEEVSKFPVHFVLSMNVFPNGPAFFARVTEDKQLELLKRAEKKPDLTITFKHVTHAFLVFSFQESTAQAFANDRMIADGDVSAAIRLVRCLNKMESLILPKLVASLAVKEYPTELTLKEKFNQAANIYLKVAKSYFKRSA

ST25 4190 cg72_0839

MGQDHIEQHRRYIVISYAFMFLALFTVIFAAFAYLVARKVAVVDDAEVWIHAHALWIMRNGILFLLMSVFAVVWFIPLFFFAWDSNLWVTASTVAGVVFSAIAWLFLLNAWLKGLSKYLKNKAVF

ST25 4190 cg72_0840

VSFFSLGFRTGLVVAFSIPLVLAMTFAGMNLFDVGLHKISLGALILALGLLVDDAIIAVEMMAIKMEQGYSRIKAAGFAWKTTAFPMLTGTLITAAGFLPIATAQSSTGEYTRSIFQVVTIALLVSWVAAVLFVPYLGEKLLPDFTKMGHQAPWYVRLWARITKKPQLQTVAISQDHHYDPYQSSFYLRFRKMVEFCVTYRKTVIVTTVGIFVLSVLMFKMVPQQFFPPSNRAEILVDLKLEEGASLTATEQAVKKVEQFLSKQKGIDNYVAYVGTGSPRFYLPLDQQLPQASFAQFVVLASSLDDRDEIRRSLDTQIKQLLPQVRTRVSLLENGPPVGYPLQYRVSGEDLNLVRKEAQQVARVISENPNTTNVHLDWGEPSKIISIQIDQDRARQMGVSSLDLANFLNASITGSAIEQYREKRELIEIRLRGDKAERVEVASLASLAVPTANGTTVPLAQIAKIEYKFEDGLIWHRNRLPTITVRADIRTNLQPATVVGELAESMDKLRAELPSGYLIEVGGTVEESARGQSSVNAGMPLFLAVVMTLLMIQLKSLSRATIVFLTAPLGLIGVVLFLLLFNKPFGFVAMLGTIALSGMIMRNSLILIDQIEQDRQAGHPTWEAIIDATVRRFRPIILTALAAVLAMIPLSRSIFFGPMAVAIMGGLIVATLLTLFFLPALYAAWFKVKKTA

ST25 4190 cg72_0841

MKFNLSEWALNNKGIVLYFMLLLGIIGAISYSKLSQSEDPPFTFKVMVVQTYWPGATAKEVSTLVTDRIEKELMTTGQYDKIMAYSRPGESMVTFVAKDSLTSAQIPDVWYNVRKKVNDIRHELPSGVQGPFFNDEFGDTFGNIYVLTGKDFDYALLKEYADRLQLQLQRVKDVGKVELIGLQDQKIWIEISNTKAVQLGIPVSAIQEALQKQNSMASAGFFETGTDRIQIRVSGQLQSVDDIKKMPLLVGDKTIQLGDVADVYRGFSQPAQPRMRFMGDNGIGIAVSMRKGGDIIALGKNLETEFAQLQKTLPLGMKLQKYLTNQ

ST25 4190 cg72_0842

MVTQPSTTLHEQKSYAGDVQARQQTALAFRVGGQVTARYVDVGDRVRVGQVLAKLDVADAQLQLNAAKAQLENAQAAAKTASDELKRFQQLLPINAVSRSQYDTVKNQYDAAQAALQQARSNYEVSANQTGYNQLVSNKNGVITARNIEIGQVVAAGQAAYQLAIDGEREVVIGVPEQAVSEIKVGQAAWITLWSKPNERFAGYVREVSPAADQSRTFTVKVALKEGQSAIQLGQSARVFFSSTQTNVMSVPLSSVSATDNQPYVWVVNANQTLRKVPVTIGAYGRDSVPVLSGLTPNDWVVIGGVHLLRDKQKIHPIDRENRAVKIQGAK

ST25 4190 cg72_0843

VQTVNQSGRPKDLEKRARILQAAKAIFLKSGYHGTSMNLIAQEAGVTKLTVYNHFQDKANLFICAITETCEETLGTKPFELDASADFYQTLYIVCSRALQIIYSPEALKLEHVLFELAAEQSPLALQFFNASHTRLQNQLVEFFQKAAQLGFIQADDPLYQTELLLTLLLGVRHHKVLLRIIPVPNAQEIDRFIRDAIDLFLLRYRH

ST25 4190 cg72_0844

MVQQIDAPLREDVRLLGNLLGETLKQHAGQELFNQIEQIRALAKGARDGQAEAEKQLEQLFLELPDEELLPLTRAFSHFLNFANIAEQYHVVRSRRQAEFDPDANSPNPLVHLFKKFKDKNISTEKLFQQICDLKIELVLTAHPTEVSRRTLIQKYDDINACLSQLDQQKLTPRERQNALANLKQQISSAWQTDEIRQHRPTPVDEAKWGFATIEQTLWNAVPKFIRELNELVQDNCQQNLPLHIAPVRFASWMGGDRDGNPNVTHQITQEVLWLSRWQAADLYLRDIENLRWELSIQSCSEEMVQAIGSPHPEPYREYLRATRERLKATRHWLAQRLQGLEADDSNVIKSKDELLQPLLLCYRSLIDSNLPEIANGQLLDFIYRVNCFGIELLKLDIRQESGRHRQAISAITEYLGLGNFESWTEQARQNFLIQELQSKRPLLPKYINEPEGSLIGHPDVQEVFATMRTLADQPPESLGAYIISMAEYPSDVLAVLLLQKEAGIQHPLRVVPLFETLKDLDGAATTMNTLFNMHWYKQHIQGKHEVMIGYSDSAKDAGFMSANWAQYRAQEELTAIARKHGVQLTLFHGRGGSISRGGAPTQQALFSQPPGSISGAIRVTEQGEMIRFKFGLEGIAMQNLEIYTAATLEATLLPPPEPKAEWRELMNRMTDHSVKVYRQTVRENPHFVKYLRTVTPELELQMLPLGSRPAKRKVSGGIESLRAIPWVFAWTQIRLMLPAWLGTGAAINEVIADQQKATLDEMLQQWPYFQTLIDMLEMVLSKADANIALYYESHLTEDEDLKVLGNQLRQRLKDAVETLLTLKDESKLLSDNEVLDQSMQVRKPYLLPLHLLQAELMKRRRDYLAERQAEHTPVDHALMVSIAGIAAGLRNTG

ST25 4190 cg72_0845

MSNWFPKWQPYQGDVDHRPVSTNEYLPPVQSAILGIQHAFAMFGATVLAPLLMGFNPNLAILMSGICTILFFLITGGRVPSYLGSSFAFIGVVAAATGHITGSGANPNLSIALGGIVACGIFYALIGFIVMLTGTRWIEKLMPPVVTGAIVMIIGLNLAPVTIKGVAGQPFEMWMALITVLCMGSIAVFTRGLLQRLLLLVGLILAYVIYAIATNGLGLGKPIDFSQISQASWFGIPNFSHPTFDTKAILIIAPVALILVAENLGHIKAVGAMTGENLTPQLGKAFVADGLATTLSGSVGAPGMTTYGENIGVMAVTRVYSTIVFVIAGIFAIFLGLSPKFGAVISTIPSAVLTGASIVVFGLITIAGAKIWIENKVDFSNNKNLIVASVTIILGAGNFELLFGNFNLGGIGTATFAAIILNWLFSLKDKT

ST25 4190 cg72_0846

MRFDFFDLQLVLHIVSTGSLTKGADRSAISLQAASERIKKLEQYFETPLFIRQTTGVELTTAGHAFVEHARQLLAQKDQLEHEMQRFRQPTTEALTLWCNSSAQSEYLPTLLPQYLVLHPEMNIDLHEAESSEIVDALTQGIASLGLVSSFFNTRHLQTKEFASDPLVLICPVTHELAYYKELNLVDALSYGFIGLKSHHSLQQSIETQAKLLGFNIQYRLRLPNFGAIAEVVAKGVGIAIMPARAAQRLQSDYDFHSIQLLGAWANRKLLLATQSFNQLPSSYQQFADFLLQHRP

ST25 4190 cg72_0847

VTFLAIIIVVFAFAGMIKGMIGLGLPAVSMGLLTIAMSPFQAASLLIVPSMVTNVWQLFAEGHVWAFIRRFWTLLVGIIVGSIWSFLPTLSQSHGHSSEILLGCMLALYGLYGLCVKRLPHLGKHEGWLSPIIGYIGGAVTVATGVIIIPIVPYLQSLHLKRDELVQALGLTFTVSTICLAVFLHHNPMSGISLDYRLSFAALLAALIGMWIGKKIRYRLNEQIFRRIFFIGLMSLGLYMILH

ST25 4190 cg72_0848

MSLLRRWFDPIRSSWFYQKPSRQAVLPTENGLSIYLRLDDVYSYLAVQQLSQLDEILSDELKPLKIIISHTASEPPNSMTHEEWQHYCLNDAKILANQHRFAFDEFPEIPSPESLKQAAVILKRTPLQGQNFLHLLEDIFHMLWQQQYGKLRTLHAMAVKHQVPQHFPERIFTDEPVRAAYFEFGGRKYHAVDDLLRLTRRLKQQKLLTGNPIFLINHIEWREHLINDAEALNEIQALHPELDVFIALEDPMSWLLLAYIKEELADYYDIQLKVYPLSYRGRDWFDWSLATRVSKRTEVAFTPFCRPTEESTLGMAKLFYSVPENQQLDTIFTILQAVWTKGKDLSFQKHFQQLQQQLGIEQLTEQDIQSVLAQNDALCQDKHQPDLPVLELRIDGQSYVFNSLYRVWMIESIFSNVLEEKYKTASTSN

ST25 4190 cg72_0849

VEVISLKHNLLETQMPDYRSKTSTHGRNMAGARGLWRATGMKDEDFGKPIIAVVNSFTQFVPGHVHLKDLGQLVAAEIQAAGGVAKEFNTIAVDDGIAMGHDGMLYSLPSRDLIADSVEYMVNAHCADAMVCISNCDKITPGMLMAAMRLNIPVVFVSGGPMEAGKVKFRGDEKAIDLVDAMVVAADDSYTDEEVAEFERSACPTCGSCSGMFTANSMNCLTEALGLSLPGNGSIVATHANRKKLFLKAGQLIVELAKRYYEQNDASILPRSIATKAAFKNAMTLDIAMGGSTNTVLHLLAAANEAEVDFTMDDIDELSRRVPVLSKVAPAKQDVHMEDVHRAGGIMAILGELDRANLLDVSVPTVHEKTLKDALDKWDIIRTEDPEVYEFYRSSPGGVPTQVAFSQNRYYSTLDGDREKGVIRNAEHAFSKDGGLAVLYGNIALDGCIVKTAGVDESILKFTGTARVFESQDAAVEAILGNEIKAGDVVVIRYEGPRGGPGMQEMLYPTSYLKSKGLGKDCALVTDGRFSGGSSGLSIGHVSPEAAEGGAIGLVEDGDTIEIDIPNRTIHLNIDDATMAHRRTVQEAKGWHPKEERKRKVSKALKVYAMHTTSAAKGAVRVL

ST25 4190 cg72_0850

VKIIFAGTPEFAATALAALLKTSHEIIAVYTQPDRKAGRGQKLTPSPVKQLALEHNIPVYQPLHFKASTEEGLAAQQELAALGADVMVVAAYGLILPQAVLDTPKYGCLNIHGSLLPRWRGAAPIQRAIATGDDETGITIMQMAAGLDTGDMMYKTYCPITSEDTSATLHDKLAAQGATAICAVLESEETLQKYLAEREVQDESLTVYAHKLVKSEARIDWSMNAVQVDRNIRAFNPWPVAFIQLDENNALRVWNSTISNQNKADAQAGEIIAIDKQGVHVACGENTFICLTSVQWPGGKALNAQQIAQTQKLHVGQILP

ST25 4190 cg72_0851

MSQVTSNSSRFNLRAQVVQTLLKVQQGQSLASILNTQLNQVAERDRALFHELVLGTLRQWFALKSISLPLLSKPLNNETVETCLYVGLYQVLCTRIAAHAAISETVDATKQLGFPALSGIVNAILRRATRETDDFQQGLQQAHGLPSWLFKRLKKDWGEQTEALCQSLKQAAPLTLRVNQRHIGRDAYLAKLQSLDIQSRACLFSEAGIVLEQSVQITQLPGFEQGWFSVQDEHAQLCATLLPDLNNKTVIDACAAPGGKMAHLLEKFKPAQLIAIDQDPSRLVRVIENLNRLALDQSHTEILAADATKWNPVQPVDCIVLDAPCSATGVIRRHPDIRLLRQSSDIAQTIELQKQILEHMWQQLKVGGTLLYITCSILKSENEQQMINFFTEHPDAKEVKIEADWGIEQVHGRQLLPEAQSGDGFYYCKIQKIT

ST25 4190 cg72_0852

MVFVWGGLALSILHLTKHPEELDEVLEEVKDQHTL

ST25 4190 cg72_0853

MTDTRENWTSRSGFIIAAVGSAVGLGNIWRFPYVAYENGGGAFLIPYLLALIIGLYYASVLTWAGSYVYFSIGQMWGSDPEGFFFNTYLQTTKATGFDLQFVGHLFWPIVGIWALTLIILYGGVKKGVELSNKIFMPLLFILFTILVIQSLRLPGAVQGLNAFFTPNWSAMMDYKVWLAAYGHTFFSLSVGFGIMVTYASYLKPKTNLTGSGLIVGFANASTEILAGIGIFAALGFMAHAAGTEVKDVVSGGIGLAFIAFPKIISSLGSGADLFGLLFFSSLFVAGISSMVSILEVPIAAMQDKLKWGRKKAVTIIGGGSALVSIILFSSVNAIKLVDIVDHFINNIGIIGGALLSIISVAWFKRSALKELRDHVNRISTIQLGKGWDFTLTVITSLILLTTLCMTVFNLIKNGYDTYSMSLQGVFGWGSVIFCAVVAIVLSKMKDR

ST25 4190 cg72_0854

MQKKEPKNTFVFVENKMHLLSIFIPLTNKQK

ST25 4190 cg72_0855

MRSKIFFLGLMMKILKLSFLALGMGLSGFAQADFIGVKGDVGYWFYDGKANMSSQSLEDQDLDRKGSAQLSLAFEHPIPFIPNAKIRYVNLDTQTKSETLGQANYNVDLDHSDFILYYELLDNIVSVDAGLGATVLNGDITAYTGKRVDIDKTYPIAYLSGEVKLPFTGLSAKGEATYTNFDDAKITDALVEAKYKFADNLLIDLGLTAGYRILNIDLDDYDNNDLKFEFKGPYVGLEAHF

ST25 4190 cg72_0856

MIIYGDVDSGNCYKIKLLLSLLNINHRWIHVDILNKDTQTAEFLSLNPNGKIPVLALDDGRVLSESNAILGYLAEGTELIPADPYMKAKMYQWMFFEQYSHEPFIAVARFINKYLGLPPERIEEYHKLQPKGHKALSIMNKALVEHDYLVGNKFTIADIALYAYTHVAEEGGFDLKLYPNIQEWCQRIQKCPKYVSMVE

ST25 4190 cg73_0858

LKVANSLSLTPQLQQAIRLLQLSSLELEQEIQIQLDSNPLLEKVEDESLAESLSTLEHKETDDLTTELNADHLPDDLPVDTEWDDIYTHQSTALGTPEFEEREDNRQVHLTLKEHILEQVNLLHFSKIDQLIAYCIVDALDDKGFLDAELEEIILAVQHLLSEMDIDEEVEEDEVLVVLKHIQRLDPIGIGARNLAECLKVQLEFLPRETEYLKEARSLLQYYELLIANDLNKLLKQTGLSKEQLKFAVDLLKTLKPYPGMDFEKQESEYQIPDVVVAKKDLHWQVQLNPDVMPKLRINSFYSSMIRRADQSDDNLYLRNQMLEAKNFIKSIDERHKTLLKVATCIVEHQKAFLEIGPEAMKPLVLRDVAEEVELHESTVSRVTTNKYMLTPRGLFELKYFFSSHVGTTTGGEASSTAIRAMIKKLVSNENPRKPLSDNAIAALLKEEGIEVARRTVAKYRESLHIPSSSERKVLI

ST25 4190 cg73_0859

MQITIRGHHLAITPAIEENIKAKFNQLTKHLDQVNSMQIKLTKDHQIDKRSHKGSSNHVAEAIVRLPGIELFAQATADDMYTSIKKLTEKLKKQLLKYRKMQCTYSQVAVSI

ST25 4190 cg73_0860

MNSEQLTQILKEAFPEAEVVVSGQAGKFDLRIVDDQFEGKRTVARQQAVYAPLNTYIASGEVHAVTIRAMTKDEWRKASLFGA

ST25 4190 cg73_0861

MDKFLITGGVKLEGEVRISGAKNAALPLLAAMILADSPITLTNVPNLKDVNTLVKLIGGLGVTISYENDTVKADTSTLDNQFAPYELVKTMRASILVLGPLLARYGNAKVSLPGGCAIGSRPVDQHLKALEALGAHIEVENGYVHATVDGRLKGGEVVF

ST25 4190 cg74_0862

MQFKIIAAQDLDRNQRHQIAELCFSAFDEDPWTQYAFMQKAIHVVGILNNQIVSHALWTDRVFTINGSSDVKTAYVEYVTTGYTMRGKGIASQLLKYLVETLTHLEYGLAALQPEDEAFYEKLGWTVWKGNLFIKLNTCSYLTDEYEIMLYPLNIQMKDQLSNSSEEDTICADWREGELW

ST25 4190 cg74_0863

MSTDPQFPTPNNLGIAVYSNNAEAIGNTPLVRINRLIKTGATVLDQNRKS

ST25 4190 cg74_0864

VKCRIGAALIADAEKRGVLKEGMHIVEPTSGNTGIALAFVAAAKGYSITLTMPASMSLERRKVLKALGANLVLTEPAKGMKGAVDEAVRLATEQPEVYFLPQQFENPANPQIHVDTTGPEIWQAIGGQVDILVAGVGTGGTITGISRYFEQVQNKPLYSVAVEPAESPIITQTKNGENITPAPHKIQGIGANFIPKNLDLDLVDEVLPVSSEEAIQWARKCATQEGILVGISSGAALAAAAKIAERPENAGKTIVVILPDSGERYLSSVLFEGLFDE

ST25 4190 cg75_0865

MTKRYNYVIGDVQGCFEALKALLKEIRFDPDQDFIWFAGDLVARGENSVGALRFIKKLADRGAAATVLGNHDLTLIAGARGLKEIKEKDRTQDVIDAVDGDDLIDWLRKQPLCLFPNEQTILTHAGVPCIWDAQKTAALAKEVEAVLAHDDLSVLDAFLAEMYGSKPDLWTDDLTGNERLRCITNYLTRMRLTNAEGALEFSFKDTLDAPMPEGYLPWFEFPSKAAQTHQIFFGHWAALEGRTISEHIQNVDGGCVWGKNLIAYRLEDQQAFSVSNPVM

ST25 4190 cg75_0866

MYQINALNPKDEGHKARKRFGQNFLHDQRVIAKIVRSVNPRTGDNIVEIGPGLAALTSPLIGECDALTVVELDRDLAAGLPERVPHPERLTIVEADALKYDFSQLVKDGRPLRVVGNLPYNISTPLLFHLLEFGSQVKDMHFMLQKEVVERITAEPNTKEYGRLSVMIQYYCQPTFLFEVPAGAFNPPPKVTSAVFRLVPYEQKPITAKDEKALARLVAHVFTQRRKTLRNSLKGMLAEDGFEKARVDPMARPETLTLAEFVALADQMVA

ST25 4190 cg75_0867

VSGVIVLPLYVTSGEPAGIGPDICLSLAKRVDERPVVVLADRNLLQQRAQKLGLDIKFLEYSGQAESSLQGELYIEHVPLETIVVDGQLNAANAAYVLEQLRRSADYAMSGKSVGVATAPVQKSVINDAGILFSGHTEYYQEFAGVERVVMMLATKTLRVALVTTHLPLRDVADAITKERLHQVIDILLHDLKTKFKINDPRVLVCGLNPHAGEDGYLGREEIDTINPVLESYRSQGVKLSLSLPADTLFTPEHLKNADAVLAMYHDQGLPVLKSQGFGEAINITLGLPFIRTSVDHGTALSLAGTGLAKSSSLNVAVDLALSLAAS

ST25 4190 cg75_0868

MKTLSAKPAEVQHDWFVVDATGKTLGRLATEIARRLRGKHKTSYTPHVDTGDYIIVINAEQVQVTGNKALDKKYYRHTEFPGGLKETNFEKLVAHKPEEIFERAVKGMLPKGPLGYAMIKKMKVYAGSEHPHAAQQPQVLDI

ST25 4190 cg75_0869

MATNYGTGRRKTATARVFLSAGTGKLVINNRTLEQYFGRETARMVVRQPLELLEATEKYDLYITVKGGGIGGQAGAIRHGITRALIAADETLKPVLRQAGFVTRDAREVERKKLGLRKARKRPQFSKR

ST25 4190 cg75_0870

MSVENTSIQGITLYSHADDFRSHWIRFLLAEKQIKYQLIVTDHEDEDLASLNPYNQLPMLVEQNLKLFSAPIIAEYLDDRYRQNKLYADAPMARAEQRQYIWRLENDWFKLADHMLRHADTLNVEQKQKAQKELRDTLISLTPLFQHFPYFMSENFSILDCMLAPIFVRLNSMGIDLPKQQCRPIFLYCKRIFSRPSFVKSMTPQEKTRYNELLNME

ST25 4190 cg75_0871

MSEQTIELTPTRPYLARAIYEWICDNQLTPYLLVDATQPHTDVPQQFVKDGQIVLNIVPHAVHQLLISNDAITFSARFGGVSKDIYVPIQAVLGIYARENGQGLFFDPEEYANVAPVEDKLDSETQETSEPTKKKPTLRILD

ST25 4190 cg75_0872

MAFDLVQYFAEQIKIQKPQLLNQYPANEKNKLIDEVNILTLGKLISLWRQDDNKIYHEIKTADPLYIQEVARHLTTSKHNKSVLKNSELEQSISEILALQLTELNQLDETGGFGQSGLKELILGQVEHLSGQAEDWVWSTNHLTELIGSKPVEQEELSLDATMKEFNQMVHQAQPHHEDLHVEEQPIETFIPGWSKVIAPLVALAILGYLYCMYTQLV

ST25 4190 cg75_0873

MKFSKVLVANRGEIAVRVMQTAKAMGYQTVAVYSDADRHARHVQEADEAVYIGASKVSESYLSITKIIDACKKTGADAVHPGYGFLSENTDFAQACIDNQITFIGPTASAIELMGSKRLSKIAMIEAGVPCVPGYEGDRQDLEYLATQAEQIGFPIMVKASAGGGGRGMRLVQQASELFEALQTARSEAENAFGSGELILEKAVIAPRHVEIQVLGDTHGNYVYLFERDCSIQRRHQKVVEEAPCPVMTPELRQKMGEAAVAAAKACAYVGAGTVEFLLDASGAFYFLEMNTRLQVEHPVTELITGLDLVEWQLRVANGEHLPLQQHELTLNGHAIEVRLYAEDPRQDFLPQTGQVLRWQPATLPNVRIDHGMLATDEVSPFYDPMVAKVIAYGKTREDAIRLLARAVNDCVLLGVNSNKQFLVNLLRHPIVVAGDTNTAFIQQHFQNDSSLHKQVLSLETLAIAAALFSQSNGTAVWQTGLGVPLPLKLKYDDQQVQLQLSSLNNTFTVQLCDQTICIEVLERMPEQLVYLIDGVRRRVQYVLNDDQVYLDRDNGNVAIRNVTYAAPEIADVAGDGKIRAPMDGAVVNILVNEGDQVVKGQTLLILEAMKIQQQIKSDVDGVVDEILGQQGQQVKKRQMLFSIQI

ST25 4190 cg75_0874

MTLSASLQVLDLDDSIQLEQDGGILYLWLNRPESRNAMNLNMVNAIQQVFAAIRDDLSIRAVIIRGEGGTFCAGGDIKDMAALRVEAANVGSLQPYVDFNRRFGALLEQVEAAPQTVVVILEGAVLGGGFGLACVSDVAISRDNAQFGLPETGLGVIPAQIAPFVVKRIGLTQARRLALLGMRFEGHTALNVGVVHQIAHNEIELEQALQETIQQIKRAAPQASRVTKTLLHRTLNEPLSGLLDDAAQQFAQAVGGTEGQEGTMAFIQKRLPNWADES

ST25 4190 cg75_0875

MKFTAEHEALRRTARQFVENELNPHIPEWEEAGRFPIHDVFKKMGDLGLLGICKPEENGGLALDYSYNLVVAEEIGRAACGGVPLAIGVQTDMATPALARFGSKELRDEFLTPAIAGEYVASIAVSEVHAGSDVAAIKTTAKKDGDDYVINGSKMWITNSLQADFFCLLANTSDDKPHVNKSMIIVPAKTKGISFSEPLNKLGMRSTTTAQVYLDNVRVPQRNLVGVEGMGFMMQMMQFQEERLWACANAIGGLENLIQKTIAYTKERTTFGQPLINNQYIHFRFAELMTEVEALKALTYQACEQHIAGEDVTKLASMAKLKAGRLSREVADSCLQYWGGNGFMWDNPASQLYRDGRLGSIGGGADEIMLGIICKLMDILPKKQK

ST25 4190 cg75_0876

MTILQSEITVGSEQYQKNKEALLAQLSEVRAIQQKSIDKSYAAKPKFDKKGKILPHERIRLLLDADSPFVELCGLVGYNMHDDKDGSEAGGGVIAGIGFVSGVRCLVSASNSAIKGGTMSPMGVQKTLRLQKIALEQKLPLITLTESGGANLNYAAEVFTYGGMTFANQARLSAAGIPQLAVVHGNATAGGAYQPGLSDYVIVVRKQTEMLLAGPPLLLAATGEVATAEELGGAEMHAQVAGTAEYLAENDADGIRLAREIFEHLNWKEQIKPVDVTYKEPRYDTEELLGVIPSDPKQPFDMKEVVARIIDDSDFLEFKQEYDDLTVCGWAKIGGLHIGIITNNGPITPQGAAKAAQFIHLCEQTNRPLLFLHNTTGFMVGTDAEQNGIIKHGSKLIQAVANCTVPKISIVVAGSYGAGNYAMCGRSLSPDFIFAWPNSHVAVMGAAQAGKVLRIVAEGKQKASGQEPNPQMLDFLEQSTAMKLEQQSTALFNTAMLHDDGIIDPRDTRKLLIFLLETIYEAQQRELNPTRFGVSRF

ST25 4190 cg75_0877

MDETKMSYQSIFRPDAFANKVIIVTGGGSGIGRCTAHELAALGAQVVITGRKIEKLEKVSQEIIEDGGLVHFIVCDNREEEQVKNMIAEVIEKFGKLDGLVNNAGGQFPSALENISANGFDAVVRNNLHATFYLMREAYNQWMAKHGGSIVNMTADMWGGMPGMGHSGAARSGVDNLTKTASVEWGKSGVRVNAVAPGWIVSSGMDNYSGDFAKVIIPSLAGNVPLKRMGTESEVSSAICYLLSDAAAFVSGVTLRIDGAASQGTRMYPLAEATNSQSYNGFHRAFIPEIFKKDKTEKVEQSNNSGSE

ST25 4190 cg75_0878

LNIEIDFGGKRYPVEIPQGVSTEQLLTLTAGENAVYQGDEIEVPLIEIAHARSGDKGNHSNIGVIARKADYLPWIRAALTEQSVASYMQHVLDAEKGRVIRYELPGLNALNFMLENALGGGGVASLRIDPQGKAFAQQLLDMPVKVPAHLLEK

ST25 4190 cg75_0879

MANNQQDDHLVVKIGCASGFWGDTNTAAFQLVHLTDINYLVFDYLSEITMSIMAKAKMVEPKHGYALDFVSRVMAPLLKKIAEKKIKVISNAGGVNPLACRDALQKIIKEYGLDLKVAVVLGDDLLPKHEQLKSQNIQEMFSGEALPEQVASSNAYLGAVAIRDALDLGADIVITGRVVDSAVVLAPLLHEYQWPLDDYDKLAQGSLAGHVIECGAQCTGGNFTDWQLVQGFDNMGFPVVEVSEDGSFVVTKPQGTGGLVSTATVAEQIVYEIGNPQAYLLPDVIADFSHVHLEQVGEHRVRVMGAKGQAPTTQYKVSATYPDGYRVLVSFLIAGREAPQKAQVIADAILTKCERVLAMRSVPPFSEKSVEILGIESTYGDHAQTLNSREVVVKIAVKHMFKEACMFFASEIAQASTGMAPALAGIVGGRPKTSPVIKLFSF

ST25 4190 cg75_0880

MIATSIEQIPYIPCFDDSPRGRLLHGAAYLFHKQGYDKTTVRELAQFIGIQSGSLFHHFKSKDDILAHVMEQTIIYNLARLNDAVTQSTDPEQQLRALIKAELISITGDTGAAMAVLVYEWFALSKEKQDYLLKMRNEYEQIWLDVIEKLRTQGKVKHDAFIWRRLIGGAISWTVTWYKSEGKVKIDELTEMVWEMALK

ST25 4190 cg75_0881

MQLNPIYYNEQHIAFADNVRRFVQKEMTPFVNEWDEAETFPRELYKKAAEIGLLGLGFSEEYGGIPDADPFYSLLAGIEMAKAGSGGVHISLMVHTIGAPPIQHFGSAELKARVLPGIISGEKISALAITEPGGGSDVAALQTKAVRDGDYYIVSGEKTFITSGIRADYYTVAVRTDPTKTGAEGISMLLIDAHSEGITKTPLKKMGWWASDTAHLHFDQVRVPASNLLGKENAGFKVIMNNFNMERFFLGVVAYGYALVCYEEALEWAQQRKTFGKRLIDHQVVRHKLVDMATQLTSTRALLEETAWKMTQPKLQGPELVAQISMLKNVATRTMQFCADAAVQTLGGMGFMRGTKSERIYREVKVNMIGGGAEEIMKDLISKQLGY

ST25 4190 cg75_0882

MMTKKQLTSKQIDMLLELLKNRFEKNIHRHKEIDWTNVRAKLIESPDKLWTLQEMENTGGEPDVIAYDQQKDEYLFIDCSVESPKGRRSLCYDREALESRKDHPPKNSAIDLVQEIGAELLTEEQYHQLQQLGEFDLKTSSWLATPEEIRKLGGALFADRRYGRVFIYHNGAQSYYAARGFRCCLRV

ST25 4190 cg75_0883

MEYKGSCHCGQVKFAAEGELTEALSCNCSICQKKGALLWFLPTNQVTVTLDSPDILANYTFNKHVINHHFCKNCGIHPYAQGIDAQGNSIFAINVRCIDDIDLEKIKINYFDGRSV

ST25 4190 cg76_0884

MKRTFQPSELKRKRVHGFRARMATKAGRQVLARRRAKGRHSLTV

ST25 4190 cg76_0885

LGVMALYSFSTDLRIRCAADYKSVFDGALFKVHQPHFLFLAKLTEQPNSRLGIVVAKKKVRRAHERNRIKRLARESFRLHQPKFGSDIDVVVMPKVGIETITNAELYQQLDFAWQKLQRLAKKHSKVVPTSQN

ST25 4190 cg76_0886

MVRILRWFIRLYQIAISPLLGPRCRYIPTCSQYALEALQTHGAIKGVWLSSKRICRCHPWGGSGYDPVPPKAIRFISFHQIDSQTHHVAVPFRDRLMKQNLSNHLG

ST25 4190 cg76_0887

MFVTAYLLILAWQKDYGNAATAPQQQAAAVSSHEVSADLPNAKTATVASDVPQANIAQSQTTDATAPVNQQLISVQTDLYHLWINPKGGDIVRIELLSHDKSKDSNQPFVMLENDAKRTYVAQSGLIGLNGPDSSRGGRPLYAVEKTSYTLADAKGVQDQSGHSEKVLTVPMVFKTPEGVEIIKTFTFTQGQYPIVVKHQVVNRSQQNWQGQMFGQLKRDNSEDPGKSSQGIFTLGTFLGGAWGTPDAHYNKLKFANFSEEKLNTEAKGGWVAMVQHYFVSAWIPGNLKLTQANGQPYVAKLESRQSADHMNIIGFTSPVINVPAGTSMEVDAKLYSGPKVQSELKDLAVGLNQTVDYGWLWPIAKLLFLGLQFFHSIVGNWGWSIILLTIMVKMILWPLSSKSYRSMAKMRVIAPEMQRMKEEFGEDRMRFSQEMMALYKREQVNPLSGCLPLLLQMPIFLALYWVLMESVELRHAPWLGWIQDLSAMDPWFILPLIMGATMFAQQMLNPQPADPMQAKVFRIMPIMFTVFMLFFPAGLVLYWIVNNSITILQQWFINKSVEKSRISKAESAS

ST25 4190 cg76_0888

MHSQTTIAAIATPPGRGGVGVIRLSGPKAYDIAQKLTQKNLPEARMAGFRKFYDADGSIMDEGIVLCFPNPHSFTGEDVVELQGHGGPVIQNALLGRLFELGAIAAKAGEFSMRAFENGKMDLVQAEAIADLIDATSQAAARSAVRSLQGAFSTKINTVLEKLIHLRLHVEAAIDFPEEEIDFLADGKILALLEDVQQSVHAVQTSARQGQLLREGLQVVIAGKPNAGKSSLLNALAGVERAIVTDIAGTTRDVLHEKISLNGLPITLTDTAGLRETGDVVEKEGIRRAIKEIEQADLLLLVYDLNQGDDPLKLAQEYFAEHIEPRRLMLIGNKCDLTGQPAEISDYQGFRHITVSAKQEMGVQGLVDAITAHAGFHPEEDTFIARTRHLDAMKRTQLYLAEAREQLVVFNAGELVAESLRLAQNALGEITGDFSADDLLGKIFGSFCIGK

ST25 4190 cg76_0889

MSHIGQDKKIVNRVKRLKGQINSIENAIEQPESSCIDILQQVAAIKGAINGLMSELMEQHLHHHVLKGAEVDQNELDEFLKVLKRYG

ST25 4190 cg76_0890

LGLNFMQDYSVSRHHQHQFDEGNPLAQKRILIATILTASMMVLEVFGGWFFNSMALLADGWHMSSHMLALGLAYFAYRAARHYSNDHRFSFGTWKIEILAGYSSAILLMVVAIFMAFQSIQRLFNPVEIFYNEAIPIAILGLVINLICAWLLHDDGHHHHHHHHHHHHHHHHHEHGHGHHHHDLNQKAAFLHVVADAVTSVFAIVALFAGKYFGWDFLDAILGILGAILVAKWSFGLMKETGKTLLDAEMDHPVVDEIREVIAEFPKHVEITDIHVWKVAKGKFSCILALETDDISLNADQIRDALSIHDEIVHISVEINTLKPVYVPRETLA

ST25 4190 cg76_0891

MIKKLGILALFSLTTSLSFANVDTVRENIKKQYPNLKISNIQKTEMPGLYSANLDQQIIYLGEDGQHMFVGSMIRLKDQKNLTKDLVLGQNSIDWKQLPLKDAIKTVKGNGQHVLAVFSDPNCPYCKQLEPELDKLKDVTIYTFIYPLKPQSIVVSRQVWCAPNQSYSWKKLIEQGVKPTVASCTNPIDRNLELGKKLGFNGTPTLIFANGFKLVGARSAEEIQAVWKELGL

ST25 4190 cg77_0892

VKINSFSPIPEDDEELSLPELIYWASLGDLEQVKQLLIDREDPNQTDDEGYTALQAAAENDHLEIVKLLVEKGAHVTYKSEYTALQLAEMAGNTDIVNYLKSL

ST25 4190 cg77_0893

MLVYIIVGSVREGRTAIKIANWLETTIPSFNLKDFQTEVIDLKEWNLPLFSGSHPPATGIYDQPKQQEWADKIAQADAFIFISPEYNHGYSPALKNALDYVGKEWSGKPAAFIGYGSTNGSRSISQIRQVTSSLSIVDPNAVIEIRDIFKRNKDEKFEANEFEVKGLQSIVEKLQKYHLS

ST25 4190 cg78_0894

VKNGFKLKVSTNNGTLTQEQTVTVTGSNSTVEADTTSIVLFDATKTSLNVRGDQTTVTLTAVDANGATLANQAITLKVRNSVLNGVKFTPNATQTDVNGQISYTLSFSENQRTSSYSAGQFVTDDLVLEANFGQSTKIYTYKLDVVNSDVPVAVGAIAVAYNPTTMEDSSDGVYYYKNISVQVTDVDGKPIPNQDVVMGVNALAYYKGRYGFVDTSNPTDGKPDEYLPVIRTACTSPVQAVNVNGDVINQLKPLKENSTVQVVSYINSAGTAATNNKYTTDANGRFDLKIQYPKIYASWLTVQLTAKSTVSGSLIKGSTSLTLGYLTKDVSLTDLNGPNMESPYGIDPNCSNGN

ST25 4190 cg78_0895

MKSMYKKQLTLKLSAIAVSIMLASCGGGGDGYYQNGSSNSNNPSSGSNNSGSQQATATALKIELDKLSVAATNDTLTVTVRALDKDKGGVAGANVTLELDDPTNNVSIEGVSTQTTDAAGNAIYIIKTPKTSSSINRVGEKWF

ST25 4190 cg78_0896

MFDLIIIGAGTAGISAYKEAVKYTNNLLIINDGPWDTTCARVGCMPSKVLISTANRMYDIQNAQEVGLSASADISTDQVMEHVRTLRDRFTKATLKDVEQWPTEHKISGKAHFIDAKTIEVNGKRYQSKSFILAVGSTPNYDQTWKQELGDRLITTDQIFELNTLPKSIAIIGSGVIALEIAQAMHRVGVETTIFARSKRIGIFTSPKLQQLAQEELSKELNFLFETLPHEVKSTSDGVILNYKIDEKEESIQTEYVLSATGRSSLLDTLKLENIDKSFKDIKLLPVNAKTKQLDNYPIFIAGDAYTSTPLQHEAAHEGKKVVYNCLNYPQVNAVKTLTPLGIVFSHPEMAIVGQSYKQLKDNGVDFVTGEASYERQGRAIVLGKNKGAIEVYIERESQKLLGAELFTEATEHMAHLLSWIIGEELTLNDILEKPFYHPTLEEGLRTALKHARRQLK

ST25 4190 cg79_0898

MVGLTRLELVTPRLSSVCSNQLSYRPSDTSMKNNLLWILTNRQSFVKEVIQPQVPLRLPCYDFTPVIGHTVVTALFAVRLATSGATNSHGVTGGVYKARERIHRGILIRDY

ST25 4190 cg79_0902

MLSAVIPSEHSYPAMRLASQPVHQRFVHSGPLVLGADPLKFPAPTVDRDRTVSRRSKPSSRTSLNGEQPYPWDLLQPQDEMSRHRGAKHRRRYELLGGISLLSPEYLLSVERWPFHTEPPDH

ST25 4190 cg80_0906

MALTNCKECGAQVSTQAKNCPSCGAKVKKRSLLKWIFLGFVILFIIGIIAGGGEGSSSSSSTRELSPKEDALKNTVLDYDWSKGGFDSVMLVDFKIKNNSKYDIKDITVECEHYSNSKTKIDSNSRVIYEIVKAGETKTVKQFNMGFIHSQAASSGCGITDLVVIQ

ST25 4190 cg80_0907

MGKDYDYVRNEMDLPRLRALSAYQQSNPPAHIGIQRLCRILEAFMGIDETPQAITVSDDDEDDMLEVLSNFPQGG

ST25 4190 cg80_0908

MQFKQVDNPRGNSKEIAGQTWIFAPAPLGTLERFQEQLSSNDVPVSVIVDMAHICLKRNYPDITREYVSDELLDMANMEEVLALVTKTSGLEYTGKPAGESSGE

ST25 4190 cg80_0909

MAQYLFGAGKIFATPIQDVYGQPIINPTPVEVGVMQSVGVDISYDLKELFGRGQFAVDAARGKGTIKCKASFGRINGALLNSIFFGGVVAEGGIETVSQTINGEVIPAGGSVTPVVPNSGTFVKDLGVTDAKAIPLKRVASAPTTGQYSVDAATGAYTFAAADVGKTVFINFRYSAMVAGAKSITVSNLDMGYTPEFAVDLQRDYKGKFMHMNFFRCTSNKLGFSSKQDDYDIPEFEFQPMADDLNRVFKIDLSE

ST25 4190 cg80_0910

VDSEAIYQALFDRLSTRVEGLKTVSRRLRHFNHVTPEQRPAMFITQGNQQEVPVHGLDSKVELAAEVYLYIHESDTTKPPSSQMNIFIDRVREAIQPDHPDFNECQTLGGLVEHCWIEGTIEVYEAVENMLDDQAIAIIPIRILTTN

ST25 4190 cg80_0911

VGAGGALVPYAPAHEFGLNGALGVKAHLRTIKQAFGRPISPVQVNVKAHSRNVRFRELRFMRDSLDIVAKIVPKNIDAAIQRGIAGG

ST25 4190 cg80_0912

MPFKYQAPEGYKPTKLVIAGQNLDIKNGVLESDNDIIHILKPLGFERYVEVVEPKKSAASAKE

ST25 4190 cg80_0913

MSLPDARIQDALEEADQIVSQIEFGALKERAVGLYAAHILKVGTISGNGAAFGTASSMTIAGQSVSYSRSSKEAFYDLSMYGQRYLALKNSIPIDDEGTNPNRLGVGVFVV

ST25 4190 cg80_0914

MKYSYIYSGLQAAFVFSGIAVLPTGTPTLVDEEAHKKLTKNKFAKHLIDIGELEVQEIAEDEPKTAGKTGGRGGKGGKQNDAAGDAAKAAEEATLAAVKAELTALEVTFSDDETLEQLQAKLAQAKE

ST25 4190 cg80_0915

MSKLAAMKLRLTPVAQVVQATIGDAFNLDALAQLFVKLEEFNEMDPQLQQVMDYAKYIPVKPVNGVFGGGEVLTRKKGVGIGKDHSGTGNDIPVAEVDYDSVSLPIKVGTISYWYSVLELETAQKMNIPLEADKVQAARLAAEKHLSNIAWYGNELTGVKGFLNQTGVTIVSAQHNWATATIEEVLSDFNASLADAEDLIDGDASVQPDTYLMASNQYLHLSTRVVADSGGKTFLKFIEENNIFASQGKPLTIRGLGRSNGKGTAGADRSIIYRRDPSCIQMKCDDVTFLAAQPVGVDIKVPGHYKYQGVWLKRVDSLRYLDHV

ST25 4190 cg80_0916

MVKQYDAVPGMKFHLIGPEDILSLPVAGTGLVNDGDVVVRSTDGKTVSAVTGATNTKFGIIVRHGVGKSGKTADGKEAYKATDVAPVMTIGSIYVKVTAPVTDINAKVYVKTANGTTAAPLGSLSPTATDGTELPNASWETISNEQGLAAVRLRGA

ST25 4190 cg80_0917

MTKHIYQLKIGDFAPSESTRSFTKEGYLKCVNVRLAKAPQVRQYYAYEFPSLEGYTADQIINVYTPAEELFKPEAIQSFNGVDATDYHPPKNEINASNWKDYHIGYCENVRQEGDYLVGDLLIKDKISIDLIQSNERLEMSLGYGALLIVEQGTAPDGTPYQAKFINFIGNHVALVKYGRCGGDCRIGDKQQTPPKGKTMEVIVNGIRFNIGDNTPLADALKQQQEQLENMKAAKLKVGDKQFSIGDELGAIQAVVDQLHAEKTALEQKVGDLEKNQMTPEKLEQAAAERAAVIADAKALVPTVKTEGCTCEQIKRDVIAAKAGDALVTALMGNVSVGDAKPEQIDTTFRALCAVKGTHPSNPVGDALHQQQSVKAGDGNPAGGGEEKTYSKENAYKTI

ST25 4190 cg80_0918

MAMKRKKFSKKRFYRRLQAQGFAKGGFVTGGDFTPPWLMSCCEGFPNLAKAAGKTAEKFQEVMESIKKIDFHPPKITTVSANLYGDGLILGRVEHFEICGSDLALDIRM

ST25 4190 cg80_0919

MNQIRELTLKKVFLSVLLISIPILLWKLDSIIMALKA

ST25 4190 cg80_0920

VHVNRRVELYYTRQLLAISKYCQEQTKELVIPTVGQNIGDAWFSDMMTAFREKLTKYVVEISRPLATKVVTDTQKEVDKQIAEHTKTIIGVDLTPFYRAADIQDEVDLNITVNVSLIKSIPQQYADKLEVLITNALQTGQTNEELAKAIKQLGLSTDYRARLIASDQMGKINGQINRARQLSMGVETYTWQTAKDERVRPDHQHKQGKTFRWDSPPDGGHPGQPIRCRCTALPNYEDILID

ST25 4190 cg80_0921

MAETKKPDAIGDAGAYTNFVSNIGTERDKASHGSFVKKVIPDEQLEAVYQHWLAKRIVNRPASDMLRAGWFFEGIQDNDLLKLKEACKAFNLDGVLLSSLVLSRLYGVCYVLLGTVDGGNLDQPFDLNKLGVGRLEFFTVLKKKHIEADTSKYLSPKEAGGVLKQPEFYKLKLDGKSNQRIHHTRLIKFGHADVVNEEPVSVLQEVYEDLLDHAAVKKATASLVHESKIDVIRTPNLVDKIKEDMKSVAERFLSVGLLKGLNGMIVLDKEEEYDSKSYSFGGLPDLMREYSIQTAGAADMPYTILFGQSPAGMNATGEHDTRNYYDSIATKQIWSLKPFMMKLLRVIVQATFGRQIPSLDVVFNPLWQLDAKVRSEVEKANAERDSKYLEMGIITEPQIAKQLVIDGVYSVIDEKHIKELETMVKLNDNDNSDPETPPPAGEET

ST25 4190 cg80_0922

MGFKFSVNWHHEYIADKIDEVIAGKVKNLVINVPPGSGKTELLTNLIARGIARNPRSRFLYLSFSQSLVEDVSATARNIVKSEDFQNLWPVKISTSTDAKSSWKTTVDGYDAGHVYSASMGGQVTGRRAGTLANEGFTGAIILDDPLKPEDAFSQTARRKANRKILNTVNSRKAKSDTPIILIMQRLHVEDPTNFVLTGNVPGDWEQISIPALIDDEYIIKLPEHIQRKIPRDVERDEKGRQSYWPLKESLLSLLQLEKGGEDKDGATVSRYTFASQYMQNPKKLGGDLVKAEWFPRYLELPVLKWRAIWADTAQKTKEHNDFSVFLCAGLGYDNNLYIIDVKRGKWEAPELLKEAKAFINKHKDSNTKIGKLRYMAVEDKASGTGLIQSISKQTTLPIRAIQRSTDKLSRTMDVILYVEERRVWLPANAPWLLNYIEEIEGLTADWSHDHDDQWDPTIDAINDSLAKKPTVFD

ST25 4190 cg80_0923

MAALKKEVKLFIVRSLAVFNTPTETAELVNQEYGIKVTKQQCEKYDPTKRAGENLSEELRKDFEKTREMFLGKPEAIPIANLAVRLQRYESQYQKHSRNRVAALSILKQAAEDIGGKYTNKTEITGAGGGPLQSENITYVTATDEQVRQAIDELENEY

ST25 4190 cg80_0924

MDQIRPFPPTDFIDQADEEEAIRLTPAPDLKKWVVANYLTIGGPLYNPDHDHIAELLHDNEEFLAFAWASSAYKSKQAMVLGQCEKVMFNVGGWRKARQEQQMRDWFGFVPTYLITVDASFCERANDTEFCYLLEHELYHIGVERDSDGEIIYSDHTGLPKHYLAGHDVEEFIGVVKRWGANENVKRLIEVAKNPPFVSDLDISKCCGNCVIT

ST25 4190 cg80_0925

MFAENIYNKIKDEELFSHDCIENMNLLMTCIRREIKGTKFKLKYNFIDFVELFSKQLDECKVKIDVSLIPPHNSEGEYILWLAGFIEKITEGGPKPPPPIKKFIPEYMSFKSELDFLPLNEEKIQNEGKEITDYFNSKLYKATFKK

ST25 4190 cg80_0926

MNKNVELINYIDVAETVYERVYENNKISNNLIVNLNRIMAEIKNQAAEKRLKLKYSSIDFEHCLSLPLADRKIKVDLSLIPHFEDREESILWLTNFIGKICEPRKMQRQKKNLH

ST25 4190 cg80_0927

MLVEKFDFIELLRLAIAQGKAEGKKISKDVVLGELALLSPAAKLWATVLVEKVDFERIAIITPAQKQTETFYSKYDFNFQTERRIEDIPGKVEFVRGEIKSGNFFRARNKLAVKIHEEMVKKKFTPTNAQGDLTNLAKGMAEIILRGHVFVKAMCGGCQGIGKLETFNSKGFSEGAKFCEKCNGTGKRPYTLNEKMKIAGIVATKTAYIKSYQKFELFGESIVAEWENEIRSGISRSFRFELPDTQETCA

ST25 4190 cg80_0928

MSSVSIAEYRKLFPIKKNKKRRSAKQVARQPSVGEMVLATHLRACKIGFEQEYKFHPDRKWRADFLITGTKILIEVEGGIWSGGRHTRGKGYIGDMEKYNSAAMMGFTVLRFSTEQVKSGMALKQIELLIKGK

ST25 4190 cg80_0929

MDSRWIEAQRREMEKLISPELIKSRDLARQSYFDHMEKEMADHVSRSIEPLSGKKQSTLVELRESIEKLAQKYKQDAHSSSLFGDQDKARVYNCFANQLENLLKGGA

ST25 4190 cg80_0930

MSKCQHCAVEELINSYCGFAEVKALCEKLRGRYNRSGLSNTDYNELLQLEKALDQAKKFNAEGAKNGQ

ST25 4190 cg80_0931

MTKFEILSCGLLISCVTAVLCGAVVLWWLARKELDEKGYRHE

ST25 4190 cg80_0932

MTKNKLFGLADDRTDVWATPQDFFEKLDRVFNFDLDVCALPENAKCERYFTPEIDGLKQEWTGTCWMNPPYGKEIIDWVAKAAETASKGHTVVALVPVRTDARWFQDYCLGREIHFIRGRLKFGGSKTNAPFGCCVVVFRPSLIDVSWEKSA

ST25 4190 cg80_0933

VRQLIASLRTVYAAQFNKQFPATGESAIPLSVVEQIALKTLVGVQQNQFNNALGRLLTAGGRFMPSFAEFRTWCIGESWMSPEEAWSRACKFTTDRSVVITQITKYALDEVRYLIEAGQMRAAQDNFFGTYNVMVAKAQLKGRQQEFYTPPLQLEHKEPKHVPVSNDEAQKHLKSLMERLKINGRKPAPVQKLEAKEKEPELIKELGPDPFDNPHEYAEMCRREGMPIPRNIQRLIDGVNV

ST25 4190 cg80_0934

MLEHGAYTLLLDACYDREQFPTLEEALDWAWARTDEEEAAVKFVLKKFFTEIDGVYVQNHIQEELDAYKAKAETNARIAKEREEKRKKGKQGVNDLPPVVNESCNSGDEPPPNHKPLTINQEPIIDSSSNAREENSQFTPIQFAQYQIDDHKRYSMREFISEYSEFQYDFISLAQQRFVSVPEIDLRTMIQNFGDWYFANESSSLNTPSIWLVKWFSWVQNNEKQVAANRKKQEQITSVGQKTQEPGYFANLFEEQNQSQIVDVTPSKKFLVSEEVGHA

ST25 4190 cg80_0935

MTKHVPQENQITLLAPVRIYTAKELAAMPLSVMNACIEAQEKFAMLELTTQMGGGSNNNTPFNGGRISIDSGYRKITHTLQDCWRILSTSDYSPIGKAWPDQVGWVI

ST25 4190 cg80_0936

MNSFTQQIKDSRQQSEIQSFYEPALRVLGHLFEVKKQNLRNKGYDENNAAVTKVEFSEVMARQFRITQWLASQIVSSLINAGLVESFGGYVKPKAGDK

ST25 4190 cg80_0937

MQTNLSNQTSKHNLQEFLVGDVVVLTEECRTFKSNDLFEVKNKTLTSLWTIKSENHLILVSSKEIRTATVAELNAKRRLTSAEQALAEVS

ST25 4190 cg80_0938

LRSLRTDCFLCSYKSKRSLGIPDTCDIRFLSSSQRFISRMANHLKYWLLDQPRSYASLATCSATGSDKCVGMHTVFFALGFCLVMVVPKLIFVHEVSYAIDDSGLAFSSSHSAVNFPPLLIASIWAYLVSPVYSVLGNTPRFSILLMAMYVRFSNAAALDRPPTASKAYLMGFKVKSPF

ST25 4190 cg80_0939

MIMESIAERIQAALDYANLKWSAASLKLGLSAQAASNWKKGKIGKETLKELAALTGVSAGWLLDGSGSMIELADNPENADAYRPVMAWEAPDDLDPNSFMIIPHVDVKFSAGNGRLVEFEPTTRMTGCAQRMEWFHKKKVSPKNLVEVDVDGDSMEPRIPSGSVVIIDKSVNRLEQVQNRKVYAIRYGDELKIKRLSRRYDGALIIDSDNPSYEREIVEPQDLEHIGIIGKYVSHSYDGEI

ST25 4190 cg80_0940

MIATLNKSKTALTINRQEFKLALGKIGEGIEKQIASLKKAKQSYDATEMACEVINEANIFEAIIEGFNEAEGTNLKLSDISNLEQAQGWVDDFLEKYST

ST25 4190 cg80_0941

MNKKYMPPELYEYRHLTSTEQMAIHQMLISYVREDHRFNIIMMGAAEPYNLVKIISVNFENEAAGIWIHFETIVGEKLALPIDFISRIEFSGQQEI

ST25 4190 cg80_0942

MQKIEVNSRNISHVLYQHFLLTVVLRTGERFIYRLLEATTFKEFVDSEDKDKFYRSHIEANKEFKRIQLFV

ST25 4190 cg80_0943

MMLLSGEGRSGIIPAPLLQAISYELTSRQIKKSSKPHWTAYVGVLLALIAAVTGVVAILK

ST25 4190 cg80_0944

MAALAISGGSLVACQLQPAFQTKEAPSLFTPKTQPSTYGVLTAKITGKHSGVAVIKLDSFRLNVSFDFEAHPDSYGVPGSEFTAVDITQLTVNEITDINGKSYNDFTEFEDIRNINDLLKGFIERNKLVEA

ST25 4190 cg80_0945

VYKFQTDYPKVWNELTQEIDFQRRKQLAIKLRETNIPTYDRKAYKTKRGFTGSR

ST25 4190 cg80_0946

MALPIITADQTLLVQAIIVYLYADPGLGKSSMGFTAEKAISFDFDRGAHRTGELRRGAVVQVQQWSDVANLTPQDLAPYKTVVIDTVGAMLECIKTHLLLTANNRQKDGSLKLKAQGLANQTFKQYINTLISLGKDVVFIAHASEDQNGDQIIYRPDLGGKNRNELYRIADVMGYLTTVTTGEGKNARVINFKPSPTHHAKNSGALGGETGEVWVPDLKAHPTFLADLITQAKDHINTLTPAQLAAAKAQEELENWKQSCEEAEHAGDLNQLTESLDKEHMYYQNMRQAMLMRAKALNCTFDKQRGTWISPPEFNGISDQQRDELQNFIAERGLDVKTVCEHFGIDALIQIEAAKLQAVKQEIEILSKTGIRA

ST25 4190 cg80_0947

VFNVNAGYRGKGTPHGVNLIKGNKATLSVSNEGVMNKAAQERYKLMLLKYFKEGRSAMDELDHEVKRIYKMVA

ST25 4190 cg80_0948

MAMACVCLGSVGSGAEFLTLKEAKDVYSALVEKESGAEG

ST25 4190 cg80_0949

MSKVIGEVNLSPSRIEGTPDQVALHIFEEIICPSTEELIKNNPEAAKVFAYHIFGLALSQLAEFHSTKSLDKAVTVTLHNLLRQLKKERNELRN

ST25 4190 cg80_0950

MGQIVKIEASILEKIVAVAERIAQSKEERRVGREEFAHMLNIEPETLDARIREGRYQRPYKDGRKSFWLLSYVQSVVTDTKESGKVATY

ST25 4190 cg80_0951

MIDNYDSFTYNIVQYFGELNQEVKVVRNDQVTLEDIERWQPKYLVIGPGPCSPSEAGISIPAINHFAGKIPLLGVCLGHQSIGQAFGGKIVRAKTVMHGRLSDMYHSNKGIFSNLPSPFSATRYHSLVIDQETLPDCLEVTCWTNEADGSMEEIMGVKHKTLPVEGVQFHPESILSQHGHQIFKNFLDIYA

ST25 4190 cg80_0952

MQRSGTAFAASFYLKLQRIKPLMLVKPSFKKRLLLTTLLSLSATQIFASNNSEQIPIGKLPEWVIPESYDLDFKIDPAQKGYTGKTTIHLKLAQATDHIWIHGKSLTVKDVNITSAQGTKTKAKYEQASEIDGVSKIKFAKTLPAGQYQLVLDFNAAYDQQLDGIYKIEFEGKPYVMTQMEAISARQSFPSFDEPRFKTPFNIRLTIPSKYSGFANTQQTSEQIEKSGWKTLSFAQTKPLPTYLLALAVGPWQLQKGPDIGATSWRKQPIQLRGIAPDAKAEKMQQALSETPAILKTLEDYFAFGYPFDKLDLLAAPDFAAGAMENPGLITFRDYLMLLDKDSPVSFVQNSFNVNAHELAHQWFGDVVTMPWWDDLWLNESFATWMQSKITQKLHPEFNADLERITDTADAMKSDSLVSVRRIRQPILSNADIQTAFDGITYQKGAAVLNMFENYLGEEKFKQGVRNYINKHQYGNATANDLISALAEQSGQGERFTRAIKSFLDQPGVPLINTALQQEDNKVFLNVKQSRYLPVGSKGDARSLWGVPLCVRYEVPNAGSKVQCELVDQAEAKIELKGASLGSWYIPNADAAGYYQFSLPQKEFTRLTAATEKLSNTEQLAYAYAISAAFNHGDINLLAVVDAAKKFANSNSRQISTALFSQLSTIHRHVLKTEAEREHFRKVLANLYLPKLNQLGYVSKTGESAENSLWRSELVRFLALDIQVPEVRTQLLKQSDVLFAQKQLNFAQVTPELLPTILAVRVQEKGQSAFDRLSGELQRVTQPTQRLAILTALGSANQEATRQQARQLILNPRVKVGEVRTVVNSINNYGDEQGGLWSWFKVNHDAVFDRLGKSSAGRFPAMFSGAACTQQQAAQLNDFFAPRTKELVGVERGLKQTKERIQLCESLVAKQDGSIVQQLKL

ST25 4190 cg80_0953

MYLNNLKYVGALKNKRSFIFVFVALFHPLESYAQETVEQTQNQAKPDKVVRVAVTGSRISKAQKDGPTSVTVITAADIEKQGFSNAFDALNNLTQNTGFVQGADYGNTFTPAANAISLRGLGPNHTLTLINGHRVADYPVPYDGSVNFVNLANIPTAIIDRIEILNGGASAIYGSDAIAGVVNIILKKKTDGTQFNIKAGGTKEGGENLRLQLSGSKTLDKLSLVYGIELSGREPIWAADRDFMKSRTRLGEKPDTIVGRKNADTGKYLSIGGCQAFNGLFNGSVNNIGQAGADNCASGLARPTYWTVQTQNRSQNGYLGLDYELNDKTQLFADFLIGANQIENNTRSPIWTSLAATSGYFLNQDSGNYEIWNRRFAPEELGGVERINKKWKELSSNLNFGIRGDIGETSWSYEAAYNGSIYTSQLRRQGLLRSNVDEYFLGQQLGTDADGIPIYSPHLDRLSRPLTASEFESISGTTKEKDKSWAQSLTLSANGDVFKLPAGTAKLATVAEIGRQGFSIKPDQAVENGEFYNTSSSGEYSGTRTRQALGAELFLPLAKPLNLTLSGRYDRYALSDNSIDKLTFGSGLEFRPHPTLLVRGNYATSFRAPDMNYLFLNKQKGYFEKTTDYLRCSQTGQSLDKCTFKDYAPGANYTLTGNKELKPEEGKSYGAGFVWSPTNKFDISVDYWDIKIDNLVRNLSDDKILRLEADCRLGNQDINSAACIDALARVERNPANAVVDPNVIKNINIVPINAASDHTRGIDFTSRWRWKTDSFGNFLWTINYSRVLEHEYQQSKDHEKDDYLKDLSSLDWRDRFNTSLSWSFGDWASTVLVNRYGKIPNGNQTAYLSPTYLVNWSGTYQISPKASASIIINNLFDKVKRDDTGGWPYYPIGSYSPFGRQGWLEFNYKF

ST25 4190 cg80_0954

MTMNIQQALNHITKNIHLTQPQMEEIMRSIMQGEATEAQIGALMMGLRMKGESIDEMTAAARVMREFAIKIDVSDIKHLVDIVGTGGDGQNLFNVSTASSFVIAAAGATIAKHGNRGVSSKSGSSDLLEQAGIHLDLDMQQTERCIREMGVGFLFAPNHHKAMKYAAGPRRELGIRSIFNLLGPLTNPAGVKRFVIGVFSDELCRPIAEVMKQLGAEHVMVVHSKDGLDEISLAAPTTIAELKDGEITEWTLNPEDVGIESQTLNGLVVADATASLKLIKDALSKNKSDIGEKAANMIALNAGAGIYVAGITKTYAQAVAFAQDIIYGGQALEKMSVLAEFTKTLKQSQAD

ST25 4190 cg80_0955

MINIQNTILGKIVDRKHEELAARLKQRNLQDVEELAKAATPVRGFANALQHKRPGVIAEIKKASPSKGIIRADFNPAEIAQQYEQAGAACLSVLTDVDFFQGADENIAIARNHCALPALRKDFLVDPYNVVEARALHADCILLIVACLSDQQLEEMSKTAFEHQLDVLVEVHDEEELERALKLSEQCLLGVNNRNLKTFDVDLNTTIRLKKLLPASRLLITESGIATPDDVRMMQEHDIHSFLVGESFMKQPRPDQAFTALFGQPQTV

ST25 4190 cg81_0956

MNTLPDLSQLTHEQLLEFTRQLALQHQSLAQSNQKLDAKVQHLEASNQHLSILNQKYEHELALFKQHKFGSKNEHLTAKQIHLWDEAVEEDIAAVDLELERLNADKTDAATQKATVNKPKRRLLPDHLHTIRIEHEPASTQCSCGCQLRRIGEDISEKLHFRPAQFYKEQHVRGKWVCDQCDTLTQQAMPAYVIDKGIASPELLSHVLVSKYADHLPLYRQRLIYQRAGIDLSRSTLSDWIGRCGVELEPLANALKEVVLQQRVLHADETPVTIMRMGENDKKPKKGYVWAYATTQYNPVQAVIYDFQDSRSGQHAEAFLKDWQGHLVCDDYSGYKARFKSGQVIEVGCMAHARRKFHELHVTKKSQVAEQALVLIQKLYAIEAELRKKTDGTAEQRREYRQQHSQPVMQQLYEWLNQHQLTVPSSSPTAKAINYTLKRWPALSRYLDDGNLPICNNWVENQMRPWALGRKNWLFAGSLRSGQRAANIMTLIQSAKLNGLDPYAYLSDVLKRLPTHKVTQIEELLPHRWKSDQN

ST25 4190 cg81_0957

MTTNHQTSIASLAKKRRTYSAEFKQQIVQACKAPDVSIASVALQHGLNTNLVSKWIRLIDGKPGNDRSPLPNKPAFIALSCSAPLDPTPTDMLKVQITLPHSKAEIGLKWQVSKISALAELLKALAT

ST25 4190 cg82_0959

MAGFCHCAFLYRMYFPTQHYACKAVPEQFRLSFDWNLFGK

ST25 4190 cg82_0960

MLLMPETSIQQETQSTWAICRDPVLLTMVGGAIDTIGFIALFGFFTAHVTGNLVLAGAALVKGGAGLWIKLAAIPLFIVTVVITKLLIDRSQTKHKTLSYLFLFEAIFLFAFMAAGLYFEPFKNADSITVAITGGLGLIALAIRNTSSKTLIKNISPSTMMTGNTTQLGIDIANLLKNNNAANRASLLKSASIVIGFVIGALMGAVLYVYFDFWSVAPFILPILYFSYLASQQKFKQA

ST25 4190 cg82_0961

LGGLLFVSIASAGYKSNAHRDFYLKCVAAGHSRATCLCIYHRLERQYSPKLMHKLGSLSLQSPEIPRDFVKSMMRTMQKCQS

ST25 4190 cg82_0962

MDKLNQVNVMVKHEQSLAPVQPVSKLARSMWARWVFISLAWLCIALGILGIFIPGLPTVDFMILAVFFAARGSEKLHQWFRNHRYIGPLIREWQEHRRIPKKAKYISTLSMSLAAGLMIWTIPHPWFVYPAILCMAGVLAWMWLKK

ST25 4190 cg82_0963

MMNSSTEQIISNSLSLRLKQETAAEHERMHQLMSEAKVFSSKEKYAQFTLSQYYFQREIEHLFEKEGVAGLIPDLDIRGRSKQALADLNDLGIQPNGQQLQSENVQLPEALGWIYVSEGSTLGAAFLFKEAQKHLGFSETFAARNLAAYPEGRAKVWKRFVKALDEAGFDQTQQDRVVQGALDAFGYFGQALDQLDELK

ST25 4190 cg82_0964

MKSKSLFPTHKPIVMLPLPQGPKHFYKFLIVVVVIGLHVFAVWMLSHLIEPYTFKATPKVDALKVSFVSLAPAKKPSVEKTESQPKNSVTPQSTLNSSVSHAPDQPKYSPTNVQTLASKTASKEVVQNNLNDKQQAQPKTTLNNSTSLQPAQRTEMLVTHTDKKLSSTDFEQTQTEQLTQNANSEIKSGGVKTPAMQQEYSKAPSTQVEHDDVVQVSSVDVLSFGGLDYDDRELKQQNRLVELRIRINEKGQPINIQLRQSSGIQSLDERVIQAARKSRFKPHKINGRAVTIVVDFPVQLKLNRGR

ST25 4190 cg82_0965

MHFISADKAFKLEAESKDLNYQKYKTLDGVESSAFATFWKIFPDQWTFFGGLDVLDHSTQEKYMAAYQQQGVRLGLSKSWSTGFNTTLLSSYRWRLFDKYAETFLARRHDFEQNYTFVVQMPRFDFYGMTPNLTYRYNHNKSNVDWLYSYDKHNISFKLEHRF

ST25 4190 cg82_0966

LSCPFLYADEDTQLRLNQSLDQTLLQEQRQFHEQGTIRSTEQLPKLQINGQEYSVEQNPNDLAKALYLAVMQKQWLKATVYLEHYKKYVGYDRALTDFAEGAVARSQGQLKLAEQKFQSSLKQQPHNLICELELARVLFEQQKNKEAARLFISIQDQLKQSDPAVIPSGVLTTVNTFVQALKKRDSWQGSVSAGYTYVSNLNSSSEQSKTWTLYGRDSEGNIIPVREVTRGTPKAESATGLDYEASLIKRYAIEGHHGVALRALAFGQSYNDHATFNESTININAGYSYFDLKNQIGVSPLFEHKRYGNDGLYNAWGGACRMDAFYFGR

ST25 4190 cg82_0967

MKISQLFLGLVACSTAFAYAGIDGISSNESNIKIGAAANASHPGGVAAVSVQAAGAPYNAFTGFSSLKGLAQAFAAQGTSNTNVTVGSKTFNISHIPVSAMPPSHSALGNFNFGQVGTQEVYFGEWWKAGDTPASASHTVYYAGDNTNTTVPTAGTATYTVAGINGSGSNLLSGTFTANYGAGTLEGTLTGTGTAVSSLSLDGVAFNPGTAAFAGLATANGTAGIDNSGVVQGQFFGANASALAGIAQFDNVSYNTAFGGAKN

ST25 4190 cg82_0968

MILGGTVQTEQGLEFREPYQRCVDDGFPNGYLVTMATPELSFHGLLGTRFFDEKLELGARATFYKAYESPLRKNNDASVNKGYYLNVPLAWDDTWIFDAYARYQVDDYNTVEFVGSNLSNQFYIDPLTRSAMAAPGRTMKISWTTKF

ST25 4190 cg83_0970

MQMKKHSLLFIALMSTTSLYANIPIESRGLSQNDGSASNTSSSNISVPTNLNWELMQKNQQLENDIRTLRGQLEEQANDIEQLKKDLANRYTDLDQRLELLHQKVDPDSATQDDSSNATSDNTTPASAPAPQTTESNKVATVPATQTSEQQPSAPTTTTQPAPAAAQNPSNSLELEKAAYTVALDAYKQGGAKKAIAPMQNFIKNHPNSIYTGNAYFWLAEFHLATDPVNYNEAKKNYNVVANQYPNSSKAPRALYQLYSIAKDVDKNTVSANQYKNKLLSQYPKSEEAKFFNK

ST25 4190 cg83_0971

MSNHDLIHSTAIIDPSAVIASDVQIGPYCIIGPQVTIGAGTKLHSHVVVGGFTRIGQNNEIFQFASVGEVCQDLKYKGEETWLEIGNNNLIREHCSLHRGTVQDNALTKIGSHNLLMVNTHIAHDCIVGDHNIFANNVGVAGHVHIGDHVIVGGNSGIHQFCKIDSYSMIGGASLILKDVPAYVMASGNPAHAFGINIEGMRRKGWSKNTIQGLREAYKLIFKSGLTSVQAIDQIKSEILPSVPEAQLLIDSLEQSERGIVR

ST25 4190 cg83_0972

MTESTTPKFAIPELPMQIQTIRQYLPHRYPFLLVDRVTEVTDNSIVGYKNVSINEEFLQGHFPEYPIMPGVLIVEALAQVSGVLGFIMNNETPKPGSLFLFAGAERVRFKKQVVAGDQLVLKSELVMQKRGIYKYNCTASVDGIVAATAEIMISHQKTEQA

ST25 4190 cg83_0973

MKVQQYRLDELAHLVKGELIGEGSLQFSNLASLENAEVNHLTFVNGEKHLDQAKVSRAGAYIITAALKEHLPEKDNFIIVDNPYLAFAILTHVFDKKISSTGIESTAQIHPSAVISETAYIGHYVVIGENCVVGDNTVIQSHTKLDDNVEVGKDCFIDSHVTITGSSKLRDRVRIHSSTVIGGEGFGFAPYQGKWHRIAQLGSVLIGNDVRIGSNCSIDRGALDNTILEDGVIIDNLVQIAHNVHIGSNTAIAAKCGIAGSTKIGKNCILAGACGVAGHLSIADNVTLTGMSMVTKNISEAGTYSSGTGLFENNHWKKTIVRLRQLADVPLTQITKRLDHIQAQIESLESTFNLRK

ST25 4190 cg83_0974

MNKLNKLMLGLGLTVASVAANAAGYGVIDLAKVVESSTYLKQQNASLNQSVKPTTTKLEQLGKELEGLQRQAQTQGQKMKEDEIKKLQSQYQSKLNEFNSTQQGLQSRVQTSLQSMNTTFETRVKQAAEQLRKENNLDFILNKNSTVAYDAKYDLTDKMIQKVNSMK

ST25 4190 cg83_0975

MPLALVSAMAAVQQAYAADDFVVRDIRVNGLVRLTPANVYTMLPINSGDRVNEPMIAEAIRTLYATGLFDDIKASKENDTLVFNVIERPIISKLEFKGNKLIPKEALEQGLKKMGIAEGEVFKKSALQTIETELEQQYTQQGRYDADVTVDTVARPNNRVELKINFNEGTPAKVFDINVIGNTVFKDSEIKQAFAVKESGWASVVTRNDRYAREKMAASLEALRAMYLNKGYINFNINNSQLNISEDKKHIFIEVAVDEGSQFKFGQTKFLGDALYKPEELQALKIYKDGDTYSQEKVNAVKQLLLRKYGNAGYYFADVNIVPQINNETGVVDLNYYVNPGQQVTVRRINFTGNSKTSDEVLRREMRQMEGALASNEKIDLSKVRLERTGFFKTVDIKPARIPNSPDQVDLNVNVEEQHSGTTTLAVGYSQSGGITFQAGLSQTNFMGTGNRVAIDLSRSETQDYYNLSVTDPYFTIDGVSRGYNVYYRKTKLNDDYNVNNYVTDSFGGSLSFGYPIDENQSLSASVGVDNTKVTTGPYVSTYVRDYLLANGGKATSKGTYCPTDANGNSQYDTEKGECKVPEETYDNAFEGEFFTYNLNLGWSYNTLNRPIFPTSGMSHRVGLEIGLPGSDVDYQKVTYDTQAFFPIGSTGFVLRGYGKLGYGNDLPFYKNFYAGGYGSVRGYDNSTLGPKYPSVNLQETKQNDSSPEEVGGNALVQFGTELVLPMPFKGDWTRQVRPVLFAEGGQVFDTKCNIDNSVYGNKGMKINGQTITDVRKYCEDNYGFDLGNLRYSVGVGVTWITMIGPLSLSYAFPLNDKPGDETKEIQFEIGRTF

ST25 4190 cg83_0976

LEAEMNALFMIAAAALLLGPLIAIHEFGHYWVARKLGVKVLVYSIGFGPTLLKWTSKKSGIKYQLSALPLGGYVKMLDEREGNVAEQDLPYAFNRQKPWKRIAIVAAGPLINLIFAVLLFWILFLPAQEQLNTKIGKIIPNSPAAAAQLHVGDKIIAVDGKETTTWEKLNFALIDRVGETGTINIDVDRAGSEQNFVLPIKDFLKNQNESALDVLGFLPYRPVIPAVVTELTEDGAAIRQGMKVGDRIVAIDGQPMKDWFDVVEVVQRSPEKLLKIDVLRHEQLVHLQVMPQGKRDSMGQVNGVLGVKSDAGKITIPDEYKQTIQYTPIQAFEMALDKTGQISSMILNSIVKMVKGLIGLENLSGPITIAKVAGQSAEMGWETFISFMALMSVSLGILNLLPIPMLDGGHLVYYIIEAIRGKPVSEQIQMFGLKIGMVLLGSMMLLALFNDFMRL

ST25 4190 cg83_0977

LGVTGSIGQSTLKILGQHPDKYSVFAVSAHSRISELVEICKQFRPKVVVVPEQKIAELKTLFAQQNISAIDVLAGQEGLVDIASHTDVDIVMAAIVGAAGLLPTLAAVKAGKRVLLANKEALVMSGEIMMQAARDHQALLLPVDSEHNAIFQSLPHNYLQADRTGQPQLGVSKILLTASGGPFLNHSLEQLVHVTPQQACKHPNWSMGQKISVDSATLMNKGLELIEACHLFSISEHFVTVVVHPQSIIHSMVQYVDGSTLAQMGNPDMCTPIAHALAWPERLQTNVPALDLFEYSQLNFQAPDTQKFPALNLARQAMRAGGLAPTILNAANEIAVEAFLMERIGFTSIPQVVEHTLEKLENAAAESIECILDKDKVARSVAQQYISSIGG

ST25 4190 cg83_0978

MLERIVTALVLVAVVLGCMFATQSHYPMLVLMIVAAGVAGYEWYKLMPREVGAVVKPKAWGYGLLVAFVSGVALFFHDIALLLWSASILTWLVSVYWVKSFPEFDGWYNATLYVIGLILICAAVTAIFVVWQSSPWWLMYLFLLVWGADSGAYFVGRKFGKRKLAPTVSPNKSVEGLYGGILTTIIVMLVVQYQYLNLTWVQQLLFLILSLITVFGSVLGDLFESMIKRRAGIKDSGRVLPGHGGVLDRIDSLLAAAPIFATGMYILKLIGVDL

ST25 4190 cg83_0979

MTDSEEYHLPQHVAIIMDGNNRFAKKNQMQKGDGHREGKNVLDPIVEHCVKTGVRALTVFAFSSENWNRPQYEVDLLMKLLEETIHEQIPRMKKFNIALRFIGDRSRLPSHLVALMEDAEQQTAHHDAMTLTIAVSYGGMWDIANAAKQVAQAVSRGEIDADQINVDLFEKYVSLNDLPAVDLLIRTGGDFRISNFLLWQAAYAELYFTDTLWPEFTVEEFDHALNVFSGRERRFGKTSEQIQQEKIEKL

ST25 4190 cg83_0980

MINDLKKDSEQRMLKTLESLEQGFAKVRTGRAHPSILNGVMVPYYGSDVPLNQVANVGVEDSRTLIVQPFERTMVAAIDKAIRESDLGLNPITADSIRVPLPALTEETRRDMQKIARSEAENAKVAIRNIRRDVLGDIKALLKEKEISEDDERRAGDDIQKITDKYVAEVDKRLAAKEAELMKV

ST25 4190 cg83_0981

MAETISPRYSRILLKLSGEALSGNKDMGIDAQVLDQMSLSIAHLVGLGVQVGIVVGGGNLYRGSQLQKDGLVGRVTGDQMGMLATVMNGLAMRDALVRRNIRTRLMSALPIGTVVESYSSRDAIRHLSQGEVCVFVAGTGNPFFTTDTAACLRGIEIEANLILKATKVDGVYNKDPSKYEDAVKYDHLTFDQVLDEKLGVMDLTAICLCRDHNVPLQVFDMNKSGALLSVVMGEKEGTRVTK

ST25 4190 cg83_0982

MKTPKVGFVSLGCPKALVDSERILTQLKTEGYQVASDYDGADLVVVNTCGFIESAVQESLDAIGEAMSENGRVIVTGCLGKDEDKIRQMHPNVLKVTGAAAYQDVMEAVHEYVPAPPKHNPFIDLVPEQGIRLTPKHYAYLKISEGCNHRCTFCIIPSMRGDLVSRPVGSVLEEAAALKRAGVKEILVISQDTSAYGVDTKYKLDFWNGQPVKTKFFDMCEALGQLGIWVRLHYVYPYPHVDAVIDLMAQGKILPYLDIPFQHASPRVLKLMKRPAHSENTLEKIKLWREKCPDLVIRSTFVVGFPGETEEDFQILLDWLVEAQLDRVGCFTYSPVEGATANDLPDHVPEEIKQERYERFMQVQQQISAAKLQKRIGQTMTVLVDSLEDEYPVAVARSYADAPEIDGNVFVEDIDKSTIQPGDMLEVEITDADEYDLFAKLIKIKSV

ST25 4190 cg83_0983

MIQLGAILSFGANIGCILVHYTKSGVSTYA

ST25 4190 cg83_0984

MDQHTPIDYRLLVDNLTTAILLIDSNFNIFYLNSACEALFDISLLRASGQPVLNLLHAPDDTFNTHEALLNTLKTGQPYTRREAIINVNFKDLHVDYTVSQLNAGKSYHPLLLIELNPIDRMLKISKEENLIQQHQVARQLVRGVAHEIKNPLAGIRGATQLLARSLNDESYAEFTDIIINEVDRLTNLADTMLGSRQLPSYENVNVHEPLERVRSLIANQTKKKIKITRDYDLSLPDVKADRDQLIQVMLNISVNAIQAITENKSFFTDQEPELILRTRIQRLVTINGVLNRSAVRVDIEDNGPGIPESILESVFYPLVTGRAKGTGLGLSIAQNIMHQHNGMIECQSVPGKTMFSLYLPWESDRVAK

ST25 4190 cg83_0985

MRWVLEKTFKEEGFDVTNFEEAQTALERLHHDAPDVILTDIRMPGIDGLTFLSKVKNSHPDLPVIIMTAHSDLESAVSSYQTGAFEYLPKPFDIDEALALVNRAILHINKLQQQEATKTASPLQSTEIIGESPAMQEVFRAIGRLSQSHITVLINGESGTGKELVAHALHKHSPRRAKPFIALNMAAIPKDLIETELFGHEKGAFTGANTQHQGRFEQANGGTLFLDEIGDMPFETQTRLLRVLADGEFYRVGGHIPVKVDVRIVAATHQDLEKLVNEGRFREDLYHRLNVIRIHIPKLAHRSEDIPMLAQHFLARAGKELGVSPKILRTETTDYMQQLPWPGNVRQLENTCRWLTVMITGREVYPEDLPSELKQVPLQKSSETSQPAPSFERISLHHWDELLSQWAIQKLKNGEMKILDIATPMFERTLINAALQQTRGRKRHAAELLGWGRNTLTRKLKELGMDSADDDDEDEHKATLSEA

ST25 4190 cg83_0986

MHDPVLESHHLVCEKPQTRRGIERRLALLLSATELFLEKGYDAVSLDDIVNHAGGSKTSIYKYFGNKDGLFTAICDYRREMFFKDICIAFQPEQTSLKDYLIQTLIRFYKHIIQPEHIAFLRLVIEQTQCNATLSQYLYEKCALDVQNTIAQALLISHQSGEITCTSPDHSSLMYFGILRDIEWRMIMGMPLPPNETEVIDYINYCVDIFLKGHHKV

ST25 4190 cg83_0987

MALRHFLTLRDLSTLELNRILERASELKKMQQSNKVYQPFVGKVLGMIFEKSSTRTRISFEAGINQFGGSAIFLSPRDTQLGRGEPIEDSARVISSMLDIVMIRTFGHDIVERFASYSKVPVINGLTDDHHPCQLLADLQTYIEHRGSIEGKTVAWIGDGNNMCNSYIEAAHMMGFKLKIASPKGYEPKPEFLAEFGHCVELFDNAEDAAVNADLIVTDVWASMGQEEEQKLREKAFADFQVNEKLMGLAHPDCLFMHCLPAHRGEEISETMLDHKNAVVWDEAENRLHAQKALMEFLLNENLKKA

ST25 4190 cg83_0988

MTTLEQSLSEIPAFSIIHEHLFSSAQPSIEQLKLIKEYGCSTVINLALSNSPDSIKNEDQVCLEYGLNYIHIPIDWETPSSDQCLLVLDLIDHLVQNEIAWIHCAKNKRVSCLMYVYRQFYMNMDMPTCQDLLHEIWEPNETWTGLIHSITLQLQGRKATLELQQSLQQTNDLV

ST25 4190 cg83_0989

MVAQIRIGQGMDVHAFEEGNFVTLAGVQIPHTHGLKAHSDGDVVLHALCDALLGALALGDIGQHFPDTDPEFKGADSRVLLKHVYQLILDRGYHLNNADITVACERPKLAKYNLEMRQSIADVLNVDLNQISIKATTTEKLGFTGRQEGILATATVLISH

ST25 4190 cg83_0990

MNQLKRLSFVVTLSCLCSSLVFAESIDCSNSKAELKICSKTFSEARKQLNNKYLSAYLVTDAPLQLLQDTQTLWLRQTQQCKTNSCIQQQFDVRGDDLNFYASLKQTLTQHYLKFENGHIASQPVHLQIHQLAKDKIKIEGLAYRNPNNRKETQTVSLMAYSSPEQKSEILDNEHNCKYKFDFQKALLIVKTQQKGCERFTGIYRLYD

ST25 4190 cg83_0991

MPHVIVIGAGITGVTSAYELSQLGYQVTVIDRHLYPAMETSFANGGQLSACNAEVWNQKATVIKGFKWMRQKDAPLLLNPSFSLHKYSWLVEFLSHIKNYEANTIETVRLALLARKRLFDVAEKEQLQFDLEKRGILHMYHTKDDYDIAKRVNDVLNKGTLERYSVSPEEMKSIEPSLTGEYFGGYYTPSDATGDIHKYSTSLAEKTKQYGVQYKFGLEVTDIKCHTDKVVLNCQPSAEHPHLSQTDSFQLEGDVLVVCGGVGSYQLADMIGERVNVYPVKGYSITVQLKDEKSIKNAPWVSLLDESAKIVTSRLGPDRLRVAGTAEFNGYNRDIRADRIQPLVNWVNRNFDISTEHVVPWAGLRPMMPNMLPVVKQSKQSLVFYNTGHGHLGWTLSAATAVLVSQDIQQKYPA

ST25 4190 cg83_0992

MLSIYLTDTQQHVQFNDYPSDQPVKFLLNLKKIFPSTGDLLLPVLPEDNDLENVTWESTSKDFEIFKKLLAGWGVIELRLNAITAYKDKNFANELIKQAQAKRKKVAQKNHQLSLVALDYIFMHEIHALIDAELFTIGEKFYLPTLREQWKGTVSDQVLNGKL

ST25 4190 cg83_0993

MNRLQLILLVLSTATFSPFVFSETTLSPTKYEFYGQESKRHKSMLEVNNSSDHKINESQHNPFRSDSNKQNVDFFEIAKHYQEEQPQNTYSHSPDHTVNMSDHERSLYYIRNHDNMGLQRDVRYQQYRSLKQQYDKGVISRDEYKNKVYKISR

ST25 4190 cg83_0994

MKIRASRTYSTYSNNYFISKEYECSVIPVKGMCFTDLGLTENGVIQPVEINEVTIDPASNSYHILLAKDSHEYTKEELKRKFEEMKANGWEYIEDLLV

ST25 4190 cg83_0995

LPFVTAPNKFEALAGRDAAVFASGALKTLAVSLNKIANDIRWLASGPRCGFGEIRIPENEPGSSIMPGKVNPTQSEAMTMVVAQVLGNDTTINVAGASGNFELNVFMPVIAYNLLQSIQLLGDACNSFNDHCAVGIEPNREKIDHFLHNSLMLVTALNPVIGYENSAKVAKTAYKENKTLKQVAVELGLVTAEQFDEVVKPEKMVSPNSK

ST25 4190 cg83_0996

MQTRIEHDTMGEIEVPNEALWGAQTQRSLQNFKIGQERLPRAMIRAMGLVKKAAAITNAELGQLPQDLSQYIVGAAEEVIAGKWDSQFPLVVWQTGSGTQSNMNCNEVIANIANQKLGQALGAQKPVHPNDHVNRAQSTNDSFPTAIHVAASLQINELLIPAVEQLKATLQKKSDEFQDIVKIGRTHLQDATPLTLGQEFSGYVSQLEHGLVRLQQALSGLYELPLGGTAVGTGFKCSS

ST25 4190 cg83_0997

MILVTGGLGFIGSHIALSLMAQGQEVVLVDNLANSTLQTLERLEYISGMYVPFVKLDVRNTPALNKVFEQYSIDAVIHTAGFKSIEESNLKPLEYYNDNVSCIMSLLRAMQRTGVRHFIHLSSLAVYGKSGSQLSETDDFNYAYPNPYIKSQQMIEEIIRDTYKIDHEWKIAILRLSNIVGAFEHGVLGEYVAQLPKNIVPLAMQVAAMQRDLIELQDQSSTEDHTVERSFLHVLDVCEAVSSTLHWLRDQTHCCEAFNIAHEQVHSIRQLLDEISQVTQAEIPTQSAIYKHEELAQIGANIGKAKTLLQWAPKRTLKQMIEDEWRFYQNTLNGR

ST25 4190 cg83_0998

MSTLLLITSAPTSIHAWHALGLAQALKSKNEDFRVFFYQDGVQVANDFQWVPDDQRNLTHEWQKLAIRLPVCVSAALARGITDAENASRHQLSHHNLANNFELVGLGELADAVQSASRLLQF

ST25 4190 cg83_0999

VKTVLVILTQANLTSLQVNESLAATMVLATFGSPVKVLLKDAALSLLQNEGNFDQLQHAFKIASNMVDSFEFYDLSPILIEAKNKQHPFVQNSEQELEFVQLDTELIQSFDHVLYW

ST25 4190 cg83_1000

VQASYHHTPRIIEELAKLFHKDDQIVFMGDSTAQLSVNICQQFGSVSCLSHEKDLIDAETLAHVKVLNYDQFADLVLQFNRCISLK

ST25 4190 cg83_1001

MNLELDQDGHLVDYTIWNPEVAQELAKSLDLELTDWHYEVLAAVRQFYQQFGHSPATRPLIKFLMKTVSPDINNAVLQQKFNTGLVARHLSRLAGIPKPANCL

ST25 4190 cg83_1002

MQKFMNGWVNGFIGVAIFAGSLPATRVAVTGFEPGFLTAARAVIAGVLGLILIFLLKEKKPAKQDWWPLAIVALGVVVGFPLFTALALQYMNAAHSIVFVSLLPLATAIFAVVRGGEKPNLFFWIFAILGSLVVFTYMFFLSGEAFFGIGDLYMLIAIILCGFGYAEGGVLSKKIGGWQVICWALILALPIMLLATLFYMPVSFQDVSTSAVAGLVYVSLFSMLIGFFFWYKGLAQGGIAAISQLQLLQPLMGLAIAALLLHEHVSWSMLMVTAVTILCVAAAKKFA

ST25 4190 cg83_1003

MYKQTVQLEINSVNQTKTKIELVISQIEQQIKNRSLTPGTRLPSVRKLAKDLGFSVSTIVEAYERLIALGKIESRSGSGFYVVAPLAPLALSELGPKLDRSIDPLWISRQSLEAEPNAFKPGCGWLPNDWMPLESIRKALRSATRSEDDSLLGYSSPLGLPALRDLLARRAQSKGIEANLNQVLLTDSGTQAIDLVCRFLLKPDDVVLIDDPCYFNFHALLKVHQVKVVGIPYTPNGPDLEAFKEAIESYNPRLYITNSGIHNPTGATLSLSTAHQLLKLIDQSNLIVIEDDIFSDFEYTPAPRLAALDNLSRVIFIGSFSKTLSASIRCGYIIAKPEWIDQITDLKIATSFSHNGLSAKILHTALTDGSYRKHLDLLKVRLAQAMQETIAKLKSIGIEPWIEPKAGIFVWCRLPEGVDAAKIARFCLNRQVILAPGNAFSQAQSAGQFIRFNITQSNHDYIYKTLAEALLQESLEKQV

ST25 4190 cg83_1004

MKWDDIGDQPCSVARMLSVIGDRWTMLILRNAFMGIRRFDDFQKSLGVTRHVLSDRLKRLVEYEILVKAPYFDRQERFEYRLTDKGFELYPIILSMANWADKWMDQGLGKPLEYRHKACGHKFEPVMVCSVCREPLHAKQVQVSAGPGYFAYMEQKQKQA

ST25 4190 cg83_1005

MSGCGAERGLISIDEALDLVINKPKNLSSIQKTLTNSLSSYLAKEIYSEINLPSFSQSAVDGYALCSHTEDLNNQKFQVTGEIRAGSESHDILGEGQAIRIFTGGKIPEGTTHVARQEIVSVISAQEICLTEHIRPQADIRFTGEEIQQGQLLAQVGQFLNIGSLAALSMAGVQTLEVYRAPKVAVLITGDEVAQTAKDLIDGKVFDANGPLLKAWFEDYGLDVELIHVADEADQVTHYFNQLKDSYDVVITTGGVSVGDYDFVRPCAFEVGFEQIFWKVKQKPGKPLFFAEYHNSQTHHSCYLLGLPGNPAAVYVAMQVYGKSLLDALQGNRHGPEWFTAILEHDLKEDARERFLRMYAYFDAGQLKVKSLAKQQSHMLSNLMQANSLVRIPASTKLASGTVLKGIFISN

ST25 4190 cg83_1006

MKPESYRVAEAQAILYAPPHCIELLRQGNTEKGDALKTARVAGILAAKRTDELIPLCHPLPIYRADVEYELEHDFVKIIAVVETIGPTGVEMEALTAASLAGLTIYDMLKPHCEPEELWMDQCKLLKKKGGKSHFKRVLRQPVSAAVIVLSDTVAAGRKPDTAGKSVVETLTEAGFDPIHYQILPDEADDLKELVLELTKSYACIMTVGGTGIGKRDITVDTLEPLLERKLDGLMEAARSFGQKRTPYAAMSRGVAGFIDRSLVVTLPGSRGGASESMAAILPALVHIFDVCRDLPHPGGYE

ST25 4190 cg83_1007

MKEFARIQEQALSLDTFDPIQSFPECGGIDIFMGTVRNHHEGKAVKALKYTSYKPLAEKMIHEIELEIEKKYQVSYVRVVHRIGYLDVGETAIIAIAYAAHRREAFQACEEAVERVKHEVPVFKEEFFTDGTSHYVEGCCIRKDAPHEHKHHHHHAGHEHSH

ST25 4190 cg84_1008

MSIIQEFKEFAIKGNMMDLAIGVIIGGAFGKIVDSLVKDIIMPLITVITGGGVDFSQKFIVLGANPNNLQSLDALQKAGINVLTYGNFLTILINFLILAWVVFLMVKLLNKLRRDKNEPEAPAATPEDIQLLREIRDELKKQA

ST25 4190 cg84_1009

MSDIKNLRNIAIIAHVDHGKTTLVDKLLQQSGALGDRAGEIERVMDSNALESERGITILAKNTAITWLDKRTDTTYRINIVDTPGHADFGGEVERVMSMVDCVLLLVDSQEGPMPQTRFVTQKAFARGLKPIVIINKVDKPSARPDWVIDQVFDLFDNLGATDEQLDFPIVYASGLRGVAGPAPEELAEDMTPLFETIVDIVEPPAVDVDGPFQMQISSLDYNSFVGVIGVGRIQRGSVKLNTPVTVIDKEGNTRNGRILKIMGYHGLERIDVDSASAGDIVCITGIDALNISDTICDPKNVEALPPLSVDEPTVSMTFQVNNSPFAGKEGKFVTSRNIRERLDRELIHNVALRVEDTDSPDRFKVSGRGELHLSVLIENMRREGFELGVSRPQVIIKEIDGEKQEPYENVTFDVEEQHQGAVMEQMGHRKGEMTNMEVDGKGRIRIEATVPSRGLIGFRSEFLTMTSGTGIMTSSFSHYGPLKQGTVAKRQNGVLISMVQGTCLGYALFSLQDRGRLFAKPQLEVYEGMIVGINSRSDDMVVNPTKAKQLTNVRASGTDDALTLTPAIEFTLEQALEFIEDDELVEVTPKSIRIRKRYLTENERKRNRDK

ST25 4190 cg84_1010

MSNQPSQDRLDLVYGLDDRPKPFVAFLAAFQHLLAIIVPIVTPGLLICLALGVPRNETNMILSMSLVISGIATFLQCKKVGPFGAGLLIVQGTSFNFIGPIIGIGSAMVAAGTPVNQVMAAIFGVVIAGSFIEMGVSQILPWVKKLITPLVTGIVVLLIGLTLIKEGLISMGGGYQAMQDHTFASADNLIMSCTVLAIIIVLNRIRIVWIKSSAILIALVIGYILAGFMGYLDFSGLKDAPVIQIPTPMHFGLSFSWGLFIPMAFIYLVTSLEAIGDVTATSKLSNQPVDGPQWMQRIKGGVLVNGANSLLAGLFNTFPSSVFAQNNGVIQLTGVASRYVGIWIAALLILLGLFPAVAGVIQAVPQAVLGGAVMVMFGAVAASGINILSSIHLDRRALLIIAISLALGLGVAQVPQILEHLPELFKNIFSSGVATGGIAALILNVVLPETHK

ST25 4190 cg84_1011

LLINAPNDALVSQLPTEIDASVWTWNYADYQGFVNAGTPAHFSVEFPSQEFDQAIIFVPKSKELLNYILHVVMSHLKTDQSVFLVGEKKRWC

ST25 4190 cg84_1012

VKKKGGVERAAKQLQNFGKILKLDSARHCQLWHLKIEKIEKIKPLESWLKTYTVQVNEQELTICALPGVFSQTHLDVGTAVLLPYLNQVKSGRIADFGCGAGIISCYLAKANSSNIIHALDIDAFALQSTEMTFSRNGIGSDQLRLQPVTGIADAPTELDAIVSNPPFHQGIHTNYDASEGLCQNAKKHLKASGELWIVANRFLNYPILIEKHFGQCEIKTDLQGFKVLYACA

ST25 4190 cg84_1013

MSETHAPEKLQRKLGARHLNMIAIGGSIGTGLFLASGSTIANAGPGGALLAYSLIGIMIYFLMTSLGELATHTPTSGAFFTYGSRYVEEGFGFALGWNYWYNWAITVAFELVAVQFIMKFWFPDVPGFYWSALFLVIIFMINAMTVKGFGESEFWFSMVKVIAIVAFIIIGIAMIIKIMLTPGVATFGNWTYKEAPFVGGLQAMIGVAMIAGFSFQGTEMVGVAAGESKDPKKTIPLAIKQIFWRILLFYILCIFIIGTLIAYDDPNLLQAAATENIALSPFTLLYEKVGFAFAASLMNAVILTAILSAGNSGMYSSTRMLFDMAQQGRAPKWFAKLDNRGVPMNALYATTFIAAFCFLTTFIGEKQVFNWLLNMSGMCGFIVWLGIAISHYRFRKGYIAQGYKLEDLAYRAKFFPFAPWFAFILCSVIILGQNYQAVLGGKIDWIGLLSTYISLPLFLVIWLGYKWKNKTKLIPYDQMDVKPEQD

ST25 4190 cg84_1014

MGIQKILNEEAQYGVPVKIFTQDIDSESIEQLKKMAQLQFIYSHIAVMPDVHVGKGATVGSVIPTKHAIIPAAVGVDIGCGMNAIRLSLKASQLPDNLSRLRDAIERKVPVGFALHKQVKAKASSIIPLEKRLEPIIKKHPGLVRMLRQFEATWQKQLGTLGGGNHFIELCIDENQDIWVMLHSGSRGLGNVIGTYFIELAKKEAQHRFGHVPDKDLSYFAEGSQSFNDYVEAVEWAQEYAFENRKEMMRLILEAIRPLLPSFQMTKEAINCHHNYVSQETHFGENLFVTRKGAIRAGLDELGIIPGSMGARSYIVKGKANPESFCSCSHGAGRKMSRSKAKVLFNQQDLIEQTQGIECRKDSGVVDEIPSAYKDIDEVIANQADLIEVVHTLKQVLCIKG

ST25 4190 cg84_1015

MKIKEMPKIKVRNQVALSPLLHKGGMHETEKPRAQHRRNRQDTKQQLKKGVW

ST25 4190 cg84_1016

MKLSRIALATMLVAAPLAAANAGVTVTPLLLGYTWQDSEHNNNKLTDHAELQDDLFVGAGLGVELTPWLGFEAEYNQVKGDLDGTGVQGAEYKQKTIAGNFYATSDLITKNYDSKFKPYVLLGAGQTKTEFDGIYEDKKDTIGNAGVGAFYRLNDALSLRTEARGTYDFDEKYWRYTALAGLNVVLGGHLKPAAPVVEVAPVEPTPVAPQPQELTEDLNMELRVFFDTNKSNIKDQYKPEIAKVAEKLSEYPNATARIEGHTDNTGPRKLNERLSLARANSVKSALVNEYNVDASRLSTQGFAWDQPIADNKTKEGRAMNRRVFATITGSRTVVVQPGQQAQ

ST25 4190 cg85_1018

VYIVRTSFIKFHNFLLIFSVCITSNATDYKLEQLDEKTIYSNSEHELNSVYSDIKGYISDNKNEVKYLVDTQRFWLKNRNLKCNFNGKEANSGNYKCLSDFNNSKIKDLKNSYLDLDNLEGKLIKPFRYTNGIMKELETGGCYCSESTIKILKDKIYIYQACDQGLKEPRIYNIVGKKKDSFSVEYQIDTNNKVPEFNLAFVTNGKNVWNIVPKIFRKEDLINLNFGINYTTDMNIKNKKNDCHDDEE

ST25 4190 cg85_1019

MRSKIFISVILFGLSACAAKADQNQLKDRELDKLVACNGGNTEYTNIDGYFTIPDFGCPYDGKNNKIGNAMVYLIAPDHNNDYIQLNNMKNIKGKYIYIVPFKFLNQDKKSGSFYIAKDNYSYEVYFNEGGKWVRKPDIIVSNNKVSVLSEINGFLNDRAKSFSKKTRKKLKKYKMDINHDGVEDFIEVVGKDNKPSSIHILNGLDNSLIYKNDKIF

ST25 4190 cg85_1020

VGHFSAFRGNRQLCDKYGRNDNHPADQVMIANIAYANRNGNGDIQSGDGWKYRGRGIIQITGKDKYDKINKAIKDNYPNVGISIDANNINNIYEGTLASMAYWKSFGLSKLATQKQVDIRTQLEVVDSLIDIINRDTASRSDRKKNFEYITAKVFKLNECKNSIVEANLLSKSTPTPTPTPTPTLSNVLKEIKQLVDRNIPYSQTGARGAGSNKNATSVITANDLKGLDCSETVAIYLLKLGVTDKFYSIHTGVMLTENDFRKAIRSNKIEYVVGSKDLNFIPQIGDIFVWRNGGGHCGIVYDVDRQNDTVTILEAIGDVGSADENFNINNGGEKKVGCTRTAVYRRSGKALAQHRGWVGYFRPIISGKKI

ST25 4190 cg86_1022

MIAMVNDGGLALDRPTAAAIVGLFAGSMYLMTVFGGWVADNWLGQARAVWYGSIIIALGHLSIALTSVFDQSFFYLGLILIVLGTGLFKTCISVIVGTLYKANDARRDAGFSIFYMGINMGSFIAPLITGLLAKDHGWHLGFGIGGIGMLIALLIFRFMAMPQLQTFNELRQEANSCSKPVVENKNAPKIVFSFLFAVAVVIALTFLSIIHINPVAVATYLTVGISIGIIAYFAYLLLFLNLEQHEKFKIIICFVLLAASALFWSAFEQKPTTFTLFAQDYTDRIVFGFEIPTVWFESINALFIIIFAPVAAWLWAKLGKANKDPSYISKFIIALLFAAGGFLLMSLASHFAIHGGVVSPFWLVGTLFLLTIGELCLSPIGLSTMTKLAPDVIRSQIMGLWFTGTALGNLMAGLIGGQVSADGINHLPSLFMRCVLALVIGAIVLFLLKKPMNKLMDKSTNKVQPDLETI

ST25 4190 cg86_1023

MNDLTVPEKLAKLRELMTNQSIDALVVMSADPHMSEYLPDYWKARQWLSGFSGSVGTLVVTQNFAGLWADGRYWVQAEQQLAGTGFQLQKLTSDESSTHLAWIEKNLPAGSVISINGQTLSIQQFKGLENTAKQRGFKLETQQDLIDSIWSNRPELPLEQIHLMPEGLNALSRKEKIQAIREILKTKAIEGHFISSLDDIAWVLNARGQDVEYNPVFLSHLYISAQQAVLFIDSNKVDLTTQQAFKADGIEIRDYQDTAKFLSNISDASVLLDPAKVSIFHEQAIAKDIQVVYDINPSTLFKSRKHESEIAHIRHAMVKDGVALCHFFNWLEKALHQGQRISELTIDEKITAFRAQQEGFIGPSFSTIAGFNANGALPHYRATEEHYSFIEGDGLLLIDSGGQYVDGTTDITRVVPVGTPTEQQKRDYTLVLKCHIALAKTIYPEGLAAPLLDSICRHTLWQYGLDYRHGTGHGVGFALNVHEGPQVLSYYAPIHAYSKLREGMILSNEPGLYHEGQYGIRIENLVANRLHSGFEKTYGDFLEFETLTLCPIHLDCIVVDMLTDEEKDWLNSYHQTVQERLAEHLSGDVLDWLIYNTRKI

ST25 4190 cg86_1024

MLSDSKIRSAKPKEKLYRIGDSDGLCIEIKPNGKKYWRYRFQWLKKTQMMSLGEYPIIGLAEARTKRDEAKSLVASGVNPVEDKEKQKKAKHDEYENRVLFKHVAAEYKAEKLNNRSERYQEAFQRALDKDILKVIGDKDIKEVTSADVLTIMKKTIARVKRQKNHGTGEVSAIQNRTFIGGVMRYAIATLRAEYDPTYAVKNVVERPEIEHARPMEKHEAVQLRNKLNSYGGSTTVKNAGLVMLYSMLRTIEIRRMKWEYVDFEARTITFPKEMMKKKRIHIVPMSDQVFNILQEQRNIVGNREYVFPAIYQDGMLSATTMNKMLDYIGLSDVTAHDFRATASTLLNEKDYDDKWIEKQLAHADGNKTRATYNHAKYLESRRKMLQDWANIVDSWAV

ST25 4190 cg86_1025

MSSQLIKIHYHAYSRVADLLADLDKKGEVTKIYDLNGNELKINFLRDEVYYKKVWWHFQKKQGG

ST25 4190 cg86_1026

MNIEQYLDELIKREGGYVNNPADRGGATKYGITEAVARTNGFKGNMKDLPLDVAKAIYKKQYWTAPRFDQVNAVSSAVAEELLDTGVNCGTGFAKPLLQRALNLLNNQGKAGYADLEVDGVYGSATLGALKTYLSKRGKEGEKVLVRVLNIMQGQRYIEICERNPKQEQFFYGWIANRIG

ST25 4190 cg86_1027

MNVISLLSHVQLTETKQQKVNELVAQCVLSACAKTPSMREVLKGDMRSTIHASRLRKVS

ST25 4190 cg86_1028

LNIISFDEELLKTTARHWIDRGIALNLDDELIELNEQFFEHIQASKDYGDYLTRESLNTYIGICEDDCDHPNVIVEVGYHRRGRELTLKIFDIYISPELDSLVDSEYDSKYAEYLIFIIQKFLQHADCSGSATKIYARTDYSQAFLQRMHDAAESIKSELDKAGLTVKFEGKRWLAFRRQ

ST25 4190 cg86_1029

MGAFLLPNSGVKGMEPVSTSGFTALLKFYGVAIVVALAVGLVAAVVLMTRMPRSPQEWAVGLICTVVSSLAGGSFIIVKWGLHEWVTDIWGMIALGGFFFVCGLPGWALVRWIFNFINKQEGKTIVEVIKEFKKARKDIENS

ST25 4190 cg86_1030

MGGIFGSTTISTSDNRINSMRVQQSAYGLCQPLVYGKNRLAANMFWYGDFSSTAHTTTTKSGGKGGKTKTSNTTYTYSASLMLGLCETKIRDIGNIWRDKEQIVPKTEGGVQLKPIDQLGFELFDGDHNPVWGYLASMHPDQAVHYPFLGYIACANYDLGGSASLSNHNFEVISDITFSDTIHDANPADVVEDLISHPRYGAAPNLNVADLSEFRRYCTATGLFISPALTEQRAAHEIVNEIVEAVNCAIVPSPDGLKIRSYGDTAVSGNGVTFTPDLTPAYHLTDDDFIGDDQPVRVKRSRDTDAFNHCQIEYVNRFNQYNTETVEAKDQANIEMFGLRTQDPVKYDFFCEPKIARHAVQLLLQRKLYVRNEYEFDLGWKYCRLEPMDIVTLTDESLGLDRFPVRITRIEEDQDGLLTVTAEELALGSRSAVEYDLQASNGYQGGNEEPGNVNAPVIFEPPLDLTDGKNQVWVAASGGSNWGGCNVWASLDNTTYEMIGTIYGSARYGQLVAAINSSATAMQVQLNTSSQIFSGTSEDAQVNTTLCRVGDEYVSYVDATLNGSGLYTLGGVLRGRFDDALAHNTGESFVRIDKAIFQHEFNSNLINKTLYLKFTSFNGLQQKEQTLDEVTAYSHTLNGGRPSGVKGLSLQSPFVGSSFKVQWQFAAGAQGYIVQVSSGSNLLRTIETTSAEYTYSMEEARVDGVQRNYTIRVASKSENGTSTFTDLNISNPVPPILANVYTSATSNSITVTWIPSEVPDLKDYQVWISKNASFDPETLAASWTGTENACTIENLDSTTTYYIRVAARDVWKPTSWNYSARVTQATLEA

ST25 4190 cg86_1031

MQKNQLAVQEALTWLGTPYHHQGRVKGVGVDCGTLICEVYEKVGLMDHLDPRPYPPDWHMHQMGERYLEHIRSVCFEVDGPPEPGDIVLYKIGKCVSHGAIVVEWPTIIHSYIHLGVILQDGTKGSLARRIAGFFRMKRLKK

ST25 4190 cg86_1032

MTDYTITDGQFYKVLDKDTGAVITMGELSDTNTLSTIHNVEFISEEQYEAERPKPEALSETKMI

ST25 4190 cg86_1033

MSNTLLLKTDGSINTRSKVFPKLGKCNIAIYRRTNGADEVELWTWGLSHAENPITINGGKFVAWAANVPDRLVVPENPIAIDTTELTLPRQIVCYFKIKIDDGLKSCQLMFNNYLTDTSLCGGNKPDQMKIDYSRRPYTINSMAKMFWNQFVAYDLSGWDMSDVVYAVSMFHNSPNFNQDLSLWDVRKITEFSSMFQGTPFNQPLNSWVTESAVAFAGMFANCVDFNQPLDKWNTSKVSDFSSMFGWAKSFNQPIGNWDTSSATNILYMFENAHAFNQDLNDWNVQKVEYMDGLFKYAKSFNQPLSNWDTRSVVTASQMFMGAESFNQTIENLNFSKCTALRMFMHQAKSFNKPVAALDVSVCTDLAQFFEEALSFNQPVESWNVSACLDMWRMFAYATAFDQPLAAWCPKFNVEVSLDSFMEGKAYRTSYYDDFLNALWLDVNTTRRNQWASRIKPRLLGMGLSKYSSVSSSARANLVSAGWTITDGGQV

ST25 4190 cg86_1034

MTVPSDYDFIGNTITESQFKNALTVLLNHIRQMSLDLVEAQGGNYSYATMALFDADKINVPANSTVRIARGDDAGLYVWDGANLTKVETSNNPYTISSTPDDLFIISDALDNVLFSIGRDGTVRGSFDLSNIDLNIESTSEVGGDTVLAVSDNTGNMLASLNSKGEWYFTKIIADEVVTPFGSSSESSDEVIEQTEIAIPELSFYRIDFTMVGQPPTDLGETTVSGVCSFSDPSNSQTFFKSNMEVTVQGQGSAYDYKKNYTLDLFNSDMESLKVKVGSMIATDSFHLKGFYRDPTHFRDQGGYRFWNSLVRKLDYPYCKVNNIIYQANTDRKADAEYTADAKYYPHGIPCVVYLNNQFYGLYTLRLKKTRQNYALNNADLNHIFLDSATYDAHLSQSFDPHDWEIKSPKMSGYEDQGPVPSKFAAVQTSIERLFNFTKDLDSNYQNHASVLVLPHWLIFYIFAELVGHWDINGNNYNIMTWDNIHWSILPYDLDWTLNWFTGENAGATQTGFIVSGDIWPRFRQVYLPQIRELYTKLRKSGDISTYAVVKHYVEVARNIPRDIYSKDKAKWGVTPIFGNSNYPDLEQAYRYIDARINYLDTVWLIN

ST25 4190 cg86_1035

MRAASEKLIALLDANQFVMADLYTITTIQNDIYRYTNYDFDLIVAGELYHSDGPIISRDGITLSLGVEVDNLSITIDVTDKQTFESLRIVQAFHNGQMDGARFKLERIFMDANTPTDTSAGTIKLFEGRIIEPEFDRNTIQASVASDLDELNVQMPRNLYQPSCSNTLFDHACGLNRENYALETTIAAGSTASRILCDINQPQGWFTQGVIEFLEGGNKGLKRTIRLHELDVLLLTLPLLENPEVGQRIKVYPGCDKRQETCQNRFNNFSRFRGAPFIPIPETSV

ST25 4190 cg87_1037

MGGAIGTGLFLGSAQVIQSAGPSIILGYAIGGLIAFLIMRHLGEMIVEEPVAGSFSHFAYKYWGKFPGFLAGWNYWMLYVLVAMSELTAVAKYINYWWPHIPAWTSVLFFFVVITAINLTNVKFYGESEFWLAIIKVAAVISMIVFGLYLLFTADVGSSISFSNLWSHGGFFPNGFSGLFYMLAFLMFAFGGIELIGMAAAEAKDTEKNHSKSN

ST25 4190 cg87_1038

LIFYIGSLAILLSLVPWNQLDLGGLDKSPFVMIFSQMGIGWAAHLLNFIILTAALSVYNSGMFANSRMLYGLAQQGNAPKIFKKVNKQGVPVPAVLLSALLIFGCVLLNYFVPEDALSHLMYIVVGALVLNWAMISMTHLKFKSAMKKLGQKTHFPALWAPFSNYLVLGFIAVVLYIMWSQGFKESVMMIPIWIILMFILFKVLNAKEQ

ST25 4190 cg87_1040

MVTMNTPASNDKNPTPDLAEDNAFFPSPYSLSQYTSPKTDYDGTTYPTPYAGNKKVLMIATDERYIQMQNGKFFSTGNHPVEMLLPMFHLDNAGFEIDVATLSGNPAKLEMWAMPKQEQVVLDTFQKYADKLKNPLKLADILENVVGENSSYAAVFIPGGHGVLAKIPHSLEVKKVLKWAVEQDKFIITLCHGPASLLAAAVDEQPENYIFKDYQICVFPDSLDKGANIDIGYMPGALPWLVGENLEKLGVKILNTGITGQCHRDRKLLTGDSPLASNNLGKLAAETLLAEVKD

ST25 4190 cg87_1041

MKIVEITVPGGPEVLKLQDSNVPVPQADEVLIEVKAAGINRPDVLQRMGLYPMPKGVTQIPGLEVAGVVVAVGEQVQQFKVGDKVCALTNGGGYAEYCAVTATQVLPIPENLSFTQAAAIPETFFTVWANLFDIGRLKKDETALIHGGASGIGTTALAICHALGIKTFATVGSEDKVEALSDLTTAINYKTQDFEQEILNHTQEQGVDVILDIVGGSYFQKNLNLLKRDGRLVIIGFMGGRIAKEFDLQELILKRATITGSTMRARNSQEKAQIAQSLHEHVWPLLAQGKCLPQIYKTYAFSDVQSAHACMEQGDHIGKIVLEMNA

ST25 4190 cg87_1042

LQEPSIVLVIQGVKRAYIGKDIFKFQQGQCLFISIAIPFDCDTLVEREPMLAIAIKFEPQMMAELATKMDKEQRPLIEDLDNKEIKLSCGLNIIEMNAVISDVALRLLNLLRSKQDTAILGEQVKRELIYRVLQAGGSDFIQNLSAIMSRSGVIYTICEIIQRDYYRNLTVQELAKQAGMSVSLFHQAFKKVTNYSPLQYIKITRLHKARDLILNNQMGVAEAAYEVGYVSASQFSREFKRLFGVPPKLSTN

ST25 4190 cg88_1043

MESWQEDLLSAFLVVKNEYQLFEIVKSTASRLGFDYCAYGMQSPLSIAEPKTIMLNNYPEAWQKRYVERQYVKIDPTVQHCMVSLQPLVWSSQSAKTQAEKDFWEEARSYGLNVGWAQSSRDFIGTRGMLTLARSNDQLSEKEQKAQYTNMYWLTQTVHSSIAKIVNDVEFAKFNLYLTNREKEALRWTAEGKTSAEIAQILGVTERTVNFHLSNSMQKLNVNNKISAAIRAVMLGLL

ST25 4190 cg88_1044

MVFTEWATSQTPALSTGWDWELIENNGITTVKRVGLPRSNIMIVDVSGMDIGFDINETLLEKKIDTLFWEPFIYAQINTSLTESSLSQTFS

ST25 4190 cg88_1045

VEALVMNIIAGFQNNFSEGLYTKFKSYRYRVFVEYLGWELNCPNNEELDQFDKVDTAYVVAQDRESNIIGCARLLPTTQPYLLGEIFPQLLNGMPIPCSPEIWELSRFSAVDFSNPPSSTSQAVSSPVSIAILQQAINFAREQGAKQLITTSPLGVERLLRAAGFRAHRAGPPMTIDGYSMFACLIDI

ST25 4190 cg88_1046

MVGTTIEWYDFFVYGAAAALIFNKLFFPNLDPLTGVLAAFATYAVGFIGRPLGGLVFGHFGDKIGRKSMLLLTLMLMGIPTVLIGLLPTYESIGYWAAIGLVILRFIQGMAMGGEWGGAVLMAVEHAPEGGKGFWGSLPQASTGGGLMLASIALGLVSLLPEQALFSWGWRLPFLASIILLAVGWYIRVKVPESPDFEKVKQQSEEVKVPAFQVFKNHPKQLITIILARAAENAWFYIASTFTLAYTTTQLGIPRQDILFATICGAAVILFMTPLCGHLSDKVGQRNMFMFGLCVLALYSYPFFSMLNTKDPVLVWTAIVLAIGVVFPIMYAPQAQLFARQFPAEIRYSGISISVQLAGVLGGGLAPMIATKLLSIGQGNPYLIMFYIGSMAVVAIISTSFMPRDWSYKKDETYYKKANLNLKTKID

ST25 4190 cg88_1047

MNTENHLMIERQGKLGVITLDRVTHLNALSLDMIEGIGAQLELWRNDAAVQAILIKSNSPKAFCAGGDIRYLYDSYKNGTADYKGYFSAEYKMLNTLREYEKPIIVVLDGYVLGGGFGLAQACHIVVSSEKSRFAMPETAIGFFPDVGATHFLSRLDDIGVYMAITGEQISSSDALYLDLIDYHVPSEQLQALQDALVEAPSLSKEGIEHIITRFITRPAESELKQLAEGIRKHFGYQHLDEIEQSLENEKDESLKTWASKMLSILQQRSFIAKQTSLKLQHLGRGLSLQQCMQLERDLQDIWFEHGDFIEGVRALIVDKDKQPRWQERNPELEQILEKLS

ST25 4190 cg88_1048

MQWQSILLEKRNGVGLITLNRPQALNALNSELISEINQALDQLEKDREIGCIVLAGSEKAFAAGADIKEMADLAFPDIYLDDFFHLADRIAQRRKPLIAAVSGYALGGGCELALMCDFIYCADNAKFGLPEVTLGVIPGIGGTQRLTHAIGKAKAMEMCLTARQMGAVEAEQSGLVARVFSKEELLEQTLQAAEKIAARSLTANMMLKETINRAFEVNLTEGLRFERRMFHSIFATADQKEGMQAFVEKRQANFKNQ

ST25 4190 cg88_1049

MQFTEEQLLIRDMAKSFAQEQIKPNASDWDRDGTFPKETLTQMGQLGFMGMLVSEEWGGSDTGNLAYVLALEEVAAADGATSTIMSVHNSVGCVPILKFGTDEQKERFLKPLAQGEMIGAFALTEPHTGSDAAAIKTRAVKDGDDYILNGAKQFITSGNNAGVIIVFAVTDPSAGKKGISAFLVPRETPGYEVIRVEEKLGLHASDTCQIALTDVRIHKSLMLGKEGEGLKIALANLEGGRIGIAAQAVGLARAALEEATRYAKERITFGKPIFEHQTIAFRLASMATEIEAARQLVHYAARLKEAGQPCLNEASMAKLFASEMTERVCSSALQVFGGYGYLRDFPIERIYRDARICQIYEGTSDIQRLVIARSL

ST25 4190 cg88_1050

MMDYQNAVQAFDLESTAKQMLSGSLDALNACYECCDRHADGDKIALYWQGKDGRKEQYTFRELKEWSSQFANFLKSQGVKAGDRISGLLPRTPELIVTILAAWRIGAVYQPLFTAFGPKAIEHRIQLAQSKLVVTDMGNRSKLDEIEKCPAIMTVADAQGTPLKAGDFNFWNEVKQQSDQCDLVMRNIQDPFLLMFTSGTTGPAKPLEVPLKALIAFGRYMQDAIGLTEEDSFWNIADPGWAYGLYYAITGPLFLGHATLFYEGGFSTDSLCQIVKDYKVNNLAGAPTAYRMMMAADPAQMAPLKGQFRVVNSAGEPLNPEVIRWFKQVLDTPIYDHYGQTEVGMVVCNHHGLKHEIHAGSAGFPSPGYRVAIVNEQGEELPPDTPGILAVDISQSPMMWFGGYKESRKSPFVGYYYLTGDTAELHADGSMSFVGRSDDVITTSGYRIGPFDVESALLEHDAVIEAAVIGVPDPDRTEVVKAFVILAAGVQPSDALAEELSQFVKRRLSAHAYPRLVEFVSELPKTPSGKIQRFLLRNQEIAKQQAKAG

ST25 4190 cg88_1051

MNIAFIGLGNMGGRMAQNLLKAGLKVYGYDLSEVAIQHFAEAGGVVCDSPQDAAKQADVVITMLPTAKHVKEVYLGENGVLEVLKAGSLCIDSSTIDPQTIKDIAAVAHSKNIKICDAPVSGGTIGAQAGTLTFMVGADEQTFNEVKPVLSHMGKNIVHCGDVGAGQIAKICNNLILGISMAAVAEGMALGVKLGIDPQALAGVINTSSGRCWSSDVCNPWPHINENAPASRGYQDGFATQLMLKDLGLAVEAAGQVKQPVLLGGMVQQLYQQMCMRGNAHLDFSSIIQQYLPQES

ST25 4190 cg88_1052

VGATIFSGVNTDMRIYKEEIFGPVLSIICVDTLDEAIALINANPFGNGVGLFTQSGAIARTFQNLIDIGQVGINIPIPVPVPFFSFTGSRGSKLGDLGPYGKQAVQFYTQTKTITSRWFEDSHEVSGVNTTISLR

ST25 4190 cg88_1053

MNTIQKIELDTAKLLINGQFIESKTQEWQDIVNPATQEVIGRVPFATVEEVDAAIQAAQDAFASWRQTPIQARMRIMLKLQDLIRANMKEIAQVLTAEQGKTLADAEGDIQRGLEVVEHACSVGTLQMGEYVEGVARGVDTYTLQQPLGVCAGITPFNFPAMIPLWMFPMAIVCGNTFVLKPSEQDPLSTMMLVELAIQAGIPAGVLNVVHGGKEVVDRLCTHKDIKAISFVGSTAVGTHVYNLAGQHGKRVQAMMGAKNHVVVMPDANKEQTLNALVGAAFGAAGQRCMALSVAVMVGDSKQWIQELVEKAKTLKVNAGHEPNTDIGPVISKRAKARVIDLINSGVEQGAEVTTSMVAMFKYKIMSQVTLWAPRFLVV

ST25 4190 cg88_1054

MKVDWDHLQFFLVLARTKTLTNAARIIGVEHSTVARRIQALELALGTTLFKREATGYELTLEGMALVPRVEQMEQAFLQIEKPHQPLQGRVRIGTPEGFGTAFLARLLAEFSIQYPLLTIDLIPVPKMIKLSHREADIVVSIERPTSGPYIITRLSDYCLKIYGSQNYLAQNPPIRRLEDLTQHRFVNYIDDLVYSPELYCLERLPLKLNANFRSSSILAQQIAVSAGAGLAILPKFLADDKPELEEVLEQQVRFTHTFWMLTFVDLQHEPRIKLVWDYLRKQADKYQHLLVD

ST25 4190 cg88_1055

MVHHSDEASSPDHLQRKLSNRHLQLIAIGGAIGTGLFMGSGKTISLAGPSILVIYMLIGGMFFFLMRALGELLLANLHYKSFVDMAYDLIGPWAGYYIGWTYWLGWVLVGIADLSAVINYLSFWLPEGASFSPMQQAMISAGCVLFVLGLNLLTVKLFGEVEFWFALIKILAIIGLIGVGGYMILTHFQAPHGDVVSISNVWSHGGLFPKGVSGFLAGFQIAVFAFIGVELIGTTAAETKDPQTNLPKAINAIPVRIILFYVLALFVVMSVTPWNHIHADKSPFVELFLNAGIPISAIIMNLVVLSSVMSSMNSGVFSTSRMLFGLSKDGQAPSVLGRLSKRAVPSNGLIFSCIFIMGGAVLQYFVPNTMEAFTLASSLCVILFISVWILIMACYLRYRKIRPELHAASTFKMPGGVLMAYAVIAFFLFTLVILALEPDTLKALYVSPVWLVVLSVTYYAFYKPRMRKLGQEIF

ST25 4190 cg88_1056

MTTARHSDTENSPDHLQRKLSNRHLQLIAIGGAIGTGLFMGSGKTISLAGPSILFIYMIIGGMFFFLMRAMGELLLANLHYKSFVDMAHDLIGPWAGYYLGWTYWLGWVLVGIADLSAVINYLSFWLPDGASFSPIQQAMISAGCVLFVMGLNLLTVRLFGEIEFWFALIKILAIIGLIGVGGYMIFSHFQAPQGAVASISNVWSHGGLFPKGTEGFLAGFQIAVFAFVGVELVGTTAAETKDPQKNLPKAINAIPVRIILFYVLALFIVMSVTPWDHIRADKSPFVELFLNAGIPVSAIIMNLVVLSSVMSSMNSGVFSTSRMLFGLSKDGQAPGAFGRLSKRAVPSNGLIFSCTFIMGGAVLQYFVPNTMEAFTLASSLCVILFISVWSLIMVCYLRYRKLRPELHEKSTFKMPGGIWMSYVVLAFMLFTLVILALEPDTLKALYVSPVWLIILGVTYHVLYKPRMKRLGRELVNDH

ST25 4190 cg88_1057

MSNSDIQKINTNEVMSAVTVFNKVVYLSGQVPKNTEQDVAGQTREILATIDELLALANTDKSRLLSAQLYLKNLSDFSTVNAIWVDWLKGCVAPSRATIQADLVNPDWLIEIAVTAAQK

ST25 4190 cg88_1058

MPRPITAVIHRQALQNNLAVVRKAMPNSKVFAVVKANAYGHGIERVYEAFKAADGFALLDLEEAKRIRALGWTGPILLLEGVFSPQDLFDCVQYQLSFTIHSEAQIEWVEQHPYPAQFDVFLKMNSGMNRLGFKPQHYVQAWERLNNLANVAKITHMMHFSDADGDRFGQQGIDYQITAFEEIVKDLPGERSVSNSAAILRYQDQLKSDYVRSGIMLYGSSPDYPTHSIADWGLQPTMSLRSEIISVQHLEPNESVGYGSNFVAEQPMTIGIVACGYADGYQRISPTGTPVLVDSVRTRTVGRVSMDMLAVDLTGIESAKVGSEVVLWGQSSTGVVLSIDDVAVSSGTVGYELMCAVTARVQFINQV

ST25 4190 cg88_1059

VREGMEMRVIVLGSGVIGVASAYYLARQGAEVTVLDRQSGPAEETSFGNAGQISPGYSTPWAAPGIPFKAVKWMFQHHAPLAINLDGSMWQLQWMAQMLKNCNPQSYAVNKERMMRVAEYSRDCLRELRKDTGIHYENRAKGTLQLFRKEAQMEAVQRDISVLEECGVSYELLNGNELGRVEPALANAQDKLVGGLHLPNDETGDCYLFTNALAQIAKELGVNFQFNQNVEKLIVEGDQIKGVQVNGKVLTADRYVLAFGSYSRDFLKPLDLQLPVYPVKGYSLTIPIVDPAFAPQSTVLDETYKIAITRFDQRIRVGGMAELSGFNLGLNEDRRATLQMVTQDLFPGGDMEQASFWTGLRPMTPDSTPIIGATRFKNLFLNTGHGTLGWTMACGSGKLISDIVLNHKTDISTDGLSIQRYSHAHAA

ST25 4190 cg88_1060

MRPLDRIDRMILDILQREGRIAISELASRVNLSTTPCSERVKRLERDGIIMGYYARLNPAYVDRNLLVFLEIKLSAKSGDVFDQVARDLVEIPEVLECHLISGEFDYLVKARLKEMSAYRRLLGDLLKKLPASASSHSYVVMEEVKETLYLDVSK

ST25 4190 cg88_1061

LITAMSLEKNIERLQSIDALRGLVIIIMLLDHVRETFYLHKQVTDPMDVTVTEPALFGSRLLAHICAPVFVLLTGISAFLFQSKKQDLQQTRAFLLKRGLFLIVLELTLVNFAWTATFPPEVIYLQVIWAIGISMVVLACCVSLPLPVLAGVALVIIFGHNLLDSVYFSQGILQNIWFVLHERGWLEFAGIKLRTSYPVLPWIGVILLGYVLGQFFSSKYSAKQRSRTLLSLGLASVGLFVLLRFINVYGDQQWQHFESLQLSLMSFFNLTKYPPSLLFILLNVGIGLLVLVAFERMQQHSFLKPLVVFGSVPMFFYLLHLYVLKLMYVLALSVWGANYGNYLSVNHVWMLWLITIVLSFALYPAVKWFSKFKHQNKHISILKYF

ST25 4190 cg88_1062

VSQNSSATKFTHDVLDVPFNRAYLSKQIMEQQDVQRIDDALTLVSGVFHQNSFGGGFWDNYSFRGFSTDPNLGAAMIRNGLSVNRGISAPKDVVNIESLEFLKGPMAALYGRGETGGLLNLNSKKPHWESESELNLRANTQEQYRISLEHTAPINDELAYRLAVAHEDNQSFRDHVSSERWFFSPQLTWKISDQTQLDFDSEFTEHKGTFDRGVSTVNHQFVMDPKTFTGEPDDGDLKIKDYFYQLHLSHEFNPDWKLNSAVSYKDAKMVGFATEPRRMQADGRTLERQRRYRDYTSEDVLAQTELLGKIDTSWARHEILLSTELGQLDYKQNQLRRNHSTSSTNTIDIYQPEYGKYLPNLTPFTDTKERQRYFALNVQDQIFFNDQWSVLLGNRFDQVEQDFKNHIKQTEDNQTLHQNSPRFGVNFKASDQWAFYTNYGRSFAMNSGMNRNGQTFAPEKGESYEVGTKYKINDQSVLSLALFKMKKQNVLTTDPIDKDFQTAAGEVSSKGIEFDLNSQINDRWFVNANYGYTDAQIEKDQDLAKGARLSNVPKHQGSVSTNYEFLQDGARKAGVGANLTYVGERSGHNLDNGFNLPSYTLVNLNGYYAPSDRLRYQLNINNLFDKTYYVSSYSDLWVQPGEPLNASISAQWKF

ST25 4190 cg88_1063

MSLSQKEIAVRFLELAAAGEVDEAYGNYTAPNFKHHNPYYAGDKTSLKEGMRESAVETPNKVLEVQHVIEDGALVAVHSKLEMQMNNKLTILAVVHICRFENGKIAEFWDIGQIQPDPLVNENGMF

ST25 4190 cg88_1064

MNRDYEQFPDDDNGNVLWQMVEDGDDLTELHEIEFSIAFQDQQNAEQCAMHLLYQEQKISLFQDDSVEPNEWIITIFVTMEPEYSDIVDLEQWFSTIAEQFNGEYDGWGCMAYVFDEEEDDILQ

ST25 4190 cg88_1065

MPLNDNFVYAPPQDPLSILFEDDDLIVVDKPAGLLSVMGRLPEHHDSAYLRVLEKFPLAKVTHRLDMATSGLLMFAKHRDAEVAVSKMFQARTVKKHYIALVQGQVKQEGSVEVPLITDWENRPRQIVHFELGKHAKTLFQPLVYDATTDQSRVLLEPVTGRSHQLRVHMMHIGHPIMGDKLYHPEPKRFHLNRMALHAAYLAFQHPLKGTDVVIESQVPF

ST25 4190 cg88_1066

MENKNQCRQCAVCGKSFPLKDLVSGEVIRNVISDEILKDYPDWSYSSFICRADLADYRIKYVQSLLRSEKGELSNLENEVIGSMQRHELISRNTESDFDQNWTFGEKLADKIASFGGSWAFLICFALFLTGWIVLNTVVMVVRPVDPYPFILLNLVLSCLAAIQAPIIMMSQNRQEAKDRLRSQHDYQINLKAELEIRHLHEKMDHLLSHQWERLAHIQEIQLDLLAEMNKKR

ST25 4190 cg88_1067

MNKPYLSALPIDSQISSQLNNAYFQDSWSIVLGQPDLSVFEQLIKLFQHTPQWIEWSMNMRNKITSKIGLKDLGSFKQIDTHKKENEYVAGDRIGIFTFLQRTENELVIGDDDKHLNVTLSIYKNEDTQVLTVTTIVHIKNWLGRLYMLPVIPAHRKIVPATLQILG

ST25 4190 cg88_1068

MNNNIIIFGYGTGISKAVAHKFGKEGYKIGLVARNAQKLEKAILELKAQGIEAYAFACDLAVLEDIPNLIKRIKDQLGEIKNIHWNAFHDIEGNILETPPLELTKSFHIRVSSYIATVQACLGDLEKNHGSILSTNGIFAFDAVGIDLVAKEYSSLATTAAAQYKTTNLLAHSLADSNVYVSQVIVNGFVDGTSGAQDKTYTVHPETIAGQFWYLHQHKQETVSFCGEAIQAA

ST25 4190 cg88_1069

MVYFNLQGVDFKFEEQDPDILKQPSEVSGIRSKDLPDLLKEIPLTKKDIYLIIKHDERIKDSVRSILFKEYKIDTSEYEK

ST25 4190 cg89_1070

MKLKHLSTAMILATLPATGVFAAALDRSGQSMSAFFQPGNYFEAGISVLDPDVAGKEAGSSATRRDIGDMANDYYFPSAALKLQINDQFSFGLLYDQPFGADAEYSGNNVFVSQPGSDSVLSATAIDGIVTKKATEALQAQGLPVNDQTLALAKGQVLASPQFKQLAGALAAANNYLGNGGTKVKVDTQNLSFVFGYQPTKNFNFYAGPVLQTVKGNVSLRGQAYSLYNGYDASIKETTGAGWLAGAAYQIPEIALRASVTYRSEIDHKVDINENLSLLNFPGLTSVLAGLDVPASKLQAINSSGKTTITTPQSVNLDFQTGIMADTVAFANVRWVNWKDFSIQPYKFGKVSEAVGGLVGRPNGFNLVEYSDDQWSVNAGVGRKLNDKWAGNVSVGWDSGAGNPVTTLGPTEGYWNVGLGVQYSPTPQTFIAGGVKYFWLGDAKAQTGAQAGSDQYVADFSDNDAIAYGLKLGYKF

ST25 4190 cg89_1071

MNILIVHAHPEPLSFTSSLKTTAQQTFKKLGHQVEISDLYAMQFNPVASKEDFLELNQPDYFNYALEQRNATKQQLLSPDIQVEIDKVKRADLVILNFPLYWTSVPAILKGWIDRVFVSGLFYGGKRFYNHGGMAGKKAMLCLTLGGRTHMFGENSIHGPIEHYLSPIQRGTLAYTGFEVLPPFIAYHVPYISQEARETILEDYVTYLTHLDQLEPLKFPKLEEFDEKLYPL

ST25 4190 cg89_1072

MSGPLESVIVALNGKNCQFLNYAILLNSGIVT

ST25 4190 cg89_1073

MTPELPGTYINLLAEIVKRRGISCEQFLEGSGVTPEQLKKPYWYVEFNTLNNLLEHAIQLTHEPALAGYFALEMKASCYGSVGMAAMVSANLGEALKILEQFIGSRCDAFKPELKQEQDDVYWSIQQPLKSFQLSSDATIFLLLGFVHIAKHLTGLSSLGTVQLNMSEPIGFSKIKDKLGVSCLFEQKHNIWIFPKEYLMQPVLTADPMLAPLLKAQCKKDIDKLKLRSGWKAEISQVIKKLLINSQGEILKVKQVAQMMNMSERSLQRYLAIENTSFSALVDLTRKQYAQELLQNSLMSIEAVALSLGYADTPHFTRAFKRRMGVTPKYYQTQLKLKNLDISE

ST25 4190 cg89_1074

MNAQVSVTDLFSRKEIQELTEPSDAYGAWAVASTWAVIGGTFASLIMMWDYLPSWGKLLACMLALAVLAGRQLCLAILMHDASHKSLFKNKKINDFVGEWLCARPIWNDLQKYRVHHVRHHAKTSTPDDPDLSLVAGFPVSKKSLTRKFLRDLTGITGLKFSLGRVLMDLDVMKWTVANDQIWLDRSDKKFVDYAKSIAKNSTGAIATNLLLYGVLKACGQQRFYWLWPLAYLTPFPLFLRIRSMAEHAGMQTSNTALTNTRTTRAGWIARSFVAPIHVNYHMEHHLMASVPYFKLPRMHKILRERGHVPTPPSYFEVIHTLSSKQELTN

ST25 4190 cg89_1075

MKNTKINARHLIIDLFLSSAYPQLSIKQILVAAKLFNLSENGIRVATTRLLNEGMIQSVERGIYQLSPSAKDWAKVILNRKNGIKQTKEWQQHYLAVFTGTLGRIDRTALKKRERALRQFGFKELETGIYIRPDNLAYSFEKTCDELVLAGLEKEAKICIIQHVDSTTLKQIFSLWDTKQLEKNYEMYSRDITHWLENYAHSPLNDAAVKALLLGRETIALLMNDPLLPEPFVNESARNQFAQDVQQLDAIGQKLWKKIYEQELNL

ST25 4190 cg89_1076

MENFEVIKALFLGFVEGLTEFLPISSTGHLILFGHIIDFHSDGGRVFEVVIQLGAILAVCWLYRQKIINLIKGFFSGDVESRHFAISVLIAFFPAVIIGVLAVDFIKSVLFSPIVVAIALIVGALIIFWVESKQFEHKTDDATKITFKQALLVGLAQCVAMIPGTSRSGATIVGGMFAGLSRKAATEFSFFLAMPTMLGAATFDLIKNADVLTSDNMVNIGVGFVAAFIAALLVVKALVLFVERHTLRVFAWYRIVLGVIILIAAMFFNLSA

ST25 4190 cg90_1077

MESTSATAAPVVATNSKTRVLFASLVGTTIEFFDFYIYATAAVIIFPHLFFPASSGSAAVLQSLATFAIAFIARPIGAALFGHLGDRIGRKATLVAALLTMGISTVCIGLLPTYAQIGIVAPLLLALCRLGQGLGLGGEWSGAVLLATENAPEGKRAWYGMFPQLGAPIGFILATGSFLLLSAAIPEQAFMQWGWRIPFIASAVLVIVGLYIRLKLHETPAFQKVLDKQKEVNIPFKEVVTKHTGKLILGTIAAICTFVVFYLTTVFALNWGTTKLGYARGEFLELQLFATLCFAAFIPLSAIFAEKFGRKATSIGVCIAAAIFGLFFSSMLESGNTLIVFLFLCTGLAIMGLTYGPIGTVLSEIFPTSVRYTGSALTFNLAGIFGASFAPLIATKLAETYGLYAVGYYLTAASLLSLIAFLLIRETKNVDVNNQI

ST25 4190 cg90_1078

VLETYDPKEELFNAYSHGAGVVMAVIASVFLIIKGSYLSTAQWIGLWVYAFSLILVFTSSTLYHFAQTQNLRYWYKKLDHTAIYYLIAGTYTPFLSIAIPTQKAHILLIALWSIAAIGTLFKLVFIHRFQKISLLAYVLMGWLAVFVMDDMRTYLSKDTLMLLITGGLAYTVGALFYALKKVRYTHAIWHIFVLLGAGAHFLAIYWYVV

ST25 4190 cg90_1079

VSVFSFEQEQQFFHEIKQMLDQQTFERLILSQYKGELTQLEKITFRVVELHGKKQLSALYHHTTQDVTKNYSFEDGLEQIAALITQCKQANLFSTHQEIQLKKNKKKAMLNMGKKHSVTTVPTIQAHDREKQRYVQQGSAFLKELGITDEKAQIIPSMARKWKQINKFIEIFASAYEQIDAEQKELNIVDFGSGKGYLTFALYDYLQEQQKVPLITGVELRRNLVEFCQNVAEKVHFNHLDFFEGDVRSYQPEKLDVMIALHACDIATDFAIHTGIRLNASMIMCAPCCHKELRPQLHSPEVLQPMLQFGIHAGQQAEMLTDTLRALLLKAYGYETKVFEFVSLEHTSKNKMILATKRKNVTQPDAKIMAQIQALKEMYGIKKQTLELLLQDQLPIENIGCKC

ST25 4190 cg90_1080

MYKVIAGSWTQFEEDAKYIREQVFIQEQGIAPEDEWDDFDSIAMHFMVYDKEQPIATARLLPQHSVGRVAVLMPYRKQGIGKILMQHIIEYARQHKLPYLKLSAQTYVTAFYEALGFKVQGEVYQDCGIPHIDMTLALS

ST25 4190 cg90_1081

MTDFSRNALVVEGGGMRGAFTSGVLDAFLQQQFNPFDLYVGVSSGSTNVANYLAGQQGRTLTFYIDHSLRPEFIDYKRFFKGGDLLDLKWMWEIGEQEHPLDQQRLFASNPDFYMVLTHAKTGHAEYLRAGKDNLLNALRASSSIPVLTRHPVDIMGEPYFDGGVADALPVRWTAQQSGVKKLLVLRTRPKNYFKASSRGDQFLAKYAFKHHYGFANSLRNRCARYNASVEFVRSSNPEQQILEVCPPPLKNMAGRLTTNPKKLRYSYEVGLETGLQAIENWNAMK

ST25 4190 cg90_1082

MSDKNSSESAILGWKFVLIVGVLSAIFLGFFYLAMSNEPDYMPGAQRKAQQHEMQQKAEKSTDQQTQHDNMPEMDMQEHQHSHQ

ST25 4190 cg90_1083

MSAIQYEKNADNIVILTLDSTGQSANTMNAEFRDSLDEVTQKLKAETELSGVIFRSAKKTFFAGGDLDELIQVQPEHATDFFNMVEKLKGHLRTIETLGIPVVAALNGTALGGGWEIALSCHHRIAINDPKSKFGLPEVTLGLLPGGGGIVRMVRLLGLQNAFPFLMEGKQFGVDKAKSLGLIHDTAENEQELLDKAIAWIKANPKSQQPFDVKGYKIPGGDPKTPAVAQVLAIAPAMLKDKTKGCYPAPEAIMAAAVEGAQVDVDTALRIESRYFTQLATGQISKNMIGTFWHGLNAIKSGASRPADIAKWQATKVGVLGAGMMGAGIAYATAIKGIPVILKDVSVENAEKGKAYSQKLLDKRVSQGRMTAEKRDQILSLITATASAEDLQGCDLIIEAVFENQELKAKVTQEAEQYLAPNGVMASNTSTLPITGLAQASKDDKAFIGLHFFSPVDKMQLVEIIKGKNTSAETLAKAYDFVQQIGKTPIVVNDSRGFFTSRVFGTFIQEGMRLLAEGVHPARIEMAALKAGMPVGPLAIQDEVSLTLTEHVASETRKALQAEGKELPKTPVDEVIHTMIHELNRKGKAAGAGFYDYPENGKKHLWEGLKRWQKDHDISEQDMIDRILFVQALDTLRCYEEGVLESVIDANVGSIFGIGYAPWTGGAIQFLNQYGIDKAQKRAEELAAKYGERFTPPTLLKTKAEQKQNIQ

ST25 4190 cg90_1084

MSEAYIIDAIRTPRGKGKKDGSLYEVKPITLLTTLLNELQQRHQLDTSKVDDIVLGCVTPIGDQGGDIAKTAAIAAGWNDDVAGVQINRFCASGLEAVNMAAMKVRSGWEDLVVAGGVESMSRIPMGSDGGPWALDPETNLKSSFVPQGVGADLIATLDGYSREDVDNFAVKSQQKAAAAQANGYFDQSVVPVKDHAGVVILEKDEFIKGNTTLEGLAKLNPSFEMMGQMGFDAVALQKYPEAQKVNHVHHAGNSSGIVDGAALVLLASEKAVKEQNLKPRAKVLATALVGTDPTIMLTGPAPAARKALEKAGLTIDDIDLFEVNEAFAAVVMRFINELNVPAEKVNVNGGAIALGHPLGATGAMILGTLLDELERQNKKRGLATLCVGGGMGIATIIERV

ST25 4190 cg90_1085

MSCSAIYLDHFGNLTCKKCFYTVVNRQFIFLDGHLGQFKVNIKETEGFNMSRDTISIHFVNAALTGVKRLGMDVETLLSHVGIEAELLRQPKARISPEQYTRFIKMLWMVTQDEHVGFDVQPRRLGTFAIMCQLIIHAKTLGQALELSSQFYKLFGDEWSVTLERDKHEARLVPLIPKTLDPDHFITESMLMIWHGLASWLIERRLPLERVHFSYPRPAHADEYDALFFAPVMQFDAPRTEITFAADYLDLPIRQDEKTLEEFLKAAPAQLLVKFKNTNSLTSRIREVLKSQIGEEMPTLNDVASMLYLSPQTLRRRLAAEGKSYQGVKDALRRDAAIHLLLNPELTLEDVAQQVGFSETSTFHRAFKKWTGVTPGLYRQLHGYH

ST25 4190 cg90_1086

MYLADMGAEVIHIESPSRPDLIRIMPPYANGQATAHSYLNRNKQSIVLDLKDAASIDLIKAKISEFDIVIEQFRPDVMRRLGLDYATLAEINPRLIYCSITGYGQTGSYKDRAGHDINYLALAGIAGYSGRQDSGPPPLGIQVADIAGGSLHAVIAILAAVVERSRSGIGQYIDISMTDCVASLNSMAASATLAAQVEQAPEQGMLNGGIFYDYYMTQDGRYLSIGSLEPQFMAGLSAALDLPVLLQKGASLDVQDRQEVKQAIQEKIKAKTFKEWHEIFANLDVCVEPVLSLSEALNSPLAQQRGWIVNVPLQNNTPDTEPQIACPIKFSRSQMRYSFVGQKLGEGQW

ST25 4190 cg90_1087

MVSCLSVLSITLFVQHAQAAAAFDPNGSWMLGDWNGQRTALQAQGYDFSFGYTGEYAGILDSKQTSTHGSAYTGQLALGSHLDLGKILGWQDTEAQITLTYRDGQSLSEHSPALAGHQSSVQEVWGREQTWRLTDLWIKKKFLDQKLDVKVGRFGEGEDFNSFDCDFQNLALCGSQVGNWVGDQWYNWPVSQWAMRVKYNLQPDLYTQVGVYEYNPENLERGKGFNLSTDGSHGAIIPAEVVWSPKLGVQSMPGEYRLGYYYSTADAKEIADSTKTSHKQGVWVTAKQKLFQPADQADRGLTGFVNLTFHDSDTNKVDNMQNIGLVYKGLLNQRPQDELALGVARIHINDDWNDVQAKEYDTEYNTELYYGIHATNWLTIRPNVQYVRHVGALKNGDNTWVGGIKFSTAF

ST25 4190 cg90_1088

MTSPIRLKQNSGIKKKTEGQDTSINDLTLDNPFSE

ST25 4190 cg90_1089

MNQPSSRSGLTTFTVIIIGLLALFLLIGGIWLATLGGSIYYIVAGVLLLIVAWQLYKRASAALWVYAALMLGTIIWSVWEVGTDFWALAPRLDILGILGLWLLVPAVTRGINNLGSSKVALSSTLAIAIVLMVYSIFNDPQEINGEIKTPQPETAQAVPGVAESDWPAYGRTQAGERYSPLKQINDQNVKDLKVAWTFRTGDFKTDNDSGETTNQVTPIKIGNNMFICTAHQQLIAIDPATGKEKWRFDPKLKTDKSFQHLTCRGVMYYDANNTTEFATSLQTKKSTSTQCPRKVFVPVNDGRLVAVNADTGKACTDFGQNGEVNLQEFMPYAYPGGYNPTSPGVVTGSTVVIAGSVTDNYSNKEPSGVIRGYDVNTGKLLWVFDTGAADPNAMPGEGTTFVHNSPNAWAPLAYDAKLDIVYVPTGVGTPDIWGGDRTELKERYANSMLAINASTGKLIWNFQTTHHDLWDMDVPSQPSLADIKDKSGKTVPAIYVLTKTGNAFVLDRRNGQPIVPVTEKPVPQTVKRGPQTKGEHYSKTQPFSDLNLAPQDKLTDKDMWGATMLDQLMCRVSFKRLNYDGIYTPPSENGTLVFPGNLGVFEWGGMSVNPDRQVAVMNPIGLPFVSRLIPADPNRAQTAKGAGTEQGVQPMYGVPYGVEISAFLSPLGLPCKQPAWGYVAGVDLKTHEVVWKKRIGTIRDSLPNLFQLPAVKIGVPGLGGSISTAGNVMFVGATQDNYIRAFNVTNGEKLWEARLPAGGQATPMTYEINGKQYVVIMAGGHGSFGTKMGDYLVAYALPDNK

ST25 4190 cg90_1090

MYLYTDFDQQLINQRVAQFRDQTERYLAGKLSEDEYRPLRLQNGLYVQRYAPMLRIAVPYGLMNSKQLRKIAEVSTQYDRGYAHVSTRQNIQLNWPALEDVPEILAELATVQMHAIQTSGNCIRNTTTDQYAGVVAGEIADPRPTCELIRQWSTFHPEFAFLPRKFKIAVSALEEKDRAATAFHDIGVYIVRNEAGEMGYKIMVGGGLGRTPIIGSVIREFLPREDLIAYLEAVLRVYNLHGRRDNKYKARIKILVKALTPEVFAQKVEAEFEHTRETLKIQPEILKKLDEEFTPFDYQDLEDEDFTALFAEHPKFKQWFNINTHAHKVKGYRIVTISLKRAGIAPGDMTTEEMNFIADLADKYTFGELRTTHEQNIALADVPQKDLFDVWQALEQHNMARAHIGFITDIISCPGGDFCSLANAKSIPIAEAITRRFEDLDKVYDLGHLDLNISGCMNACGHHHVGNIGILGVDKKGAEFYQITLGGNSDHDASIGDILGPSFAAEAVPDVIEEVLNTYLDLRTEGERFVDTYRRVGIQPFKERAYA

ST25 4190 cg90_1091

MLNTALPVLYKDGTIADNTYQIIAEDGVIPQGDVVLTTAQLDQLANIQGKKALYVTVNDSPEEHTFPLSELDAIFIEFAGFGDGRGYSFAALLRRQGFQGELRATGDVFKDVLNYLKRSGFDSFVIKEGKDVQEAAAGLQDFTHPYQASTAVPKASYQTGA

ST25 4190 cg90_1092

MFALISKINPGALWLIFAIVTDVLSTFYSAKGNGLVNKTAQGIALVLYIISFACAAIALKYMQAGILYVLWSGVGVLATAFLAKIFLGQNIDVAGWIGIGFITVGLMIIAQYSNIDV

ST25 4190 cg90_1093

MQLNHYLNFQGQAEQAFNFYKSVFGGEFAMLSRYSDMPPHDGVTLSEAQKNLVLHVSLPINEYTVLMASDVIDQFCTPNSVFTQGNNHYIAINLEKDEQEKAKQLFDALSVNGKIEMPLEKTFWGALYGAFTDQFGVKWMVNCQLDT

ST25 4190 cg90_1094

MTDIVIVNGARTAMGGFQGSLSGLTAPELGAVTIKEAIARAGLQPTDVEEVIMGCVLPAGLKQGPARQAMRKAGLPDSTGAVTINKLCGSGMKAVMQAADMIKAGSAEIVVAGGMESMTNAPYVLPKARAGYRMGHGEIKDHMFFDGLEDAETGRLMGSFAQDMANTRGYTREQMDDFAIRSLKRAQTAITEGYFKDEIVPVTVSTRKGDVIVDQDEQPLNAKIDKIPSLKPAFAKDGTITAANASSISDGASALVLTSSEVAAQRGLQPLAKIIATASNSQHPSEFTIAPVGAIEKVLKKAGWNAQDDDLWEINEAFAMVTMCPIDDFKLDPEKVNIHGGACALGHPVGSTGSRIILTLIHVLKRTGGKKGVAALCIGGGEATAVAIEIL

ST25 4190 cg90_1095

MASVELKNTPRILTIHIQKAKYDAIIRHKNVVLCPSSDQLVCNTNWDNRVISFVDNNRNLQHDLNEELLTSIDLNHHYGSMKLQRFGKKQNSIIFQGSSGLPIESNGSFIYCSYDQLKNFKLILSKMGHVRLEELKNC

ST25 4190 cg91_1096

MLQKVLIANRGEIALRITRACKTLGIKTVGIYSDADKDLMHLRFVDEAVCIGPGASSDSYLNIPAIITAAEITGADAIHPGYGFLSENAEFAEIVESSGFTFIGPRPEHIRLMGNKVSAIVAMKKAGVPTVPGCDHAVTIHNALAEAKEIGFPLIVKAAAGGGGRGMRIVERVDTLLESVQAAQRDAEMWFGDDTVYMERFLQKPRHVEVQVLGDGNGHAIHLYDRDCSLQRRHQKVLEEAPAPNLPEQARADILQACVHACQLMQYRGAGTFEFLFEDGEFFFIEMNTRVQVEHPVTEMVTGVDIIEQQLRIAAGLGLELQQEDIEVRGHAIECRINAEDPTTFLPSPGKIESFYAPGGAGIRLDSHIYPGYSIPPYYDSMIAKLIAHGKDRETSLARMRQALDEMILTGIKTNIPLHKDLILQDKNFCSQAMDIHYLEKHLLKQVEEEKKAETA

ST25 4190 cg91_1097

MDIRKIKKLIDLMIESDLQAIEVKEGDQSIALTRRNPVVAAAGVALPAAPVAEAPVAKTPRGAVETSPMVGVFYAAPSPGEAPFVKVGQTVSAGETLGIIEAMKIMNPIEATQSGVIEEILVKNGEVIQFGQPLFRYRA

ST25 4190 cg91_1098

MSSTILVIHGPNLNLLGKREPEVYGHLTLDNINQQLIAQAEQASITLDTFQSNWEGAIVDRIHQAQTEGVKLIIINPAALTHTSVALRDALLGVAIPFIEVHLSNVHAREAFRHHSYLSDKAIGVICGLGAKGYSFALDYAIEKIQPSNPN

ST25 4190 cg91_1099

MSRRFFAVLGEFFSPDDLEAYSIDECFIHLTPYLQSIDISDYCNKVRNTLLKWLGLPCCIGIGYSKTQAKLANHYAKKIKSFKGVCNFITLDPLIMEDLMQQTSVKEVWGIGYQLVKQLQSYEVYTCLDLTFANEHHMAKAFSVVMARTIRELKGQSCIQLDDPAIPTKRILASRSFAQALSSIEIIKQALIFHLNRAHRRLMKQEQLCACVQVMLYEKTDKPPYKKATSQAIGLHYATDDLCILTKAAMQQIDVLYKENKSYIKIGVLFCALHARQQHIDDLWQPLELIHQRQQLMETLGTVRKRFGSHYLQVGYHSRNPSWQMKQCHRSKNYLTRWNEMLTIEDAYTPVTQNT

ST25 4190 cg91_1100

MSKLEKTQLQLGYIPLLDCIALLWAKQQGFFEEVDLDVTLVKEASWASLRDRLAFGLLDAAHCLSAMLPAAAMGADQIGIALQTPLVLSKNRAFISLSQKLIHQLAIQENDNAQTTAQKVIQYIEQDHTLSLAHVFKHSIHHYCLREWLALADSRIAQMLKLKALPPPYMVEALDNHVIDGFCVGEPWNTQGELLGLSKIVCSSQDIIPNVADKVLAVTQEWAEQHPQTLVALTTAIMKAQKELSNLKDFAPVLKLLVEFGIVRFHCSEEVHVDKYYMIQNIVKYLVKENAAPQPEDFHWLFQQMQKWEKLQLAPDQIIELSAQCINLECYNQAKL

ST25 4190 cg91_1101

MPKLKIALIDDDHARADYIKNSLLENDFEVVACLTLDHLNIFRLEDLQADVILLDMDHPHRDIIESCVSSYDLPTVLFTKNSDKDTIKQAIDAGVTAYIVDGIDPARLHTILEISIEQYKKHKKLEGDLKEAQTKLADRKDVEKAKVLLMQLHGLPEDTAFQLLRKNAMSHRITIGEMARRLLDAQKLLNDQLKDE

ST25 4190 cg91_1102

MIGHGMVGHKFIEAILEKADDELEITILAEEPRIAYDRVHLTEYFSGKSAKDLSLARFDFADAYGIDLRLNTKAVAIDTTAQTVTTNHGDVISYDKLVLATGSYAFVPPIPGNDRENCFVYRTIEDLDAIRAASLKAKTGVVIGGGLLGLEAAKALRDLDLETHVVEFAPRLMAVQIDDLGGKVLRRKIEDLGVKVHTQKATQSIESGVNTTHVMKFADGSELETDVILFSAGIRPRDELARNSGLAIGERGGIVINDYCQTSDQNIYAIGECALWQNKIYGLVAPGYDMARIAAKHILDEECHCFAGADMSTKLKLMGVDVASVGDAHAMTPGALSYFYADEHALVYKKIVVNADKTKLLGAVLVGDAKEYNDLLQMMLNGLALPEVPESLIMPGFEQSAAKSGGSGVDLLPDSATICSCNNVSKADICQAISDGSTSLGALKKCTKAATACGGCAPLVTQVLKSELQRQGVTVNNHICEHFPYSRQELYHLVRVNEIKTFDDLIHQHGHGLGCDICKPAAANILASCWNDFVLKPSHAGLQDSNDYYLGNIQKDGSYSIVPRMAGGEVTPDGLIAIGQIAKKYNLYTKITGGQRVDMFGAQVHELPFIWEELNAAGFESGHAYGKSLRTVKSCVGSTWCRYGVDNSVGLAIELENRYKGLRSPHKLKMAVSGCTRECAEAQGKDVGVIATEKGWNLYVCGNGGMKPRHAELLASDLDKETLIRYIDRFFMFYIQTADRLQRTSVWRDNMEGGLDYLKSVIVDDSLGLAAELERRMEHIIGTYQDEWRTAVENPEVRKRFQTYINAGANEQADPHIQFTTERGQIRPLTEAERSEDRIPMVEA

ST25 4190 cg91_1103

MNIVQDKNMLPDDQWIDVCALDDLTPNTGAGALVGGQAVAIFRVGHEKRVYVLSNKDPFSQANVMSRGIIGDLQGERVVASPIYKQHFSLATGRCLEDKDQKLAVYPSKIVDGRVWVNAVPQKTYITNTGVSQDKLRLVLIGNGLAGMRCLEDLLDMAPDRYEVTVIGEEPWGNYNRIMLSPVLSGEKTIEDIMLHPPKWYDDKGIKFIAGDKAVKIDRPRKVVYTEKGQTVDYDRLILATGSAPFIPPVQGVDLKGVLTFRDIYDVNTMIEYCGSKTNAVVIGGGLLGLEAAYGLKQRGMNVTVLHLMDRIMERQLDGRASQLLRHSIEQKGIQIITEANTEALIGDENGHVKQIRLKDGTVLDADLVVFAVGIRPNIALAQSAGLRCNRGVLVNDTMQTFDPSIYAVGECIEHRGQTFGLVEPLWGQAFICATHLAEHGSLTFKAPTVPTQLKVSGVDVFSAGNFEPKNDYEDIILNDEKRQIYKRIIIQSDRVIGAVLFGDTEDGMWYAELIADQTPVSSFRNKLLFGRDFALKNAG

ST25 4190 cg91_1104

MNSIPSVEIDSSDHVAKTTKTTCPYCGVGCGVSVNVQQKPQGPVVQVEGDAEHPSNFGRLCIKGSRLADTLGLETRLLQPMFGRKPLRTVTTWDAAINKIADKFQSCIDQYGRDSIAFYVSGQLLTEDYYVVNKFVKGYLGTANIDTNSRLCMSSAVAGHKRSFGEDIVPASYEDFEHADMVVLVGSNTAWCHPVLYQRIMQAKSHNPDMFVVVIDPRFTSTCEQADLHLPILPGQDVALFNGLFQYLYQNGHADQAFVDAYTEGLQEVLVSSQQETDIAYVAKRSGISLDKLQQFFEKFAQTEKVITLFSMGVNQSSQGVNKANSIINCHLLTGKIGKLGAAPFSMTGQPNAMGGREVGGLANMLAAHMDLDSPLHQKVVQTFWDSPFIATQAGLKAVDLFRAVEAGKIKAIWIMATNPVVSLPDADQVKRALEKCELVVVSDICADTDTTAYADVLLPALGWGEKDGTVTNSERRISRQRAFLPAPGEAKADWWSVSQVAKKLGFKGFDFNSAVDIFNEHAALSAQDNADINTREQTDTFRYFNLKGLMNLSTAEYDALQPVQWPVWDKNQDAKAVQQLFGKGQFSHKNAKAKLIATVAINPVHAISEDYPLILNTGRIRDQWHTMTRTGLSPNLTSHRAEPFCEIHPSDALKFGVRDQGLVEVRSKWGSCVLRVTFSSGVRRGQIFAPIHWTEQVASDARIGKVVNPEVDAISGEPEFKHTPVTIQPFYTTWQGVLYIREGYDSHIQESLQHCAWWTKVKMVKTNRYELADRQTFHDTQKNLKSFLPFADETFEWLSIEDISSQLSHSIILKDGIVIASLYIAPPDLLPDRDWVASLFKRERLSALHRKALLAGMPMSATNNDGPLVCSCFKVGKNKIIEAIKTQNITHEKQVTACLKAGGNCGSCLPEIRGLIKACQQEVEV

ST25 4190 cg91_1105

VNKGYPITDLVILAGGQARRMNGLNKLLQQFDGDTQLLKIHQKLKSSVSEIWVNSHRDYSIYQSIVPDIKCFQDDASGFFGPLMGMKSAWSHVKADYVLFIPCDVTYIPTQVVAKLHSALRKNKQAQAAYVSINGDALYPFCLLKRESLEVLEQQIDKQQLSLKNCFKLLHAQVAIFQKQNLFFHSINSLDELQQYKQIKAFKEIFSTN

ST25 4190 cg91_1106

MMDAAAAQQSKTDVTACILCSRNCGLSVEIKDNQFVKN

ST25 4190 cg91_1107

LSKIKGNPEHPFSQGYICQKAARLQHYQKHADRLTAPLKRQPDGSFQEISWDVAIQEIADQLVQIRDKFGGTAFASVGGGGQGNHLGAAYGRQLLLAMKSYYAYNSLAQEKTGDFWLNGRLFGSQACHTTEDVEYADYVLFIGTNPFQAHGIPNARDTLKHIKKDSNRTMVVFDPRVTETAKQADIHVQLKPGTDAFLMSAMIAIIIKEKLYDAAFIEQHTHGFEEVKAAFNHVPIEDYIAKADVPVDLIYQVVRDFSKAKRGCVRIDLGIQHTLNTTLNGYLEKLLYLLTGNFGKQGTNNLHTMFIPILTDTDERKPKYRRSVYHKMFPISGFFPPNILPDEILKAGEKRIRAVFVDSCNPLLTYPDTPAFEEAFKALDLLVVVDVAMTETARLADYILPAHTQFEKWEFTGFNLEFPKNGFHLRHPVLAAQANTLPEAEIYTRLLEAMYAIPKNFPILEKIAAVDSRKTAYLPYLSALGLTLARHKKYIPFAASIIYRTLGKRLGNSADSVAFLLPLSIQYAVLHTKAVRRAGYKGNPLTQGVKLFEQILKQRSGMVLSQHEYDEVWKLVAYKDKKIRLAIPEMLSELASLKSHNVNVEEFPFILLSGERRSYNANQIYRDPAWRKVDAEGALRIHPEDARHLNVDAGGQLKCISAHGEIQVAIELDDGMRRGVVSLPHGYGMRFQNGEPIGPQLNLLISAEHCDPLSKTPYHKYVPIRLEKC

ST25 4190 cg91_1108

VQMMKQYHSETAPSLLQDQYGRIKRKLRISVTDRCNFKCVYCMPEHPEWLNKQDLLSFEALFQFCHFMVQQGIESIRITGGEPLMRQGIVHFIRDLQSLKSLGLKRISMTTNGHYLAKHAKQLKDAGLDDLNISLDSLDPVQFKELTKKKLEPVLEGIQAAKEVGLPFKINCVLMKDRNDDQILPMVKWSIAHHIPLRFIEFMPLDGDALWSNKDVVSEAEILQVLQPYYSVQVIEQQHEPARQYLINGSYHLGIISTITHSFCHQCDRIRLTAKGELYNCLFAPQGLNIKPQLQTLVSKQHTPEYGVYIQNLKNLVHPYIWHKAKGFHALQHQQTRKISMHMLGG

ST25 4190 cg92_1110

LTQENNLNVDARLLNIENLSISTQDQPLLEGLNLHLQAGDTLAIVGESGSGKTISCLAMMGLLPEKLKTSGRIIIDSKNILEIDEKTQTHIRGCQIAMIFQEPSTALNPLHRVEKIVGENFILQGGSKAKAQQQVIQLLKDVGIANPEYILKRYPHELSGGQKQRVMIAAALVLQPKILIADEPTTALDVILQTQILELLKSLQSKYGMALILISHDLNLVRRYADKLIVLHKGQVVEQGQLQQIFKKSCVNLYPAIIRT

ST25 4190 cg92_1111

MSFIVRSRLQKFRQHKLGWASFILFSLILVMSLAAELIANDKPLLVKYEDAYYLPIFKTYSETTFGGVFETEADYKDPAVQELINHKGWAIWPLIRFSYQTPNLDLAVPVPSPPSKQNWLGTDDQGRDVLARILYGLRVSLLFGLALTGCAALLGIFVGAIQGYYGGWVDLLGQRLVEVWNGLPMLFMVMILVSMFTPNVYWLFFIMLFFSWTVLVGIVRAEFLKARQLDYVRAAQALGVTDRAIIFKHILPNAISSSLSQLPFILTTNIIALTALDFLGYGLPPDAASLGELLLQGKNNLDAPWLALSGFFSLAFVLSLLIYIGEAARDAFDPRK

ST25 4190 cg92_1112

MIPTLFIILLINFVVVQIAPGGPVEQAIHEVESGLGAGRILGTEMYYQGAKGLSPEMVEQIKAQYGFDHPPVERFLLMLKGYLTLDFGQSFFKDKSVVELLWEKMPVSISLGLWSTLLIYLISIPLGIKKAKQQGTWFDRSTSLLLVVGYAVPSFVFGILLIVFFAGGSYFQWFPLQNLVSDNFYQLSWFGKITDYLWHMTLPLITMVLGGFASLTYLTKYSFLEELNKPYVLAAYAKGLNSQQVLYKHVFRNAILVVLVGLPEALAGIFFVGNLFIEIIFHLDGVGLLGFEAIVQRDYPVIFGVLFFFTLFSLLLRLICDVLYQWIDPRIHFDVQGGK

ST25 4190 cg92_1113

MQKLYSQLSLLVGLSVFSQVIFAAQQTTPYIAIHSKPKYLAMAAMPYANPNAPKGGILSQAAQGTFDNLNSMNGKGNATEGVNYLFDTLMTQSLDEVGVLYPLLAEKVSYDPIKTQSVTFYLNPKARFSNGLPVTAEDVKFSFDTYQTKSNFGLQMYLGDLAKTEVVNPHQVKFTFKSGHNPKMPFVVASLPIYSKADWQKRDFTRISLQPIIGSGPYVVEKIDAGRSISYKRNPNYWAKDLPINKGRYNFDHLKYVYYRNWDIAFEGFKSGQYTLHEETNPKKWVTDYHFPAVKAGLVTQYKFRHHNPIATESYVFNTRRKPFNDIRFRQALTYAYDFEWQNKALFYGQYQRLQSYFENSDLAATGRPSNNELAILKPLLPKLSPIMQKAVLADWKYPASDASGFNRQNLLIARQLLIQAGYKIKEGQLYTPEGKPVKIEFLIQQDGKQRTLMPFVRNLKKLGININLRQVDAPQYLERTRRYDFDMTTMNLPQSLNPGNEQAQFWGSAAAVQDGNYNYAGIRNPVIDEVISKLVTAKDREQQITYTHVLDRLLRAGYYQIPTYGKGDYWYAYWNMYQQPKVKPVLSAGIEYWWSNANQAKKVAQYLHQQ

ST25 4190 cg92_1114

MYKPTTFVWQPSAASLFKITVLSSALAALGITTGCSSTPQSAKTSKTKQVSGAGYLDASSLDSLEDLLSATDMRAVEGDRLLILKHGDVWKRMAVGFKMDLNHWDPRIEAQRSWFISRQPYLDRLSARASRYLYHTVKEAERRGLPTELALLPVIESSYDPAATSSAAAAGLWQFIPSTGRIYGLQQTGMYDGRRDVVESTRAAYEFLGSLYNQFGSWELALAAYNAGPGRIQQAINRNQAAGLPTDYWSLKLPQETMNYVPRFLAVAQIIKNPRAYGVSLPPIANRPHFREVTLSAPLSLNEIASVTGLSRAELYALNPGYRGETVDPASPMRILIPADISPSVDNKLKGMKAGGSSGWWASVTSPSKPTTTTSTSVTVRTTPSTPAQPVRPSTPAKTSSSSVTVKTATPRGSDALAAFAASADVPSAPRIPVAVTPAANIKPVRTEPPISATEREKILAAVRAEGEKETVDQALEPQATQAEKDQVVAELKALAPQGTEIVDPYDGKIKLTAIQTSQSVAEQQGKEVSKGFAYPKTLAEDATLANSEDAQRNKDKPYIKTDTDVVVVQPKGKRSTYTVQPGDTLAVIAMKNGVNWRDVAKWNQIDPEKTLFVGTSLYLYDAKPQEVETTAKSVAKPDVYVVQANDSLTGVANQFNLSVKQLAEYNDLSVTDGLFVGQKLQLKEPKGNRAAKVEPKAIQASTRRIATKSYTVKRGEYLKLIADRYALSNQELADLTPGLSAGSNLIVGQKINVPAKEITVDEVDDSKASGKYEKLAAGPSYKTESYKVQRGDTLSSIATKSKISLAELAELNNLKANSHVQLGQTLKVPAGASVPDQYVVQSGDSLNAIAAKYNLQTSYLADLNGLSRTAGLRAGQRLKLTGEVETTSKVSAKNTKEETPETYTVKSGDSLGNIANRYHLQLDYLAALNGLSRNSNVRVGQRLKLTGDLPTVETAKTDTAKSSPKAVVAGKNTEKYTVKAGDSLNAIASRAGISVRELAEMNALKANANLQRGQNIVIPKTVVEYKVKRGDTLIGLASKYGLETTLLAELNNLTPSTQLRIGDIIKVPNL

ST25 4190 cg92_1115

MSQTITLYVDGACRGNPGLGGWGAYVITEQGEHKLFGGQPDTTNNRMELTAAIEGISFCPPDAQLIVWTDSNYVKQGITEWIHGWKKKNWKDVKNPDLWQKLDAVCAGRNIEWNWIKGHAGHAGNEMADQLANLGADKTAQELKQPQSATVSQDIKKPEQDWLLDDPFGFDLVETTEDETVTLEQTQEVEMIVADEEMNVVEAVEQEEQQQNTNIHPQIVMTEAKLKLQGPRQLILDTETTGFYFQDGDRIIEVGAIEMINRKLTGSSIHIYINPEKPVGDSVQVHGITDEFLVDKPKYAEIADTLFEYLKGAEIIAHNATFDMNFLDMEFKRVGLPALSEVCEVTDTLALAKNKHPGQKNSLDALVRRYEIPARDRTFHGALLDAEILSDVYLAMTGGQVSFDMDALSQSESDQQKTTKARVQIELPVIYPSDEELNIHETWVKEFEKKHGEPCLFAKLNF

ST25 4190 cg92_1116

MSEHNSIQFDPTALLIIKNEIDNSIKLVEGAVSTLIEEQALPFGIDDALEQFKQCTHVLRLIDIPYLAKITQYSTELMQKIMANPEHINTDDVVALSEGTTMVKRYIEFICLREVEVPQFLLDTLNNLEKALNKPLTSSGKQIASKLSTASLELPLPEVLINERTQFIHQLYKLSLHQFLNKTESARDFQAFKLIGSYLVSMAQGQPSQQYWQLVNSAFSHIDELVLNDARLRVFINLENAISLFLASPEGFEANLTALADILSIVIGQEDQLAQQIRSQLNIGHEFLTDTQLKALSQHLYGPDFDTMQTVSQLILSEMNKVRNDIEYNYQNMSPEKAQQLQSNLMQLAHTFKLLNLNEAASELSQQASSLSQINILSNENYAQQLMKSILSAMNAIGILVRHYSSNRLQIRVNNTNISLDRLDEAHQTLLNETKNLTDFVCQSLTLYANDQTQNIEAIAGSLKELAGAAEFLGSTVQQNALLETAKFVQKQIDQNQPFNHDQIHCIFNVLAGLDMLVDNLKNKQPVLQSMFDVALLSSQQLQKKAA

ST25 4190 cg92_1117

MSELSLAYIFHHQQLLVDQNLQLPKVEKLASDLLFTHDEQVIARDLLSEEAIPEGLQLVPIRQLISNWSKEQFLQASRAVQLLEWRRNHKFCSHCGHATEVHPTEYAMVCPSCRYHQYPRVNPCIITIITKGDDEILLAKSIHNKTNMYGLIAGFVEVGETLEEAVQREALEEVGLKLKNIQYMSSQPWPFPSNLMIAFRAEYESGEISLQEDEIADAQFFKFDQLPEIPFKGSIAHAMITQITQAK

ST25 4190 cg92_1118

MNLIEQLQSKIEQARTLYKEFRLYDLGSFALNRFTPKDGFEQVLNVRYGLKPRHRLDIYRSTKRLAHRPLIVFVHGGAWQHGNKRDYLFVGEAFTKEGYDVAVINYQLAPKNIFPSYVDDLTQALNYLHQNQEKLEISTENIVLMGHSAGAFNVMSAVYHPKPNTIQCLGNIKAIFGLAGPYHFDYKGDPLAEDAFDQSISYKEVMPYYFVNQNHIKHYLLMAENDQLVKKENTFDLDQALRQSGNHSHIAVIPKTGHITILASLSSFVSHYFRTKRTILHFLDEVF

ST25 4190 cg92_1119

VLKRIAVTALCVASLSGCASFISGGTGTAPVGTDSGVRSLGQVFIDSSIKRTANINLYKLDQRFKQSRINIESFHSTVLLTGQVSDPYLKQLAEDNVKAMSDVKAVHNYITVGNKVSYNTIMQDAGVTANTRALLMKAPVVSDSKVLVHTEDGVLYVMGRLNTAEINDLNNVLQNVGNVTKIVTLIDNIDLAPAPAASTASATTTPVINNVLAQPTVQTPVAIDPDQTDPASSAQ

ST25 4190 cg92_1120

MLVAQQLGQWAEQTALKLLKEQNYEWVASNYHSRRGEVDLIVKRGNELIFVEVKARGQGNYGQACEMVTLSKQKKIIKTAMRFLQRYPSYQDFYCRFDVICFDFPQKIAKTVQQDFSKFHYDLQWIENAFTLD

ST25 4190 cg92_1121

MSAQLFVVATPIGHLDDMTFRAIDILKSVSVVAAEDTRQSAQLFKHYNISTPLTACHDHNESNKIEQLVQKLLAGENIALISDAGTPLISDPGFKLVRAAQENGIRVVPVPGACAAIAALSAVGLPSDRFSFEGFLPSKASQRITQLEKLKNETQTLIFYEAPHRILECVKNMAEVFGENRPVGFAREITKTFETIKKMTLKDLVSFIENDHNQEKGEIVLVVGGAPEKTDLEQEKLDELLKRLLQDLSVKAASQLAADLTGIKKKVAYQRALELTQS

ST25 4190 cg92_1122

MTESLGKLGPHEGQELELLLSGKKPIAYFYELLPIEFIKHLEQGSLSMISKDIETSLSLPFSIMLIYKDASLADLNELMLCIERSLKATQLEERLELDRRIGQLLGYSVQDIEFYIQHISNRHLKTKI

ST25 4190 cg92_1123

MLDSKQCEAFLAVAEVGSFDAAGEHLCITPSAVSLRVQALEKYLGQILIIRGRPCVLTQAGQTLLQHLRHTRLMEQNLLQGLMGKSSESEFYKIALASNADSLATWLLPSIQQTLFKEKIVLELKIDDQSHTHTLLETGQVNACITAEEQVMAGCLAQPLGKMRYKMLASAEFVNRWFSGGVNREVLRKTPAVIFNHKDLMHSEVLLKGYGLPMQSYPYSFIPATDAFVKAIQLGLGYGMVPELQVQPLLENGTLVDIMPEAQLDVPLYWHHWKRQSKQLDVLTETIVESAKQILR

ST25 4190 cg92_1124

VFFKGLGIGSGLIVAIGAQNAFVLKQGLKQQYVFWLCLICALSDSILIACGVLGFAEIMTASPILITVAKYLGATFLFVYGAKAFYAAFKTNQGMELDSSQKQTLTQALITCLAFTWLNPHVYLDTIVLIGSVATQLEDKISFALGSILASWIFFFSLGYGARFLKPLFINPKAWKILDFMIGCVMWSIAISLLI

ST25 4190 cg92_1125

MAGCGCASTCSPTKKVSPRFRKALWIALVINALMFVVEIVGGYKAQSVSLWADALDFAGDAANYALSLVVLSMSLYWRATAALVKGITMAAFGFFVIAKVVWSFFHGVSPEPMVMGAIGVIALIANVSVALMLYAFRDGDANMRSVWLCSRNDSIANIAVVLAAVGVFGTGTIFPDLFVAFVIAYLGVSSGYTVIKQSLQERKQSKLMLGSEA

ST25 4190 cg92_1126

MSLSIGQLSKKADVPIDTIRYYEKVGVLDRIQRSENNYRVYTDQTLADLLFIKHCRELDISLSDIKTLKEMKAQPKRACTEVDNLVNKYLIEVSEKIERLLLLKETLIDLKQHCSTDRTVDECGIFKRTTKPSLNHHKTGNKKASLK

ST25 4190 cg92_1127

MTDINDYEDAEDSAVDEDEAKAAESGAADEEAGNLTDADLAEAETLTVTAKQKQRQALEDEVAAFLARGGRITEVPPDEHSDR

ST25 4190 cg92_1128

MSEVLDVVIVGGGLVGGLTALLLAQGGVQPTVLDAAPVLDVEKTLSVMNPRVLALSQATIHLLKTVNVWDDLARQMPYTGMQVWNLNGYGEINFGHESVQRPIPEQSLGSMVEPSVLNVAIQQKMLEQLTDYRTQVRVTRIEQGVGCWHIQLADGTTLKTKLLIGADGANSFVREQAFIDLDVLDYKQAAISCAIKTSKPHHYVARQIFLPTGPLAYLPMASLEESENGYRQSIVWTLPDDYADEYSALSDAEFMQLLTRESQHMLGEVLDVRSRAQFSLKARAAKQYVKAGLALIGDAAHVIHPLAGQGVNIGCLDAAVLCDALLHDLGRGVWAHEQTLMRYEHRRKGQNDAMMHSMSAIGWLESSELFPLIWARNVGLKQVEQISFLKERFMQQANGLGALQNTQYSR

ST25 4190 cg92_1129

MQQQVIIVGGGMVGLSLSLMLAKANIAVKLLEAVKYPNYDDQNVAPYHSSFDARNTALSRRSVQIYQKLGLWDALQQHATPILQVHITEQGSFGKARLIAEQEKVESFGQVIENAWLGRVLLTQVRQQPLIELIDGVQVTALTQDAEKVHIEAQRADEVLKLESKLLIAADGRDSFCRQAIGVGVDVHDYDQVAIVTTVQTSKPHEHVGFERFSALGPLALLPLPGEYRRSVVWPVKKGTEGEWLGEENDQHFLDALQKTYGDRAGKFEKTGKRFSYPLSQVLAHKQAVGRVILMGNAAHTIHPVAGQGFNLCLRDADVLLRYLVNQLSTSDDIGNPDNLLAYEQARLSDQQRVIKFCDTVVRGFSNQNPLLKLIRNTGLIAFDVIPGVKPLVANYAMGLKA

ST25 4190 cg92_1130

MKLPQADFQHRRNRLAEKMGPNSIAIIATREEMYRNRDADYKFRADSSFFYLTGFAEPEAVAVIETFDDVTDYSYSLFCRERNREMEIWNGYRAGIDGAIEEYEADEAYAIDLLDEEIIEKLLNKERLYYRIGHNAAFDARVSQWIKKANAEHRHEAAPAQLVQLDRIIDEMRLIKSPQEIELMQIASTISAKAHTRAMQTVRPGMMEYALEAELNYIFGQNGCVPSYNSIVGGGANACILHYVENNQALKDGDLVLIDAACEYEFYASDITRTFPVNGKFSPEQKALYEVVLASQYAAIDAVRIGNSYREPHEVAVKILTEGLVDLGLLKGDVSELIETEAYRQFYMHGTGHWLGMDVHDVGSYKKDDDWRQYEEGMVVTVEPGLYIAPDDETVDKKWCGIGIRIEDDVVATSKGPRVLTADVVKDIADIEHLMAQAKA

ST25 4190 cg92_1131

MQDDISGWTEWNAHFEGIEEISSPSELHGLLTGIVCVTEAPTREEWTQILTTLNVPELNEEALALLTDEAEDVAHALSEDELDYLPMLPDDEHLLQDRVQALSDWCAGVVLGFGLASGHVRTDERELIEHLQDVAAVEFEDSDNDEEGESSYEELYEFVRLIPVSLSIGRKKVTVAESSLLKNFYAKSKTSTVGTADQNIVEMFTPHRPS

ST25 4190 cg92_1132

MLEQLQRLQAHIGVLKTRLHHLESENSALSEAKELAETEHHAQVVQKNSIITKKQEEIETLTEKLTQLQGQFQQLNQDANTLAERYSRLEKSTTDLKNRFQEILAERNELRVTKEKLQSQQRQTQQELHDLQQDRDRLLQKNELAKAKVEAIIQRLAILGTAQDQHAQEIQQLAHPNAEAGEETQS

ST25 4190 cg92_1133

MSEQVMVELRLIEQTFRLATTSDKREELERAAELLNEKFNDMRRSAPRVEHNKLVIMVALQLTQEVLSLNKSLQEYAHCERLLQTILEDVEQIV

ST25 4190 cg92_1134

MKFKILLLSFIATGCYANESTADPDICNIVKKVAYNVMEARQQKVPAQDLQQIADGLADEEAKQLYQDLISSAYAAKVFKTSFFKRQAIEDFQAGWYEECLRRNE

ST25 4190 cg92_1135

MKLKILTMMLCVALLSACTKQAESEAPQMDYKAQFEESDRKIGEFLDQLDNPNTPQEVKVKILCHDYPDVYKKQYMPALIKVSPKPYTEEKLLSDLKSATDYYKGTLGIKCNE

ST25 4190 cg92_1136

MNELSFIRKNLRSRRRALTQFEQKQAQLNVLHCLNHLPIFHSSKKIGLYLHAFGEIHTDLLIKLCFKKNKQVYLPMICSMNQHLVWVKINKNQYLSRRFSHHPLGMKEPMATRGKHVSQLDLLLMPLLACDHYGTRIGMGGGYYDRTLASAKHKPYRLGLAHQFQFIEHTLERQSWDQPLDGLLTPQHFLLF

ST25 4190 cg92_1137

MSELIMNEKTDLEPQVPSVLPLLALRDVVVYPHMQIALFVGREKSINAVDVARNSDNLVFVVAQKDSLTEEIDHDNLYQYGTVAKIVQVVNHENDENCIKVLIEGLHRSKLKKIIDEDSYLTAEHELSPMTINVDKATQETRLQELRNLFAQYAEAKLRNARELVAAANKIEDLLQLMFFVATRVPLNIEIKQKFLEYDEFEAHLQELMNYLMNQSAEQQIEQTLHDSVKRQMEKNQREYFLNEKMKVIQRELSDMNGGAEDDVAEIEKRLAEADLPEHVRKKAEAEFRKLKAMQPASSEAAVVRNYLEVILDTPWNKASKVSINLNKAQEILDADHYGLDDVKDRIVEYLAVQSRVKKLKGPILCLVGPPGVGKTSLGESVAKATGREFVRMALGGVRDEAEIRGHRRTYIGAMPGKIVQSLTKVGVKNPLFLLDEIDKMAQDYRGDPASALLEVLDPSQNSKFNDHYLDLDLDLSEVMFICTANSMNIPEALLDRMEVIRLPGYTEDEKVNIAERYLVPKAIKNNGLRPKELTIHEEAIRDIVQRYTREAGVRNLEREVSKIARKVVKEAVSKKSKNLQLDVTSANLPEYLGPHKFDFGMAEDEAQVGRVNGLAWTSVGGELLTIEVAAVKGKGKFITTGSLGDVMKESITTAMTVVRTRADELGIEASRFEETDVHVHLPEGATPKDGPSAGLALTTALVSAFTGIAIRPDIAMTGETSLGGRAMRIGGLKEKLLAAHRGGIKLVFIPQDNVRDLAEIPDNVKEGLEIKAVKSIDEILPLALTSMPKPLPKTPIVKPVEGSKAARH

ST25 4190 cg92_1138

MKIRILTIGQKMPAWVLTGFEDYFKRIQPFVQTQVIELPMAKRGKNDSEADILKYCQIEGESILNALKPNETLIALEVGGRELSTEKLADTMKQWMLEGNDVALAIGGPDGLSDQVRKAAAWHWSLSKLTMPHPLVRILLIEQLYRAMSINHNHPYHRA

ST25 4190 cg92_1139

MELQTLPGLFISHGSPMLALDPEQVGPALHRLSVNLPTPKAIIVMSAHWESKALEVSSSTRPETWHDFRGFPPELYEIRYSAPGQPELAEEILKLLADAHFFAHANSTRPRDHGVWMPLLHMYPEADIPVVEISLPRGLTAQEIYRIGQTLAPLREKQILLIGSGSITHNLAELSWQGDNSKVPEWASTFRNTVVSKLSHQDYDGVLEWQTLPFVKRNHPSLEHFAPLFFAMGTGHRFTIVHSSFTMGSLGMDIYRFD

ST25 4190 cg92_1140

MKIKYLILALLPFSLMACQTVSNTQAPIVSEQQQNLATTLSEYAWTYQNVKASKPLILNFNADGKLAINTGCNGQGGTWKVEGNQLVTSPLASTMMACQDDLMKQEQLSNSIFSEAKLPIEISNNNGQVILSVTDKAGQKHIFQGEKATNTQALTDYSWSYQPENTKKPIVLNFTNDRLSIDTGCNRQGTTWKVENNTIVTTDVMSTMMACEPALMKQEQFSSSLFQKRAIPFELNTTNVDQPTLTVADAQGQKYTFTGKMTPEAKYQSEGKTVFLEVAPETKSCTGVAPQTCLQVREVKYDDKGVKTYADKNWSLYYGQIEGFEHNPNQRVILRVKRFEVKNPAADQSSQADVLDMVVEQELVKKA

ST25 4190 cg93_1142

MPSLTPLHETYCKWDRTPPSQADVLEGLAQLFSTSLNGVHELIQVINIEVLKNAFGISAQNAKNFQKRPSVQKAYQFSYGALQKYGSCFLAPGLRRIIEKFPTLHEKPLTPTLHFLVGVLNGIFGDYLLKQHSPLALPMVLYDHYGAIQQGELAGRIVILVHGLCMNHLTWSNAHYGGIGERLLAQRDHNTMLYLNYNTGRRISANGRSFSNLLEDLVQRNPRITSIDLIGHSMGGLVSRSALFYGKQNMYQWIHMVENLVCIGSPHHGAVLERFGFALQDRLGAFPFVGLIGQLVNIRSNGILDLRHGSVRDDDWEYMDARIGMMDDNRAPAPLPSHINTFLVAGTLEFEKVRNKALTVIGDYLVSVKSALGEHPNPRFQLKVPDSHKAVFYGLNHFEIQYHSSVAEQITRWLYPHVNDYVQDGIQTHIIDMPNYTLEDLEGIVET

ST25 4190 cg93_1143

VHLPDLNRSPEQVVAQVSELIESLQEVALVGSSLGGFFATYFVAKYNIPAVLINPAMQPWKLFDELFQVESMPYKVNDQWSIDHVQLRQLEQLELKQPVNADKILVLLQQGDEILDYRQAQRYYSAATPSSLILTDAHGNHAMEDFEEKLPLVIEFFAHTIK

ST25 4190 cg94_1145

MEPMVVMAARAAQTVGQELLKAHQNRHKLDLQVEEKGIDGPVTRVDRYLEQLTIDTLRKSYKNHSFLGEEFGLQEGKGHDADWCWVIDPLDGTQNFINGFPHFCISIAVQHKGVTQHGVIYDPVRDELFSASRGRGAVMNQRRIRVNVKDSLENTFLAVGHPYRAKRAGEIVSYAEQHFASLLAVTQAGAQIRRGGSAALDLAYVAAGRFDGFFELGLKPWDIAAGELIIKEAGGVVVDARGGNDSLENGQVIACSLKMLKPLMQTVVPAWDKAAK

ST25 4190 cg94_1146

MLYTEIPTQRPVTPLLDAIDHPQQLRQLEHSQLLQVADELRQYILYAAGQSGGHFGANLGVVELTVALHYCFNTPNDRLVWDVGHQAYPHKILTGRREQITTIRAKNGLAAFPAREESVFDTFGVGHSSTAISAGLGMSLARRYQKDPCEVVCIVGDGAMTAGMAFEAMNDAVAHDADLIVVLNDNDMSISCSTGGFAKHLAAIWEKGHLVNVNEHGEAYIQPHPKWTYNSRLHQSATDAADNLFKAIGFDYFGPFDGHDVTQLVQVFNALKKRKGPRLVHVYTKKGKGFAPAEADPITYHAIGKINAASGGKTPPKYSDVFGEWLCDEAAQDERLLAITPAMCEGSGMVKFAKQFPQRFFDVAIAEQHAVTLAAGMACEGLKPVVAIYSTFLQRGYDQLIHDVALQNLDVTFGIDRAGLVGEDGPTHAGAYDYAYMRTVPNMVIMAPKDENECRQMLHTAYAYNGPAAVRYPRGAGVGVEIQKEMTVLELGKAEIVAEIKANSDEQITVLAFGSRVMVALEAAEQFAQKHDVSVCVVNMRFVKPLDEQMIRDLAEHTHLFVTVEEHAIMGGAGSAVNEFMAQEQIVKPIINLGLPDSFLHQATHNQMLQDCGLDAKGILNSIERAWLKVNQVV

ST25 4190 cg94_1147

VPIEFIATSKLPTAFGEFNISVFQDPVTGEEHVALSKGLENPPTGPVLVRVHSECLTGDAFASLKCDCGPQLQATQKLINEAGQGVILYLRQEGRGIGLTNKIRAYALQDQGHDTVDANLLLNLPADARRYDMCSIMLDHLKVKEVKLITNNPLKIQALKDQGINVVDRVPLTVGRNPFNEQYLKTKRERMDHLYQKDDF

ST25 4190 cg94_1148

MQHPTSTDIQRVREFLLDLQARICAGLEQQEKAGGGTAEFIIDDWERPEGGGGRSRVLQNGTVIEKGGVMFSHINISKLPASATERHPQIAGAKAQALGVSLVIHPKNPNIPTSHANVRLFVAEREGQDPIWWFGGGFDLTPFYPDDQDVLNWHQAAYDLCKPFGDNVYAEHKKWCDDYFYLKYIAMNNVVWAVYSLMI

ST25 4190 cg94_1149

VGGLFFDDLNCWDFETCFKYIQAVGNGYLNAILPIFEKHREQPYTEAQREFQLYRRGRYVEYNLVYDRGTLFGLQTGGRIESILVSLPNLAAWSYRPEWDEDSSEKRLTDYYLKPRDWLGLEEKVA

ST25 4190 cg94_1150

VQALSASCHFKILDTRMTKQFAVIGNPIEQSRSPELHHAFAEKTGVELNYQKRLAPLDGFESSMRSFFAEGGSGMNVTVPFKEQAFALCDVLTERAQIAKAVNTLWMENGKLHGDNTDGQGLVAAIQALEWKLENTTILILGAGGATRGVIYPLVQAGAKKIIIANRTLARAEQLVDDLKTAVPQAQLQAISLNDLEGDFDIVINATSASLSGDALQLPEKLEFKYAYEMAYGKPSSFLDQAKQRNVPYAEGFGMLVGQAIEAFSIWNGVRPQLKDFL

ST25 4190 cg94_1151

MHLKYLSLCLFSLGLTACAQHGVRSQVASASLEQKAIQSVNAMYEYPSYDYRGNFKITVDPSQIKQNVKAENTAKLDAELQKKVDQYLREQKVALSKAQKQTLYAAIANEQGDLGLTSSTRSEKINTVLFNLLNDLQFSYDGSIHYRQKMGSFNLTARYEKPTLLVQAKLPMVLDLENYKFYINYFGLMPYLVNKDNQNNLAYVDFSKYKAFFKNVDKKKFIEYLKASSAVSYRLAEPQNLQRVSLTEADRKAGAVERIRLKTTVEQLLLEVDLFGQVNKKYLQKSVLGLDEEKLAETLAAEIAASDAKKGTADKEEQKVSSDDAAAVSQQLYSLVNAHLGNTSTSEDEEVESASSEEASDVAVAETEQTSENEEVVALTEDQCIELKSLKKPVALGDINYCQIYGIDVLDQSDTSIQKAQIKSRQDALKQIFEAYNQNQFINDEAFKALWLKHKDEIEQALPKQRNPITIDVALDDKGRAVNMDYDVDYTPAEFKHRFNIKADMQILNYGKATSIDQQQLKQAKSVAEASKGSMLENFIKGFSEKLGQSDVSEHPVGTHSDVQDLDANLAVLADKTYDATHAYDKTYKAVFIAKLTAEKPSYIKYYSVQQLQEIAEVYAYWFSDEDTYNPQGKALERITALQKKHHLEQDDQFDHELGRAVDHIVLTTIQGKTGREAWQKLQKQYKQPAQLFSKQYQLEFEKQNGVSAEEKHLLSETADILGNVYVAARKKQLSEKTIQNLKPEHNEFIDYEVFREVYKQMVAAKK

ST25 4190 cg94_1152

MPAYKAPLHDIRFLMNEVLDYPAHYKTLSNGESADPDTVDMILEGAADYCENVLSPLNQSGDEEGCHFDNGEVKTPKGFKEAYDQFVMGGWQGLSYPEEFGGQGLPMSLNLIKSEMMGTANWSFTMYPGLSSGCMNTILQFGTDEQKQTYMPKLVEGTWSGTMCLTEPQCGTDLGQVKTKAEPQADGTYKISGTKIFISAGEHDLTENIIHIVLARLPDAPAGTRGISLFIVPKFIPTADGGVGERNTVSCGSIEHKMGIRASATAVLNFDNATGYLIGEVNKGLHAMFTFMNTARIGTAVQGIAHAELSFQGALPYAKERISMRALSGKKDPDKVADAIIHHADVRRMLLTQKAIAEGGRSMIYYAAQLADKMTDALTRGDQAAFEDYDDKLGFYTPILKGFLTELGLEAANHGMQVYGGHGYIKEWGMEQIVRDARISTLYEGTTGVQALDLIGRKVLLSSKGKVVREYTAEILKFCAAHARNKYLRRFAWDLTKLCAQWNTLTVRIMLAARKDRDIVSSASVDFLMFSGYVMMAYFWAQQAVVASEKLEAGNGQETPEFYKAKIKVADFYFDRLLPRAQGHAESMVTTSRTLTSLPAEHFSFDY

ST25 4190 cg94_1153

MMYMSLKWSGLLIKHLGVAAILCMGLSACDVNHADSKDHSAVATTSRHEDSLIKSTTKVKTLDDAAGRTFLPEGVPDQTSKLSEADQQYAGRYHTRISCEDNFADCETGEAEYILNLLPDGTAYWNVVHFGRVGSKDGSKSAVINQLCPSLKWKVEPAAHELTIQCPLSDVNFYFDIDAKQRLVVNLEKLFYSDFGKNREFLEQNYFVPTKAYVLDKE

ST25 4190 cg94_1154

MANSGLYRSNRQSMIAGVMGGIAERFGWNANLLRIIFVLVSIMSAAFPGILVYLVLWLVIPKKQPEQVQGYQQPVRTVREEQIH

ST25 4190 cg94_1155

VSVIQPEWLQILKPNFKVLPLKERLLCGVGALCGLAISSLISWYVLGGINAWYIAPMGASSVLLFAVPTSPLAQPWNVIVGNTLAGIIGVACAQWIPDLTTAFSVAVGFAIFLMMTTDSLHPPSGAVAITAVLGGAAIHKLGFYFVFYPVLLNSLLLLGFAVFFNRLIGRHYPVTAHLNERSKDPTPTQKVSIQPKDIEYALEHHTELLDISQYDLEKIILEAQEHANERMVNQYTCQDIMSRDVIKLHENDDIHQALDKFKQENLMSLPVVNADNHLVGTLALYEVVEWFKGATDPRNSWQHYVRQIMSRRVVTVLPTQPIQDLVPYFVEKSFNYIPVVEERKLIGIISRADMIAALQQQLNRKL

ST25 4190 cg94_1156

MFNEEVAYYFDGSMVGLLSCVFRAFQFKELQVRLCLSDTAQHGLFADKIEVSNNEQHAERVWSALKKKLSSSSLKQFYFASLSESLDAYQHLFNYCIYVFSSHSSVEKDYSNPSVLAIAQWTKKVGREKHRMEAFIRFKKTKDELFLSLVRPDFNVLPLIQPHFKRRYQDQRWLIYDEQRKFGLYYDLREIHEVSLEASDVDRNLKNGMSQSFQLELNEQEVLYDQLWKDYFKSVNITERQNIKLHVQYLPKRYWRYLNEKLIEY

ST25 4190 cg94_1157

MSDRIREKLQILADAAKYDVSCSSSGSDRKNKNKGLGDASHSGICHSYTEDGRCVSLLKILFSNVCIFDCAYCVSRRSNDVQRAAFTVQEVVDLTINFYRRNYIEGLFLSSGIFKSADHTMERMLQVVKKLRLEENFNGYIHLKTIPGASPELIHEAGLYADRMSINLEMPTEIGLKTFAPEKSHQEVQKDLGLVRDRLIQLKDERQIIKHVPKYVPAGQTTQMVVGAHQESDQDVLFMADKHYKEFKLKRVYFSGYIPINTENNYLPAVGSAPPLLRENRLYQSDWLMRFYGFEVNEIVNEKHPNLDLDVDPKLSWALRHPEQFPVDLNRADYQMILRVPGIGVKSAKKIVQARRFGKIHIDLLKKLGVAYQRAKFFIRCEDSPKFQKELSSSFIRQQILTQGSSKYVQQLSPQLSLGF

ST25 4190 cg94_1158

MLKFCCKLIVLSSLFCSPWLYAQTETDEQDEYNQCVDQTIKEMKLESINNMVVHSCSEQAKKTYEEKIVALIDQIRTQSEEYKQPERYQDILKSQRLWKAYVDQECNNAGSYIGSPMYSYCPMQEYEKRVKQLEEYVN

ST25 4190 cg94_1159

MLQYNKKMIIHALALAPIPLLSLSALGVIILNAEFNLYSIGVIFLAHFLFYLLFYGLLVIPFAYIISYFLARKNRLNLMSIFISATAIWILIGPITRLIFVGSFPSPWWHIYKIYSFYLMILFTGFCYWLGLKWLSQKNK

ST25 4190 cg94_1160

MSRAISHIDTFQGLILALQNYWAEQGCVVLQPYDMEMGAGTFHTATFLRALGPETWNAAYVQPSRRPKDGRYGENPNRLQHYYQFQVVLKPNPDNIQQLYLDSLKAIGIDTLTHDIRFVEDNWESPTLGAWGLGWEVWLNGMEVTQFTYFQQVGGVECYPVTGEITYGLERLAMYLQGVDSVYDLVWTKGQFGTVTYGDVFHQNEVEQSTYNFEYAPVDKLFELFDFYESEANRLMEAKLPLPAYEQVVKASHTFNLLDARGAISVTERQRYILRVRTLARAIAQSYVQARAELGFPMAEPHLRDEVLAQLKAQAESEAAKAEKN

ST25 4190 cg94_1161

MSKHTVLFELGCEELPPKSLKTLRDALQAETVKGLNEAGLNFASVEAYAAPRRLALKIVDVDAAQADTQKRFDGPAVQAAYDAEGKPTKALEGFMRGQGITVEQLSTFQAGKVEKVCYLKDVKGQSLDALLPQILQTALDNLPIAKRMRSAASRTEFVRPVKWVVLLKDDQVIEATIQDHKAGNVTYGHRFHAPEAVTLAHANDYLAALEKAYVVANFEKRQATIQEQVKKLADEVNATAIVPTDLLDEVTSLVEWPVALRATFEERYLAVPQEALITTMQDNQKYFCLINAEGKLQPYFITVSNIESKDPTQIIEGNEKVVRPRLSDAEFFFLQDQKQPLASRKEKLANMVFQAQLGTLWDKSTRIAKLAVALSSITGANPADAEKAALLAKCDLTSELVGEFPELQGIAGTYYARIEGENTEVSEALGEQYLPKFAGDILPKTKTGTTIALADRLVYAGWYFWYWTSAYRF

ST25 4190 cg94_1162

LEGRYRAKYEDQGVAVDVLQAVQALAPKSPLDFDKRVNAVNHFRTLPEAAALAAANKRVANILAKEAAPEGSVVEANLVEDAEKALFAELQAVTPVVEPLLAAKDYTAALSKLAALRAPIDAFFDGVMVMADDADLKANRLRLLAQLRNLFTAVADVSVLQG

ST25 4190 cg94_1163

MIKYISIENFRCFHQSTFKEFSHVNLIGGLNNSGKSALLEAILLLLSPQLETITLLNKYRNELVTKKNTFDKTWDYLFFNANKLEPIKLKTLSDRYNYEYCLAIKKSENDQFNNVAFYEKLKNSKEESLERAIKDFELFTQKQNIIHSLDLYITDDELNNSFMGSFSFTDSGRLTMLETPTYLFNEVAYRPAKQVITPNQLSKLLDISVDKGHLNEIMETIQIIDDSIIDIRVVENKVLLSRDRVSYLPISLFGDAITSTLYMILTLFSVDEGGYLLIDEVENGIHHSKQLNFIRHICNLAFKRNIQIFMTTHSAEFIKSFNEYSLENEGTKLRYLELVRTRKNRIISNIIEPEVLEYKINHSKNFRGE

ST25 4190 cg94_1164

MNHKIIVESFNDQAIYAHVLKNFCTSNTDVETFTDGLDWIELAGLERTKLITKLKDIRSELIRAQEITKIGIIIDLDDSSVEERIKFLNELCSEAFELTIDIEKVSEFKLYEIPEQDIEFKLAYCFSGLNGQGELEHLLKEIADTSNSHHANCLETGWKQCLASKGIIVRDKDLRKLWMDFYKRMDCLSHKERKQAKQNVQWDTFLSLYPEKFDFSKDIPELNDLRQFLTFFG

ST25 4190 cg95_1165

MDGVEALQTYVDKLEEAGVNQQQMSFYLESMGSDLTGLIPILQDGGKLWKEYQSAMEEAGIITGEEAIQKSIELKAQTEVLQMQYTGLKNQLAQAVMPALSGVISHFMNGTTKGGAFTGVIQTLGSVAKGVAVVIVGLGAGLQNLVRLMSGVMSNLRTIGSTAVNFVNADGILAKGKALAGGVKAIWTETKDTVVDIAGTTKAAINSASNIFSGTPSFDRLTQAKIDIQNAQLGARGGSKGVTSGIGQNKALNPDGGKSEKAKQGKSDAVRQAEQAAKALADIRYKYASEEKKVALDLQKALDEIEKSKMTADEKAAAKVKAEKDASDKIIAIRLKEFEEYKKAREEQIDNYQQQAQRLYEIEAARIQAEFDAKKISNVRKVQLEKQLEDQLREIKRQGLLERLALENEQTNIIGKQGNQNQITNNISDLETDQKVADTKSMGLISDAEMKDFEAKFGGFTSRLSNLWDQGIQSLMNGTLTWSNATKAVLADMGQFALQTATKELQGWLRIQAIKLARKLGFVGAETAAEASGQAAQTGATIAGEATRTSVTAAGGLARLGLKAAEAIKGIMMSAWEAMAGAFKAMVAIPYVGPILAVGAGAAAFGLVAGLAGKIKSARGGYDIPSGVNPMTQLHEEEMVLPKQHANTIRALGKSMANAGFADPASASGGDSYHFNLGFVDTKGADRWLKKNGKAVANSLKGYNRNFGK

ST25 4190 cg95_1166

VGGTIAATAGLATLAIEVANNNVELARFSALANTSIQSFQGLSGAAQTLGFSQEKLSDMMKDFNEKIGEFASVGSGGAKDFFEQIAVKTESGAEGAKKARRRNVQDGWGRSLTNLCR

ST25 4190 cg96_1168

MLKRSIAFALLAAAGHAYSADIEVTTTIDEDVDNTVCSLREAVELINKRNSSDSTVVASVKDGYHGCGNKDSSSNIILQRDKEYTLNSRITITAPLTISTAKNDSTLVDTDQPGSHNATIKMAGTDQLFKIDDESVEKLSFAVTLSDLNLQGARSSNTILEGGLIYNHEQLTIRNSRLINGYATRGGAIYNAGNLSNTLNTAGTVTLVNNLIQNNKAAEGGILYSEMPLYFITQSVLRDNEVTTADNALFYTQTKFSDESTGGYLTSRAIGISNSTIFHNKGGFIANVRDGMFVNNITMIKNDKGLFLEAPQGNASVSNSILVGNTINCQASSTDKAIIQSNLVISDCNRNATSKLPNILFPADEKLIAGDNDEGICDVTAKDGLLCPFNIPKDSFLGFFKPRLLESYNTLADSLIINKGRLYSDGSSVGLASCERLDQRGKNRSGYDELCDLGAIEYILNNEIGTVGQDIKYGQVARFNILNSLSEADLVTPATCKRLFGERPDGKEWQPGCLKIKQSTDTPFSKGSVTIDQNGNVIYTPNGNWHGADIFSIQVVTSVSRFNDGSDFQYISIPATVVQEPVSGIEDKSVSTGGGGSVGGGLILGLFGLIALRRFKS

ST25 4190 cg96_1169

MRQLQLDIEPQLDARISDFSGPGWGHVVDAVRQLHAGLVNRFYIYGGAGTGKSHLLSAICDSYLEVGKSAIKVSLLELLDAPIEAITCLEHYDLVALDDIESISGVPHWQKAVFHLMNNHEGQLVFSSRVAPIELKLELPDLQSRLTQAVSVKVPSGSLYADRYALVTSVMARRGIHFDQQIVDYLLLHGPHQASVLLQTVAQLEKLLKGEKTKLSNATLRQIYALIDEYQQ

ST25 4190 cg96_1170

MQDRILRRIFLLAGIVLLVWVLYLLKPVVIPFIGAFFLAYLFSPLVEVLVKIKIPRWLAISIVFIGIGVTLTVALWYLVPLIWKQLVYARDSIPAGIHWINAELLPWISSTFHVQQMEIDTDQMSKAVMDYVQTNYSADSIQAVLLKLAQSGLNFIQIGGTVVLIPIIAFYFLLDWDRMLQNLRRLIPRPYEATTLQIVRECHSVLGAFVKGQFLVMLLLGVVYAVGLQLIGLEVGLIIGMVAGLASIIPYLGFAVGIIAAVIASLFQFGMDWTHLLLVGVVFMIGQAVEGYILQPFLLGDKIGLSPVAVVFAVLAGAQLAGFLGMLIALPVAAVIVVLLKHLRENYERSSFYAPSSTLLIQEGNIEQLHVETENTKLDLEVKKQQDTDTPNLSDQK

ST25 4190 cg96_1171

MSNSTSTPNTGLSYKDAGVDIEAGDALVDRIKSVAKRTTRPEVMGGLGGFGALCKIPKGYEEPVLVSGTDGVGTKLRLALNLNRHDTIGQDLVAMCVNDLLVCGAEPLFFLDYYATGHLNVDVAANVVTGIGKGCELAGCALVGGETAEMPGMYEGEDYDLAGFAVGVVEQSKIIDGSKVKSGDVLIGVASSGAHSNGYSLLRKILDVKNVDLTQVIDGRPLADVAMEPTRIYVKPVLELCKQVDVHAMAHITGGGLPGNLPRVLPNGAQAVINEASWEWPELFKLLQREGNVERFEMYRTFNCGVGMVIAVDANDAEKAIEVLNAQGEKAWKIGHIQENAESVEGADEKIRVIFE

ST25 4190 cg96_1172

MMKIAVLVSGNGSNLQALIDANLSGQIVGVLSNKADAYALERAQNANIATAVISHKDFPSREDFDEAMHQQLIAWQADVVILAGFMRILTADFVNKWQGKMLNIHPSLLPAYKGINTHQRVLNTGDRLHGCTVHFVTSELDAGQAIAQSAIEVKEHDNVASLAERVHKLEHFIYPQVAEWLCNGQLTWKNGQAYFRNQILEHPIRFASW

ST25 4190 cg96_1173

MSVKPVIHFAHANGVPSMVYQKLFDQLKDEYDVIYVPLIGPDKRYPITNHWPFLVDQVIDSIVRQAKGRKVIGLGHSLGSVLTLMASFRRPELFSQVIMLDPPLILGRYSFAFHMAKLFKPKIVDDMTPAGLSARRREHWESREQAAALLRPKGFYKDFDEDCFQAYIDYALKEDSVRGGVTLTISREDEVAIFRKNPSWWWLPMPKPKMPVHLVVGKESAFLKRGFPQMAKRKMGIPFSVVEGGHMFPLEHPIDTVNYIKELIHRNSR

ST25 4190 cg96_1174

MKSYYLDSFYQINVYNDIISIDLDWNFMSDWPPKPQNEPITPKQNGPEWQILEKAVLASVEEQRRSRRWGIFFKTLTFIYLLFIIVLMGKGCSTTKDGSTASSSSAHLAVVDIIGTIDASSNQAVNSEDTNKALKRAFEASNSKAIALNINSPGGSPVQSDEIWQEIRYLKKEHPDKKVYAVIGDMGASGAYYIASAADEIIVNPSSLVGSIGVIMPNYGITGLAQKLGIEDRTLTAGTNKDILSMTKPINPVQRQHIQSVLDNVHTHFINAVKEGRGKRLKTNDPAIFSGLFWTGEQAIQLGVADRSGNITSLMRELKLDNKVDYTIERNPLQSILGRMGSEMGKGLSESVAERLQTSQDAKLQ

ST25 4190 cg96_1175

MNYQLLKTFSRQPIQFGRFLARLLAGLVNTLKITRTSKSIELNLRIALPYLTPQQRIAITEKAVRNELTSYFEFLSIWGSSNSKNISRIHRIEGEHFFHEALAAKKGVVLIVPHFGTWEVMNAWCAQFTSMTILYKPVKNADADRFVREARSREQANLVPTDESGVRQIFKALKQGETTVILPDHTPNVGGDMVNYFGVPLASSNLSAKLIQKTKAKALFLYAIRNENDGFTMHIEPMDEKIYEGTADDGTYVIHQAIEQLIYQYPEHYHWSYKRFKANPALDNIYNIDPTEALKIVDRLKAEALKTSTQPEPIQTSVM

ST25 4190 cg96_1176

LGWIGGIALMGIDFPLIMQYEKVGEALLLLAFIFYLYKRPMFVDRPFLKAVFCLLCTTSLFMVGYHYAEKALIERLEQRETDTRNLDIIVYINRLSEEKDNKVQQTAQVLNFSKEPVNWLLYLKSNNQNLLKNNQNLELGHYYRISGKTRPAHSYATPGAFDQEKWFIQRNIMSGFNVRYIEPLSLDEIYRLGYQQHLKEQQSFSNSFRLNIEKLRLTFRQILNSSSLQQKGLILALLTGDESLLSDETQLQFKQLGISHLLAISGPHVLIFAIMLSWACHQFISRYYPQIYLWKPKQVLMTVPCCLGVLIYTAFVGFEIPALRTLLSALIFIGFLLLKQPIKPFTLLVYSASLLLLIDPFSVLSAGFWLSYGACFILLRIYQTIAQLPEQHFLSLSSKMIFMGKVLIESQGKIFIALSPLTLLFFQQISWVAPLTNIIAVPIVGGVIVPLNIMAACAWFIVKPFGNMLFHFNDMLLSILLSCLGLLEKLSLPLQGISLTPLSLLAISCAIIILFLPKGILPKTWGILCCLPLVIMNKTSQQIQLNILDVGQGQAIFLQHPQQNWLIDTGGSYDEKIFSIGQNVVVPFLRQQGVRRLDHVVLSHLDQDHSGAFPIIQQEIPVKQLISNEQLPNDLKQPFQYCHQGQQWHYPELDIQILWPKEKDLAFVTSNQNQYSCVVYLQFKKVGGYQNFLIMGDAGWEAEYELLKDYPNLKIDVLVLGHHGSKHSSAYDFLATLKPKLAIASAGFDNRYGHPSQQVIARLKALHIPLKSTLEQGTLSFVLENHKLVLHDRRLDRLWLSRGF

ST25 4190 cg96_1177

MSKVVLEAKDIYKHFDDGKSKVEVIKGLSLQVEAGQFVSIVGASGSGKSTLLHVLGGLDQPTKGQVFLNGQRFDNLGEAERGFQRNQYLGFVYQFHHLLPEFTALENVAMPLMLRADSQYKSVKAQAEYLLDRVGLSHRMDHKPGELSGGERQRVALARALVTKPAVVLADEPTGNLDRKTAVGIFELLTDLKKELNMAMLIVTHDEQLAQAADSILHMEDGLWVNGS

ST25 4190 cg96_1178

MVGLTLGVAVLITVLSVMNGFDRELKNRVLGMVPQATVSSTQILTDWPELVKRVENHPHVTGVAPFTQLQGMLTAQGQVAGIMVTGIDPKYEKNVSIIQNHIVAGSLDSLKKGEFGIVLGKDMADSLGLRLNDSVTLVLPEATPSPAGVVPRFKRFKVVGIFSVGAEVDSMVGYIALYDASTLLRLPDGAQGVRLKLDDIFAAPQVADDIVKNLPSNFYATNWTYTHGNLFNAIQMEKTLVGLLLVLIIVVAAFNIVSSLVMVVTDKKSDIAILRTLGASPSMITKIFMVQGTVIGVIGTVAGTVLGVILALTISDIISWFNNVLGLNLFDAYFVHYLPSYLRWQDVTIIVIVSLLLSFLATIYPALRAAKVQPAEALRYE

ST25 4190 cg96_1179

MPSQTNLASTYVLLVFIWATTPLAIVWSVSDLHVMWALLLRFYIALPLSVIVLLVLKTSFPTHKQALHSYFAGSFSLIGSQIFTYAATQYLSSGMIALMFGLAPIMAGLIGRFAFGQHLFKLQWLGMAVAVCGLAIICLNGSNQHMHPFGIVLMLVSVFVYSFSIFWVKKVNAKIQPMAQATGSILVSSILASCLIPFIWQYAPTHLPQAKSLFALIYTVLMASLVAMFCYFKLVQNIKPTTLSLTTVITPMLAMIIGAVLNHEQLSIMVFVGAFIILFGLFLYFYKDIQANRNFAQHIKSK

ST25 4190 cg96_1180

LWVDADALPKILREVILRASDRYQLEVIFVANQNVGITPSVRIKSLQVLSGADQADREIVNRMSENDIVITQDIPLAAQVIEKGGIAIHPRGEVYTTANVKARLHLRDFMDTLRGAGVQTGGPPPISERDKREFSSALDQTILKQKRKTA

ST25 4190 cg96_1181

MKNQNSPEVITVNDQNFGSHGEHWNLLTSHPETDVPKWLGLALDAPVMPMGLCQNEDEMDQSFWLIQGPQGQKVTINQIIAVENQKPRALKTAFPSFDSPYQYNAQIERIITCDSATQAVLSLKLNKSTTIYAFDNLFSVNRCQYDKTQTYQVQFNAWAYELESVPKGETIVVDDPASIKHHRALNAILTEHNGIAPENLQELINEWQPKTKEDQEPVTVDFSKMVAYLYGENLGQEDEAWFQGNIVGKTSMSFMGAEYTLYDVTLVLEDNLPAILVRIANKK

ST25 4190 cg96_1182

MICNKNFNIGEYIRGNIWIQANIYAKNSETN

ST25 4190 cg96_1183

MRAYNFCAGPAALPTAVLEKAQQELLDWQGKGLSIMEMSHRSADYVAVAEKAEADLRKLMNIPENYKVLFLQGGASLQFSAIPLNLLGKNNKADYIHTGIWSEKALKEAKRYGDINVVEAGIKVDGKFAISEQSEWNLSDDAAYVHYADNETIGGLQFAGVPDVKAPLVCDFSSSILSAPLDVSKFGLIYAGAQKNIGPAGLTIVIIRDDLLDQAKAEIPSILKYADQAKNGSMVNTPSTYAWYLSGLVFEWLLEQGGVDAIHKVNLEKAQLLYGYIDSSEFYNNPIAIPNRSIMNVPFTLADEALEKQFLKEAEANHLLNLAGHRSVGGMRASIYNAVPLEGVQALIRFMDDFAKRNG

ST25 4190 cg96_1184

LLKVAAASLVNISMLGRVYAYFFNNYFIYFIAFTYFFYKFY

ST25 4190 cg96_1185

MPTHQYRQSKRPFLFKPNYLALVLGVLIATPIYAQSLSYAEAEQQALKSSYSTQANQALQQASQLEAEAVKGLGLPRVDLNVRAYAFHSETDVPLGSFKQKLENDLSQGLNDKLSQWNNVIPSDVLGQVQEGSNQIIHDGINRFPDYANLTVEDQVVRPSISVVMPLYTGGLTTSAKK

ST25 4190 cg96_1186

VQLQQQLVASSLFNFNAMQKHYSNALKLEQQGFISKGQRMQFEVARNNAERTLQNAQANLNASQFNLNNLLHQQNNADLSTPLFVNTVRSQSLESLLSSYSQKSSLVQKMQLDTQLANANIQAQQAAKKPSLFAFGEYSLDENENWIVGVMAKYNLFSGVDKNKNIHAAELKRYASELMTERTKQEIEALLNKSYNELNSAQQSHTLLQRNISAAQENLRIQELSFREGMGTATQVIDAQNALSALKTEMALNAYKYVMSLATLLQSHGSMDQFKAYVTQPHTDYIR

ST25 4190 cg96_1187

MNQDSAHSEQNSPVADTAETNQNQQNTQTDNTQKVKTSKSDSQPPKNSKIKLIIAVVVIALLGLIIFGLWKSYQPKPIELQGRVEAETVHVSTKVPSRIEEIYVQEGQKISKGQPLVRLVSPEIEAKKQQALATLQSALAFQSTVDRGSQQENIDTLYANWQSMKAQANLAKTTYQRGENLYRQGVISRQRRDEMLAAQTSAQELSEAAYQQYARAKRGSTSQQKSTADAQVEIAKAAVSEAQALEAETRLLAPISGTVSKTYGKPSELVAMGVPVVSILEDDDLWVSLNVGENQYASVYKNKTLEGFIPALNQTVMFKVKNIDAEGEFATIKTTRQTGGYDIRSFKLHLVPERPVKDLKVGMSVLFKIKEEK

ST25 4190 cg96_1188

MFYAGKAEHLPIAIIDQDQSELSRNIGKYLSLNHTVDVKTITTSSSEAERLLNETKIWGYIFIPDGAEKRFVKAQDAQISIAFNQSFFSVGNTISSAMLVSTLNALADYAGQNYLANNIPYLDVPTAHVKISTLYNPSMSYEFYLEPFMVPAVLHLLLCCCVAFAIGQEIKRGTLTQWVNRESFIQGLLAKNLLYSFIFCFWTWLWMFWLVEIRGWFVAGSLSFLLAAQFLLYFSYALISSTVILATKNLSKTFGFIAVYGGSSLSFAGVTLPLNNAPIFTKFWSLIIPYTPYAKLQTEQWVIGSPLFISMSPFLILIGYCLIYFFLSCLLLKKLVQGATS

ST25 4190 cg96_1189

MKEFLKAYVQTFKDIVSNSSVFTTLILSVILYSFFYPTAYKAERAESIPIVIVDEEQSLLTSQVIGQTANSPHVKIVDVTGNFLEAEQMVREQKADGILLLPSNLTQSLRRGETGGIGLYLSTTNFLKTKEIGLGLATSIEATLKEYIERFGQRTHFQPALSIHQMPLFNTLSGYGSYIFPAVASLIIHQTIVLGLAMLVASYREQHEKITPIRFAGIFASIFTIGCLGSFYLLVLPCGSMAIHTVETLSVC

ST25 4190 cg96_1190

LWFNGYPHGGNFVGLLVAVPIFISCVIGLGMLIGSLLDMPERAGHIIVFSSVPLFLLTGAAWPHQAMPEWLQWFAWCLPSTHGVQMFVQLNQMGVPLNVVVPKLIFLATIGVIFLITAYSRLKVSK

ST25 4190 cg96_1191

MAKVCYCADDFAMNAEISDAIIQLIEQGALQATSCMTQSDLWETAAAKLKPFSDRVDIGLHLNLTHTFASGNLVFPLPMLIVRAWSASLNRELITQCIEEQWDLFVSVLGKQPDFIDGHQHIHQFPFIRDILLQLLKEKKFTGWIRNLQQPINPPPYRFKTRMLSALGSNSLAKACQTYHFNQNGQFAGIYDFKLTNYGQLNQYWLANAKDHLLIMCHPALAQSKDQDPIQHARIQEYQYFSSDQFQLDCQQYGIQLTRLGAMQ

ST25 4190 cg96_1192

MNIKPFLSCVVPAYNEAENLKTFIPALANSLKQQNLSYEIIVVDDGSKDDTIPILQTMIKDYPLVVLELSRNFGKEAALSAGLDHVTGEVALLIDADFQHPFEAIPTMINLWKNGYDMVYGIRNRTTESWLKRVLTQAYYRILNLSSPIDIPESAGDFRLLDARVVEAIKQLPEKNRYMKGLYAWVGFKSIGINFSEQERQHGRSSFNLKSLFNLAMSGLTGFSDLPLRVCIYLGAILALGAMSYGVWIIIKTLIEGISIPGWATLAAGMTLLGGIQLLFIGILGEYIGRIYAEVKNRPKYIISTEHSRTKQEHSIAHQENTFSSF

ST25 4190 cg96_1193

LVAGLGFLFNESFLLLSNLYFNVPVSTLVCTAIVLTSMVTFLLNRFFAFQH

ST25 4190 cg96_1194

MQQWLRSSKFLLIFLPIWLFVLSWIRPLSVPDEGRYGDISRTMFESGDWLTPRIDGLPFMHKPPLLHWLSSMFMELFGVHVWVLRLVPVLAATLMLVGLFLFVKKHISESVAQLTVIILATNLLFFGSSQYINHDLLLASWITISVLCFVDFTISARKSILFLGYIAGAAAFLSKGLIGILIPGMILLPWLIYTKQWRKIPSLLNPLAILLFLLIVSPWLYLVQSKYPQFLHYFFIDQQFNRFSSKEFNNKQPWCFYLMILFVSFLPWLFASRFTSIKTIFKDYKSCSLLALFVWWFVSVTVFFSIPPSKLAGYILPAVPPLAIFFALVMNKVLESSNKTRLQTWGIPVFTVLVGIGVSATPYFIRVHQPFFQNQAIFIYLIGALLIVLPLVLVGLYKKQKLNYLTYIFISLIVLCSAVPFAVRIFRYQKQCRPNRFC

ST25 4190 cg97_1195

MSYHHILVPVDGSPTSLIAVNHAASLAKAFSSKVTLVYALTIDPFISVEIIDSTEIAQDYFNKARASIQSILDQAKEQFSQHGISVETKIVEGQTIHTEIIKAATELKADLLVIGSHGRKGFKKFFLGSVTQALLGEIHVPVLVVTE

ST25 4190 cg97_1196

MTSMSNTTQKHFIVEQFPLPTWVSDEKGLILYCNTECTKYWRDTSPPLFQQWLDFVYPADLEEVKNNWLDAIRLRSRIEFKCRLLHKTHYYRWCKISIQLSKYHESSQENEWFISFLDIDQDIQEQQSLRKDIDIQNQMLDISVDCIKVLNTDGTVSHMNKSGCLALGIPVDEKKFGMRWLDLLPPHIRKRGRVALKKALQGKVARFAGMSCLPNQDPEYWDNMLTPIQHTNGEIIQILCVSRNVTQQYLTENRLRDISERDELTGLFNRRSFKLQLKRTLAYSRETQTSVGLLLIDLDHFKHINDTIGHSAGDHLLKILSKRFNQCVDPERCSVARLGGDEFAVIVSHLKNESDVRELAEVLLQQLNQPITYAGNVLNGGMSIGCAIYPQDAVDQSSLLSCADMALHDLKARGRGGIRMYNPSMMQLTEAIASQLNIARQLIRHHTIQPYYQPKVDLKTHAVVGFEALLRWHTTSAQRGLPSQIAEAFKDYDLASKIGEIMQQQVLHDIAKWIKLGFVPLPVSLNAAPVEFLRDNYAERLLKKINQLEIPTHLIEIEVTEHMLGDRGSEYVIRALNKLKQHGVRIALDDFGTGFSSLTHIRDYPIDCLKVDCSFIQKMQHDPSIYAIVQAIGLLAPNLSLGLIAEGIETPEQEELLLQFGYNIGQGFLFESAIDANQVISILKQGQAYLESHYSMN

ST25 4190 cg97_1197

MTKKYARFLPTTESFTLEDFPYYWITQVHAQYVQNIDNALKKYGLDNSRRRIMLALSSKPHASVSELSDMIISKMSTTTKIVYRLKDEGLVETYSCQSDGRITRVYLTERGTEMINKINDLTSVVLEQSFEGITPLQLEKMMESLKLLLKNLSR

ST25 4190 cg97_1198

MQTAKHLLFVTNSPQQQVNALILARKLALVATQHGYKHSVISLDEFESSDHFDHVIIIGQQPKNLNIFGQNALSLVSIEDIKDDADKALLTALEHSKPANEWEQKPKQASNTATHFVAITACPTGVAHTFMAAEALQQGAERLGYQIDVETQGSVGAKNILSPQAIADADIVILATDIEVNTDRFVGKRVYRCSTGFALKQTDKAFAEAIANAQVLEQGKQQATTENKDKTEKVGVYKHLLTGVSYMLPMVVAGGLLIALSLCFGLNAAEQAGSLPAILKQIGAAAFTLMVPMLSGYIAYSIADRPGLAPGLIGGLLAAQLQAGFLGGIVSGFLAGYIALFIAKKVKLPTSLESLKPILIIPLLGTLSVGLIMFYVVGQPVAHIFELMKDFLNNMGTTNAVLMGIILASMMCIDLGGPINKAAYAFTVGLLTTNTYMPMAATMAGGMVPAIGMAIATFLARNKFSTGEKDAGKAAFVLGLCFISEGAIPFAAKDPMRVIPTCILGGAVTGALVALFHCELVTPHGGVFVLLIPNAINHAWLYLAAIAAGSIVTGVSYAIIKKNQEEKLLTNS

ST25 4190 cg97_1199

MAKILTITLNPAIDVTIQLNELLVGEVNRQESVEIHAAGKGLNVAQVLKDLGHDVIVTGFLGEHNRQIFDTHFAQAQFQPEFIYIDGETRQNIKIAEHSGRMTDLNGKGFFVSETDKQRLFNKIEALLPQVEVVAIAGSLPQGFSIDELQQLIQLIKKNNKKVALDTSGKALVAAIECQPWMIKPNTDELVESYQRPATSYAEQRKLFDSLTNIEHVVISMGEDGVNWLHAIHPLHAQAPKVIVKSTVGAGDSLLAGMIHGLVNQMPPEETLKTATAIASHAVTQIGFRIPTSEVLNQLKAQTIINSLSESDANC

ST25 4190 cg97_1200

MLALEPQHIHMNQHAVDKTHALKCLVDILEKDGLVTPEYITGLINREQQSATYLGQGIAIPHGTPQSREYILKTGIRLAHFPEGVIWDGENKIYLAVVIAAKSDEHLQVLQILTRALMHDVSEQVKNASKPEQIIELLQAQPLSLALHENLIQTEIDAQDIEDLFWAASQTLKKHNFVKCGFLSSLDPEQAIQLQDQIWSISSNHFVQQPAISITKPSQALELNGKKLNTLVCIAANDQLDNERFNRLIDILFNPEQVAELDKTQTPNEIAKIIGADVVPDWPQRSVVLANAHGLHARPATHLVNLTKTFQGDIQVAVDDGSFVSAKSLTRLLALGCKRGQTLRFIAEPETDAVEALDQVILAVQQGLGEEVEALPVTSETPDQLPAQTAISNATTLGNNTGIAASSGLAFGPVHVIKPKVYQYERMGSGVKVEKENLDIALHAVKNNIHQVIAKADASEIKQIFRAHLEMLDDPDLINGVYQKIYQNLSAPAAWHEHIEAAAKAQEALPDRLLAERATDLRDIGDRVLTQLCGEVVIEEPKEPYILIMHDVGPSDVARLNKDRVAGILTAIGGASAHSAIVARALGIPAIVGAGEQVLEIDPKSTLLINGDTGTFVLNPDAAQIEQAKQEREQQRQIREEAERHSHEPAITLDQHQIEIAANLGKVQATAQAVECGAEAIGLLRTELVFMSHSSAPNEAVQEADYRVVLDALAGRPLVVRTLDVGGDKPLPYLPIAEEENPFLGLRGIRLTLRRPELLKQQLTALLKASDNRPLRIMFPMIGRVEEWRAAKAILDEVRAQHPCDNLQVGIMIEVPSAALLAPVLAQEVDFFSIGTNDLTQYTLAIDRGHPILSAEADGLHPSILNLIDQTVKAAHKHGKWVGICGELAADPKAVPILMGLGVDELSMSPNSIPLVKAQIRTLSYSHAQKLAQQALLCDSAPAVRQLSEQDL

ST25 4190 cg97_1201

MDKTFESFGITCKDGYQLAAQFYPAQEKKAEYPILICPATGITKNFYHSFATWLSQQGYDVLSFDFRGIGESLHGALKQSTASITDWGTLDIPAAIDALLIKTKANQVILIGHSAGGQLLGVVPNYNKVAKVITVAGSTGHIKGLKGKTKVLAPVMFNLIFPISSLVKGYGATQFIGMGENLPKKVAQQWREFCSRPGYVKNAIGKTIFHDFHSDIQCPITAIWADDDEIATKRNVQELLSLYPNANKKMIELSPKDLGYKSIGHMLLFKKSHQKLWSILEQEIKH

ST25 4190 cg97_1202

MEIMDLSKAVQLAIVFSGVFLWIGMLTGVWKYWQIRRSELSRAHYYVDIAHRSSLLYAAATLILAVLSYFTILNEDIALWCVLANILFFSFSILVYIIHGFLQDTTNQFKQPHRLGHFSLPTWLMTFMMFALIITELLATAILVMGTILLFLSK

ST25 4190 cg97_1203

MKSKYYLTLADAEFLMEEAQKYAIEHNFKVSIAIVDETSSLLLMKRLDGASPLTTHLCLEKAKCSSMSGRPSKFYEELLQNGRLGFLSMPSAKGMLEGGKAIIYEGQILGAIGVSGVQSFEDAEIAQHAIDVFLKKQSS

ST25 4190 cg97_1204

MQQLRTGDIIALGFMTFALFIGAGNIIFPPIVAQQAGDHVWLAAIGFLITAVGLPVITIMALSRMQGSIEIISSPLGRIASLILTVVCYLSVGPLFATPRTATVSYEIGFSSYFGTSSSSLLIYSAIYFAFVTVVSLYPNKLLDTVGHVLAPLKIIALAILGIAAVMIPAGYVPAPINHYVNSPVSEGFVNGYLTMDTLGALVFGIVIIQAIYSRGVTDNKLVTKYAIIASLISGVGLTLVYLSLFKLGLGSHEVAPNAANGAVILHAYVQHAFGNMGSLFLTGLIFLACMVTAIGLTCACAEYFAQLTKLPYKLLVFILVGFSFIISNLGLTKLIAVSVPVLSAIYPPAIVVIMLSFFWKFWHKPSFIVGSVTSIALIFGIIDGLKVAGFGDYLPNFLQHLPLNEQNLAWLIPSLVVLVVTTLIDKVKHKSI

ST25 4190 cg98_1205

TPWAGAGYGARFLPRVGEIVVIDFFDGNVDRPFVVGRIHEAERHPTQFDQKGQLPDTKKLSGIRSEEVDGKGFNQLRFDDTTGQISAQLQSSHAASQLNLGNLSHPKDKAESDGRGEGFELRTDQWGAVRAGSGLLVSTHKQDQAQGVHLDASEAKQQIEGGLNNAKALSEVAKNQQTDPLEMLENLKTFIEQIEEKDQDKAAAFKQALMILTAPNSIALASNEDIHLSADGQLNQTAGDSINLSTQKNLIAHAQNKISLFAAQQGARLYAGKGKVEIQAQGDGADLIARKAVQVISTEDKIEATAAKEIVLTAGGSQVKITGSGIFMTTSGKFEVKAGQHLFMGGGSVNSSLPYLPEQGKQKYGVWFDVMDKQGNKLKPGTEYIIFDEHDKEIERGKLDRTGLVKLETEEPNKQYKIHVVN

ST25 4190 cg98_1206

VLTPWAGAGYGARFLPRVGEIVVIDFFDGNVDRPFVVGRIHEAERHPTQFDQKGQLPDTKKLSGIRSEEVDGKGFNQLRFDDTTGQISAQLQSSHAASQLNLGNLSHPKDKAESDGRGEGFELRTDQWGAVRAGSGLLVSTHKQDQAQGVHLDASEAKQQIEGGLNNAKALSEVAKNQQTDPLEMLENLKTFIEQIEEKDQDKAAAFKQALMILTAPNSIALASNEDIHLSADGQLNQTAGDSINLSTQKNLIAHAQNKISLFAAQQGARLYAGKGKVEIQAQGDGADLIARKAVQVISTEDKIEATAAKEIVLTAGGSQVKITGSGIFMTTSGKFEVKAGQHLFMGGGSVNSSLPYLPEQGKQKYGVWFDVMDKQGNKLKPGTEYIIFDEHDKEIERGKLDRTGLVKLETEEPNKQYKIHVVN

ST25 4190 cg99_1207

LAGQEAYREPLRLFAGHFGNAFQIIDDILDYTSDAETLGKNIGDDLMEGKPTLPLISALAHSTGEEHAIIRRSIATGGVDQLPKVIEIVQKSGALDYCQRRAQEETEAALQALSILPDTPYRQALINLTRLALHRIQ

ST25 4190 cg99_1208

MHINFSEHFFKIQKQLENPQAVIDENILIDLVNALRPSDPQDTDEIEQKIQAFIDSLLLTPTAPALLQTFLLRLINQYKQVSLYADSGILSLDGFWNQLGQRLGGHFLPLIEDASQLKILIGKIFYLESDSIWLNNVDDKDWATLFGLIGQSNSNVDEKHAIQREMIKAITVLSYRISGIGLYPEFINAQPELTEYESPFLVQNREIIEFIEKYKKQDISSNDIAVLPPPDASQAFVMLEQCRDVVLKIRRATKRIGVSLSLTYLLSLLEQCLDRIELLLYLVVDDSEGRYVSLGNLISDLTKAHYSEKSVRSLLSTTSELIAFQVTENASRTGEHYVSTDTKGFWGMYKAAAGAGVIIACMASLKILAARMTMAPLMQAFTFSMNYSLGFILIHVLHFTVATKQPAMTAAALAATVQQRKGSKTAQIAELAALIINIIRTQFIAILGNISIAIPTAALITFAWQFYLDEPLLTHTKATYLLHSLNPFTSLAVPHAAIAGVCLFLSGLIAGYFDNMAVYRKVGPRLKAHRRLRNLFGQERLNRFAEYIERNLGALAGNFLFGVMLGSMGTIGFILGLPLDIRHIAFASANFIQGLMTINGSPDIGLIIVSFLGVLCIGLTNLFVSFTLTIIVALRARRVRFEQWKPLAKLVMTHFLTRPSDFFWPPKQPLELEENAQANSGKKAEH

ST25 4190 cg99_1209

MPYLLLCIGCVFLGLGVLGLFVPSLQSLDLLTVQTLSHHRLDYLNTITTFLARVGGMPFVCFLSFLVCIYLAWYKKYITVIFISLGVIGSITMGWLLKWCVNRPRPPEAYHIVESYGASFPSAHSVYASTLACLAMIMLCHKHNINSPYIVLISCLWFVCMGLSRIYAGVHFPTDVLAGWGIGFIWIALLWLWLLQTQSRLSRKQIYF

ST25 4190 cg99_1210

MMSAKLWAPALTACALATSIALVGCSKGSDEKQQAAAAQKMPPAEVGVIVAQPQSVEQSVELSGRTSAYQISEVRPQTSGVILKRLFAEGSYVREGQALYELDSRTNRATLENAKASLLQQQANLASLRTKLNRYKQLVSSNAVSKQEYDDLLGQVNVAEAQVAAAKAQVTNANVDLGYSTIRSPISGQSGRSSVTAGALVTANQTDPLVTIQQLDPIYVDINQSSAELLRLRQQLSKGSLNNSNNTKVKLKLEDGSTYPIEGQLAFSDASVNQDTGTITLRAVFSNPNHLLLPGMYTTAQIVQGVVPNAYLIPQAAITRLPTGQAVAMLVNAKGVVESRPVETSGVQGQNWIVTNGLKAGDKVIVDGVAKVKEGQEVSAKPYQAQPTNSQGAAPNAAKPAQSGKPQAEQKAASNA

ST25 4190 cg99_1211

MAQFFIHRPIFAWVIALVIMLAGILTLTKMPIAQYPTIAPPTVTIAATYPGASAETVENTVTQIIEQQMNGLDGLRYISSNSAGNGQASIQLNFEQGVDPDIAQVQVQNKLQSATALLPEDVQRQGVTVTKSGASFLQVIAFYSPDNNLSDSDIKDYVNSSIKEPLSRVAGVGEVQVFGGSYAMRIWLDPAKLTSYQLTPSDIATALQAQNSQVAVGQLGGAPAVQGQVLNATVNAQSLLQTPEQFKNIFLKNTASGAEVRLKDVARVELGSDNYQFDSKFNGKPAAGLAIKIATGANALDTAEAVEQRLSELRKNYPTGLADKLAYDTTPFIRLSIESVVHTLIEAVILVFIVMFLFLQNWRATIIPTLAVPVVVLGTFAVINIFGFSINTLTMFAMVLAIGLLVDDAIVVVENVERVMSEDHTDPVTATSRSMQQISGALVGITSVLTAVFVPMAFFGGTTGVIYRQFSITLVTAMVLSLIVALTFTPALCATILKQHDPNKEPSNNIFARFFRSFNNGFDRMSHSYQNGVSRMLKGKIFSGVLYAVVVALLVFLFQKLPSSFLPEEDQGVVMTLVQLPPNATLDRTGKVIDTMTNFFMNEKDTVESIFTVSGFSFTGVGQNAGIGFVKLKDWSKRTTPETQIGSLIQRGMALNMIIKDASYVMPLQLPAMPELGVTAGFNLQLKDSSGQGHEKLIAARNTILGLASQDKRLVGVRPNGQEDTPQYQINVDQAQAGAMGVSIAEINNTMRIAWGGSYINDFVDRGRVKKVYVQGDAGSRMMPEDLNKWYVRNNKGEMVPFSAFATGEWTYGSPRLERYNGVSSVNIQGTPAPGVSSGDAMKAMEEIIGKLPSMGLQGFDYEWTGLSLEERESGAQAPFLYALSLLIVFLCLAALYESWSIPFSVLLVVPLGVIGAIVLTYLGMIIKGDPNLSNNIYFQVAIIAVIGLSAKNAILIVEFAKELQEKGEDLLDATLHAAKMRLRPIIMTTLAFGFGVLPLALSTGAGAGSQHSVGFGVLGGVLSATFLGIFFIPVFYVWIRSIFKYKPKTINTQEHKS

ST25 4190 cg99_1212

MQKVWSISGRSIAVSALALALAACQSMRGPEPVVKTDIPQSYAYNSASGTSIAEQGYKQFFADPRLLEVIDLALANNRDLRTATLNIERAQQQYQITQNNQLPTIGASGSAIRQVSQSRDPNNPYSTYQVGLGVTAYELDFWGRVRSLKDAALDSYLATQSARDSTQISLISQVAQAWLNYSFATANLRLAEQTLKAQLDSYNLNKKRFDVGIDSEVPLRQAQISVETARNDVANYKTQIAQAQNLLNLLVGQPVPQNLLPTQPVKRIAQQNVFTAGLPSDLLNNRPDVKAAEYNLSAAGANIGAAKARLFPTISLTGSAGYASTDLSDLFKSGGFVWSVGPSLDLPIFDWGTRRANVKISETDQKIALSDYEKSVQSAFREVNDALATRANIGERLTAQQRLVEATNRNYTLSNARFRAGIDSYLTVLDAQRSSYAAEQGLLLLQQANLNNQIELYKTLGGGLKANTSDTVVHQPSSAELKKQ

ST25 4190 cg99_1213

MLLSNLESVPGHQILKQLDVVYGSTVRSKHVGRDLMASLKNIVGGELTGYTELLEESRQEAMQRMIVKAQQLGANAIVGIRFSTSNIAQGASELFVYGTAVVVQPQAPHLPDPFNA

ST25 4190 cg99_1214

MDNLIFQLVIFLILFSIGWAFGRHIERKHLNELLEKEQQFAHIRIDTNRFATSDQLGHFISSNVVISHDYFKYVLASIKNILGGRLTSYESIVERARREAIVRLKQQAQSVGANHIMGVRLSTTELGMQGGMVEVFAYGTAVKD

ST25 4190 cg99_1215

VVSCHEYKGSRQQALSHLRLQHNLSNPLIRIEQDQAWLECNYQIYRFSENDYFHSFGRYYFTLAKQQGIWKITGICQHLTKNIGNPRIHFSAMGQC

ST25 4190 cg100_1217

MNIQTIVIIGAGQAGASAILELRNNNYDGKIILIGDEPHLPYERPPLSKDMILRPEQTKIEILSQQKLEDLGVEFIKGNAVVKIVPEQYQVILESGEHVHYDKLLLATGGAPRRLPIFDILGKNVYTLRNLEDSQALFSVLQPEKRIVLIGGGVIGLELASSARFKGCDVVVLEQSPMIMGRSPAIISEFLLDQHRSRGVDVRLNVKIVETKLRGEEIVMLLDSGEELIADAVIYGIGIVPNAQLAIDAGLDVDFAIKVNENCQTSNPDIYAAGDVATQLRSDGNYARIETWENANNQAGTFARHVMGLAQPQANPSWFWTDQYDFNFQFVGDMSAKEWHIRGNMNPQEDQNVGFTLFGLDQGIVVAGIAVNQARDVRHLKKLVSSSSAFDKEKLLDISIELRKLI

ST25 4190 cg100_1218

MNRAGNKHSIITIAICFLIAVIEGIDIQAAGIAAVGIRESFGLNSSQLGIFFSAGILGLLPGALMGGRYADRIGRKMVLIGAVAIFAVFTLFTVWVNTFYTLLLVRFLAGAGLGAAMPNLIALVSEAVSNENRGRAVGLMYCGMPIGAILVSLLAAYDVSGHWQTIFYVGGVVPLLVLPLMIKFLPESREFLQTRFQDKSITHIQSSFKNLFQNNYASQTLLLWASYFFTLMVVYIMLSWLPSLFMELGFSRQQGSLAQVFFQMGAAIGTVVLGILIDRWNKAYVIILMYIGILAGLFSLNGAGNLTAMYAAAALMGTFTIGGQGVLYAFGSIVYPTEIRATGVGSAAAVGRIGAMLGPAIAGQLLAMGSGATGVISAAIPGIVISALLMLLVIRRLHQAK

ST25 4190 cg100_1219

MHPIANEKLWNRSFILCVLNNLFLFIYYYALLTILPLYILKELSGSISQAGLALTLFLISSIAIRPFAGLIIERLGEKTALRGSGLLFAFLAFGYLFINQLWVLLLIRFVHGIWFSILTTVTVPIVNEFIPEQRKGEGMGYFVMSTNFAVVFGPMIALATLRYTSFYNLFILLTLIVCLGLVFCFLIPVSSLKDRQWVQAKQKISLNGIIERRVVPIGVVALFVAMAYSSIMSFISVYSETKHLLAETSIFFIIFAISMIIARPWVGYSYDKRGANSVVYPSFILFAVGLFLVSQMQTQWEYWFAAVLIGAGYGSLFPVFQTMAIQTVEKNRIGYAVSTFFTLFDLGLAIGSVLVGLIIAQIGYEKTYQLCAWVTIMTLFLYRWLCNRDIKTKIGI

ST25 4190 cg100_1220

MELRHLRYFVAVAEELNFTKAALRMHTVQPSLSQQIKDLEDEVGVQLFIRSKRKVELTKAGEVFFKEALIILEHTEKAISLARQTAKVEQEQLNIGFVPAAEMKVFPYIMPNIRAHFPELTIDFQSLTDDAQLQALKKGEIDIAFTRYGANTNELVSIQIFQEPLTLIIPKNSPDAQQERISIKSFEHHNFVMCNEEASPELYRIIDGFFKKSKLNINVVQYSTNILLNVNLVGMEIGWSLVPQYVIPLLGDKIVVKKTVEPLPMIGLYVSYRKDHVNKAIDLILDVLKKQFHLNFSF

ST25 4190 cg100_1221

VNQTQHHTTEVAILGAGPVGLTIANYLSKQGVQVTVIEQLDQLIDYPRAIGIDDESLRTIQSVGLIKQVLPHTTPNHAMRFLTPKGRCFADIQPMTREFGWPRRNAFIQPQVDRVLLEGLAAYPTTEVRFSRQLNSFTQDKDHVTLNLTTKDQKQEIVQAQYLIACDGGNSFVRRTLNINFEGETAPNQWIVIDLANDPLATPHVYLCCDPVRPYVSAALPHGIRRFEFMVMPGETQEELSKPENINRLLRKVLPHTDNIEVIRQRVYTHNARIADRFRVDRILLAGDAAHIMPVWQGQGYNSGMRDAFNLAWKMALVIKKVAGPEILDSYQLERRDHAQAMINLSVLAGHVLAPPKKWQGVVRDGIAYVLNYIQPIKRYLLEMRFKPMPKYHQGILAPFPEKNTPVGKMFIQPHVQLENKDKILLDEVIGNNFAILAWGVNPTWGISPNVMKKWDKLGIKFIQVVPAVQLDAPNRSSHEHVITIGDVDTEIRSWFSQTNQSMVILRPDRFIAGLAIPQTVNSISEELFQKMHLVI

ST25 4190 cg100_1222

MPKFENEGEYLLWLASFIERITDGGKPKFPPISQLIPHGFKISKESFPVTVAPPKEEHADMIIKYFKSEDFMKTKKTPS

ST25 4190 cg100_1223

MEISLRFATPADLKAINERYAKIDFVPSHDDELIVLASISEKTVGQGRVVGIDANSGELGGIYVFPGNEGLGIARKVVDFLIKNSDFSMLYCLPFAELEGFYGSMGFAAVKDMAQVPEAVLKKHEWCNSNYDKPVLLLERSK

ST25 4190 cg100_1224

MYVTRALFFAVGVMALDVSPIVISNTHAATEQKMDNLSTTLSLIFADKPIEASLFSPQFLGQVSITQIQKIVDDLKVSLGALKSINVSNGSGTIDFEKGELPVSISLNEQGQISTLWFSAPHFKTISLDEMVKGLHENTIGKTSLLVIVDNKPVVVENDKTPMAVGSTFKLLVLKAYEDAIKKGELKRETIVSLKEKNRSLPTGVLQNLPASTPVNLELLAQLMIQISDNTATDSLIEVLKKPRIEALSPRNSPLLTTRELFQLIDPSNEKLRNKFKTGTKSTRLEVLAELDKLPLPNVSSIGKSATWQDAEWYMSANEICPLLESVQDAPALNSSLNPLFKNLNWQKIGFKGGSEYGVINFSVIGKTQKGHKVCAVFTANGNEPQPESKLALLFTGILQAVDSMNY

ST25 4190 cg100_1225

MCQAQSLRLCCAFFSPKKGTGFFISTSNKKFYNGKTMPNLVLPTRALYVVNKAIDLFHHRGFHLIGIDRIVKESEITKATFYNYFHSKERLIEICLMVQKEKLQEQVVAMVEYDLNTAAIDKLKKLYYLHTDLEGPYYLLFKAIFEIKNSYPNAYQTAMRYRTWLKNEIYSQLRMLNADASFNDAKLFVYMVEGTIIQQLGNNQVDEKDKLLDYFISKFS

ST25 4190 cg100_1226

MGNKIKSNKLFTRKSEAKNIIPLLSLGGAIFLSNSAFAASPSETTDAEKKPEALPTITITASRADELSTSAKQVTKLDEKQIELLRNGSSGNIATVLAKAVPGLSDSSRTITDYGQTLRGRNALILVDGVPMNLTRDTSRGLSAIDPESIANIEVIRGSNAIYGGGASGGIISITTKAAGGEPTAKTVVGLQTPLTNFRSNALSGDIHQYFTGSFNAFDYALDFGYQRIGSPYDASGDRVAPEPSQGDLYDADAYSVGGKLGYHIDDNQYVQFAANYYNAEQDSDYASDPSVKKAPAGTVPAKAIKGLKLKDQTKNENQIYNLTYNHKDFFGNKVEAQIYYRDFFTRFSPFDARVSENEVSPRGGQVDQVSQENNVLGSRLTVTTPLEFLGDTSLVWGGDFNREKSEMPLDIFDSDIYDQSGGLEFVKTGKLIYLPELTTQSVGGFVQLKHRFNDQWSAEAGTRYEDSYAQIDSFIPLSQLDPDTQSYKPNPYTVEGGKVKADAWLYNANVTFSPNDQHSIYASFNQGFQLPDVGVVIRNASTGFNLGSSFLEPVKVDNYELGWKGNFNNFSSSLAIFHSTSDLGAVQPVSNSLVMLRTKEKITGVEATFDYLDDANVWGTGGSVTWMKGREKPQDGAERDMTGFRIPPLKLTAYVSYSPTETWTNRLQATYFGSEDYRLNGVNSFGRYDVKTYTTADLISSFALNKKDTVTIGLENMFNRKYYPLYSQLLRSGNNTSHLMANGATLKVSYSHKW

ST25 4190 cg100_1227

LILFAALANFIAKRIIVRGIRHLVTKIKSLNQSVFAQHSVIKRFANIVPAIVIMNGITTVPHLSEKAVALVQMGAQAFIFLTIALTISELLNIFNLIYQRNPKSRNKPIKGYLQLVKLIIFVVCGLMILGTFLKKDVFTLLAGFGAMAAVLMLVFQNTILSLVASVQIASYDMVRIGDWIEMPSLNADGDVIDISLHTVTVQNFDKTYTTIPTNKLVTDTFKNWRGMSNAGCRRMKRSLYIDQTSVHFMTEEEQQKLKDFLLLDQYLNAKQSEIDEFNKHLGSQSRYNKRRLTNIGTFRAYVEFYLRQHKGIAQNQTLIVRQLQPTSEGLPLEIYAFTNTTAWVSYEAIQSDIFDHLIAILPEFGLRVYQAPSGHDFQKLTVE

ST25 4190 cg100_1228

MVGAYPPRLAFLSCLALFSIAVAIQTLQLT

ST25 4190 cg101_1230

MMGWLEGEVALITGGGSGLGLALVERFLQEGAHVGVLQRSQSKVDELIARYGDKIVAVTGDVANFQDNVRAVQATVERFGKLDCFIGNAGIWDHYADIVNMSGEQLDKAFDEIMSINTKGYLLGAKAALDELLKTEGSMIFTLSNSAFYSSGGGPIYTASKHADVGLVRELAYELAPKVRVNAVAPSGMNVNVKGAASLGQENLGLLDARDPQRIGQGMPLRFLPEPEDMTGSYVLLASRQNNRPLSGVFINADCGLGIRGLRQANAGFFDA

ST25 4190 cg101_1231

MNKIFVCQIDELDEGEALKVDCNVGDIAALAVFNFNGEFFAMNDKCSHGNASMSEGYLEDDGTVECPLHASRFCLKTGVPQCVPATDSIQTFPVVIEDGALYVEMVGA

ST25 4190 cg101_1232

MNQIIDSLKPQPVWRSIGLELHHDISQFLYAEAQLLDDWKFRDWLEILSEDILYTLKTTTNAQTRDRRKCIAPPSTWIYHDDKFVLERRVAKLETGMSWSEEPPSRTRHLITNIRVEATAQANEYQVFSNYLLYRSQKEKDILTYIGKRVDLIRKDESSSLGWKIAKRDITLDQATLISHNITVFF

ST25 4190 cg101_1233

MNGKIEVCEIDALVDAQNGRISPAIYTDPQLYELELERVFGRCWLFLCHESQIPKSGDFFNTYMGEDPIIVIRQKDGSIKALLNQCRHRSMRVSYADCGNTRAFTCPYHGWSYGIDGSLKDIPLEGVAFPHGACKEEWGLQEVPRVASYKGLVFGCWADDTPELLDYMGDIAWYLDAMIDRREGGSEVIGGIHKWEINCNWKFAAEQFASDQYHAPYSHASAVQVLGGKPNDDASKALGTAQTARPVWETAESCVQYGQRGHGSGFFLTERPDANFWVDGQVADYFRETYEETKERLGDTRALRLAGHNTMFPTLSWLNGMTTMRIWHPRGPDKTEVWAFCITDKAAPQHIKEAMELNACRAFGPAGFAEADDGENWIEIQKVLRGYKARKSKLIMEMGLGNEKYREDGIPGITNHVFSETAARGMYRHWANLLMHEKWEDVQRADEAYEQELLKKAAKKGEKELVK

ST25 4190 cg101_1234

MELRHLRYFVMVAEELNFSKAALRLYTAQPSLSQQIKDLEDEVGVTLLHRTKRKVELTEEGSIFLEQARLTLAQADKAIAMARQVSKLKKQQLTIGFVPVVEMKVFPSVLPSLRVQNPNLKIELRSMNNTEQMEALKNGIIDIAFMRDNLNNDEIQSQFVLREPLMLLIPHNHSLAQFKRIPVKLLDNIDFIIPSPEQSSILHHTILNFAEIHNIKFNIVQTAHNILFNINSIGMGLGCGILPSYIEPLTLNNIVTRPLDVDLPFLDLYVSYRKDAESSGVKEFIDLLSKVFYLNINKA

ST25 4190 cg101_1235

MGTVQIPEYKTDPFFGLEDKWIETAAGELTHYHELGEGTPILFLHGSGTGVSAAANWWLNLPQIAEQARCIAIDSIGYGQTVVAEGTAYGIRAWVDHAVRVLDALGIEKTWLVGNSLGGWLAFQFILDYPERVLGIVSMGTGGAKQTAALKAHANPELTEAGIRKTLEMFVVDKSLVTDELVKVRFDSAKNDYASDRLMDVVGARDRDRFEFPLDFEKMKEIQVPVLLIHGVQDVVIPVSRTWDILNIVPHADAHIFSQCGHWSQVEKAQEFNQIIKQYLAVHDVK

ST25 4190 cg101_1236

MRRQTLWIAMACLVATIGTTCTYAGFVEDSQVQLKFKNFYLDRQYDDTPAKNWGSWSQAVTLDAKSGYAEFGALQLGVDVLAQYAVRLDGRDRNADWVLPYSGISDTGTGKQERQFGKIGATLKAKISQTELRIGELLPVTPVIHFDPSRQLLTTYNGVWLESKDFKNTKLTLGYLDSINARYENQPMDFGLWPKALNNDGKTDGMYVAGIDYQLNKNWTASYFYGDVTNIYRQNYLGVNYKNNYDKLKVDSHIRFFDNAESGDALYGDIDNQALSIGSTLTYGSHSIGLSYQQMFGDHGSNPSAATGAPYFPSLAGWVPQPYLDNWSVASFIRKDEKSVGVAYSYDFSDLGIKGLKTTAKYWHGWGIDSAYESSALTGKGKEDEFNFILNYVVPEGRLQGLGFQWMYIDVNFDNIAGQASDLQEHRIATTYTYKF

ST25 4190 cg101_1237

MTLRDGMHPQRHQTTVEQMIAISTALDEAGVPLIEVTHGDGLGGSSVNYGFAAATDEEYLSAVVPRMKNAKVSALLLPGIGTVDHLKMAHEIGVSTIRVATHCTEADVSEQHITAARKLGMDTVGFLMLAHMASPKHLLEEAKKMVSYGANCIYVTDSAGYMLPQDVTDRIGLLRQELDSSIELGFHGHHNLSMGVANTVAAVEAGAIRVDLAAAGLGAGAGNTPLELFIAVANRMQIETGVDLFKVQDIAEDLVIPMMLNPIRADRDAATLGYAGVYSSFLLFAKRAEAKYGVSAREILLELGRRGTVGGQEDMIEDLALTMSKAKVISA

ST25 4190 cg101_1238

MVGIDPASEGLARAEKIGLKTTAEGVDGLLPHVLEDDIKIAFDATSAYVHAENSRKLNELGVLMIDLTPAAIGPFCVPPVNLEALLDAGEVPNVNMVTCGGQATIPMVAAISRVQTVNYAEIIATVSTKSVGPGTRKNIDEFTRTTAGAIEKVGGAKQGKAIIIINPAEPPLMMRDTVHCLVDGEPDQVAITESVNAMIKEVQKYVPGYKLVNGPVFDGNRVSIFLEVEGLGDYLPKYAGNLDIMTAAAARTAEMFAERVLSNPKAEVV

ST25 4190 cg101_1239

MSNSAVVDSVALALRQAELSQTAIIPIRAELGGEQANVDIAYAVQEINTVRALSEGRRLVGRKIGLTSKVVQAQLGVDQPDFGMLFADMAYGDGEEIPISKLIQPKVEAEIALVLKKDLTQTRHTYADIISATDYALPAIEIVDSRIENWKISLIDTVADNASSAAFVLGSRPVQLNQLDLVNCKMTMTRSEEVVSQGVGKACLANPLNAAIWLADEMVRRGRPLQAGDIVLTGALGPMVVAHPGDEFKVEIEGFNSVVACFAAE

ST25 4190 cg102_1240

VVVLSLSFIAYTLNQQVKPNVRQVVEDTLAENANIIAQLIAEDVAQKQVSTPEFDQKIQAALSRQLNATIWQHKKDEINQQLYITDQHGIVIYDSEDQAIGQDYSKWNDVYLTLQGKYGARSTATTPYDRNSSVMYIAAPIIYKHQLIGVVSIGKPNQSVQPYITRAERQLIYQALWIAALSLLLASMVAYWLRHSIDKVRRYAQALAPVNQAPFFYSAKELNQVTQALSEMREKLEDRAYVENYVNTLTHELKSPLTAIQASAELLQDDLPLEDQQQFAAHIVEQSHRLQSLIERMLLLTRLEKNQHVLEFQNIDLSKVIRDCLIQQHSALQIKHLQLEVNLPEHYIIYADPFWLKQAINNLLDNARDFCSEQGLLTIQVRKNTVTDGIEILLFNEGEAIPEYALNRVFETYFSLPRPHNQQRSTGIGLSIVQQIIQQHQGHITIQNISENYLDFLKQHKSGVLLKILLHQNFT

ST25 4190 cg102_1241

LIFYIGLFFISSLVDERQSYQQQVIQDIAKEQIRPQQVIAPYLKIPYQVQTICTDEQKKTYACIQTLFVTLGAESTDWKAQFKVSDNTYKRNMYKAISYQNHMLGKGVFKGKMLESQRNYLWDQAEIIFPIQDARGLNTKPIFNIDGKNYKFDFSAQSQGQHGFDLLHITSKQYPELILKLQKGFSFSLQFDLEGLSEFAFVPTSYEMTYQAKGNWGDVKYDGQSLPFKKLSKKQFFEADWKNIALGKRNLDRLSTCANSQCFYQAVNTQSNLISNVDAAYAASNTSSNNISGISTQFLEPVNIYTRTDRAIKYGIMVIIITFGCFFLFEVLKSLKIHPIQYALVAMAQGVFFVLLLSISEYYAFGLAYLVAAVACIGLITWYLYFVVQGFKAAILFGVLLSALYGMMYLLLQSSGKTFLFGSILSFILIACVMYITRHVNWYQSEQ

ST25 4190 cg102_1242

MYQTTKSALSQLKQLCPNQSSVAACLNQLRRAKIQFLNLGNIIVCPQYRSILIFKQRKLMEIETFSA

ST25 4190 cg102_1243

MSVQRLHVTSRYCEVAISGNLVHLAGQLADDTSVDITAQTQQTLDNIDRLLAEAGTDKSHILSVMIFLKDIDKDYAAMNAVWDAWISKEHPPARTCVESKLYAPDVLVEMTVVAVLP

ST25 4190 cg102_1244

MAAKSLSRKHQQDSALKRRAVLSSHEQMTLTRLQTVLPKFTVLAHVSFDALLTTKYAHTRRKYQNMTADFVVLDQNYQVVVVVMLEESGLRRAHQQYERRLLQLAGYRVLSYSTVPDYHELRRDLLTPESEMATLHATPQLERISNAFVKS

ST25 4190 cg102_1245

MSTAYTMQTAPKALFDYDKYWASCFEPAPFLPMSREEMDQLGWDACDFILVCGDAYIDHPSFVSGVIGRVLEAQGFRVGIIAQPDWTNVESFRVLGKPTIAWGVTAGNMDSMINRYTADRKIRSDDAYSPNNEPNKRPDRAATVYCQRCREAFPDVPVLLGGIEGSLRRIAHYDYWSDKVRRSILMDSKADMLMYGNGERAIIDVMHRLAKGEKIHEITDVRGTAFIINKHNKAAKAKFVEIASNDVDTIGRVDPIINPYVMTEDIDGCEIEKDKGNTLAQYQGFQKEIVANPIVREGDQLDENTQIVQLKPAQSKAIKHKLPPRELAVIRLPSFEEVANDQVLYAHANRILHLETNPGNARALVQRHGERDVWINPPPIPLTTEEMDYVFDLPYARLPHPAYGDARFPAFDMIKFSVNIMRGCFGGCTFCSITEHEGRIIQNRSEDSILREIEKIRDTAPNFTGIISDLGGPTANMYRLHCKDPEIEKNCRKPSCVYPGVCQNLHTDHAPLVQLYRKARAIKGVKKILIGSGLRYDLAVLNPEYVKELVQHHVGGYLKIAPEHTEQGPLSKMMKPGIGTYDRFKQMFDRFSKEAGKEQYLIPYFIAAHPGTSDYDMMHLAIWLKKNGFRADQVQTFYPSPMATATTMYYSGKNPLAKVARYTENVDIVKGEKRRRLHKAFLRYHDPNNWPLLREALKEMGRADLIGNSKQHLIPTYQPKGAEGEYKSARKKNSTVAGDSVKRSGQTVQNKNQHKRPQKGQVLTQHTGLPPRETGEKRPFGGKSKPKSKARG

ST25 4190 cg102_1246

MSKKTLIALLCCASLGLTACNDDNDQDQTSPTENVTEPTLISFAKLPVETYAAGPDSGAYVKGANGIYPPFKGQPVQGFSAALKNEDGSYMAMADNGFGTQDNSSDFLLRIYKIKPDFKNKTKGTGQVTVQSFIQLRDPNKLISFNIINGNTPERLLTGADFDPESMQRTSDGTYWIGDEFGPYLLHFSADGVLLDAPIALPNPLNPAQELRSPQNQFNKAQINFVEPLVQQSGGFEGMAISPDGQYLYPLIEKPIKSIRETERQLLISQFDLKKKAYTGKYYWFQLDSKATNIGDFQLFNDKEGIIIERDATQNNLGGYKKLIRIKLNESGQLVSREDLVDLIKIANPNMLYGTARAGDIGTGQVFAFPFETIEDVIIENGTTLTVFNDNNFPGSTGRNAKLADDNEIIQIRLPKALF

ST25 4190 cg102_1247

MDIGEVAKKAKVTTATLRFYEEKGLIKSIGRQGLRRQYGKQVIDQLALIALGRAAGFSLNEIATMFRQDGKPDLDRQKLKDKAEQLEQMAKRLHFISQGLEHAAVCPAENHMECPTFQKFLKAAIAGEYQPTKL

ST25 4190 cg102_1248

MNTSTLFFQALCLGIGATIVMDIWLLILKIFKIPTLNFAFLGRWVGWIFQGKLVHHSIAKSPPMKGEYLLGWIAHYSVGIIFALNFLLIVGSVWLDHPQFYSALLFGLVTVLIPFFIMQPAMGSGFASSKTPHPFLNCLKSLLNHSVFGCGLYLTAKLFQI

ST25 4190 cg102_1249

MKTVAIIYHSRQGHTQFIAQQIQIGVLSHHNIKADLLNAEDLIERPEILIQYDGLIWGSPTYLGGVSSKLKQLMDATGPLWKKQSFKGKLAAGFTVSSLPAGDKQSTLISIFTFCMQHGMLWVGNPILPEQHQGVAYTQAANRLGSWSGLMAQAEHASNADGFDEGDIKTAQQFGENFALTLNAYQGT

ST25 4190 cg102_1250

LKFDYWFCILLTSSLAQDHFAPSQLQRLSLKLFIIALMTIMTGCTSLGPNSGSFSWRSSKLSSGLQKAYSVPPSTANRLSPMIIQSADRYNVPPLLLAAVIRQESSYNSNARSPTGAIGLTQIIPSYWQQTCAGNLYDESTNIQCGAYILNHYYQTADSWFKATAYYNVGPTGYERSFWTRHKAKKYARSVKRHQKTLKSAL

ST25 4190 cg103_1251

MIKEIVANIKSDDLEILFRMDSGYFDEKIIETIESLGCKYLIKAKSYSTLTSQATNSSIVFVKGEEGRETTELYTKLVKWEKDRRFVVSRVLKPEKERAQLSLLEGSEYDYFFFVTNTTLLSEKVVIYYEKRGNAENYIKEAKYDMAVGHLLLKSFWANEAVFQMMMLSYNLFLLFKFDSLDSSEYRQQIKTFRLKYVFLAAKIIKTARYVIMKLSENYPYKGVYEKCLV

ST25 4190 cg103_1252

MSHQLTFADSEFSTKRRQTRKEIFLSRMEQILPWQNMTAVIEPFYPKAGNGRRPYPLETMLRIHCMQHWYNLSDGAMEDALYEIASMRLFARLSLDSALPDRTTIMNFRHLLEQHQLARQLFKTINRWLAEAGVMMTQGTLVDATIIEAPSSTKNKEQQRDPEMHQTKKGNQWHFGMKAHIGVDAKSGLTHSLVTTAANEHDLNQLGNLLHGEEQFVSADAGYQGAPQREELAEVDVDWLIAERPGKVKTLKQHPRKNKTAINIEYMKASIRAKVEHPFRIIKRQFGFVKARYRGLLKNDNQLAMLFTLANLFRVDQMIRQWERSQ

ST25 4190 cg103_1253

MVKKSLRQFTLMATATVTLLLGSVPLYAQTADVQQKLAELERQSGGRLGVALINTADNSQILYRADERFAMCSTSKVMAVAAVLKKSESEPNLLNQRVEIKKSDLVNYNPIAEKHVDGTMSLAELSAAALQYSDNVAMNKLISHVGGPASVTAFARQLGDETFRLDRTEPTLNTAIPGDPRDTTSPRAMAQTLRNLTLGKALGDSQRAQLVTWMKGNTTGAASIQAGLPASWVVGDKTGSGGYGTTNDIAVIWPKDRAPLILVTYFTQPQPKAESRRDVLASAAKIVTNGL

ST25 4190 cg103_1254

MAAGTWHAVLSLDTGGIIFEVKHGGYQPVAADDYAHWAPAEGEPGTTELMAWYAQAQVGDSAFAV

ST25 4190 cg103_1255

MFMSVFHNWLLEIACENYFVYIKRLSANDTGATGGHQVGLYIPSGIVEKLFPSINHTRELNPSVFLTAHVSSHDCPDSEARAIYYNSRHFGKTRNEKRITRWGRGSPLQNPENTGALTLLAFKLDEQGGDCKEVNIWVCASTDEEDVIETAIGEVIPGALISGPAGQILGGLSLQQAPVNHKYILPEDWHLRFPSGSEIIPRNLRAPAPTPSHLRTTVAIKGSFRGAEF

ST25 4190 cg103_1256

MIIGYARKSTHLQDVTHQVDELTKAGCEQIYHEQISRGGTKRAKNGAPELENCLKALREGDTLVVWALDRLGGSLSQVITLLDDLKKRGITFIAIKDRIDTSAPVIGEIYTHLMAIFSNFERNRNIERTRSGLAAARARGRVGGRKPSLSEDDIKQMKILLADPEMTVGAVAKRFNVSRMTIYRYTTKGNHSEKINDE

ST25 4190 cg104_1257

MAKKPIYKSLYFQVIIAIIAGVLVGHFSPSTTQIVNGVEQHIPGLGEQLKPLGDAFIRLIKMIIAPVIFCTVVSGIAGMESMKSVGKTGGVALLYFEIVSTIALLIGLVVINIAKPGVGMNVDPATLDTSGIQKYVTSGESQSTIDFLMHIIPDTVVGAFANGEILQVLLFAILFGFALHKLGDAGRPVLKLIDQIAHVFFNIVNMIMKLAPIGAFGAMAFTIGKYGVGSLVQLGQLIICFYITCLLFIFLILGTISRISGFSILKMIRLIREELLIVLGTSSSESVLPRMLRKLEIAGCEKSVVGLVIPTGYSFNLDGTSIYLTMAAIFIAQATNTQLDIQHQITLLLVLLISSKGAAGVTGSGFIVMAATLSAVGHIPVAGLALILGIDRFMSEARALTNLVGNSLATIVVAKWVGALDKDKLNDALNNPDEVDRKMLAPKNPQVAEGLD

ST25 4190 cg104_1259

MNTKRNIYKDAEHPFAQYVRILGKGKTGSRSLSYEEAYQAFTMILKGEVLDVQLGAFLMLLRVKEESIDELAGFVQATKDQLHFAPLEVDLDWSSYAGKRKHYPWFLLAALTLAHHGYKIVMHGASGHTINRVYTEQVLEYLDYKICHSEQEVRTQLAEQNFAYLPLDVISPVLSELISLRNVMGLRSPIHTLARLINPFNAKATLQAIFHPAYRTSHQHSALRLGYQNSAVIKGEGGEFERNPDAKTLICGIKNGELYEHELPKLTPERSPIEEELDLATFKAVWLGQQHHEYGEMAVTETMGIALYTMGVVSNFEEAMQKAKVLWETRNLSNLNL

ST25 4190 cg104_1260

MNIEQMLSVLNPEIVERLKTAVEIGKWPNGVALTKEQRETCMQAVIAWELKNLPEEQRSGYIDRGTKEEGEVCEDDHHKHEPEFKPIRFV

ST25 4190 cg104_1261

VQISHKSFRNVGLIGRPDKSSVVETLCLIHDHLLSLGLNPIFDQETAELVPYDHAQVVSRHLLGEVADLVIVVGGDGSLLHAARALVRYNTPVIGINRGRLGFLTDIKPSEAIFKLDQVLQGHFQLDRRFLLEMEVRTNGEVIYDAIALNDIVLHSGKSVHMIDFELNIDGQYVYRQHSDGLIVSTPTGSTAYALSGGGPILHPSMDAIALVPMHPHTLSSRPIVVGGQSEIKIVIRENRVLPMVSADGQHSVSLNVGDSLHIRKHPFKLSLLHPPGYDFYMACRTKLGWNQDFESFQRDES

ST25 4190 cg104_1262

MKFERGIGFFALIFSILVIGAFIALSIYLIRLDNIIREKFEGQRWDIPAKVFARPLEIYNNAPITQANFTQELKLLGYKTSSNYDKSGTYVAQGSNMYVHTRGFDYGDSVEPEQVLELSFANDQVVEVRSTKPSSTGVARLEPLLIGGIYPQHNEDRVLIKLNSVPKPLIEALISTEDRNFYHHHGISIRGTARALVSNVTGGRRQGGSTLTQQLVKNFYLTPERTLKRKVNEALMALLIELHYSKDEILEAYLNEVNLGQNGNYSINGYGLASQFYFGLPLRELNVAQQAYLVGLVQGPSLYNPWKNPEGAKNRRDTVLNNMRVMGYLTQAEYETEIARPLNVLSKPSLGPAKFPDFLDIVRRQLRTEYQESDLTNQGLRIFTTLDPIAQTQVQNAFKASVERLANSNPARLKNLQGAVLIAHPENGELIAAVGSTQDFTGFNRALDAKRQVGSLLKPVIYLSAIESGRYNWASQIEDAPISVPVDGGKSWTPKNYSGGGHGIVSLSEALANSYNLSAVRLGQEFGLSTFTNNLRKFGVESTIPAYPSIFLGAVNMSPMEVLGIYENFATGGFKYPTRAIRSVVDANGRLLDRYGLNVQQTIDPSVGYIMNYGLQQVMSSGTGRAAYNSLSPALKLAGKSGTTNDTRDSWFAGYSGNHVAVVWLGLDDNKVTGLTGSSGALPVWINVMKQLRQTPVNLRQPDSVQWQWIDRASGDLSAQACDGAMYIPMLAHTVPHRATPCGAPYYQVDPTYTPQSDNTIPEPQDDNTDSYIRESENQMEQDLSNNTRIISSGSYNN

ST25 4190 cg104_1263

MLKKGVFYIGAVLMVGCTTLPDHSAPKEKTPPVSEKKKSESSSGVKITPYDHPEIQRKSLQVIVPQQKKPQRFNDDGSQLPAFKTLMQKTEQAYKNQQWSEAERYALQAQRLAPQAAETYLFLALTANHKQQYSNAESLARRGLSFAQSQAMKKQLWLVILKAGQQRNNPKTVQEAQQALKAF

ST25 4190 cg104_1264

MPKPIVDVAIAILIHRGKILVGWRGEQQHQGGKHEFPGGKVEQGETPEEACRREIYEEVGIGLKDWHQFDYIHHEYDDIIVNLHLFHSYVPDELLNLIHQPWTWYTREQLLHLNFPKANKDIIKRLYWPHFIKISHTLTSVANSDALLYWRIEDEFGPREVEQLTALDEGQRSNLIINVDIWQQLNSELKKQIKTVHLKQSQLMSLHKGDLEIGIRFIAACHDAVSLQHAQQIGCDAVFVSPVKVTATHPDVSALGWDRFADLIEKCQIPVFALGGMSPDDLATAQQHGAYGLAGIRNF

ST25 4190 cg104_1265

MNAPFSLFTRNTDSAHALPMLHSNNLFALGREIRIMHAGEEYRLRLTRNNRLILTK

ST25 4190 cg104_1266

LQSQLVKTTMWSSIVVGLLALSLLTIFSIYHNMSVQDEIMDEISDTLLVSDLSKHSMKQFDELSDEFDIQYELLDTGQVLTHSHTYQHELFEKGNLSEGFSYFWFDGQLWRSLAAQQEDSELQVEVFQPISTRIEEVLKALAGYSGLMVLFWLLQWVIVSWRTEQQLAALNLLSKRIAQKTASNLEPIQEPDVITEIQPVIDALNQLLARLQRALVAEQRFTADASHELRSPLSAIQMRLQVLQRKYQHIPELDHDFERIQEDVSRSTKILENLLLLARLEPNETEQLELSKSVVDLNHVLARVIDTVNIDAQAKHMVIETNILSNETKTFANEELMFIAFRNLFDNAIRYSPALGAIHVELSQYQQKLKISIEDTGNGVDDEVLRRLGQRFFRVLGTKQQGSGLGLSITKKIIQLHGGELHFMHASQGGLKVEVILPFNREIQK

ST25 4190 cg104_1267

MRILLAEDDRSQAESIQSWLELDGYQVDWVERGDYALTAIEQHDYDCILLDRGLPQLTGEKVLTTIRHKQKNVPVIFITARDSIHDRVEGLDLGANDYLVKPFSLEELSARIRAQLRKQTLSQQSILTWGDLQLDTQAKVVLCAGKTIDLTAKEFQILRKLMVHPEHIITRDQLEEALYAWGEEIESNAIEVFIYQLRKKIGSSSIKTIRGLGYRMGDLK

ST25 4190 cg104_1268

MKMLKVILMTAGMATAGMVVANTPVNQAAIAPATVTTVKQALASKDNTPVKLHGQVVKSLGDEKYQFRDKSGSITIDVDDELWQGRAVSANTNVTLIGEVDIDYKPLKRVEIDVDQVQF

ST25 4190 cg104_1269

MNIHVEIDTHWCLEQLLQEGRITERDKLLVQTTNRQKDQLKWHPLQWIAHFNLKDQHHVQAHLSLNRLCQWFADRAQLPLFVIDPLKADVSALTQVMSQEFAIRNHILAVEVHADRIVIGTDQPFQTDWLNNLEKSLAPKKIERVFLNPEQLQRYLREYYQVSRAVNSAQKDAAHDRDNKGVEALLQLGDSQNPDANDQHIVKLVDWILQFAFEQSASDIHMEPRKDNGKVRFRIDGVLHTIYNMPSNTLTAVISRIKILGRLNVAEKRKPQDGRLKTRTPKGQETELRLSTLPTAFGEKLVMRIFDPDVLVRSFQQLGFDQSLLQQWQRITQNSHGIILVTGPTGSGKTTTLYSSLKQLATEQVNVCTIEDPIEMLEPSFNQMQVNHAIELGFADGVRALMRQDPDIIMIGEIRDQDTANMAIQAALTGHLVLSTLHTNDAPSSLTRLHDLGVQPFLTAATILGVLAQRLVRKLCPNCKQQTHINEEEWQHLTFDYIMEMPEKVYRAVGCEECRHTGYKGRIGIYEFMPVSLELKHLISSHATLNELRSQTKKESVEPLRIAGARKVVEGLTTLEEVLRVVPLN

ST25 4190 cg104_1270

MNLPMTMSLQTVSFEEFITTIAAALGCGLLVGLERERSKLKHEYKTFAGFRSFAISSLLGAICFLFGTAIGIVGALLIGAISIVSLKNQPNDPGVTTELAFIMTYFIGALCIWNISLAAGLAVIMTIILLAKQSMHGIASQWITESELRDGIFLLALLLIALPLVPNKPFWGPVLNPHVILKLLTLILFVQALAHIAKRLLSSKNALLLSSLASGFVSSTATIASLGLEVRSGRANAKTNAGAALMSCVSTLVQTLIIVVGISLAWFKLIIFPTLIALAFLAVWAFILLRKAEPSTTSSELDTRMFSLKEAIIIAGTLTLIQAGVYGLSLYLGNAGLIAGTLLASLFEIHAAIAAVIVQGEPNNSQTSLLIAFMGGFAVHAIAKSINSAISGGLHYALAFIPAQILHMTIFIGLLWMNIHWF

ST25 4190 cg104_1271

MCSPFREKFGIPRQPNLVNIESYIEMVEPYNDLLAFEGIEQFSHLWLIWQFHDNKNQETATKFRPQVRPPRLGGNEKIGVFATRSMYRPAPIGLSVVRLNKLEKFGKSLRVYVTGSDLLNGTPILDIKPYIHYSDAIVDAQSGYAQAEPERKSVLWSENALSTQQSLFKNKEINAQYLDELEQVLSLDPRPAYQEDPERVYKMKFSNFDIHFKVNGNVITVVDMVKDTSLV

ST25 4190 cg104_1272

MAAFNVERITHVHHWNDTLFSFKTTRDASLRFKNGQFVMIGLEVNGKPLMRAYSIASANYEEELEFFSIKVQDGPLTSILQKVQVGDEILVSKKPTGTLVHDDLLPGKNLYLLSSGTGLAPFLSIIRDPETYERFEKVIVVHGTRYISELAYQDLILNELPNNEFFEELGIKDKLVYYPTVTREPFHTQGRVTTAIETGALFEKIGLPRFNRETDRAMLCGSPAFLKDVAALLDQHGLVESPRMGEMGDYVIERAFVEK

ST25 4190 cg104_1273

MSVVAFKQVDVFTSQAFKGNPVAVIMDASTLTSEQMQAIANWTNLSETTFVLPATDSQADYQVRIFTPQSELPFAGHPTIGTAYALLEAGLIKAKEGQLVQQCAAGLVTLTVNDSNHISFELPKPKITLLDALQTEKLAEILKCKINTQWNAALVDVGARWVVLQAVNAKAVLASQPDLNALKQFSLEMKVTGSTIYGFYEESNEQKRIEVRSFAPAYGVNEDPVCGSGNGSVASFIRYHGILPAQNDVVLSSQGQALGREGQLQLELHQDKILVGGAAVTCIDGTIKL

ST25 4190 cg104_1274

MYKYKKVSDDLTLQIQNGRQPVGSRLPSIRDCAQHYQVSINTVKEAYRKLEDQGLISVRPQSGYYVCHTPSNLPDLEERYISEEKVSLSGISCFLAQIVKKQMDPDYVNLALACPSGENFYPIERLKRLTAQVLRTQPDIFKSYTLPPGSIRLRTQIARRSLQLGMFLSADDIVLTHGTMEALSLAIMATTQAGDSIALEIPTFHNLYPLLQNLGRKIVEVPTSPHTGMCLDALEELLKSQSVQAILTIPTGHNPLGFSMAENNRRRLAELANQFQIPVIEDAMYAELQLTEPLLPNIKAFDQNGWVLVCGSYTKTVAPDYRIGWLEAGRFRNIVQQLKFTTTVAEPALLTETLGLFLENGGYDLHLRQLKKRYQQQIDTIKSYICRYFPVGTRVSRPQAGFILWLELPESIDTLELFHQAMDEKILCMPGILCSADKRFSHCLRIAACFELNPKMLNALMRLGELAKNMLPVQKDVDQIPTNSVG

ST25 4190 cg104_1275

MKTELLSPAGSLKNMRYAFAYGADAVYAGQPRYSLRVRNNGFDHDNLKIGIDEAHALGKKFYVVVNIQPHNSKLKNFIRDLEPIVAMQPDALIMSDPGLIMMVREHFPEMQIHLSVQANAINWATVKFWKDYGLSRVILSRELSLEEIKEIQQHVPDIELEVFVHGALCMAYSGRCLLSGYMNKRDANQGACTNACRWDYKLHKAQEDLNGDVIPVTQLNTPEKSCCSSGQSEATSVQTLLVQRNEEEMFAAEEDEHGTYFMNSKDLRAVQHVDRLTKMGIASLKIEGRTKSYFYCARTAQIYRKAIDDALAGKPFDPSLMTQLEGLANRGYTEGFLRRHVHSEYQNYTDGSSHFNYQQFCGEVVERHGNYIRIEVKNRFVVGDSLELMTPQGNITFNLTEMRDLKGNPITDAKGSGHFVEIPLSQDVDISYALLIRNLPHAKESITASTLAYSAS

ST25 4190 cg104_1276

MCLPECPNTAIFEGNKVYEIDPLRCTECVGFYDAPTCKAVCPIDCIKQDPAHIENKEQLLEKFKGLNLIGSFSS

ST25 4190 cg104_1277

MSYLYLAIAIACEVIATSALKASQGFTVPIPSIITVVGYAVAFYLLSLTLKTIPIGIAYAIWSGAGIILISAIGWIFYKQHLDLAACIGLALMIAGIVIINVFSKNTHL

ST25 4190 cg104_1278

MEQVAKLVIRDLHKTFGDHEVLKGISLDANAGDVISIIGSSGSGKSTLLRCINFLEQPSNGTIKLNGEKLRTVFDKKGNLKVVEPKQLQKMRTQLMMVFQHFNLWSHMTVLENVIEGPIHVLGVKRSEAEERARKYLRKVGLPESVESKYPAFLSGGQQQRVAIARALAMEPEVMLFDEPTSALDPELVGEVLKVMQGLAEEGRTMIVVTHEMGFARHVSNQVIFLHQGKIEEQGHPDEVLNNPKSERLKQFLTGSLK

ST25 4190 cg104_1279

MIEILQQYWQSYLWTDGLQITGVAMTAWLLVLSIGIGFVLAVPLSLARVSENIWLHGPVWLFTYVFRGTPLYIQLLIIYSGIYSFDYIHEHQTLDAFFREGINCTILAFALNTGAYTTEIFAGAIRSIPHGEIEAAQAFGMSKWKLYTRIIIPSMLRRALPFYGNEVILMLHATTIAFTATVPDILKIARDVNAATYDTFSAFGIAAVLYAVLAFILIWIFRKLEKRWLAFLKPSNH

ST25 4190 cg104_1280

MTIQLALLSLLLSVIIGLIGASSKLSNIKALRYIATAYTTLIRSVPDLVIMLLLFYSLQLGLNQITEALQMDQIDINPFVAGVITLAFIYGAYFTETFRGAFQSVPRGQIEAAMAYGMTPWQVFHRVLFPQMMRFALPGIGNNWQVLIKATALVSIIGLTDIVKITQDAGRSTMQLFFFSIVAAAIYLAITTVSNLILIWLERHYSAGVRKGQL

ST25 4190 cg104_1281

MASFMKKIFVSTTLVLAAVATNVQAKDWKVIRFGTESSYAPFEYKTPDGKLTGFDVDLGNAICAKLKAKCVWVENSFDGMIPALKAKKFDGILSSMTVTDERAKQILFSSKIYNTPTRMVAKKGSPLLPTPTSLKGKRVGVQQGTIQETYAKTYWAPKGVSVVPYPTQDLIYQDMMSGRLDATLQDAIMVDGAFLKQPKGKNFSFAGGNVIDVKTLGVGAAIGLRKEDADLKANIDKALAAIIADGTYKKLEKKYFSFSIY

ST25 4190 cg104_1282

VDLFHAMRVFNKVVETNSFSLAADSLGLPRASVTTTIQALEKHLQVRLLNRTTRKISLTPDGAVYYDRTARILADVADIESSFHDAERGPRGQLRIDVPVSIGRLILIPRLRDFHARYPDIDLVIGLNDRPVDLVGEAVDCAIRVGELKDSSLIARRIGTFQCATAASPIYLEKYGEPTSIEDLQKNHKAIHFFSSRTGRNFDWDFVVDDLIKSVSVRGRVSVNDGDAYIDLALQGFGIIQGPRYMLTNHLESGLLKEVLPQWTPAPMPISAVYLQNRHLSLKVKVFVDWVAELFAGCPLLGGTALPFDQKCEFACDKETGHEYTIRTLVEQHNIAEAYTLKT

ST25 4190 cg104_1283

MSFSRKQFALSAIFVAILATGGSFMLLHENADAKAAPTAAQQAATVDVAPVVSKTITDWQEYSGRLEAIDQVDIRPQVSGKLIAVHFKDGSLVKKGDLLFTIDPRPFEAELNRAKAQLASAEAQVTYTASNLSRIQRLIQSNAVSRQELDLAENDARSANANLQAARAAVQSARLNLEYTRITAPVSGRISRAEVTVGNVVSAGNGAQVLTSLVSVSRLYASFDVDEQTYLKYISNQRNSAQVPVYMGLANETGFTREGTINSIDNNLNTTSGTIRVRATFDNPNGVLLPGLYARIRLGGGQPRPAILISPTAVGVDQDKRFVVVVDAKNQTAYREVKLGAQQDGLQIVNSGLQAGDRIVVNGLQRIRPGDPVTPHLVPMPNSQITANATPQPQPTDKTSTPAKG

ST25 4190 cg104_1284

MNISKFFIDRPIFAGVLSVLILLAGLLSVFQLPISEYPEVVPPSVVVRAQYPGANPKVIAETVASPLEESINGVEDMLYMQSQANSDGNLTITVNFKLGIDPDKAQQLVQNRVSQAMPRLPEDVQRLGVTTLKSSPTLTMVVHLTSPDNRYDMTYLRNYAVLNVKDRLARLQGVGEVGLFGSGDYAMRVWLDPQKVAQRNLTATEIVNAIREQNIQVAAGTIGASPSNSPLQLSVNAQGRLTTEQEFADIILKTAPDGAVTRLGDVARVELAASQYGLRSLLDNKQAVAIPISKLRVRMLYKFPIKCVAR

ST25 4190 cg104_1285

MKELSKDFPSSIKYDIVYDPTQFVRASIKAVVHTLLEAIALVVVVVILFLQTWRASIIPLLAVPVSIIGTFALMLAFGYSINALSLFGMVLAIGIVVDDAIVVVENVERNIEAGLNPREATYRAMREVSGPIIAIALTLVAVFVPLAFMTGLTGQFYKQFAMTIAISTVISAFNSLTLSPALAALLLKGHDAKPDALTRIMNRVFGRFFALFNRVFSRASDRYSQGVSRVISHKASAMGVYAALLGLTVGISYIVPGGFVPAQDKQYLISFAQLPNGASLDRTEAVIRKMSDTALKQPGVESAVAFPGLSINGFTNSSSAGIVFVTLKPFDERKAKDLSANAIAGALNQKYSAIQDAYIAVFPPPPVMGLGTMGGFKLQLEDRGALGYSALNDAAQNFMKAAQSAPELGPMFSSYQINVPQLNVDLDRVKAKQQGVAVTDVFNTMQIYLGSQYVNDFNRFGRVYQVRAQADAPFRANPEDILQLKTRNSAGQMVPLSSLVNVTQTYGPEMVVRYNGYTSADINGGPAPGYSSSQAEAAVERIAAQTLPRGIKFEWTDLTYQKILAGNAGLWVFPISVLLVFLVLAAQYESLTLPLAVILIVPMGILAALTGVWLTAGDNNIFTQIGLMVLVGLACKNAILIVEFARELEMQGATAFKAAVEASRLRLRPILMTSIAFIMGVVPLVTSTGAGSEMRHAMGVAVFFGMIGVTFFGLFLTPAFYVLIRTLNSKHKLHSAAVHEAPLASPHDH

ST25 4190 cg104_1286

VITSKQNWLLSSLMGSLLLAGCSLAPEYQPAKVIVPVKFKESDPKLEDNNWKIAQPADQQTRGEWWRIYNDAQLNELEQQAIAGNQNLKAVAANIQASRALRSAAQAERLPSIDAGFGPTRQKPSPASLGLDDKAHTSAQTLWRAQANVSYELDLFGRVASSVNAATADLQQQEALYQSALLALQADVAQGYFLIRQLDTEQAIYNRTIKLLGETRDLMQLRFKNGLVSELDVSRAQSELATAQTTALNIARNRASAEHALAVLLGKPPADFNLAVQPLTANSIRLPAGLPSTLLERRPDIAAAERAMAADNARIGIARAAFFPKLSLTGALGYESSSLSELGKWSSRTFLLGPVAGTILSLPLFDGGQRKAGVAQARAAYEESVANYRQTVLNAFREVENGLSDQRILDQQIQAQNQALSSSRHANQLSHLRYREGAISYLDVIDSDRTILQQEQLAAQLKGSQIIASINLIRALGGGWSS

ST25 4190 cg104_1287

MFANISISEFDPELAQAIASEDERQEAHIELIASENYCSPAVMEAQGSKLTNKYAEGYPGKRYYGGCEFVDVIEQMAIDRAKELFGADYANVQPHAGSQANSAVYLALLNPGDTVLGMSLAHGGHLTHGAKVSFSGKTYNAVQYGLNAETGEIDYEEVERLALEHKPRMIVAGFSAYSRVVDWQRFRDIADKVGAYLFVDMAHVAGLVAAGVYPNPVQIADVTTTTTHKTLRGPRSGLILAKANEEIEKKLQSAVFPGNQGGPLMHAIAAKAICFKEAMSDDFKAYQQQVVKNAQAMAEVFIARGYDVVSGGTDNHLFLLSLIKQDVTGKDADAWLGAAHITVNKNSVPNDPRSPFVTSGIRIGTPAVTTRGFGEAEVRELAGWIADVIDSKGDEKVIADVKAKVEAVCAKFPVYAK

ST25 4190 cg104_1288

MNFIIKQNEVSSVLNKNGSSFIKNEPLFINQLKSSFKAYILNLSIVNFSLQ

ST25 4190 cg104_1289

MKMKLLMTSVLSTSLFLVACGGGSSDDGPATTNPSGTPTNNIQNPVVKVEAYTSTNLGSVAAESSILTYKMLGQSGQEVQATSLVFTPNTPPPVGGWPIVVWAHGTTGVADVCAPSKAALADSTKDLISKLLAAGYVVVAPDYEGLGTPGIHPFLNVKSEAFSITDAVVATRNYLSQRNLLTSKKWVTVGHSQGGHAALGAAQYASRAQLEYKGTVAVAPASNLGFILIAGEQSVANATLDKKISMYAQLDTYTALVTAGIRNTQPTFDYPQVFTPQISSIAQQAETICSGPLGQAFGAGMTQYVTEHNGTLDGYTRTQPNFMAVPLVKTFLDKDSQPLQVKVTTPIIIYQGLADSTVPKVATDILISNATVVGTKINSYVTGNWDHGTAMSSNVDNIVGNVQSLLAAQ

ST25 4190 cg104_1290

MQAIILDTETHTLNGLPIEIAYAPIEINAGKLTLDKSKLFDQLYQVGTPISYAAMAVHHILESDLENQPHYKTFKLPDTTTYIIGHNIDYDIAAIARCGVDVSHIKPICTLALARKTWPDAEAHNISALIYMISQGSSKARELLKGAHRADADIILTANILMHIVYHLNIHDIEELYRVSEEARIPTTINFGKHKGTAIAELPKDYIQWLLRQDELDVYLRKALESAF

ST25 4190 cg104_1291

LSFIVSPIYILLRTVLFDRKIKQAASFLYWGKMMAGFYRTNLGRVALQQRNIALNAKQRRLLLLIDHEDFQTLNTEFKKRIAPPELIQQLIDLKLIAPISENDSEFAEQIPLSESPTTSLEVKAQQKSTIDENESADLTGEIKVSLEPSCHSSNIENTQPPIPVQQLTFEEIQLLMKQSLSQYCGLMAKPLIQKIEQIKNLQELKMCQMQWITSLQESRIPPHELAHTLHSINYSIQLIQQKN

ST25 4190 cg104_1293

MTDALVLRDLSKTYRNGFQALKGINLTVPEGEFYALLGPNGAGKSTTIGIISSLTKKTSGTVEIFGHNLDTHPSLAKQQLGVVPQEFNFGQFEKAFDILVTQAGYYGIHRKIAEKRAEHYLEKLGLWEKRNIQARMLSGGMKRRLMIARAMMHEPKLLILDEPTAGVDIELRRSMWDFLTEMNENGTSIILTTHYLEEAEMLCRQIAIIDRGVIKEDTSMKSFLNQLSEESFIFDLAEPIAPLQLNIIGVKFNLIDSSTLEVTMDKAHTLNDLFQLLESQGIRVRSMRNKSNRLEELFVKMVEKNLEGAAK

ST25 4190 cg104_1294

MNFNQLQIALWTLVRKEVRRFLRIWPQTLLPPAITMSLYFVIFGNLVGSRIGEMGGVSYMQFIVPGLIMMAVITNSYANVSSSFFSVKFQKSIEELIMSPVPLHIILWGYVIGGICRGVLVGAIVTAMSMFFTDLFIHNWFVTIYTVLITSVLFSLGGFINAVYAKSFDDISIIPTFVLTPLTYLGGVFYAISALGPFWQKLSLINPIVYMVNAFRFGILGHSDVNVSFSLIIVTLCCAVLYGIAYYLLARGSGMRE

ST25 4190 cg104_1295

MSVEQSLLGKETQYPTSYQPDVLFPIARAQSREKYAHIEGITQGKDWWHVFEISWLNAHGIPQVAIGRITLPASSPNLIESKSLKLYFNSLNFTQFDSTQSFIKTVEKDLSAAAGAKVELTLFQVDDLEISKPQGICIDDLMPERLERHPDATLLKLDESGEEIEVELYSHLLRSNCPVTGQPDWGTVFIRFKGKKPCYRSLLAYIISYRQHNGFHEQCVEQIFADIWQNLQPEKLMVYATYTRRGGLDINPCRVSDLTWMPKPIRLARQ

ST25 4190 cg104_1296

MPFFGFTPSEGLLNDIQTGIANKNSSEPLYPLRDKIALQLNEEIIENVLTQLVQHFPASEKRDTAEKLAGYVKSTVAVLLKQLLSKAPNDVVKQSVEFSEKSLFKDPQGQYKVGVALDAGLVTNLKHNFAELQAGNDINKAALAELYKQFGDAMVRHFMSDFNKTLDLGMIKRKAADIGAAAVTKAVHIAIDKLIPSLNRTELKAMAEYHDGLFFN

ST25 4190 cg104_1297

MQPSRWQQLHIDPWLCLFLFLNALLGLTVLYSASAQDVGLVSKQAMSFGIGFLVMISLAQIPPKVYQAFSPYFYLFGLFSLIGVMVFGEVRMGAQRWIDIPGFGSVQPSEFMKIGMPMMVAWFLARKPLPPSFSQVILSLMLIGVPFLLIAEQPDLGTSLLVLASGIFVLFLSGLSWRMIGAAAACAAIVIPIAWEFLLHDYQRQRVLTLLDPEADALGTGWNIIQSKTAIGSGGFSGKGFLEGTQSHLHFLPEGHTDFIIAAYSEEFGLIGVLILVILYSAIIFRTFQIGLQSFHNYGRLVAGAFGLSFFVYVFVNAGMVSGILPVVGVPLPFMSYGGTAIITLMATFGLVMSIHTHR

ST25 4190 cg104_1298

MLSQFVNKTLKVLALCTGLISTSHFAQANDFVTHPNYLNFKQKAMSTYGLSGEQVDAAMNGAKNLPNIINIMTRPGESKPWYDYRSMFLVEGTIQRGVRFKNQYADALNRAEQQFGVSQAVILGILGVETGYGANKGSFITRDALATLAFGYPRRAEYFGDELAALIAWTYKEGYPTNSIVGSYAGAIGFPQFMPSNISKYGVDFDGNGHIDLRNSAEDAIGSIANYLAKQGWQRDQPIGFMARYTGSNPESIIAKDLTQPTPYGALKNQGISPLNPLVKIDDLDMVNVIQLQDYNGPIYYLTYPNFQVITTYNKSRMYATAVWLLGTEVASR

ST25 4190 cg105_1299

MNALNTLLTQSNTKETPVSLPVQLKAKYPLVQTFARQIAEHRQIIQNILAGKDQRLMVITGPCSIHDPVAVLEYADRLQKLQEKVKDQIFIVMRAYIEKPRTTVGWKGFMYDPNLDGSSNLQLGLEKSRELYLQIIEKGLPIASEILSPMATGYFDDLLAWGAIGARTSESQIHREISSHMPYSIGFKNGTDGSIQIALDAIQSAQNEHQFLGMNQQGLPSVIQSAGNPLPHLILRGANHGPNYDLASIQAIREKHKQNLPALVIDCSHGNSGKDPLRQPEVLQQIVAERLKTQVKGIMIESHLVDGNQKISCEMTYGQSVTDGCLGWDKTEQLLLNVAKQLKASELAHSA

ST25 4190 cg105_1300

MPYYTMPDQESLFVRRVGRGEPVLVLSGLGMQSWQWLPFIYANRKEYEFIIPDWRGFGGSKNCAIPEQDAISSHWQDVAHLIEQLKLDKFILIGYSMGATTAMHGMKHAHLAQQLKAYLHIDQTPKISVDATWQYGLFGPLHAQFKQLLNDIYQLLSIHKNAKYLYELSLPDRQKLVRLWVDFIELQSSNPFSPFVFKNCIKTTIFTKISSSDSAFRLLILVCRKLSFPQ

ST25 4190 cg105_1301

MSLPHVLLTSLLERPSTGFELARRFDRSMGFFWNATHQQIYRELNNMLKKGWVSTLENEMDSGRKKTYQVEQLGRIELASWMTQQSEPAQLRDDLMVRLRAEAQLGNNQILPELLRHLGLHQEKLKLYQTIYDKDFKDSDDLNNRVLYIHKMILKLGITMETEWIKWLEQVIPQLKHFAQDNVSGE

ST25 4190 cg105_1302

MSLYPHLLKPLDLGFTTLKNRVLMGSMHVGLEEAPGGYERMAAFYAERAKGDVGLIVTGGIAPNQAGLTFAHASKLDSTEEAEKHKVITEAVHAAGGKIALQILHTGRYSYQPEIVAPSAIQAPINPIKPKAMTSAEVQQTIDDFANCAKLAQYAGYDGVEIMGSEGYLINEFIAARTNHRDDEWGGSYENRIRFPVEIVKRTREIVGENFIIIYRLSMLDLVEGGSTLEEVIQLAKAIEKAGATIINTGIGWHEARIPTIATKVPRAAFTWVTEKLKGEVSVPLITSNRINTPEMAEHVLASGHADMVSMARPMLADPDFVLKASEGRSDEINTCIGCNQACLDHIFSMKIATCLVNPRACYETELIFKEAQNQKNIAVIGAGPAGLSFAVYAADRGHQVKIFEASHQIGGQFNIAKTVPGKEEFYETLRYFNRQIELRPNIELVLNHPATYEELSQSDFDEIVVATGVTPRQLQFEGIDHPKVLSYLQVLKERVAVGQRVAIIGAGGIGFDTAEYLTHEGESGSLNPEKFYEEWGIDTHYEHVGGLKQPKVEASEREIYLLQRKASSVGAGLGKTTGWIHRTGLKNRNVKMLAGVQYDKVDDQGLHITVDGKPTVLEVDNVVICAGQESFTAMYDQLKADGKNVHLIGGAKEAGELDAKRAIRQGAELAAVL

ST25 4190 cg105_1303

MTLEITKQSLEKWHHMIQSNDLSNLNDLLADEVVFHSPVAYKPYEGRQVVFFILTNVIQVFQNFTYHREFYTEDGENVVLEFSADVGDKSLKGIDMIRFNEQGKIVDFEVMIRPMSGLAALAEQMGIRIAQFKPQ

ST25 4190 cg105_1304

MWLKLSTLFLLPVLFIQGHKVRKNTPRLAEAKGEREGRAGQGKSLSLLILGDSAAAGVGVENQKDALSGAIIQELQNEFSLQWKLHAKTGDTTRQVFNALQHLEEQKYDVIVTSIGVNDVTKLTSAKSWIKQQKQLFEHIQKRFQPKLIIISGVPPMQHFPALPNPLAWLFGQYAEQMNQKLQQWLAPQSHFKFLEYDIETFQAMNLPMASDGFHPSKDVYAVWGRQVAALVRQSFNS

ST25 4190 cg105_1305

MGISVFNKIKDFGSELFDLVLGAEHPKVYYHPQGKIKDILDKLPQLKQKYRPTPWLTNSHVHLLYFDLIRKQTIKLAYDRIDQLTMSDGGITAIAWYGYNLPPDTPTIVIMHTITGTPESMRELVRDLNQYTGWRIALCLRRGHAGLPMPVPQISLFGSTSDLKEQLNHIQSLFPASDLYAVGSSAGTGLLVRYLGEQGEDTPFKAAFAMCPGYNTEIGFKNVHPFYSKMMTKKLFKYFIYPYQNTWSQIASLEKVLSATNLEEFEKEYFEMAGFEDYETYCKSINPIYVFENIKIPLMILNAEDDPVCSIKNLEPYKEPIQQMSNVAVVTTKKGSHCAFYEGWRSTSWAARLMADYFLIEHNK

ST25 4190 cg105_1306

LELNLKEDWSKKMKTALNRTPASFPVRRMDYNFEDTPRYWCNHEPSLTHYFTGLSTLFPEGESYFVRSVRALRAKAKENEILDREIGAFIGQEAMHSKEHHAFHVSAQQYGLNPESLEKATGIVLKAIEKVFSKKWNLLVTVGLEHYTAVLVVSMMQSVNELMTDNTIRNLWLWHSVEETEHKAVAFDLYQHLYGNGLDAYIPRVAVFTFSLVLITAFSTIYHIVLMKRDKQLTNFKTWRNFFNFASKQYKVLIPKFLEYYRFDFHPNQTDEKALVAATKVKLGISDRSPLLS

ST25 4190 cg105_1307

MANKKLLICAAIAAGLLLTACVKKETPKEEEQDKIETAVSEPQPQKPAKFESLESVDTQEAQVQEQPQVEVHREETANTTTEIRRETRPARSDESSQTQVAEQPKSETPKVEPKPEKKPEPKAEPKPEKAQSKPAAKATEPANTEDDAVAAAIAAATPALKN

ST25 4190 cg105_1308

MTHFIFVTGGVVSSLGKGISAASVAALLEARGLKVTMVKMDPYINVDPGTMSPFQHGEVFVTEDGAETDLDLGYYERFLRRAKMTKLNNFTSGRVYQDVLNKERRGDYLGGTVQVIPHITDNIKERVLRAGEGYDVAIVEIGGTVGDIESLPFMESVRQLMVELGHKRTMLMHLTLLPYIKSAAELKTKPTQHSVKELLSIGIQPDILICRTEYDVDADTKRKIALFTNVEARAVVVCKDAKTIYQIPRGFYEQNVDDLICERFGFTDLPEADLTDWDNVVEALLNPEYTVRVAMVGKYVELPDAYKSVNEALLHAGIKNRVKVQIDYVNAEELESQDVSILKTADAILVPGGFGERGTEGKMKAIQYARENGIPFLGICLGMQLAVIEYARHVAGMPEASSTEFNRSTKYPLIGLITEWLDERGELQQRSLESDLGGTMRLGAQKSELVEGTKTREVYGKAEITERHRHRYEMNNRFIEAIEQAGMKISGYSSAQHLVETVEIPEHPWFIAVQFHPEFTSSPRDGHPLFASFIDAAKTQHQKSK

ST25 4190 cg105_1309

MSQLKPQEVVRLGDIQMANHLPFVLFGGMNVLESKDLAFEIAETYIDICKRLDIPYVFKASFDKANRSSLHSFRGPGLEKGIEWLGDIKKHFNVPIITDVHEPYQAAPVAEVADIIQLPAFLSRQTDLVEAMAKTQAIINIKKAQFLAPHEMRHILHKCLEAGNDKLILCERGSAFGYNNLVVDMLGFDIMKEMNVPVFFDVTHALQTPGGRSDSAGGRRAQITTLARAGMATGLAGLFLESHPDPDKAKCDGPSALRLSQLEPFLAQLKELDTLVKGFKKLDTH

ST25 4190 cg105_1310

MSQIVDIRAREILDSRGNPTIEADVILESGVVGRACAPSGASTGSREALELRDGDKSRYLGKGVRTAVQNVNSSIHELLVGQSVFEQKALDEKMIAFDGTENKSKLGANATLAVSLAAAHAAAAEQKLPLFQYIANLRGQTTLTMPVPMMNILNGGAHADNTVDIQEFMIEPVGFTSFAEALRAGAEVFHSLKSVLKKQGLNTAVGDEGGFAPNLRSNEEAITVILQAIEQTGYKAGSDIMLALDCASSEFYKNGQYILEGEGNKSFTSNQFADYLAGLVKQYPIISIEDGLDESDWEGWSYLTSILGDKIQLVGDDLFVTNPKILQRGIDEKVGNSILIKYNQIGTLTETLDAIYLAKANGYTTVISHRSGETEDSTIADLAVGTAAGQIKTGSLCRSDRVSKYNQLLRIEELTKAVYRGKAEFKGLN

ST25 4190 cg105_1311

MFIIIISFLRLSGKRGIRQLSLFELAIILCLGSAAGDPMFTKDLPIAHALIAFIAILSLYRLVTWGMVKHKKIEDLLEGKALCVVKEGLLVYKDFQKQTYSHDEFFSEMRQQNVEHLGQVRTALLESDGILSLLYYEDEDVKWGLPLFPDAYRKAEVLKINTFYSCMKCGETKILNKLDQECSRCHHHSWAESLKTRRLG

ST25 4190 cg105_1312

MTIHRFAGTLQEFCMLLVNCHTIQKLNQSF

ST25 4190 cg105_1313

VAILQYQFWLGEGGYFPHQALMQQIQQQAEVNDELKERNRILAAEVFDLKNGTEAIEEHARLDLGLVKPHETFVQMSTISTHYKPIYIDPNAKVDLETNETPPSPDIPD

ST25 4190 cg105_1314

MRHLHHQTSQTKLWAVIPAAGSGSRFSKTELKQYQYIQDATVIEHTVKRLSQLPLTGYVLAIGKQDTFASTLSFQDKHKAHFCNGGVERVHSVLNALNYLSQIADEDDWVLVHDAARPCVTFECLNTLIKNAIETNQSAILAIPVRDTLKQVNQEQQIDKTVSRELLWQAQTPQIAKIGILKKAIETALKNNLTITDEASALESIGESVLVVMGRSDNIKITYPDDLELARLILQSQN

ST25 4190 cg106_1315

MYLIDRPIITKTYNNLESIEIENDSVYLYGQSLEDRADISTLIQTASKNIKYIKLNINNEETTLSVHLKEILTVNLNDENSINNFFSHFQEKNFYLDITGLTHSAWAPLIKFFSKHNELSIKVIYIEPSSYSRKLAPLDNEFYNLSSHIKGVAPVNGFHTLIDSDEFTFVPLLGFEGARFNLINNTVEPKSKDSIIPLVGLPGFEPWYVFETIRGNRQILKITESYQWIDYIPSDCTFSCYYKLEEILNIKKNLKVAPIGTKPHALAAFILALNYPNNVEIVYDHPIRTSKRTDGASKVHVYDIKHLFRYPPSINLNLADRRRERRERRT

ST25 4190 cg106_1316

LGWRKRSTLRENEILNSPADYFKIDMRTVFQENSFSVFAKQVVESRLPELKKIGGIEKLLPSLVEEEEANILIGAEQEAQIRKNLASSLPKKYLKLLDQKSIGHIQFIDYMSRSREEPFIENLINWLENEGEWKDKFNNYFHAYLFSIRKGKSGIRKYYTGWDTFILLANNNIRYLIELVNKSIQIYLDNQSIAQIQYIDFDSQTKATQIIAKKNFTELEGISINGATLTKLLLGLGRLFEVLASNSEGHAPEVNQFYLNKTSEENQNVEAILTHAVMHLALIRIPGTKIGDITSTRDFDYMIHPIFAPFFVFSHRKKRKLALEATEIITLIDDPKKALKHLIQKNKRPAEVLQSSLPDQLDLFGEYLNVSN

ST25 4190 cg106_1317

MNLTYQELKDGNEKLGLYKAEWLSDKIFDYFSEPGYFHQLVNSRPCIIVGGRGTGKTTVLKSLSYEGQSRLNKESSPSEWNFYGLYWKVNLNRITSFVKRGLSDNEWQPYFIHYLNLILCHKLCQFAVWYEKTQDQKLNLDERLLRKAVTTLNIPIEYVQNIEDLEDEIDILIAELESKLNTISSDDKIFLTMLGAPIDRVSELLLQTTELNGKQFVFLIDEFENFEDYQQCIVNTLMKQINHLYTFKNRCQRVGLEKTFNFKRK

ST25 4190 cg106_1318

MIDEDEIERHLRISKDMWSDLVMLQSWPQQRYFNPRGWVQNFRKSEIPYALRLIDNMTYYSDEMSKALFKSAFHRLCKIILQNETCVHYNQASINWQTFKNSAYIIPISGETPNPSDSGFRYARYARDLCKIEEANILSLEQAIRTIQNGRPAKLIFVDDFLGSGEQFLKTWSKKFDIGGSYKSLANSVCSNSRIEIYICTIISTQYAIENIHQVLPNAVISPAHIFTPYHSVLSEHSYIWRDDMKTEGPQFIQEISSRLGIPDLNGELGENDEICWRGFKKLGLCVAFQDSIPDASIPLLNFSSEEWQPLIRIG

ST25 4190 cg106_1319

LEHLSWKWWSHNKNNIEQAQLELIDIWHFGLSDLLQKYGTSEEVINFLKETHNEDEPKIENVTDIHLLIERFALSTLKNKSFDISLFFNLCEILNLEFNELYKIYLGKNVLNEFRQKNGYKTGNYKKIWNKHEDNYYLFKIINELDVDKSEFCHLVYEKLDNQYQESFKS

ST25 4190 cg106_1320

MDVQSQLLFIGSSAKALKLWWQNEKGLADW

ST25 4190 cg106_1321

MVQKEKLQDKVVAMVEYDHDTSTIDKLTTLYELHTNLEGQYYLLFKAIFETKNSYPNAYQTAVRYRTWLTNEIYSQLRLLKADVSFTDARLFLYMIDGAILQLLMSEQADENERLLMRFLRQFIVNSVFQYS

ST25 4190 cg107_1322

LTINVNSTVDAYISSLISYLFEKSYPDIYKQIVEQIIFMRFDELSKQDFHFIIDQINRLSSSLDYLPDDVKNGIYWWNKLIKPLVDRDYRIVL

ST25 4190 cg108_1323

MTNGLEDFSYDEVSTENSTHSEQAKKVLGHFIQGRIVSKTARKQPVYNPATGEISKEVEIADAQTVNEAVQAAEQAFPAWRDTPVIKRARVMFKFKQLLEQNAEKICALIGQEHGKISHDAQGELQRGIENVEYACGAPELLKGEYSKNVGPDIDSWSEFQPLGVVAGITPFNFPAMVALWMFPMALVCGNCFILKPSEKAPSVVLYLAELLKQAGLPDGVFNVVNGDKEAVDALLHHPRIQAVSFVGSTPIAEYIYRTATSTGKRCQALGGAKNHAIIMPDADIDNVVTSLLGAAFGSSGERCMALSVAVAIGDEVADVVIDKLTQEMKKLKFGNYADASNDFGPLITQAHKDKVQAYIQSAEQQGAKIVVDGRDAKPAGYEKGFFVGPTLIDQVTPNMTSYQQEIFGPVLQVMRVNTMQEAMQLINDHEYGNGTCIYTRDGEAARYFSSHIQVGMVGINVPLPVPVAYHSFGGWKRSLFGDLHAYGPDGVRFYTRRKTITQRWPSAQVREAKQFSMPTLN

ST25 4190 cg108_1324

MVMFDTDKFSDSEHTLDAVQTNNNMHINYQAHWMPFSANRNFAKDPRMIVGAKGSYLIDDSGREIFDSLSGLWTCGAGHSLPEIQQAVSAQLGQLDYSPAFQFGHPLSFKLADKIVQHMPGKLQHVFFTNSGSESADTSIKMARAYWRIKGKPSKTKLIGRARGYHGVNVAGTSLGGIGGNRKMFGQLMDVDHLPHTLQPDLTFTKGCAETGGVELANEMLKLIELHDASNIAAVIVEPISGSAGCIVPPTGYLQRLREICDEHDILLIFDEVITGFGRLGTWTAAEYFGVTPDILNFAKQVTNGAIPLGGVVASHEIYSAFMQQDLPEHAIEFTHGYTYSAHPVACAAALAALEILEKKNLLAQSAALAPSFEKMLHGLKGAPHILDIRNCGLIGALQLAPRDGDATIRGFEIGMKLWKEGFYVRFGGDTLQFGPMFNSTEADIDRLMNAVGDALYQVN

ST25 4190 cg108_1325

MKPKKLNQVTDFDIKLLKIFKTVCDCHSFTSAESILGISRSAISLHMSDLENRLGIRLCQRGRAGFALTDEGREILEYIEVLTAAIEDFRSKVNQMHNRLKGEFNIGIINNLVTMPRGYITNTLTQLAEEHAEVIINISMSTLSDIECRVLDNRLHAGVIPLVTPLSGLDYFDLYSESSFLYCGKNHPLFNQCSNIHLNELKKWQAILPNYAITSEAAKLHQLLDCKATASDREGIAFLILTGKFLGFLPDHYAKKWVEDGVMQPILKDKMHYSTPICLITHKGKNHNNILKTFMEMLEKRIANN

ST25 4190 cg108_1326

LYADEFQRNGQMIYSSPKAIYNVTADEIESSLAEDVVQTYDLNSFGLFTKKTYQKQNNGWPEGYIVASQGSQITTAQFNDSCSLNSDNVSFDYEKINVSGKKVADIFPPNIINSIPKHSDYIYISDQFSRILKDNQTAFANLVNSNATFPSGSFVYVPKSVIYNNTEFYLFDSSLTDFKTLAEWQQKLYPNFNYKFDTVAGYKVTYFVDSAGNPIFDNGKDPAIEMNGKIYDGEWQVKGNVISETYGAPPTTWNTNYQSKSEFALYNKASYDFLVAQIQTYYK

ST25 4190 cg108_1327

MVQKEKLQEQMVAMIEYDLSTPAIDKLKKLYDLHTDVEGPYYLLFKAIFEIKNSYPNAYQTAVRYRTWLKNEIYSQLRILKFGASFNDAKLFLYMIEGATIQCLDKNDAHEGNKALDYFLLKIGLV

ST25 4190 cg109_1328

MFRKALLCLSLISLVGCNDDDKTETTPTTPEYQLPKILVVGHRGASALRPEHTLASYQKAIDDGADFIEPDLVSTKDGVLVARHENEIGGTTNVSTLSQFADRKKTKNIDGVDLTGWFTEDFTLSELQQLKARERIPEFRPANTAYNDLYPVPTLEQIIELAEANYKKTGKIIGLYIETKHPTYFKNQNLAMEDTLLKTLAKYKYTRDIAPVYLQSFEVQNLKDLKRELDLHKTLKHAQIIQLYDSKTSRPADFVESGDTKTYADLATAQGLKDVAKYANGVGPSKGYILTFNNDGSYKTSTFISDAHTAGLKVHPYTFRPENNFLPAPLKCSPDKPAERCPTGALKEFEAYFKAGVGWRLYR

ST25 4190 cg109_1330

MLSKFFIQRPIFANVLAIIVMAFGIFSVMNLPVERYPDIAPPKITVSANYSGADAQTVEQSVTQILEQQIQGIDHLLYFSSSSDSSGRSRITISFDNGTNPDTAQVQVQNSISGVIRRLPDEVQRQGVTVSKSLGDTFMVIGLYDSTGKTGNIELSDYLTTHVVDNLNRIEGVGETDVFGSQYAMRIWLNPDKLKQYNLMPSDVANAITAQNTQVAAGAIGDLPVIDGQYLNTKVTAGSRLKTVEDFKNIVVKSNKTASYVYLKDIARVELGAENYQSFNTINGYPAAGLGISLSSGANAIQTSKLIHQTLDQLTTKLPAGYKIVYPRDNTPFVQESIKEVVKTLVEAIILVILVMFLFLQSWRATLIPSITVPVVILGTFAVLYVLGFSINTLTLFALVLAIGLLVDDAIVVVENVERLMHEQHLSPKEAAIESMGEISGALVGITLVLTAVFIPMSFLGGSIGVIYRQFSITLVAAMALSLIVALILTPALCALILKPNPQPQRWAVWFNQKIEQLKNQYIKLVQTSIHYSKSVIVIFVALIAVFTLFYNGLKSGFIPKEDQGILSVQIKLVDSAPISQSQKIGEQVRQYFLTQEDKNVDLVLIRYGRNYSGTGQNLAQGFIALKPWDVRTGKENSAEAIQKRAMKYFSHFNNAQINVTLPASVNGLGQTDGLDLWIQDLNGQGQDFLDSTFRQLQAQSKNYSTFENFDKQSTNSKANLNIKIDQKQALANGLQLSAINNTLSSAWGGTYVNDFIDRGRIKRVMIQGDAEFRSKPEDLYNWSVRNDQNEMVPFSSFANFSWGGAPEIVKRYMGYSALQLQADVASGSSSGQAMKDVEQLVNQQKDIGLAWTGLSFEEQKSTNQAVWLYLISAGFIFLCLAALYESLSIPAAVMTSIPLGVGGSVIFSYIFGLPNDVYFQIALLTTIGLSCKNAILIVEFAALAQEKGKNAIQAALEGASLRLRPILMTSLAFGAGVIPLVFAQGAGAVSRQEIGISILGGVMFGTVLVLFFIPVMYVLLRSLFKSKAST

ST25 4190 cg109_1331

MPQEKKLTQQQVIALERDLAKFATMMDSAVRIPFTKQGIGADAALSTIPIAGDVAGFALTCYAIYKAKQIGVPQHKLTPVIKLAVVDAVVGFVPVAGTIFDIFIRPSRKALDVVHTHILEEYAIQSDIHVIHPYLHEKLEKKQQQSAFWRNPVVSWLWLHIPDLLGAFVLLLIGLAVWWGASYLWGLYQTM

ST25 4190 cg109_1332

MMRIGLFLLTNLAVLVVAGIILSLFGVGSYHGAGGLNLGNLLVICFVFGMVGSLVSLFMSKWMAKKTTGTELIDPNAPRNQAESWLLQTVAELSQRAGINMPEVGIFPSYQSNAFATGWNKNDALVAVSSGLLERMNKDELRAVLAHEIGHVANGDMVTLALIQGVVNAFVMFFARVVGDFIDRNVFGRQDNEAPGMGYFIITMVLDIVFGILASAIVMWFSRYREYRADEAGARLAGKQAMISALLRLQAETELPDQMPKEMKAFAIAEGKEQGFSLAALFQTHPTIEQRVAALHQLDCP

ST25 4190 cg109_1333

MTTFSVGITPTSAGQMYIYQDKNGSTLLTNRKSYDHSLKKVKVTYYPDSNIHSYSNWGTSEASVLPSYSKNKNAFDHIIKQAAQQHGVSEGLIKAVMHTESGFNVNARSPVGAQGLMQLMPATARRFNVSNAYDPQQNIFAGAKYLSWLLKRFNGNTQMALAAYNAGEGNVDKYGGIPPFRETQDYVRRVTSRYQNLYSSGVGLSSFSNSSISAQAINQPAIPHSTSTQVSAQPIKYSSSRQIVTLPDGTYTDAPTGTYVTNNATAIAHIRIE

ST25 4190 cg109_1334

MYAPVESNEGFNFKPELPTSSAYYRLLKKLRRQVGHAIRDFNMIEDGDKVMVCVSGGKDSYTLLDILLQFKRIAPINFDIVAVNLDQKQPGFPEDVLPRYMEENNIPYYILEKDTYSITKRLTPEGKTYCAVCSRLRRGSLYGFAQEIGATKVALGHHRDDIIATFFLNLFHGGSLKAMPPKLLSSDKKNILIRPLAYVEEKDIIKYAELRKFPIIPCNLCGSQENLQRAMINEMLREWDKQYPKRLHSIFGALQNVSPSQLADRDLFDFEVLDSQRELDFKDPEELKKRLDVVNLSFAAE

ST25 4190 cg109_1335

MPAFLTDDWFATVEKLTAEAGDLNLPPALANLAINLVVTDASGNTELALDGGKIQKGLSSNAKTTLNMDAETLRKVFLEFDMAAAMQAFMTGKIKVQGDMSQLMALQTAKPSQEQKDLFKKVLEQTA

ST25 4190 cg109_1336

VVAVESPTTVFKEDQFLHGFGYDLARNYAQSLNVKLDFKIVTDNATALKWVQQGKANLAMTTASLSSIENKGLMSFSASCGDIVNLQKNGLNPNLSWVFKQADDPLTQTASGFVCQSKQNGLTQQLASFYNRNVVKPEAWSTIQRDLSARIPIYKASFKQSAAQYDLDWHLLAAIGYQESYLKPESVSPTGVRGLMMLTNSTARAMGVSNRNDPAQSIQGGAKYYDLMLSEYDDIPFPDRNWYALVAYNMGPGAVNQIQKRLQAQGKDPNQWVNLYNYLQSNKTRNGRYKQAVQYVTRIRAYLEHIKTAQTRINI

ST25 4190 cg109_1337

MKSNLITRSGHDKLVAELKQLWHEERPEITKKVNWAASLGDRSENADYQYNKQLLRKIDRRVRYLGKRLEELKIVDYSPEQEGKVYFGAWVDIENEEGEQKTLRIVGVDEIYDHHPQHISIESPMARALLSKEVDDEVEVHTPLGKKLWYINAIRYEKPKNDEI

ST25 4190 cg109_1339

LEGELRSPVSFKHVEVSLFIIFENGLKELNSGAGMSQHIINPKEIPLAFQTAWNKHDMQAFAALFDKEATFVNRFGHYVKGVDEIIAMHQPIHETIYRDSTLENELIDLIPMSEDICISHFWSRLTAGVAHPQGPHQIDTLILTVLTKKNHSWYIQALENVTLTNPRTGETILRNI

ST25 4190 cg109_1340

MNQFVTNTKNVIRGKYHPEFLQNEVLADIFAHTAQTLPDKTALIEADKTLSYGELYQQALIMAQHLALKGVKPGHIVGLWLPRGIELLKAQLAICLSGAAWLPFDMDTPADRIAVCLADAEAVGMITTDEWYEHLAEVPQTKWTNTELQKTLSESVSLAKTTPDQPAYIIYTSGSTGKPKGIVITQKNICHFLRSENSILGIQEQDKVYQGFSVAFDMSFEEIWLSYLVGATLWIAPKSLVSDPERLCQTLKHEQITVLHAVPTLLALFPEDVPNLRIINLGGEMCPDSLVDRWALPHHQMFNTYGPTETTVSASLELLERGKPVTIGKPLPNYGMLVINSERELLEQGETGELCIFGPSVAQGYLGRPDLTADKFIENPWAMSVEEELLYRTGDLAKIDEFGQVHCLGRADDQVKIRGFRVELGEIEAALCDIDGIGTAAVILRPEDGIDQLIAFIAPEIDAKQAIEIKELRHNLSQRLPPYMVPNRFEIIEEVPRLLSGKIDRKALKARPLTSVVDRSESDQPQNPAEEILFEILNRLFPNMPIKLDSDFFDDLGGHSLLAAVLISNLREHAEYSHLTIQNLYQARRVGAIAALMLEQPEPTLFDSQIGQDNPRNQTYKWLCGIAQLVTIPVLISINILQWLAPFFTYHYFTGGTRDSIPYAIALSLLVYVSVIMSSFVLSITVKRLLMLGIGAGRYPLWGLTYFRWWLADRISNISPVYLLSGSTLLNLYLKALGAKIGHDVTISSVHIRMPSLLTIEDGVSIGSQVNLENAKVEHGHLVLGSIHLKQDSYVGSYAVLEENTVLEKQAHVNALTSIEYDTVVPEGEIWDGTPAQKIGHIDEQAKLPERPKLSFIRKIAEYGYYGVSALVIACLFFIPIFPSFLLVDWLDVNVFNINPNNHLQIALYYFILAIPASAMMMITAVISSGLRKIALPRLETGTYAVHGSTYYRKWFAAQILETSLQTLHGLFATIYAPTWFRMLGAKVGKNTEISTATGVIPEMLTLGEESFIADAVMLGDEEIKGGWMSLKATKIGNRSFVGNSAYIADGTVLPDNVLIGVQSKTPDNREMYDGQTWFGSPALLLPAREAAEKYPDHLTFKPSIKRRLMRGFIEGLRIVLPAALAIGVGYMIVLDVIDVINNYNIETGLVALTLAGLLYGVGCFLIVALLKWILIGRYQPRSAPMWTMFVWLSEGITSLYESVAIPNFLNYLRGTPMLPFFLRILGVRIGKDVYMDTADITEFDCVSIGDRAEFNSFSGPQTHLFEDRIMKIGQVNVGHDVVVNARSIILYNANVSNHAVLGPLTLVMKGENIPAKSAWIGSPAVPWVHK

ST25 4190 cg109_1341

MNIIIRDEQPEDIKNIEALTKAAFLNEEHSSHTEQFIVNSLRKHKQLTISLVAIEDDLIIGHVAISPVTITSGDTGWYGLGPISVCPKKQGIGVGSLLMNAALQKLKELGGKGCVLLGDPNYYSRFGFKSYPDLNLPDVPNEYFQALSFINHIPSGEVTYHDAFTATE

ST25 4190 cg110_1342

MPETNGVWHWSNGENWLQAASLDQLIQDLQIHQGKEATVFFPSRHAQMLQQTMAKSHYKQLGADGVKYLLEEFVTLPIDHMKVVHHFHADRLTVLGVAQGMVETWQHSLALLPTKLVALLPDFLVLPEPQAQQVILCNIGHQLLVRENKWLGNSIDDLGLFLEFQSAETHYQYSGLAAEQLESLEAASSAEQRSEFVYQFQPLDKTKQHPFNVLPKSKGQERTFSNYWKACAAVVLAIIVVQFSYDLLRWVKLKKVADQTAEQAIEQYKYWFGPSSRVTEQNIKGQFESHLRMSQQGDTQALSLLSRVGPILMQRQILAQQLNYDASILTMALKAKSADDLQALTQQLNQQGFKAELGNVQADGSGAIGVVKIQ

ST25 4190 cg110_1343

MLAQLQNRFDQWVEQIVQYLDRLTVRERIMVVFTTIFVVVVIVGYSLWKMHSLAEQQQKRLNDLKDLMVWMQSNAVTMKPANELELDKSGKIQRVAQQQGLTVSSQQNGEQLQIVVTHQNYAILANFLTQLAQMGLSIQKMEMVSSEGQIKLTATVQ

ST25 4190 cg110_1344

VIGMLYSLARPMLFSLAPERAHELTLSMLDKAHKLGMMRQTVEAKPTTCMGIEFPNPVGLAAGLDKNGAHIDALAGLGFGFIEIGTITPRPQIGNPKPRLFRIPEAKAIINRMGFNNDGVDKLIENVKASKFRGILGINIGKNADTPVEKAVDDYLICLEKVYNYASYITVNISSPNTKNLRSLQSGDALTELLQTLKARQLELAEQYNHYVPLVLKVAPDLTAEDVEFISAQLLDFKIDGLIVTNTTLSREGVENLPYGNESGGLSGAPVFEKSTECLRLFAQTLKGQIPLIGVGGILSGEQAAAKQQAGATLVQIYSGLIYTGPTLVKQCVEAMT

ST25 4190 cg110_1345

LVGWIFALIMGAKYAVLLAPSMSGLSQDPVVQKIAAFAFIALLIIVLTWIVTAFLNGLLKSLKLGPLNRLAGGAFGSLKGLLVVLITMQGVGPWVESSPHWKQSKFIQFLLPYAPLATELSKDAASEAFHQITSGGGVTRTSPKPMEESEETELRPDHSTKNPFY

ST25 4190 cg110_1346

MCGVVGIAGKSPVNQMLFDALTMLQHRGQDAAGIVTCHEGRLFLRKDNGMVRDVFHTRHMRALLGNYGIGHVRYPTAGSSSSAEAQPFYVNSPYGITLAHNGNLTNAEEIHDDLFKTDLRHMNTDSDSEVLLNVFAHELQKIGTLNPTPEDIFHTVSRVHERCQGAYGVVAMITGHGLVGFRDPNGIRPLIYGSRVTEQGEMEYIIASESVAITALGFKIERDIAPGEAIFINADGELFTKQCAANPKYRPCIFEYVYFARPDAIIDGISVYKARLKMGEKLAHKILRDWGEDHDIDVVIPIPDTSRTSALELANILGVKFREGFMKNRYIGRTFIMPGQQQRKKSVRQKLNPVELEFKGKNVLLVDDSIVRGTTCNEIIQMARDSGAKKVYFASAAPKVMYPNVYGIDMPAKTELIASERSVEEIQEIIGADRLIFQDLEDLKNAVRTSKVPTLTEFDCSVFDGIYVTGGIDADYLNNLEQKRNDSAKKKKMVILMSILMQHQ

ST25 4190 cg110_1347

LKEVSHYLIENKNMAWSITMCLVAVLLTIFILNLILGLLVHFFDYSEPIWWHALSPYFIALLLFIMVWSVVTELYILRKGGHSLAKQLKARRLVKGESTPEEHAALKITEHLAQTFSLNVPTLYVLPDEVGVNALTAGFHSNDIVIILTWGALQNLDELELYGLLGHEFNQILSGEAVENTKLKILYSGLTTFSQWGSKLAKQGFKRYSPGYKHKFETVFVAVGGVIWLAGSLGVLITRFIKYLTLSGRTFRNDQKTMRLLKNSTNTQTLLRIYVHHSGSQIHSAYSESIAHMCFANSLSPQSWMNIHPSIRERIYELNPTLLQDLQLENLKKLRNRPLFSLFHVLEESETEIYTPWSSPQPLPLLRLSPISFALNDAIKPLSSDIRRNKKRPELIQRALQTATGSREVMVAILMIRQYREFIPQDAPVSHAIVDALLNLDGRIHIQIFHDACKNIGHMPASIARQFLTKLALIIQEDGEIGLLDALLLERVKYELNLMPLHLPTAFEEVKPQIVRLIDALLHVQQINSPNQLEVRERILRSLLNPDEMHIYDEISDEPLDLAEILNDIAGLLLRDRLSILAIAEMCLWSDRIITQDELDVLELLYWRFGFESDEIVEQMQKKNSVMII

ST25 4190 cg110_1348

MIGQQRNILATLGIDVWIPRTQVCQKNNAHTLWRDQVVEPHESITVPTIDVPAFEKKNTQPQVLEIPKVVEEPSIVVAEVSQPEKLVEKPKVIEQETIAPFELQAYCLEKCVIFVDVTALETEEKQLWANIQKVKVGQYSELRWPFPLVAYQDQRGVGSYIQGFLDAVAAEKKILCLGKCTYIQHANIIHLASLKEMLDKPLLKKRLWQLMQDNNE

ST25 4190 cg110_1349

MKKVVVFSQIDEEILSRLQQDYYVVVLNPKLGDINEQIRQEVVDADGMIGAGRLLNESNLSPAQKLKIISSVTVGYDNYDVGYLNQKKIWLANTPHVLTETTADLAFTLLLSAARKVPFLDHWTKQGEWKRTVGPQQFGLDVFGKTLGIIGLGNIGAAIARRGFYGFNMNIVYHNRREKPELAEPLKAQYLGLEELLQQSDFVVTAVDLNAESKALMGKAQFELMQKHAIFINIARGSVVDEQALIEALQNEVIFAAGLDVYEKEPLQESALFNLPNVVTLPHVGSATAETRKKMANLAYKNLVEALEDKTPRYLVNPNFV

ST25 4190 cg110_1350

LPVILSLALITNASYANPFDPKPASGQVEIPNIGSGIGLLDQQKEKFIGEKVFREVHKQMPVIQDVWLEDQFFQVFSNILSETQLGQPIALVVIKDPQINAFAVPGGLFALNTGLISSARNIDEIAGVMAHEIAHVSQRHFSRSEEAFKGQTLLSLAGLLAGVALAAQAGGDAGAAVMLGTQAALLDKQLTYSRNQEREADRIGMQYMYAAGYNPQSMADYFETMHRATSRVSFLPDFWLTHPLTSERMSEARLRANQMPKVKSRIYDVDFEILKWYTMVVAGEATENQLQSLASQKNLAGLLALSAFYLKQGDYTQAQATLEQAKSSGKPLVALIQTDIYLGQNKLDQAYNSIASLQMTMPENKAFSYKLAEVLLRQGKYAQVQTLVQRFINKNARDIQGWQLLQQAANLDKNSPLRAVNVLRYRAEAQYWSGSEEDAIKSMLHAQRLAKGNQAMSARIDSRLKQMQDERRMKI

ST25 4190 cg110_1351

MTTLKNQITDALKTSMRAKDMATVTVLRSLQAAIKQIEVDERIELDDAQVLAVIEKQIKQRKESIKAFEGAGRDDLASKEQAEAEVLSQFLPEAMTEEELDSLIEQTIAAQEATSMKDMGKVMNSLRPIIAGRADPAQVSAKIKAKLA

ST25 4190 cg110_1352

MPQVKLKEGEPVDVAIRRFKRSCEKAGVLADVRKREFYEKPTQERKRKKAAAVKRYQKKLARESVRTTRLY

ST25 4190 cg110_1353

MIVLGLETSCDETGLALYDSELGLRGQVLYSQIKLHAEYGGVVPELASRDHVRKLIPLMNQLLEQSGVKKQEIDAVAYTRGPGLMGALMTGALFGRTLAFSLNKPAIGVHHMEGHMLAPLLSSQPPEFPFVALLVSGGHTQLMAAHGIGQYELLGESIDDAAGEAFDKVAKMMNLPYPGGPNIAKLALSGDPLAFEFPRPMLHQGLDFSFSGLKTAVSVQLKKLNGENRDADIAASFQEAIVDTLVKKSVKALKQTGLKRLVIAGGVSANLRLREQLETSLAKIKAQVYYAEPALCTDNGAMIAFAGYQRLKAGQHDGLAVTTTPRWPMTELTIPE

ST25 4190 cg110_1354

MKKQLPKISTTSKALAKSLLLAQGVLDQAKKYSTLPFTQTHIIRPRIDEKYYSWTHYGIFFPLLPEPHRYLNIMILIGTPGALAFDHDDIITGNPRKTATFFSSTAALEQALLKAYIIPEDTKINKDGALIELGQEILLQGKFPHIHINGHYNGFDFDFDIDITSHVSWFIKTPIYDHFSLLAKFKGFLNYQAKCIETQGLCTYEYARAVGPHSITNKLIPDAYKLPLDFFTYQIINLNEATQLLLTKADIAGQTAAYTLHIRHLDQPAEIYTDVSFDIISHQVDDFVSPSGQKMRLPKYFSWIARNDAKQIILNIQAEIDCPFRYGHGRGYASSYIFTGHYFGNEVQGRGYIEYIDIENPKAFEDG

ST25 4190 cg110_1355

MATIDLPDNLVQTLSLVLNQLQQVLPEPKQETDFTAPAFRWENQQLKAIYTPKNIYLDDLKGIERQKEKIIQNTLQFLNGLPANDVLLTGSRGTGKSSIVRALLTEYAPQGLRLIEIERDDLADLPKIQKIIQNRPEKYIVYCDDLAFNAEDENYRSLKSVLDGSLQSGSTNFIIYATSNRRHLLPEFMHENTPVTKVDVPQYTELHPQEAIEEKISLSDRFGLWLSFYPMDQNLYLEIVEHYLDKANMPFNDEVRAESLRWCQMRGQRSGRAAYQFSKHWIGLNALKDLSNN

ST25 4190 cg110_1356

MFEKIRSDMNPHQAYRIKKLQRQLDMAESYEEWKSFALKLDEETGAQEWKFDNSSPYFDAELISYRYTLLKRYRQQHRTLDLIYLLKEGLTYDIANIGHPMLFAATHVGTKKLIEDYIEEVSQSLAYIASSECITFQRKEKIEFFENCEKAYGQPALMFSGGATLGLFHTGVCKALIEQDLMPKVLSGSSAGAIMTGMLGISASEDIQNLLNGEQFFSDAFHFRKLRELIKGNGGIADVHYLKKFLIENLGDLTFEEAFKKSGLNINVAVAPYDATENPRIMNAIMTPNVLVWSAVLASCAVPVLFPPVRLTSKRYDGEHTPYMANTKWVDGSVRSDFPQERMARLYNLNYTIASQVNPHVVPFMQDDARRFRKDVLSWPERILRRQGKVLSMGLMDFTRQRLGAISPVRRLLDHGYGVVGQRYYGDVNIIAKYSLKHYAYTLQNPRPHLFKRLQREGERATWPKISSIETHARIGKTIQHCLEVLRFEEKKQQPESYYAEA

ST25 4190 cg110_1357

LPNVHAQSEQDFLHELQFYEDITYKIANWLLPFKIIEGSTVSQQLQFQGRVLVLPDTDCWFDDLDQKQNLKNINEKIYLTLVKLAELHELGWIHGDIKKEHFRKFKQELYLIDFEKTRLISSPDPITDATPRYMAPELFHGANKTVQSDLYALGIVLYEWLTQTRLQANSYHEWAVLHCQKLNVELPSSLQIFLPLLSGLLQKQQQNRFSNVHEAINCLKALST

ST25 4190 cg111_1359

KTDVSFTDAKLFLYMVEGTIIQLLSSGRVSERESVFEYFLRGLTSCK*KVLGALVVTKHGVMERCYYCKYMFIKIFAII*K*KKQFFKTRCI*KDKFLNIF*LLSL*AYIRTKNIFIRALQRFFSFS*KYLFEI*ELSICIFDCIHLSI*MYIS

ST25 4190 cg112_1360
[truncated: 3,210,793 more chars]
