# Supplementary material for: Genome organization of epidemic Acinetobacter baumannii strains
Source: BMC Microbiol. 2011 Oct 10;11:224. doi: 10.1186/1471-2180-11-224 (PMC3224125; doi:10.1186/1471-2180-11-224)
Supplement: Additional file 8 — Genomic regions, amplified genes, primers, amplicon sizes and cycling conditions used in PCR surveys. (none, title sufficiently describes data). [file 1471-2180-11-224-S8.DOC]

| **GEI** | **amplified genes (ORFs)** | **function** | **primer sequences** | **amplicon size (bp)** | **cycling conditions (°C/min)^a^** |
| --- | --- | --- | --- | --- | --- |
| **G08** | 4190_2042  4190_2044 | czcA, heavy metal RND efflux p  arsC, arsenate reductase | F_GTTATTCCAGCTGCATATCAG  R_ CTAATTCGTCATGACTAGGT | 747 | D(94/1')A(58/1')E(72/1') |
|  |  |  |  |  |  |
| **G63** | 4190_3267 | satR, streptothricin acetyltransferase | F_GAAGATTTCGGTGATCCCT  R_GTACCAGTACATCGCTGTT | 493 | D(94/1')A(56/1')E(72/1') |
|  |  |  |  |  |  |
| **G43** | 4190_1237 | mhpE, m-hydroxyphenylprop. acid | F_CGTCATCAAACTACAGTTGAG  R_GCTCCTAGACCAGCTGCT | 626 | D(94/1')A(58/1')E(72/1') |
|  |  |  |  |  |  |
| **G46** | 4190_0542 | salA, salicylate 1-monoxygenase | F_GGCAGTCATCTGTTCATCGT  R_CATGGCATGTGCAGCGTCA | 644 | D(94/1')A(60/1')E(72/1') |
|  |  |  |  |  |  |
| **G23** | 4190_4036 | dmpK, phenol hydroxylase subunit | F_GTACAGCATGACGCAACCT  R_GGCACTGCTTCAGTGCCAT | 249 | D(94/1')A(58/1')E(72/30'') |
|  |  |  |  |  |  |
| **G21** | 4190_1761 | tartrate dehydratase sub. beta | F_GGTAACTTGTCGTGACGTTG  R_GGCATACCTAGATCTTGCCA | 363 | D(94/1')A(60/1')E(72/1') |
|  |  |  |  |  |  |
| **G33** | 4190_1454  4190_1456 | tatB, twin-arginine transloc. p  ssuB, sulfonate/nitrate transp. p | F_ GGACTGATCTTAACGTGTGA  R_ GCGTATCTGGCAACACGAA | 699 | D(94/1')A(58/1')E(72/1') |
|  |  |  |  |  |  |
| **G03** | 4190_1240 | creC, sensory histidine kinase | F_ GTGGATCGCTGCACTCAG  R_ GCTCAGGAAGATTCACCTCT | 509 | D(94/1')A(58/1')E(72/1') |
|  |  |  |  |  |  |
| **G20** | 4190_3211 | benP, benzoate transport porin | F_ CTGGTCAACCAAGCTTAATG  R_ GTCATATTCACCAAGCTTTG | 571 | D(94/1')A(58/1')E(72/1') |
|  |  |  |  |  |  |
| **G51** | 4190_3291 | RtcA, RNA 3'-terminal phosphate cyclase | F_ GGTTCAGCGGGTAGTACAA  R_ GTGTGATATTGATGAGTAACTT | 464 | D(94/1')A(58/1')E(72/1') |
|  |  |  |  |  |  |
| **G18** | acb_1079 | dichlorophenol hydroxylase | F_ GCGATCTTCCTCAACATCTAT  R_ CCACGACCTGCTTTGCCAT | 700 | D(94/1')A(60/1')E(72/1') |
|  |  |  |  |  |  |
| **G53** | acb_2563  acb_2562 | siderophore-interacting p  multidrug resistance pump | F_ CTGGAGCTGTGCATGAGCA  R_ GGCAATACTCGCCACTTGT | 584 | D(94/1')A(58/1')E(72/1') |
|  |  |  |  |  |  |
| **G06** | abc_0533  abc_0534 | fabG, dehydrogenase  fabF, 3-oxoacyl-synthase | F_ GCCAGACGGTTCAACAGATT  R_ CTCATGCTCTGGTGAAGCAA | 918 | D(94/1')A(60/1')E(72/1') |
|  |  |  |  |  |  |
| **G11** | abc_0688  abc_0689 | glucose dehydrogenase  hypotetical protein | F_ GCCACCTAAGCTAACAAGTT  R_ GAGGCTTCAACTTGGTCATT | 1614 | D(94/1')A(58/1')E(72/2') |
|  |  |  |  |  |  |
| **G37** | abc_1895  abc_1896 | phosphoglycerate dehydrogenase  MFS permease | F_ GCTGCATTAGTCGATGCTCT  R_ GCCACCATTCTGGCTCTATA | 772 | D(94/1')A(60/1')E(72/1') |
|  |  |  |  |  |  |
| **G47** | abn_2549  abn_2548 | desC, d-9 acyl-lipid desaturase 1  lipocalin family p | F_ CTCATCTGAAGGAGTTGCTTA  R_ CCAGCATGAAGATCACAGCA | 601 | D(94/1')A(60/1')E(72/1') |
|  |  |  |  |  |  |
| **G32** | abc_1699  abc_1700 | hlyD, membrane-fusion p  ABC transport system, ATPase | F_ GCGACATGACAACTGCTCAA  R_ GGCTTGTACAATCCGAGTCT | 1101 | D(94/1')A(60/1')E(72/90'') |

**Table S5**. Genomic regions, amplified genes, primers, amplicon sizes and cycling conditions used in PCR surveys

**^a^** D, A and E stand for denaturation, annealing, and extension, respectively.
